# Leishmaniasis Worldwide and Global Estimates of Its Incidence

**DOI:** 10.1371/journal.pone.0035671

**Published:** 2012-05-31

**Authors:** Jorge Alvar, Iván D. Vélez, Caryn Bern, Mercé Herrero, Philippe Desjeux, Jorge Cano, Jean Jannin, Margriet den Boer, the WHO Leishmaniasis Control Team

**Affiliations:** 1 Department for the Control of Neglected Tropical Diseases (HTM/NTD/IDM), Leishmaniasis Control Program, World Health Organization, Geneva, Switzerland; 2 PECET, Universidad de Antioquia, Medellin, Colombia; 3 Division of Parasitic Diseases and Malaria, National Center for Global Health, Centers for Disease Control and Prevention, Atlanta, Georgia, United States of America; 4 Disease Prevention and Control Programmes, World Health Organization, Addis Ababa, Ethiopia; 5 Institute of OneWorldHealth, San Francisco, California, United States of America; 6 National Centre for Tropical Medicine and International Health, Instituto de Salud Carlos III, Madrid, Spain; The Australian National University, Australia

## Abstract

As part of a World Health Organization-led effort to update the empirical evidence base for the leishmaniases, national experts provided leishmaniasis case data for the last 5 years and information regarding treatment and control in their respective countries and a comprehensive literature review was conducted covering publications on leishmaniasis in 98 countries and three territories (see ‘Leishmaniasis Country Profiles Text S1, S2, S3, S4, S5, S6, S7, S8, S9, S10, S11, S12, S13, S14, S15, S16, S17, S18, S19, S20, S21, S22, S23, S24, S25, S26, S27, S28, S29, S30, S31, S32, S33, S34, S35, S36, S37, S38, S39, S40, S41, S42, S43, S44, S45, S46, S47, S48, S49, S50, S51, S52, S53, S54, S55, S56, S57, S58, S59, S60, S61, S62, S63, S64, S65, S66, S67, S68, S69, S70, S71, S72, S73, S74, S75, S76, S77, S78, S79, S80, S81, S82, S83, S84, S85, S86, S87, S88, S89, S90, S91, S92, S93, S94, S95, S96, S97, S98, S99, S100, S101’). Additional information was collated during meetings conducted at WHO regional level between 2007 and 2011. Two questionnaires regarding epidemiology and drug access were completed by experts and national program managers. Visceral and cutaneous leishmaniasis incidence ranges were estimated by country and epidemiological region based on reported incidence, underreporting rates if available, and the judgment of national and international experts. Based on these estimates, approximately 0.2 to 0.4 cases and 0.7 to 1.2 million VL and CL cases, respectively, occur each year. More than 90% of global VL cases occur in six countries: India, Bangladesh, Sudan, South Sudan, Ethiopia and Brazil. Cutaneous leishmaniasis is more widely distributed, with about one-third of cases occurring in each of three epidemiological regions, the Americas, the Mediterranean basin, and western Asia from the Middle East to Central Asia. The ten countries with the highest estimated case counts, Afghanistan, Algeria, Colombia, Brazil, Iran, Syria, Ethiopia, North Sudan, Costa Rica and Peru, together account for 70 to 75% of global estimated CL incidence. Mortality data were extremely sparse and generally represent hospital-based deaths only. Using an overall case-fatality rate of 10%, we reach a tentative estimate of 20,000 to 40,000 leishmaniasis deaths per year. Although the information is very poor in a number of countries, this is the first in-depth exercise to better estimate the real impact of leishmaniasis. These data should help to define control strategies and reinforce leishmaniasis advocacy.

## Introduction

Although estimated to cause the ninth largest disease burden among individual infectious diseases, leishmaniasis is largely ignored in discussions of tropical disease priorities [1], [2]. This consignment to critical oblivion results from its complex epidemiology and ecology, the lack of simple, easily-applied tools for case management and the paucity of current incidence data, and often results in a failure on the part of policy-makers to recognize its importance [3], [4]. Based on the World Health Assembly Resolution 2007/60.13, the World Health Organization (WHO) convened the Expert Committee on Leishmaniasis in March 2010, which subsequently issued the first updated technical report on leishmaniasis in more than 20 years [5], [6]. Both the WHA Resolution and the Expert Committee report highlighted the need to update the epidemiological evidence base in order to plan appropriate approaches to the control of leishmaniasis.

Estimates of disease burden are widely used by policy-makers and funding organizations to establish priorities [7], [8], [9], [10]. These estimates are most commonly expressed as disability-adjusted life years (DALYs) lost, a measurement first promoted in the 1993 World Development Report and the focus of intense scrutiny ever since [11], [12], [13]. The accuracy of this measure depends on the reliability of the incidence, duration, severity and mortality data for a given condition, as well as the underlying assumptions used in the calculations [7], [14]. Although a new round of global disease burden estimation is currently underway, empirical data collection and field validation are neither included nor supported as part of the exercise [15].

**Table 1:** Reported and estimated incidence of visceral leishmaniasis in the American region.

	Reported VL cases/year	Years of report	Estimated annual VL incidence		
Argentina	8	2004–2008	20	to	30^1^
Bolivia	0	2008			
Brazil	3481	2003–2007	4200	to	6300^2^
Colombia	60	2004–2008	70	to	110^2^
El Salvador		no data			
Guatemala	15	2004–2008	20	to	30^2^
Honduras	6	2004–2008	7	to	10^2^
Mexico	7	2004–2008	8	to	12^2^
Nicaragua	3	2003–2007	3	to	5^2^
Paraguay	48	2004–2008	100	to	200^1^
Venezuela	40	2004–2008	50	to	70^2^
Region	3668		4500	to	6800

1 Underreporting considered moderate (2–4-fold) based on recent introduction of VL into the country.

2 Underreporting considered mild (1.2–1.8-fold) based on data from Brazil [25].

**Table 2:** Reported and estimated incidence of visceral leishmaniasis in the sub-Saharan African region.

	Reported VL cases/year	Years of report	Estimated annual VL incidence		
Central African Republic		no data			
Cameroon		no data			
Chad		no data			
Cote d’Ivoire	0	2004–2008			
DR Congo	0	2004–2008			
Gambia		no data			
Mauritania		no data			
Niger		no data			
Nigeria	1	2004–2008			
Senegal	0	2004–2008			
Zambia		no data			
Region	1				

**Table 3:** Reported and estimated incidence of visceral leishmaniasis in the East African region.

	Reported VL cases/year	Years of report	Estimated annual VL incidence		
Djibouti		no data			
Eritrea	100	2008	200	to	400^1^
Ethiopia	1860	2004–2008	3700	to	7400^1^
Kenya	145	2004–2008	610	to	1200^2^
Somalia	679	2009	1400	to	2700^1^
Sudan	3742	2005–2009	15,700	to	30,300^2^
South Sudan	1756	2004–2008	7400	to	14,200^2^
Uganda	288	2004–2008	350	to	520^3^
Region	8569		29,400	to	56,700

1 Underreporting considered moderate (2–4-fold).

2 Underreporting considered severe (4.2–8.1-fold).

3 Underreporting considered mild (1.2–1.8).

**Table 4:** Reported and estimated incidence of visceral leishmaniasis in the Mediterranean region.

	Reported VL cases/year	Years of report	Estimated annual VL incidence		
Albania	114	2004–2008	140	to	210^1^
Algeria	111	2004–2008	130	to	200^1^
Bosnia and Herzegovina	2	2002–2005	2	to	3^1^
Bulgaria	7	2004–2008	8	to	12^1^
Croatia	5	2004–2008	6	to	8^1^
Cyprus	2	2008	2	to	4^1^
Egypt	1	2008	1	to	2^1^
France	18	2004–2008	20	to	30^1^
Greece	42	2004–2008	50	to	80^1^
Israel	2	2003–2007	3	to	4^1^
Italy	134	2003–2007	160	to	240^1^
Jordan	0	2004–2008	0	to	0
Lebanon	0	2004–2008	0	to	0
Libya	3	2004–2008	5	to	10^2^
Macedonia	7	2005–2009	9	to	13^1^
Malta	2	2002–2005	3	to	4^1^
Monaco		no data			
Montenegro	3	2004–2008	4	to	5^1^
Morocco	152	2004–2008	300	to	610^2^
Palestine	5	2004–2008	10	to	20^2^
Portugal	15	2003–2007	20	to	30^1^
Slovenia		no data			
Spain	117	2004–2008	140	to	210^1^
Syria	14	2004–2008	30	to	60^2^
Tunisia	89	2004–2008	110	to	160^1^
Turkey	29	2003–2007	60	to	120^2^
Region	875		1200		2000

1 Underreporting considered mild (1.2–1.8-fold).

2 Underreporting considered moderate (2–4-fold).

**Table 5:** Reported and estimated incidence of visceral leishmaniasis in the Middle East to Central Asia.

	Reported VL cases/year	Years of report	Estimated annual VL incidence		
Afghanistan		no data			
Armenia	7	2004–2008	10	to	30^1^
Azerbaijan	28	2004–2008	60	to	110^1^
China	378	2004–2008	760	to	1500^1^
Georgia	164	2004–2008	330	to	660^1^
Iran (Islamic Republic of)	149	2004–2008	300	to	600^1^
Iraq	1711	2004–2008	3400	to	6800^1^
Kazakhstan	1	2004–2008	2	to	4^1^
Kyrgyzstan	0	2004–2008			
Oman	1	2004–2008	2	to	4^1^
Pakistan		no data			
Saudi Arabia	34	2004–2008	40	to	60^2^
Tajikistan	15	2004–2008	30	to	60^1^
Turkmenistan	0	2004–2008			
Ukraine	2	2005–2008	4	to	7^1^
Uzbekistan	7	2004–2008	10	to	30^1^
Yemen	0	2004–2008	20	to	50^1^
Region	2496		5000		10,000

1 Underreporting considered moderate (2–4-fold).

2 Underreporting considered mild (1.2–1.8).

**Table 6:** Reported and estimated incidence of visceral leishmaniasis in the Indian subcontinent and Southeast Asia.

	Reported VL cases/year	Years of report	Estimated annual VL incidence		
Bangladesh	6224	2004–2008	12,400	to	24,900^1^
Bhutan	2	2005–2009	10	to	20^2^
India	34,918	2004–2008	146,700	to	282,800^3^
Nepal	1477	2004–2008	3000	to	5900^1^
Sri Lanka		no data	6	to	10^4^
Thailand	2	2006–2010	5	to	10^5^
Region	42,623		162,100	to	313,600

1 Underreporting considered moderate (2.0–4.0-fold; based on lower proportion of cases treated in private sector compared to India).

2 Underreporting range based on 2 assessments in Bihar [27], [28].

**Table 7:** Reported and estimated incidence of cutaneous leishmaniasis in the American region.

	Reported CL cases/year	Years of report	Estimated annual CL incidence		
Argentina	261	2004–2008	730	to	1200^1^
Belize		no data			
Bolivia	2647	2004–2008	7400	to	12,200^1^
Brazil	26,008	2003–2007	72,800	to	119,600^1^
Colombia	17,420	2005–2009	48,800	to	80,100^1^
Costa Rica	1249	2002–2006	3500	to	5700^1^
Dominican Republic		no data	0	to	0
Ecuador	1724	2004–2008	4800	to	7900^1^
El Salvador		no data	0	to	0
French Guyana	233	2004–2008	650	to	1100^1^
Guatemala	684	2004–2008	1900	to	3100^1^
Guyana	16	2006–2008	50	to	70^1^
Honduras	1159	2006–2008	3200	to	5300^1^
Mexico	811	2004–2008	2300	to	3700^1^
Nicaragua	3222	2003–2007	9000	to	14,800^1^
Panama	2188	2005–2009	6100	to	10,100^1^
Paraguay	431	2004–2008	1200	to	2000^1^
Peru	6405	2004–2008	17,900	to	29,500^1^
Suriname	3	2005–2007	8	to	14^1^
Venezuela	2480	2004–2008	6900	to	11,400^1^
REGION	66,941		187,200		307,800

1 Underreporting considered mild (2.8–4.6-fold) based on data from Argentina [29].

**Table 8:** Reported and estimated incidence of cutaneous leishmaniasis in the sub-Saharan African region.

	Reported CL cases/year	Years of report	Estimated annual CL incidence		
Burkina Faso		no data			
Cameroon	55	2007–2009	280	to	550^1^
Chad		no data			
Cote d’Ivoire	1	2004–2008	5	to	10^1^
DR Congo	0	2009			
Ghana	27	2004–2008	140	to	270^1^
Guinea		no data			
Guinea-Bissau		no data			
Mali	58	2004–2008	290	to	580^1^
Mauritania		no data			
Namibia		no data			
Niger		no data			
Nigeria	5	2004–2008	30	to	50^1^
Senegal	8	2004–2008	40	to	80^1^
South Africa		no data			
REGION	155		790	to	1500^1^

1 Underreporting considered moderate (5–10-fold).

**Table 9:** Reported and estimated incidence of cutaneous leishmaniasis in the East African region.

	Reported CL cases/year	Years of report	Estimated annual CL incidence		
Djibouti		no data			
Eritrea	50	2008	250	to	500^1^
Ethiopia		no data	20,000	to	50,000^2^
Kenya		no data			
Sudan		no data	15,000	to	40,000^3^
South Sudan		no data			
REGION	50		35,300	to	90,500

1 Underreporting considered moderate (5–10-fold).

2 Based on conference report (Armauer Hansen Research Institute, Federal Ministry of Health of Ethiopia and World Health Organization. Consultative meeting for the control of cutaneous leishmaniasis in Ethiopia; June 4–5, 2011; Addis Ababa, Ethiopia).

3 Based on estimates by Dr. Nuha Hamid, national project officer, WHO-Khartoum, Sudan (see Annex).

**Table 10:** Reported and estimated incidence of cutaneous leishmaniasis in the Mediterranean.

	Reported CL cases/year	Years of report	Estimated annual CL incidence		
Albania	6	2004–2008			
Algeria	44,050	2004–2008	123,300	to	202,600^1^
Bosnia and Herzegovina	0	2008			
Bulgaria	0	2008			
Croatia	2	2004–2008	6	to	10^1^
Cyprus	1	2006–2008			
Egypt	471	2008	1300	to	2200^1^
France	2	2004–2008	6	to	10^1^
Greece	3	2004–2008	8	to	13^1^
Israel	579	2003–2007	1600	to	2700^1^
Italy	49	2003–2007	140	to	230^1^
Jordan	227	2004–2008	630	to	1000^1^
Lebanon	0	2004–2008			
Libya	3540	2004–2008	9900	to	16,300^1^
Macedonia	0	2008			
Malta	0	2008			
Monaco		no data			
Montenegro	0	2008			
Morocco	3430	2004–2008	9600	to	15,800^1^
Palestine	218	2005–2009	610	to	1000^1^
Portugal	0	2004–2008			
Slovenia		no data			
Spain	0	2004–2008			
Syria	22,882	2004–2008	64,100	to	105,300^1^
Tunisia	7631	2004–2008	21,400	to	35,100^1^
Turkey	2465	2003–2007	6900	to	11,300^1^
REGION	85,555		239,500		393,600

1 Underreporting considered mild (2.8–4.6) [29].

**Table 11:** Reported and estimated incidence of cutaneous leishmaniasis in the Middle East to Central Asia.

	Reported CL cases/year	Years of report	Estimated annual CL incidence		
Afghanistan	22,620	2003–2007	113,100	to	226,200^1^
Armenia	0	2008			
Azerbijan	17	2004–2008	50	to	80^2^
China	0	2004–2008			
Georgia	5	2004–2008			
Iran (Islamic Republic of)	24,630	2004–2008	69,000	to	113,300^2^
Iraq	1655	2004–2008	8300	to	16,500^3^
Kazakhstan	15	2004–2008	40	to	70^2^
Kyrgyzstan	0	2004–2008			
Mongolia		no data			
Oman	5	2004–2008	15	to	20^2^
Pakistan	7752	2004–2008	21,700	to	35,700^2^
Saudi Arabia	3445	2004–2008	9600	to	15,800^2^
Tajikistan	25	2007–2008	125	to	250^3^
Turkmenistan	99	2004–2008	490	to	990^3^
Ukraine	2	2004–2008	10	to	20^3^
Uzbekistan	142	2004–2008	710	to	1400^3^
Yemen	603	2005–2009	3000	to	6000^3^
REGION	61,013		226,200		416,400

1 Underreporting considered moderate (5–10-fold) based on estimates of incidence from population-based surveys [30].

2 Underreporting considered mild (2.8–4.6) [29].

3 Underreporting considered moderate (5–10-fold).

**Table 12:** Reported and estimated incidence of cutaneous leishmaniasis in the Indian subcontinent.

	Reported CL cases/year	Years of report	Estimated annual CL incidence		
India	156	2005–2009	1000	to	2000^1^
Sri Lanka	322	2004–2008	900	to	1500^2^
REGION	478		1900	to	3500

1 Based on estimates by Dr RA Bumb, Department of Skin, STD and Leprosy, SP Medical College, Bikaner, Rajasthan, India.

2 Underreporting considered mild (2.8–4.6) [29].

**Table 13:** Global reported and estimated incidence of visceral leishmaniasis.

	Reported VL cases/year	Countries with 5 years of data	Estimated annual VL incidence		
Americas	3662	8/11 (73%)	4500	to	6800
Sub-Saharan Africa	1	3/11 (27%)			
East Africa	8569	5/8 (63%)	29,400	to	56,700
Mediterranean	875	21/26 (81%)	1200	to	2000
Middle East to Central Asia	2496	14/17 (82%)	5000	to	10,000
South Asia	42,623	3/6 (50%)*	162,100	to	313,600
Global total	58,227	54/79 (68%)	202,200	to	389,100

* 3/3 (100%) of high burden countries (India, Bangladesh, Nepal) reported 5 years of data. Reports incomplete for Sri Lanka, Bhutan and Thailand.

**Table 14:** Global reported and estimated incidence of cutaneous leishmaniasis.

	Reported CLcases/year	Countries with 5 yearsof data	Estimated annual CL incidence		
Americas	66,941	14/20 (70%)	187,200	to	307,800
Sub-Saharan Africa	155	5/15 (33%)	770	to	1500
East Africa	50	0/6 (0%)	35,300	to	90,500
Mediterranean	85,555	17/26 (65%)	239,500	to	393,600
Middle East to Central Asia	61,013	16/18 (89%)	226,200	to	416,400
South Asia	322	2/2 (100%)	1900	to	3500
Global total	214,036	53/87 (61%)	690,900	to	1,213,300

The evidence base for the neglected tropical diseases (NTDs) is acknowledged to be particularly problematic [9], [16]. Leishmaniasis, like many other NTDs, occurs in a focal distribution and in remote locations, making extrapolation from official data sources difficult [4]. Visceral leishmaniasis (VL) results in death if not treated, the majority of leishmaniasis deaths go unrecognized, and even with treatment access, VL may result in case-fatality rates of 10–20% [17], [18], [19], [20], [21], [22]. Reported leishmaniasis case figures are widely acknowledged to represent gross underestimates of the true burden, but studies that measure the degree of underreporting are rare [23]. As part of the WHO effort to update the leishmaniasis evidence base, a series of regional meetings were held. National program managers and expert professionals were asked to provide detailed information on epidemiology, ecology, geographical distribution and trends, drug access and management of leishmaniasis for their respective countries. These data, accompanied by literature reviews, are compiled in extensive profiles of each endemic country or territory in the Annex of this publication (see ‘Leishmaniasis Country Profiles Text S1, S2, S3, S4, S5, S6, S7, S8, S9, S10, S11, S12, S13, S14, S15, S16, S17, S18, S19, S20, S21, S22, S23, S24, S25, S26, S27, S28, S29, S30, S31, S32, S33, S34, S35, S36, S37, S38, S39, S40, S41, S42, S43, S44, S45, S46, S47, S48, S49, S50, S51, S52, S53, S54, S55, S56, S57, S58, S59, S60, S61, S62, S63, S64, S65, S66, S67, S68, S69, S70, S71, S72, S73, S74, S75, S76, S77, S78, S79, S80, S81, S82, S83, S84, S85, S86, S87, S88, S89, S90, S91, S92, S93, S94, S95, S96, S97, S98, S99, S100, S101’). This paper focuses on an analysis of the findings, and estimates of leishmaniasis incidence derived from the epidemiological data.

## Methods

From 2007 to 2010, WHO organized a series of regional meetings (EMRO countries, Geneva 2007; PAHO countries, Medellin 2008; EURO countries, Istanbul 2009; AFRO countries, Addis Ababa 2010; SEARO countries, Paro 2011). In preparation for each meeting, country representatives were asked to provide yearly reported VL and cutaneous leishmaniasis (CL) incidence data for at least the last 5 years prior to the meeting. In addition, an electronic epidemiological questionnaire was sent to the national control program managers and/or to reputable national scientists to fill information gaps. Data collected included administrative divisions affected, whether VL and CL case notification is mandatory, characteristics of known reservoirs and vector control programs, estimated and reported case numbers, and outbreaks in the previous 5 years.

A comprehensive literature search was also conducted, and the resulting information was used as an independent validation of these data. We reviewed the literature based on MEDLINE searches using the terms *leishmaniasis* and *epidemiology* with the name of each endemic country or territory. For the initial search, we included all articles listed in MEDLINE in English, French, Spanish or Russian up to October 2010, when the search was conducted. We selected articles that explicitly addressed incidence, geographic distribution, surveillance and/or trends over time, and preferentially chose articles published since 2000 if available. For countries with sparse data on leishmaniasis, we broadened the review to include all articles that shed light on the occurrence of the disease within that country. We reviewed titles for all references, abstracts when available for those whose titles were not sufficient to lead us to exclude the paper, and the full article when the abstract indicated possible relevance. The search for country-specific literature yielded 3242 potentially relevant articles, of which 340 were retained based on our selection criteria. Five recent review articles were also included. Twenty-six additional unpublished reports were provided by national or international experts. The literature was reviewed by at least 2 authors and regular meetings were held among the authors to discuss the findings in depth.

A MEDLINE search was also performed using the terms *leishmaniasis* and *underreporting* to identify articles that would aid in making incidence estimates. This search yielded 8 articles of which 5 presented data on the magnitude of leishmaniasis underreporting. One additional article was identified from author literature collections, yielding 3 articles with empirical data regarding VL and 3 for CL underreporting [24], [25], [26], [27], [28], [29]. These articles were used to establish probable degrees of underreporting for the countries in which their analyses were performed, and were also used for estimates in countries judged similar in their degree of underreporting. National and international experts provided their judgements of the magnitude of underreporting. In addition, for countries where reporting is sparse, but surveys have been performed, the published data were used as a basis to select the appropriate degree of underreporting [30]. Wherever possible, estimated plausible VL and CL incidence ranges were assigned by country and/or region based on reported incidence and multiplication by the probable underreporting factors. Estimates less than 20 were retained as the precise product of the reported case number times the underreporting factor, those between 20 and 1000 were rounded to the nearest 10 and those over 1000 were rounded to the nearest 100. Where reporting was absent but incidence was known to be substantial, estimates were assigned based on the judgment of national and international experts. The regional estimates represent the sum of the country estimates followed by the same rounding process. Similarly, the global estimates represent the sum of the regional estimates followed by rounding as described above. In order to facilitate expert judgment regarding the probable accuracy of the figures presented here, we defined geographical regions consistent with the major ecological foci of leishmaniasis transmission, rather than official WHO regions [31], [32], [33].

A second questionnaire addressed access to antileishmanial medicines, and included specific questions: whether the public sector provides health care free of charge; the existence of a national program for control of leishmaniasis; inclusion of antileishmanial medicines in the National Essential Drug List; the number of different medicines purchased for the public sector or donations received in the last two years; sale of antileishmanial drugs in the private sector and price per tablet or vial; percentage of people using the for-profit private sector versus public sector for leishmaniasis treatment; health care level providing treatment in the public sector; presence of NGOs or other non-profit agencies providing leishmaniasis treatment; and barriers to access for treatment of leishmaniasis. Basic social and health data from each country were obtained from the websites of the relevant international agencies [34], [35], [36], [37], [38].

The epidemiological data were used to produce maps with 2008 as the reference year using ArcGIS 9.3– Desktop (Esri, Redlands, CA) and following WHO guidelines for GIS usage. The numbers of confirmed cases by clinical form (VL, CL, mucocutaneous leishmaniasis) were mapped by official first level administrative division. These data were used to calculate annual incidence rates. A single standard range of values was used for each clinical form to facilitate visual comparison between countries. Draft maps were shared with data providers and other leishmaniasis experts for validation. The following maps were developed for each country: situational map with neighbouring countries and world globe, maps of cases by clinical form, and maps of incidence per 10,000 inhabitants. All maps follow a consistent set of characteristics: five categories of colours in a yellow-to-red scale were chosen for the maps of cases, and six categories of colours in blue tones scale were chosen for the maps of incidence. The sparse information in a few countries required the use of *ad hoc* scales. Only WHO GIS shapefile databases were used; the maps follow the administrative limits and frontiers recognized by United Nations conventions.

The parasitological information has been reproduced from the WHO Technical Report Series 949 (http://whqlibdoc.who.int/trs/WHO_TRS_949_eng.pdf) published in 2010.

Basic social and health data, results of literature reviews, data on the magnitude of underreporting, maps, data regarding epidemiology, case load, access to treatment and access to drugs, and parasitological information are presented in a series of extensive Profiles of each endemic individual country and territory and are presented in the Annex of this publication (see ‘Leishmaniasis Country Profiles Text S1, S2, S3, S4, S5, S6, S7, S8, S9, S10, S11, S12, S13, S14, S15, S16, S17, S18, S19, S20, S21, S22, S23, S24, S25, S26, S27, S28, S29, S30, S31, S32, S33, S34, S35, S36, S37, S38, S39, S40, S41, S42, S43, S44, S45, S46, S47, S48, S49, S50, S51, S52, S53, S54, S55, S56, S57, S58, S59, S60, S61, S62, S63, S64, S65, S66, S67, S68, S69, S70, S71, S72, S73, S74, S75, S76, S77, S78, S79, S80, S81, S82, S83, S84, S85, S86, S87, S88, S89, S90, S91, S92, S93, S94, S95, S96, S97, S98, S99, S100, S101’).

## Results

A total of 98 countries and 3 territories on 5 continents reported endemic leishmaniasis transmission (Tables 1, 2, 3, 4, 5, 6 and 7, 8, 9, 10, 11, 12). In total, official case counts totalled more than 58,000 VL cases and 220,000 CL cases per year (Tables 13 and 14). However, only about two-thirds of countries had reported incidence data for a five-year period; data were sparsest for the foci in Africa. A number of countries are listed here as endemic despite the lack of reported human cases, usually reflecting an absence of surveillance or other investigations. [39] For example, although Mongolia has not reported human CL cases, *L. major* genetically identical to that found in countries with proven endemic transmission has been isolated on multiple occasions from gerbils. [40] Only countries with circulating species known to be pathogenic to humans are included as endemic. For this reason, Australia is not considered endemic despite reports of CL among red kangaroos caused by a newly described leishmanial species. [41] Human infections due to lower trypanosomatids are also excluded. [42].

There are few published empirical assessments of underreporting in official surveillance data. Two studies from Bihar, India, compared VL case numbers ascertained through active house-to-house surveys to those reported in the official surveillance system; official figures were shown to be 4.2-fold and 8.1-fold lower than the incidence found by active case detection in the two studies, respectively. [27], [28] A study in Brazil used the capture-recapture method to estimate underreporting of VL, based on data from 3 different sources; the degree of underreporting was found to be 1.3- to 1.7-fold. [25] Data from one province in Argentina estimated the degree of CL underreporting to be 2.8 to 4.6-fold; however, studies from Guatemala and Jordan indicate that CL incidence may be underestimated by 40- to 47-fold in national surveillance data. [24], [26], [29] Based on these publications, country-level VL underreporting magnitude was categorized as follows: mild (1.2- to 1.8-fold based on data from Brazil [25]); severe (4.0- to 8.0-fold based on data from India [27], [28]); and an intermediate category of moderate (2.0 to 4.0-fold) underreporting. Despite the high published range of CL underreporting [24], [26], we chose conservative multipliers: mild (2.8 to 4.6-fold based on data from Argentina [29]) and moderate (5.0- to 10.0-fold). No estimates could be made for most countries in sub-Saharan Africa, where almost no data were available.

Based on these estimates, approximately 0.2 to 0.4 million VL cases and 0.7 to 1.2 million CL cases occur each year. More than 90% of global VL cases occur in just six countries: India, Bangladesh, Sudan, South Sudan, Brazil and Ethiopia (Table 13). Cutaneous leishmaniasis is more widely distributed, with about one-third of cases occurring in each of three regions, the Americas, the Mediterranean basin, and western Asia from the Middle East to Central Asia (Table 14). The ten countries with the highest estimated case counts, Afghanistan, Algeria, Colombia, Brazil, Iran, Syria, Ethiopia, North Sudan, Costa Rica and Peru, together account for 70 to 75% of global estimated CL incidence.

Mortality data are extremely sparse and generally represent hospital-based deaths only. The reported case-fatality rate for VL in Brazil in 2006 was 7.2%. In the Indian subcontinent, the focus responsible for the largest proportion of global VL cases, reported case-fatality rates ranged from 1.5% (93 deaths/6224 VL cases from 2004–2008) in Bangladesh to 2.4% (853/34,918) in India and 6.2% (91/1477) in Nepal. However, community-based studies that included active searches for deaths due to kala-azar estimate case-fatality rates of more than 10%, while data from a village-based study in India suggest that as many as 20% of VL patients, disproportionately poor and female, died before their disease was recognized. [43], [44], [45] In South Sudan, one community-based longitudinal study demonstrated a case-fatality rate of 20% in a settled village in peacetime; in areas of conflict, famine or population displacement mortality rates are much higher. [22], [46] A recent study from South Sudan estimated that 91% of all kala-azar deaths went unrecognized. [47] Using an overall case-fatality rate of 10% and assuming that virtually all deaths are from VL, we reach a tentative estimate of 20,000 to 40,000 leishmaniasis deaths per year, in line with previous WHO estimates. [10]

## Discussion

The data presented here and in the accompanying Annex (see ‘Leishmaniasis Country Profiles Text S1–S101’) represent the first update of the empirical database for leishmaniasis since 1991. [48], [49] We are acutely cognizant of the uncertainties inherent in the data, and for that reason, have presented rough ranges rather than single estimates for each outcome. We deliberately used conservative assumptions for the underreporting rates and resultant multipliers; true leishmaniasis incidence rates may be substantially higher. Due to the lack of data, we made no estimates for post-kala-azar dermal leishmaniasis, mucocutaneous leishmaniasis, and other less frequent forms of leishmaniasis. Our mortality estimate contains even more uncertainty than the incidence estimate, because studies affirm that a large proportion of kala-azar deaths occur outside of health facilities and the cause likely never recognized, precluding the possibility of accurate passive reporting. [43], [45], [47].

The limitations of these data are obvious: surveillance and vital records reporting in the countries most affected by leishmaniasis are incomplete, and we have very sparse data on which to base correction factors for underreporting. The figures in this report should not be considered precise and should be interpreted with caution. Nevertheless, these data include a more comprehensive review of leishmaniasis incidence than any previous publication, and represent a major improvement in the evidence base for one of the most neglected diseases. [50] Better surveillance systems are urgently needed, in particular in disease foci targeted for more intensive control or elimination. [4], [51] Many key measures of progress, such as validation of trends seen in surveillance data and accurate case-fatality rates, can only be obtained through the active collection of community-based data. [4], [52] We hope the data presented here will allow a more nuanced interpretation of published disease burden estimates, and the uncertainties in these data will spur activities to improve the evidence base for leishmaniasis and other neglected diseases.

## Supporting Information

Text S1 **Leishmaniasis Country Profiles, Afghanistan.**
(DOCX)

Text S2 **Leishmaniasis Country Profiles, Albania.**
(DOCX)

Text S3 **Leishmaniasis Country Profiles, Algeria.**
(DOCX)

Text S4 **Leishmaniasis Country Profiles, Argentina.**
(DOCX)

Text S5 **Leishmaniasis Country Profiles, Armenia.**
(DOCX)

Text S6 **Leishmaniasis Country Profiles, Azerbijan.**
(DOCX)

Text S7 **Leishmaniasis Country Profiles, Bangladesh.**
(DOCX)

Text S8 **Leishmaniasis Country Profiles, Belize.**
(DOCX)

Text S9 **Leishmaniasis Country Profiles, Bhutan.**
(DOCX)

Text S10 **Leishmaniasis Country Profiles, Bolivia.**
(DOCX)

Text S11 **Leishmaniasis Country Profiles, Bosnia.**
(DOCX)

Text S12 **Leishmaniasis Country Profiles, Brazil.**
(DOCX)

Text S13 **Leishmaniasis Country Profiles, Bulgaria.**
(DOCX)

Text S14 **Leishmaniasis Country Profiles, Burkina Faso.**
(DOCX)

Text S15 **Leishmaniasis Country Profiles, Cameroon.**
(DOCX)

Text S16 **Leishmaniasis Country Profiles, Central African Republic.**
(DOCX)

Text S17 **Leishmaniasis Country Profiles, Chad.**
(DOCX)

Text S18 **Leishmaniasis Country Profiles, China.**
(DOCX)

Text S19 **Leishmaniasis Country Profiles, Colombia.**
(DOCX)

Text S20 **Leishmaniasis Country Profiles, Costa Rica.**
(DOCX)

Text S21 **Leishmaniasis Country Profiles, Cote d’Ivoire.**
(DOCX)

Text S22 **Leishmaniasis Country Profiles, Croatia.**
(DOCX)

Text S23 **Leishmaniasis Country Profiles, Cyprus.**
(DOCX)

Text S24 **Leishmaniasis Country Profiles, Democratic Republic of the Congo.**
(DOCX)

Text S25 **Leishmaniasis Country Profiles, Djibouti.**
(DOCX)

Text S26 **Leishmaniasis Country Profiles, Dominican Republic.**
(DOCX)

Text S27 **Leishmaniasis Country Profiles, Ecuador.**
(DOCX)

Text S28 **Leishmaniasis Country Profiles, Egypt.**
(DOCX)

Text S29 **Leishmaniasis Country Profiles El Salvador.**
(DOCX)

Text S30 **Leishmaniasis Country Profiles, Eritrea.**
(DOCX)

Text S31 **Leishmaniasis Country Profiles, Ethiopia.**
(DOCX)

Text S32 **Leishmaniasis Country Profiles, France.**
(DOCX)

Text S33 **Leishmaniasis Country Profiles, French Guyana.**
(DOCX)

Text S34 **Leishmaniasis Country Profiles, Gambia.**
(DOCX)

Text S35 **Leishmaniasis Country Profiles, Georgia.**
(DOCX)

Text S36 **Leishmaniasis Country Profiles, Ghana.**
(DOCX)

Text S37 **Leishmaniasis Country Profiles, Greece.**
(DOCX)

Text S38 **Leishmaniasis Country Profiles, Guatemala.**
(DOCX)

Text S39 **Leishmaniasis Country Profiles, Guinea Bissau.**
(DOCX)

Text S40 **Leishmaniasis Country Profiles, Guinea.**
(DOCX)

Text S41 **Leishmaniasis Country Profiles, Guyana.**
(DOCX)

Text S42 **Leishmaniasis Country Profiles, Honduras.**
(DOCX)

Text S43 **Leishmaniasis Country Profiles, India.**
(DOCX)

Text S44 **Leishmaniasis Country Profiles, Iran.**
(DOCX)

Text S45 **Leishmaniasis Country Profiles, Iraq.**
(DOCX)

Text S46 **Leishmaniasis Country Profiles, Israel.**
(DOCX)

Text S47 **Leishmaniasis Country Profiles, Italy.**
(DOCX)

Text S48 **Leishmaniasis Country Profiles, Jordan.**
(DOCX)

Text S49 **Leishmaniasis Country Profiles, Kazakhstan.**
(DOCX)

Text S50 **Leishmaniasis Country Profiles, Kenya.**
(DOCX)

Text S51 **Leishmaniasis Country Profiles, Kuwait.**
(DOCX)

Text S52 **Leishmaniasis Country Profiles, Kyrgyzstan.**
(DOCX)

Text S53 **Leishmaniasis Country Profiles, Lebanon.**
(DOCX)

Text S54 **Leishmaniasis Country Profiles, Libya.**
(DOCX)

Text S55 **Leishmaniasis Country Profiles, Malawi.**
(DOCX)

Text S56 **Leishmaniasis Country Profiles, Mali.**
(DOCX)

Text S57 **Leishmaniasis Country Profiles, Malta.**
(DOCX)

Text S58 **Leishmaniasis Country Profiles, Mauritania.**
(DOCX)

Text S59 **Leishmaniasis Country Profiles, Mexico.**
(DOCX)

Text S60 **Leishmaniasis Country Profiles, Monaco.**
(DOCX)

Text S61 **Leishmaniasis Country Profiles, Mongolia.**
(DOCX)

Text S62 **Leishmaniasis Country Profiles, Montenegro.**
(DOCX)

Text S63 **Leishmaniasis Country Profiles, Morocco.**
(DOCX)

Text S64 **Leishmaniasis Country Profiles, Namibia.**
(DOCX)

Text S65 **Leishmaniasis Country Profiles, Nepal.**
(DOCX)

Text S66 **Leishmaniasis Country Profiles, Nicaragua.**
(DOCX)

Text S67 **Leishmaniasis Country Profiles, Niger.**
(DOCX)

Text S68 **Leishmaniasis Country Profiles, Nigeria.**
(DOCX)

Text S69 **Leishmaniasis Country Profiles, Oman.**
(DOCX)

Text S70 **Leishmaniasis Country Profiles, Pakistan.**
(DOCX)

Text S71 **Leishmaniasis Country Profiles, Panama.**
(DOCX)

Text S72 **Leishmaniasis Country Profiles, Paraguay.**
(DOCX)

Text S73 **Leishmaniasis Country Profiles, Peru.**
(DOCX)

Text S74 **Leishmaniasis Country Profiles, Portugal.**
(DOCX)

Text S75 **Leishmaniasis Country Profiles, Romania.**
(DOCX)

Text S76 **Leishmaniasis Country Profiles, Saudi Arabia.**
(DOCX)

Text S77 **Leishmaniasis Country Profiles, Senegal.**
(DOCX)

Text S78 **Leishmaniasis Country Profiles, Slovenia.**
(DOCX)

Text S79 **Leishmaniasis Country Profiles, Somalia.**
(DOCX)

Text S80 **Leishmaniasis Country Profiles, South Africa.**
(DOCX)

Text S81 **Leishmaniasis Country Profiles, South Sudan.**
(DOCX)

Text S82 **Leishmaniasis Country Profiles, Spain.**
(DOCX)

Text S83 **Leishmaniasis Country Profiles, Sri Lanka.**
(DOCX)

Text S84 **Leishmaniasis Country Profiles, Sudan.**
(DOCX)

Text S85 **Leishmaniasis Country Profiles, Surinam.**
(DOCX)

Text S86 **Leishmaniasis Country Profiles, Syrian Arab Republic.**
(DOCX)

Text S87 **Leishmaniasis Country Profiles, Taiwan.**
(DOCX)

Text S88 **Leishmaniasis Country Profiles, Tajikistan.**
(DOCX)

Text S89 **Leishmaniasis Country Profiles, Thailand.**
(DOCX)

Text S90 **Leishmaniasis Country Profiles, The Former Yugoslav Republic of Macedonia.**
(DOCX)

Text S91 **Leishmaniasis Country Profiles, Tunisia.**
(DOCX)

Text S92 **Leishmaniasis Country Profiles, Turkey.**
(DOCX)

Text S93 **Leishmaniasis Country Profiles, Turkmenistan.**
(DOCX)

Text S94 **Leishmaniasis Country Profiles, Uganda.**
(DOCX)

Text S95 **Leishmaniasis Country Profiles, Ukraine.**
(DOCX)

Text S96 **Leishmaniasis Country Profiles, United States of America.**
(DOCX)

Text S97 **Leishmaniasis Country Profiles, Uzbekistan.**
(DOCX)

Text S98 **Leishmaniasis Country Profiles, Venezuela.**
(DOCX)

Text S99 **Leishmaniasis Country Profiles, West Bank and Gaza Strip.**
(DOCX)

Text S100 **Leishmaniasis Country Profiles, Yemen.**
(DOCX)

Text S101 **Leishmaniasis Country Profiles, Zambia.**
(DOCX)
