# Supplementary material for: Leishmaniasis Worldwide and Global Estimates of Its Incidence
Source: PLoS One. 2012 May 31;7(5):e35671. doi: 10.1371/journal.pone.0035671 (PMC3365071; doi:10.1371/journal.pone.0035671)
Supplement: Text S1 — Leishmaniasis Country Profiles, Afghanistan. (DOCX) [file pone.0035671.s001.docx]

**AFGHANISTAN**

**
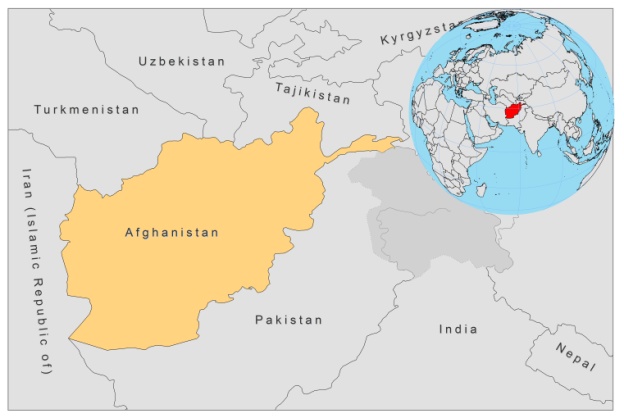
**

**BASIC COUNTRY DATA**

Total Population: 34,385,068

Population 0-14 years: 46%

Rural population: 75%

Population living under USD 1.25 a day: no data

Population living under the national poverty line: 36%

Income status: Low income economy

Ranking: Low human development (ranking 172 )

Per capita total expenditure on health at average exchange rate (US dollar): 51

Life expectancy at birth (years): 48

Healthy life expectancy at birth (years): 36

**BACKGROUND INFORMATION**

CL is well known in Afghanistan under the name ‘saldana’ ('that what lasts one year'). Leishmaniasis in Afghanistan was almost eradicated in the 1960s and 70s, when an extensive DDT spraying campaign majorly reduced the sandfly population. However, ACL by *L.tropica* has now returned as a major public health problem. Through mass migration and a lack of treatment and control measures, spreading to new areas has occurred. Now, the disease is endemic in independent foci in the major cities of Kabul, Herat and Kandahar, as well as in dependent foci in Charikar, Panjsheer valley, Gorband valley and villages around Kandahar. The case load is especially high in Kabul. A survey, conducted in 2002, showed that 22% of people in Kabul had scars and in 2004, there were an estimated 67,500 active cases in Kabul alone [1]. In the past decade, Kabul had the highest incidence of CL in the world and the number of cases is still increasing. In 2007 and 2008, the number of newly reported CL cases in Kabul was around 30,000 and the incidence was over 200,000 cases [2]. In 2010, an incidence of 155/100,000 population was reported. Poor housing conditions, living in close proximity to animals, and displacement and migration of people, particularly to cities, are thought to prolong the epidemic.

A survey conducted in Kabul in 2003 revealed that females are more affected than males and that active lesions are more prevalent among those aged 6-20 years, living in dwellings with animals or in mud houses [3]. Because of sandfly vector exposure, most leishmaniasis lesions occur on the face; the marring and disfigurement following the disease causes severe stigma for women [4]. In 2009, gender distribution became equal and 24% of cases were under 5 years, 39% were between 5 and 14 years and 37% were over 14 years old.

ZCL caused by *L. major* is endemic in Afghanistan, but knowledge of its regional and seasonal distribution, as well as its public health impact, is limited. It occurs mainly in the northern plains of the Amu Darya River.

CL is underreported. Surveys consistently show higher case numbers than the officially reported ones. Reporting systems are weak and many patients resort to the informal private sector because of the lack of drugs in government clinics.

ZVL has a widespread distribution, but the number of reported cases is low. Neither vectors nor reservoirs have been identified and it is unknown whether the transmission cycle is anthroponotic or zoonotic. The first case of VL was reported in 1980. VL is very likely to be underreported, due to a lack of awareness of the disease among the public and among health personnel. The risk of future local outbreaks cannot be excluded.

No cases of HIV-*Leishmania* co-infection have been reported.

**PARASITOLOGICAL INFORMATION**

| ***Leishmania* species** | **Clinical form** | **Vector species** | **Reservoirs** |
| --- | --- | --- | --- |
| *L. tropica* | ACL | *P. sergenti* | Human |
| *L. major* | ZCL | *P. papatasi,*  *P. caucasius* | *Rhombomys opimus,*  *Meriones spp.* |
| *L. infantum* | ZVL | *unknown* | *Canis familiaris* |

**MAPS AND TRENDS**

**Cutaneous leishmaniasis**


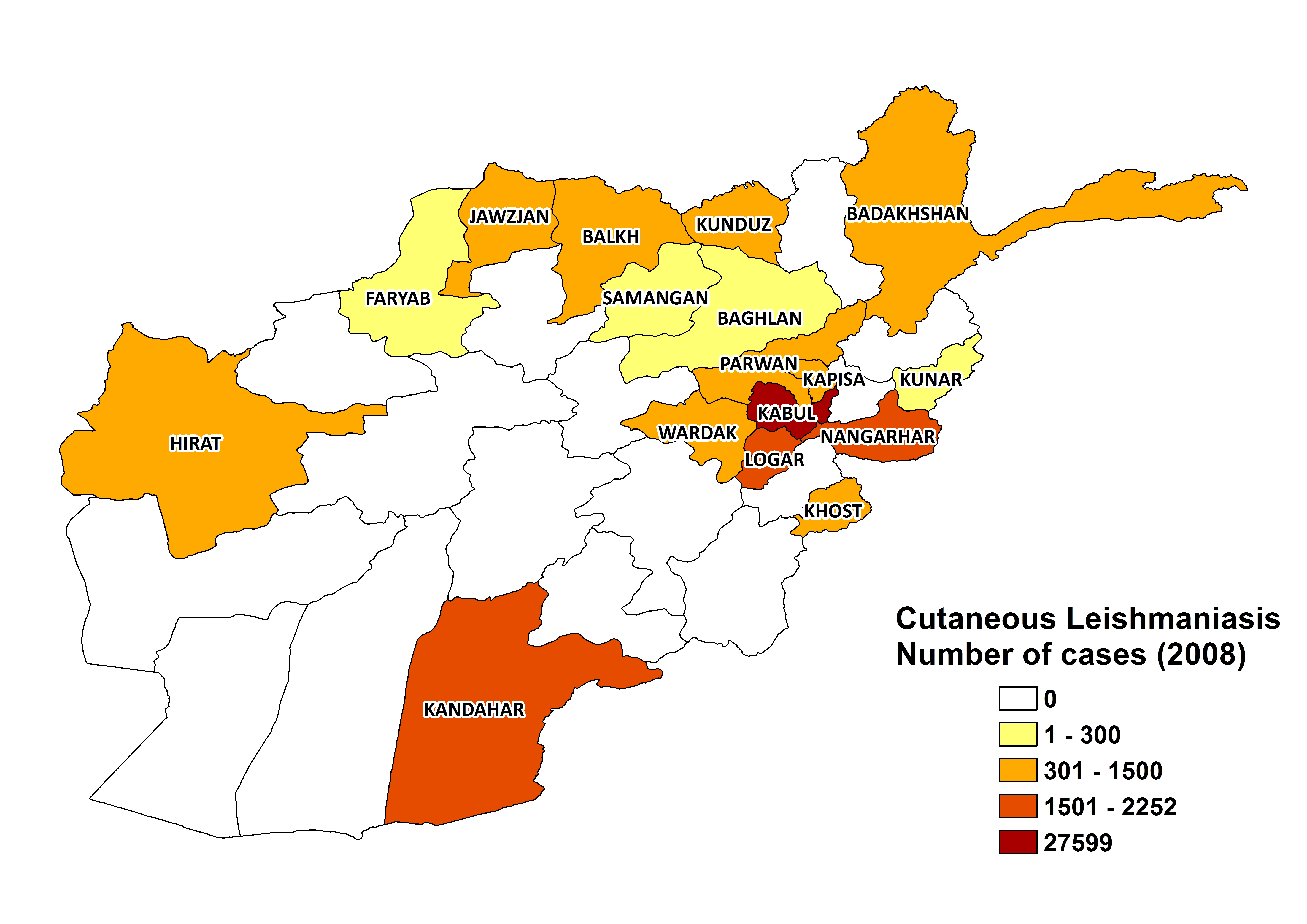

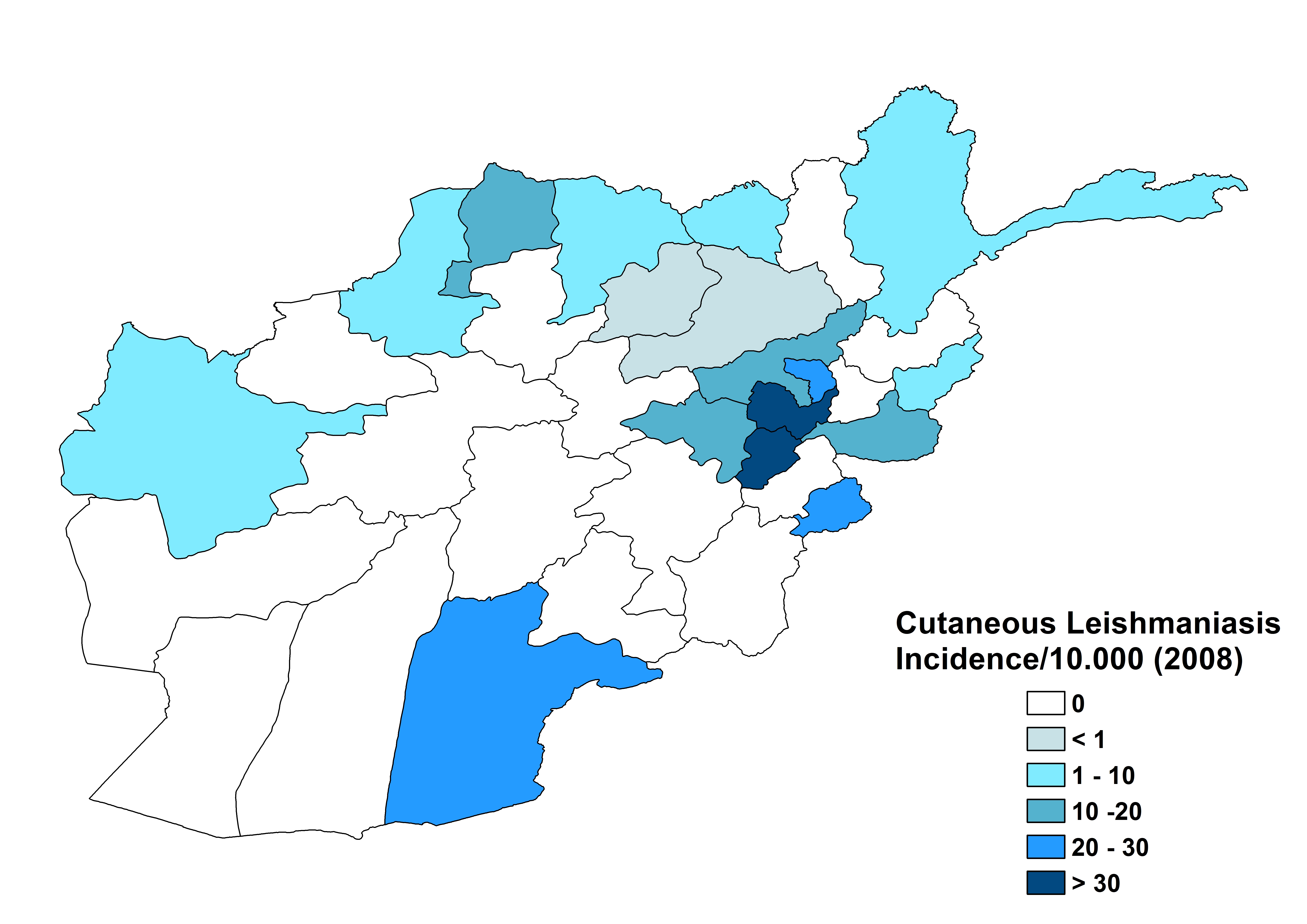


**Visceral leishmaniasis**

**
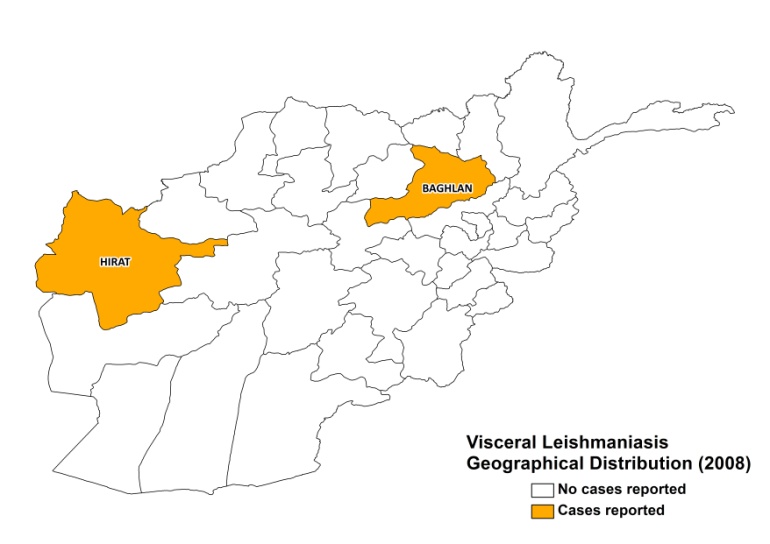
**

**Cutaneous leishmaniasis trend**

**Visceral leishmaniasis**

| **Year** | **Cases** |
| --- | --- |
| **2010** | **15** |

**CONTROL**

A national leishmaniasis control program has been in place since 1947, but decades of war and their destructive effect on the health system have now rendered it largely ineffective. Nevertheless, over the past three years the 'National Malaria and Leishmaniasis Program' (NMLP) has been revitalized and is meeting periodically with the main stakeholders. There is no vector or reservoir control in place. However, the free distribution of long lasting insecticide nets (LLINs) for malaria control may have had positive effects on the prevalence of leishmaniasis. A national protocol was implemented in 2011.

**DIAGNOSIS, TREATMENT**

**Diagnosis**

CL: clinical, confirmation by microscopic examination of skin lesion sample.

VL: clinical, confirmation by microscopic examination of bone marrow aspirate.

**Treatment**

CL: Antimonials, intralesional or systemic (20 mg Sb^v^/kg/day for 3 weeks). Patients with less than 6 lesions smaller than 3 cm are not treated unless the lesions are facial. Information about treatment outcomes is not available. In some private clinics and NGO facilities, CL is treated with thermotherapy.

**ACCESS TO CARE**

Medical care is not provided for free in Afghanistan. A nominal user fee is charged, and this includes care for leishmaniasis. Drugs for leishmaniasis are distributed in primary and secondary levels of healthcare. Care for leishmaniasis is also provided by NGOs on a relatively large scale (10,311 CL patients were treated by Today Japan in 2008). Approximately 30-50% of VL patients and 5-15% of CL patients are estimated to use the private sector. There are serious problems with access to care. For treatment, most patients depend on public health facilities, which often have no drugs to treat leishmaniasis and are currently fully dependant on WHO donations of antimonials. Patients have to purchase the drugs themselves if these run out. WHO donated 30,000 vials of meglumine antimoniate (Glucantime, Sanofi) in 2008. This amount is sufficient for the topical treatment of CL of approximately 1,800 patients. That same year, 41,137 patients were reported in the public sector, most of whom had to purchase antimonials themselves. In 2009, WHO donated 5,400 vials of sodium stibogluconate (Pentostam, GSK) and 1,390 vials of Glucantime, sufficient for topical treatment of about 8,000 patients.

Apart from a lack of drugs, public health facilities also suffer from a serious lack of health personnel, both in numbers and in quality of training in leishmaniasis diagnosis and treatment.

Patients living in Kabul generally have access to public health facilities. However, considerable numbers of patients living in remote areas outside of Kabul, with no health facilities and no or unaffordable transport, suffer major economic loss when they spend time away from home in order to receive treatment. Gender inequality in seeking treatment has been observed.

**ACCESS TO DRUGS**

Sodium stibogluconate and meglumine antimoniate are included in the National Essential Drug List for the Essential Package of Hospital Services (EPHS) only, which has been a main barrier to access for patients at the PHC level. No drugs are registered for leishmaniasis. In private pharmacies, antimonials are available at high prices; Glucantime (Sanofi) costs 7-8 USD per ampoule, leading to a cost of over 100 USD for topical treatment of CL. Also available in pharmacies are Meglutin (Star Laboratories, Pakistan) and Miglom (made in Syria), generic forms of meglumine antimoniate, not approved by WHO, that cost 3 to 5 USD per ampoule.

**SOURCES OF INFORMATION**

- Dr Najibullah Safi and Dr. Ahmad Walid Sediqi, National Malaria & Leishmaniasis Control Program. Ministry of Public Health, Afghanistan.
- Drs Elena Vuolo and Waqar Butt, Malaria & Leishmaniasis Program WHO/EMRO, Afghanistan.

1. Reithinger R, Mohsen M, Aadil K, Sidiqi M, Erasmus P et al (2003). Anthroponotic cutaneous leishmaniasis, Kabul, Afghanistan. Emerg Infect Dis 9(6):727-9.

2. Reyburn H, Rowland M, Mohsen M, Khan B, Davies C (2003). The prolonged epidemic of anthroponotic cutaneous leishmaniasis in Kabul, Afghanistan: 'bringing down the neighbourhood'. Trans R Soc Trop Med Hyg 97(2):170-6.

3. Reithinger R, Mohsen M, Leslie T (2010). Risk factors for anthroponotic cutaneous Leishmaniasis at the household level in Kabul, Afghanistan. PLoS Negl Trop Dis 4(3):e639.

4. Kassi M, Afghan AK, Rehman R, Kasi PM (2008). Marring leishmaniasis: the stigmatization and the impact of cutaneous leishmaniasis in Pakistan and Afghanistan. PLoS Negl Trop Dis 2(10):e259.
