# Supplementary material for: Leishmaniasis Worldwide and Global Estimates of Its Incidence
Source: PLoS One. 2012 May 31;7(5):e35671. doi: 10.1371/journal.pone.0035671 (PMC3365071; doi:10.1371/journal.pone.0035671)
Supplement: Text S2 — Leishmaniasis Country Profiles, Albania. (DOCX) [file pone.0035671.s002.docx]

**ALBANIA**

**
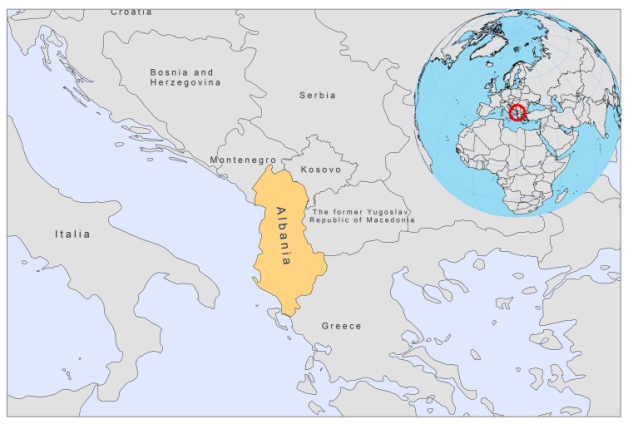
**

**BASIC COUNTRY DATA**

Total Population: 3,204,284

Population 0-14 years: 23%

Rural population: 52%

Population living under USD 1.25 a day: 0.6%

Population living under the national poverty line: 12.4%

Income status: Upper middle income economy

Ranking: High human development (ranking 70)

Per capita total expenditure on health at average exchange rate (US dollar): 265

Life expectancy at birth (years): 77

Healthy life expectancy at birth (years): 61

**BACKGROUND INFORMATION**

VL is a frequently occurring disease in Albania. It is a typical childhood disease, but VL is also recorded in adults [1,2]. The first registered case of VL caused by *L infantum* occurred in 1938. Until 1962, an average of 8 cases per year was reported [2]. After this year, registered case numbers increased to 50-100 yearly. From 1984 to 2001, about 85 cases per year were registered in 35 out of the 36 districts. From January 1997 to December 2001, 867 parasitologically confirmed VL cases were recorded in 35 of 36 Albanian districts, with a cumulative morbidity of 2.8/10,000 population [2]. In 2001, the incidence rose to 0.7/10,000 population, which was 20-40 times higher than in the other European endemic countries [2]. The incidence decreased to 0.4/10,000 in 2004 and to 0.3/10,000 in 2007. The cumulative mortality of VL from 1997-2001 was 0.5%, indicating that diagnosis and treatment were of good standard [2].

The majority of cases (68%) occur in children under the age of 5 and 80% of cases are in children under 10 years of age [2]. This is different than in other European countries where most cases now occur in adults. Probable causes are thought to be an increased susceptibility of children, due to the presence of other diseases, and a low nutritional status. Cases are seen more in male (58%) than in female children and predominantly occur in urban areas (74%) [3].

HIV-*Leishmania* co-infections have been diagnosed in 26 adult patients, out of some 300 HIV/AIDS cases recorded between 1993 and 2010 [3].

CL is also present in Albania, but only a few cases per year are reported. VL may be underreported to some degree. CL is suspected to be underreported as well.

**PARASITOLOGICAL INFORMATION**

| ***Leishmania* species** | **Clinical form** | **Vector species** | **Reservoirs** |
| --- | --- | --- | --- |
| *L. infantum* | ZVL, CL | *P. neglectus, P. perfiliewi, P. tobbi* | *Canis familiaris* |
| *L. major* | ZCL | *P. papatasi* |  |

**MAPS AND TRENDS**

**Visceral leishmaniasis**


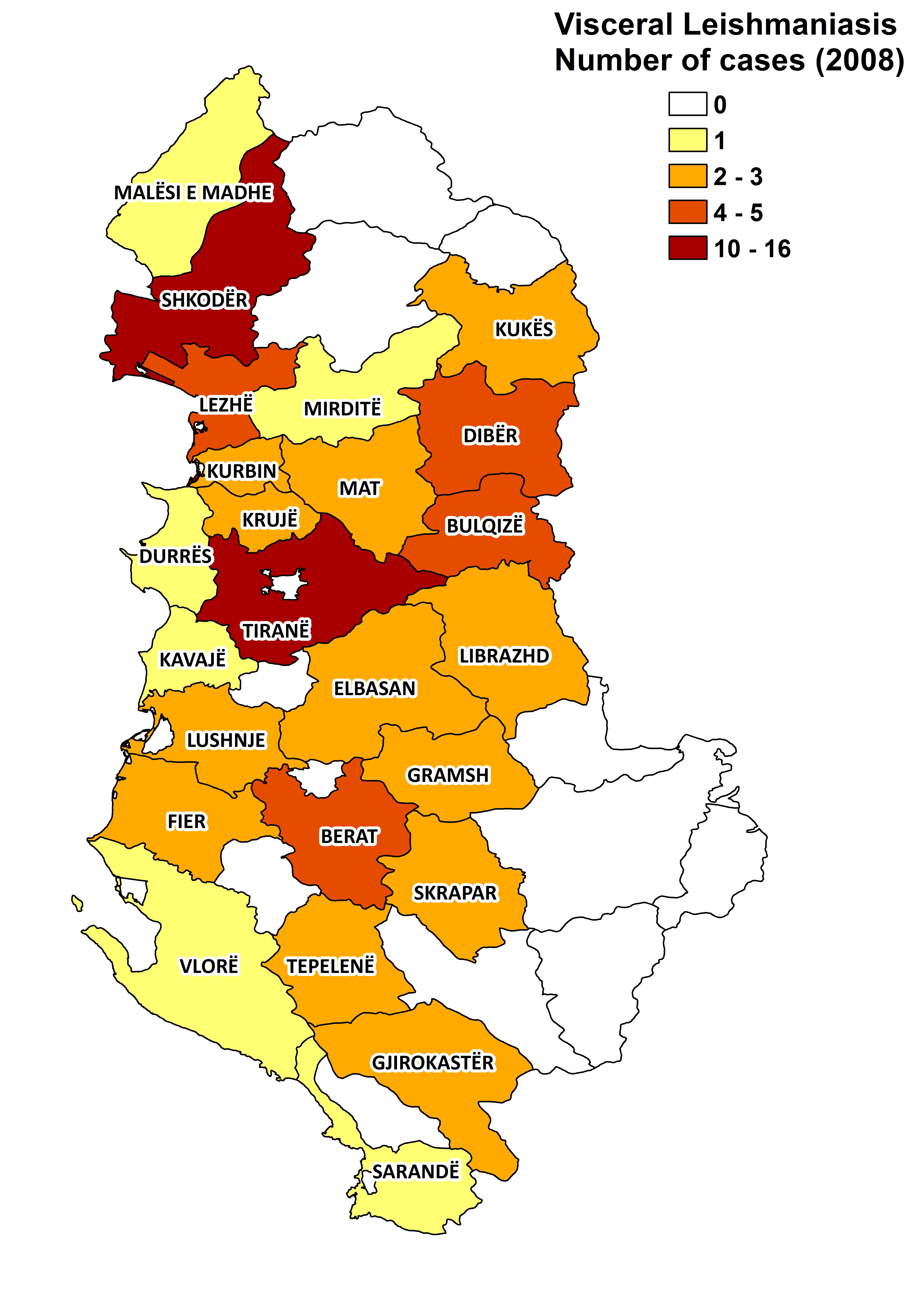

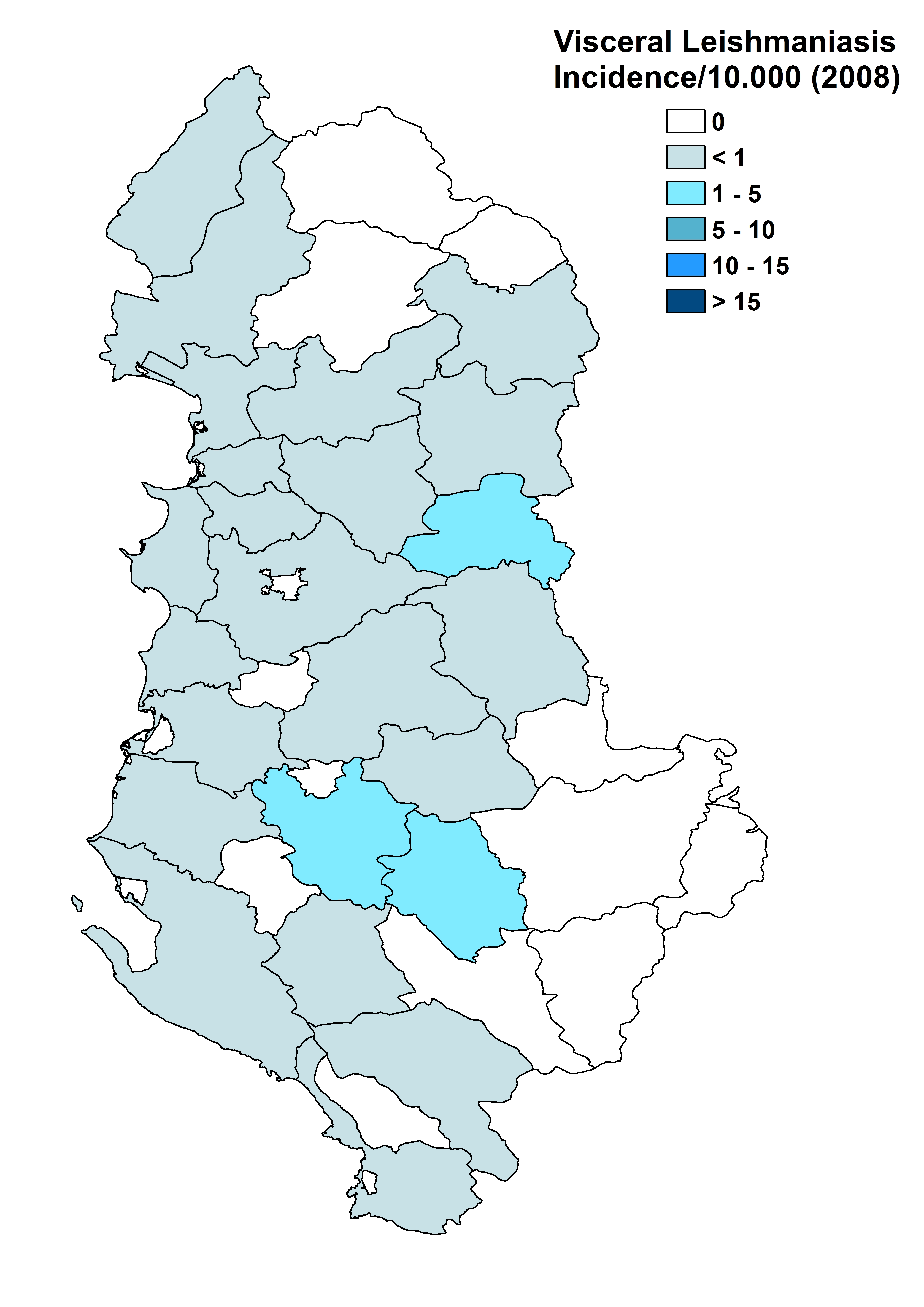


**Cutaneous leishmaniasis**


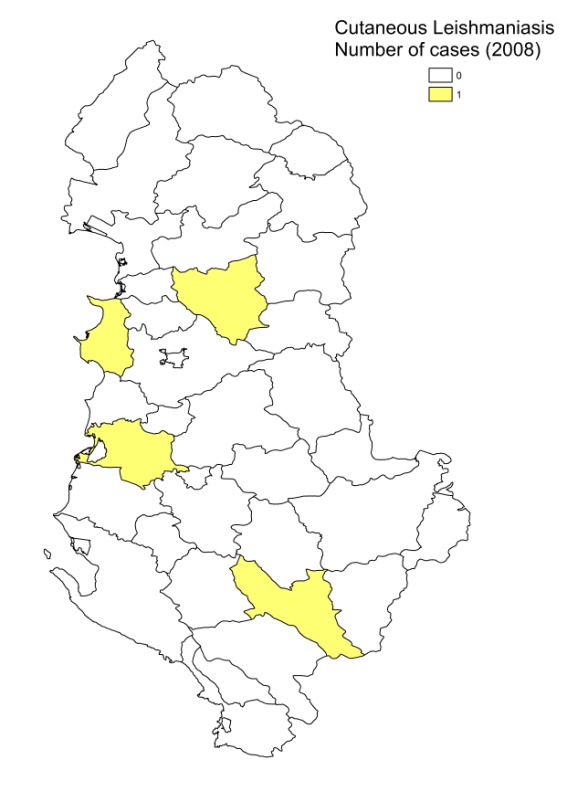


**Visceral leishmaniasis trend**

**Cutaneous leishmaniasis trend**

**CONTROL**

Notification of visceral leishmaniasis is mandatory in the country. There is no national leishmaniasis control program. However, it is planned to be established in 2010. Active human case detection was performed in 2008, and a second round is planned for 2010. There is no reservoir control program, but positive dogs are sacrificed.

**DIAGNOSIS, TREATMENT**

**Diagnosis:**

VL: microscopic examination of bone marrow aspirate. At tertiary level, serological diagnosis is possible (ELISA, IFAT, DAT).

CL: clinical, and confirmation with microscopic examination of skin lesion sample.

**Treatment:**

VL: antimonials, 20 mg Sb^v^/kg/day for 28 days. Cure rate is 98.2%, with 1% relapses and a fatality rate of 0.8%. Second line: AmBisome, 3 mg/kg daily, at day 1-5, day 17 and day 21.

CL: antimonials. Cure rate is 98%, with 2% recurring lesions.

**ACCESS TO CARE**

Treatment for leishmaniasis is officially provided for free (antimonials), but health workers occasionally charge informal payments. Antimonials are not always available in health facilities, in which case patients have to resort to private care. They cannot always afford this. About 5% of patients seek private care.

In case patients need liposomal amphotericin B, they have to pay for it themselves. The Ministry of Health supplied 300 vials of Glucantime (Sanofi) in 2007 and another 300 in 2008. This is only enough for the treatment of 6 patients of 35 kg. Hospitals, therefore, often need to import Glucantime for patients, which is difficult and time consuming, and creates delay in treatment.

VL is diagnosed and treated in district and other hospitals. For CL, diagnosis and treatment is only possible in specialized hospitals. Some patients live in remote areas and cannot afford transport to the hospital, or they suffer major economic consequences by staying away from work.

**ACCESS TO DRUGS**

Meglumine antimoniate is the only drug included in the National Essential Drug List for leishmaniasis. Until 1994, pentamidine was used for VL but was not included in the List. Liposomal amphotericin B (AmBisome, expensive and not reimbursed) is only available on order in the private sector, at 100 Euro per vial.

**SOURCES OF INFORMATION**

- Drs Silva Bino, Raida Petrela, Kela Dikolli, Teita Myrseli, Najada Como, Institute of Public Health, Tirana. *Leishmaniasis in the European Region, a WHO consultative intercountry meeting, Istanbul, Turkey, 17–19 November 2009.*

1. Lita G, Davachi F, Sulcebe G, Bregu H, Basha M (2002). [Pediatric visceral leishmaniasis in Albania.](http://www.ncbi.nlm.nih.gov/pubmed/12044305) Int J Infect Dis 6(1):66-8.

2. Velo E, Bino S, Kuli-Lito G, Pano K, Gradoni L, Sanofi (2003). [Recrudescence of visceral leishmaniasis in Albania: retrospective analysis of cases during 1997 to 2001 and results of an entomological survey carried out during 2001 in some districts.](http://www.ncbi.nlm.nih.gov/pubmed/15228243) Trans R Soc Trop Med Hyg 97(3):288-90.

3. Petrela R, Kuneshka L, Foto E, Zavalani F, Gradoni L (2010). [Pediatric visceral leishmaniasis in Albania: a retrospective analysis of 1,210 consecutive hospitalized patients (1995-2009).](http://www.ncbi.nlm.nih.gov/pubmed/20838650) PLoS Negl Trop Dis 4(9): e814.
