# Supplementary material for: Leishmaniasis Worldwide and Global Estimates of Its Incidence
Source: PLoS One. 2012 May 31;7(5):e35671. doi: 10.1371/journal.pone.0035671 (PMC3365071; doi:10.1371/journal.pone.0035671)
Supplement: Text S3 — Leishmaniasis Country Profiles, Algeria. (DOCX) [file pone.0035671.s003.docx]

**ALGERIA**


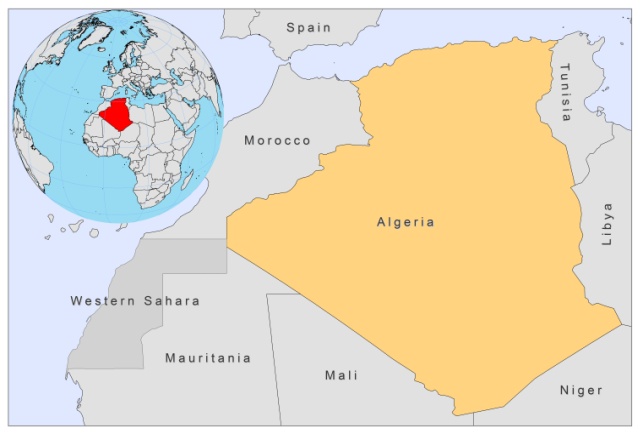


**BASIC COUNTRY DATA**

Total Population: 35,468,208

Population 0-14 years: 27%

Rural population: 34%

Population living under USD 1.25 a day: 6.8%

Population living under the national poverty line: no data

Income status: Upper middle income economy

Ranking: Medium human development (ranking 96)

Per capita total expenditure on health at average exchange rate (US dollar): 191

Life expectancy at birth (years): 73

Healthy life expectancy at birth (years): 61

**BACKGROUND INFORMATION**

The number of VL cases recorded between 1965 and 1974 totaled 497. Between 1975 and 1984, 721 cases were notified, 84% of which were children from 6 months to 4 years old. In the past, only the north (between the Atlas chain and the coast) was endemic and cases were mainly detected in central and eastern parts of the Tell region: principally in the Grande Kabylie, Algerois and Constantinois areas (humid and sub-humid bioclimatic stages) [1]. Since 1980 a resurgence of the number of cases (1,121 cases from 1985 to 1990) and the appearance of new foci have occurred [2,3].

CL is a serious public health problem in Algeria, it being the second largest focus in the world after Afghanistan. This very old cutaneous disease, also called ‘Biskra boil’, used to be mainly endemic in the sub-Saharan steppes (southern Atlas foothills), where the most important foci are located. More recently, however, geographical spread towards the north and west (M'sila, Batna, Ksar Chellala, Djelfa and Bou-Saada) has taken place. In 1984-1985, only a few cases were registered, probably because of the application of DDT in 1983 [4], but since 1986, the number of cases increased rapidly to more than 2,000 per year [3]. Three CL outbreaks occurred in 2004-2006, with respectively 17,100, 32,200 and 15,000 cases.

2.8% of visceral leishmaniasis cases are co-infected with HIV.

**PARASITOLOGICAL INFORMATION**

| ***Leishmania* species** | **Clinical form** | **Vector species** | **Reservoirs** |
| --- | --- | --- | --- |
| *L. infantum* | ZVL, CL | *P. longicuspis, P. perniciosus, P. perfiliewi* | *Canis familiaris* |
| *L. killicki* | CL | Unknown |  |
| *L. major* | ZCL | *P. papatasi* | *Psammomys obesus* |

**MAPS AND TRENDS**

**Cutaneous and visceral leishmaniasis**

**
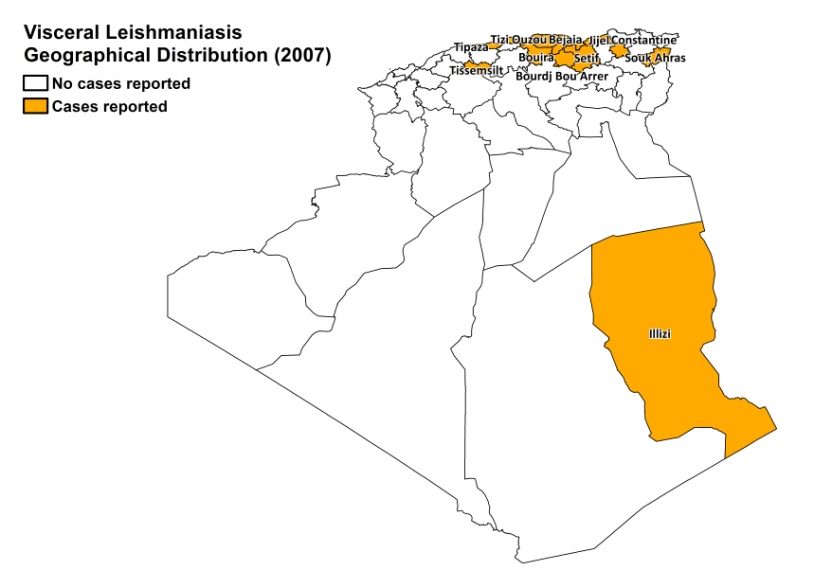

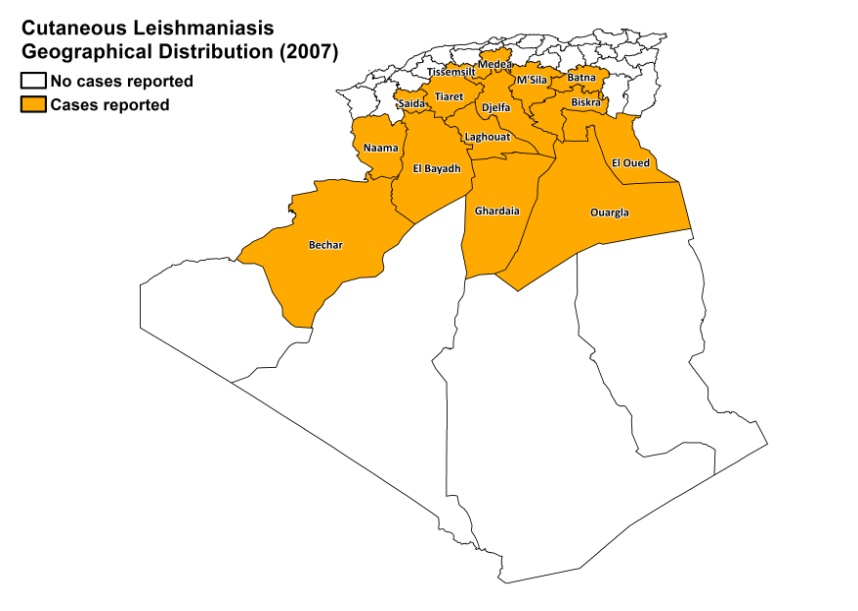
**

**Visceral leishmaniasis trend**

**Cutaneous leishmaniasis trend**

**CONTROL**

The notification of leishmaniasis is mandatory in the country and a national leishmaniasis control program for VL and CL has been in place since 2006. There is a leishmaniasis vector control program. It does not include bednet distribution. Insecticide spraying is regularly done. There is a leishmaniasis reservoir control program. Serological surveys of dogs are occasionally performed. Positive dogs are sacrificed and rodent control is regularly performed.

**DIAGNOSIS, TREATMENT**

**Diagnosis**

VL: confirmation by microscopic examination of tissue sample in specialized centers, cultures, IFAT and Western blotting are used.

CL: on clinical grounds, confirmation by microscopic examination of skin lesion sample.

**Treatment**

VL: antimonials, 20 mg Sb^v^/kg/day for 30 days. Cure rate is 85-90%, with recurring cases in 5-10% and a fatality rate of < 6%.

CL: antimonials, intralesional or systemic, 20 mg Sb^v^/kg/day. The cure rate is 95%, with recurring cases in 2%.

Second line treatment is with conventional amphotericin B.

**ACCESS TO CARE**

Care for leishmaniasis is provided for free. Diagnosis of CL takes place at health centre level. VL can only be diagnosed in hospitals. The private sector is not used for the treatment of leishmaniasis. All patients are believed to have access to care.

**ACCESS TO DRUGS**

Meglumine antimoniate (CL and VL) and conventional amphotericin B (VL) are included in the National Essential Drug List. Drugs for leishmaniasis are not available in pharmacies and unregulated drug markets. Meglumine antimoniate (Glucantime, Sanofi) is registered in Algeria.

**SOURCES OF INFORMATION**

- Drs Seridi Nabila and Boudrissa A, Institut Pasteur d’Algérie.
- Relevé Epidémiologique Mensuel (REM – INSP). INSP: Institut National de Santé Publique.

1. Addadi K, Dedet JP. [Epidemiology of leishmaniasis in Algeria (1976). 6. Survey of clinical cases of infantile visceral leishmaniasis from 1965 to 1974](http://www.ncbi.nlm.nih.gov/pubmed/1036477). Bull Soc Pathol Exot Filiales 69(1):68-75.

2. Harrat Z, Addadi K, Belkaid M, Tabet-Derraz O (1992). [Visceral leishmaniasis in Algeria. Cases reported of visceral leishmaniasis (1985-1990)](http://www.ncbi.nlm.nih.gov/pubmed/1446179). Bull Soc Pathol Exot. 85(4):296-301.

3. Harrat Z, Pratlong F, Belazzoug S, Dereure J, Deniau M et al (1996). Leishmania infantum and L. major in Algeria. Trans R Soc Trop Med Hyg 90(6):625-9.

4. [Benzerroug EH, Benhabylles N, Izri MA, Belahcene EK (1992). Indoor and outdoor nebulization of DDT in the campaign against cutaneous zoonotic leishmaniasis in Algeria](http://www.ncbi.nlm.nih.gov/pubmed/1567269). Ann Soc Belg Med Trop 72(1):5-12.
