# Supplementary material for: Leishmaniasis Worldwide and Global Estimates of Its Incidence
Source: PLoS One. 2012 May 31;7(5):e35671. doi: 10.1371/journal.pone.0035671 (PMC3365071; doi:10.1371/journal.pone.0035671)
Supplement: Text S4 — Leishmaniasis Country Profiles, Argentina. (DOCX) [file pone.0035671.s004.docx]

**ARGENTINA**


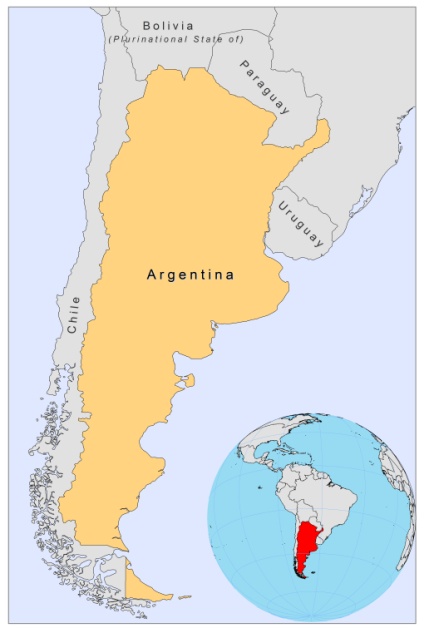


**BASIC COUNTRY DATA**

Total Population: 40,412,376

Population 0-14 years: 25%

Rural population: 8%

Population living under USD 1.25 a day: 0.9%

Population living under the national poverty line: no data

Income status: Upper middle income economy

Ranking: Very high human development (ranking 45)

Per capita total expenditure on health at average exchange rate (US dollar): 730

Life expectancy at birth (years): 75

Healthy life expectancy at birth (years): 65

**BACKGROUND INFORMATION**

Although CL caused by *L. braziliensis* is widely distributed, transmission tends to be within micro-foci and is epidemic. Between 1995-2004, 426.6 cases per year were reported [1]. Since 2000 a total of 2,527 cases of CL have been registered, most of them concentrated in northwest Argentina. Three eco-epidemiological areas can be recognized. First, in the Yungas area, CL is associated with forest activities, but is changing to a peri-urban [1,2] and even an urban transmission [3]. Within an endemic pattern of up to 30 cases per 1,000 inhabitants in some localities of Salta province [2], outbreaks have been reported [4], associated with outdoor activities, poor housing and sleeping outside [2]. In Salta, almost 20% of the dogs harbour skin lesions compatible with leishmaniasis and 27% are seropositive, suggesting their possible role as *L.braziliensis* reservoir [5]. In the second largest area, the subtropical forest along the Paraná river, in the province of Corrientes, the number of recorded CL cases used to be very small, but increased from an average of 2 cases per year in the period 1955-1974 to 12 cases per year between 1995 and 2004, eventually leading to urban outbreaks [6]. The touristic Iguazú Falls, in the Misiones province, belong to the same ecological transmission pattern [7]. The third region is Chaco, which has a xerophylic vegetation. In the province of Formosa, 85 cases have been reported in the period 1992-2001, 10.5% of which with mucosal involvement [8]. Higher incidences in outside workers and peridomestic transmission have been described.

Epidemic peaks have been recorded in 1985, 1998 and in 2002, with a rising trend. The last large CL outbreak took place in 2002, with 507 cases in Salta and 102 in Formosa. Mucocutaneous cases are fairly common with 2.6% of all CL cases [9] (25 reported cases) in 2009.

The first case of VL was described in 1926 in the Chaco region. Until 1989, 14 cases were reported in the Salta province, interspersed within the endemic area of CL and probably caused by *Leishmania* dermotropic species. However, the sharp onset of VL in nearby Asunción, Paraguay, in 2000 and the local presence of *Lu. longipalpis* allowed for the establishment of a new autochtonous urban focus in the city of Posadas, in the Misiones province [10], with yearly increasing case numbers. Between 2006 and 2009, 54 cases were reported in this focus. Out of 110 tested dogs in Posadas 47% yielded positive PCR [11].

CL and VL are both suspected to be underreported to some degree due to the lack of awareness of health workers, the recent new urban focus of VL and an increasing number of infected dogs.

There are 2 reported cases of HIV/*Leishmania* co-infection.

**PARASITOLOGICAL INFORMATION**

| ***Leishmania* species** | **Clinical form** | **Vector species** | **Reservoirs** |
| --- | --- | --- | --- |
| *L. guyanensis* | ZCL | unknown | unknown |
| *L. amazonensis* | ZCL | unknown | unknown |
| *L. braziliensis* | ZCL, MCL | *Lu. whitmani,*  *Lu. neivai,*  *Lu. migonei* | *Canis familiaris* |
| *L. infantum* | ZVL | *Lu. longipalpis* | *Canis familiaris* |

**MAPS AND TRENDS**

**Visceral leishmaniasis**

**
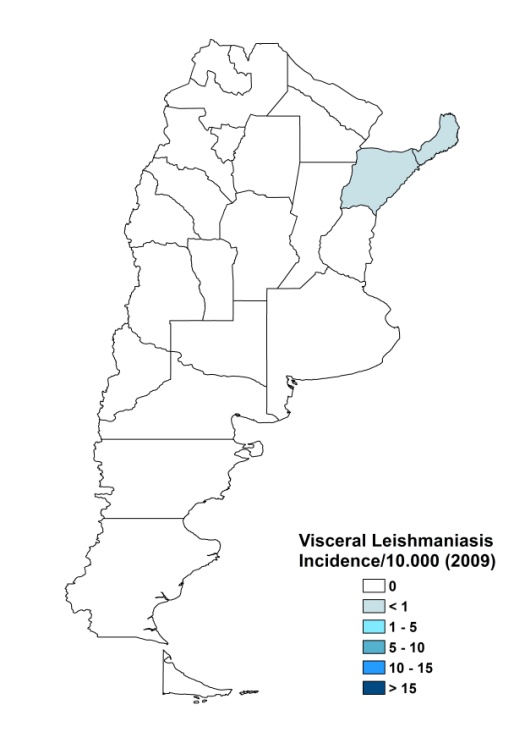

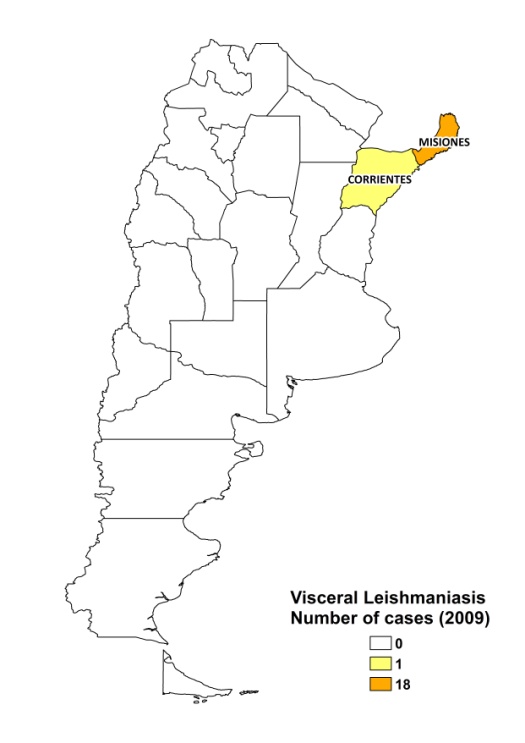
**

**Cutaneous leishmaniasis**


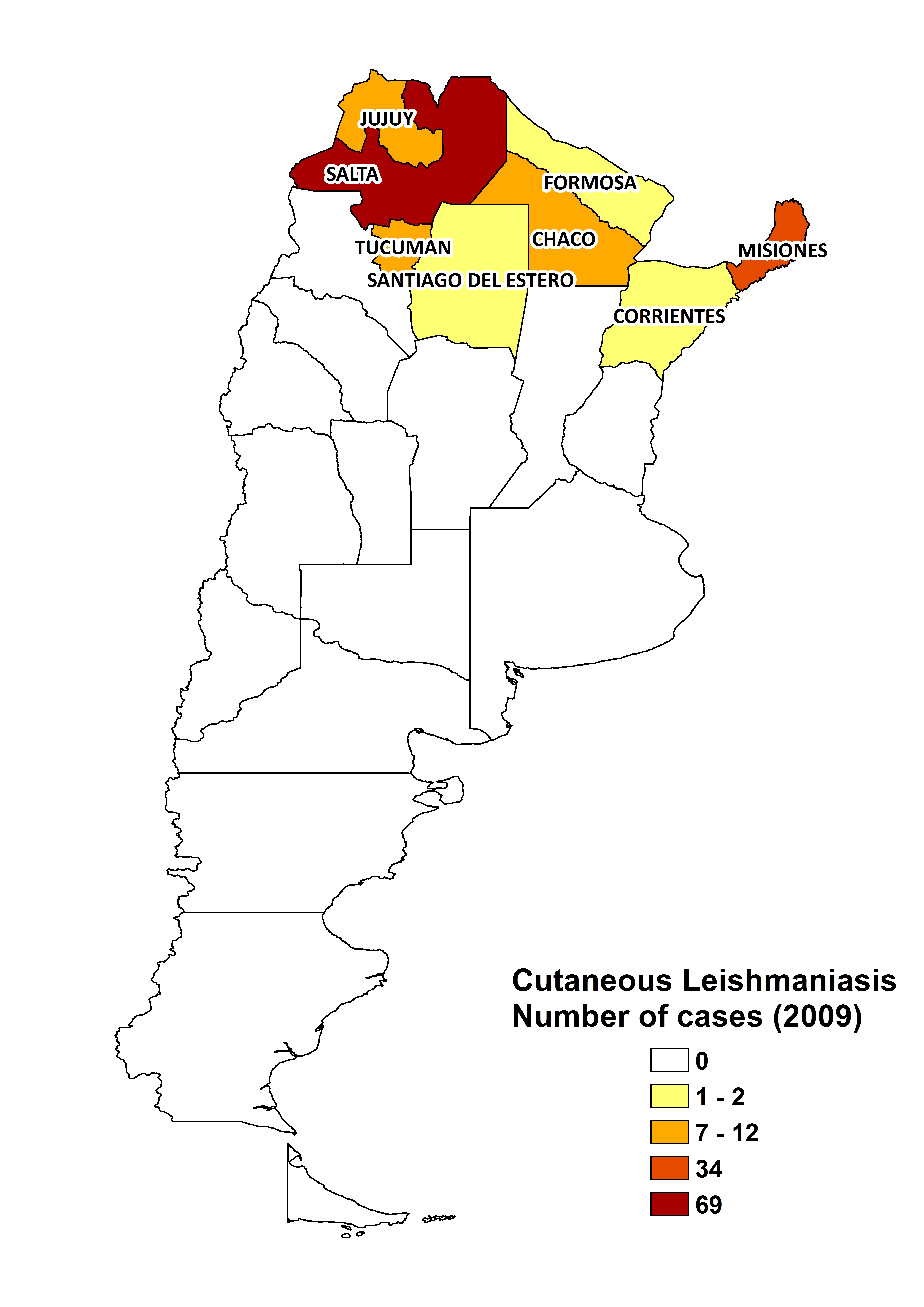

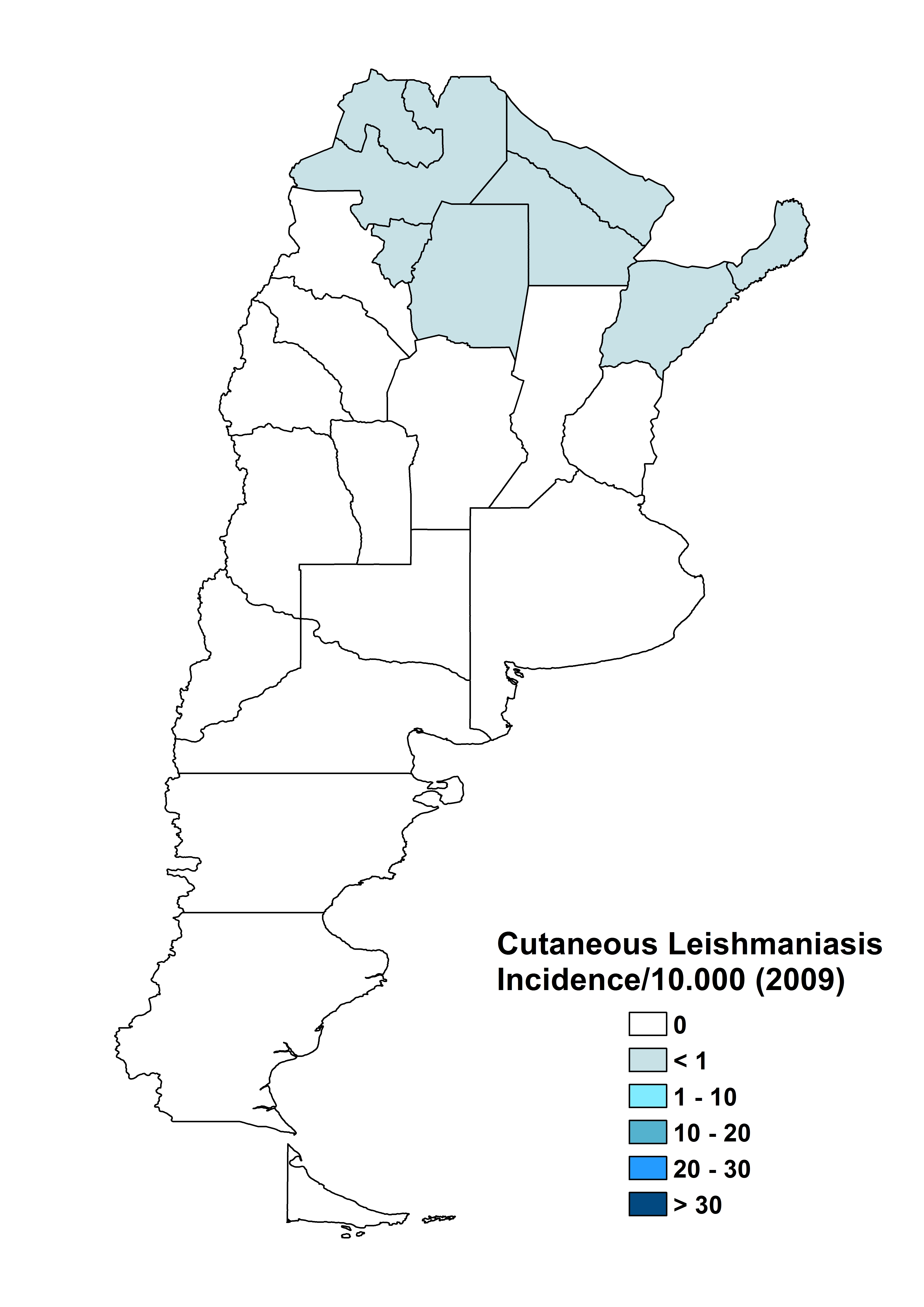


**Visceral leishmaniasis trend**

**Cutaneous leishmaniasis trend**

**CONTROL**

Notification of leishmaniasis has been mandatory since 1964 and a national leishmaniasis control program has existed since 1998. There is a vector control program and a reservoir control program has recently been put in place for VL. Sacrifice of positive dogs is recommended.

**DIAGNOSIS, TREATMENT**

**Diagnosis:**

VL: serological, by rK39 dipsticks (at district hospital level) and parasitological, by microscopic examination of bone marrow aspirate or PCR in National Reference Centers.

CL: on clinical picture, parasitological confirmation by microscopic examination of skin lesion sample or PCR in National Reference Centers.

**Treatment:**

VL: antimonials, 20 mg Sb^v^/kg/day for 30 days. Second line: liposomal or lipid amphotericin B (total dose 20 mg/kg). Fatality rate is 8%.

CL: antimonials, 20 mg Sb^v^/kg/day for 21 days. Cure rate is > 95%. In 10% of patients, recurring lesions occur and in < 10% there is mucosal involvement. Second line: conventional amphotericin B (total dose ~1,5 g).

MCL: antimonials, 20 mg Sb^v^/kg/day for 30 days. Cure rate is > 85%. Recurring lesions occur in > 25% of patients. Second line: conventional amphotericin B (total dose ~2-3 g). Fatality rate is > 1%.

**ACCESS TO CARE**

Care for leishmaniasis is provided for free. The private sector is not used by patients for treatment. The government purchased sufficient antimonials (Glucantime, Sanofi) for all reported patients in 2008. Since 2009, amphotericin B lipid complex (Abelcet ®, Cephalon) has also been provided. However, the supply of drugs has not been continuous at health facilities due to problems with both their costs and their suppliers.

Diagnosis of suspected VL takes place at health centres and patients are referred to hospitals where diagnosis is confirmed. Treatment of VL takes place only in secondary and tertiary hospitals. CL can be diagnosed on clinical grounds and treated at health centre level. There is a lack of trained human resources for treating leishmaniasis. For patients in very remote areas, access to care is problematic due to transport that is either too expensive or not available.

**ACCESS TO DRUGS**

Meglumine antimoniate and sodium stibogluconate (for VL and CL) and amphotericin B (liposomal and conventional, for VL) are included in the National Essential Drug List. Drugs for leishmaniasis are not available at pharmacies or drug markets. Glucantime (Sanofi) is not registered in Argentina, but milltefosine (Paladin, Canada) is. A generic of meglumine antimoniate is produced in Argentina.

**SOURCES OF INFORMATION**

- Dr. Oscar Daniel Salomón - CeNDIE-ANLIS-Ministry of Health. National System of Epidemiologic Surveillance, Ministry of Health. *Leishmaniasis en la Región de las Américas. Reunión de coordinadores de Programa Nacional de Leishmaniasis. OPS/OMS. Medellín, Colombia. 4-6 junio 2008.*

1. Sosa-Estani S, Segura EL, Salomón OD, Gómez A, Peralta M et al (2000). [Tegumentary leishmaniasis in Northern Argentina: distribution of infection and disease, in three municipalities of Salta, 1990-1992.](http://www.ncbi.nlm.nih.gov/pubmed/11175588) Rev Soc Bras Med Trop 33(6):573-82.

2. Sosa-Estani S, Segura EL, Gomez A, Salomón OD, Peralta M et al (2001) [Cutaneous leishmaniasis in Northern Argentina: identification of risk factors in a case-cohort study of three municipalities in Salta.](http://www.ncbi.nlm.nih.gov/pubmed/11813056) Rev Soc Bras Med Trop 34(6):511-7.

3. Gil JF, Nasser JR, Cajal SP, Juarez M, Acosta N et al (2010). Am J Trop Med Hyg 82(3):433-40.

4. Salomón OD, Zaidenberg M, Burgos R, Heredia VI, Caropresi SL (2001). [American cutaneous leishmaniasis outbreak, Tartagal city, province of Salta, Argentina, 1993.](http://www.ncbi.nlm.nih.gov/pubmed/11340485) Rev Inst Med Trop Sao Paulo 43(2):105-8.

5. Padilla AM, Marco JD, Diosque P, Segura MA, Mora MC et al (2002). [Canine infection and the possible role of dogs in the transmission of American tegumentary leishmaniosis in Salta, Argentina.](http://www.ncbi.nlm.nih.gov/pubmed/12446084) Vet Parasitol 110(1-2):1-10.

6. Salomón OD, Sosa-Estani S, Ramos K, Orellano PW, Sanguesa G et al (2006). [Tegumentary leishmaniasis outbreak in Bella Vista City, Corrientes, Argentina during 2003.](http://www.ncbi.nlm.nih.gov/pubmed/17160285) Mem Inst Oswaldo Cruz 101(7):767-74.

7. Salomón OD, Acardi SA, Liotta DJ, Fernández MS, Lestani E, López D et al (2009). [Epidemiological aspects of cutaneous leishmaniasis in the Iguazú falls area of Argentina.](http://www.ncbi.nlm.nih.gov/pubmed/18983809) Acta Trop 109(1):5-11.

8. Salomón OD, Sosa Estani S, Dri L, Donnet M, Galarza R et al (2002). [Tegumental leishmaniasis in Las Lomitas, Province of Formosa, Argentina, 1992-2001.](http://www.ncbi.nlm.nih.gov/pubmed/12532691) Medicina (B Aires) 62(6):562-8.

9. Sosa Estani S, Campanini A, Sinagra A, Luna C, Peralta M et al. [Clinical features and diagnosis of mucocutaneous leishmaniasis in patients of an endemic area in Salta.](http://www.ncbi.nlm.nih.gov/pubmed/10347960) Medicina (B Aires). 1998;58(6):685-91.

10. Salomón O, Sinagra A, Nevot M, Barberian G, Paulin P et al (2008). [First visceral leishmaniasis focus in Argentina.](http://www.ncbi.nlm.nih.gov/pubmed/18368242) Mem Inst Oswaldo Cruz 103(1):109-11.

11. Cruz I, Acosta L, Gutiérrez MN, Nieto J, Cañavate C et al (2010). [A canine leishmaniasis pilot survey in an emerging focus of visceral leishmaniasis: Posadas (Misiones, Argentina).](http://www.ncbi.nlm.nih.gov/pubmed/21122107) BMC Infect Dis 10:342.
