# Supplementary material for: Leishmaniasis Worldwide and Global Estimates of Its Incidence
Source: PLoS One. 2012 May 31;7(5):e35671. doi: 10.1371/journal.pone.0035671 (PMC3365071; doi:10.1371/journal.pone.0035671)
Supplement: Text S5 — Leishmaniasis Country Profiles, Armenia. (DOCX) [file pone.0035671.s005.docx]

**ARMENIA**

**
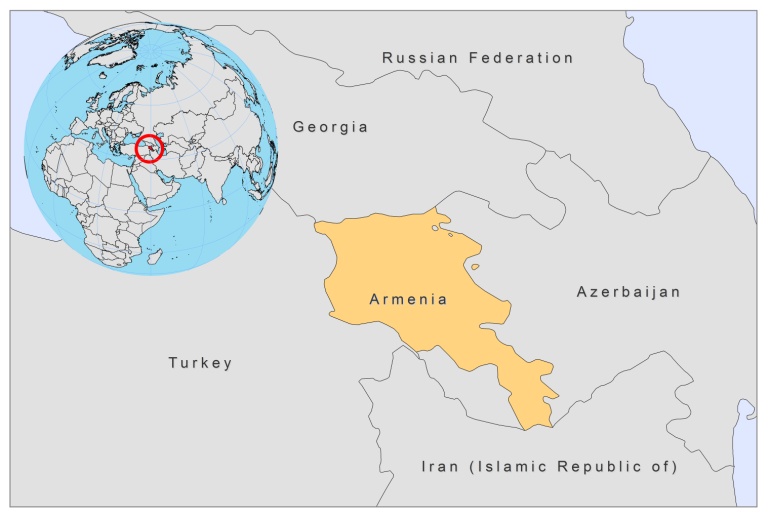
**

**BASIC COUNTRY DATA**

Total Population: 3,092,072

Population 0-14 years: 20%

Rural population: 36%

Population living under USD 1.25 a day: 1.3%

Population living under the national poverty line: 26.5%

Income status: Lower middle income economy

Ranking: High human development (ranking 86)

Per capita total expenditure on health at average exchange rate (US dollar): 129

Life expectancy at birth (years): 74

Healthy life expectancy at birth (years): 61

**BACKGROUND INFORMATION**

The first CL case was reported in Armenia in 1920. CL occurred mainly in the south of the country. Between 1938 and 1970, 133 CL patients have been reported. CL has not been reported since, apart from one probable autochtonous case in 1999. To date, no more cases have been registered.

The first VL case (by *L infantum*) was registered in Armenia in 1913. From 1926 to 1970, 919 VL cases were registered. Between 1949 and 1956, the incidence increased and 486 cases were registered, mainly in children under the age of 13. Cases were reported in 16 regions (at 700–1580m above sea level), about 80% of which occurred around the city of Yerevan. Extensive studies of over 4,000 animals revealed that foxes and dogs are natural reservoirs. Elimination of stray dogs and vector control in 1954-55 decreased the case load sharply and after 1969 no more cases were found. In 1999, an autochtonous VL case was again recorded in a 4-year-old child. Since then, cases have increased up to 13 in 2008 and 10 between January-November 2009. Most of the cases are young children (under 5) and occur in areas bordering Afghanistan, Georgia, Iran and Turkey. In 2006 and 2008, two cases were fatal, probably due to late diagnosis and treatment.

Underreporting of VL is suspected to be a serious problem. There is a low awareness of the disease, among patients and doctors. Probably significant numbers of patients are misdiagnosed and remain untreated for VL. In 2008 and 2009, training programs for doctors were started in order to address this problem.

*Leishmania*/HIV co-infection has not been reported.

**PARASITOLOGICAL INFORMATION**

| ***Leishmania* species** | **Clinical form** | **Vector species** | **Reservoirs** |
| --- | --- | --- | --- |
| *L. infantum* | ZVL, CL | *P. kandelakii, P. balcanicus* | *Canis familiaris* |

**MAPS AND TRENDS**

**Visceral leishmaniasis**


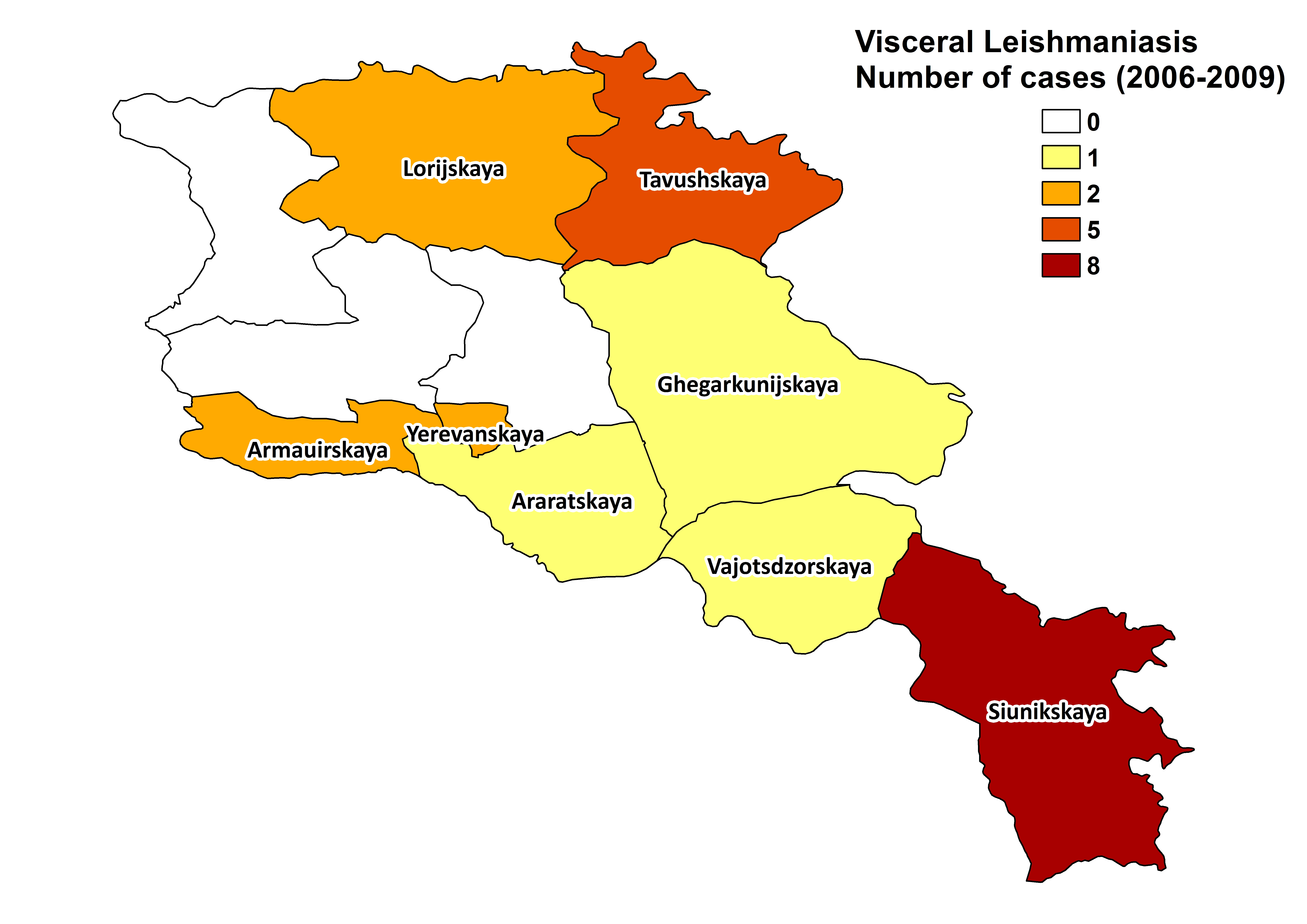

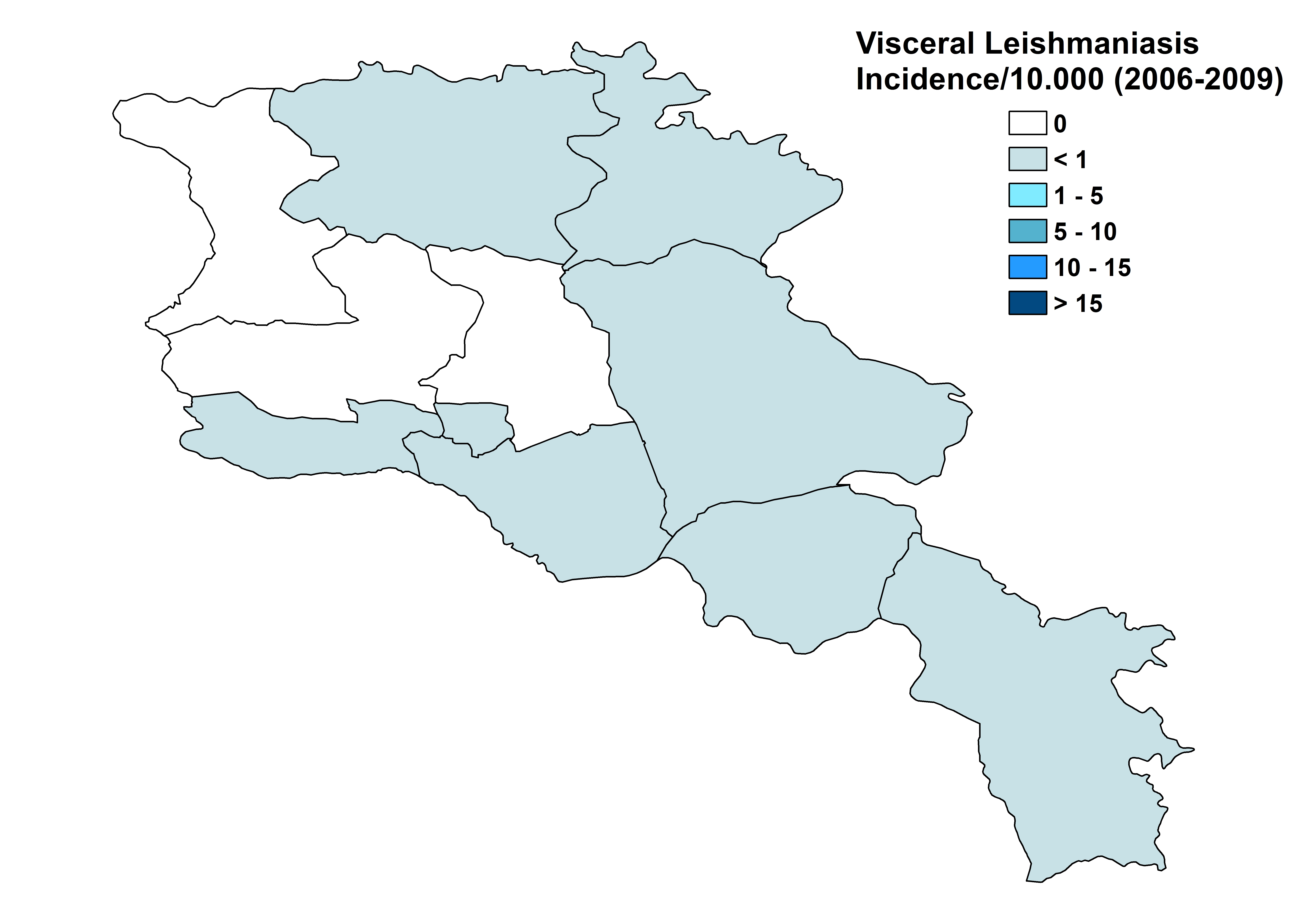


**Visceral leishmaniasis trend**

**CONTROL**

The notification of leishmaniasis is not mandatory and there is no national leishmaniasis control program. There is no leishmaniasis vector control program and no bednet distribution program. Insecticide spraying is regularly done (for malaria). There is no leishmaniasis reservoir control program.

**DIAGNOSIS, TREATMENT**

**Diagnosis:**

VL: Microscopic examination of bone marrow aspirate and serology (ELISA).

**Treatment:**

VL: Antimonials, 20 mg Sb^v^/kg/day for 14 days. Relapses have been reported.

**ACCESS TO CARE**

Diagnosis of VL is only possible in two specialized centers and treatment only in one hospital, in the capital of Armenia. There is no national protocol and the duration of treatment used in this hospital is too short, leading to relapses. Due to a lack of knowledge of the disease in the country among doctors as well as patients, VL is often reported very late, up to one year after onset.

**ACCESS TO DRUGS**

Meglumine antimoniate is on the National Essential Drug List. It is, however, not registered in Armenia. Therefore, it is not available. Care for leishmaniasis is provided for free in Armenia, but patients have to purchase antimonials themselves from unregulated drug markets. Glucantime (Sanofi) costs 11 USD per vial (5 ml), which would lead to a treatment cost of about 500 USD for a full adult treatment. The price of the drugs is a serious problem, as most patients are poor. A generic form of meglumine antimoniate, produced in Iran, is also available via unregulated drug markets. Glucantime (Sanofi) is planned to be registered.

**SOURCES OF INFORMATION**

- Drs Ara Keshishyan and Romella Abovyan. State Hygienic and Anti-Epidemic Inspectorate MoH.National Center for Diseases Control and Prevention, Ministry of Health, Yerevan. *Leishmaniasis in the European Region, a WHO consultative intercountry meeting, Istanbul, Turkey, 17–19 November 2009.*
