# Supplementary material for: Leishmaniasis Worldwide and Global Estimates of Its Incidence
Source: PLoS One. 2012 May 31;7(5):e35671. doi: 10.1371/journal.pone.0035671 (PMC3365071; doi:10.1371/journal.pone.0035671)
Supplement: Text S6 — Leishmaniasis Country Profiles, Azerbijan. (DOCX) [file pone.0035671.s006.docx]

**AZERBAIJAN**


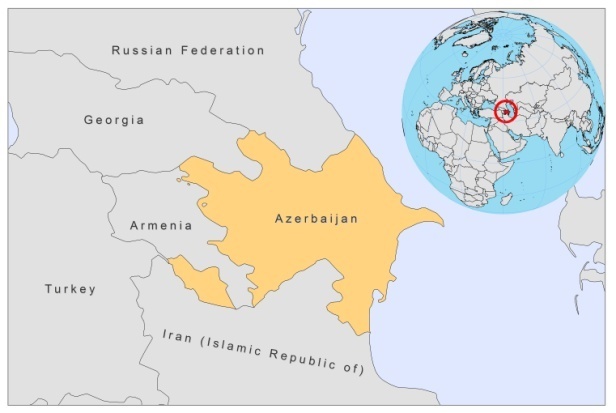


**BASIC COUNTRY DATA**

Total Population: 9,047,932

Population 0-14 years: 21%

Rural population: 48%

Population living under USD 1.25 a day: 1%

Population living under the national poverty line: 15.8%

Income status: Upper middle income economy

Ranking: High human development (ranking 91)

Per capita total expenditure on health at average exchange rate (US dollar): 285

Life expectancy at birth (years): 70

Healthy life expectancy at birth (years): 57

**BACKGROUND INFORMATION**

Azerbaijan used to be endemic for CL by *L tropica*. Over 2,000 cases were registered in the 1950s, but almost none in 1966. After a long period of the absence of any leishmaniasis morbidity in Azerbaijan, in 1987 new cases of CL were registered in Sharvan (Kura- Arax) valley, situated in the western and central part of the country [1]. During 1987-1988, an outbreak of CL occurred with 68 patients in Geokchai district, situated in the same zone. *L. Infantum* was identified as the causative parasite in this outbreak [2]. However, autochtonous cases of *L. major* and *L. tropica* have been identified again in Azerbaijan as well. The disease incidence in 1990 was almost ten times higher than in 1985 [3]. During the period 1989-1997, 1,340 cases of CL were registered in the country, decreasing to 257 cases during the period of 1998-2009.

The first VL case was recorded in 1912; 186 further cases were registered until 1956. The disease was endemic in areas bordering the Russian Federation and Armenia, as well as in the south of the country. In 1957, a Leishmaniasis Control Program was launched and consequently, the number of cases dropped. Until 1988, only single cases have been recorded, but the incidence increased after the breakdown of the Soviet Union, just like it did with CL. During the period 1970-1979, 145 cases were recorded and 347 cases in the period 1989-2009. Most foci of VL are located at 500 m above sea level and most cases are in very young children.

For both VL and CL, the figures do not reflect the real morbidity rate because they are based on passive case detection. Both VL and CL are probably underreported to a large degree in Azerbaijan.

There are no reported cases of HIV/*Leishmania* co-infection.

**PARASITOLOGICAL INFORMATION**

| ***Leishmania* species** | **Clinical form** | **Vector species** | **Reservoirs** |
| --- | --- | --- | --- |
| *L. infantum* | ZVL, CL | *P. kandelakii, P. transcaucasicus* | *Canis familiaris, Vulpes vulpes* |
| *L. major* | ZCL | *P. papatasi* | *Rhombomys opimus* |
| *L. killicki* | CL | unknown |  |

**MAPS AND TRENDS**

**Visceral leishmaniasis**

**
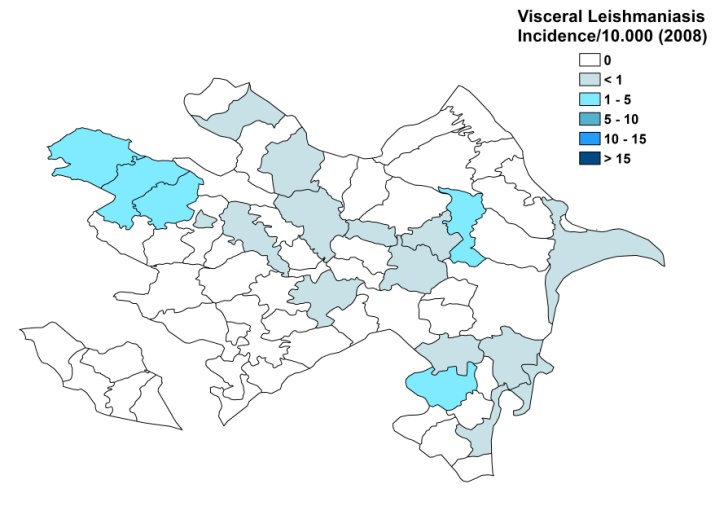

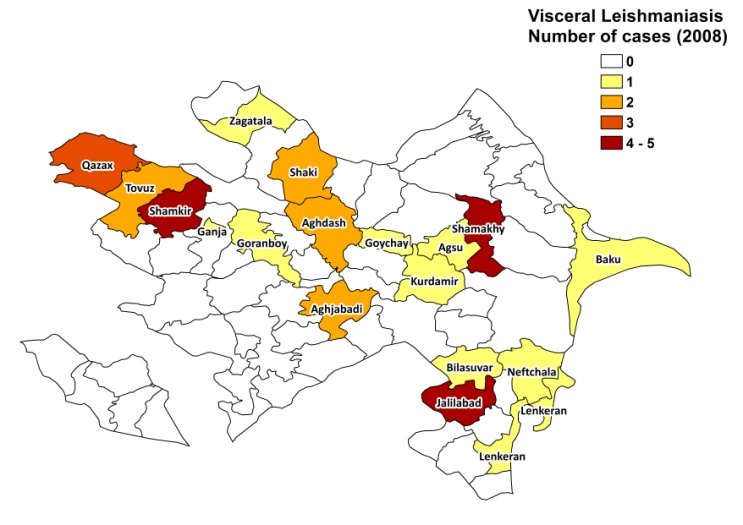
**

**Cutaneous leishmaniasis**

**
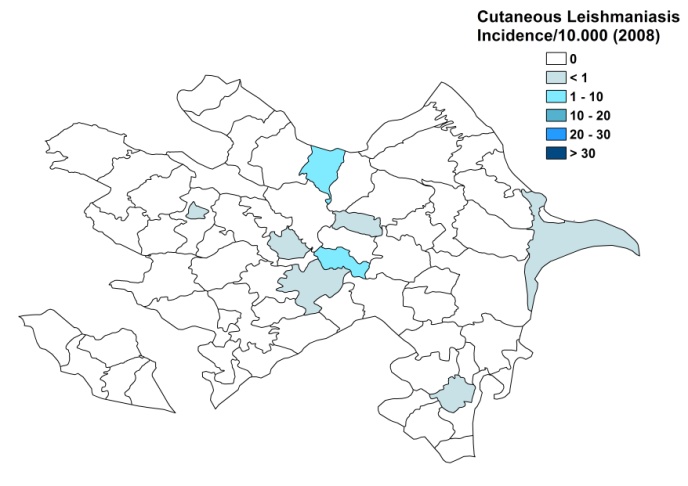

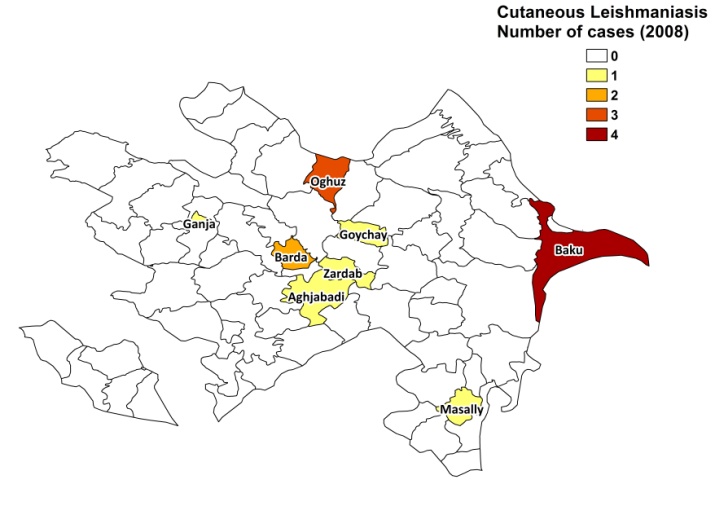
**

**Visceral leishmaniasis trend**

**Cutaneous leishmaniasis trend**

**CONTROL**

The notification of leishmaniasis is mandatory. There is no national leishmaniasis control program, but it is expected to be established in 2010, both for VL and CL. Active human case detection is not regularly performed. There is no leishmaniasis vector control program. Bednet distribution and insecticide spraying take place under the National Malaria Elimination Program. There is no formal leishmaniasis reservoir control program, but stray dogs are sacrificed and rodent control is regularly performed.

**DIAGNOSIS, TREATMENT**

**Diagnosis:**

CL: mainly on clinical picture, in rare cases confirmation with microscopic examination of skin lesion sample is made.

VL: microscopic examination of bone marrow aspirate.

**Treatment:**

VL and complicated CL: antimonials, 20 mg Sb^v^/kg/day for 14-17 days.

Cure rate for VL: 90%, with 10% relapses.

Cure rate for CL: 100%.

**ACCESS TO CARE**

Not all patients have access to treatment for leishmaniasis. Diagnosis and treatment for VL is only offered at advanced health care levels. There are only two laboratory experts able to identify *Leishmania* parasites. There is no national protocol and the duration of treatment used for VL is too short, which causes relapses. CL is mostly diagnosed on clinical grounds and treated in local clinics with traditional methods or antibiotics (amphotericin B, erythromycin). Antimonials are only used in complicated cases.

**ACCESS TO DRUGS**

Meglumine antimoniate is on the National Essential Drug List. It is, however, not registered and it is not available in health centers. Although care for leishmaniasis is provided for free, patients have to purchase the antimonials themselves, but cannot always afford them. Antimonials are available at private pharmacies (Glucantime from Sanofi and a generic meglumine antimoniate form, produced in Iran) or patients buy the drugs abroad.

**SOURCES OF INFORMATION**

- Dr Faramazov Abdulla Zikrulla, Azerbaijan Medical University, National Research Institute V.Y. Akhundov. *Leishmaniasis in the European Region, a WHO consultative inter-country meeting, Istanbul, Turkey, 17–19 November 2009.*
- Republic Centre of Hygiene and Epidemiology, National AIDS Center.

1. Edrissian GH, Hafizi A, Afshar A, Soleiman-Zadeh G, Movahed-Danesh AM et al (1988). An endemic focus of visceral leishmaniasis in Meshkin-Shahr, east Azerbaijan province, north-west part of Iran and IFA serological survey of the disease in this area. Bull Soc Pathol Exot Filiales 81(2):238-48.

2. Gasanzade GB, Saf'ianova VM, Tagi-zade TA, Agaev A, Gadzhibekova EA et al (1990). [An outbreak of cutaneous leishmaniasis caused by Leishmania infantum in Geokchaĭ District, Azerbaijan SSR](http://www.ncbi.nlm.nih.gov/pubmed/2142993). Med Parazitol (Mosk) (2):41-5. Russian.

3. Soleimanzadeh G, Edrissian GH, Movahhed-Danesh AM, Nadim A (1993). [Epidemiological aspects of kala-azar in Meshkin-Shahr, Iran: human infection.](http://www.ncbi.nlm.nih.gov/pubmed/8313493) Bull World Health Organ 71(6):759-62.
