# Supplementary material for: Leishmaniasis Worldwide and Global Estimates of Its Incidence
Source: PLoS One. 2012 May 31;7(5):e35671. doi: 10.1371/journal.pone.0035671 (PMC3365071; doi:10.1371/journal.pone.0035671)
Supplement: Text S7 — Leishmaniasis Country Profiles, Bangladesh. (DOCX) [file pone.0035671.s007.docx]

**BANGLADESH**

**
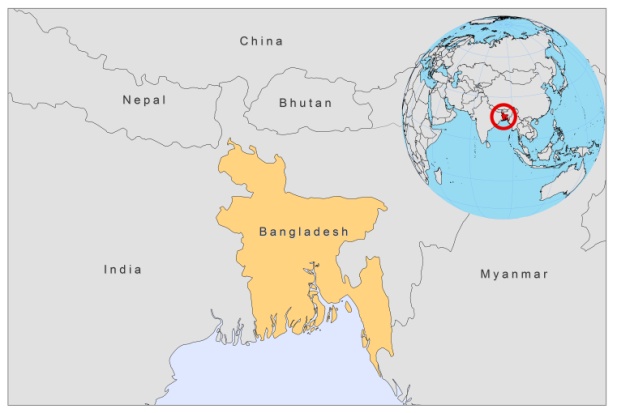
**

**BASIC COUNTRY DATA**

Total Population: 148,692,131

Population 0-14 years: 31%

Rural population: 72%

Population living under USD 1.25 a day: 49.6%

Population living under the national poverty line: no data

Income status: Low income economy

Ranking: Low human development (ranking 146)

Per capita total expenditure on health at average exchange rate (US dollar): 19

Life expectancy at birth (years): 68

Healthy life expectancy at birth (years): 54

**BACKGROUND INFORMATION**

VL was first described in 1824 in Jessore district, in what is now Bangladesh [1]. Subsequently, the disease spread to other districts and in the 19^th^ and early 20^th^ centuries, epidemic outbreaks of VL, with case fatality rates of over 95%, occurred every 15-20 years in a wide range of districts. An epidemic outbreak in Jessore killed 75,000 people between 1824 and 1827. Between 1931 and 1943, more than 1,000,000 VL cases were reported in former Bengal [1]. The case numbers declined during the DDT spraying campaign of the National Malaria Eradication Program and VL was thought to be eliminated after the 1970s. Between 1968 and 1980, only 59 cases were reported in the country [2]. But since the 1980s, following the suppression of DDT spraying, there has been a resurgence of VL, with 73,467 cases reported between 1994 and 2004 [1]. VL is now endemic in many Bangladeshi areas, with the Mymensingh district representing over 50% of the cases. There is substantial underreporting. In 2007, the estimated number of active cases was 136,500 [3]. However, less than 5,000 cases were reported that same year. The estimated incidence of VL, according to recent studies, is 15.6/1,000 person-years in Fulbaria [4] and 27/10,000 population in Godagari and Rajshahi [5].

VL is focal, clustered within households, and mostly affects the poorest of the poor. VL is known to prolong the poverty cycle through loss of productivity and unaffordable treatment costs for the affected families. Risk factors for infection include living in or within 50 m from the same household as an active case (which multiplies the risk of infection by 25), but also include poverty related factors such as poor nutrition and housing conditions, with cracked mud walls [6].

Humans seem to be the only reservoir, with infected patients and PKDL cases constituting the source of infection. PKDL remains mostly untreated, and its prevalence rose from 1 per 10,000 person-years in 2002-2004 to an estimated 21 per 10,000 person-years in 2007 [7]. A later survey, performed in 2008, showed a prevalence of 6,2/10,000 population [8]. This high PKDL rate is thought to have a negative impact on the prospect of VL elimination in Bangladesh.

No cases of HIV*-Leishmani*a co-infection have been reported.

No data on CL are available in Bangladesh.

**PARASITOLOGICAL INFORMATION**

| ***Leishmania* species** | **Clinical form** | **Vector species** | **Reservoirs** |
| --- | --- | --- | --- |
| *L. donovani* | AVL, PKDL | *P.argentipes* | Human |


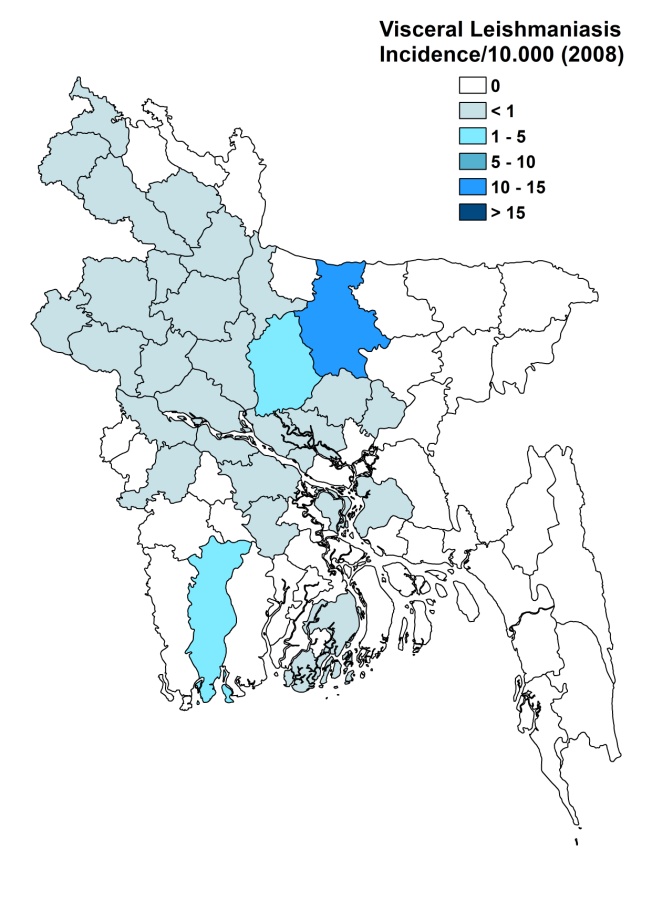
**MAPS AND TRENDS**


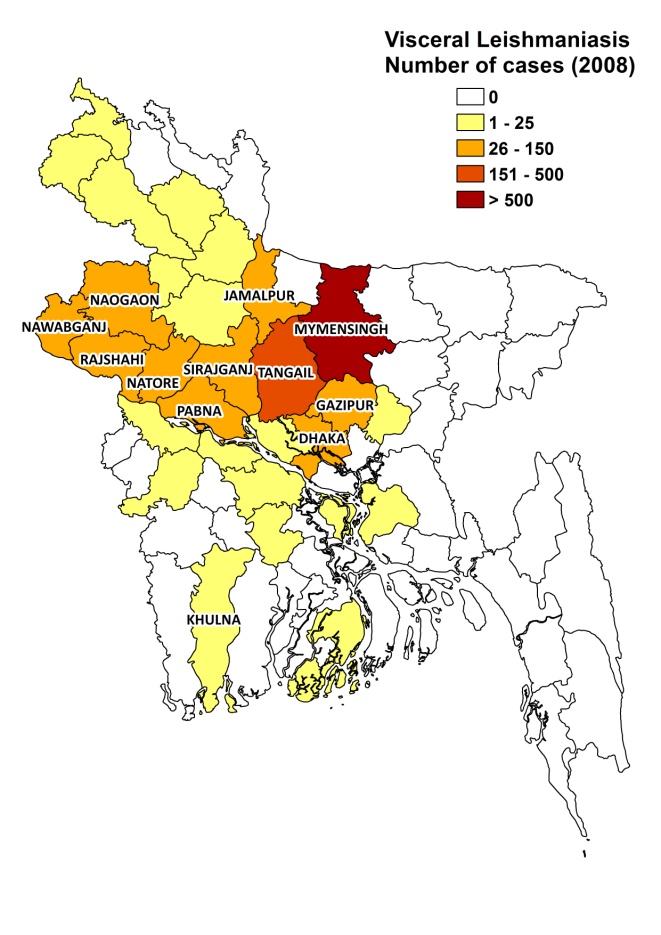


**Visceral leishmaniasis trend**

**CONTROL**

The notification of VL is mandatory. Case reporting is based on passive surveillance. An agreement was signed in 2005 to commit to a regional Kala Azar Elimination Program, which should reduce the incidence of VL at upazila level to 1/10,000 in 2015. The plan includes early diagnosis and treatment, vector control, bednet distribution, active surveillance, and social mobilization, but it has only been implemented on a limited scale due to lack of funding.

**DIAGNOSIS, TREATMENT**

**Diagnosis**

VL: rK39 antigen-based immunochromatographic test (ICT).

**Treatment**

VL: miltefosine, 2.5 mg/kg for 28 days. In case it is not available or contra-indicated: antimonials, 20 mg Sb^v^/kg/day for 30 days. Second line: conventional amphotericin B, 1 mg/kg/day for 20 days or liposomal amphotericin B 3 mg/kg/day for 5 days.

PKDL: antimonials, 20 mg Sb^v^/kg/day for 6 cycles of 20 days. Second line: conventional amphotericin B, 1 mg/kg/day for 15 days, 6 cycles with 10-day intervals between cycles or liposomal amphotericin B, 3 mg/kg/day for 5 days, six cycles with 10-day intervals between cycles.

**ACCESS TO CARE**

The national control program for leishmaniasis includes free diagnosis and treatment in public health facilities. However, access to free treatment and diagnosis is poor. A survey, conducted between 2001 and 2003, showed that 79% of patients had out-of-pocket expenses due to informal payments and additional costs such as transport. On average, they paid 7 visits to 6 different health providers before seeking care in public health facilities [9]. Due to the great distance to health centers and transport costs, 63% of patients first sought care through their local village health worker, who is unqualified to diagnose and treat leishmaniasis [9]. A survey, conducted in 2006-2007, showed that when seeking care outside the community, 52% of patients made use of the public sector, 13% used poorly trained private practitioners and 28% used local chemists in order to obtain treatment [5]. The awareness of VL is very low [5]. Generally, in communities, VL is seen as ‘any fever that cannot be cured by the local drug sellers’ (Dr Asish Kumar Das, personal communication).

The lack of access to free treatment has serious consequences. The total VL treatment cost exceeds 1.2 times the annual income. Therefore, getting loans and selling or renting out assets are standard practices [9]. A survey, held in a highly endemic village in 2000-2002, showed that the average time between onset of disease and treatment was 4 months and the case fatality rate was 6% [10]. There was gender inequality in access to treatment. Women were shown to be nearly 3 times more likely to die from VL than men and children [11].

In the past years, there have been severe shortages of anti-leishmaniasis drugs and diagnostic tests. In a survey from 2001-2003, only 14% of VL patients received a full drug course in public health facilities [9]. Consequently, many patients were treated with incomplete courses of antimonials, which may have contributed to the high levels of PKDL currently seen in Bangladesh.

**ACCESS TO DRUGS**

Severe supply gaps of antimonials led many patients to purchase incomplete treatments in the private sector. In 2008, fear of the development of resistant strains led to the decision to restrict the sale of antimonials in the private sector. However, vials of generic sodium stibogluconate (Albert David) are still available via unregulated drug markets for 12 USD/vial (leading to a treatment cost of 84 USD for a full treatment of a 35 kg patient).

Miltefosine was introduced as first line drug for VL in 2008. Unfortunately, the program distributed a counterfeit form of miltefosine that contained no active ingredient. This led to a temporary absence of treatment in Bangladesh, until WHO donated antimonials in order to continue the program. Miltefosine distribution throughout the public sector has now been resumed.

In 2008, rapid diagnostic tests were introduced in health facilities. However, supply shortages have occurred, so in order to get diagnosis many patients have to use private laboratories, where they pay around 5 USD for the test.

Sodium stibogluconate, paromomycin and miltefosine are included in the National Essential Drug List for VL. No VL drugs are registered in Bangladesh, except miltefosine. Second line drugs for VL are not available in Bangladesh, except very highly priced ones via unregulated drug markets. No amphotericin B formulations are registered in the country or included in the National Essential Drug List.

**SOURCES OF INFORMATION**

- Dr. Ahmad Raihan Sharif, Directorate General of Health Services, Ministry of Health & Family Welfare.
- Dr Asish Kumar Das and Gabriela Popescu, MSF, Bangladesh.
- Dr Mannan Bangali, WHO, Bangladesh. *WHO informal consultation on epidemiological information on disease burden due to kala-azar in Bangladesh, India and Nepal. Paro, Bhutan, 8-10 March 2011.*

1. Bern C and Chowdhury R (2006). The epidemiology of visceral leishmaniasis in Bangladesh: prospects for improved control. Indian J Med Res 2006 123:275-288.

2. [Rahman KM](http://www.ncbi.nlm.nih.gov/pubmed?term=%22Rahman%20KM%22%5BAuthor%5D), [Islam N](http://www.ncbi.nlm.nih.gov/pubmed?term=%22Islam%20N%22%5BAuthor%5D) (1983). Resurgence of visceral leishmaniasis in Bangladesh. [Bull World Health Organ.](javascript:AL_get(this,%20'jour',%20'Bull%20World%20Health%20Organ.');) 61(1):113-6.

3. Joshi A, Narain JP, Prasittisuk C, Bhatia R, Hashim G et al (2008). [Can visceral leishmaniasis be eliminated from Asia?](http://www.ncbi.nlm.nih.gov/pubmed/18592839) J Vector Borne Dis 45(2):105-11.

4. [Bern C](http://www.ncbi.nlm.nih.gov/pubmed?term=%22Bern%20C%22%5BAuthor%5D), [Haque R](http://www.ncbi.nlm.nih.gov/pubmed?term=%22Haque%20R%22%5BAuthor%5D), [Chowdhury R](http://www.ncbi.nlm.nih.gov/pubmed?term=%22Chowdhury%20R%22%5BAuthor%5D), [Ali M](http://www.ncbi.nlm.nih.gov/pubmed?term=%22Ali%20M%22%5BAuthor%5D), [Kurkjian KM](http://www.ncbi.nlm.nih.gov/pubmed?term=%22Kurkjian%20KM%22%5BAuthor%5D) et al (2007). The epidemiology of visceral leishmaniasis and asymptomatic leishmanial infection in a highly endemic Bangladeshi village. [Am J Trop Med Hyg.](javascript:AL_get(this,%20'jour',%20'Am%20J%20Trop%20Med%20Hyg.');)76(5):909-14.

5. Mondal D, Singh SP, Kumar N, Joshi A, Sundar S et al (2009). [Visceral leishmaniasis elimination programme in India, Bangladesh, and Nepal: reshaping the case finding/case management strategy.](http://www.ncbi.nlm.nih.gov/pubmed/19159009) PLoS Negl Trop Dis 3(1).

6. Bern C, Courtenay O, Alvar J (2010). Of cattle, sand flies and men: a systematic review of risk factor analyses for South Asian visceral leishmaniasis and implications for elimination.PLoS Negl Trop Dis 4(2):e355

7. [Rahman KM](http://www.ncbi.nlm.nih.gov/pubmed?term=%22Rahman%20KM%22%5BAuthor%5D), [Islam S](http://www.ncbi.nlm.nih.gov/pubmed?term=%22Islam%20S%22%5BAuthor%5D), [Rahman MW](http://www.ncbi.nlm.nih.gov/pubmed?term=%22Rahman%20MW%22%5BAuthor%5D), [Kenah E](http://www.ncbi.nlm.nih.gov/pubmed?term=%22Kenah%20E%22%5BAuthor%5D), [Ghalib CM](http://www.ncbi.nlm.nih.gov/pubmed?term=%22Ghalib%20CM%22%5BAuthor%5D) et al (2010). Increasing incidence of post-kala-azar dermal leishmaniasis in a population-based study in Bangladesh. [Clin Infect Dis.](javascript:AL_get(this,%20'jour',%20'Clin%20Infect%20Dis.');)1;50(1):73-6.

8. Mondal D, Nasrin KN, Huda MM, Kabir M, Hossain MS et al (2010). Enhanced case detection and improved diagnosis of PKDL in a Kala-azar-endemic area of Bangladesh. PLoS Negl Trop Dis 4(10):e832

9. Sharma AD, Bern C, Varghese B, Chowdhury R, Haque R et al (2006). [The economic impact of visceral leishmaniasis on households in Bangladesh.](http://www.ncbi.nlm.nih.gov/pubmed/16640630) Trop Med Int Health 11(5):757-64.

10. Ahluwalia IB, Bern C, Costa C, Akter T, Chowdhury R et al (2003). [Visceral leishmaniasis: consequences of a neglected disease in a Bangladeshi community.](http://www.ncbi.nlm.nih.gov/pubmed/14740879) Am J Trop Med Hyg 69(6):624-8.

11. Ahluwalia IB, Bern C, Wagatsuma Y, Costa C, Chowdhury R et al (2004). [Visceral leishmaniasis: consequences to women in a Bangladeshi community.](http://www.ncbi.nlm.nih.gov/pubmed/15186651) J Womens Health (Larchmt) 13(4):360-4.
