# Supplementary material for: Leishmaniasis Worldwide and Global Estimates of Its Incidence
Source: PLoS One. 2012 May 31;7(5):e35671. doi: 10.1371/journal.pone.0035671 (PMC3365071; doi:10.1371/journal.pone.0035671)
Supplement: Text S8 — Leishmaniasis Country Profiles, Belize. (DOCX) [file pone.0035671.s008.docx]

**BELIZE**

**
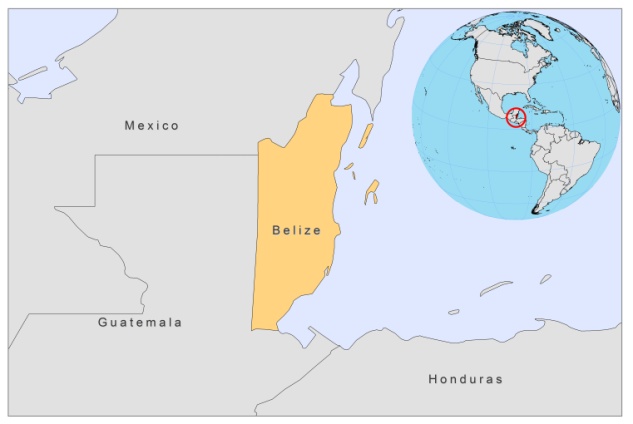
**

**BASIC COUNTRY DATA**

Total Population: 344,700

Population 0-14 years: 35%

Rural population: 47%

Population living under USD 1.25 a day: 13.4%

Population living under the national poverty line: no data

Income status: Lower middle income economy

Ranking: High human development (ranking 93)

Per capita total expenditure on health at average exchange rate (US dollar): 223

Life expectancy at birth (years): 76

Healthy life expectancy at birth (years): 60

**BACKGROUND INFORMATION**

VL has never been reported, but CL by *L.mexicana* is a well known disease in the northern area of the country [1]. In 1984, *L. braziliensis* was reported for the first time among British military personnel, infected in the southern part of Belize [2]. The infection frequency among soldiers is rising and suggests that the vector is a highly anthropophilic species, such as *Lu. ovallesi* or *Lu. Cruciata* [1].

Cutaneous leishmaniasis is an emerging health problem among travelers to Belize [3]. The nearby area in Mexico, Quintana Roo, is one of Mexico's most important endemic areas.

**PARASITOLOGICAL INFORMATION**

| ***Leishmania* species** | **Clinical form** | **Vector species** | **Reservoirs** |
| --- | --- | --- | --- |
| *L. braziliensis* | ZCL | *Lu. ovallesi* | Unknown |
| *L. mexicana* | ZCL | *Lu. olmeca olmeca* | *Heteromys sp., Nyctomys sp., Ototylomys sp., Sigmodon sp., Oryzomys sp.* |

**DIAGNOSIS, TREATMENT, MAPS AND TRENDS**

Data not available

**ACCESS TO DRUGS**

The antimonials Pentostam, GSK (SSG) and Glucantime, Sanofi (meglumine antimoniate) are not registered.

**SOURCES OF INFORMATION**

1. Desjeux P (1991). Information on the epidemiology and control of the leishmaniases by country or territory. WHO/LEISH/91.30.

2. [Evans DA](http://www.ncbi.nlm.nih.gov/pubmed?term=%22Evans%20DA%22%5BAuthor%5D), [Lanham SM](http://www.ncbi.nlm.nih.gov/pubmed?term=%22Lanham%20SM%22%5BAuthor%5D), [Baldwin CI](http://www.ncbi.nlm.nih.gov/pubmed?term=%22Baldwin%20CI%22%5BAuthor%5D), [Peters W](http://www.ncbi.nlm.nih.gov/pubmed?term=%22Peters%20W%22%5BAuthor%5D) (1984). The isolation and isoenzyme characterization of Leishmania braziliensis subsp. from patients with cutaneous leishmaniasis acquired in Belize. [Trans R Soc Trop Med Hyg.](javascript:AL_get(this,%20'jour',%20'Trans%20R%20Soc%20Trop%20Med%20Hyg.');)78(1):35-4.

3. Herwaldt BL, Stokes SL, Juranek DD (1993). American Cutaneous Leishmaniasis in U.S. Travelers. Annals of Internal Medicine 118 (10): 779-84.
