# Supplementary material for: Leishmaniasis Worldwide and Global Estimates of Its Incidence
Source: PLoS One. 2012 May 31;7(5):e35671. doi: 10.1371/journal.pone.0035671 (PMC3365071; doi:10.1371/journal.pone.0035671)
Supplement: Text S9 — Leishmaniasis Country Profiles, Bhutan. (DOCX) [file pone.0035671.s009.docx]

**BHUTAN**


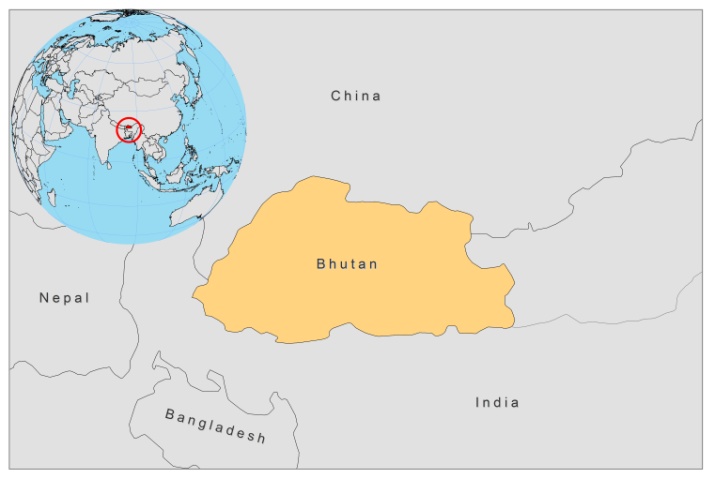


**BASIC COUNTRY DATA**

Total Population: 725,940

Population 0-14 years: 29%

Rural population: 63%

Population living under USD 1.25 a day: no data

Population living under the national poverty line: 23.2%

Income status: Lower middle income economy

Ranking: Medium human development (ranking 141)

Per capita total expenditure on health at average exchange rate (US dollar): 98

Life expectancy at birth (years): 67

Healthy life expectancy at birth (years): 53

**BACKGROUND INFORMATION**

VL occurs sporadically in 10 districts in Bhutan. A total of 22 cases was reported between 1999 and March 2011. Most (10) cases were from Mongar. In 2007, the presence of VL in Bhutan was officially documented for the first time [1]. Consequently, in 2011, WHO carried out an epidemiological survey in which *L.donovani* was confirmed to be the causative parasite. The sandflies have not yet been typed. Bhutan is in the process of developing a national strategy for leishmaniasis.

**PARASITOLOGICAL INFORMATION**

| ***Leishmania* species** | **Clinical form** | **Vector species** | **Reservoirs** |
| --- | --- | --- | --- |
| *L. donovani* | AVL | Unknown | Human |

**MAPS AND TRENDS**

**Visceral leishmaniasis**


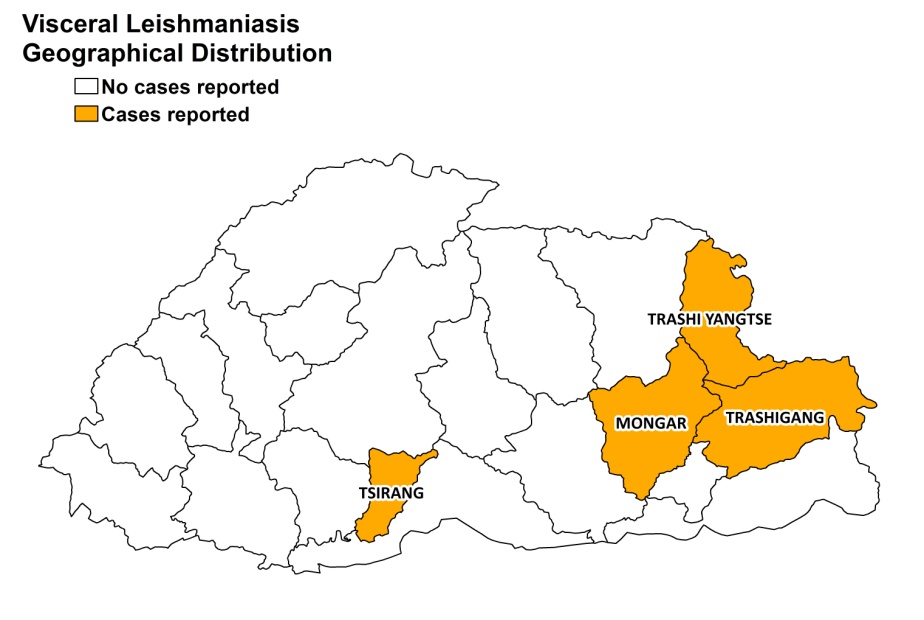


**Visceral leishmaniasis trend**

| **2005** | **2006** | **2007** | **2008** | **2009** | **2010** |
| --- | --- | --- | --- | --- | --- |
| 1 | 7 | 0 | 0 | 2 | 6 |

**CONTROL**

There are currently no control activities regarding VL in Bhutan. Vector control activities are planned for affected villages in Mongar.

**DIAGNOSIS, TREATMENT**

**Diagnosis**

VL: rK39 antigen-based immunochromatographic test (ICT), microscopic examination of spleen aspirate.

**Treatment**

VL: Antimonials, 20 mg Sb^v^/kg/day for 30 days.

**ACCESS TO CARE**

Health care is provided for free in Bhutan. All leishmaniasis patients have access to care and are treated with antimonials (provided by the government). In 2007, WHO donated miltefosine treatment and rapid diagnostic rK39 antigen-based dipsticks for diagnosis and treatment of suspected patients.

**ACCESS TO DRUGS**

No drugs for leishmaniasis are registered in Bhutan. Sodium stibogluconate is included in the National Essential Drug List.

**SOURCES OF INFORMATION**

- Dr Thinley Yangzom, Vector-borne Disease Control Programme, Gelephu, Dept. of Public Health, Ministry of Health. *A WHO informal consultation on epidemiological information on disease burden due to kala-azar in Bangladesh, India and Nepal. Paro, Bhutan, 8-10 March 2011.*

1. Bhattacharya SK, Rinzin N, Chusak P, Dash AP, Chowdhury R et al (2010). Occurrence and significance of kala-azar in Bhutan. Indian J Med Res 132: 337-338.
