# Supplementary material for: Leishmaniasis Worldwide and Global Estimates of Its Incidence
Source: PLoS One. 2012 May 31;7(5):e35671. doi: 10.1371/journal.pone.0035671 (PMC3365071; doi:10.1371/journal.pone.0035671)
Supplement: Text S10 — Leishmaniasis Country Profiles, Bolivia. (DOCX) [file pone.0035671.s010.docx]

**BOLIVIA (Plurinational State of)**


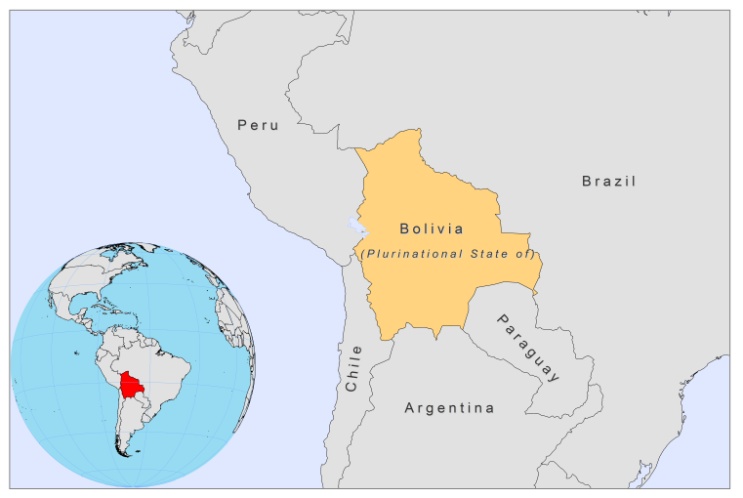


**BASIC COUNTRY DATA**

Total Population: 9,929,849

Population 0-14 years: 36%

Rural population: 34%

Population living under USD 1.25 a day: 14%

Population living under the national poverty line: 60.1

Income status: Lower middle income economy

Ranking: Medium human development (ranking 108)

Per capita total expenditure on health at average exchange rate (US dollar): 89

Life expectancy at birth (years): 66

Healthy life expectancy at birth (years): 54

**BACKGROUND INFORMATION**

Bolivia has the highest incidence of CL in Latin America, with 33 cases per 100,000 population reported in 2006 [1,2]. The considerable scale of migration from Andean to tropical areas and uncontrolled logging are two of the main reasons for the emergence of new highly active foci that are continually increasing in scale and extent. Migrants moving into endemic areas have been proposed to be more susceptible for infection [3]. Since 1983, the incidence of CL has increased from 278 cases reported in 1983 to 3,153 in 2007 and since 1997, over 2,000 cases have been reported annually. As most cases are identified through passive case detection, underreporting is suspected to be high.

In the department La Paz, the forest of the Yungas area is highly endemic for CL. The ongoing expansion of coca plantations towards the north is considered a major factor in the increase of cases. The transmission is mainly intra-domiciliary and at night time, affecting children of both sexes equally. In the Alto-Beni region, transmission occurs in sylvatic environments during the day and it is related to professional activities, like deforestation or military activities. A third epidemiological pattern happens in secondary forest like in Pando, at day time and is associated with farming, harvesting of crops, collection of rubber or Brazil nuts, etc. [1,4]. Young males are affected in approximately 80% of cases, although the number of cases among women is increasing. Out of a total number of 24,542 cases reported, the majority were among the age group 15 to 49 years old (67.3%), followed by the age group 5 to 14 years old (16.6%). Children under one year of age accounted for 1.5% of the cases.

As most CL cases are caused by *L. braziliensis*, 10-20% of all CL cases progress to mucosal leishmaniasis, more than anywhere else in the Americas [1], with the municipality of Palos Blancos in the Yungas region the most affected. 12% of dogs are seropositive in areas highly endemic for CL [5].

VL is rare and was described for the first time in an endemic focus in the Yungas region, where dogs and *Lutzomyia longipalpis* were also found infected [6,7]. In 2004, one proven case and 10 seropositive cases were detected in Taypiplaya, and in 2010, two confirmed cases were reported in Puerto Suárez, east of the country, close to the Brazilian border.

Between 1983 and 2006 a total of 35,714 cases of CL, fewer than 10 cases of VL, and 4 cases of DCL were registered nationwide. These numbers do not represent the reality as until 2008, VL, CL and ML were not reported separately.

In 2009, the first case of HIV-*Leishmania* co-infection was detected [8].

**PARASITOLOGICAL INFORMATION**

| ***Leishmania* species** | **Clinical form** | **Vector species** | **Reservoirs** |
| --- | --- | --- | --- |
| *L. braziliensis* | ZCL, MCL | *Lu. nuneztovari anglesi,*  *Lu. carrerai carrerai,*  *Lu. llanosmartinsi, Lu. shawi,*  *Lu. ayrozai, Lu. yucumensis* | unknown |
| *L. amazonensis* | ZCL, DCL | *Lu. flaviscutellata* | *Oryzomys sp.* |
| *L. infantum* | ZVL | *Lu. longipalpis* | *Canis familiaris* |
| *L. guyanensis* | ZCL | *Lu. shawi* | *Choloepus sp., Didelphis sp., Tamandua sp.* |
| *L. lainsoni* | ZCL | *Lu. nuneztovari anglesi* | *Agouti* *paca* |

**MAPS AND TRENDS**

**
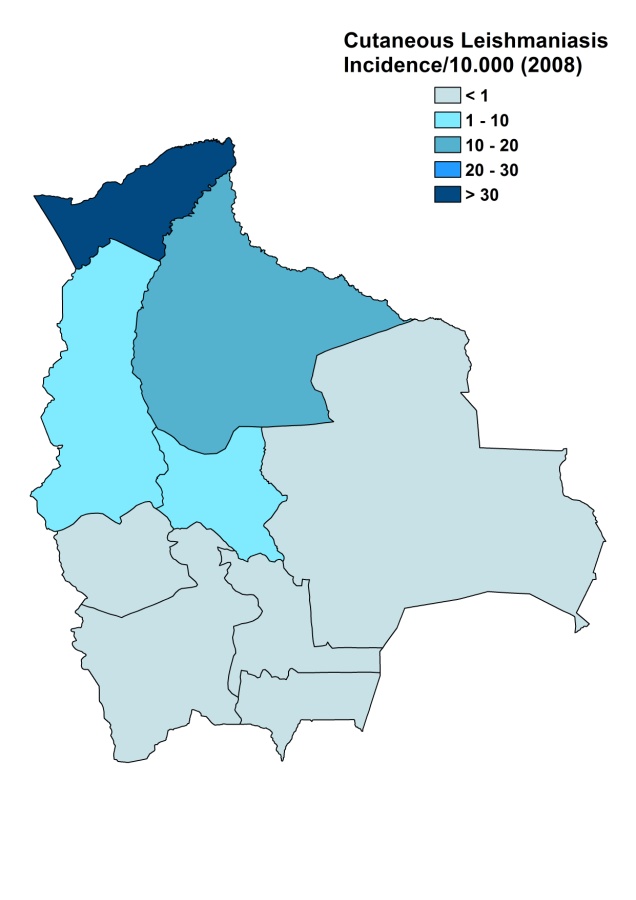
Cutaneous leishmaniasis**

**
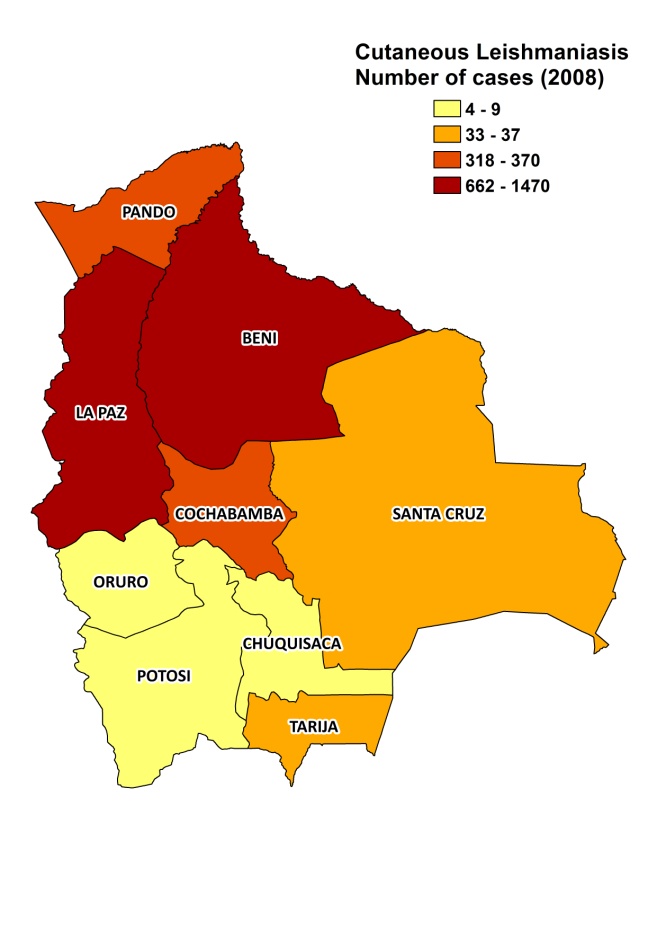
**

**Visceral leishmaniasis**

**
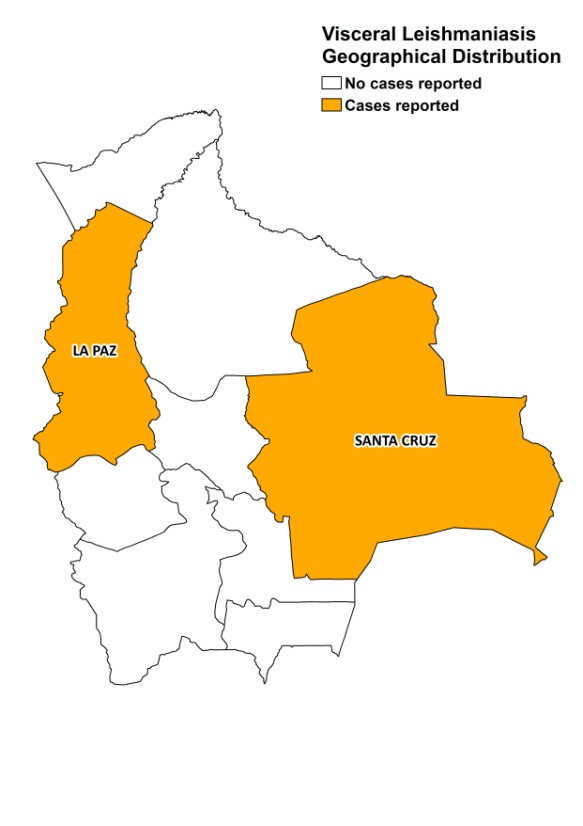
**

**Cutaneous leishmaniasis trend**

**CONTROL**

Notification of cases is mandatory and a national leishmaniasis control program is in place. Case detection is passive. There is no vector or reservoir control program.

**DIAGNOSIS, TREATMENT**

**Diagnosis**

VL and CL: parasitological confirmation by microscopy.

**Treatment**

VL: antimonials, 20 mg Sb^v^ /kg/day for 30 days. Cure rate is 100%, with a 0% fatality rate.

CL: antimonials, 20 mg Sb^v^/kg/day for 20 days. Cure rate is >70% for CL with 15% recurring lesions and <40% for MCL, with recurring lesions in 70% of patients.

Second line treatment is with conventional amphotericin B, 0.5-1 mg/kg/day, every other day for 30-45 days. Miltefosine is provided by some NGOs.

**ACCESS TO CARE**

Health care is not provided for free in Bolivia. All patients have to pay 1.5 USD for consultations and in case of MCL, 3 USD for hospitalisation. Primary diagnosis of leishmaniasis is free, but subsequent consultations are not. Health workers also commonly charge informal payments to patients. Most patients are very poor and cannot always afford the fees. Many patients use traditional herbal medicines ('evanta'). CL is diagnosed and treated at health center level with antimonials. However, there is no continuous supply of drugs and patients suffer major economic loss due to days of missed work when they spend time away from home. Also, they do not seek treatment in time due to a lack of awareness of the potentially serious nature of the disease and the lack of transport in very remote areas. There is a lack of trained human resources for treating leishmaniasis in Bolivia.

With help of the French government and USAID, the Ministry of Health was able to provide approximately sufficient antimonials and amphotericin B for the treatment of all reported patients in 2007 and 2008.

**ACCESS TO DRUGS**

Meglumine antimoniate is included in the list for VL and (M)CL, and conventional amphotericin B for (M)CL. Glucantime (Sanofi) and miltefosine are registered in Bolivia. Drugs for leishmaniasis are not available in private pharmacies or drug markets.

**SOURCES OF INFORMATION**

- Dr. Sergio Mollinedo. Leishmaniasis National Program, Ministry of Health. *Leishmaniasis en la Región de las Américas. Reunión de coordinadores de Programa Nacional de Leishmaniasis. OPS/OMS. Medellín, Colombia. 4-6 junio 2008.*

1. [García AL](http://www.ncbi.nlm.nih.gov/pubmed?term=%22Garc%C3%ADa%20AL%22%5BAuthor%5D), [Parrado R](http://www.ncbi.nlm.nih.gov/pubmed?term=%22Parrado%20R%22%5BAuthor%5D), [Rojas E](http://www.ncbi.nlm.nih.gov/pubmed?term=%22Rojas%20E%22%5BAuthor%5D), [Delgado R](http://www.ncbi.nlm.nih.gov/pubmed?term=%22Delgado%20R%22%5BAuthor%5D), [Dujardin JC](http://www.ncbi.nlm.nih.gov/pubmed?term=%22Dujardin%20JC%22%5BAuthor%5D) [et](http://www.ncbi.nlm.nih.gov/pubmed?term=%22Reithinger%20R%22%5BAuthor%5D) al (2009). Leishmaniases in Bolivia: comprehensive review and current status. [Am J Trop Med Hyg.](javascript:AL_get(this,%20'jour',%20'Am%20J%20Trop%20%0d%0aMed%20Hyg.');)80(5):704-11.

2. David C, Dimier-David L, Vargas F, Torrez M, Dedet JP (1993). Fifteen years of cutaneous and mucocutaneous leishmaniasis in Bolivia: a retrospective study. Trans R Soc Trop Med Hyg 87(1):7-9.

3. Alcais A, Abel L, David C, Torrez ME, Flandre P et al (1997). Risk factors for onset of cutaneous and mucocutaneous leishmaniasis in Bolivia. Am J Trop Med Hyg. 57(1):79-84.

4. Le Pont F, Mouchet J, Desjeux P, Torres Espejo JM, Richard A (1989). Epidemiology of cutaneous leishmaniasis in Bolivia. 2. Transmission patterns. Ann Soc Belg Med Trop 69(4):307-12.

5. Parrado R, Rojas E, Delgado R, Torrico MC, Reithinger R et al (2011). Prevalence of Leishmania spp. infection in domestic dogs in Chapare, Bolivia. Vet Parasitol 177(1-2):171-4.

6. Desjeux P, Aranda E, Aliaga O, Mollinedo S (1983). Human visceral leishmaniasis in Bolivia: first proven autochtonous case from 'Los Yungas'. Trans R Soc Trop Med Hyg 77(6):851-2.

7. Dimier-David L, Inofuentes A, Carrasco M, David C, Vargas F et al (1991). A new case of autochtonous visceral leishmaniasis in Bolivia. Ann Soc Belg Med Trop 71(4):275-278.

8. [Torrico F](http://www.ncbi.nlm.nih.gov/pubmed?term=%22Torrico%20F%22%5BAuthor%5D), [Parrado R](http://www.ncbi.nlm.nih.gov/pubmed?term=%22Parrado%20R%22%5BAuthor%5D), [Castro R](http://www.ncbi.nlm.nih.gov/pubmed?term=%22Castro%20R%22%5BAuthor%5D), [Marquez CJ](http://www.ncbi.nlm.nih.gov/pubmed?term=%22Marquez%20CJ%22%5BAuthor%5D), [Torrico MC](http://www.ncbi.nlm.nih.gov/pubmed?term=%22Torrico%20MC%22%5BAuthor%5D) et al (2009). Co-Infection of Leishmania (Viannia) braziliensis and HIV: report of a case of mucosal leishmaniasis in Cochabamba, Bolivia. [Am J Trop Med Hyg.](javascript:AL_get(this,%20'jour',%20'Am%20J%20Trop%20%0d%0aMed%20Hyg.');)81(4):555-8.
