# Supplementary material for: Leishmaniasis Worldwide and Global Estimates of Its Incidence
Source: PLoS One. 2012 May 31;7(5):e35671. doi: 10.1371/journal.pone.0035671 (PMC3365071; doi:10.1371/journal.pone.0035671)
Supplement: Text S11 — Leishmaniasis Country Profiles, Bosnia. (DOCX) [file pone.0035671.s011.docx]

**BOSNIA-HERZEGOVINA**


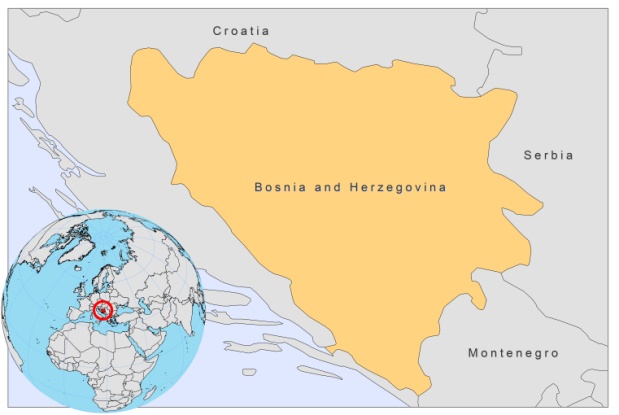


**BASIC COUNTRY DATA**

Total Population: 3,760,149

Population 0-14 years: 15%

Rural population: 51%

Population living under USD 1.25 a day: no data

Population living under the national poverty line: 14%

Income status: Upper middle income economy

Ranking: High human development (ranking 74)

Per capita total expenditure on health at average exchange rate (US dollar): 495

Life expectancy at birth (years): 75

Healthy life expectancy at birth (years): 64

**BACKGROUND INFORMATION**

VL is sporadic and hypo-endemic in south and southeast Herzegovina [1]. The first case of VL in Bosnia and Herzegovina was described in Herzegovina in 1949. Over the past ten years, the endemic area has expanded from the area southeast of Mostar to the north and into Bosnia, from Blagaj, Drežnica to the territory of Sarajevo Canton. Underreporting to a small degree is suspected due to a lack of awareness of medical personnel. 7 patients with suspected *L. infantum* infection have been hospitalized at the Clinic for Infectious Diseases of the Clinical Center in Sarajevo during the last 10 years.

In 2005, one case of CL was diagnosed.

There are no reported cases of HIV/*Leishmania* co-infection.

**PARASITOLOGICAL INFORMATION**

| ***Leishmania* species** | **Clinical form** | **Vector species** | **Reservoirs** |
| --- | --- | --- | --- |
| *L infantum* | ZVL, CL | *Unknown* | *Canis familiaris* |

**MAPS AND TRENDS**

**Visceral leishmaniasis**


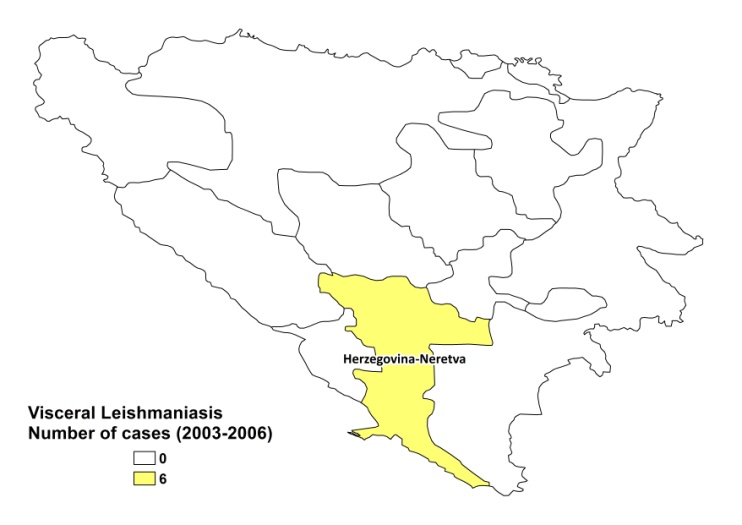

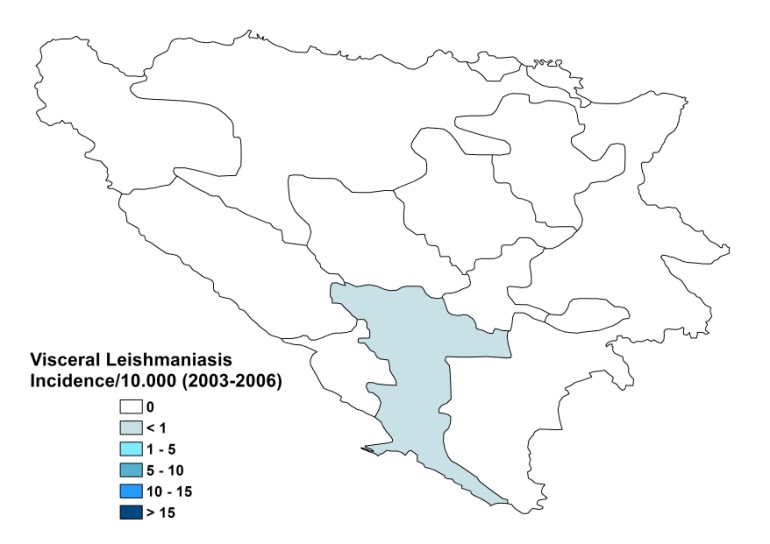


**Cutaneous leishmaniasis**

**
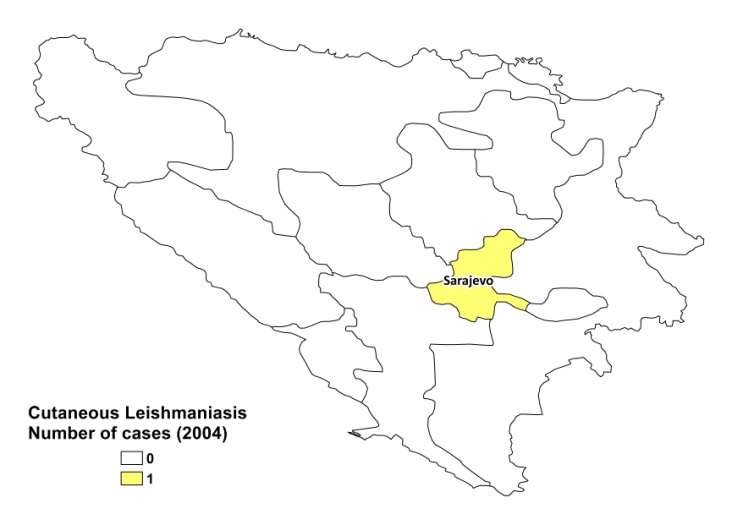
**

**Visceral and cutaneous leishmaniasis trend**

| \|  \| **2002** \| **2003** \| **2004** \| **2005** \| \| --- \| --- \| --- \| --- \| --- \| \| **VL** \| 3 \| 1 \| 1 \| 1 \| \| **CL** \|  \|  \|  \| 1 \| |  |  |
| --- | --- | --- | --- | --- | --- | --- | --- | --- | --- | --- | --- | --- | --- | --- | --- | --- | --- |

**CONTROL**

The notification of leishmaniasis is mandatory in the country. There is no national leishmaniasis control program. Case detection is passive. There is no leishmaniasis vector control program and no bednet distribution program. Insecticide spraying is not done regularly. There is no leishmaniasis reservoir control program.

**DIAGNOSIS, TREATMENT**

**Diagnosis:**

VL: microscopic examination of bone marrow, spleen or liver aspirate, cultures and serological diagnosis with ELISA and IFAT. At tertiary level, PCR is possible.

CL: confirmation with microscopic examination of skin lesion sample.

**Treatment**

VL: antimonials. Second line treatment is with conventional amphotericin B. Two patients underwent splenectomy during the last 10 years.

**ACCESS TO CARE**

Diagnosis of leishmaniasis is possible in district hospitals. Treatment is only provided in specialized hospitals. In the last years, two patients were admitted only in a very late and almost lethal stage of the disease due to a lack of awareness of medical personnel. All patients are thought to have access to care.

**ACCESS TO DRUGS**

Conventional amphotericin B is included in the National Essential Drug List. No antimonials are registered in the country. Pentamidine is the only drug for leishmaniasis available at private pharmacies.

**SOURCES OF INFORMATION**

- Dr Alija Durda, Clinic for Infectious Diseases Sarajevo. *WHO exploratory meeting on Leishmaniasis in the Balkan Countries. Dubrovnik, Croatia, 10-12 February 2010.*

1. Gvozdenovic M, Miladinovic Z (1959). [Epidemiological studies on two autochtonous cases of kala-azar in Bosnia.](http://www.ncbi.nlm.nih.gov/pubmed/13830541) Med Arh 12:95-101.
