# Supplementary material for: Leishmaniasis Worldwide and Global Estimates of Its Incidence
Source: PLoS One. 2012 May 31;7(5):e35671. doi: 10.1371/journal.pone.0035671 (PMC3365071; doi:10.1371/journal.pone.0035671)
Supplement: Text S12 — Leishmaniasis Country Profiles, Brazil. (DOCX) [file pone.0035671.s012.docx]

**BRAZIL**

**
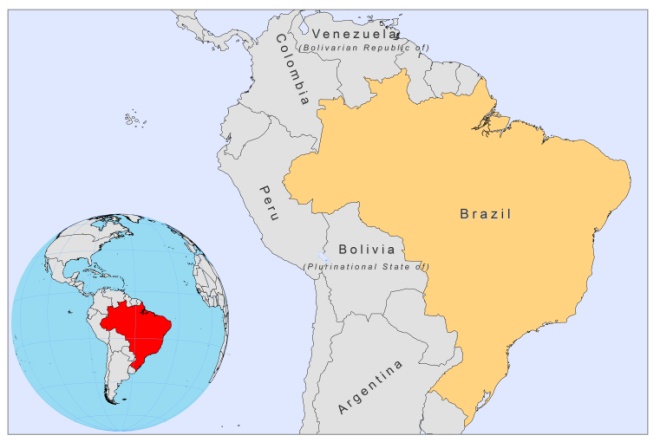
**

**BASIC COUNTRY DATA**

Total Population: 194,946,470

Population 0-14 years: 25%

Rural population: 14%

Population living under USD 1.25 a day: 3.8%

Population living under the national poverty line: 21.4%

Income status: Upper middle income economy

Ranking: High human development (ranking 84)

Per capita total expenditure on health at average exchange rate (US dollar): 734

Life expectancy at birth (years): 73

Healthy life expectancy at birth (years): 60

**BACKGROUND INFORMATION**

CL and MCL are widespread and Brazil is the country with the most CL cases in South America. Until the 1950s, most of the cases were concentrated in the states of Sao Paulo, Paraná, Minas Gerais, Ceará and Pernambuco, and associated with deforestation and new settlements. Due to the extension of these rural activities, the disease has spread to many other areas, including metropolitan Belo Horizonte [1,2]. Since the 1980s, there has been an increase in the registered number of cases, from 4,560 in 1980 to 35,748 in 1995, with frequent outbreaks [3]. From a disease typically transmitted through wild reservoirs in the forest CL has now become a periurban disease in deforested areas. Transmission peaks occur every five years, and there has been a further upward trend in the number of cases since 1985, when surveillance and control measures for CL were introduced. In the period between 1988 and 2007, 554,475 cases were notified, with an annual average of 27,723 indigenous cases and an average incidence of 17.3 cases per 100,000 inhabitants. During the 1980s, CL and MCL were registered in 19 states, and in 2003, confirmed autochtonous cases occurred in 27 states. Nevertheless, the incidence has decreased from 20.3 in 2000 to 10.5 per 10,000 population in 2008 [4]. The epidemiological risk has been categorized, by taking the average number of cases in the last three years, into areas with no transmission, sporadic transmission (≥0.1 to <2.4 cases), moderate transmission (≥2.4 to 4.4 cases), and intense transmission (≥4.4 cases). 217 municipalities have moderate or intense transmission in 2010 [4]. CL mainly affects the age group older than 10 years, which accounts for 90% of cases, and males, who represent 74% of cases.

The MCL rate is less than 5%, but varies according to regions. A higher prevalence has been correlated with areas where CL was only recently introduced and with clustered distribution [5-7].

VL represents a serious public health problem due to its scale and geographical extent. The disease is most prevalent in the north-eastern part of the country [8]. In Piauí state, VL was endemic in rural areas, but at the end of the 1970s, urban transmission started in the capital, Teresinam, with more than 1,000 cases in the period from 1981 to 1986. Underlying reasons were the population movement from rural to urban areas, poor housing, uncontrolled deforestation, and an increasing number of infected dogs [9,10]. Between 1998 and 2010, a total of 32,459 cases of VL were registered, among 21 states. The average annual number of cases was 3,246, with an incidence rate of approximately 1,85 cases per 100,000 inhabitants, and has a tendency to increase [11]. Areas in which VL is transmitted are classified according to the epidemiological risk, using the average number of cases over the last five years [11]: sporadic transmission (<2.4 cases), moderate transmission (≥ 2.4 and < 4.4 cases) and intense transmission (≥ 4.4 cases). Priority to control measures is given to areas in which transmission is moderate and intense. In recent years, the number of deaths and the case fatality rate related to VL have gradually increased, from 117 deaths (3.4%) in 1994, to 262 (7.2%) in 2006, representing an increase in case fatality rate of almost 124% [4]. In 2007, 52.8% of confirmed cases of VL affected children younger than 10 years of age, the majority being male (61.8%) and risk factors, such as concomitant malnutrition or infectious diseases, seem very important with respect to future clinical evolution [4]. Canine visceral leishmaniasis is widespread, with up to 20% of dogs infected in the highly endemic localities. The culling of infected dogs, for the control of VL, is a matter of intense debate [12].

Cases of VL-HIV co-infection have increased over the years. In 2006, 2.5% of cases of VL were coinfected [13]. An active surveillance system and guidelines for co-infected cases management have been established by the Ministry of Health [14].

**PARASITOLOGICAL INFORMATION**

| ***Leishmania* species** | **Clinical**  **form** | **Vector species** | **Reservoirs** |
| --- | --- | --- | --- |
| *L. guyanensis* | ZCL | *Lu. umbratilis,*  *Lu. anduzei,*  *Lu. whitmani* | *Choloepus sp., Tamandua sp., Didelphis sp., Proechimys sp.* |
| *L. amazonensis* | CL | *Lu. flaviscutellata,*  *Lu. longipalpis* | *Proechimys sp., Oryzomys sp., Wiedomys sp.* |
| *L. braziliensis* | ZCL, MCL | *Lu. whitmani,*  *Lu. intermedia,*  *Lu. wellcomei,*  *Lu. complexa, Lu. neivai,*  *Lu. edwardsi, Lu. migonei* | *Canis familiaris, Rattus rattus, Akodon arviculoides, Bolomys sp., Nectomis sp., Thrichomys sp.,* |
| *L. infantum* | ZVL | *Lu. longipalpis, Lu. cruzi,*  *Lu. almerio, Lu. salesi* | *Canis familiaris, Lycalopex vetulus, Cedocyon thous, Didelphis albiventris* |
| *L. lainsoni* | ZCL | *Lu. ubiquitalis* | *Agouti paca* |
| *L. shawi* | ZCL | *Lu whitmani* | *Cebus apella, Chiropotes satanus, Nasua nasua, Bradypus tridactylus, Choloepus didactylus* |
| *L. naiffi* | ZCL | *Lu. squamiventris, Lu. paraensis, Lu. amazonensis, Lu.ayrozai* | *Dasypus novemcinctus* |
| *L. lindenbergi* | ZCL | unknown | unknown |

**MAPS AND TRENDS**

**Visceral leishmaniasis**


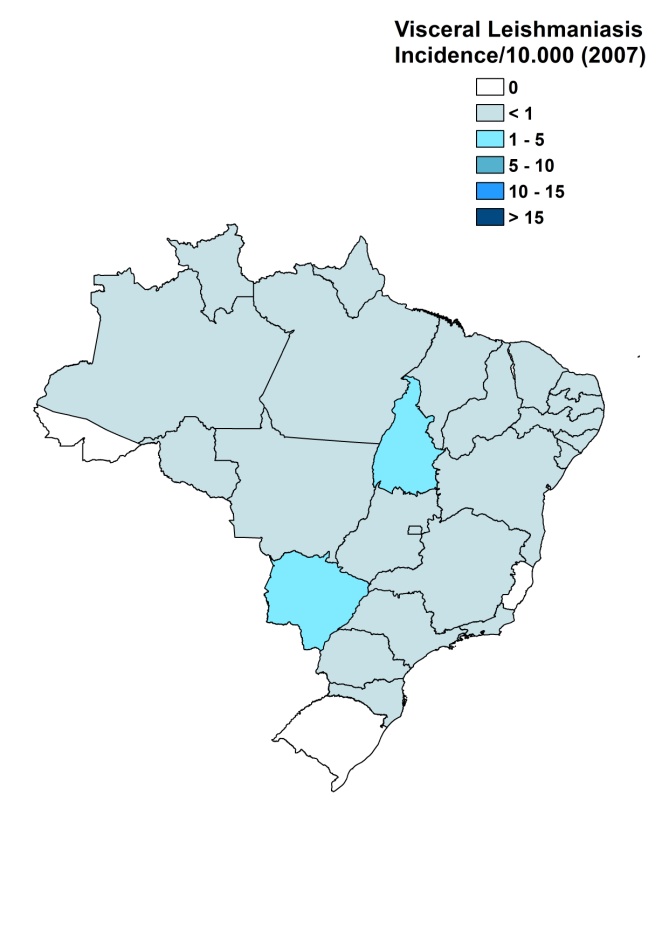

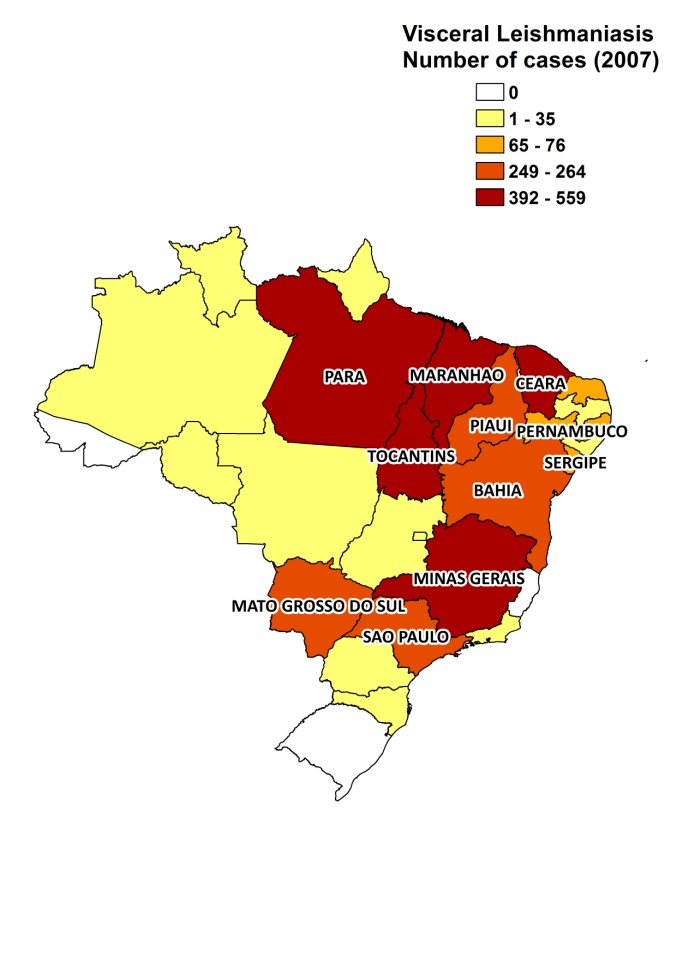


**Cutaneous leishmaniasis**

**
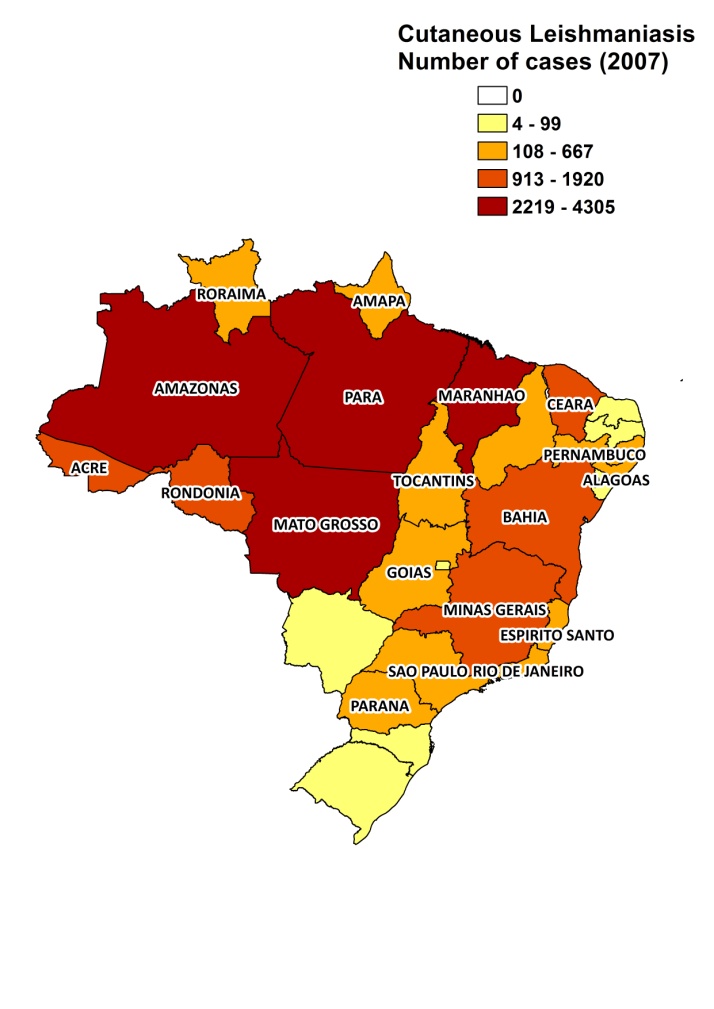

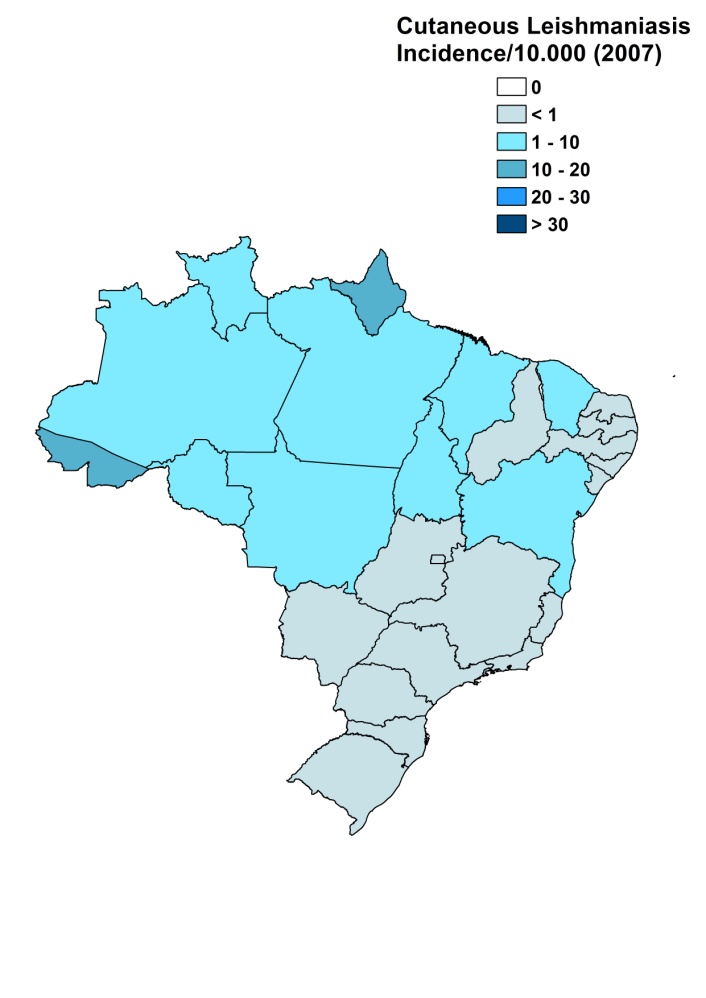
**

**Visceral leishmaniasis trend**

**Cutaneous leishmaniasis trend**

**Mucocutaneous leishmaniasis trend**

**Mortality trend**

**CONTROL**

Notification of leishmaniasis is mandatory in Brazil. A national leishmaniasis control program has been in effect since 1985 for VL and (M)CL. Strategies to control VL focus on early diagnosis and appropriate treatment for human cases, supervision and monitoring of the canine population and the elimination of dogs that test seropositive or positive for the parasite. Strategies also entail entomological surveillance, environmental health and chemical control measures, using residual insecticide, and finally preventive measures targeting humans, the vector and dogs. CL control strategies are specific, depending on the epidemiological characteristics of each site and region; the basic requirements are determination of the maximum number of possible suspect cases, early diagnosis and treatment of confirmed cases, identification of the causal agent in circulation and of the predominant vector, knowledge of the areas of transmission, and reduction of the number of human-vector contacts by means of specific individual and collective measures.

**DIAGNOSIS, TREATMENT**

**Diagnosis**

VL: parasitological and serological (IFA and rK39 antigen-based rapid test, the latter being under implementation).

CL: parasitological, the Montenegro Leishmanin Skin test and with PCR.

**Treatment**

VL: antimonials, 20 mg Sb^v^/kg/day for 30 days. Second line treatment is with amphotericin B (liposomal and conventional). The cure rate with antimonials for VL is 83%.

CL: antimonials, 20 mg Sb^v^/kg/day for 20 days.

**ACCESS TO CARE**

Care for leishmaniasis is provided for free. The government purchased sufficient quantities of liposomal amphotericin B (AmBisome, Gilead), meglumine antimoniate (Glucantime, Aventis), pentamidine and conventional amphotericin B for the treatment of leishmaniasis in 2007 and 2008. It is not known how many people use the private sector for treatment, but it is believed that all patients have access to treatment in the public sector. The level of health care at which diagnosis and treatment occur depends on the situation. Patients treated with antimonials, especially for the cutaneous forms, are seen at the primary level. Treatment with other drugs requires hospitalization (secondary or tertiary level). Diagnosis of VL takes place only at hospital level.

**ACCESS TO DRUGS**

Meglumine antimoniate and amphotericin B (lipid formulations and conventional) are included in the National Essential Drug List for VL and CL. Pentamidine is also included for CL. Glucantime (Aventis) and AmBisome (Gilead) are registered in Brazil. Amphotericin B (AmBisome and conventional) are available at private pharmacies.

**SOURCES OF INFORMATION**

- Drs Ana Nilce Silveira Maia Elkhoury and Marcia L de Sousa Gomes. Leishmaniasis National Program. Ministry of Health. *Leishmaniasis en la Región de las Américas. Reunión de coordinadores de Programa Nacional de Leishmaniasis. OPS/OMS. Medellín, Colombia. 4-6 junio 2008.*

1. Passos VM, Falcão AL, Marzochi MC, Gontijo CM, Dias ES et al (1993). [Epidemiological aspects of American cutaneous leishmaniasis in a periurban area of the metropolitan region of Belo Horizonte, Minas Gerais, Brazil.](http://www.ncbi.nlm.nih.gov/pubmed/8246744) Mem Inst Oswaldo Cruz 88(1):103-10.

2. Brandão-Filho SP, Campbell-Lendrum DH, Brito MEF, Shaw JJ, Davies CR (1999). Epidemiological surveys confirm an increasing burden of cutaneous leishmaniasis in north-east Brazil. Trans R Soc Trop Med Hyg 93:488-494.

3. Manual de Vigilância da Leishmaniose Tegumentar Americana / Ministério da Saúde, Secretaria de Vigilância em Saúde, Departamento de Vigilância Epidemiológica. – 2. ed. – Brasília : Editora do Ministério da Saúde, (Série A. Normas e Manuais Técnicos), 2007. 182 p.

4. Ministério da Saúde, Secretaria de Vigilância em Saúde, Boletin Epidemiológico, 2010; 2:11-13.

5. Marsden PD (1986). Mucosal leishmaniasis ("espundia" Escomel, 1911). Trans R Soc Trop Med Hyg 80:859.

6. Bedoya-Pacheco SJ, Araujo-Melo MH, Valete-Rosalino CM, Pimentel MI, Conceição-Silva F et al (2011). [Endemic Tegumentary Leishmaniasis in Brazil: Correlation between Level of Endemicity and Number of Cases of Mucosal Disease.](http://www.ncbi.nlm.nih.gov/pubmed/21633026) Am J Trop Med Hyg 84(6):901-5.

7. Castellucci L, Cheng LH, Araújo C, Guimarães LH, Lessa H et al (2005). [Familial aggregation of mucosal leishmaniasis in northeast Brazil.](http://www.ncbi.nlm.nih.gov/pubmed/16014836) Am J Trop Med Hyg 73(1):69-73.

8. Azevedo ACR, Souza NA, Meneses CRV, Costa WAC, Costa SM et al (2002). Ecology of sand flies (Diptera: Psychodidae: Phlebotominae) in the north of the state of Mato Grosso, Brazil. Mem Inst Oswaldo Cruz 97:459-64.

9. Costa CHN, Pereira HF, Araújo MV (1990). Epidemia de leishmaniose visceral no Estado do Piauí, Brasil, 1980-1986. Rev Saúde Pública 24:361-72.

10. Werneck GL, Costa CHN, Walker AM, David JR, Wand M et al (2002). The urban spread of visceral leishmaniasis: clues from spatial analysis. Epidemiology 13:364-7.

11. Maia-Elkhoury AN, Alves WA, Sousa-Gomes ML, Sena JM, Luna EA (2008). [Visceral leishmaniasis in Brazil: trends and challenges.](http://www.ncbi.nlm.nih.gov/pubmed/19082286) Cad Saude Publica 24(12):2941-7.

12. Dantas-Torres F, Brandão-Filho SP (2006). Visceral leishmaniasis in Brazil: revisiting paradigm of epidemiology and control. Rev. Inst. Med. Trop. S. Paulo **48:** 151–156.

13. Maia-Elkhouri ANS, Lucena F, Sousa-Gomes ML, Alves WA, Paz L (2007). Co-infecção da leishmaniose visceral e AIDS no Brasil. Rev Soc Bras Med Trop 40 Suppl 1:124.

14. Ministério da Saúde/Secretaria de Vigilância em Saúde 2004. Manual de recomendações para diagnóstico, tratamento e acompanhamento da co-infecção *Leishmania*-HIV, Brasília. Programa Nacional de DST/HIV/Aids. Available from:

[portal.saude.gov.br/portal/arquivos/pdf/manual_leish_hiv.pdf](http://portal.saude.gov.br/portal/arquivos/pdf/manual_leish_hiv.pdf).
