# Supplementary material for: Leishmaniasis Worldwide and Global Estimates of Its Incidence
Source: PLoS One. 2012 May 31;7(5):e35671. doi: 10.1371/journal.pone.0035671 (PMC3365071; doi:10.1371/journal.pone.0035671)
Supplement: Text S13 — Leishmaniasis Country Profiles, Bulgaria. (DOCX) [file pone.0035671.s013.docx]

**BULGARIA**


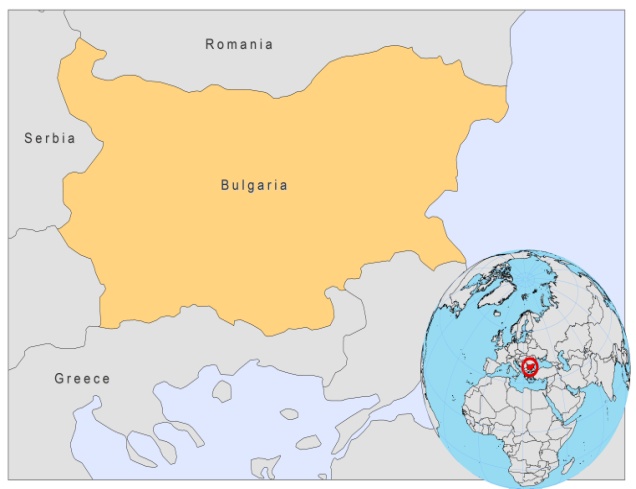


**BASIC COUNTRY DATA**

Total Population: 7,543,325

Population 0-14 years: 14%

Rural population: 28%

Population living under USD 1.25 a day: 1%

Population living under the national poverty line: no data

Income status: Upper middle income economy

Ranking: High human development (ranking 55)

Per capita total expenditure on health at average exchange rate (US dollar): 413

Life expectancy at birth (years): 73

Healthy life expectancy at birth (years): 65

**BACKGROUND INFORMATION**

The first imported case of VL in Bulgaria was described in 1921 and the first autochtonous case of the disease was observed in 1932 [1]. From 1937 to 1953, 57 autochthonous cases of VL were reported, 50 of which (88%) were in children. No data are available on VL between 1953 and 1988. 110 patients with VL have been registered from 1988 until 2008, 53% of which were children. It is now specific to Bulgaria that cases occur at all ages and there is no predominance of VL among children. 92 patients were from southern Bulgaria, the Thrakian Lowlands and the valley of Struma river. However, currently, the most highly endemic region is the municipality of Petrich; most cases observed during the last years are from this region (16 cases between 2004 and 2008).

The first investigations of canine VL were performed in southwestern Bulgaria in 1941. More recently, serology examinations of 172 dogs at the southern border found a seropositivity rate of 10%, but no active infections. Two infected dogs were found for the first time in 2006 [2].

In 2002, the first case of visceral *Leishmania*/HIV co-infection was reported [3].

**PARASITOLOGICAL INFORMATION**

| ***Leishmania* species** | **Clinical form** | **Vector species** | **Reservoirs** |
| --- | --- | --- | --- |
| *L. infantum* | ZVL, CL | Unknown | *Canis familiaris* |

**MAPS AND TRENDS**

**Visceral leishmaniasis**


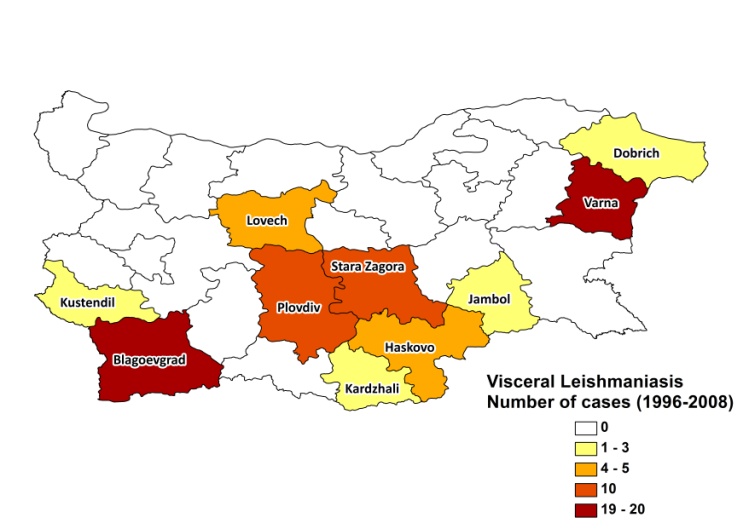

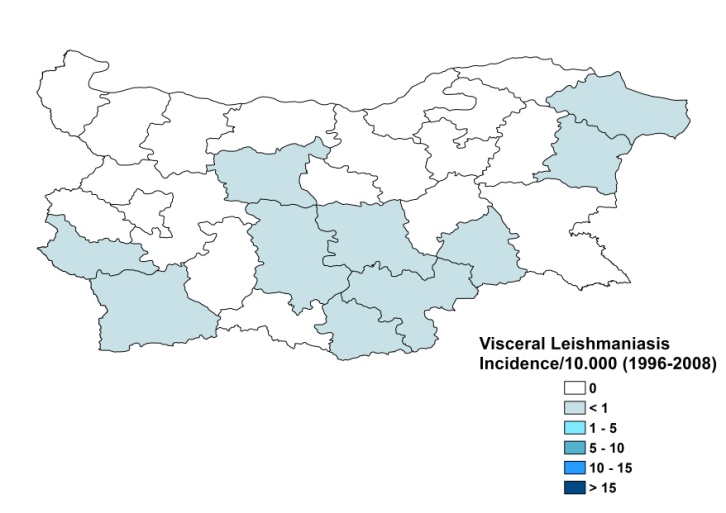


**Visceral leishmaniasis trend**

**CONTROL**

The notification of leishmaniasis is mandatory in the country. There is no national leishmaniasis control program. Active human case detection is not regularly performed. There is no leishmaniasis vector control program and no bednet distribution program. There is a leishmaniasis reservoir control program. Serological surveys of dogs are not regularly performed and positive dogs are not sacrificed.

**DIAGNOSIS, TREATMENT**

**Diagnosis**

VL: confirmation by microscopic examination of bone marrow aspirate or ELISA.

**Treatment**

VL: antimonials (Glucantime) 20 mg Sb^v^/kg/day for 20 - 28 days.

**ACCESS TO CARE**

Care for leishmaniasis is provided for free in Bulgaria, but patients have to pay for the drugs (meglumine antimoniate (Glucantime, Sanofi) themselves as these are not provided by the government. Cases can only be diagnosed and treated in one specialized hospital. The drugs are unaffordable for poor patients. They seek substandard private care or care by traditional healers before reporting to the health facilities.

From 1988 to 2008, 17 (16%) patients died from VL due to late diagnosis and treatment, and due to a lack of awareness of the disease. The period between disease and diagnosis of VL varied between 1 month and a year.

**ACCESS TO DRUGS**

There are no officially registered drugs for leishmaniasis in Bulgaria and none are included in the National Essential Drug List. They are not available at pharmacies or drug markets. No antimonials are registered in Bulgaria.

**SOURCES OF INFORMATION**

- Drs Tatiana Tcherveniakova and Nina Yancheva, Specialized Hospital for Infectious and Parasitic Diseases, Sofia. *A WHO exploratory meeting on Leishmaniasis in the Balkan Countries. Dubrovnik, Croatia, 10-12 Feruary 2010.*

1. Chakarova B, Tsachev I, Filipov G Filipova V, Chakarov S et al (2005). New cases of leishmaniasis visceralis in southeast Bulgaria. Trakia Journal of Sciences 3 (4) : 75-77.

2. Tsatchev I, Kyriazis ID, Boutsini S, Karagouni E, Dotsika E (2010). First report of canine visceral leishmaniasis in Bulgaria, Turk J Vet Anim Sci 34(3): 465-469.

3. Valkova I, Kurdova R, Elenkov I, Jordanova D, Filippov G et al (2004). International Conference on AIDS (15th : 2004 : Bangkok, Thailand). Int Conf AIDS. 2004 Jul 11-16; 15: abstract no. B11340.
