# Supplementary material for: Leishmaniasis Worldwide and Global Estimates of Its Incidence
Source: PLoS One. 2012 May 31;7(5):e35671. doi: 10.1371/journal.pone.0035671 (PMC3365071; doi:10.1371/journal.pone.0035671)
Supplement: Text S14 — Leishmaniasis Country Profiles, Burkina Faso. (DOCX) [file pone.0035671.s014.docx]

**BURKINA FASO**


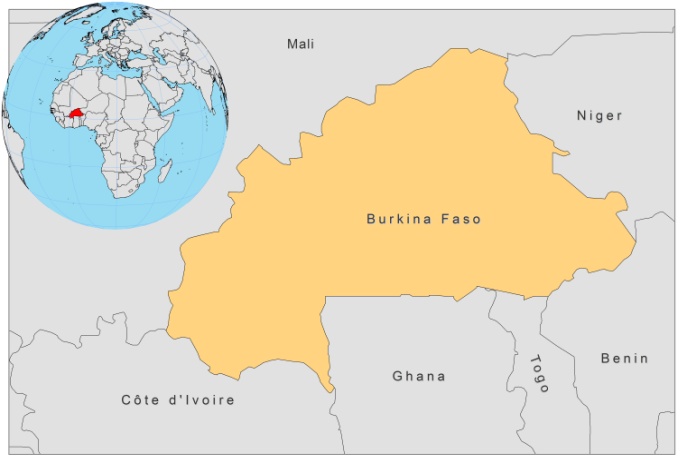


**BASIC COUNTRY DATA**

Total Population: 16,468,714

Population 0-14 years: 45%

Rural population: 80%

Population living under USD 1.25 a day: no data

Population living under the national poverty line: no data

Income status: Low income economy

Ranking: Low human development (ranking 181)

Per capita total expenditure on health at average exchange rate (US dollar): 38

Life expectancy at birth (years): 54

Healthy life expectancy at birth (years): 36

**BACKGROUND INFORMATION**

Very few data on prevalence or incidence are available. Burkina Faso is a well known focus of cutaneous leishmaniasis caused by *L. major*. The first documented cases were reported in 1962 [1]. Sporadic cases were reported from many localities between 1953 and 1984, but the number of cases has increased since 1996. The foci are in the northwest and in the south and have a low endemicity; in the eastern areas, only a few cases were diagnosed. The incidence of CL has increased in the town of Ouagadoudou in 1996. Between 1996 and 1998, 1,845 cases were reported, 50% of which were men [2]. The major increase in the number of cases started in 2000, following new developments near Ouagadoudou. The disease is now known as 'Ouaga 2000'. Cases occur in all age groups and in equal rates for men and women.

In a survey, held between September and November 2000, 10 out of 74 CL patients were coinfected with HIV (14.3%) [3]. HIV/*Leishmania* (CL) co-infection has been reported regularly since then.

Many unusual clinical features were found in coinfected patients, including lepromateuse and diffuse, ulcerative, infiltrative, papulo-nodular, psoriasis-like, Kaposi-like, cheloid, and histioid forms [3]. In 2008, a *L.major*/HIV co-infection was reported in a patient from Burkina Faso with diffuse cutaneous involvement and where *L. major* spread to bone marrow [4].

Only a few cases of VL have been reported. The first case was described in 1971 [5]. In 1978, another case was described [6].

**PARASITOLOGICAL INFORMATION**

| ***Leishmania* species** | **Clinical form** | **Vector species** | **Reservoirs** |
| --- | --- | --- | --- |
| *L. major* | ZCL | *P. duboscqi,*  *P. bergeroti* | Unknown |

**MAPS AND TRENDS**

**Cutaneous leishmaniasis**

**
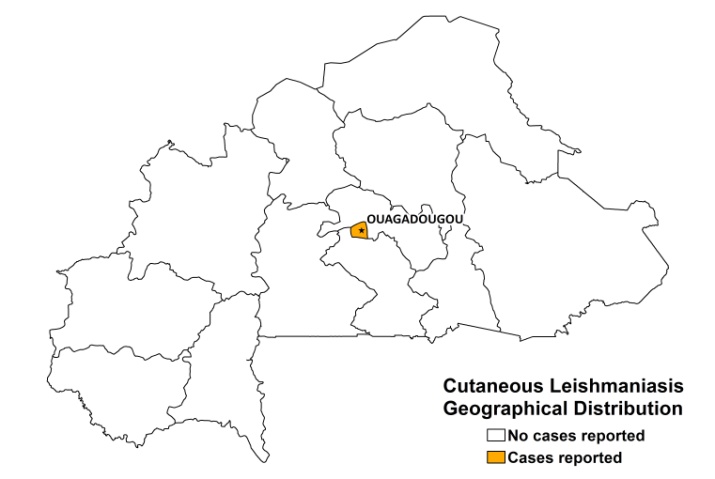
**

**Cutaneous leishmaniasis trend**

**CONTROL**

No information available

**DIAGNOSIS, TREATMENT**

**Diagnosis**

CL: On clinical grounds, confirmation by microscopic examination of tissue sample.

**Treatment**

CL: Antimonials, intralesional or systemic, 20 mg Sb^v^/kg/day for 21 days. Cure rate of systemic therapy in coinfected patients is 50%. Relapses occur regularly. The cure rate increases to 75% with three treatment cycles [7].

**ACCESS TO CARE**

No information.

**ACCESS TO DRUGS**

No antimonials are registered in Burkina Faso. Meglumine antimoniate (Glucantime, Sanofi) is used in hospitals.

**SOURCES OF INFORMATION**

- Dr Robert T. Guiguemdé. Université Polytechnique de Bobo-Dioulasso, Bobo-Dioulasso, Burkina Faso. *Fifth WHO Consultative Meeting on* Leishmania*/HIV Coinfection, Addis Ababa, Ethiopia, 20–22 March 2007.*

1. Oddou A (1962). Sur treize cas de leishmaniose cutanee en Haute-Volta. Bulletin de la Societe de Medecine d’Afrique Noire 7: 284-287.

2. Traoré KS, Sawadogo AS, Traoré A, Ouedraogo JB, Traoré TR et al (2001). Étude preliminaire de la leishmaniose cutanée dans la ville de Ouagadougou de 1996–1998. Bull Soc Path Exot 94:52–55.

3. Guiguemdé RT, Sawadogo OS, Bories, Traore KL, Nezien D et al (2003). Leishmania major and HIV co-infection in Burkina Faso. Trans Roy Soc of Trop Med Hyg 97 (2): 168-169.

4. Barro-Traoré F, Preney L, Traoré A, Darie H, Tapsoba P et al (2008). Cutaneous leishmaniasis due to Leishmania major involving the bone marrow in an AIDS patient in Burkina Faso. Ann Dermatol Venereol 135(5):380-3.

5. Desjeux P (1991) Information on the epidemiology and control of the leishmaniases by country or territory. World Health Organization. WHO/LEISH/91.30.

6. Andre LJ, Sirol J, Le Vourch C, Lebegorre J and Cochevelou D (1978). Sudanese kala-azar in West Africa. Med Trop 38(4): 435-442.

7. Alvar J, Aparicio P, Aseffa A, Den Boer M, Cañavate C et al (2008). The Relationship between Leishmaniasis and AIDS: the Second 10 Years. Clin Microb Rev 21 (2): 334–359.
