# Supplementary material for: Leishmaniasis Worldwide and Global Estimates of Its Incidence
Source: PLoS One. 2012 May 31;7(5):e35671. doi: 10.1371/journal.pone.0035671 (PMC3365071; doi:10.1371/journal.pone.0035671)
Supplement: Text S15 — Leishmaniasis Country Profiles, Cameroon. (DOCX) [file pone.0035671.s015.docx]

**CAMEROON**


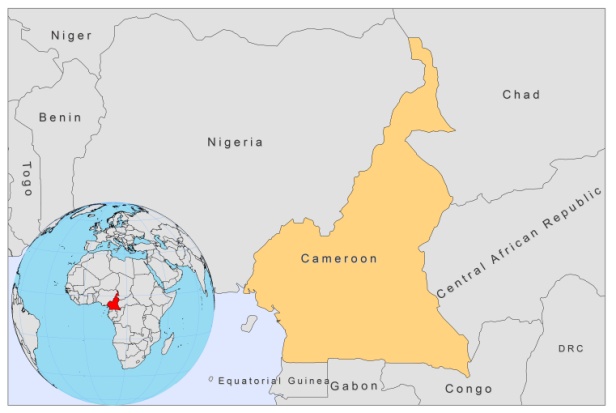


**BASIC COUNTRY DATA**

Total Population: 19,598,889

Population 0-14 years: 41%

Rural population: 42%

Population living under USD 1.25 a day: 9.6%

Population living under the national poverty line: 39.9%

Income status: Lower middle income economy

Ranking: Low human development (ranking 150)

Per capita total expenditure on health at average exchange rate (US dollar): 61

Life expectancy at birth (years): 51

Healthy life expectancy at birth (years): 41

**BACKGROUND INFORMATION**

CL was first described in Cameroon in 1930 [1]. 326 cases were reported in the town of Garoua, in the north of Cameroon; the majority of cases, however, were migrants from the forest zone in the south of the country. 108 further cases were reported in 1950-1958. In 1972, the first case was reported from Mokolo, in the far north of the country. A further 58 cases were found in this region during a survey held in 1974-1975 [2]. Between 1976 and 1994, no data on leishmaniasis were reported.

In 1994 in a survey sample of 887 from the Mokolo region, 162 people were found with leishmanian scars and 14 cases with active leishmaniasis. In a larger survey held among 6,503 people from 1996 to 1997, 82 active cases and 40 persons with scars were identified. Mokolo is now thought to be the main endemic focus for CL in the country. 147 cases of CL were reported within the period January 2007 to June 2009, 60% of which were younger than 15 years old and 52% were male. Underreporting is thought to be substantial [3].

Very little is known about VL in Cameroon. VL may be endemic in Kousseri region in the north of Cameroon. The first time a suspected case was found was in 1976. The first confirmed case was a child from the far north of the country, in 1986. Between 1987 and 1988, in a survey among 120 persons in Kousseri, 46 had clinical symptoms of VL and 9 of them were confirmed parasitologically and/or serologically [4].

There are no reported cases of HIV-*Leishmania* co-infection.

**PARASITOLOGICAL INFORMATION**

| ***Leishmania* species** | **Clinical form** | **Vector species** | **Reservoirs** |
| --- | --- | --- | --- |
| *L. major* | ZCL | *P. duboscqi* | Unknown |

**MAPS AND TRENDS**

**Cutaneous leishmaniasis**


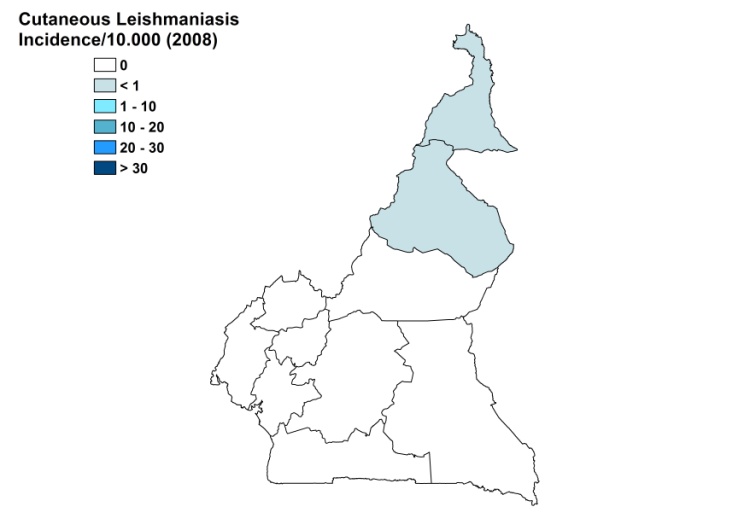

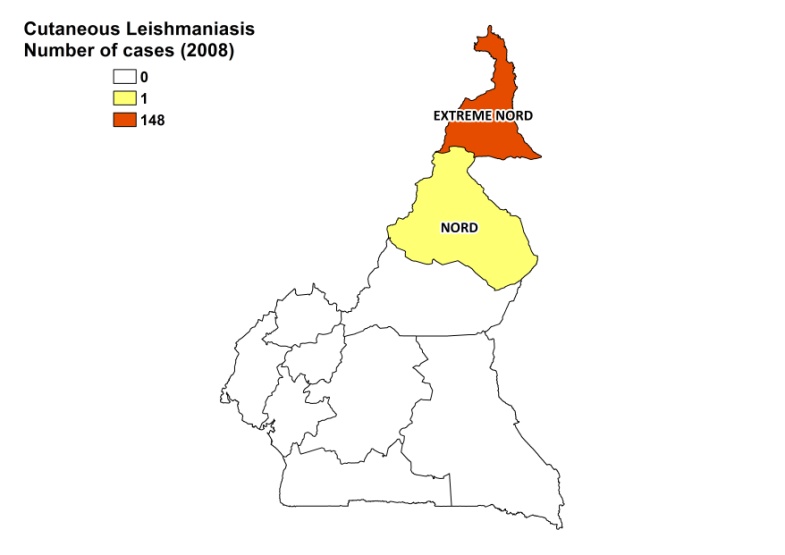


**Visceral leishmaniasis**

**
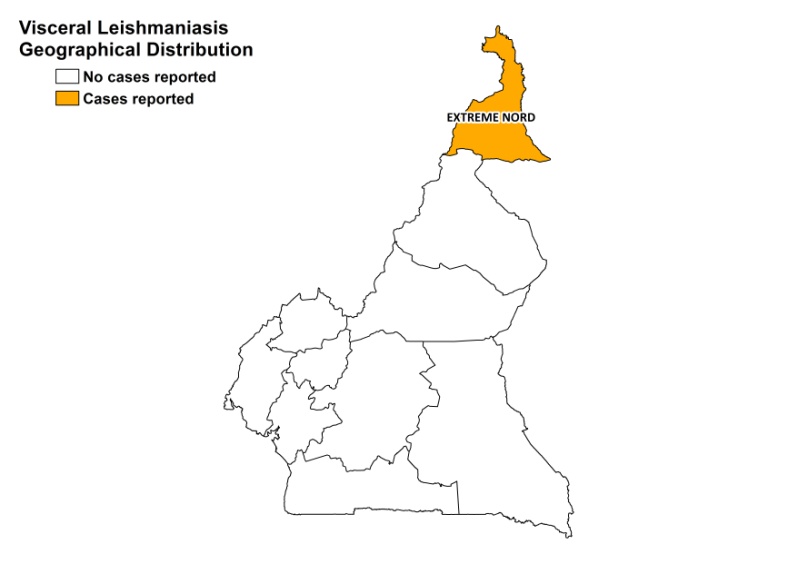
**

**Cutaneous leishmaniasis trend**

No reports, only data from surveys

**CONTROL**

In June 2009, the Minister of Public Health reorganized the National Committee for Leprosy and Buruli ulcer Control (CNLP2LUB) to include yaws and leishmaniasis control. It is expected to be operational in 2010. The notification of leishmaniasis is not mandatory in the country. Case detection is passive. There is no leishmaniasis vector control program and no leishmaniasis reservoir control program.

**DIAGNOSIS, TREATMENT**

**Diagnosis**

CL: on clinical grounds, and confirmed parasitologically or serologically.

**Treatment**

CL: a combination of allopurinol and metronidazole, and sometimes topical amphotericine B (if available). In some cases, these drugs were not effective. The response rate has not been recorded.

**ACCESS TO CARE**

Care for leishmaniasis is not provided for free. Patients have to pay for registration, consultation (1.5 USD), drugs (about 20 USD) and lab tests (3 USD). The leishmaniasis control program has not been implemented yet due to a lack of funding. This means that there are no drugs for leishmaniasis at health facilities and hospitals and that there is a lack of trained human resources for diagnosis and treatment of leishmaniasis. Patients are often too poor to pay for the drugs themselves.

The private sector is not used. CL patients are diagnosed and treated in some of Moloko’s health centers or at the Moloko district hospital. Some patients are treated by ALES (Aide aux Lépreux Emmaüs-Suisse). VL is only diagnosed and treated in the University teaching hospital.

**ACCESS TO DRUGS**

Metronidazole is included in the National Essential Drug List. No drugs for leishmaniasis are included. Metronidazole and allopurinol are available in pharmacies and unregulated drug markets. No drugs for leishmaniasis are registered.

**SOURCES OF INFORMATION**

- Dr. Ernest Njih Tabah, Ministry of Health. *Consultative Meeting on The Control of Leishmaniasis in the African Region WHO/AFRO Addis Ababa, 23-25 Feb 2010.*

1. Hervé (1937). Note sur la leishmaniose cutanée au Cameroun. Ann. Méd. Pharm. Colon. 35: 928-934.

2. [Djibrilla Kaou B](http://www.ncbi.nlm.nih.gov/pubmed?term=%22Djibrilla%20Kaou%20B%22%5BAuthor%5D), [Ripert C](http://www.ncbi.nlm.nih.gov/pubmed?term=%22Ripert%20C%22%5BAuthor%5D), [Ravisse P](http://www.ncbi.nlm.nih.gov/pubmed?term=%22Ravisse%20P%22%5BAuthor%5D), [Durand B](http://www.ncbi.nlm.nih.gov/pubmed?term=%22Durand%20B%22%5BAuthor%5D), [Carrie J](http://www.ncbi.nlm.nih.gov/pubmed?term=%22Carrie%20J%22%5BAuthor%5D) (1979). Epidemiologic study of the focus of cutaneous leishmaniasis in Mokolo (North Cameroon). [Bull Soc Pathol Exot Filiales.](javascript:AL_get(this,%20'jour',%20'Bull%20Soc%20Pathol%20Exot%20Filiales.');)72(5-6):442-50.

3. Dondji B (2001). Leishmanioses et phlebotomes du Cameroun: le point sur les donnees actuelles. Bull Soc Pathol Exot 94(5) :418-420.

4. Kaptue L, Zekeng L, Fomekong E, Nsangou A, Tagu JP Sanofi (1992). La leishmaniose viscérale au Cameroun. A propos de quelques observations et d’une prospection clinique dans la région de Kousseri, extrême-nord Camerounais. Bull. Soc. Pathol. Exot 85**:**156-158.
