# Supplementary material for: Leishmaniasis Worldwide and Global Estimates of Its Incidence
Source: PLoS One. 2012 May 31;7(5):e35671. doi: 10.1371/journal.pone.0035671 (PMC3365071; doi:10.1371/journal.pone.0035671)
Supplement: Text S16 — Leishmaniasis Country Profiles, Central African Republic. (DOCX) [file pone.0035671.s016.docx]

**CENTRAL AFRICAN REPUBLIC**


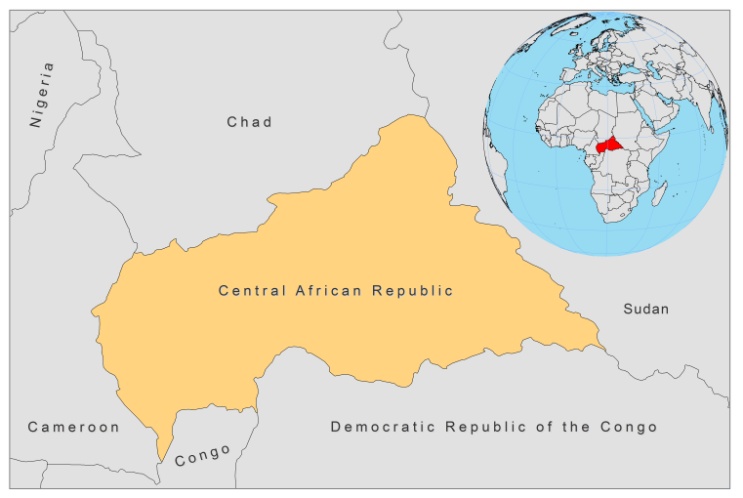


**BASIC COUNTRY DATA**

Total Population: 4,401,051

Population 0-14 years: 40%

Rural population: 61%

Population living under USD 1.25 a day: 62.8%

Population living under the national poverty line: 62%

Income status: Low income economy

Ranking: Low human development (ranking 179)

Per capita total expenditure on health at average exchange rate (US dollar): 19

Life expectancy at birth (years): 47

Healthy life expectancy at birth (years): 37

**BACKGROUND INFORMATION**

Very few data on prevalence or incidence are available.

The first VL case was reported in 1949 [1]. A second parasitologically proven case was seen in a child who seemed to have acquired the disease in the Bangui area; 3 other cases have been noted since 1969 [2]. Since then, no more cases have been reported.

Sporadic cases of CL have been reported in the past from the northwest and southwest regions of the Central African Republic [1]. More recently, no cases have been reported. In 2003, a case was imported from neighbouring Chad [3]. In 2007, there was a suspected case imported from Sudan, and in 2009, an unconfirmed CL case in a soldier from Birao region was treated successfully with antimonials (WHO, unpublished data). More cases of CL are suspected to be present among the military in this region.

**PARASITOLOGICAL INFORMATION**

| ***Leishmania* species** | **Clinical form** | **Vector species** | **Reservoirs** |
| --- | --- | --- | --- |
| *L. infantum* | ZVL | Unknown | Unknown |

**MAPS AND TRENDS**

**Cutaneous leishmaniasis**


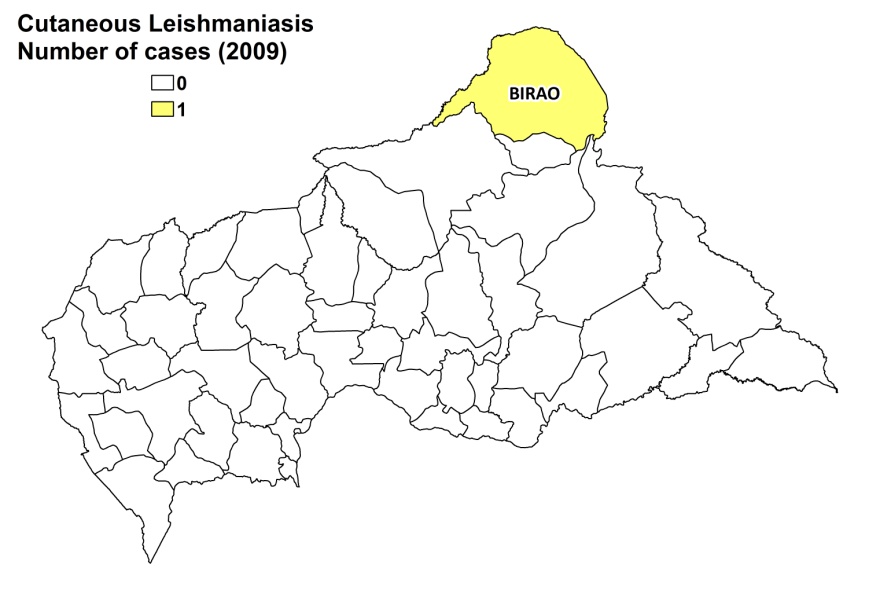


**CONTROL**

The notification of leishmaniasis is not mandatory in the country and there is no national leishmaniasis control program. There is no leishmaniasis vector control program and no leishmaniasis reservoir control program.

**DIAGNOSIS, TREATMENT**

There are no provisions for diagnosis and treatment of leishmaniasis. In 2009, a soldier suspected of having CL had to obtain antimonials (Glucantime, Sanofi) in Chad.

**ACCESS TO CARE**

No information.

**ACCESS TO DRUGS**

Amphotericin B and pentamidine are included in the National Essential Drug List (but not for leishmaniasis). No antimonials are registered.

**SOURCES OF INFORMATION**

- Dr Bernard Boua. Programme national de lutte contre les maladies tropicales négligées en République Centrafricaine, Ministry of Health. *Consultative Meeting on the Control of Leishmaniasis in the African Region WHO/AFRO Addis Ababa, 23-25 Feb 2010.*

1. Desjeux P (1991) Information on the epidemiology and control of the leishmaniases by country or territory. World Health Organization. WHO/LEISH/91.30.

2. Cagnard V, Lindrec A (1969). A case of visceral leishmaniasis in Bangui, Central African Republic. Med Trop 29(4):531-5.

3. Kassa-Kelembho E, Kobangue L, Huerre M, Morvan JM (2003). First cases of imported cutaneous leishmaniasis in Bangui Central African Republic: efficacy of metronidazole. Med Trop 63(6):597-600.
