# Supplementary material for: Leishmaniasis Worldwide and Global Estimates of Its Incidence
Source: PLoS One. 2012 May 31;7(5):e35671. doi: 10.1371/journal.pone.0035671 (PMC3365071; doi:10.1371/journal.pone.0035671)
Supplement: Text S17 — Leishmaniasis Country Profiles, Chad. (DOCX) [file pone.0035671.s017.docx]

**CHAD**


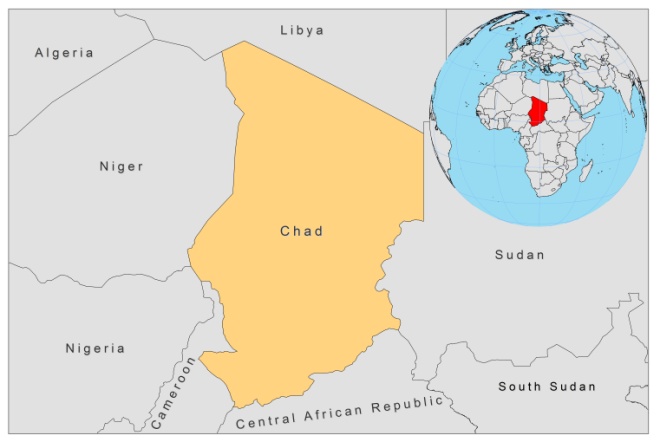


**BASIC COUNTRY DATA**

Total Population: 11,227,208

Population 0-14 years: 45%

Rural population: 72%

Population living under USD 1.25 a day: no data

Population living under the national poverty line: no data

Income status: Low income economy

Ranking: Low human development (ranking 183)

Per capita total expenditure on health at average exchange rate (US dollar): 42

Life expectancy at birth (years): 49

Healthy life expectancy at birth (years): 41

**BACKGROUND INFORMATION**

There are only very few data on prevalence, incidence and epidemiology of leishmaniasis in Chad.

VL is endemic, but cases are sporadic. VL occurs in the N’Djamena and Lake Chad areas, with foci extending eastward throughout southern Chad. Between 1966 and 1973, 64 cases were reported from the N'Djamena central hospital, and 2 infected dogs were identified within a 6-year period of observation in N'Djamena [1].

Between 1969 and 1971, 6 cases of oronasal leishmaniasis were reported in an area known to be endemic for VL [2,3]. In 1978, another case of oronasal leishmaniasis was detected. No more recent cases have been documented.

CL occurs in the Chari-Baguirmi Prefecture (which also includes N’Djamena), along the Chari River in south central Chad, and in the northern and northeastern sub-desert and desert areas. CL is more frequent than VL, with 121 reported cases in 1968, 836 in 1975 and 164 in the first semester of 1976 [2]. CL is also seen following population movements in Sudanese refugee camps in Chad [4]. In September 2007, an outbreak of CL happened in the refugee camp of Treguine, Adré district, east Chad, involving more than 200 patients (WHO records). Antimonials were provided for by WHO.

**PARASITOLOGICAL INFORMATION**

| ***Leishmania* species** | **Clinical form** | **Vector species** | **Reservoirs** |
| --- | --- | --- | --- |
| *Unknown* | VL | *P. orientalis* | *Unknown* |
| *L. major* | ZCL | *P. duboscqi,*  *P. bergeroti* | *Unknown* |

**MAPS AND TRENDS**

**CONTROL, DIAGNOSIS, TREATMENT, ACCESS TO CARE**

No information available.

**ACCESS TO DRUGS**

No medicines for leishmaniasis are registered.

**SOURCES OF INFORMATION**

1. Sirol J, Vedy J, Barabe P, Cesari C, Berger P (1976). Kala-Azar in the Republic of Chad. 6 year survey at the Central Hospital of N'Djamena (Fort-Lamy). Bull Soc Pathol Exot Filiales 69(3):232-7.

2. Desjeux P (1991) Information on the epidemiology and control of the leishmaniases by country or territory. World Health Organization. WHO/LEISH/91.30.

3. Sirol J, Vedy J, Delpy P (1971). Mucocutaneous leishmaniasis in Chad (1st 6 cases). Bull Soc Pathol Exot Filiales 64(6):856-65.

4. Bern C, Maguire JH, Alvar J (2008). [Complexities of assessing the disease burden attributable to leishmaniasis.](http://www.ncbi.nlm.nih.gov/pubmed/18958165?ordinalpos=1&itool=EntrezSystem2.PEntrez.Pubmed.Pubmed_ResultsPanel.Pubmed_DefaultReportPanel.Pubmed_RVDocSum) PLoS Negl Trop Dis 2(10):e313.
