# Supplementary material for: Leishmaniasis Worldwide and Global Estimates of Its Incidence
Source: PLoS One. 2012 May 31;7(5):e35671. doi: 10.1371/journal.pone.0035671 (PMC3365071; doi:10.1371/journal.pone.0035671)
Supplement: Text S18 — Leishmaniasis Country Profiles, China. (DOCX) [file pone.0035671.s018.docx]

**CHINA**


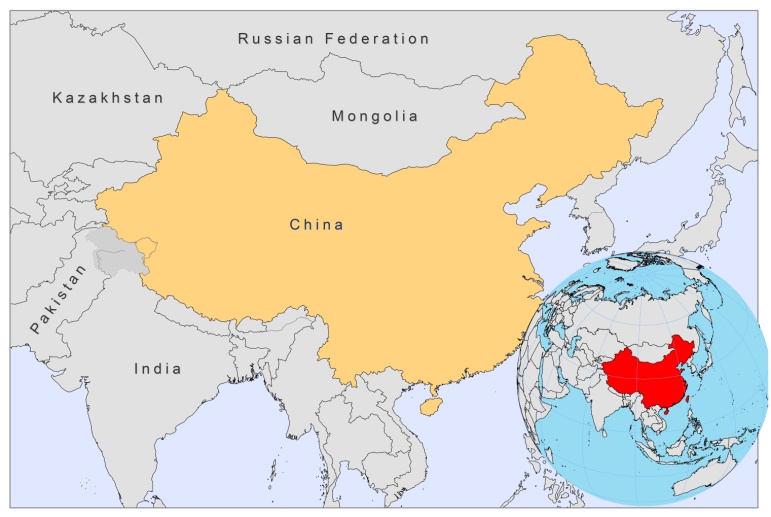


**BASIC COUNTRY DATA**

Total Population: 1,338,299,512

Population 0-14 years: 19%

Rural population: 55%

Population living under USD 1.25 a day: 15.9%

Population living under the national poverty line: no data

Income status: Upper middle income economy

Ranking: Medium human development (ranking 101)

Per capita total expenditure on health at average exchange rate (US dollar): 169

Life expectancy at birth (years): 73

Healthy life expectancy at birth (years): 64

**BACKGROUND INFORMATION**

VL is probably an old disease in China [1]. Symptoms of VL were already described in Chinese medical literature over 120 years ago. Before 1950, VL was highly endemic; in 1951, a survey concluded that an estimated 530,000 people had VL. Foci were in the northeast and the northwest of China. Following an 8-year campaign, with nationwide control measures (mass treatment of patients, killing of infected dogs and use of insecticides against the sandfly vectors), the disease has been under control in the north- east zoonotic foci since 1958 [2]. However, transmission was not interrupted in western China, where VL is still endemic with an increasing case load: about 2,629 cases were recorded between 1990 and 1999, and increased to 2,450 cases from 2005-2010 [3]. Most of these cases (98%) occurred in Xinjiang (50%), Gansu (34%) and Sichuan province (14%). A total of 61 counties, in 6 provinces, are now identified as highly endemic, accounting for 91% of cases. Both *L.donovani* and *L.infantum* are causative parasites in China. Sporadic cases are still reported from zoonotic foci of central and western China.

VL by *L.donovani* is present in the eastern plains and desert regions of eastern China [3]. In these areas, VL is now under control; no more cases have been reported since 1960. Since 1970, sporadic residual cases have been reported in the eastern part of the plains region, including PKDL cases.

VL by *L.infantum* seems to be present in two subtypes [3]. One is in the western high altitude regions of the provinces of Gansu, Sichuan, Shaanxi and Shanxi. Patients are mostly children under 10 years of age. Dogs are the principal source of infection, with infection rates up to 69.6%. The other subtype is in the deserts of the northwestern regions of China, including Xinjiang, western Inner Mongolia and northern Gansu. Transmission is probably sylvatic. In 2008 and 2009, an outbreak of this type occurred in Jiashi county, resulting in a twenty-fold increase of the incidence rate of VL compared to the average annual incidence rate [4]. The majority of the cases (96.6%) occurred in infants under 2 years of age.

CL by *L.infantum* occurred in Karamay, Xinjiang, showing a prevalence of 1.0%-1.6% [5]. CL by *L.major* is suspected to be present in Xingjian as well, with an endemic area reaching into Mongolia.

No cases of HIV/*Leishmania* co-infection have been reported.

**PARASITOLOGICAL INFORMATION**

| ***Leishmania* species** | **Clinical form** | **Vector species** | **Reservoirs** |
| --- | --- | --- | --- |
| *L. infantum* | ZVL, CL | *P. chinensis, P. alexandri,*  *P. wui* | *Canis familiaris*,  *Nyctereutes procyonoides* |
| *L. donovani* | AVL | *P. longiductus* | Human |
| ? (in Province of Taiwan) | CL, DCL | Unknown | Unknown |

**MAPS AND TRENDS**

**Visceral leishmaniasis**

**
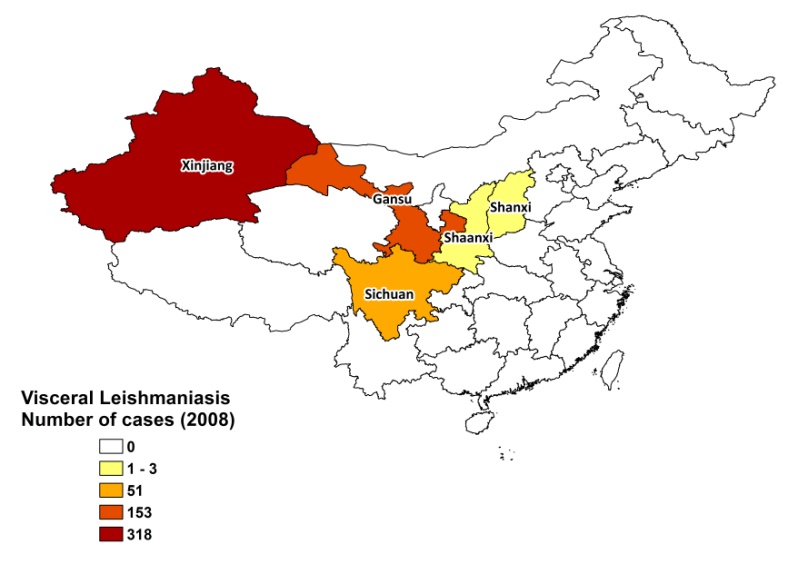

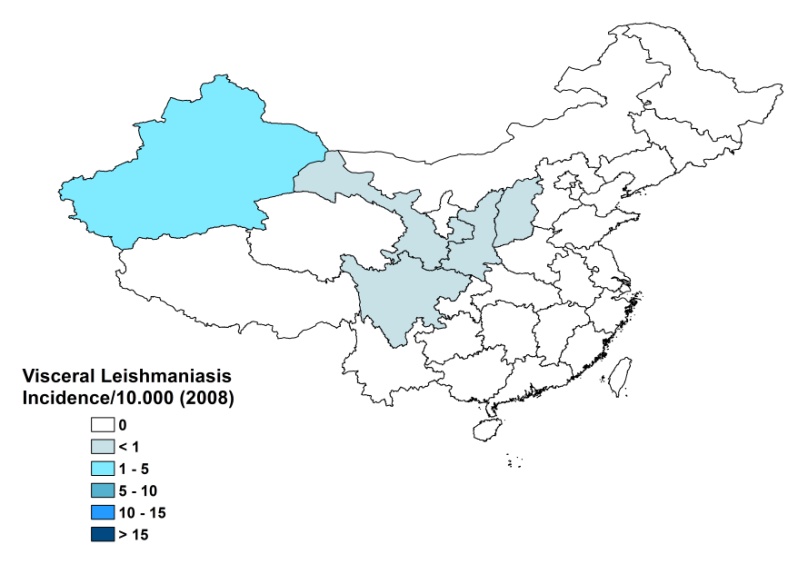
**

**Visceral leishmaniasis trend**

**CONTROL**

Notification of VL has been mandatory since 2004. There is no national leishmaniasis control program. There is no leishmaniasis vector control program, although in-house spraying takes place in some areas that are endemic for anthroponotic VL, and irregularly in Xinjiang. There is no leishmaniasis reservoir control program.

**DIAGNOSIS, TREATMENT**

**Diagnosis**

VL: rK39 antigen-based immunochromatographic test (ICT).

**Treatment**

VL: antimonials, 20 mg Sb^v^/kg/day for 10-12 days. Cure rate is 90%, relapses are seen in 10% and fatality rate is 1%.

**ACCESS TO CARE**

Care for leishmaniasis is not provided for free. Part of the costs are covered by the health system, but patients have to pay up to 1 USD for a consult, 4-70 USD for diagnostic tests and around 35 USD for drugs. Diagnosis and treatment for VL is only possible in district, secondary and tertiary hospitals. Not all patients are thought to have access to care. The main reasons are a lack of awareness among patients of the serious nature of the disease and a lack of awareness and training among health personnel. Patients also often live in very remote regions with no health facilities and transport, and are too poor to afford treatment.

**ACCESS TO DRUGS**

Sodium stibogluconate is included in the National Essential Drug List for VL. Antimonials are not available in private pharmacies. They are purchased by the provincial CDC (SSG, Xinhua pharmaceutical industry, Shandong, China) and provided to hospitals only.

**SOURCES OF INFORMATION**

- Dr Jun-yun Wang, National Institute of Parasitic Diseases, Chinese Center for Disease Control and Prevention; WHO Collaborating Center for Malaria, Schistosomiasis and Filariasis, Shanghai, 200025, China.

1. Leng YJ (1982). [A review of kala-azar in China from 1949 to 1959.](http://www.ncbi.nlm.nih.gov/pubmed/6926774) Trans R Soc Trop Med Hyg 76(4):531-7.

2. Zhi-Biao X (1989). [Present situation of visceral leishmaniasis in China.](http://www.ncbi.nlm.nih.gov/pubmed/15463223) Parasitol Today 5(7):224-8.

3. Guan LR (1991). [Current status of kala-azar and vector control in China.](http://www.ncbi.nlm.nih.gov/pubmed/1959161) Bull World Health Organ 69(5):595-601.

4. Wang JY, Gao CH, Yang YT, Chen HT, Zhu XH et al (2010). [An outbreak of the desert sub-type of zoonotic visceral leishmaniasis in Jiashi, Xinjiang Uygur Autonomous Region, People's Republic of China.](http://www.ncbi.nlm.nih.gov/pubmed/20434585) Parasitol Int 59(3):331-7.

5. Guan L, Yang Y, Xu Y, Qu J, Zho X et al (1994). Leishmaniasis in Karamay. XIV. Identification of promastigote isolates from naturally infected Phlebotomus major wui. Zhongguo Ji Sheng Chong Xue Yu Ji Sheng Chong Bing Za Zhi 12(4):257-61.
