# Supplementary material for: Leishmaniasis Worldwide and Global Estimates of Its Incidence
Source: PLoS One. 2012 May 31;7(5):e35671. doi: 10.1371/journal.pone.0035671 (PMC3365071; doi:10.1371/journal.pone.0035671)
Supplement: Text S19 — Leishmaniasis Country Profiles, Colombia. (DOCX) [file pone.0035671.s019.docx]

**COLOMBIA**


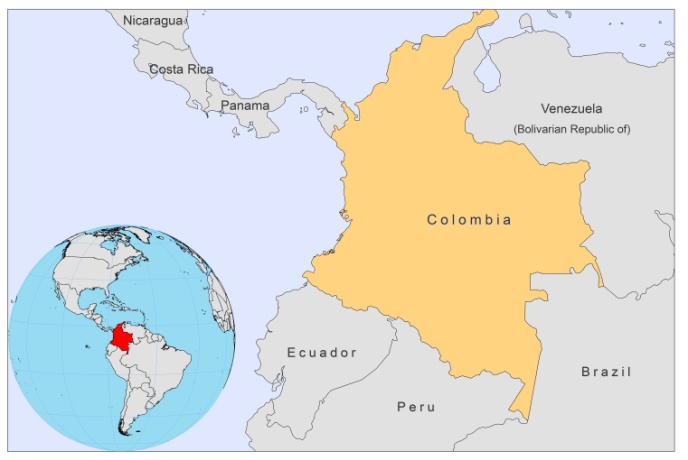


**BASIC COUNTRY DATA**

Total Population: 46,294,841

Population 0-14 years: 29%

Rural population: 25%

Population living under USD 1.25 a day: 16%

Population living under the national poverty line: 37.2%

Income status: Upper middle income economy

Ranking: High human development (ranking 87)

Per capita total expenditure on health at average exchange rate (US dollar): 323

Life expectancy at birth (years): 73

Healthy life expectancy at birth (years): 62

**BACKGROUND INFORMATION**

Colombia is one of the 3 countries with the highest number of *Leishmania* species affecting humans in the world (seven species in total) [1]. Around 90.3% of all cases are CL, 0.4% are MCL and 0.3% are VL [2]. The disease predominates in a large part of the country, especially in sylvatic foci, and in the interandean valleys, where working in coffee plantations can be seen as a risk factor [3]. Epidemic outbreaks of CL caused by *L. braziliensis, L. guyanensis* and *L. panamensis,* with intra and peridomiciliary transmission, have been reported since 1984 [4]. Urbanization of the disease has been observed in recent years [5]. There was a regular increase in the number of cases between 2003 and 2006, during which period the numbers rose from 9,417 to 17,205.

The greatest outbreak of CL occurred during the past 4 years (2005-2009), with more than 35,000 cases in the military forces, 80% caused by *L. braziliensis* and 20% caused by *L. panamensis*. Another important outbreak, affecting the civilian population, occurred in Andean valleys in 2003-2004, with 2,810 cases of CL caused by *L. guyanensis*, 80% of which were between 15 and 44 years old. This age-group distribution is influenced by an increase in the number of cases involved in the armed conflict. The population at risk is estimated at 12,277,606 people for CL and 6,795,047 people for MCL in 31 and 12 departments, respectively. In 2007, the incidence of CL was 106.5 cases/100,000 inhabitants; MCL represented 0.6% of the total.

VL by *L.infantum* has been diagnosed in Colombia since 1944. There are two clearly identified foci of VL in Colombia, the most important of which is in the northern coastal area, where *Lu. evansi* is the vector and accounts for 88% of the total number of cases. The second focus is located in the Magdalena river valley, where *Lu. longipalpis* is the vector and accounts for 12% of cases. Most cases (89%) occur in children under five years of age [6]. Around 3,449,831 people are at risk for VL in 6 departments. The incidence of VL was 5.4 cases/100,000 inhabitants in 2007.

No HIV-*Leishmania* co-infection has been reported.

**PARASITOLOGICAL INFORMATION**

| ***Leishmania* species** | **Clinical form** | **Vector species** | **Reservoirs** |
| --- | --- | --- | --- |
| *L. braziliensis* | ZCL, MCL | *Lu. spinicrassa,*  *Lu. colombiana,*  *Lu. pia,*  *Lu. towsendi* | *Canis familiaris, Akodon sp., Micoureus demerarae, Melanomys caliginosus, Rattus rattus, Didelphis marsupialis* |
| *L. panamensis* | ZCL, MCL | *Lu. trapidoi,*  *Lu. gomezi,*  *Lu. panamensis,*  *Lu. yuilli* | *Canis familiaris, Choloepus hoffmani, Metachirus nudicaudatus, Didelphis marsupialis, Coendou sp.* |
| *L. guyanensis* | ZCL, MCL | *Lu. umbratilis,*  *Lu. longiflocosa* | Unknown |
| *L. colombiensis* | ZCL | *Lu. hartmanni* | Unknown |
| *L. amazonensis* | ZCL, DCL | *Lu. flaviscutellata* | Unknown |
| *L. mexicana* | ZCL | *Lu. columbiana* | *Didelphis marsupialis* |
| *L. infantum* | ZVL | *Lu. longipalpis,*  *Lu. evansi* | *Canis familiaris,*  *Didelphis marsupialis* |

**MAPS AND TRENDS**

**Visceral leishmaniasis**


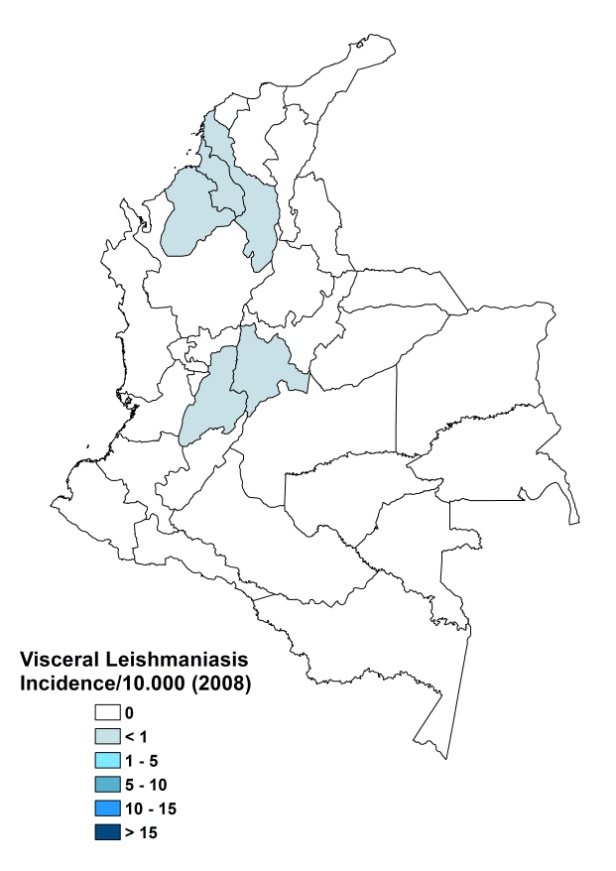

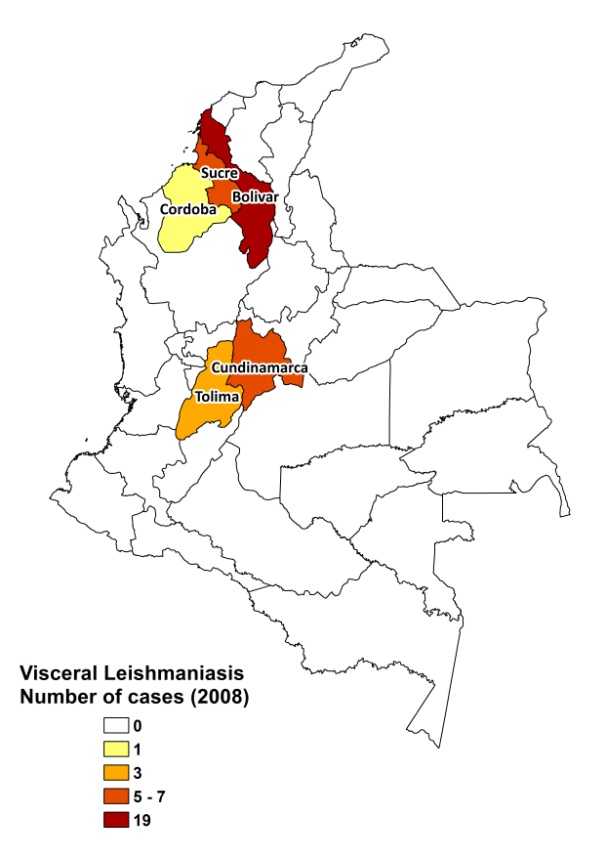


**Cutaneous leishmaniasis**


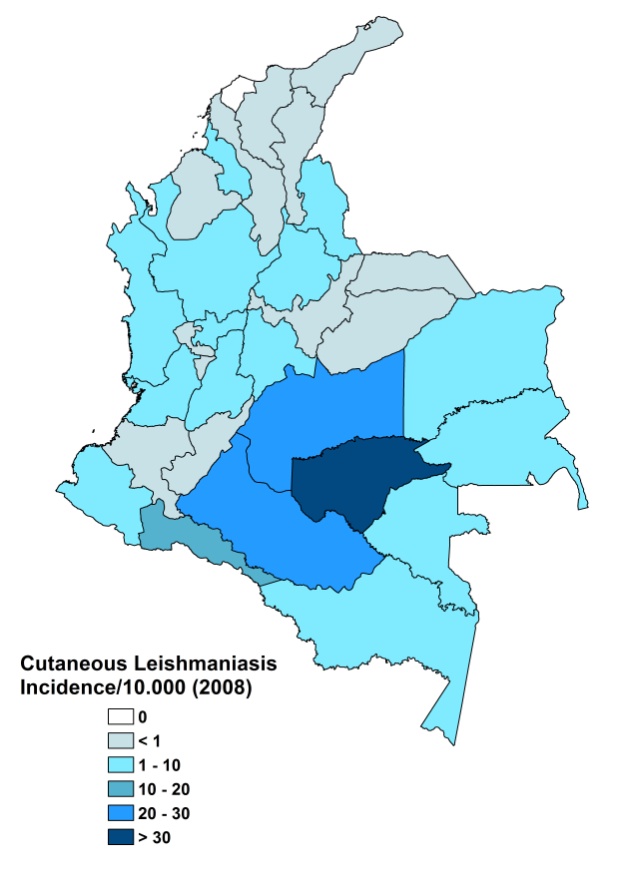

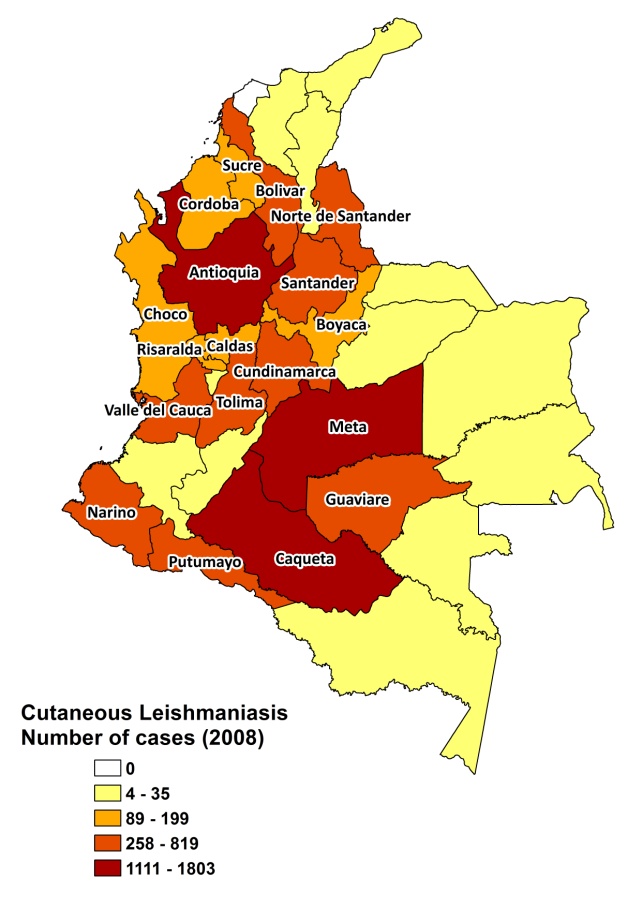


**Visceral leishmaniasis trend**

**Cutaneous leishmaniasis trend**

**Mucocutaneous leishmaniasis trend**

**CONTROL**

Leishmaniasis has been a notifiable disease since 1983 and the National Leishmaniasis Program was set up in 1998. Case detection is passive. There is a vector control program, with regular insecticide spraying and bednet distribution. There is a reservoir control program, where positive dogs are sacrificed and rodent control is performed.

The program suffers from the following limitations: under-reporting of cases (failure to identify them), inaccessibility of diagnosis, lack of human resources to provide diagnosis and treatment (rural communities), high turnover of medical and paramedical personnel in the municipalities, competition with other vector-borne diseases of high importance (malaria) and urban diseases (dengue).

**DIAGNOSIS, TREATMENT**

**Diagnosis**

CL: confirmation by microscopic examination of skin lesion sample and IFAT. Sometimes, ELISA is used.

VL: IFAT.

**Treatment**

VL: antimonials, 20 mg Sb^v^/kg/day for 28 days. Second line treatment is with miltefosine. 11 deaths were reported in the last five years.

CL: antimonials, 20 mg Sb^v^/kg/day for 20 days. In the last five years, there have been at least 5 deaths from CL, which were directly related to the toxicity of the antimonials. Second line treatment is with miltefosine, 2.5 mg/kg/day for 28-40 days. The cure rate of miltefosine is 90%.

MCL: antimonials, 20 mg Sb^v^/kg/day for 28 days. One death was reported due to complications of MCL. Second line treatment is with miltefosine, 2.5 mg/kg/day for 28-40 days. Third treatment is with pentamidine, 3 mg/kg/day, 4 to 7 doses. The cure rate for pentamidine is 90%.

The overall cure rate for antimonials is 90-95%.

**ACCESS TO CARE**

Treatment is acquired by the Ministry of Social Protection and provided free of charge to all patients with confirmed diagnosis of the disease. Sufficient drugs were obtained in order to treat all patients. Diagnosis for VL and (M)CL is possible at outreach level: blood samples are taken and sent for analysis by IFAT. Treatment with antimonials, miltefosine and pentamidine is possible at health center level. All patients are thought to have access to treatment.

**ACCESS TO DRUGS**

The drugs provided by the government are meglumine antimoniate (Glucantime, Sanofi), sodium stibogluconate (GR Intercomerce) and miltefosine (Tecnopharma).

Meglumine antimoniate is included in the national Essential Drug List for leishmaniasis. The list will soon be updated with sodium stibogluconate, miltefosine and pentamidine. Drugs for leishmaniasis are not sold in private pharmacies in order to prevent misuse. Sodium stibogluconate (Pentostam, GSK), meglumine antimoniate (Glucantime, Sanofi) and miltefosine (Paladin, Canada) are registered in Colombia, as well as generic forms of miltefosine and SSG.

**SOURCES OF INFORMATION**

- Dr Pilar Zambrano H. - National Health Institute. *Leishmaniasis en la Región de las Américas. Reunión de coordinadores de Programa Nacional de Leishmaniasis. OPS/OMS. Medellín, Colombia. 4-6 junio 2008.*
- Drs Julio Cesar Padilla and Maria Cristina Carrasquilla, National Program of Vector Borne Diseases, Ministerio de la Protección Social, Colombia. *Leishmaniasis en la Región de las Américas. Reunión de coordinadores de Programa Nacional de Leishmaniasis. OPS/OMS. Medellín, Colombia. 4-6 junio 2008.*

1. Corredor A, Gallego J, Tesh RB, Morales A, Ferro C et al (1989) Epidemiology of visceral leishmaniasis in Colombia. Am J Trop Med Hyg 5:480-486.

2. Weigle KA, Santrich C, Martinez F, Valderrama L, Saravia NG (1993) [Epidemiology of cutaneous leishmaniasis in Colombia: a longitudinal study of the natural history, prevalence, and incidence of infection and clinical manifestations.](http://www.ncbi.nlm.nih.gov/pubmed/8354912) J Infect Dis 168(3):699-708.

3. Alexander B, Agudelo LA, Navarro JF, Ruiz JF, Molina J et al (2009). [Relationship between coffee cultivation practices in Colombia and exposure to infection with Leishmania.](http://www.ncbi.nlm.nih.gov/pubmed/19555985)Trans R Soc Trop Med Hyg 103(12):1263-8.

4. Valderrama-Ardila C, Alexander N, Ferro C, Cadena H, Marín D et al (2010). [Environmental risk factors for the incidence of American cutaneous leishmaniasis in a sub-Andean zone of Colombia (Chaparral, Tolima).](http://www.ncbi.nlm.nih.gov/pubmed/20134000) Am J Trop Med Hyg 82(2):243-50.

5. Cortés LA, Fernández JJ (2008) [Species of Lutzomyia involved in an urban focus of visceral and cutaneous leishmaniasis](http://www.ncbi.nlm.nih.gov/pubmed/19034365). Biomedica 28(3):433-40.

6. Velez ID, Travi BL, Gallego J, Palma GI, Agudelo S et al (1995). Ecoepidemiological evaluation of visceral leishmanias in the native Zenu community of San Andres de Sotavento Cordoba: first step for its control. Rev, Col. Entomol 21(3): 111-122.
