# Supplementary material for: Leishmaniasis Worldwide and Global Estimates of Its Incidence
Source: PLoS One. 2012 May 31;7(5):e35671. doi: 10.1371/journal.pone.0035671 (PMC3365071; doi:10.1371/journal.pone.0035671)
Supplement: Text S20 — Leishmaniasis Country Profiles, Costa Rica. (DOCX) [file pone.0035671.s020.docx]

**COSTA RICA**


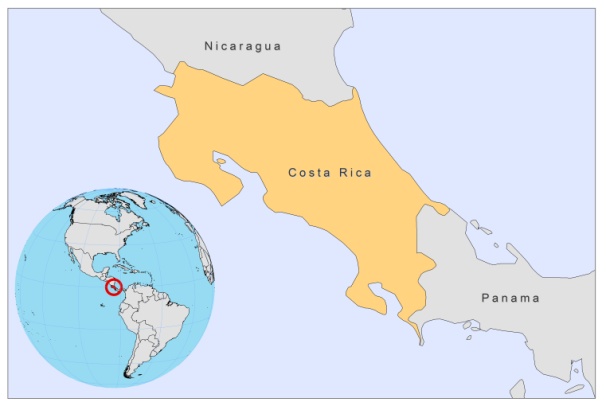


**BASIC COUNTRY DATA**

Total Population: 4,658,887

Population 0-14 years: 25%

Rural population: 36%

Population living under USD 1.25 a day: 0.7%

Population living under the national poverty line: 24.2%

Income status: Upper middle income economy

Ranking: High human development (ranking 69)

Per capita total expenditure on health at average exchange rate (US dollar): 668

Life expectancy at birth (years): 79

Healthy life expectancy at birth (years): 67

**BACKGROUND INFORMATION**

CL is considered an important public health problem and is highly endemic in different regions. In 1986-1987 in the Guanacaste province, northwestern Costa Rica, an outbreak of CL occurred among Nicaraguan refugees, affecting 200 people, the majority of which were children. The etiological agent was identified as *L. infantum* [1]. The number of cases has been increasing during the past years, rising from 690 in 2002 to 1,870 in 2007. The reported incidence of CL was 27.3 cases/10,000 inhabitants in 2006.

One case of VL has been reported so far [2].

**PARASITOLOGICAL INFORMATION**

| ***Leishmania* species** | **Clinical form** | **Vector species** | **Reservoirs** |
| --- | --- | --- | --- |
| *L. panamensis* | ZCL, MCL | *Lu. ylephiletor,*  *Lu. trapidoi* | *Bradypus griseus, Choloepus hoffmanni, Heteromys desmarestianus* |
| *L. mexicana* | ZCL, MCL, DCL | *Lu. olmeca olmeca,*  *Lu. olmeca bicolor* | Unknown |
| *L. braziliensis* | ZCL, MCL | *Lu. youngi* | Unknown |
| *L. garnhami* | ZCL | *Lu. youngi* | Unknown |
| *L. infantum* | ZCL | *Lu. longipalpis,*  *Lu. evansi* | *Canis familiaris* |

**MAPS AND TRENDS**

**Cutaneous leishmaniasis**

**
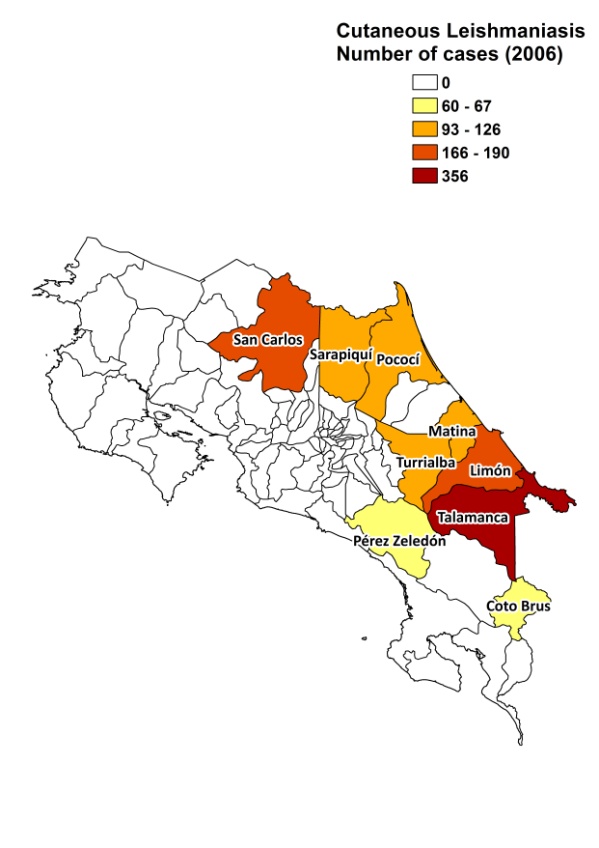
**

**
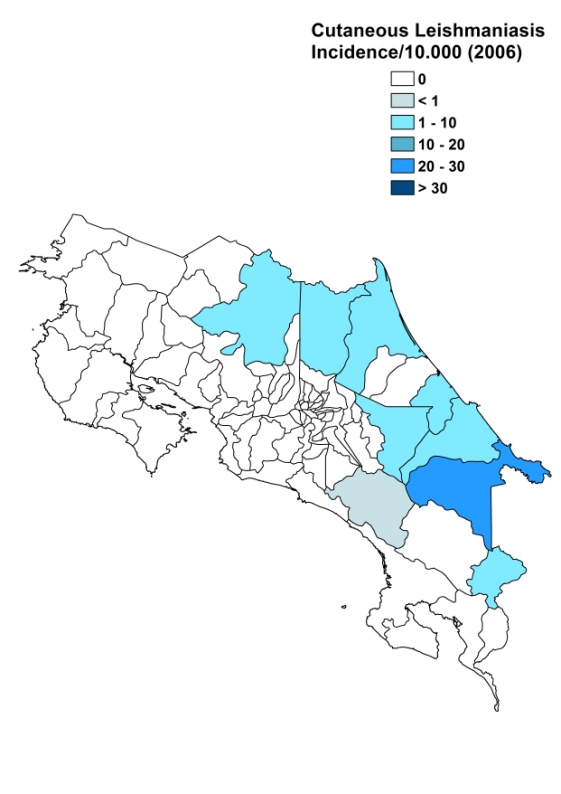
**

**Cutaneous leishmaniasis trend**

**CONTROL**

The notification of leishmaniasis is mandatory and there is a national leishmaniasis control program. Case detection is passive. Control strategies rely on case diagnosis and timely treatment; there is no vector or reservoir control program.

**DIAGNOSIS, TREATMENT**

**Diagnosis**: no data

**Treatment**:

CL and VL: antimonials.

**ACCESS TO CARE**

The Costa Rican Social Security Fund, which is the agency responsible for the provision of health services, provides coverage for approximately 90% of the population.

**ACCESS TO DRUGS**

Meglumine antimoniate (Glucantime, Sanofi) is registered and used for treatment in public health facilities.

**SOURCES OF INFORMATION**

- Dr Teresita Solano – Ministry of Health. *Leishmaniasis en la Región de las Américas. Reunión de coordinadores de Programa Nacional de Leishmaniasis. OPS/OMS. Medellín, Colombia. 4-6 junio 2008.*

1. Zeledón R (1991). [Cutaneous leishmaniasis and Leishmania infantum.](http://www.ncbi.nlm.nih.gov/pubmed/1755077) Trans R Soc Trop Med Hyg 85(4):557.

2. Carrillo J, Chinchilla M, Valverde B, Porras O, Mora L (1999). [Visceral leishmaniasis in Costa Rica: first case report.](http://www.ncbi.nlm.nih.gov/pubmed/10530466) Clin Infect Dis 29(3):678-9.
