# Supplementary material for: Leishmaniasis Worldwide and Global Estimates of Its Incidence
Source: PLoS One. 2012 May 31;7(5):e35671. doi: 10.1371/journal.pone.0035671 (PMC3365071; doi:10.1371/journal.pone.0035671)
Supplement: Text S21 — Leishmaniasis Country Profiles, Cote d’Ivoire. (DOCX) [file pone.0035671.s021.docx]

**CÔTE d'IVOIRE**


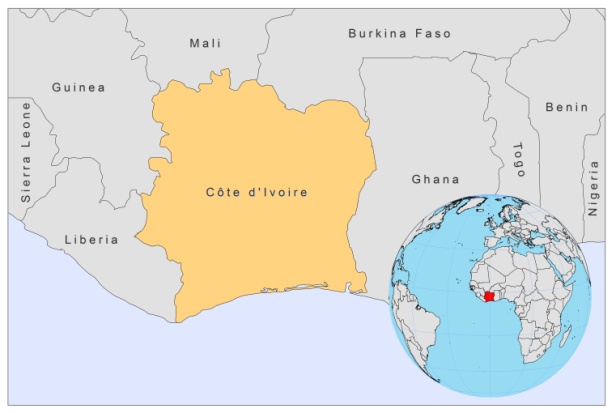


**BASIC COUNTRY DATA**

Total Population: 19,737,800

Population 0-14 years: 41%

Rural population: 50%

Population living under USD 1.25 a day: 23.8%

Population living under the national poverty line: 42.7%

Income status: Lower middle income economy

Ranking: Low human development (ranking 170)

Per capita total expenditure on health at average exchange rate (US dollar): 57

Life expectancy at birth (years): 54

Healthy life expectancy at birth (years): 39

**BACKGROUND INFORMATION**

Although Ivory Coast is part of a proposed CL endemicity belt running across West Africa, both VL and CL have been very sporadically reported in the last 15 years. Only 11 cases of VL, and no CL, were reported between 1965 and 1993 and from 1995 to 2008, a further 4 VL cases and 5 CL cases were reported. Endemic areas are difficult to determine as patients' locations are often imprecise and diagnostic facilities are not present outside of Abidjan and Bouake region. 89% of cases are autochtonous. 44% of the reported cases are coinfected with HIV. VL was documented in 5 individuals, 2 of which were immunosuppressed, one was HIV infected and one was under the age of 5 [1,2].

Both VL and CL may occur regularly and go unreported, due to a lack of diagnostic facilities and a lack of awareness among health personnel.

**PARASITOLOGICAL INFORMATION**

| ***Leishmania* species** | **Clinical form** | **Vector species** | **Reservoirs** |
| --- | --- | --- | --- |
| Unknown | VL, CL | Unknown | Unknown |

**MAPS AND TRENDS**

**Cutaneous leishmaniasis**


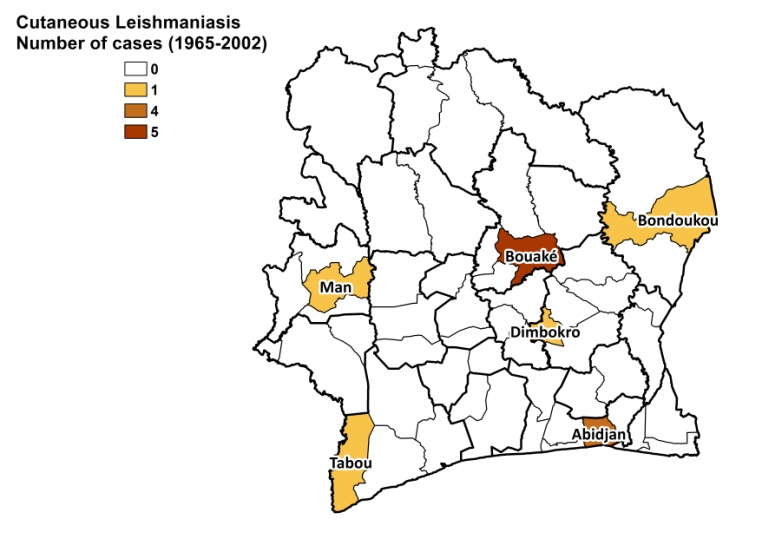

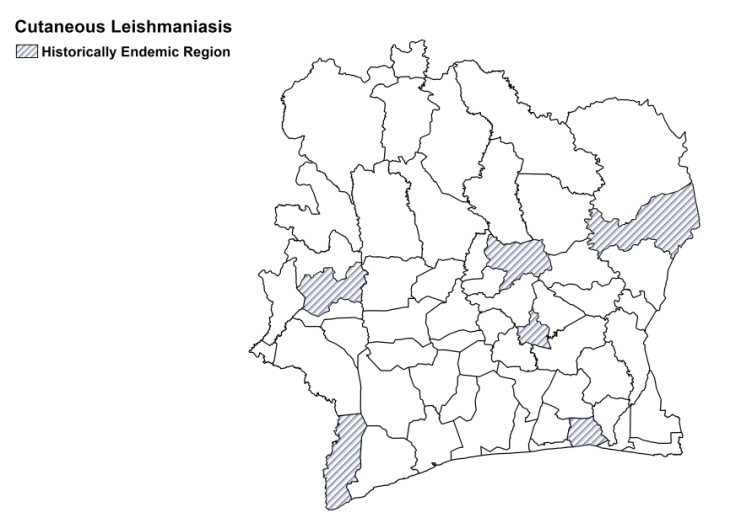


**Visceral leishmaniasis**


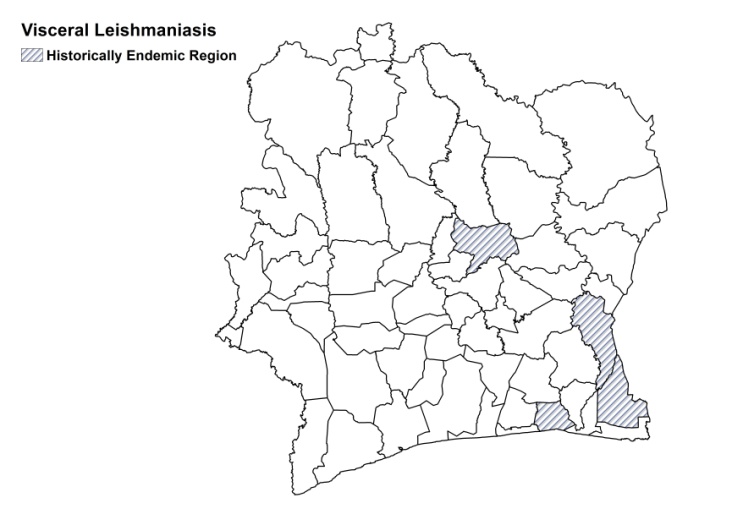

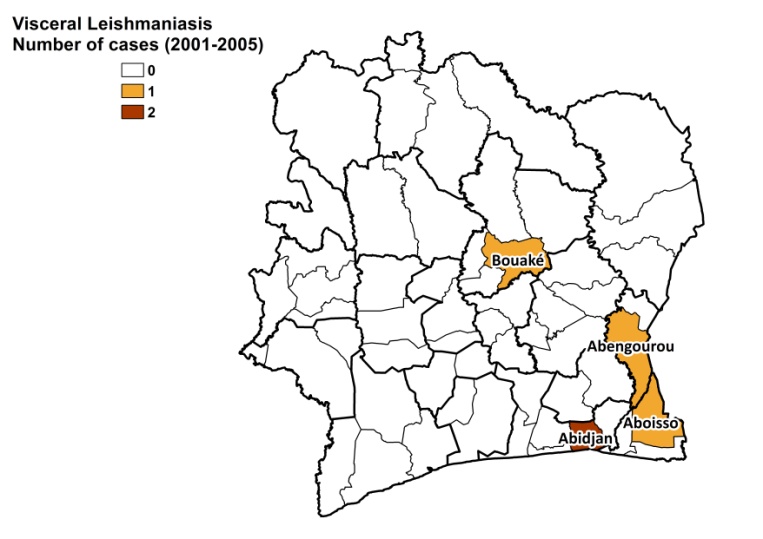


**Number of cases**

|  | **1995** | **1996** | **1997** | **1998** | **1999** | **2000** | **2001** | **2002** | **2003** | **2004** | **2005** | **2006** | **2007** | **2008** |
| --- | --- | --- | --- | --- | --- | --- | --- | --- | --- | --- | --- | --- | --- | --- |
| **VL** | 0 | 0 | 0 | 0 | 1 | 1 | 1 | 1 | 0 | 0 | 0 | 0 | 0 | 0 |
| **CL** | 0 | 0 | 0 | 0 | 0 | 0 | 2 | 0 | 0 | 2 | 1 | 0 | 0 | 0 |

**CONTROL**

The notification of leishmaniasis is not mandatory in the country and there is no national leishmaniasis control program. There is no leishmaniasis vector control program and insecticide spraying is not regularly done. There is a no leishmaniasis reservoir control program.

**DIAGNOSIS, TREATMENT**

**Diagnosis:**

VL: confirmation by microscopic examination of lymph node tissue sample.

CL: confirmation by microscopic examination of tissue sample.

**Treatment:**

VL: antimonials, 60 mg Sb^v^/kg/day meglumine antimoniate equivalent to 16.8 mg Sbv /kg/day for 20 days. Cure rate is 46%. Second line treatment is with amphotericin B, 50 mg total dose every 2 days or a combination of pentamidine, 4 mg/kg/day, and metronidazole, 500 mg 3 dd. Cure rate of this regimen is 15%. Overall cure rate of VL is 40%, fatality rate is 60%.

CL: antimonials, intralesional or systemic, 60 mg Sb^v^/kg/day meglumine antimoniate equivalent to 16.8 mg Sbv /kg/day for 20 days. The cure rate is 93%, with a fatality rate of 7%.

**ACCESS TO CARE**

Most health practitioners are not trained to diagnose leishmaniasis. Diagnosis and treatment is only possible in 4 hospitals.

**ACCESS TO DRUGS**

No antimonials are registered. Meglumine antimoniate (Glucantime, Sanofi) is used in hospitals.

**SOURCES OF INFORMATION**

1. [Kouassi B](http://www.ncbi.nlm.nih.gov/pubmed?term=%22Kouassi%20B%22%5BAuthor%5D), [Horo K](http://www.ncbi.nlm.nih.gov/pubmed?term=%22Horo%20K%22%5BAuthor%5D), [Achi VH](http://www.ncbi.nlm.nih.gov/pubmed?term=%22Achi%20VH%22%5BAuthor%5D), [Adoubryn KD](http://www.ncbi.nlm.nih.gov/pubmed?term=%22Adoubryn%20KD%22%5BAuthor%5D), [Kakou ES](http://www.ncbi.nlm.nih.gov/pubmed?term=%22Kakou%20ES%22%5BAuthor%5D) et al (2005). Three cases of visceral leishmaniasis in Abidjan, Cote d'Ivoire. Med Trop 65(6):602-3.

2. [Eholié SP](http://www.ncbi.nlm.nih.gov/pubmed?term=%22Eholi%C3%A9%20SP%22%5BAuthor%5D), [Tanon AK](http://www.ncbi.nlm.nih.gov/pubmed?term=%22Tanon%20AK%22%5BAuthor%5D), [Folquet-Amorissani M](http://www.ncbi.nlm.nih.gov/pubmed?term=%22Folquet-Amorissani%20M%22%5BAuthor%5D), [Doukouré B](http://www.ncbi.nlm.nih.gov/pubmed?term=%22Doukour%C3%A9%20B%22%5BAuthor%5D), [Adoubryn KD](http://www.ncbi.nlm.nih.gov/pubmed?term=%22Adoubryn%20KD%22%5BAuthor%5D) et al (2008). Three new cases of visceral leishmaniasis in Côte d'Ivoire. [Bull Soc Pathol Exot.](javascript:AL_get(this,%20'jour',%20'Bull%20Soc%20Pathol%20Exot.');)101(1):60-1.
