# Supplementary material for: Leishmaniasis Worldwide and Global Estimates of Its Incidence
Source: PLoS One. 2012 May 31;7(5):e35671. doi: 10.1371/journal.pone.0035671 (PMC3365071; doi:10.1371/journal.pone.0035671)
Supplement: Text S22 — Leishmaniasis Country Profiles, Croatia. (DOCX) [file pone.0035671.s022.docx]

**CROATIA**


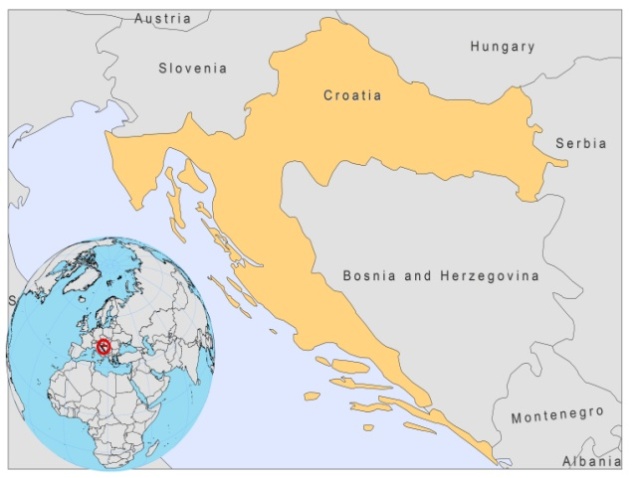


**BASIC COUNTRY DATA**

Total Population: 4,424,161

Population 0-14 years: 15%

Rural population: 42%

Population living under USD 1.25 a day: no data

Population living under the national poverty line: no data

Income status: High income economy

Ranking: Very high human development (ranking 46)

Per capita total expenditure on health at average exchange rate (US dollar): 1,120

Life expectancy at birth (years): 76

Healthy life expectancy at birth (years): 67

**BACKGROUND INFORMATION**

VL and CL are sporadic and hypo-endemic. A total of 124 cases of VL and CL have been reported from 1954 until the end of 2006. During 1999-2009, 49 cases were reported, of which 32 with VL and 17 with CL. 39% of patients were under 15 years of age [1]. The disease is more prevalent in males (72% of cases). Underreporting is suspected to a small degree.

Dogs are the main reservoir [2], although the wolf has been incriminated [3]. A cross-sectional serological survey on 306 dogs (in Split) revealed an average prevalence of infection of 15%.

Four HIV coinfected leishmaniasis cases have been reported.

**PARASITOLOGICAL INFORMATION**

| ***Leishmania* species** | **Clinical form** | **Vector species** | **Reservoirs** |
| --- | --- | --- | --- |
| *L infantum* | ZVL, CL | *P. tobbi,*  *P. neglectus* | *Canis familiaris,*  *Canis lupus* |

**MAPS AND TRENDS**

**Visceral leishmaniasis**

**
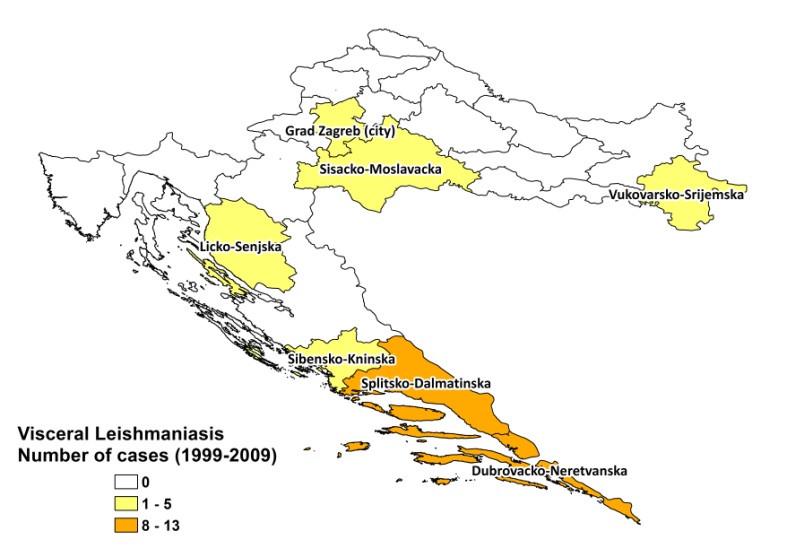

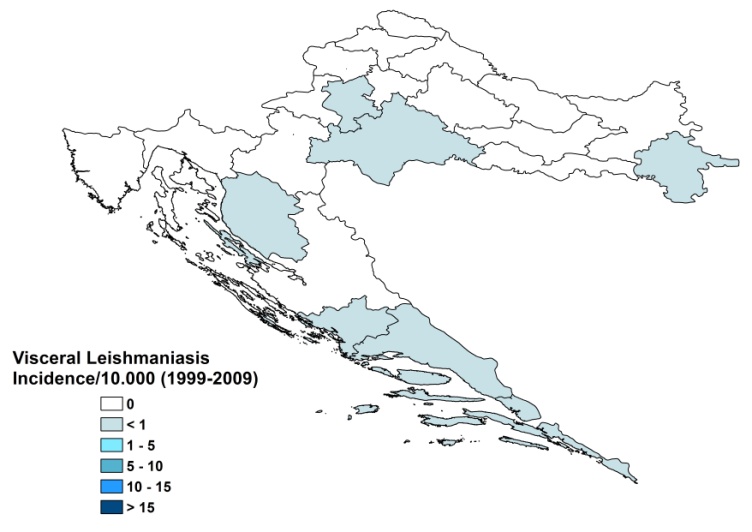
**

**Leishmaniasis trend**

|  | **2000** | **2001** | **2002** | **2003** | **2004** | **2005** | **2006** | **2007** | **2008** | **2009** |
| --- | --- | --- | --- | --- | --- | --- | --- | --- | --- | --- |
| **CL cases** | 1 | 1 | 3 | 1 | 0 | 2 | 1 | 5 | 3 |  |
| **VL cases** | 2 | 1 | 2 | 2 | 2 | 7 | 6 | 4 | 4 | 1 |

**CONTROL**

The notification of CL and VL is mandatory in the country. There is a national leishmaniasis control program for VL and CL. Case detection is passive. There is no leishmaniasis vector control program and no bednet distribution program. Insecticide spraying is not done. There is a leishmaniasis reservoir control program. Serological surveys of dogs are regularly performed. Positive dogs are not sacrificed.

**DIAGNOSIS, TREATMENT**

**Diagnosis**

VL: microscopic examination of bone marrow aspirate, cultures and serological diagnosis with IFAT. At tertiary level, PCR is possible.

CL: confirmation with microscopic examination of skin lesion sample and cultures. At tertiary level, PCR is possible.

**Treatment**

VL: antimonials, 20 Sb^v^/mg/kg/day for 28 days. Second line: conventional, or lipid formulations of amphotericin B. Overall cure rate was 91%, with a fatality rate of 9% (3 patients).

CL: antimonials.

**ACCESS TO CARE**

Treatment for leishmaniasis is provided for free. All patients are believed to have access to treatment with antimonials. Access to second line treatment with liposomal amphotericin B, which is only given in specialized hospitals, is problematic as it costs up to 17,000 Euros for a full treatment of a patient of 70 kg. Both antimonials and liposomal amphotericin B are not included in the list of the Croatian Institute for Health Insurance (National Essential Drug List) and have to be paid for by hospital budgets. Hospitals purchased 130 vials of liposomal amphotericin B (AmBisome, Gilead) in 2007 and 160 in 2008, plus 5 and 6 vials of sodium stibogluconate (Pentostam, GSK) in the same years, leading to a total drug cost of about 100,000 Euros for the treatment of 17 patients.

Diagnosis is only performed in second and third line hospitals.

**ACCESS TO DRUGS**

Colloidal amphotericin B (Amphocil), conventional amphotericin B and liposomal amphotericin B (AmBisome) are registered in Croatia. Only the first two, colloidal amphotericin B (Amphocil) and conventional amphotericin B, are included in the essential drug list and provided for by the Ministry of Health. No antimonials are registered nor included in the essential drug list, which creates considerable difficulties in obtaining them. Drugs for leishmaniasis are unavailable in private pharmacies.

**SOURCES OF INFORMATION**

- Dr Davorka Lukas, University Hospital for Infectious Diseases “Dr Fran Mihaljević” Zagreb. *Leishmaniasis in the European Region, a WHO consultative intercountry meeting, Istanbul, Turkey, 17–19 November 2009.*

1. [Mulić R](http://www.ncbi.nlm.nih.gov/pubmed?term=%22Muli%C4%87%20R%22%5BAuthor%5D), [Custović A](http://www.ncbi.nlm.nih.gov/pubmed?term=%22Custovi%C4%87%20A%22%5BAuthor%5D), [Ropac D](http://www.ncbi.nlm.nih.gov/pubmed?term=%22Ropac%20D%22%5BAuthor%5D), [Tripković I](http://www.ncbi.nlm.nih.gov/pubmed?term=%22Tripkovi%C4%87%20I%22%5BAuthor%5D), [Stojanović D](http://www.ncbi.nlm.nih.gov/pubmed?term=%22Stojanovi%C4%87%20D%22%5BAuthor%5D) [et](http://www.ncbi.nlm.nih.gov/pubmed?term=%22Klismani%C4%87%20Z%22%5BAuthor%5D) al (2009). Occurence of visceral and cutaneous leishmaniasis in Croatia. [Mil Med.](javascript:AL_get(this,%20'jour',%20'Mil%20%0d%0aMed.');)174(2):206-11.

2. Zivicnjak T, Martinković F, Marinculić A, Mrljak V, Kucer N et al (2005). [A seroepidemiologic survey of canine visceral leishmaniosis among apparently healthy dogs in Croatia.](http://www.ncbi.nlm.nih.gov/pubmed/15946800) Vet Parasitol 131(1-2):35-43.

3. Beck A, Beck R, Kusak J, Gudan A, Martinkovic F et al (2008). [A case of visceral leishmaniosis in a gray wolf (Canis lupus) from Croatia.](http://www.ncbi.nlm.nih.gov/pubmed/18436678) J Wildl Dis 44(2):451-6.
