# Supplementary material for: Leishmaniasis Worldwide and Global Estimates of Its Incidence
Source: PLoS One. 2012 May 31;7(5):e35671. doi: 10.1371/journal.pone.0035671 (PMC3365071; doi:10.1371/journal.pone.0035671)
Supplement: Text S23 — Leishmaniasis Country Profiles, Cyprus. (DOCX) [file pone.0035671.s023.docx]

**CYPRUS**


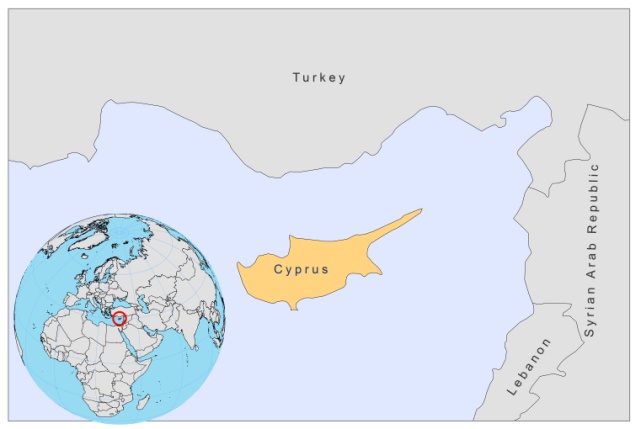


**BASIC COUNTRY DATA**

Total Population: 1,103,647

Population 0-14 years: 18%

Rural population: 30%

Population living under USD 1.25 a day: no data

Population living under the national poverty line: no data

Income status: High income economy

Ranking: Very high human development (ranking 31)

Per capita total expenditure on health at average exchange rate (US dollar): 1,747

Life expectancy at birth (years): 79

Healthy life expectancy at birth (years): 68

**BACKGROUND INFORMATION**

Canine VL was highly prevalent in the southern part of Cyprus before 1945, but after extensive insecticide spraying in the 1950s and a reduction of the dog population from 46,000 to 6,000 dogs during the echinococcosis campaign between 1970 and 1975, it was thought to be eradicated. However, the dog population steadily increased again and entomological surveys of 1944 and 1985 showed that the sandfly fauna remained essentially unchanged, despite regular and widespread insecticide use. As a consequence, canine VL reemerged in 1996 [1].

A survey, carried out in 2005-2006, showed a 9-fold increase in dog seroprevalence (from 1.7% to 14.9%) in 10 years [2]. However, paradoxically, only 2 cases of human VL by *L.infantum* have been reported in the southern part of Cyprus since 1935. As a number of tourists developed VL after visiting the southern part of Cyprus, it is thought that indigenous -the southern part- Cypriots may have a protective mechanism against *L.infantum*. Controversially, in the northern part of Cyprus, the number of CL cases increased from 2 to 36 between 1985 and 1990 and one case of VL was reported.

CL had not been reported since 1935, but in 2006, 6 cases of leishmaniasis by *L.donovani* were reported in the southern part of Cyprus, making it the first Mediterranean country where this occurred [3]. It is not known if the strain was imported. As in Sri Lanka, *L.donovani* caused CL in healthy individuals, but 2 cases of VL occurred in an immunosuppressed and an elderly patient. The spread of *L.donovani* along the Mediterranean coast is of concern, as this could have a profound effect on the epidemiology of leishmaniasis.

**PARASITOLOGICAL INFORMATION**

| ***Leishmania* species** | **Clinical form** | **Vector species** | **Reservoirs** |
| --- | --- | --- | --- |
| *L infantum* | ZVL, CL | *P. tobbi* | *Canis familiaris* |
| *L. donovani* | VL, CL | Unknown |  |

**MAPS AND TRENDS**

**Cutaneous and visceral leishmaniais**


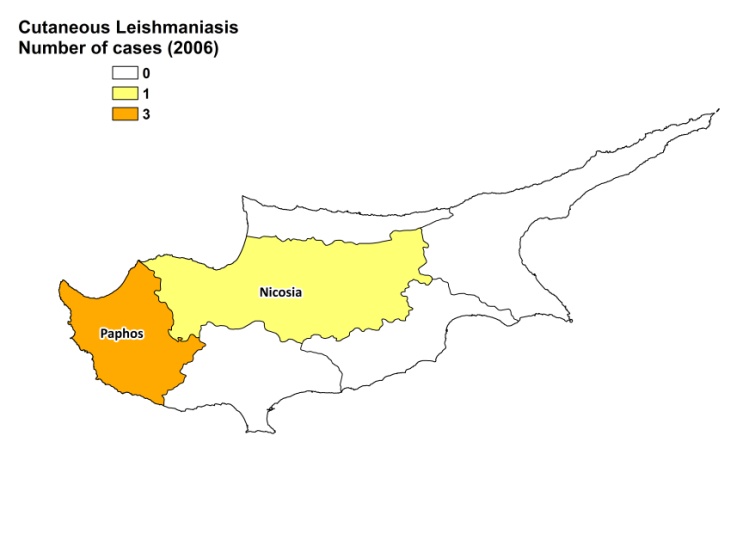

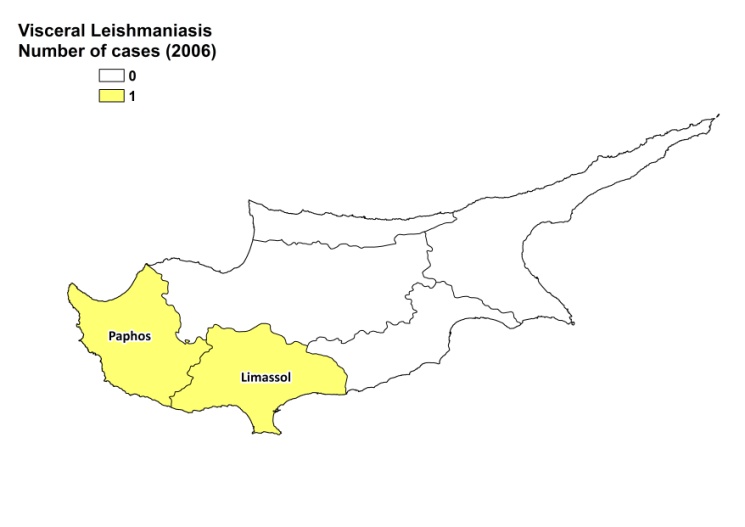


**Leishmaniasis trend**

|  | **2006** | **2007** | **2008** |
| --- | --- | --- | --- |
| **CL cases** | 4 | 0 | 0 |
| **VL cases** | 2 | 0 | 0 |

**CONTROL**

The notification of leishmaniasis in humans and dogs is mandatory in the country. There is no national leishmaniasis control program. There is no leishmaniasis vector control program, although regular insecticide spraying is done, and no leishmaniasis reservoir control program.

**DIAGNOSIS, TREATMENT**

**Diagnosis**:

CL and VL: microscopy, serological tests and PCR.

**Treatment:**

VL: amphotericin B lipid complex or liposomal amphotericin B, 3 mg/kg/day, day 1-5 and day 10. Cure rate is >95%.

**ACCESS TO CARE**

All patients are believed to have access to care.

**ACCESS TO DRUGS**

Lipid formulations of amphotericin B are registered. No antimonials are registered. Miltefosine is only registered for dogs.

**SOURCES OF INFORMATION**

1. Deplazes P, Grimm F, Papaprodromou M, Cavaliero T, Gramiccia M et al (1998). [Canine leishmaniosis in Cyprus due to Leishmania infantum MON 1.](http://www.ncbi.nlm.nih.gov/pubmed/9821465) Acta Trop 71(2):169-78.

2. Mazeris A, Soteriadou K, Dedet JP, Haralambous C, Tsatsaris A et al (2010). [Leishmaniases and the Cyprus paradox.](http://www.ncbi.nlm.nih.gov/pubmed/20207870) Am J Trop Med Hyg 82(3):441-8.

3. Antoniou M, Haralambous C, Mazeris A, Pratlong F, Dedet JP et al (2008) [Leishmania donovani leishmaniasis in Cyprus.](http://www.ncbi.nlm.nih.gov/pubmed/18156082) Lancet Infect Dis 8(1):6-7.
