# Supplementary material for: Leishmaniasis Worldwide and Global Estimates of Its Incidence
Source: PLoS One. 2012 May 31;7(5):e35671. doi: 10.1371/journal.pone.0035671 (PMC3365071; doi:10.1371/journal.pone.0035671)
Supplement: Text S24 — Leishmaniasis Country Profiles, Democratic Republic of the Congo. (DOCX) [file pone.0035671.s024.docx]

**DEMOCRATIC REPUBLIC OF THE CONGO**

**
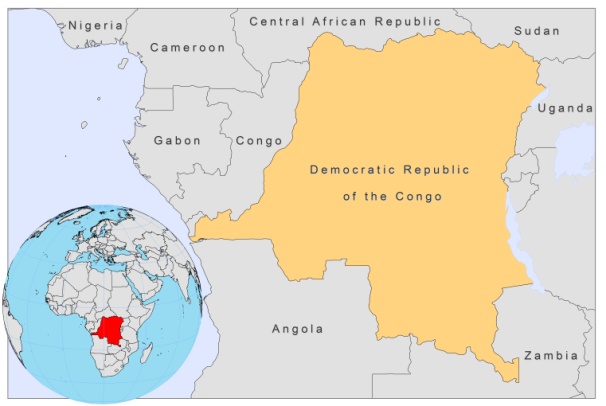
**

**BASIC COUNTRY DATA**

Total Population: 65,965,795

Population 0-14 years: 46%

Rural population: 65%

Population living under USD 1.25 a day: 59.2%

Population living under the national poverty line: 71.3%

Income status: Low income economy

Ranking: Low human development (ranking 187)

Per capita total expenditure on health at average exchange rate (US dollar): 3

Life expectancy at birth (years): 48

Healthy life expectancy at birth (years): 37

**BACKGROUND INFORMATION**

Very few data on prevalence or incidence of leishmaniasis are available. In 1978, the first autochtonous confirmed VL case was reported from the Gemena region in the Guinean savanna, north of the equatorial forest in the northwest of the country [1]. From 1983 to 1988, 6 cases with disseminated cutaneous nodules were reported (at least 3 autochtonous) from the Southeast (Kasai and Shaba), in the savanna belt, south of the forest. No more cases have been documented recently.

**PARASITOLOGICAL INFORMATION**

| ***Leishmania***  **species** | **Clinical form** | **Vector species** | **Reservoirs** |
| --- | --- | --- | --- |
| Unknown | VL, CL | Unknown | Unknown |

**MAPS AND TRENDS**


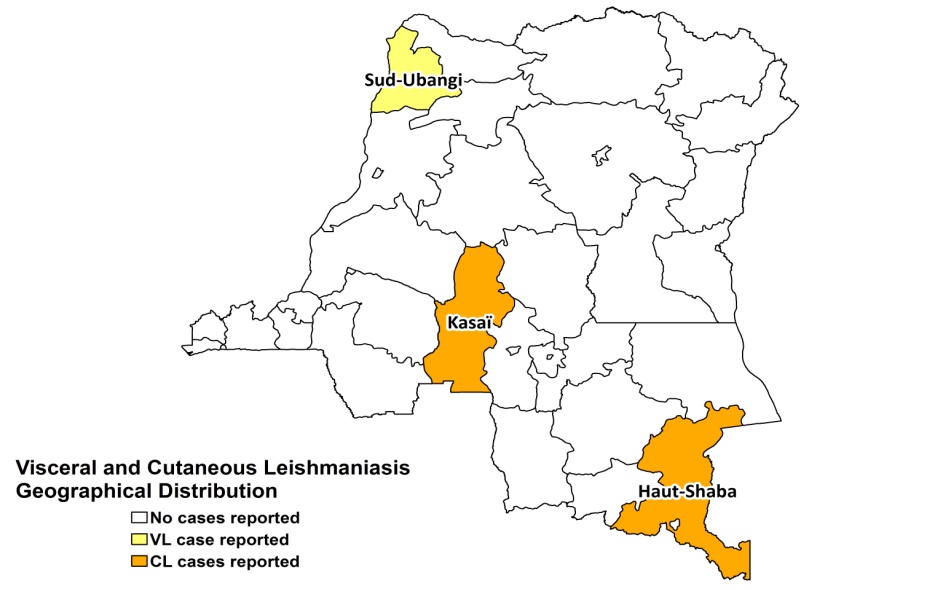


**CONTROL, DIAGNOSIS, TREATMENT & OUTCOMES, ACCESS TO CARE**

No information available.

**ACCESS TO DRUGS**

No medicines for leishmaniasis are registered.

**SOURCES OF INFORMATION**

1. [Gigase P](http://www.ncbi.nlm.nih.gov/pubmed?term=%22Gigase%20P%22%5BAuthor%5D), [Moens F](http://www.ncbi.nlm.nih.gov/pubmed?term=%22Moens%20F%22%5BAuthor%5D), [Van Emelen J](http://www.ncbi.nlm.nih.gov/pubmed?term=%22Van%20Emelen%20J%22%5BAuthor%5D), [Van Marck E](http://www.ncbi.nlm.nih.gov/pubmed?term=%22Van%20Marck%20E%22%5BAuthor%5D), [Van Mullem J](http://www.ncbi.nlm.nih.gov/pubmed?term=%22Van%20Mullem%20J%22%5BAuthor%5D) (1978). Autochtonous visceral leishmaniasis in Zaire. Ann Soc Belg Med Trop 58(3):235-40.
