# Supplementary material for: Leishmaniasis Worldwide and Global Estimates of Its Incidence
Source: PLoS One. 2012 May 31;7(5):e35671. doi: 10.1371/journal.pone.0035671 (PMC3365071; doi:10.1371/journal.pone.0035671)
Supplement: Text S25 — Leishmaniasis Country Profiles, Djibouti. (DOCX) [file pone.0035671.s025.docx]

**DJIBOUTI**

**
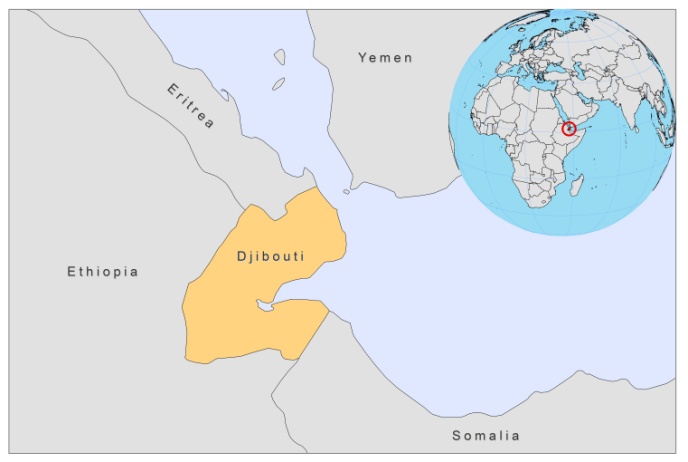
**

**BASIC COUNTRY DATA**

Total Population: 888,716

Population 0-14 years: 36%

Rural population: 12%

Population living under USD 1.25 a day: no data

Population living under the national poverty line: no data

Income status: Lower middle income economy

Ranking: Low human development (ranking 165)

Per capita total expenditure on health at average exchange rate (US dollar): 84

Life expectancy at birth (years): 57

Healthy life expectancy at birth (years): 43

**BACKGROUND**

CL as well as VL,are endemic in Djibouti. The first case of VL was described in 1971 [1]. VL is sporadic in Djibouti; between 1971 and 2003, 59 cases have been reported: five between 1971 and 1976 [2], 10 between 1994 and 1996 [3] and 44 between 1996 and 2000 [4,5]. VL was confirmed to be caused by *L.donovani*, zymodemes MON-268 and MON-287, unique to Djibouti and very similar to MON-37 found in Kenya [5]. MON-268 was identified in a coinfected patient.

CL is also sporadic and only few data are available. Between 1971 and 1976, five cases were reported. Only a few more cases have been described since then, one of which was resistant to antimonials [6].

It seems that VL infections were acquired in the vicinity of Hoi Hoi and Ali Sabieh, in the south, and that CL infections originated in the mountainous northern region Randa [2]. However, it is difficult to establish defined endemic regions as the population is largely nomadic.

Although the vector remains unconfirmed, a countrywide survey revealed extensive presence of *P.alexandri*, but not of *P. orientalis*. *P. alexandri* is a suspected vector for VL, as well as CL, in Djibouti [7].

Ten cases of HIV/*Leishmania* co-infection have been reported.

**PARASITOLOGICAL INFORMATION**

| ***Leishmania* species** | **Clinical form** | **Vector species** | **Reservoirs** |
| --- | --- | --- | --- |
| *L.donovani* | VL | Unknown |  |
| Unknown | CL | Unknown | Unknown |

**CONTROL, DIAGNOSIS & TREATMENT, ACCESS TO CARE , ACCESS TO DRUGS**

No information available.

**SOURCES OF INFORMATION**

1. Courtois, D (1971). Leishmaniose viscérale en territoire français des Afars et des Issas (TFAI). Méd Trop 31: 535–537.

2. Ardouin C, Carteron B, Morvan D, Rodhain F (1978). La leishmaniose en République de Djibouti. Bordeaux Médical 2699–2704.

3. Faure S, Faure E, Massenet D, Masseron T, Fabresse FX et al (1996). La leishmaniose viscérale à Djibouti. A propos de dix cas. In Deuxièmes Journées Médicales de la Corne de l’Afrique. Djibouti: Ministère de la Santé Publique et des Affaires Sociales.

4. Bronstein JA, Galzin M, Hovette P, Simon F, Cellarier G et al (2002). La leishmaniose viscérale à Djibouti: à propos d’une série de 41 patients suivis au CHA Bouffard. Méd Trop 62: 287.

5. [Pratlong F](http://www.ncbi.nlm.nih.gov/pubmed?term=%22Pratlong%20F%22%5BAuthor%5D), [Debord T](http://www.ncbi.nlm.nih.gov/pubmed?term=%22Debord%20T%22%5BAuthor%5D), [Garnotel E](http://www.ncbi.nlm.nih.gov/pubmed?term=%22Garnotel%20E%22%5BAuthor%5D), [Garrabé E](http://www.ncbi.nlm.nih.gov/pubmed?term=%22Garrab%C3%A9%20E%22%5BAuthor%5D), [Marty P](http://www.ncbi.nlm.nih.gov/pubmed?term=%22Marty%20P%22%5BAuthor%5D) et al (2005). First identification of the causative agent of visceral leishmaniasis in Djibouti: Leishmania donovani. Annals of Tropical Medicine & Parasitology 99 (1) 21–25.

6. [Rapp C](http://www.ncbi.nlm.nih.gov/pubmed?term=%22Rapp%20C%22%5BAuthor%5D&itool=EntrezSystem2.PEntrez.Pubmed.Pubmed_ResultsPanel.Pubmed_RVAbstract), [Imbert P](http://www.ncbi.nlm.nih.gov/pubmed?term=%22Imbert%20P%22%5BAuthor%5D&itool=EntrezSystem2.PEntrez.Pubmed.Pubmed_ResultsPanel.Pubmed_RVAbstract), [Darie H](http://www.ncbi.nlm.nih.gov/pubmed?term=%22Darie%20H%22%5BAuthor%5D&itool=EntrezSystem2.PEntrez.Pubmed.Pubmed_ResultsPanel.Pubmed_RVAbstract), [Simon F](http://www.ncbi.nlm.nih.gov/pubmed?term=%22Simon%20F%22%5BAuthor%5D&itool=EntrezSystem2.PEntrez.Pubmed.Pubmed_ResultsPanel.Pubmed_RVAbstract), [Gros P](http://www.ncbi.nlm.nih.gov/pubmed?term=%22Gros%20P%22%5BAuthor%5D&itool=EntrezSystem2.PEntrez.Pubmed.Pubmed_ResultsPanel.Pubmed_RVAbstract) et al (2003). Liposomal amphotericin B treatment of cutaneous leishmaniasis contracted in Djibouti and resistant to meglumine antimoniate. [Bull Soc Pathol Exot.](javascript:AL_get(this,%20'jour',%20'Bull%20Soc%20Pathol%20Exot.');)96(3):209-11.

7. Fryauff DJ, Cope SE, Presley SM, Hanafi HA, Bailly C et al (1995). Sand Flies of the Republic of Djibouti: Ecological Distribution, Seasonal Population Trends, and Identification of Species. J Vect Ecol 20(2):168 -188.
