# Supplementary material for: Leishmaniasis Worldwide and Global Estimates of Its Incidence
Source: PLoS One. 2012 May 31;7(5):e35671. doi: 10.1371/journal.pone.0035671 (PMC3365071; doi:10.1371/journal.pone.0035671)
Supplement: Text S26 — Leishmaniasis Country Profiles, Dominican Republic. (DOCX) [file pone.0035671.s026.docx]

**DOMINICAN REPUBLIC**

**
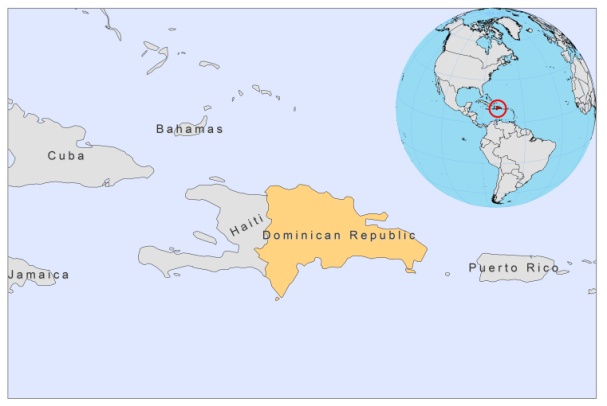
**

**BASIC COUNTRY DATA**

Total Population: 9,927,320

Population 0-14 years: 31%

Rural population: 30%

Population living under USD 1.25 a day: 4.3%

Population living under the national poverty line: 50.5%

Income status: Upper middle income economy

Ranking: Medium human development (ranking 98)

Per capita total expenditure on health at average exchange rate (US dollar): 271

Life expectancy at birth (years): 73

Healthy life expectancy at birth (years): 60

**BACKGROUND INFORMATION**

Since 1975, human cases of diffuse CL have been reported from the provinces of El Seibo, La Altagracia and Sánchez Ramírez in the northeastern part of the country [1]. Subclinical forms are frequent. In 1982, one of the DCL cases also presented a mucosal involvement. *Lu. christophei* and *Lu. cayennensis hispaniola* are present. *Lu. christophei* is highly suspected to be the vector. Suspected reservoirs include the black rat (*Rattus rattus*), a capromid rodent (*Plagiodontia aedium*) and the mongoose (*Horpestas auropunctatus*) [2]. VL is unknown.

No HIV-*Leishmania* co-infection cases have been reported.

**PARASITOLOGICAL INFORMATION**

| ***Leishmania* species** | **Clinical form** | **Vector species** | **Reservoirs** |
| --- | --- | --- | --- |
| Unknown | DCL | Unknown | Unknown |

**MAPS AND TRENDS**

**Cutaneous leishmaniasis**


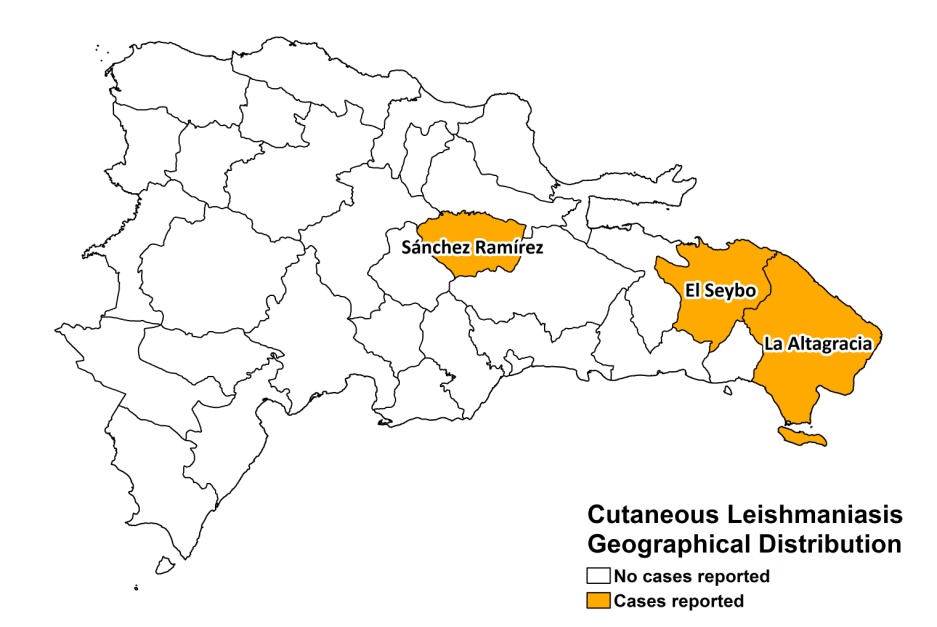


**CONTROL**

The notification of leishmaniasis is not mandatory in the country and there is no national leishmaniasis control program. Case detection is passive. There are no leishmaniasis vector or reservoir control programs.

**DIAGNOSIS, TREATMENT, ACCESS TO CARE**

Not available.

**ACCESS TO DRUGS**

The antimonials Pentostam, GSK (SSG) and Glucantime, Sanofi (meglumine antimoniate), are not registered.

**SOURCES OF INFORMATION**

1. [Zeledón R](http://www.ncbi.nlm.nih.gov/pubmed?term=%22Zeled%C3%B3n%20R%22%5BAuthor%5D) (1992). Leishmaniasis in the Caribbean Islands. A review. Ann N Y Acad Sci 653:154-60.

2. [Johnson RN](http://www.ncbi.nlm.nih.gov/pubmed?term=%22Johnson%20RN%22%5BAuthor%5D), [Young DG](http://www.ncbi.nlm.nih.gov/pubmed?term=%22Young%20DG%22%5BAuthor%5D), [Butler JF](http://www.ncbi.nlm.nih.gov/pubmed?term=%22Butler%20JF%22%5BAuthor%5D), [Bogaert-Diaz H](http://www.ncbi.nlm.nih.gov/pubmed?term=%22Bogaert-Diaz%20H%22%5BAuthor%5D) (1992). Possible determination of the vector and reservoir of leishmaniasis in the Dominican Republic. Am J Trop Med Hyg 46(3):282-7.
