# Supplementary material for: Leishmaniasis Worldwide and Global Estimates of Its Incidence
Source: PLoS One. 2012 May 31;7(5):e35671. doi: 10.1371/journal.pone.0035671 (PMC3365071; doi:10.1371/journal.pone.0035671)
Supplement: Text S27 — Leishmaniasis Country Profiles, Ecuador. (DOCX) [file pone.0035671.s027.docx]

**ECUADOR**

**
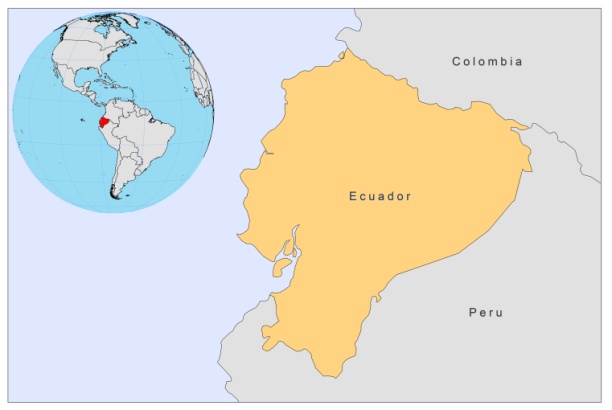
**

**BASIC COUNTRY DATA**

Total Population: 14,464,739

Population 0-14 years: 30%

Rural population: 33%

Population living under USD 1.25 a day: 5.1%

Population living under the national poverty line: 36.0%

Income status: Upper middle income economy

Ranking: High human development (ranking 83)

Per capita total expenditure on health at average exchange rate (US dollar): 255

Life expectancy at birth (years): 75

Healthy life expectancy at birth (years): 62

**BACKGROUND INFORMATION**

CL is a public health problem in Ecuador because of its wide distribution, mainly in rural areas in the regions of Costa, Sierra and Oriente [1]. It is present in 23 out of the 24 provinces. MCL and DCL have been reported. No cases of VL have been found yet.

According to the Ministry of Health’s register, during the period of 1990 to 2006, incidence levels ranged from 4.36 to 20.79 per 100,000 inhabitants, and the disease followed an endemic-epidemic pattern.

The highest number of cases and the highest rates of infection are in the Amazon region, followed by the Andean region (eastern and western slopes of the Andean cordillera, which have a subtropical climate) and the Costa region [2]. An estimated 3,500,000 inhabitants live in regions with a disease transmission risk [3].

Case registration is passive and relies on persons infected with the disease seeking treatment in facilities operated by the Ministry of Health (hospitals, health centers and satellite health centers). This means that the disease is underreported, since it essentially occurs in rural areas and affects a population many of which lack access to health services, especially in the Amazon region [4]. Contrary to what is happening in the Peruvian Andes, *L. peruviana* has not been found in the Andean valleys of Ecuador [5].

**PARASITOLOGICAL INFORMATION**

| ***Leishmania***  **species** | **Clinical**  **form** | **Vector species** | **Reservoirs** |
| --- | --- | --- | --- |
| *L. braziliensis* | ZCL, MCL | Unknown | Unknown |
| *L. panamensis* | ZCL | *Lu. trapidoi,*  *Lu. hartmanni,*  *Lu. gomezi* | *Potus flavus, Tamandua tetradactyla, Sciurus vulgaris, Choloepus didactylus* |
| *L. guyanensis* | ZCL | Unknown | Unknown |
| *L. amazonensis* | ZCL, DCL | *Lu. flaviscutellata* |  |
| *L. mexicana* | ZCL, DCL | *Lu. ayacuchensis* | Unknown |

**MAPS AND TRENDS**

**Cutaneous leishmaniasis**


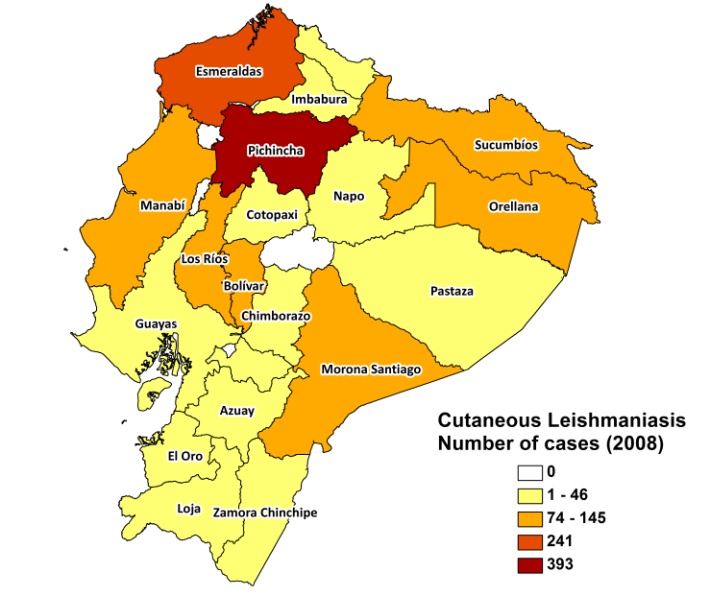


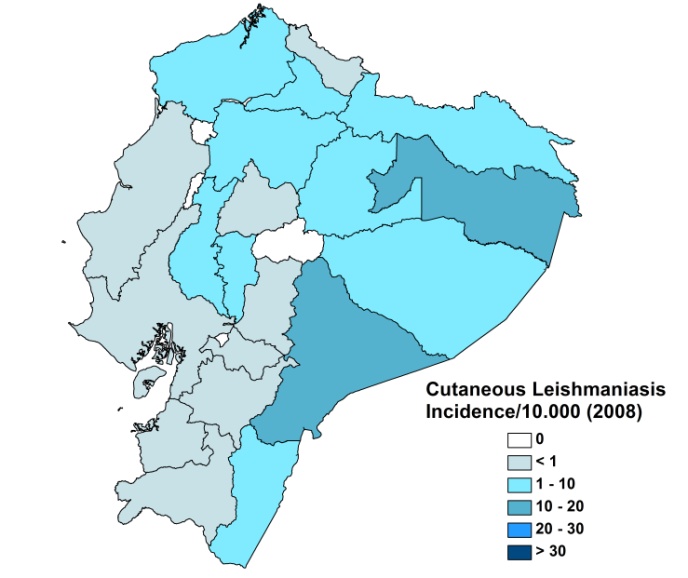


**Cutaneous leishmaniasis trend**

**CONTROL**

Notification of leishmaniasis has been mandatory in Ecuador since 2005. Case registration is passive. There is a vector control program in place that includes bednet distribution and regular insecticide spraying during epidemic outbreaks. Control measures are to be adopted in response to outbreaks or epidemics and involve indoor and peridomestic spraying, carried out by the National Malaria Eradication Service; active case detection and treatment of cases; use of insecticide-treated bed nets, and educational campaigns in coordination with other Ministry of Public Health agencies and the community. However, due to a lack of budget, this program has not been implemented yet, apart from the purchase and distribution of drugs.

**DIAGNOSIS, TREATMENT**

**Diagnosis**

CL: confirmation by microscopic examination of skin lesion sample.

**Treatment:**

CL: antimonials, 10-20 mg Sb^v^/kg/day. Second line: conventional amphotericin B, 0.5-1 mg/kg/day. Third line: pentamidine, 4 mg/kg/day. Miltefosine was introduced as an oral alternative in 2008.

**ACCESS TO CARE**

Confirmed cases receive treatment free of charge in the facilities ran by the Ministry of Health, which purchased sufficient drugs (meglumine antimoniate, Sanofi) for all reported cases in 2007 and 2008. However, access to treatment is incomplete. CL is a problem that mainly affects people living in rural areas, to whom the disease does not pose a serious threat since it is not disabling and only occasionally causes complications. It does not generate much concern in communities (it is rarely epidemic), as the lesions may heal spontaneously, so patients wait a long time before seeking medical attention. Most patients self-medicate or use home remedies before resorting to public health centers. When the need arises, they experience difficulties in accessing public health facilities. Diagnosis and treatment for CL is only offered at advanced health care levels, but patients live in very remote areas with no health facilities and no transport. There is a lack of trained human resources for treating leishmaniasis, and confirmatory laboratory diagnosis is only made in a few establishments (usually one in each province).

The private sector is not used much. About 2% of MCL patients seek care in the private sector.

**ACCESS TO DRUGS**

Meglumine antimoniate and miltefosine (10 and 50 mg tablets) are included in the National Essential Drug List for CL. Drugs for leishmaniasis are not sold in the private sector nor in unregulated drugs markets. Meglumine antimoniate (Glucantime, Sanofi) and miltefosine (Paladin, Canada) are registered.

**SOURCES OF INFORMATION**

- Dr. Lenin Vélez Nieto, SNEM Guayaquil - Ministerio de Salud. *Leishmaniasis en la Región de las Américas. Reunión de coordinadores de Programa Nacional de Leishmaniasis. OPS/OMS. Medellín, Colombia. 4-6 junio 2008*

1. Armijos RX, Weigel MM, Izurieta R, Racines J, Zurita C et al (1997). [The epidemiology of cutaneous leishmaniasis in subtropical Ecuador.](http://www.ncbi.nlm.nih.gov/pubmed/9472299) Trop Med Int Health 2(2):140-52.

2. Calvopiña M, Armijos RX, Hashiguchi Y (2004). Epidemiology of Leishmaniasis in Ecuador: Current Status of Knowledge - A Review. Mem Inst Oswaldo Cruz, Rio de Janeiro 99 (7): 663-672.

3. Calvopiña M, Armijos RX, Marco JD, Uezato H, Kato H et al (2006) [Leishmania isoenzyme polymorphisms in Ecuador: relationships with geographic distribution and clinical presentation.](http://www.ncbi.nlm.nih.gov/pubmed/16968553) BMC Infect Dis 6:139.

4. Weigel MM, Armijos RX, Racines RJ, Zurita C, Izurieta R et al (1994). [Cutaneous leishmaniasis in subtropical Ecuador: popular perceptions, knowledge, and treatment.](http://www.ncbi.nlm.nih.gov/pubmed/8069334) Bull Pan Am Health Organ 28(2):142-55.

5. Hashiguchi Y, Gomez EA, de Coronel VV, Mimori T, Kawabata M et al (1991). [Andean leishmaniasis in Ecuador caused by infection with Leishmania mexicana and L. major-like parasites.](http://www.ncbi.nlm.nih.gov/pubmed/1672799) Am J Trop Med Hyg 44(2):205-17.
