# Supplementary material for: Leishmaniasis Worldwide and Global Estimates of Its Incidence
Source: PLoS One. 2012 May 31;7(5):e35671. doi: 10.1371/journal.pone.0035671 (PMC3365071; doi:10.1371/journal.pone.0035671)
Supplement: Text S28 — Leishmaniasis Country Profiles, Egypt. (DOCX) [file pone.0035671.s028.docx]

**EGYPT**


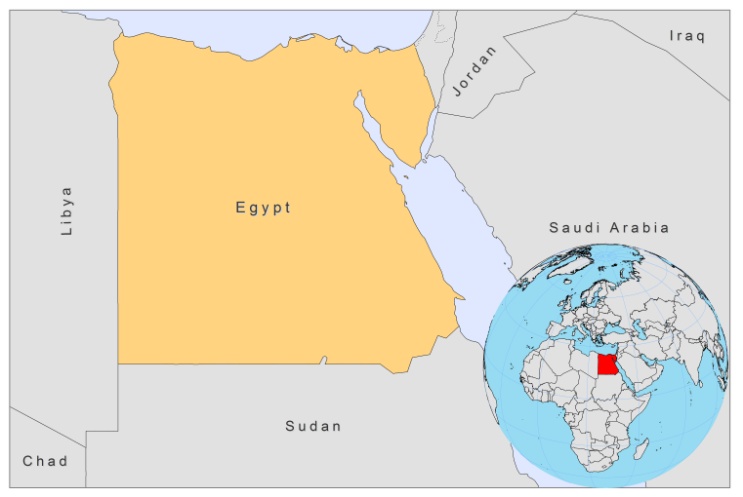


**BASIC COUNTRY DATA**

Total Population: 81,121,077

Population 0-14 years: 32%

Rural population: 57%

Population living under USD 1.25 a day: 2%

Population living under the national poverty line: 22%

Income status: Lower middle income economy

Ranking: Medium human development (ranking 113)

Per capita total expenditure on health at average exchange rate (US dollar): 112

Life expectancy at birth (years): 73

Healthy life expectancy at birth (years): 59

**BACKGROUND INFORMATION**

*Leishmania donovani* DNA was already found in bone marrow samples taken from ancient Egyptian and Nubian mummies that originate from around 4000 BC [1]. Since 1904, rare cases of VL have been reported in Egypt; some were imported, others were probably autochtonous. A focus of VL was discovered in El Agamy, 25 km west of Alexandria in 1982 [2]. The last case there was reported in 2005 and only one more case of VL was reported, in the Suez region in 2008. However, due to a lack of awareness among medical practitioners, VL is suspected to be underreported. Import of cases from Libya and Sudan may occur regularly and go unnoticed [3].

CL has been an increasing problem in Egypt [4]. Known foci are among nomads in North Sinai with 471 reported cases in 2008 [5]. In an active case detection survey 200 new cases were identified, confirming underreporting to a large degree. The reported number of cases is estimated to be 4-5 times lower than the real number of cases. In 2010 (until July only), 133 cases were reported. People at risk are soldiers, laborers, and immunocompromised adults.

There are no reported cases of HIV-*Leishmania* co-infection.

**PARASITOLOGICAL INFORMATION**

| ***Leishmania* species** | **Clinical form** | **Vector species** | **Reservoirs** |
| --- | --- | --- | --- |
| *L infantum* | ZVL | *P. langeroni* | *Canis familiaris* |
| *L major* | ZCL | *P. papatasi* | *Psammomys obesus, Gerbillus sp.* |
| *L. tropica* | CL | unknown |  |

**MAPS AND TRENDS**

**Visceral leishmaniasis**


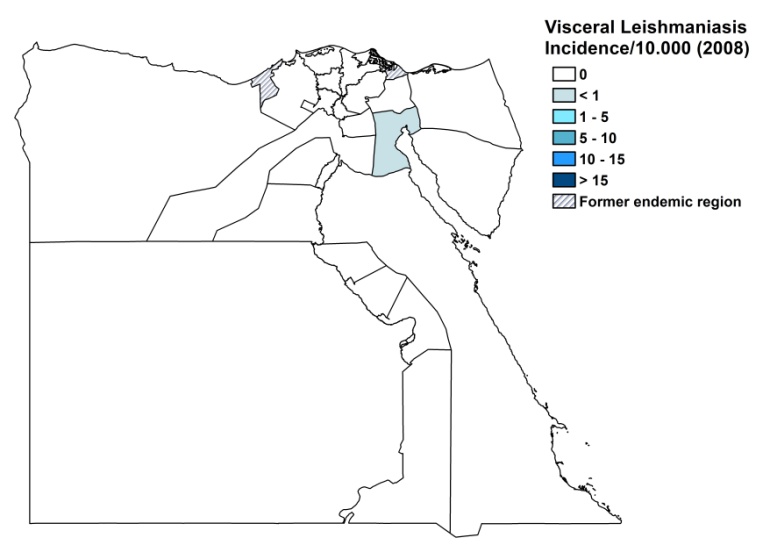


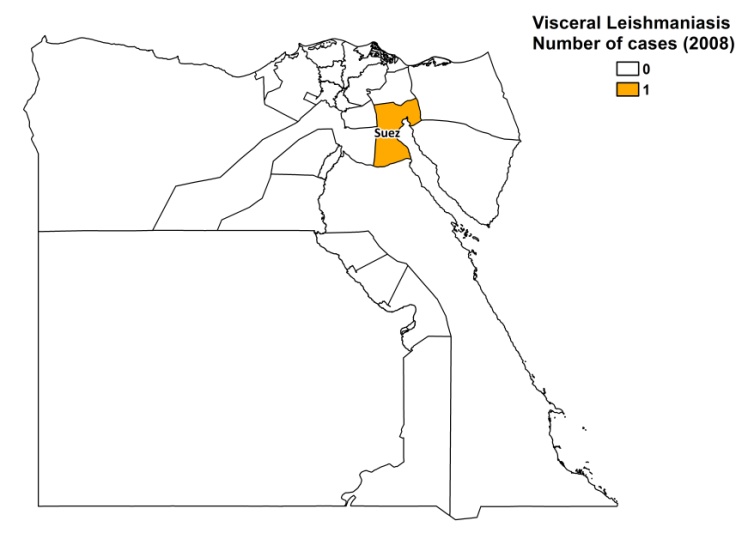


**Cutaneous leishmaniasis**

**
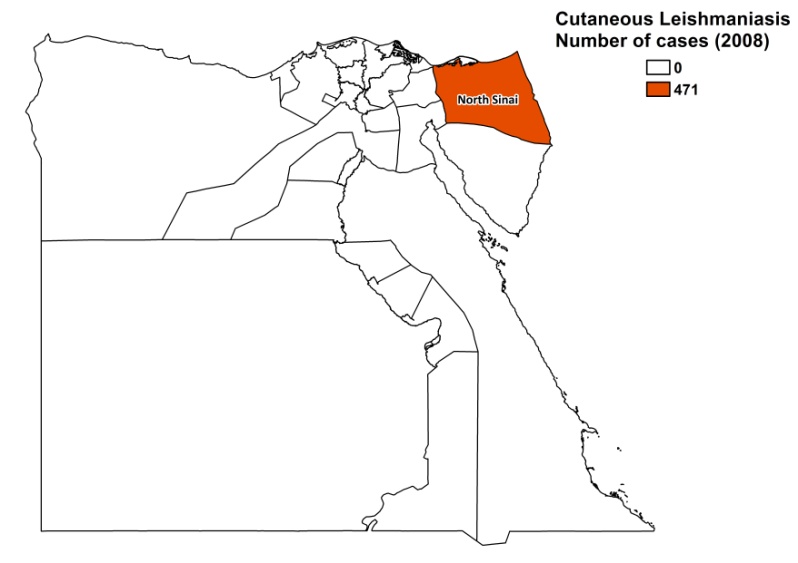

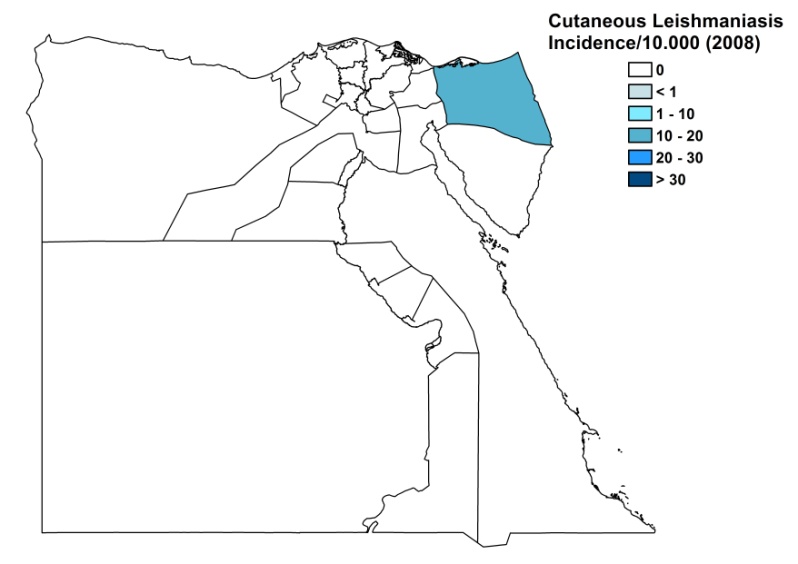
**

**Cutaneous leishmaniasis trend**

**CONTROL**

Notification of leishmaniasis is not mandatory in Egypt. There has been a national leishmaniasis control program in place since 1985, but it is not functioning well due to a lack of financing and political commitment. Active human case detection is regularly performed, but on a limited scale. Insecticide spraying is regularly done, but deemed ineffective. There is a reservoir control program, but it is not specifically targeted for leishmaniasis.

**DIAGNOSIS, TREATMENT**

**Diagnosis**

CL: on clinical picture and exclusion of other infections.

VL: clinical suspicion and confirmation by microscopic examination of bone marrow aspirate.

**Treatment**

CL: antimonials, intralesional or systemic (20 mg Sb^v^/kg/day) with a maximum of 850 mg Sb^5^/kg/day for 20 days, to be repeated after 10 days.

VL: antimonials, 20 mg Sb^v^/kg/day for 20 days, to be repeated after 10 days.

Reported cure rate for VL is 100%. Outcome data are not available for CL treatment.

**ACCESS TO CARE**

Medical care is provided for free in Egypt, with only a nominal fee of 0.4 USD charged as registration fee. CL is diagnosed in health posts and health centers and treated with antimonials, if available. VL is only diagnosed and treated in specialized hospitals. There are no NGOs treating leishmaniasis in Egypt. Drugs for leishmaniasis are not provided by the Ministry of Health. WHO is the only source of drugs; it donated antimonials in 2007 and 2008. In 2008, WHO donated antimonials for the topical treatment of less than 100 patients, with 471 reported CL patients in that same year. Subsequently, in 2008, not all cases could be treated. In lack of treatment, nomads from the North Sinai region extinguish cigarettes on their lesions or use remedies such as vinegar and bleach.

**ACCESS TO DRUGS**

No other drug than sodium stibogluconate is included in the National Essential Drug List for leishmaniasis. Liposomal amphotericin B (AmBisome, Gilead) is registered in Egypt, but no antimonials are registered. Drugs for leishmaniasis are not available at private pharmacies.

**SOURCES OF INFORMATION**

- Dr Samir Mahfouz. Ministry of Health.

1. Kupfer B, Vehreschild J, Cornely O, Kaiser R, Plum G et al (2006). Leishmaniasis in Ancient Egypt and Upper Nubia*.* Emerg Infect Dis 12(10):1616.

2. [Morsy TA](http://www.ncbi.nlm.nih.gov/pubmed?term=%22Morsy%20TA%22%5BAuthor%5D) (1997). Visceral leishmaniasis with special reference to Egypt (review and comment). J Egypt Soc Parasitol 27(2):373-96.

3. WHO, Report of the consultative meeting on cutaneous leishmaniasis. 30 April–2 May 2007; Geneva, World Health Organization, Geneva (2008) WHO/HTM/NTD/IDM/2008.7.

4. [Postigo JA](http://www.ncbi.nlm.nih.gov/pubmed?term=%22Postigo%20JA%22%5BAuthor%5D) (2010). Leishmaniasis in the World Health Organization Eastern Mediterranean Region. Int J Antimicrob Agents 36 Suppl 1:S62-5.

5. Shehata MG, Samy AM, Doha SA, Fahmy AR, Kaldas RM et al (2009). First Report of Leishmania tropica from a Classical Focus of L. major in North-Sinai, Egypt Am J Trop Med Hyg 81(2): 213 - 218.
