# Supplementary material for: Leishmaniasis Worldwide and Global Estimates of Its Incidence
Source: PLoS One. 2012 May 31;7(5):e35671. doi: 10.1371/journal.pone.0035671 (PMC3365071; doi:10.1371/journal.pone.0035671)
Supplement: Text S29 — Leishmaniasis Country Profiles El Salvador. (DOCX) [file pone.0035671.s029.docx]

**EL SALVADOR**

**
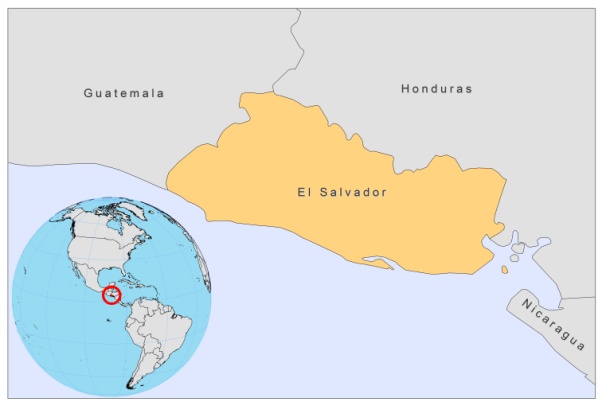
**

**BASIC COUNTRY DATA**

Total Population: 6,192,993

Population 0-14 years: 32%

Rural population: 39%

Population living under USD 1.25 a day: 5.1%

Population living under the national poverty line: 37.8%

Income status: Lower middle income economy

Ranking: Medium human development (ranking 105)

Per capita total expenditure on health at average exchange rate (US dollar): 229

Life expectancy at birth (years): 72

Healthy life expectancy at birth (years): 60

**BACKGROUND INFORMATION**

The leishmaniasis incidence rate in El Salvador is 0.5 cases per 100,000 inhabitants. Most of the cases occur in rural areas. From 1900-1985, only 5 cases of CL were reported, probably related to widespread insecticide spraying on cotton and coffee crops [1]. Afterwards, during the beginning of the civil war, the disease spread significantly. A total of 91 cases were reported from 2003 to 2007. 87 of these were CL and 4 VL, and all VL cases died. 50% of these cases were in children under 10 years of age and 30% between 10 and 19 years. Women account for the majority of cases (70%). There is considerable underreporting of cases and limited knowledge about the disease among health workers and population.

VL is very rare. The first cases of VL were described in 1954 [2]. No HIV/*Leishmania* co-infection has been reported.

**PARASITOLOGICAL INFORMATION**

| ***Leishmania***  **species** | **Clinical form** | **Vector species** | **Reservoirs** |
| --- | --- | --- | --- |
| *L. infantum* | ZVL, CL | *Lu. longipalpis* | *Canis familiaris* |

**MAPS AND TRENDS**

**Cutaneous leishmaniasis**

**
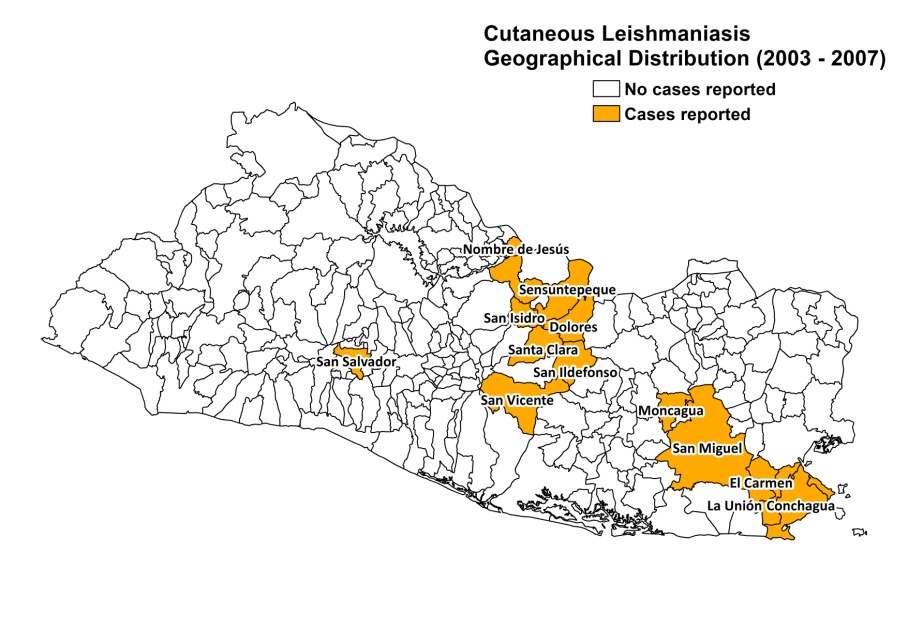
**

**Cutaneous leishmaniasis trend**

**CONTROL**

Notification of leishmaniasis has been mandatory since 1991; the completion of an individual case report form has been mandatory since 2005. Case detection is passive, and there are no established vector or reservoir control programs.

**DIAGNOSIS, TREATMENT**

**Diagnosis:**

**CL:** microscopic examination of skin lesion samples.

**Treatment:**

**CL**: antimonials, 10-20 Sb^v^/kg/day.

**VL**: antimonials, 10-20 Sb^v^/kg/day.

**ACCESS TO CARE**

Drugs are provided by the Ministry of Health, free of charge. Access to treatment is poor. There is an important lack of awareness of CL and VL among health workers and the population. Diagnosis is only possible in a few specialized centres.

**ACCESS TO DRUGS**

Meglumine antimoniate (Glucantime, Sanofi) is registered.

**SOURCES OF INFORMATION**

- Dr Reina Hernández Santamaría. *Leishmaniasis en la Región de las Américas. Reunión de coordinadores de Programa Nacional de Leishmaniasis. OPS/OMS. Medellín, Colombia. 4-6 junio 2008.*

1. Ramires O (1986). Autochtonous cutaneous leishmaniasis in El Salvador. Report of 5 cases. [Med Cutan Ibero Lat Am.](http://www.ncbi.nlm.nih.gov/pubmed/3528714)14(2):91-4.

2. [Guillen Alvarez G](http://www.ncbi.nlm.nih.gov/pubmed?term=%22GUILLEN%20ALVAREZ%20G%22%5BAuthor%5D) (1954). First four cases of kala-azar discovered in El Salvador. [Arch Col Med El Salv.](http://www.ncbi.nlm.nih.gov/pubmed/14362894)7(3):238-45.
