# Supplementary material for: Leishmaniasis Worldwide and Global Estimates of Its Incidence
Source: PLoS One. 2012 May 31;7(5):e35671. doi: 10.1371/journal.pone.0035671 (PMC3365071; doi:10.1371/journal.pone.0035671)
Supplement: Text S30 — Leishmaniasis Country Profiles, Eritrea. (DOCX) [file pone.0035671.s030.docx]

**ERITREA**


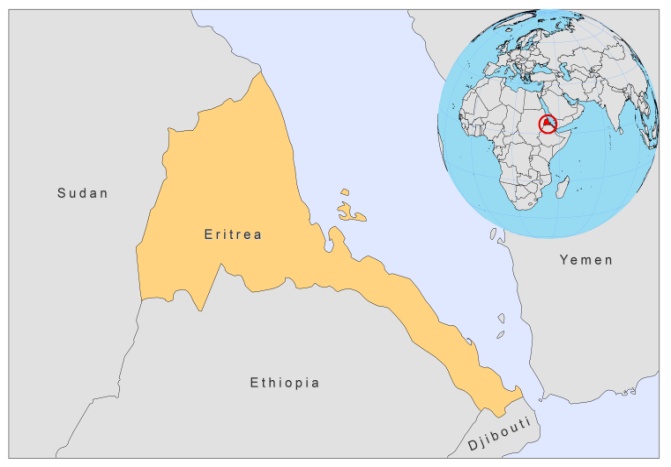


**BASIC COUNTRY DATA**

Total Population: 5,253,676

Population 0-14 years: 42%

Rural population: 78%

Population living under USD 1.25 a day: no data

Population living under the national poverty line: no data

Income status: Low income economy

Ranking: Low human development (ranking 177)

Per capita total expenditure on health at average exchange rate (US dollar): 10

Life expectancy at birth (years): 61

Healthy life expectancy at birth (years): 50

**BACKGROUND INFORMATION**

VL as well as CL has been historically present in Eritrea. Leishmaniasis is distributed throughout the country. VL and CL are not reported as separate clinical manifestations, but the majority of reported cases are visceral. 95% of reported cases are over 5 years old. The usual distribution of VL in the Horn of Africa is in the lowlands (in Eritrea, in the west and south) and for CL in the highlands (in Eritrea, in the north and center), although population movements should be taken into account. It is not known if outbreaks occurred in the past.

A HIV positive Eritrean person was found to be coinfected after moving to the Netherlands [1].

**PARASITOLOGICAL INFORMATION**

| ***Leishmania* species** | **Clinical form** | **Vector species** | **Reservoirs** |
| --- | --- | --- | --- |
| Unknown | | | |

**MAPS AND TRENDS**

**Cutaneous and visceral leishmaniasis**

**
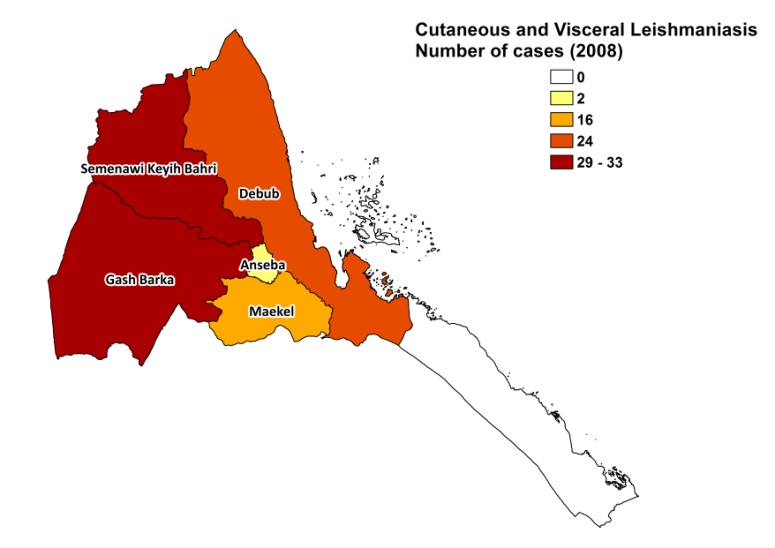

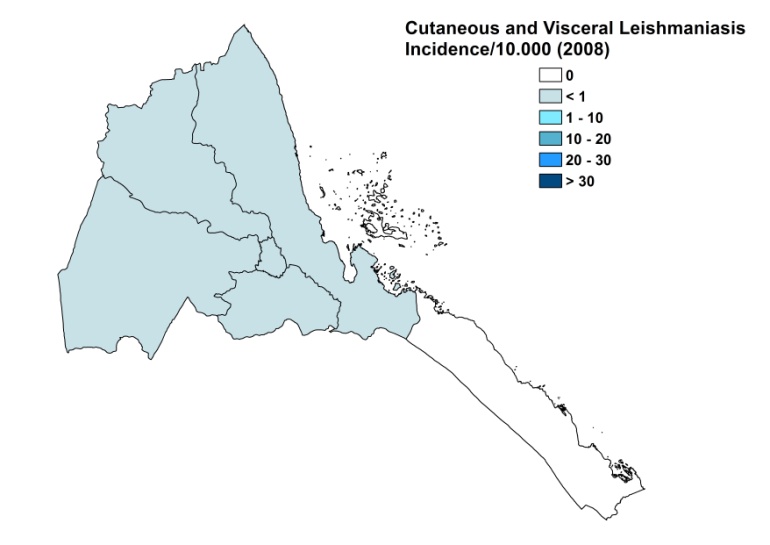
**

**Leishmaniasis trend**

**CONTROL**

The notification of leishmaniasis is mandatory in the country. There is no national leishmaniasis control program, but there is a strategic plan of action for integrated control of neglected tropical diseases (2010-2014), including leishmaniasis. There is no leishmaniasis vector control program, but in the context of the malaria eradication program there is a bednet distribution program and regular indoor insecticide spraying.

**DIAGNOSIS, TREATMENT**

**Diagnosis**

VL: confirmation by microscopic examination of bone marrow or spleen aspirates.

CL: on clinical grounds, confirmation by microscopic examination of skin lesion samples.

**Treatment**

VL: antimonials, 20 mg Sb^v^/kg/day for 30 days.

CL: antimonials, 20 mg Sb^v^/kg/day for 21 days. The national referral hospital in Asmara treated 10 cases in 2 years, all with a good response in follow up after 2-3 months.

**ACCESS TO CARE**

Care for leishmaniasis is not provided for free. Health care in Eritrea is charged according to a cost recovery scheme. Antimonials were present in hospitals in 2007 and 2008, but it is unknown if all patients have access to treatment. Both forms of leishmaniasis are suspected to be underdiagnosed.

**ACCESS TO DRUGS**

Meglumine antimoniate and sodium stibogluconate are included in the National Essential Drug List. No antimonials are registered in Eritrea.

**SOURCES OF INFORMATION**

- Dr. Yohannes Ghebrat. Disease Prevention and Control; WHO Eritrea. *Lymphatic filariasis, schistosomiasis, soil transmitted helminthiases and leismaniasis assessment. Internal report, September 2009.*

1. Meenken C, Agtmael MA van, Kate RW ten, Horn GJ van den (2004). Fulminant ocular leishmaniasis in an HIV-1 positive patient. AIDS 18:1485-1486.
