# Supplementary material for: Leishmaniasis Worldwide and Global Estimates of Its Incidence
Source: PLoS One. 2012 May 31;7(5):e35671. doi: 10.1371/journal.pone.0035671 (PMC3365071; doi:10.1371/journal.pone.0035671)
Supplement: Text S31 — Leishmaniasis Country Profiles, Ethiopia. (DOCX) [file pone.0035671.s031.docx]

**ETHIOPIA**


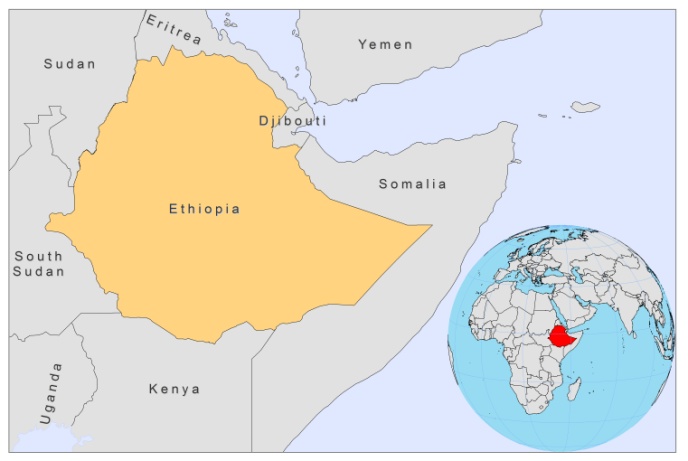


**BASIC COUNTRY DATA**

Total Population: 82,949,541

Population 0-14 years: 41%

Rural population: 82%

Population living under USD 1.25 a day: 39%

Population living under the national poverty line: no data

Income status: Low income economy

Ranking: Low human development (ranking 174)

Per capita total expenditure on health at average exchange rate (US dollar): 15

Life expectancy at birth (years): 61

Healthy life expectancy at birth (years): 41

**BACKGROUND INFORMATION**

Both CL and VL are growing health problems in Ethiopia, with endemic areas that are continually spreading. The first case of VL in Ethiopia was documented in 1942 in the southern parts of the country. Now, there are an estimated 2,000 to 4,500 cases yearly, with endemic areas in the lowlands of the northwest, central, south and southwestern parts of the country. In the north, the vector is associated with Red Acacia and Balanites trees, in the south with termite hills. VL affects mainly children and young adults (the mean age is 23).

CL has been well known since 1913 and is endemic in most regions [1]. It is a highly neglected disease. There are an estimated 50,000 cases yearly, but only 450 cases were reported in 2008. There are three clinical forms of CL in Ethiopia: localized CL, mucosal leishmaniasis and diffuse cutaneous leishmaniasis (DCL), all mainly caused by *L. aethiopica*. The first case of DCL was reported in 1960; currently the incidence of this form is high in the highlands of Ethiopia [2]. CL is most common in children, with the highest prevalence occurring between 10 and 15 years of age.

Several outbreaks occurred in Ethiopia in the last 5 years. Between 2005 and 2008, an outbreak of VL occurred in Amhara Region (Libo Kemkem), where VL had not been reported before, with 2,500 cases and initially a very high mortality [3]. The disease was probably imported from the endemic areas in Humera and Metema [4]. An outbreak of 300 CL cases took place in Silti district between 2003 and 2005 [5]. In 2010, 30 cases of VL formed the beginning of an outbreak in a formerly non-endemic area in Tigray (Shiraro district), and 40 VL cases more, mainly children, were diagnosed in East Imey, a district in Somali region, previously non endemic.

The incidence of HIV-*Leishmania* coinfection was 23% in 2008, far higher than anywhere else in the world. The real burden is likely to be higher, as only 17% of VL cases are screened for HIV in some facilities. The affected populations are mainly very poor male seasonal migrant workers that travel in the harvesting season from non endemic highlands to the cotton, sesame and sorghum fields of Humera and Metema on the Sudanese border [6,7]. In Humera, the proportion of VL patients that was coinfected was 40% in 2006 [6].

PKDL seems to occur in varying rates within different regions and patient populations. PKDL is reported in 2.2% of patients, but this figure does not represent the real number of cases as PKDL grade 1 cases are not treated, and consequently not reported. In coinfected patients, PKDL is seen in 15-40% of patients.

**PARASITOLOGICAL INFORMATION**

| ***Leishmania* species** | **Clinical form** | **Vector species** | **Reservoirs** |
| --- | --- | --- | --- |
| *L. aethiopica* | ZCL, DCL, ML | *P. longipes,*  *P. pedifer*,*  *P. sergenti* | *Procavia capensis, Heterohyrax brucei* |
| *L. major* | ZCL | *P. dubosqui* | *Arvicanthis niloticus* |
| *L. tropica* | CL | *P. sergenti,*  *P. saeveus* |  |
| *L. donovani* | VL, PKDL | *P. orientalis,*  *P. martini, h. celiae* |  |

**MAPS AND TRENDS**

**
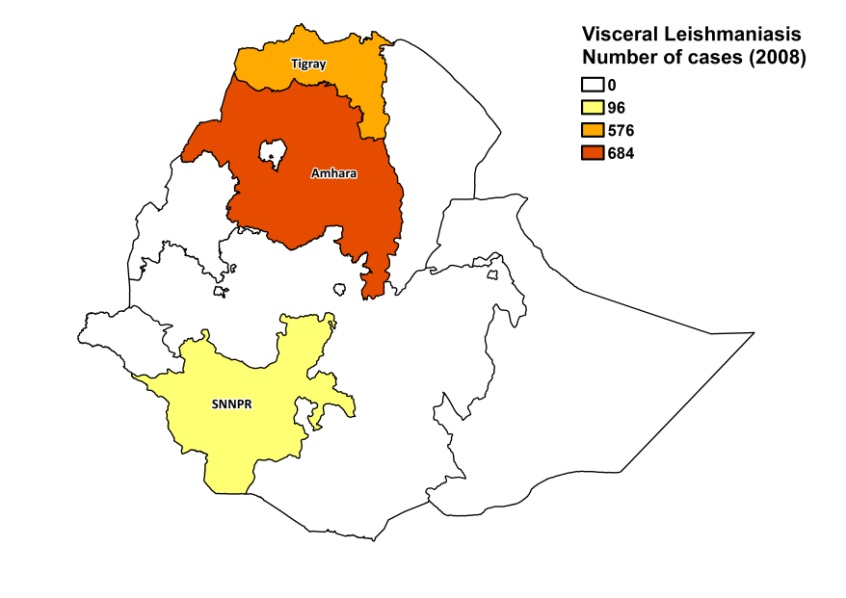
Visceral leishmaniasis**

**
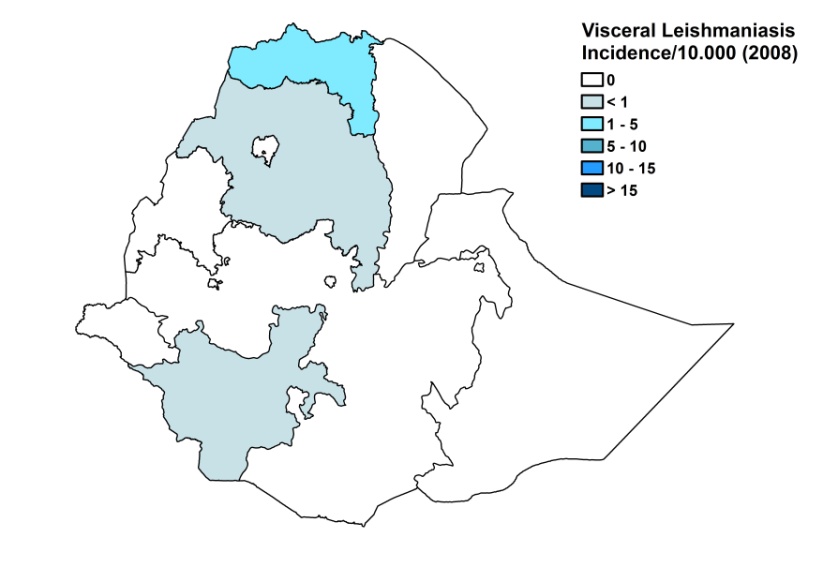
**

**Cutaneous leishmaniasis**

**
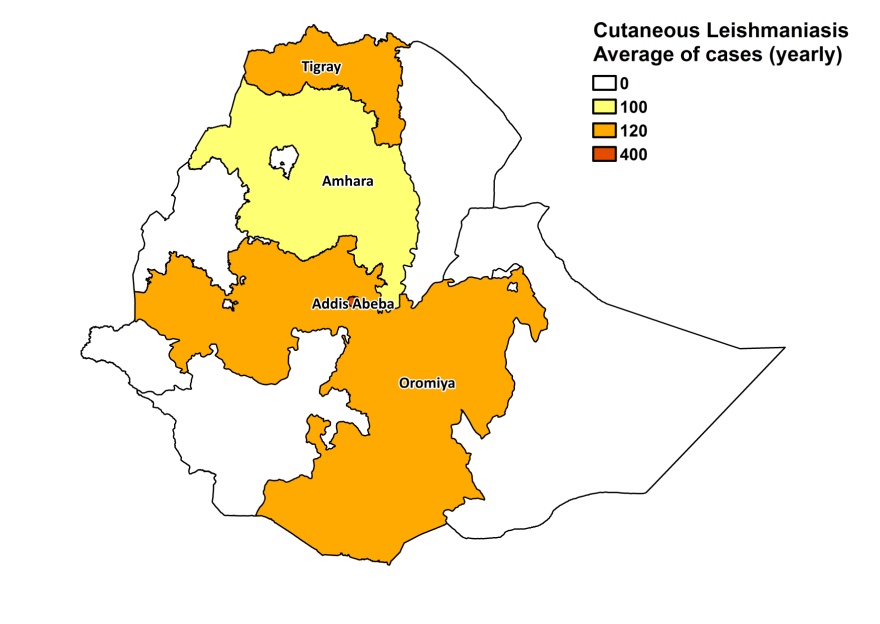
**

**Visceral leishmaniasis trend**

**Cutaneous leishmaniasis trend**

Data not available.

**CONTROL**

The notification of leishmaniasis is mandatory and there has been a national leishmaniasis control program since 2006. Active human case detection for VL is regularly performed by mobile teams in remote endemic areas. There is no leishmaniasis vector control program. Bednet distribution and insecticide spraying take place in the context of malaria control.

**DIAGNOSIS, TREATMENT**

**Diagnosis:**

VL: confirmation by rK39 ICT.

CL: clinical, confirmation with microscopic examination of skin lesion sample.

**Treatment:**

VL: antimonials, 20 mg Sb^v^/kg/day for 30 days. Cure rate is 90%. Relapse is seen in 4% of cases. Fatality rate is 5-10%. Second line treatment: amphotericin B at a dose of 1 mg/kg every other day for 30 days or liposomal amphotericin B 4 mg/kg/day for 5-10 days. Miltefosine (100 mg/day, 28 days) is used as 3rd line treatment, mainly for coinfected patients. For coinfected patients, liposomal amphotericin B for 4 mg/kg/day for 5-10 days is first line treatment, with a cure rate of 60%. PKDL is treated with a 2-month course of antimonials (20 mg Sb^v^ /kg/day).

CL: cryotherapy (with liquid nitrogen); antimonials, intralesional or systemic (20 mg Sb^v^/kg/day for 20 days) for severe cases. Cryotherapy has a cure rate of 93%. Failure rate was 28% for antimonials. Pentamidine is used as second line treatment with a cure rate of 88%.

**ACCESS TO CARE**

Care for leishmaniasis is not provided for free. Patients do not have to pay for VL drugs and rK39 tests, but other tests are not free of charge. The registration fee for health facilities is 0.25-2 USD (with or without referral letter). Hospitalization costs 12 USD. In total, a VL patient usually has to spend 20 USD to complete a full treatment in hospital. Patients are often too poor to pay for this and also suffer major economic loss when spending time away from home. Many patients live in very remote areas with no health facilities and no transport, or they cannot afford transport if available. There is also a lack of awareness of the disease. These issues are currently addressed by mobile teams that are active in a number of remote endemic areas.

In Ethiopia, MSF treated considerable numbers of VL patients. In 2007, a total of 2,500 patients received treatment and in 2008, a total of 1,500 patients. For both years, the number of patients was estimated at 4,500, meaning that over half of patients had problematic access to treatment.

The Ministry of Health provided conventional amphotericin B and generic sodium stibogluconate in 2007 and 2008. As the amounts were insufficient, WHO donated considerable amounts of antimonials.

There are only very few health centers that diagnose and treat CL. It is usually treated by traditional healers that use local heat application and local plants.

**ACCESS TO DRUGS**

Sodium stibogluconate, liposomal amphotericin B, pentamidine and miltefosine (50 mg tablets) are included in the National Essential Drug List. Drugs for leishmaniasis are not available in pharmacies and unregulated drug markets. No drugs for leishmaniasis are registered in Ethiopia.

**SOURCES OF INFORMATION**

- Drs Daniel Argaw and Merce Herrero: Disease Prevention and Control, WHO Ethiopia. *Consultative Meeting on The Control of Leishmaniasis in the African Region WHO/AFRO Addis Ababa, 23-25 Feb 2010.*
- Dr Abraham Aseffa, Armauer Hansen Research Institute/ALERT. *Consultative Meeting on The Control of Leishmaniasis in the African Region WHO/AFRO Addis Ababa, 23-25 Feb 2010.*

1. Ashford RW, Bray MA, Hutchinson MP, Bray RS (1973). The epidemiology of cutaneous leishmaniasis in Ethiopia. Trans R Soc Trop Med Hyg 67(4):568-602.

2. Bryceson AD (1970). Diffuse cutaneous leishmaniasis in Ethiopia. II. Treatment. Trans R Soc Trop Med Hyg 64:369-379.

3. Herrero M, Orfanos G, Argaw D, Mulugeta A, Aparicio P et al (2009). Natural History of a Visceral Leishmaniasis Outbreak in Highland Ethiopia. Am J Trop Med Hyg 81(3):373-7.

4. Bashaye S, Nombela N, Argaw D, Mulugeta A, Herrero M et al (2009). [Risk factors for visceral leishmaniasis in a new epidemic site in Amhara Region, Ethiopia.](http://www.ncbi.nlm.nih.gov/pubmed/19556563) Am J Trop Med Hyg 81(1):34-9.

5. Negera E, Gadissa E, Yamuah L, Engers H, Hussein J et al (2008). Outbreak of cutaneous leishmaniasis in Silti woreda, Ethiopia: risk factor assessment and causative agent identification. Trans R Soc Trop Med Hyg 102(9):883-890.

6. Alvar J, Aparicio P, Aseffa A, Den Boer M, Cañavate C et al (2008). The Relationship between Leishmaniasis and AIDS: the Second 10 Years. Clin Microb Rev 21(2):334–359.

7. Horst R ter, Collin SM, Ritmeijer K, Bogale A, Davidson RN (2008). Concordant HIV Infection and Visceral Leishmaniasis in Ethiopia: The Inﬂuence of Antiretroviral Treatment and Other Factors on Outcome. Clin Infect Dis 46:1702–9.
