# Supplementary material for: Leishmaniasis Worldwide and Global Estimates of Its Incidence
Source: PLoS One. 2012 May 31;7(5):e35671. doi: 10.1371/journal.pone.0035671 (PMC3365071; doi:10.1371/journal.pone.0035671)
Supplement: Text S32 — Leishmaniasis Country Profiles, France. (DOCX) [file pone.0035671.s032.docx]

**FRANCE**


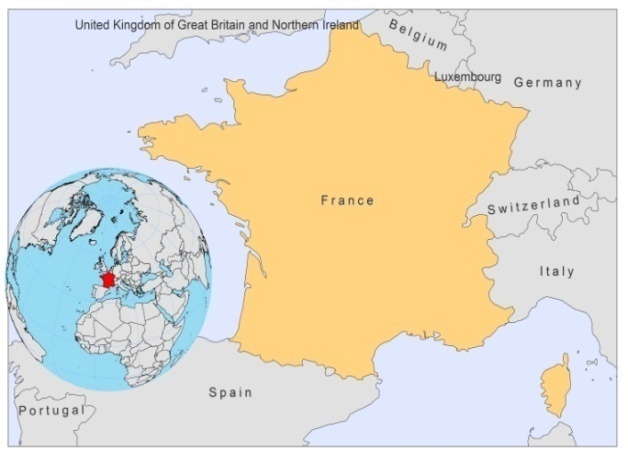


**BASIC COUNTRY DATA**

Total Population: 64,876,618

Population 0-14 years: 18%

Rural population: 22%

Population living under USD 1.25 a day: no data

Population living under the national poverty line: no data

Income status: High income economy: OECD

Ranking: Very high human development (ranking 20)

Per capita total expenditure on health at average exchange rate (US dollar): 4,798

Life expectancy at birth (years): 81

Healthy life expectancy at birth (years): 72

**BACKGROUND INFORMATION**

Human VL caused by *L infantum* has been present in France since the 1920s. Humans are only occasional hosts. Dogs are the main reservoir. In southern France, the seroprevalence in dogs varies between 10 to 20% and molecular methods detected 66% of asymptomatic dogs [1,2]. There is no program for the control of canine leishmaniasis.

Human disease may manifest as VL or CL. A total of 267 cases were reported between 1999 and 2010 (mean of 22 cases per year), 232 of which were VL, 29 CL and 6 mucosal leishmaniasis. The majority of cases occur in immunocompromised adults, although VL in young children is observed. Cases of *Leishmania*/HIV co-infection are diagnosed in the Mediterranean area and Paris, and represent 38% of all VL cases. In the Mediterranean foci, VL predominates, representing 97-99% of all cases. Areas at risk are the Cevennes, the Provence, the Cote d’Azur, the Pyrenées Orientales and Corsica. CL is less frequent than VL, with only 24 cases reported from southern France between 1999-2008. In the Cevennes, 20% of the cases are CL and 78% VL, whereas in the Pyrenées Orientales, 78% of the cases are CL and 22% VL. In the Cote d’Azur and the Provence, 97%, resp. 99% of cases are VL. CL, however, often remains undiagnosed.

Both VL and CL are imported diseases. Between 2006 and 2010, 31 cases of VL and 248 cases of CL were imported.

**PARASITOLOGICAL INFORMATION**

| ***Leishmania* species** | **Clinical form** | **Vector species** | **Reservoirs** |
| --- | --- | --- | --- |
| *L. infantum* | ZVL, CL | *P. perniciosus,*  *P. ariasi* | *Canis familiaris,*  *Vulpes* *vulpes* |

**MAPS AND TRENDS**

**
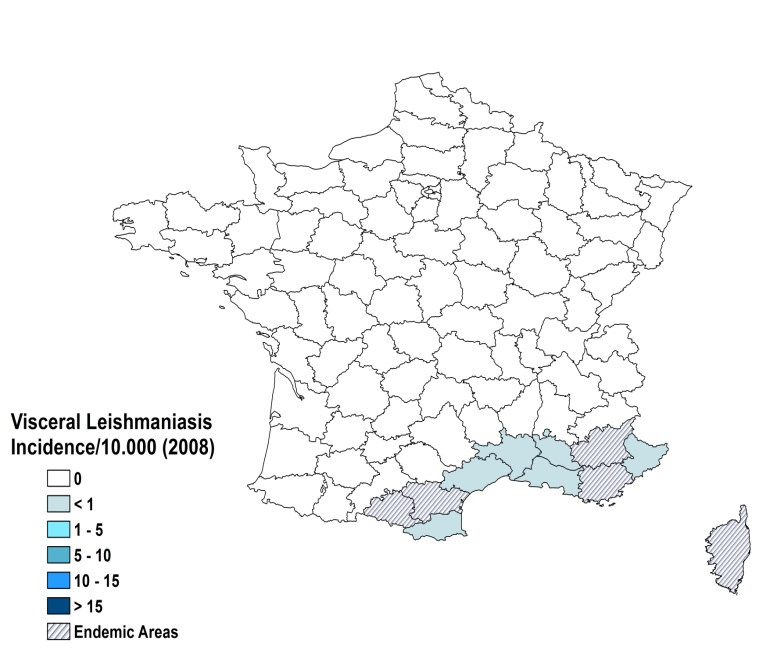
Visceral leishmaniasis**

**
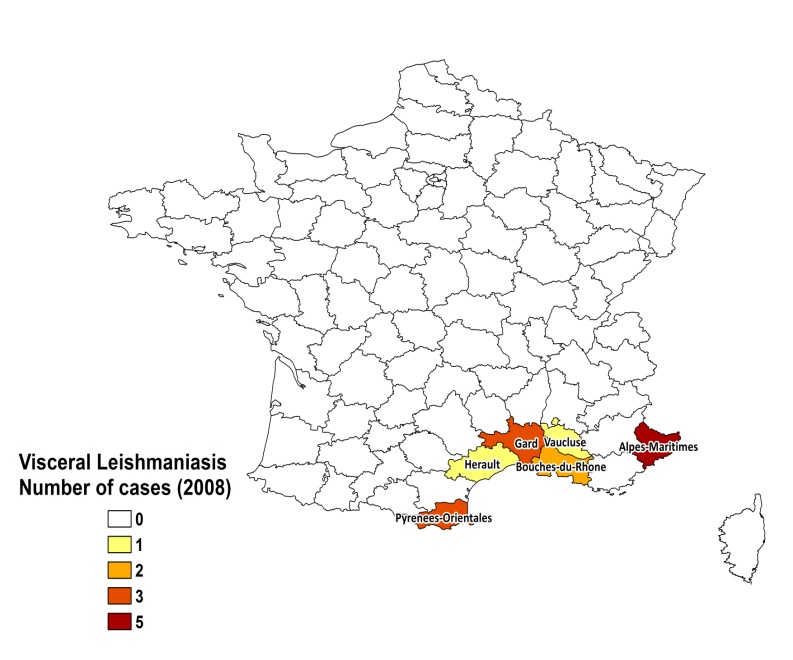
**

**Cutaneous leishmaniasis**


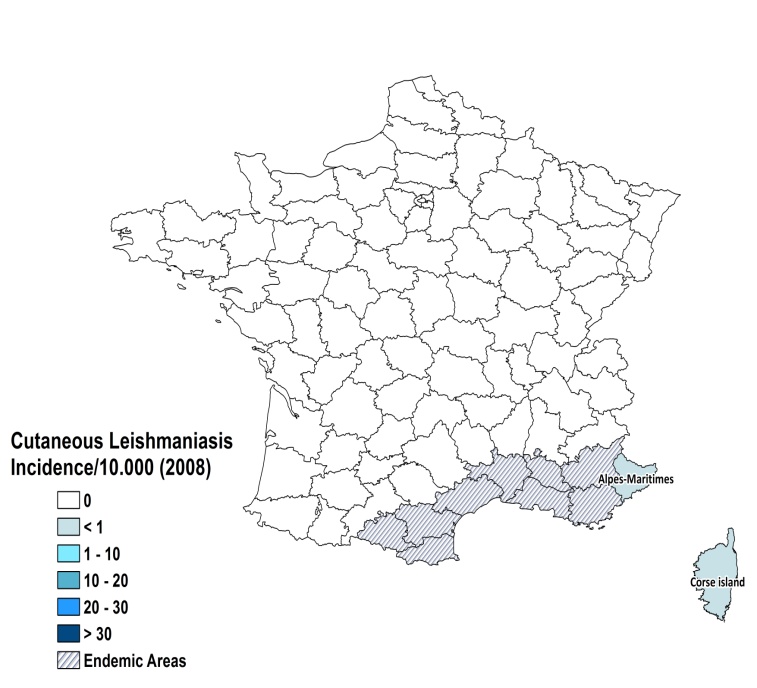

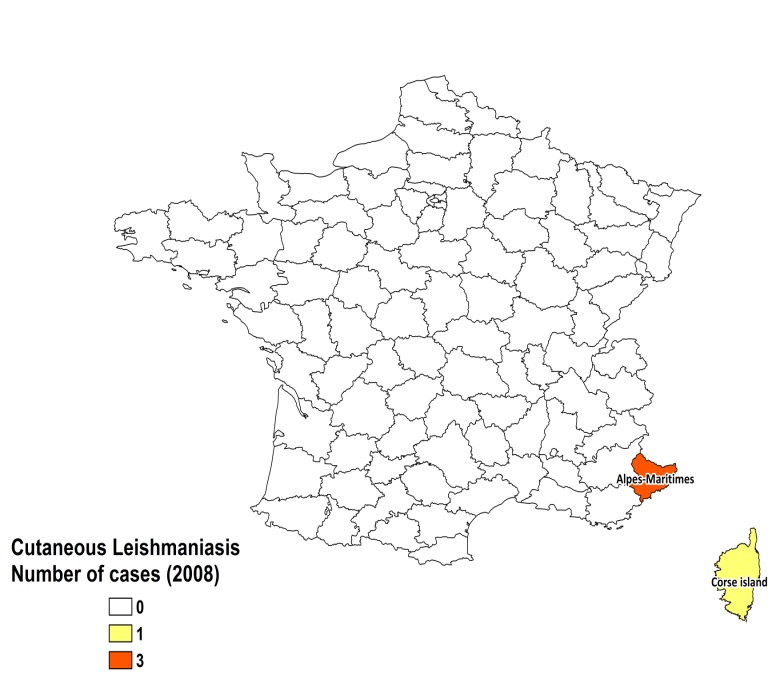


**Visceral leishmaniasis trend**

**Cutaneous leishmaniasis cases**

| **1999** | **2000** | **2001** | **2002** | **2003** | **2004** | **2005** | **2006** | **2007** | **2008** | **2009** | **2010** |
| --- | --- | --- | --- | --- | --- | --- | --- | --- | --- | --- | --- |
| 2 | 0 | 4 | 5 | 3 | 0 | 0 | 3 | 3 | 4 | 2 | 8 |

**CONTROL**

The notification of leishmaniasis is not mandatory and there is no national leishmaniasis control program. Case detection is passive. There is no leishmaniasis vector control program, no bednet distribution program and no insecticide spraying. There is no leishmaniasis reservoir control program, but serological surveys of dogs are regularly performed.

**DIAGNOSIS, TREATMENT**

**Diagnosis:**

VL: parasitological, immunological methods and PCR are used.

CL: parasitological, sometimes PCR.

**Treatment**

VL: First line treatment is with liposomal amphotericin B, 3 mg/kg/day for 5 + 1 day. Cure rate is > 95%. Second line treatment is with miltefosine 2.5 mg/kg/day for 28 days and antimonials, 20 mg Sb^v^/kg/day for 28 days.

CL: antimonials, 20 mg Sb^v^/kg/day for 28 days.

**ACCESS TO CARE**

Care for leishmaniasis is provided for free. Diagnosis and treatment are only offered at advanced health care levels. All patients are thought to have access to care in France.

**ACCESS TO DRUGS**

Meglumine antimoniate, liposomal amphotericin B and miltefosine are included in the National Essential Drug List for VL. For CL, meglumine antimoniate and pentamidine are included. Glucantime (Sanofi) is registered.

**SOURCES OF INFORMATION**

1. [Lachaud L](http://www.ncbi.nlm.nih.gov/pubmed?term=%22Lachaud%20L%22%5BAuthor%5D), [Chabbert E](http://www.ncbi.nlm.nih.gov/pubmed?term=%22Chabbert%20E%22%5BAuthor%5D), [Dubessay P](http://www.ncbi.nlm.nih.gov/pubmed?term=%22Dubessay%20P%22%5BAuthor%5D), [Dereure J](http://www.ncbi.nlm.nih.gov/pubmed?term=%22Dereure%20J%22%5BAuthor%5D), [Lamothe J](http://www.ncbi.nlm.nih.gov/pubmed?term=%22Lamothe%20J%22%5BAuthor%5D) Sanofi (2002). Value of two PCR methods for the diagnosis of canine visceral leishmaniasis and the detection of asymptomatic carriers. Parasitology 125:197-207.

2. [Marty P](http://www.ncbi.nlm.nih.gov/pubmed?term=%22Marty%20P%22%5BAuthor%5D), [Izri A](http://www.ncbi.nlm.nih.gov/pubmed?term=%22Izri%20A%22%5BAuthor%5D), [Ozon C](http://www.ncbi.nlm.nih.gov/pubmed?term=%22Ozon%20C%22%5BAuthor%5D), [Haas P](http://www.ncbi.nlm.nih.gov/pubmed?term=%22Haas%20P%22%5BAuthor%5D), [Rosenthal E](http://www.ncbi.nlm.nih.gov/pubmed?term=%22Rosenthal%20E%22%5BAuthor%5D), Sanofi (2007). A century of leishmaniasis in Alpes-Maritimes, France. Ann Trop Med Parasitol 101(7):563-74.
