# Supplementary material for: Leishmaniasis Worldwide and Global Estimates of Its Incidence
Source: PLoS One. 2012 May 31;7(5):e35671. doi: 10.1371/journal.pone.0035671 (PMC3365071; doi:10.1371/journal.pone.0035671)
Supplement: Text S33 — Leishmaniasis Country Profiles, French Guyana. (DOCX) [file pone.0035671.s033.docx]

**FRENCH GUYANA**


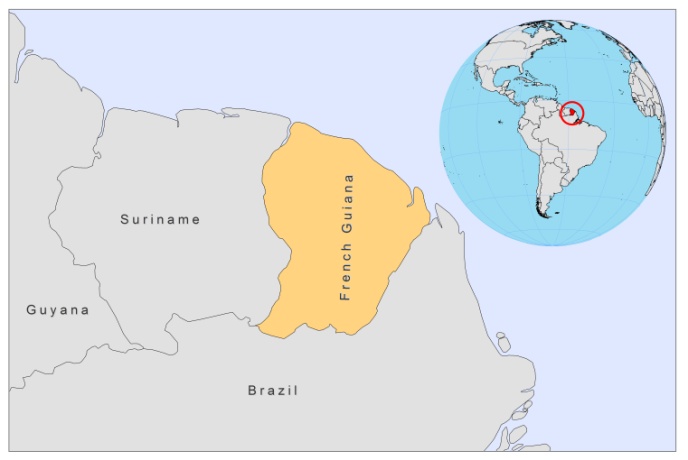


**BASIC DEPARTMENT DATA**

**No data**

**BACKGROUND INFORMATION**

Cutaneous leishmaniasis, called "Pian Bois" or "bush yaws", is a very old endemic disease [1]. Transmission is zoonotic and related to occupational activities in the primary rain forest. The first parasitologically confirmed case was described in 1943 [2], and the parasite described as *Leishmania tropica guyanensis*. Since then, more species have been identified. The prevalence of the various *Leishmania* spp. was 84.4% for *L. (V.) guyanensis*, 8.0% for *L. (V.) braziliensis*, 5.0% for *L. (L.) amazonensis*, and 2.6% for *L. (V.) lainsoni* [3]. Between 1943 and 1951, 83 cases were diagnosed, only half parasitologically confirmed [1]. Since 1977, the number of cases has increased and infections occur regularly among young male adults who work in or near the forest (84.2% of cases) as shows a survey taken from 1981-1987 [4]. 206,000 people are thought to be at risk.

In French Guyana, there are at least two distinct cycles of leishmaniasis, both occurring in the rain forest but at two different altitudinal levels (canopy and ground level) [1]. Most transmission occurs during periods of low rainfall. Establishment of human settlements has given rise to intradomiciliary transmission.

**PARASITOLOGICAL INFORMATION**

| ***Leishmania* species** | **Clinical form** | **Vector species** | **Reservoirs** |
| --- | --- | --- | --- |
| *L. guyanensis* | ZCL | *Lu. umbratilis* | *Choleopus didactylus,*  *Proechimys sp.,*  *Didelphis marsupialis* |
| *L. braziliensis* | ZCL, MCL | *Lu. wellcomei,*  *Lu. intermedia* | unknown |
| *L. amazonensis* | ZCL | *Lu flaviscutellata* | *Proechimys sp.* |
| *L. naiffi* | ZCL | Unknown | unknown |
| *L. lainsoni* | ZCL | unknown | unknown |

**TREND**

**Cutaneous leishmaniasis**

**CONTROL**

Notification of leishmaniasis is mandatory.

**DIAGNOSIS, TREATMENT**

**Diagnosis**:

CL: confirmation by microscopic examination of skin lesion sample.

**Treatment**

CL: Pentamidine.

**ACCESS TO CARE**

Care is provided by public hospitals where diagnosis is possible.

**ACCESS TO DRUGS**

The antimonials Glucantime (meglumine antimoniate, Sanofi) and Pentostam (SSG, GSK) are not registered. Treatments are provided free of charge.

**SOURCES OF INFORMATION**

- Dr Laurence Lachaud, Centre Hospitalier Caremeau, Nîmes. *Leishmaniasis in the European Region, a WHO consultative intercountry meeting, Istanbul, Turkey, 17–19 November 2009.*

1. Dedet JP (1990). Cutaneous leishmaniasis in French Guiana: A review. Am J Trop Med Hyg 43(1): 25-28.

2. Floch H (1943). Rapport sur le fonctionnement technique de l'Institut Pasteur de la Guyane francaise et du territoire de l'Inini pendant l'annee 1943. Inst Pasteur Guyanefrse Terr mini 75:79.

3. Simona S, Verona V, Carmea B (2010). Leishmania spp. identification by polymerase chain reaction–restriction fragment length polymorphism analysis and its applications in French Guiana. [Diagn Microbiol Infect Dis.](http://www.ncbi.nlm.nih.gov/pubmed?term=Leishmania%20spp.%20identification%20by%20polymerase%20chain%20reaction%E2%80%93restriction%20fragment%20length%20polymorphism%20analysis%20and%20its%20applications%20in%20French%20Guiana%E2%98%86)66(2):175-80.

4. Dedet JP, Pradinaud R, Gay F (1989). Epidemiological aspects of human cutaneous leishmaniasis in French Guiana. Trans Roy Soc Trop Med Hyg 83 (5): 613-5.
