# Supplementary material for: Leishmaniasis Worldwide and Global Estimates of Its Incidence
Source: PLoS One. 2012 May 31;7(5):e35671. doi: 10.1371/journal.pone.0035671 (PMC3365071; doi:10.1371/journal.pone.0035671)
Supplement: Text S34 — Leishmaniasis Country Profiles, Gambia. (DOCX) [file pone.0035671.s034.docx]

**GAMBIA**


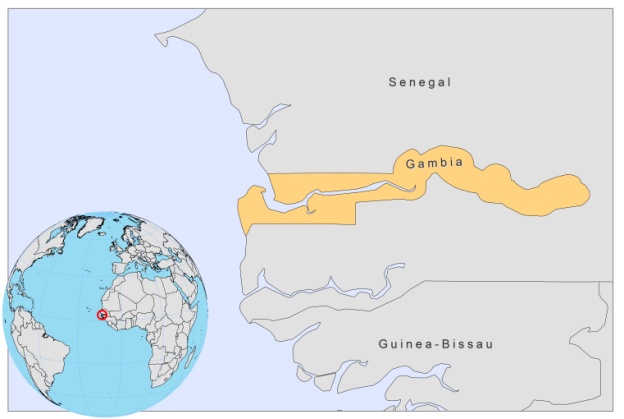


**BASIC COUNTRY DATA**

Total Population: 1,728,394

Population 0-14 years: 44%

Rural population: 42%

Population living under USD 1.25 a day: no data

Population living under the national poverty line: no data

Income status: Low income economy

Ranking: Low human development (ranking 168)

Per capita total expenditure on health at average exchange rate (US dollar): 26

Life expectancy at birth (years): 58

Healthy life expectancy at birth (years): 50

**BACKGROUND INFORMATION**

Leishmaniasis is uncommon in Gambia. No recent cases of either VL or CL have been reported. Only 3 human cases of VL have been described in 1949, 1980 and 1982 [1,2]. In 1980, a positive dog was found in the same compound as the human case [3]. This strongly suggests that dogs could be a reservoir of this infection in this area.

For CL, only 10 cases have been reported, 3 before 1980, one in 1980, and 6 in 1982 [1]. No further cases of VL and CL have been documented. As Gambia is part of a proposed CL endemicity belt running across West Africa, cases may occur regularly but remain unreported. *P. duboscqi*, the proven vector in Senegal, has also been found in the Gambia region [1].

**PARASITOLOGICAL INFORMATION**

| ***Leishmania* species** | **Clinical form** | **Vector species** | **Reservoirs** |
| --- | --- | --- | --- |
| *L. infantum* | ZVL | Unknown | *Canis familiaris* |

**MAPS AND TRENDS, CONTROL, DIAGNOSIS, TREATMENT, ACCESS TO CARE, ACCESS TO DRUGS**

No antimonials are registered.

**SOURCES OF INFORMATION**

1. Desjeux P (1991). Information on the epidemiology and control of the leishmaniases by country or territory. WHO/LEISH/91.30.

2. [Greenwood BM](http://www.ncbi.nlm.nih.gov/pubmed?term=%22Greenwood%20BM%22%5BAuthor%5D), [Ajdukiewicz AB](http://www.ncbi.nlm.nih.gov/pubmed?term=%22Ajdukiewicz%20AB%22%5BAuthor%5D), [Conteh S](http://www.ncbi.nlm.nih.gov/pubmed?term=%22Conteh%20S%22%5BAuthor%5D), [Hagan P](http://www.ncbi.nlm.nih.gov/pubmed?term=%22Hagan%20P%22%5BAuthor%5D), [Mabey DC](http://www.ncbi.nlm.nih.gov/pubmed?term=%22Mabey%20DC%22%5BAuthor%5D) [et](http://www.ncbi.nlm.nih.gov/pubmed?term=%22Panton%20LJ%22%5BAuthor%5D) al (1984). Leishmaniasis in The Gambia. 3. Is its incidence increasing? [Trans R Soc Trop Med Hyg.](javascript:AL_get(this,%20'jour',%20'Trans%20R%20Soc%20Trop%20Med%20Hyg.');)78(3):407-9.

3. Desjeux P, Bryan JH, Martin-Saxton P (1983). Leishmaniasis in The Gambia. 2. A study of possible vectors and animal reservoirs, with the first report of a case of canine leishmaniasis in The Gambia. Trans R Soc Trop Med Hyg 77(2):143-8.
