# Supplementary material for: Leishmaniasis Worldwide and Global Estimates of Its Incidence
Source: PLoS One. 2012 May 31;7(5):e35671. doi: 10.1371/journal.pone.0035671 (PMC3365071; doi:10.1371/journal.pone.0035671)
Supplement: Text S35 — Leishmaniasis Country Profiles, Georgia. (DOCX) [file pone.0035671.s035.docx]

**GEORGIA**

**
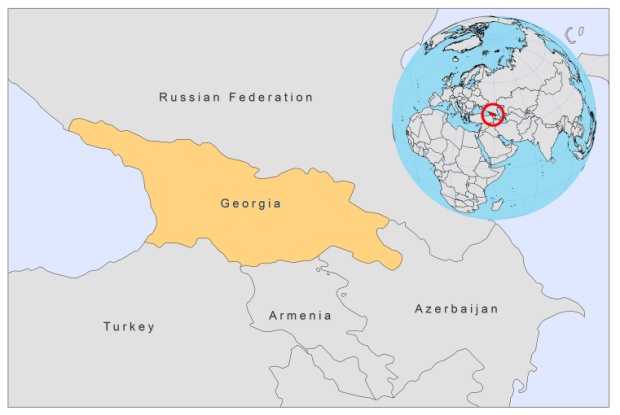
**

**BASIC COUNTRY DATA**

Total Population: 4,452,800

Population 0-14 years: 17%

Rural population: 47%

Population living under 1.25 USD a day: 14.7%

Population living under the national poverty line: 23.6%

Income status: Lower middle income economy

Ranking: High human development (ranking 95)

Per capita total expenditure on health at average exchange rate (US dollar): 256

Life expectancy at birth (years): 73

Healthy life expectancy at birth (years): 64

**BACKGROUND INFORMATION:**

VL by *L infantum* was first reported in Georgia in 1913. 1,355 local and 15 imported cases of VL were registered in Georgia during the period 1928-1999. All cases were registered in 6 cities and 164 villages, mainly in the east of the country, in South Kartly and Kahetia districts. In the following years, patients began registering in Marneul and Bolnis districts of Tbilisi [1]. The number of cases has been increasing since 1996 (from 20 cases in 1996 to 182 in 2008) and has also been spreading to new areas. Between 2004 and 2008, 10 cases occurred in west Georgia, a previously non endemic area [2]. The main endemic area is between Tbilisi and the Armenian border, with many cases occurring in the capital, Tbilisi, which is an active focus with a high prevalence of human and canine cases. Most cases are recorded in children [3]. There is, however, also a relatively high number of adult cases, indicating that the disease seems to re-emerge from an endemic to an epidemic situation.

Two cases of *Leishmania*/HIV co-infection were diagnosed in 2008; both of them died.

Cutaneous leishmaniasis is less frequent. 125 CL cases were registered in the period 1928-1964, of which 110 (88.0%) occurred in Tbilisi and villages situated in the western part of the Kura river valley. After a long interval with no registered cases, new cases of CL started to appear. Mandatory registration of CL started in 2001. From 2001 until 2007, 1-5 cases of CL were reported yearly, which increased to 12 cases in 2008-2009, 8 of them in Tbilisi. Both CL and VL are underreported, due to their relatively recent re-emergence and a consequent lack of awareness and the lack of a training program for medical doctors.

**PARASITOLOGICAL INFORMATION**

| ***Leishmania* species** | **Clinical form** | **Vector species** | **Reservoirs** |
| --- | --- | --- | --- |
| *L. infantum* | ZVL | *P. kandelakii, P. balcanicus,*  *P. halepensi* | *Canis familiaris* |
| *L. major* | CL | unknown |  |

**MAPS AND TRENDS**

**Visceral leishmaniasis**


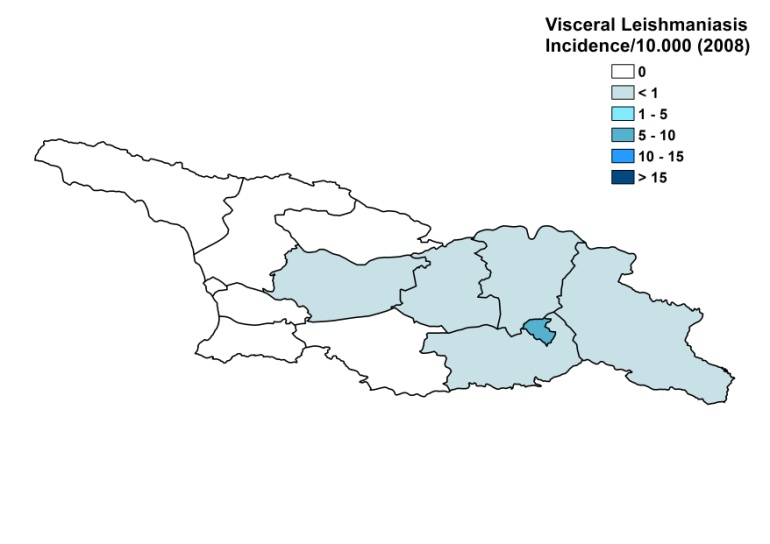

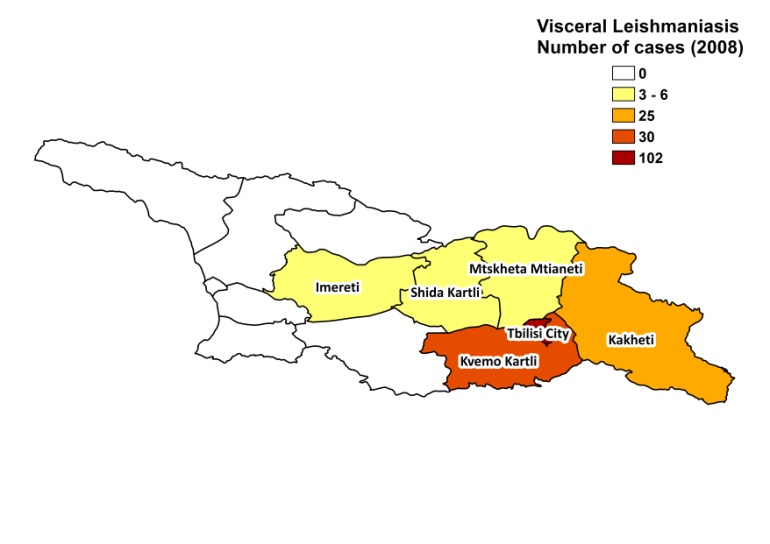


**Cutaneous leishmaniasis**


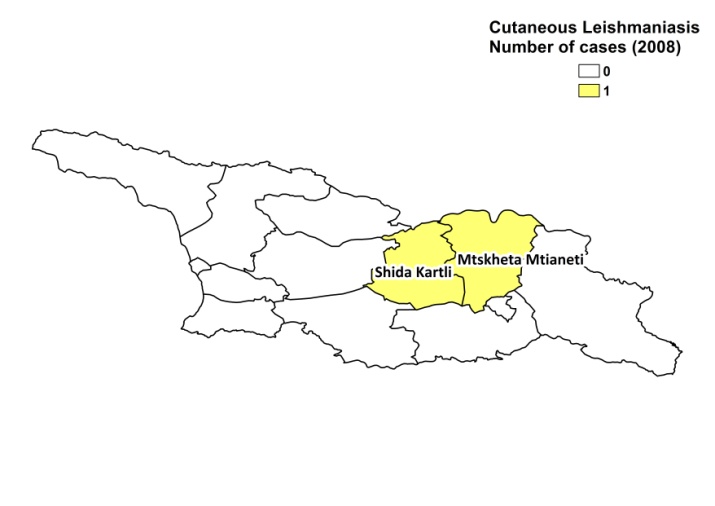


**Visceral leishmaniasis trend**

**Cutaneous leishmaniasis trend**

**CONTROL**

The notification of leishmaniasis is mandatory in the country. There is no national leishmaniasis control program. There is no leishmaniasis vector control program and no bednet distribution program. Insecticide spraying is not done and there is no leishmaniasis reservoir control program.

**DIAGNOSIS, TREATMENT**

**Diagnosis**

CL: on clinical grounds, in rare cases confirmation with microscopic examination of skin lesion sample.

VL: microscopic examination of bone marrow aspirate.

**Treatment**

VL: antimonials, 20 mg Sb^v^/kg/day for 12-20 days. Cure rate is 96%, with 2% relapses and a 2% fatality rate.

**ACCESS TO CARE**

Care for leishmaniasis is not provided for free. Meglumine antimoniate (Glucantime, Sanofi) must be purchased in pharmacies. Patients between 0-18 years old receive 80% funding from the government for the treatment costs, those over 18 years receive 50%, leading to total treatment costs of resp. 142 and 356 USD. Despite these high costs, patients are thought to have access to care in Georgia.

VL diagnosis can only be performed in a specialized center in Tbilisi. There is a lack of awareness of the disease among doctors and patients. A review revealed that the period between manifestation of the first clinical signs and admission to treatment varied from 1 week to 1 year (6.8+/-0,6 weeks on average) between 2000-2005.

The majority of VL cases is treated in one hospital only. As the duration of treatment is shorter than recommended by WHO, relapses, mostly after 3-4 months of treatment, occur regularly. In 1996–1999, 18 (5.9%) of treated patients suffered relapse. During this period, 9 patients died, as diagnosis was made too late and they suffered concomitant diseases.

.

**ACCESS TO DRUGS**

Glucantime (Sanofi) is registered in Georgia and sold in pharmacies for 4 USD per 5 ml vial. Meglumine antimoniate is included in the National Essential Drug List.

**SOURCES OF INFORMATION**

- Dr Merab Iosava, National Center for Disease Control and Public Health, Tbilisi. *Leishmaniasis in the European Region, a WHO consultative intercountry meeting, Istanbul, Turkey, 17–19 November 2009.*

1. Chubabria GA, Zenanshvili OP (2002). Modern peculiarities of VL in Georgia. Med Parazitol (Mosk) 2: 27-30.

2. [Zenanishvili OP](http://www.ncbi.nlm.nih.gov/pubmed?term=%22Zenanishvili%20OP%22%5BAuthor%5D), [Bakashvili LZ](http://www.ncbi.nlm.nih.gov/pubmed?term=%22Bakashvili%20LZ%22%5BAuthor%5D), [Pagava EK](http://www.ncbi.nlm.nih.gov/pubmed?term=%22Pagava%20EK%22%5BAuthor%5D), [Mandzhagaladze MR](http://www.ncbi.nlm.nih.gov/pubmed?term=%22Mandzhagaladze%20MR%22%5BAuthor%5D), [Pagava KI](http://www.ncbi.nlm.nih.gov/pubmed?term=%22Pagava%20KI%22%5BAuthor%5D) (2005). Visceral leishmaniasis: clinical and epidemiological features among children and adolescents in Georgia. [Georgian Med News.](javascript:AL_get(this,%20'jour',%20'Georgian%20%0d%0aMed%20News.');)129:85-7.

3. Zenaishvili O, Gugushvili G, Chubabria G, Manjgaladze M, Kokaia N (2009). New data on epidemiology of visceral leishmaniasis in Georgia. Georgian Med News 172-173:76-80.
