# Supplementary material for: Leishmaniasis Worldwide and Global Estimates of Its Incidence
Source: PLoS One. 2012 May 31;7(5):e35671. doi: 10.1371/journal.pone.0035671 (PMC3365071; doi:10.1371/journal.pone.0035671)
Supplement: Text S36 — Leishmaniasis Country Profiles, Ghana. (DOCX) [file pone.0035671.s036.docx]

**GHANA**

**
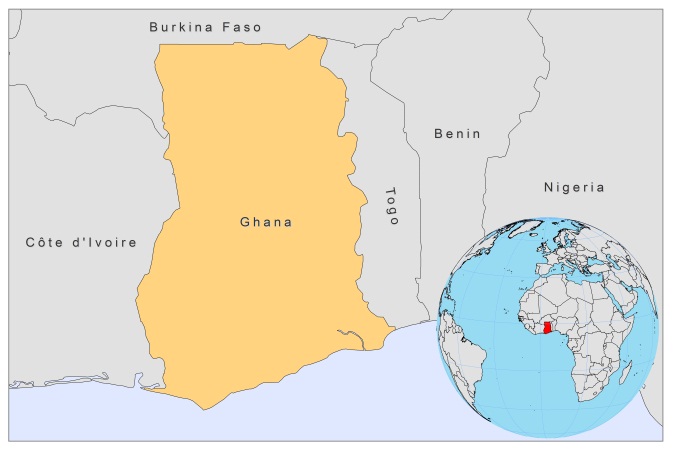
**

**BASIC COUNTRY DATA**

Total Population: 24,391,823

Population 0-14 years: 39%

Rural population: 49%

Population living under USD 1.25 a day: 30%

Population living under the national poverty line: 28.5%

Income status: Lower middle income economy

Ranking: Medium human development (ranking 135)

Per capita total expenditure on health at average exchange rate (US dollar): 53

Life expectancy at birth (years): 63

Healthy life expectancy at birth (years): 50

**BACKGROUND INFORMATION**

Ghana has three ecological zones: an arid northern Savanna, a forest middle belt and a coastal savanna zone. Epidemiologically, the northern zone lies within the leishmaniasis belt in Africa, but no cases have been reported from this area. Most case reports are from a focal outbreak in a moist semi-deciduous forest area, outside the leishmaniasis belt [1].

The first cases of CL in Ghana were reported in the Ho district in 1999-2001, when health centers in two sub districts registered cases of chronic skin ulcers [1]. A survey of towns in the Ho district, during 2002, identified suspected lesions in 12.2–32.3% of local school children [1]. The disease became known in the area under the name ‘Agbamekanu’, which means ‘a gift from a visitor or somebody who had travelled and just came back’. In neighboring Togo, there were no reported cases, but CL was frequently observed in Burkina Faso in the same period [2]. In 2002, an active case search found 2,348 infected individuals in the Ho district, and in 2003, an outbreak of over 6,000 cases was reported in this same district [1]. After that, the case load decreased to 105 cases in 2004 and 14 cases in 2005. Reported cases remained very low and decreased to zero in 2008 and 2009. It is unknown why the case load decreased to this extent. A possible explanation is that, as no treatment is available in Ghana, case detection by health workers and self-reporting declined. The self-healing nature of the disease and the epidemic pattern found in West-African countries with an increase in frequency over a few years, followed by a drastic reduction in incidence, may also have played a role, as well as an insecticide spraying campaign that took place after the outbreak [2].

During the outbreak in 2003, entomological research was performed and the reservoir host was searched for identification, but vector nor reservoir could be discriminated [2].

No cases of VL have been reported in Ghana.

One case of HIV/CL co-infection has been reported in 2005. The patient was lost to follow up [3].

**PARASITOLOGICAL INFORMATION**

| ***Leishmania* species** | **Clinical form** | **Vector species** | **Reservoirs** |
| --- | --- | --- | --- |
| *L. major* | CL | *P. duboscqi* | Unknown |

**MAPS AND TRENDS**

**Cutaneous leishmaniasis**

**
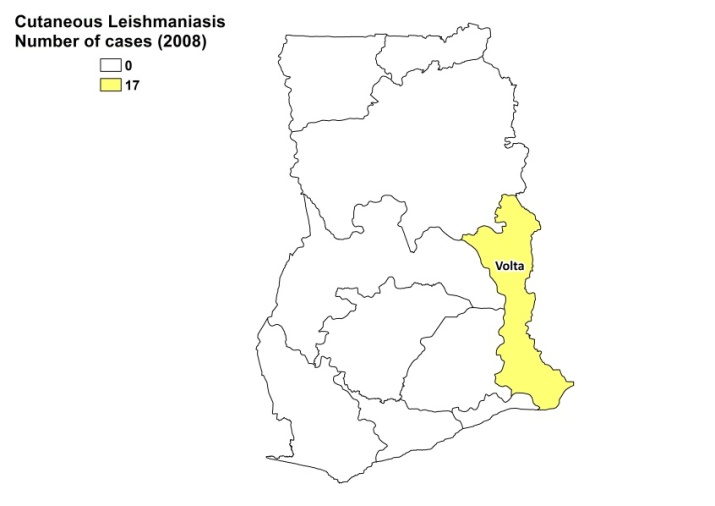
**

**Cutaneous leishmaniasis trend**

**CONTROL**

The notification of leishmaniasis is mandatory in the country. There is no national leishmaniasis control program. However, training of health workers and community-based volunteers in order to improve case detection, reporting and prevention has taken place. Insecticide spraying was performed in the endemic communities of Ho, Hohoe and Kpando districts, in the Volta Region, after the outbreak in 2003. Case detection is passive. There is no leishmaniasis reservoir control program.

**DIAGNOSIS, TREATMENT**

Not available.

**ACCESS TO CARE**

No treatment for leishmaniasis is available in Ghana. Self-medication was frequently observed during outbreaks.

**ACCESS TO DRUGS**

No drugs for leishmaniasis are registered in Ghana. Pentamidine is included in the national Essential Drug list for leishmaniasis.

**SOURCES OF INFORMATION**

• Dr Atsu Seake-Kwawu. Ghana Health Service*. Consultative Meeting on the Control of Leishmaniasis in the African Region. WHO/AFRO Addis Ababa, 23-25 Feb 2010.*

1. [Fryauff DJ](http://www.ncbi.nlm.nih.gov/pubmed?term=%22Fryauff%20DJ%22%5BAuthor%5D), [Hanafi HA](http://www.ncbi.nlm.nih.gov/pubmed?term=%22Hanafi%20HA%22%5BAuthor%5D), [Klena JD](http://www.ncbi.nlm.nih.gov/pubmed?term=%22Klena%20JD%22%5BAuthor%5D), [Hoel DF](http://www.ncbi.nlm.nih.gov/pubmed?term=%22Hoel%20DF%22%5BAuthor%5D), [Appawu M](http://www.ncbi.nlm.nih.gov/pubmed?term=%22Appawu%20M%22%5BAuthor%5D) et al (2006). Short Report: ITS-1 DNA Sequence confirmation of Leishmania Major as a cause of cutaneous leishmaniasis from an outbreak focus in the Ho district, Southeastern Ghana. Am J Trop Med Hyg 75(3):502–504.

2. Boakye DA, Wilson MD, Kweku M (2005). A Review of Leishmaniasis in West Africa. Ghana Med J 39 (3): 94-97.

3. Lartey M, Adusei L, Hanson-Nortey L, Addy JH (2006). Coinfection of cutaneous leishmaniasis and HIV infection. Ghana Med J 40 (3): 110-112.
