# Supplementary material for: Leishmaniasis Worldwide and Global Estimates of Its Incidence
Source: PLoS One. 2012 May 31;7(5):e35671. doi: 10.1371/journal.pone.0035671 (PMC3365071; doi:10.1371/journal.pone.0035671)
Supplement: Text S37 — Leishmaniasis Country Profiles, Greece. (DOCX) [file pone.0035671.s037.docx]

**GREECE**

**
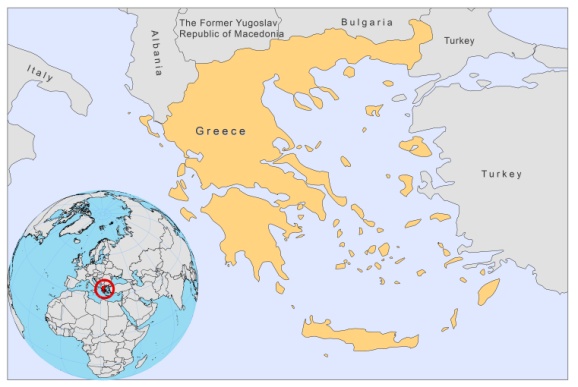
**

**BASIC COUNTRY DATA**

Total Population: 11,319,048

Population 0-14 years: 15%

Rural population: 39%

Population living under USD 1.25 a day: no data

Population living under the national poverty line: no data

Income status: High income economy: OECD

Ranking: Very high human development (ranking 29)

Per capita total expenditure on health at average exchange rate (US dollar): 3,041

Life expectancy at birth (years): 80

Healthy life expectancy at birth (years): 71

**BACKGROUND INFORMATION:**

VL has been common in Greece since 1935. During the 1940s, an average of 160 cases were recorded each year; during the 1950s, 32 cases per year; and from 1962 to 1978, VL increased again to approximately 60 cases per year. From 1979 to 1981, 153 VL cases were reported in Attica (near Athens), the lonian islands and some other areas. Canine VL was predominant before the Second World War (5-15% of the dogs being infected in Crete). Different surveys were held between 1934 and 1938, showing rates of infection in dogs from 6.5% to 22.5%. Currently, the average seroprevalence in dogs in endemic areas varies between 25 and 50% (with most data derived from Attiki and Crete), with a significant high rate of asymptomatic infections in the Greater Athens and in northwestern Greece [1,2]. Between 1999 and 2006 (based on modelling), the seroprevalence among dogs had increased with 2.4% [3]. VL is still frequent in several areas of Greece. From 2004 to 2008, 30-50 VL cases were reported. 15% of blood-donors from endemic areas were seropositive [4].

There are few HIV/VL co-infected patients (0.6% of cases). Recently, a shift occurred from infantile disease to a disease of old people (>60 y).

CL used to be frequent in Crete (4,000 cases in 1938) and endemic in several areas of continental and insular Greece (1951-1975: 271 cases). Now, it occurs sporadically, particularly in the island of Zakynthos (4 cases in 2008).

Both forms are probably underreported, especially CL.

**PARASITOLOGICAL INFORMATION**

| ***Leishmania* species** | **Clinical form** | **Vector species** | **Reservoirs** |
| --- | --- | --- | --- |
| *L. infantum* | ZVL, CL | *P. neglectus, P. tobbi,*  *P. perfiliewi* | *Canis familiaris* |
| *L. tropica* | CL | *P. sergenti* |  |

**MAPS AND TRENDS**

**Visceral leishmaniasis**

**
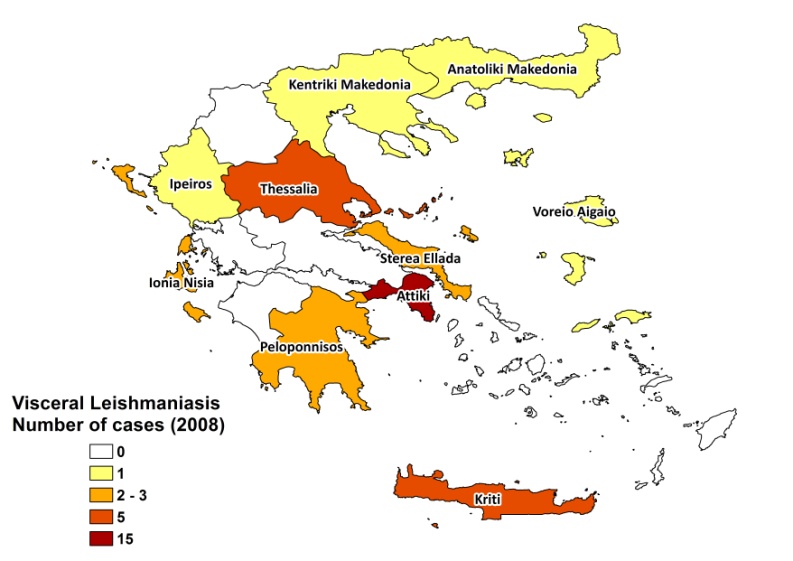
**

**
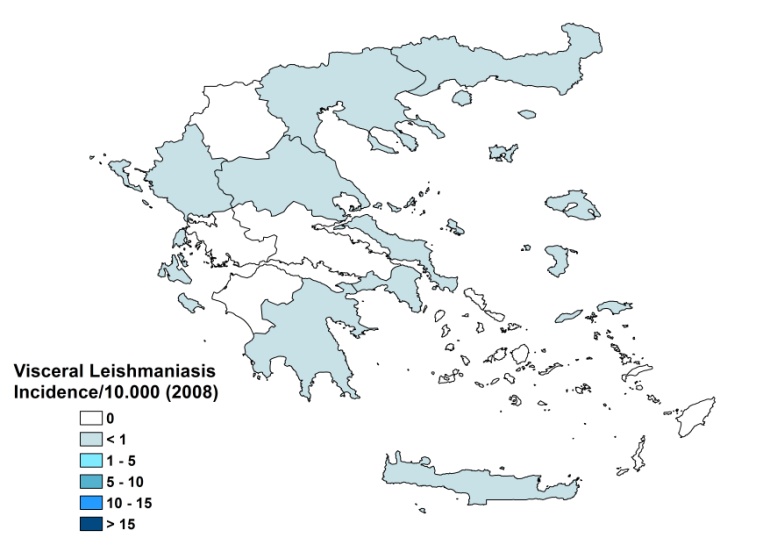
**

**Cutaneous leishmaniasis**

**
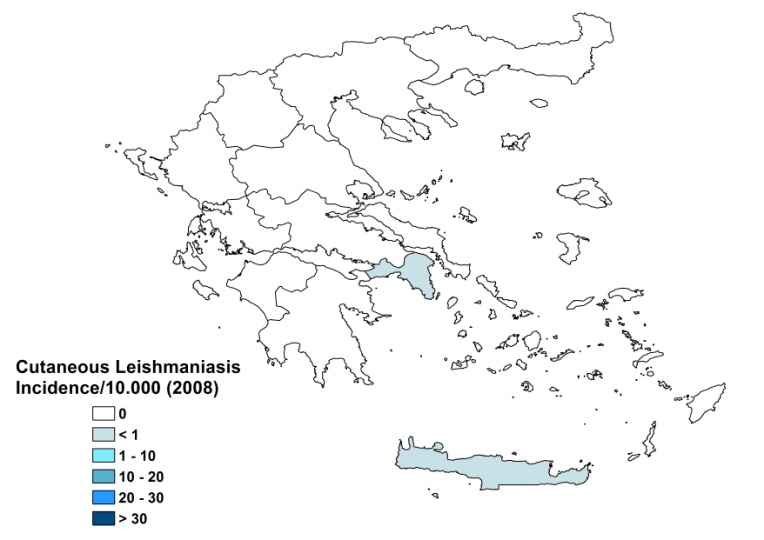

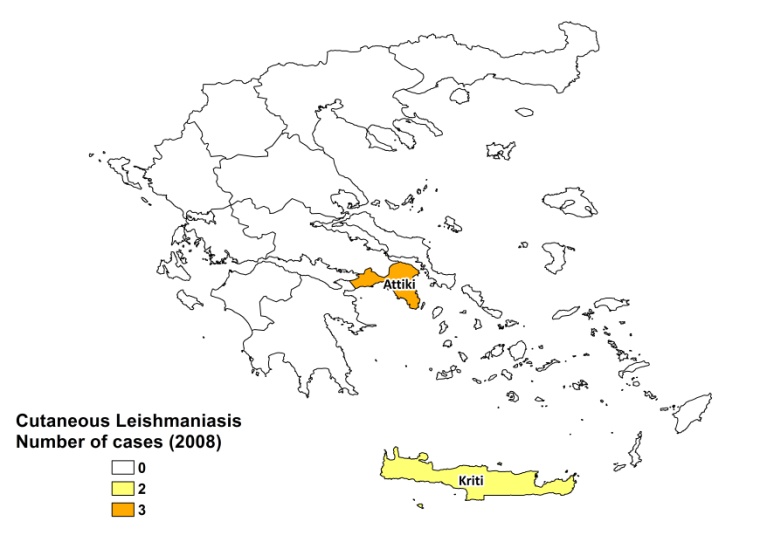
**

**Visceral leishmaniasis trend**

**Cutaneous leishmaniasis trend**

**CONTROL**

The notification of leishmaniasis is mandatory in the country. There is no national leishmaniasis control program. Case detection is passive. There is no leishmaniasis vector control program, no bednet distribution program and no leishmaniasis reservoir control program.

**DIAGNOSIS, TREATMENT**

**Diagnosis**

VL and CL: confirmation by microscopy and serology (ELISA, IFAT). PCR-based techniques are used for detection, typing and follow up (real-time PCR).

**Treatment**

VL: antimonials, 20 mg Sb^v^/kg/day for 20-30 days. The cure rate is >95%. Second-line treatment is liposomal amphotericin B (Ambisome), 3 mg/kg/day for 6-10 days; Children: 5 mg/kg/day for 2 days.

CL: antimonials, 20 mg Sb^v^/kg/day. Cure rate is >95%.

**ACCESS TO CARE**

Treatment is free for both VL and CL in the public sector. All patients are thought to have access to care.

**ACCESS TO DRUGS**

Meglumine antimoniate is included for VL and CL in the National Essential Drug List. Liposomal amphotericin B is included for VL. AmBisome is registered in Greece. Meglumine antimoniate (Glucantime) is not registered.

**SOURCES OF INFORMATION**

- Dr Katina Nicolaidou. Hellenic Pasteur Institute. *Leishmaniasis in the European Region, a WHO consultative intercountry meeting, Istanbul, Turkey, 17–19 November 2009.*

# 1. [Sideris V](http://www.ncbi.nlm.nih.gov/pubmed?term=%22Sideris%20V%22%5BAuthor%5D), [Papadopoulou G](http://www.ncbi.nlm.nih.gov/pubmed?term=%22Papadopoulou%20G%22%5BAuthor%5D), [Dotsika E](http://www.ncbi.nlm.nih.gov/pubmed?term=%22Dotsika%20E%22%5BAuthor%5D), [Karagouni E](http://www.ncbi.nlm.nih.gov/pubmed?term=%22Karagouni%20E%22%5BAuthor%5D) (1999). Asymptomatic canine leishmaniasis in Greater Athens area, Greece. [Eur J Epidemiol.](http://www.ncbi.nlm.nih.gov/pubmed/10395058##)15(3):271-6.

2. Papadopoulou C, Kostoula A, Dimitriou D, Panagiou A, Bobojianni C et al (2005). [Human and canine leishmaniasis in asymptomatic and symptomatic population in Northwestern Greece.](http://www.ncbi.nlm.nih.gov/pubmed/15603841) J Infect 50(1):53-60.

3. Antoniou M, Messaritakis I, Christodoulou V, Ascoksilaki I, Kanavakis N et al (2009). Increasing incidence of zoonotic visceral leishmaniasis on Crete, Greece. Emerg Infect Dis 15(6):932-4.

4. Kyriakou DS, Alexandrakis MG, Passam FH, Kourelis TV, Foundouli P et al (2003). Quick detection of Leishmania in peripheral blood by flow cytometry. Is prestorage leucodepletion necessary for leishmaniasis prevention in endemic areas? Transfus Med 13:59–62.
