# Supplementary material for: Leishmaniasis Worldwide and Global Estimates of Its Incidence
Source: PLoS One. 2012 May 31;7(5):e35671. doi: 10.1371/journal.pone.0035671 (PMC3365071; doi:10.1371/journal.pone.0035671)
Supplement: Text S38 — Leishmaniasis Country Profiles, Guatemala. (DOCX) [file pone.0035671.s038.docx]

**GUATEMALA**


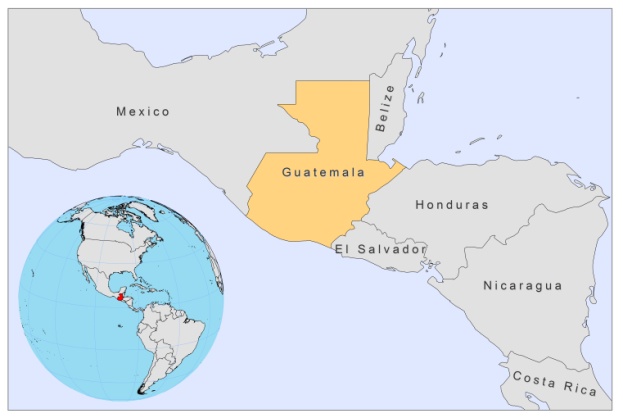


**BASIC COUNTRY DATA**

Total Population: 14,388,929

Population 0-14 years: 41%

Rural population: 51%

Population living under USD 1.25 a day: 13.1%

Population living under the national poverty line: 51%

Income status: Lower middle income economy

Ranking: Medium human development (ranking 131)

Per capita total expenditure on health at average exchange rate (US dollar): 186

Life expectancy at birth (years): 71

Healthy life expectancy at birth (years): 57

**BACKGROUND INFORMATION**

Cutaneous leishmaniasis was first described in Guatemala in 1928. Both clinical presentations are common forms of leishmaniasis in the country [1]. In 1986, a National Commission for the study of Leishmaniasis in Guatemala was established and since then, several clinical and epidemiological studies have been conducted to determine the epidemiology of the CL [2,3]. The most common clinical form observed in Guatemala is the single cutaneous ulcer, which can range from a single small ulcer to a large or multiple lesions. Other clinical forms, such as nodule, papule or verrucum lesions, are also observed, but much less frequently. Most lesions are located in exposed areas of the body, such as the face, ears, and upper limbs. The majority of cases affect men over 10 years of age [4].

During 2000-2007, 84.7% (4,837/5,709) of cases concerned people aged over 10. A total of 67% (3,805/5,709) of patients were male. The factors associated with the disease were: transmission outside the home (wooded areas), and occurrence in males aged over 10 years (workers in rural and wooded areas) [5]. In 2008, the reported incidence for this form of the disease was of 0.1 cases/100,000 inhabitants, with 4,262,387 people at risk in 6 departments.

Most cases of human cutaneous leishmaniasis (CL) in Guatemala are caused by one of two species of *Leishmania*: *L. braziliensis* or *L. mexicana*, both forms having a very distinct natural history. Infections caused by *L. braziliensis* grow rapidly, rarely resolve without specific therapy, and often respond rapidly to treatment with antimonials. Infections caused by *L. mexicana*, in contrast, grow more slowly, often resolve initially without therapy, but often recur despite treatment with antimonials [4].

Very few cases of MCL have been documented; 1,085,357 people are at risk for MCL, mostly in one department.

In 1949, the first case of visceral leishmaniasis was reported in a two-year-old child. Incidence of human VL is low in Guatemala [1]. From 2000-2007, 67 VL cases were diagnosed. However, 4,359,734 people are considered to be at risk for VL in 6 departments of the country. The reported incidence for this form of the disease was 9.8 cases/100,000 inhabitants in 2008.

There have been no reported cases of HIV-*Leishmania* co-infection.

**PARASITOLOGICAL INFORMATION**

| ***Leishmania***  **species** | **Clinical form** | **Vector species** | **Reservoirs** |
| --- | --- | --- | --- |
| *L. braziliensis* | ZCL, MCL | *Lu. ovallesi,*  *Lu. ylephiletor,*  *Lu. panamensis* | *Rattus rattus* |
| *L. panamensis* | ZCL, MCL | *Lu. ylephiletor,*  *Lu. panamensis,*  *Lu. trapidoi* | Unknown |
| *L. mexicana* | ZCL, DCL | *Lu. olmeca olmeca* | Unknown |
| *L. infantum* | ZVL | *Lu. longipalpis,*  *Lu. evansi,*  *Lu. pseudolongipalpis* | *Canis familiaris* |

**MAPS AND TRENDS**

**Cutaneous leishmaniasis**

**
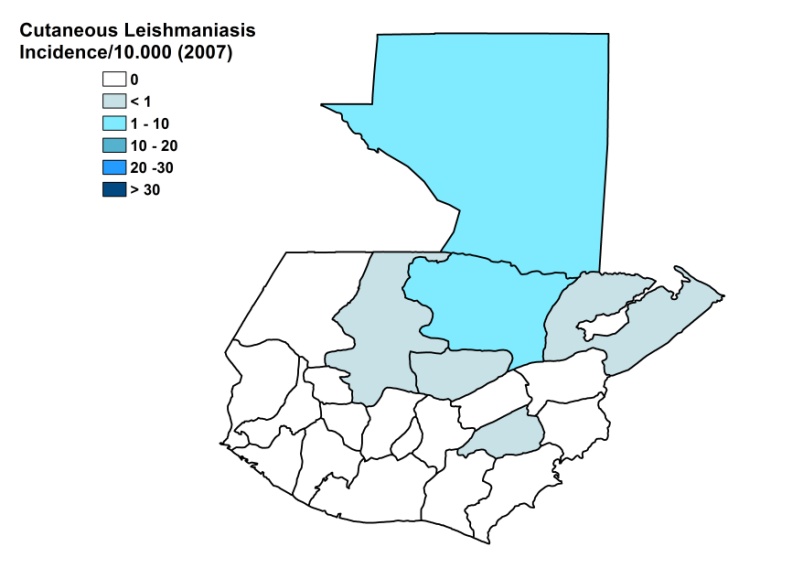

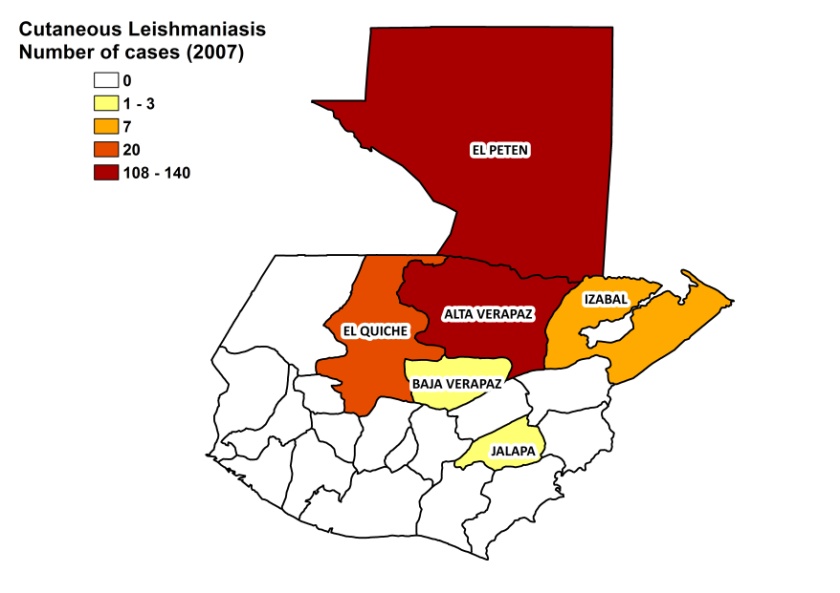
**

**Visceral leishmaniasis**

**Visceral leishmaniasis**


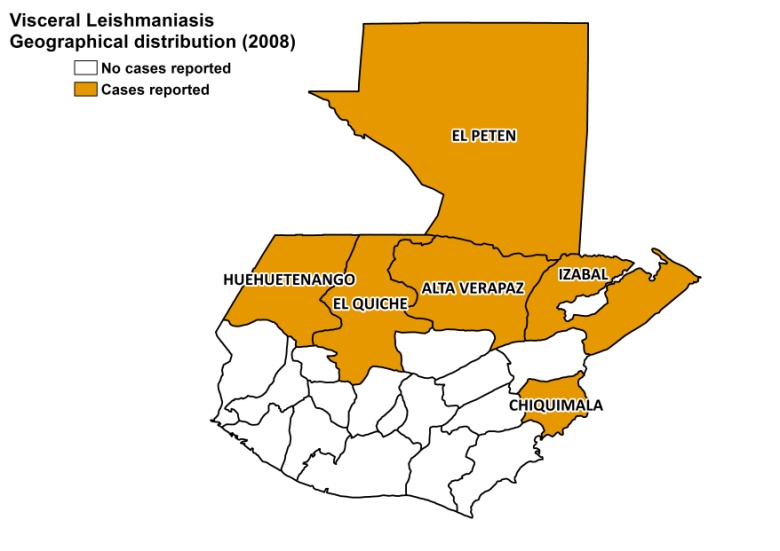


**Cutaneous leishmaniasis trend**

**Number of visceral leishmaniasis cases**

| **2008** | **2009** |
| --- | --- |
| 1 | 1 |

**CONTROL**

In endemic areas in Guatemala, passive epidemiological surveillance is carried out at local level. However, the risk of under-reporting is high, making it impossible to determine the actual scale of the problem nationwide. The factors associated with the disease were: transmission outside the home (wooded areas) and occurrence in males aged over 10 years (workers in rural and wooded areas).

Notification of leishmaniasis is mandatory in the country, and there has been a national control program in place since 2003. Active human case detection is not performed. There is a vector control program, a bednet distribution and insecticide spraying is performed regularly. There are no reservoir control programs and serological screening is not performed in dogs.

**DIAGNOSIS, TREATMENT**

**Diagnosis:**

CL: confirmation by microscopy and sometimes PCR by the National Health Laboratory

VL: on clinical symptoms and confirmation by PCR by the National Health Laboratory

**Treatment:**

CL: antimonials. 95% healing rate for CL and 100% for ML. 5% recurrence rate.

VL: antimonials, 20 mg Sb^v^/kg/day for 20 days. Second line: Ketoconazol, 600 mg/kg/day for 28 days. Third line: Amphotericin B, interdaily dose of 1 mg/kg for a total dosage of 2-3 g.

**ACCESS TO CARE**

Treatment is provided for free, but not all patients have access to treatment. The Ministry of Health purchases insufficient quantities of drugs to enable treatment of all patients. VL can only be diagnosed in one specialized center (blood samples are sent there from health centers) and treated in the national hospital. CL can also be diagnosed and treated at health center level, but there is a lack of health personnel trained in diagnosis and treatment of leishmaniasis. Patients often live very far away from treatment facilities and suffer major economic loss when they spend time away from home. Transport is often unaffordable. There is a great lack of awareness of leishmaniasis among the population. Often substandard treatment is sought in the private sector, or through traditional healers.

**ACCESS TO DRUGS**

Meglumine antimoniate is included in the National Essential Drug List for leishmaniasis. Miltefosine (Paladin, Canada) and meglumine antimoniate (Glucantime, Sanofi) are registered. Drugs for leishmaniasis are not available at private pharmacies or drug markets.

**SOURCES OF INFORMATION**

- Dr Zoraida Morales Monroy. Programa de.Enfermedades Transmitidas por Vectores, Ministerio de Salud. *Leishmaniasis en la Región de las Américas. Reunión de coordinadores de Programa Nacional de Leishmaniasis. OPS/OMS. Medellín, Colombia. 4-6 junio 2008*

1. Padilla E (1928). Contribucion al estudio de la leishmaniasis forestal americana en Guatemala. Thesis, Facultad de Medicina y Cirugia e Institutos Anexos. Universidad San Carlos de Guatemala.

2. Copeland HW, Arana BA, and Navin TR (1990). Comparison of active and passive case detection of cutaneous leishmaniasis in Guatemala. Am J. Trop Med Hyg 43:257-259.

3. Arana BA, Rizzo NR, Navin TR, Klein RE, Kroeger A (2000). Cutaneous leishmaniasis in Guatemala: People's knowledge, concepts and practices. Annals of Tropical Medicine & Parasitology 94: 779-786.

4. Herwaldt BL, Arana BA, Navin TR (1992). The natural history of cutaneous leishmaniasis, Guatemala. Journal of Infectious Diseases 165:518-527.

5. Arana BA (1998). Toward a better understanding of cutaneous leishmaniasis in Guatemala. Ph.D. Thesis, Liverpool School of Tropical Medicine.
