# Supplementary material for: Leishmaniasis Worldwide and Global Estimates of Its Incidence
Source: PLoS One. 2012 May 31;7(5):e35671. doi: 10.1371/journal.pone.0035671 (PMC3365071; doi:10.1371/journal.pone.0035671)
Supplement: Text S39 — Leishmaniasis Country Profiles, Guinea Bissau. (DOCX) [file pone.0035671.s039.docx]

**GUINEA BISSAU**

**
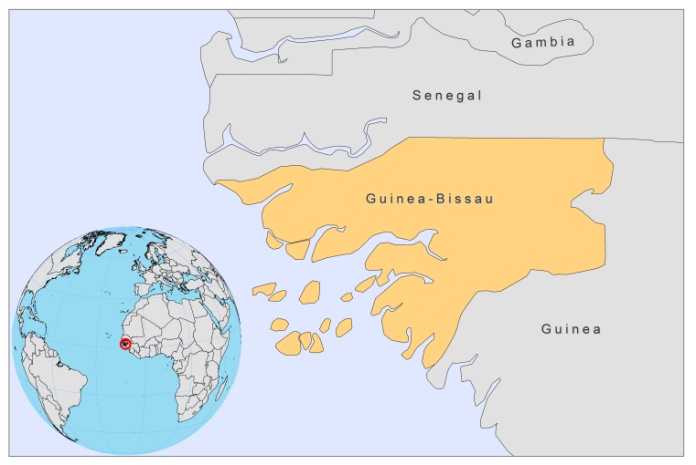
**

**BASIC COUNTRY DATA**

Total Population: 1,515,224

Population 0-14 years: 41%

Rural population: 70%

Population living under USD 1.25 a day: no data

Population living under the national poverty line: no data

Income status: Low income economy

Ranking: Low human development (ranking 176)

Per capita total expenditure on health at average exchange rate (US dollar): 18

Life expectancy at birth (years): 47

Healthy life expectancy at birth (years): 41

**BACKGROUND INFORMATION**

Up to date, no cases of leishmaniasis have been reported in Guinea Bissau. In 1990, a case of atypical visceral leishmaniasis, associated with an HIV infection, was reported in a 10 year old girl [1]. As leishmaniasis species had not been previously described in the country, it was ascribed to infection with a reptilian trypanosomatid.

CL has not been documented, but as Guinea Bissau is part of a proposed CL endemicity belt, running across West Africa, cases may occur regularly and remain unreported [2] .

**PARASITOLOGICAL INFORMATION**

| ***Leishmania***  **species** | **Clinical form** | **Vector species** | **Reservoirs** |
| --- | --- | --- | --- |
| *L. major* | CL | *P. duboscqi* | Unknown |

**MAPS AND TRENDS, CONTROL, DIAGNOSIS, TREATMENT, ACCESS TO CARE, ACCESS TO DRUGS**

No data available.

No antimonials are registered.

**SOURCES OF INFORMATION**

1. Sabbatani S, Isuierdo Calzado A, Ferro A, Lopez Goudiaby AM, Borghi V et al (1991). Atypical leishmaniasis in an HIV-2-seropositive patient from Guinea-Bissau. AIDS 5(7):899-901.

2. Boakye DA, Wilson MD, Kweku M (2005). A review of leishmaniasis in West Africa. Ghana Medical J 39 (3):94-7.
