# Supplementary material for: Leishmaniasis Worldwide and Global Estimates of Its Incidence
Source: PLoS One. 2012 May 31;7(5):e35671. doi: 10.1371/journal.pone.0035671 (PMC3365071; doi:10.1371/journal.pone.0035671)
Supplement: Text S40 — Leishmaniasis Country Profiles, Guinea. (DOCX) [file pone.0035671.s040.docx]

**GUINEA**


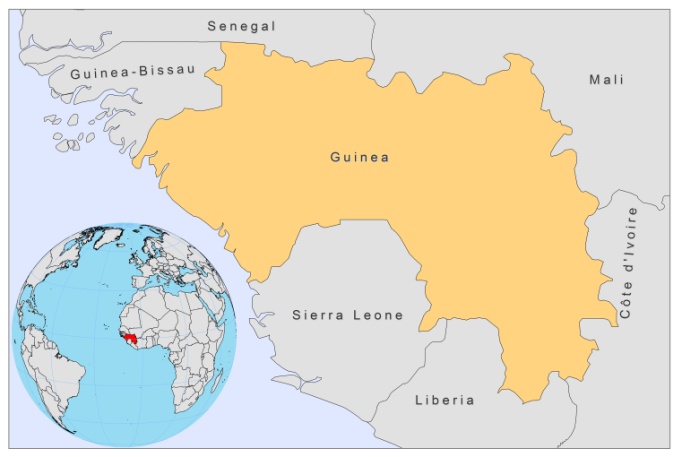


**BASIC COUNTRY DATA**

Total Population: 9,981,590

Population 0-14 years: 43%

Rural population: 65%

Population living under USD 1.25 a day: 43.3%

Population living under the national poverty line: 53%

Income status: Low income economy

Ranking: Low human development (ranking 178)

Per capita total expenditure on health at average exchange rate (US dollar): 19

Life expectancy at birth (years): 53

Healthy life expectancy at birth (years): 45

**BACKGROUND INFORMATION**

Very few data on prevalence or incidence are available. A case of CL has been reported in Conakry in 1977, and an earlier survey found 14.7% of positive response to LST in a population without any symptoms or history of CL [1]. No more recent cases have been documented, but as Guinea is part of a proposed CL endemicity belt, running across West Africa, cases may occur regularly but remain unreported [2].

**PARASITOLOGICAL INFORMATION**

| ***Leishmania***  **Species** | **Clinical form** | **Vector species** | **Reservoirs** |
| --- | --- | --- | --- |
| *L. major* | CL | *P. duboscqi* | Unknown |

**MAPS AND TRENDS, CONTROL, DIAGNOSIS & TREATMENT, ACCESS TO CARE, ACCESS TO DRUGS**

No data available.

**SOURCES OF INFORMATION**

1. Pampiglione S, Marton K (1977). Cutaneous leishmaniasis in the Republic of Guinea. Bull Soc Pathol Exot Filiales 70(5):479-84.

2. Boakye DA, Wilson MD, Kweku M (2005). A review of leishmaniasis in West Africa. Ghana Medical J 39 (3):94-7.
