# Supplementary material for: Leishmaniasis Worldwide and Global Estimates of Its Incidence
Source: PLoS One. 2012 May 31;7(5):e35671. doi: 10.1371/journal.pone.0035671 (PMC3365071; doi:10.1371/journal.pone.0035671)
Supplement: Text S41 — Leishmaniasis Country Profiles, Guyana. (DOCX) [file pone.0035671.s041.docx]

**GUYANA**


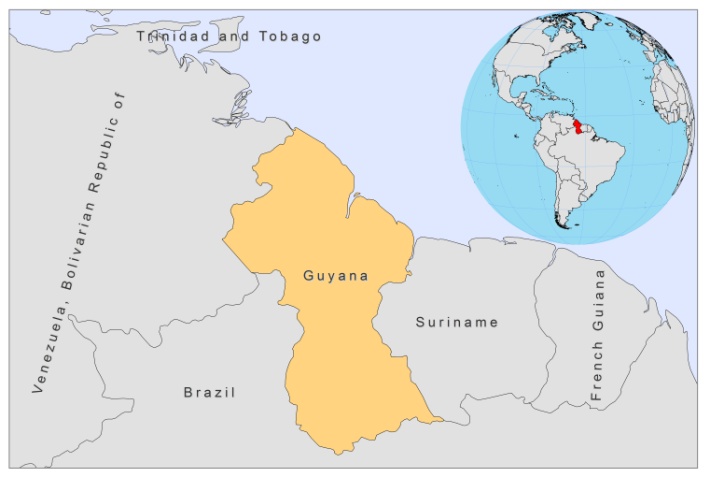


**¨**

**BASIC COUNTRY DATA**

Total Population: 754,493

Population 0-14 years: 34%

Rural population: 72%

Population living under USD 1.25 a day: no data

Population living under the national poverty line: no data

Income status: Lower middle income economy

Ranking: Medium human development (ranking 117)

Per capita total expenditure on health at average exchange rate (US dollar): 133

Life expectancy at birth (years): 69

Healthy life expectancy at birth (years): 55

**BACKGROUND INFORMATION**

VL is unknown in Guyana. CL was first reported in 1912 and has traditionally been an occupational disease of forest workers from inland Guyana. The epidemiology has not been studied.

A retrospective review of 185 patients, presented at the dermatology clinic of Georgetown between 1992 and 1998 with skin ulcers indicative of CL, showed that 43% of the patients were infected with *L. guyanensis.* Most of the cases had very likely been infected in the interior of Guyana and were involved in outdoor work in sylvatic or jungle areas, such as the mining or lumber industry. 95% of the cases were male and most were aged 20-39 [1]. There is only one clinic in Guyana where CL can be treated and the disease is expected to be severely underreported.

No cases of HIV*/Leishmania* co-infection have been reported.

**PARASITOLOGICAL INFORMATION**

| ***Leishmania* species** | **Clinical form** | **Vector species** | **Reservoirs** |
| --- | --- | --- | --- |
| *L. guyanensis* | ZCL | *Lu. umbratilis,*  *Lu. anduzei* | unknown |

**MAPS AND TRENDS**

**Cutaneous leishmaniasis**

**
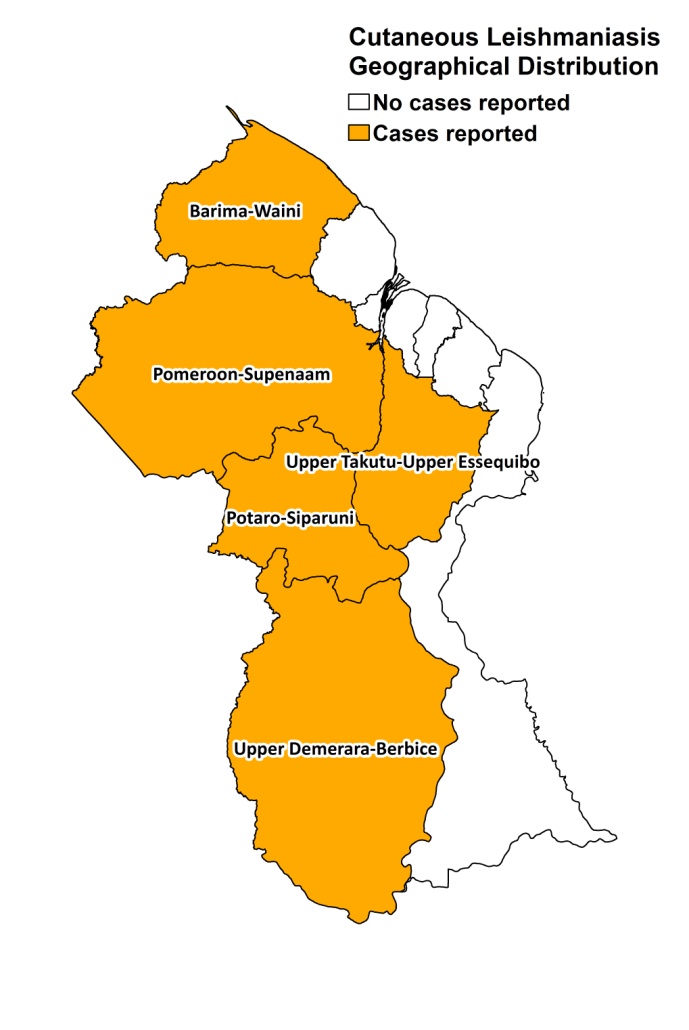
**

**Cutaneous leishmaniasis trend**

| **2006** | **2007** | **2008** |
| --- | --- | --- |
| 17 | 13 | 19 |

**CONTROL**

Notification of CL is mandatory (but in practice this is not done). There is no national leishmaniasis control program. There is no vector control program, but insecticide spraying is done in the context of malaria control. There is no reservoir control program.

**DIAGNOSIS, TREATMENT**

**Diagnosis**

CL: microscopic examination of skin lesion sample.

**Treatment**

CL: antimonials, 20 mg Sb^v^/kg/day for 7-10 days. Sometimes, pentamidine is used. Overall cure rate of 19 patients was 90%, with 16% recurring lesions, 5% mucosal involvement and 5% severe adverse events.

**ACCESS TO CARE**

Treatment for CL is given for free at the skin clinic in Georgetown, but patients have to purchase their own drugs as the Ministry of Health has not provided drugs for leishmaniasis for over 3 years. Out of 49 patients reported in 2006-2009, only 19 received treatment. Diagnosis can only be performed in a few specialized centers. Around 10% of patients seek diagnosis in the private sector before being referred to the public sector. Also, some NGOs and mission hospitals see cases of suspected CL and refer those to the public sector (3 cases in the last 3 years). Most cases occur in the swampy inlands of Guyana. Patients often seek traditional care because of the great distance to the treatment center in Georgetown and the high costs of drugs. In a review about 185 patients held in the 1990s, 46% of cases presented 1-5 weeks after the onset of lesions and 3% after 6 months, having used local herbal remedies (37%); antibiotics and antifungals (39%); and abrasive chemicals, such as lead salts and battery acid [1]. Many patients seek care across the border in Brazil.

**ACCESS TO DRUGS**

Amphotericin B, meglumine antimoniate and pentamidine are included in the National Essential Drug list for leishmaniasis. Meglumine antimoniate (Glucantime, Sanofi) and pentamidine are available in private pharmacies, but are very expensive. Glucantime costs 30-40 USD per vial, leading to a treatment cost of 330-440 USD for an average intralesional treatment. Pentamidine costs 101 USD per vial.

**SOURCES OF INFORMATION**

- Dr Holly Alexander. Public Health Clinic, Ministry of Health.

1. Rawlins SC, Tiwari T, Chadees DD, Validum L, Alexander H et al (2001). American cutaneous leishmaniasis in Guyana, South America. Ann Trop Med Par 95 (3): 245-51.
