# Supplementary material for: Leishmaniasis Worldwide and Global Estimates of Its Incidence
Source: PLoS One. 2012 May 31;7(5):e35671. doi: 10.1371/journal.pone.0035671 (PMC3365071; doi:10.1371/journal.pone.0035671)
Supplement: Text S42 — Leishmaniasis Country Profiles, Honduras. (DOCX) [file pone.0035671.s042.docx]

**HONDURAS**


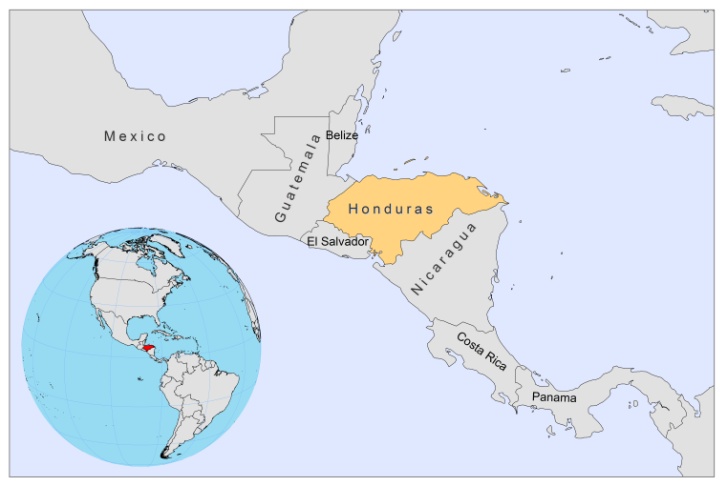


**BASIC COUNTRY DATA**

Total Population: 7,600,524

Population 0-14 years: 37%

Rural population: 51%

Population living under USD 1.25 a day: 23%

Population living under the national poverty line: 60%

Income status: Lower middle income economy

Ranking: Medium human development (ranking 121)

Per capita total expenditure on health at average exchange rate (US dollar): 117

Life expectancy at birth (years): 73

Healthy life expectancy at birth (years): 58

**BACKGROUND INFORMATION**

In Honduras, cases of CL, MCL, VL and non-ulcerative CL have been notified. CL occurs especially along the Caribbean coast, but cases have been reported in the El Paraíso department, in the central south-eastern region of Honduras, and also in the north. Approximately 1,500 cases of CL are notified annually, with an incidence of 0.5 per 10,000 inhabitants, although there is considerable underreporting. CL caused by *L. infantum (*syn. *L.chagasi)* is often atypical, with generally few small lesions that are papular and non-ulcerative with a chronic progression, indicating that the population, in frequent exposure to the parasite, has developed semi-immunity [1]. Mostly older children and young adults are affected. Patients respond very well to antimonial therapy. Although present in the mainland, it is more prevalent in the island El Tigre, near the Pacific coast, always in dry areas where dogs have been found to be infected also, with up to 15.6% seropositivity [1]. The biochemical variant of *L.infantum* found in the lesions is the zymodeme MON-1, the main causative variant of VL in the Mediterranean basin [2]. The homogeneity of the Honduran strains is remarkable when compared to the Mediterranean strains [3]. Other *Leishmania* species causing CL are found in the country's inner forests.

VL has been present since 1974 and a total of 300 cases have been notified; it particularly affects young children under five years of age, most of them under the age of two [4]. The incidence is 0.03 per 10,000 inhabitants. The most important focus of VL is in southern Honduras, in the departments of Choluteca, Valle and El Paraíso, and is restricted to the arid hills close to the Pacific, overlapping with atypical CL [1].

**PARASITOLOGICAL INFORMATION**

| ***Leishmania* species** | **Clinical form** | **Vector species** | **Reservoirs** |
| --- | --- | --- | --- |
| *L. infantum* | ZVL, CL | *Lu. longipalpis* | *Canis familiaris* |
| *L. panamensis* | ZCL, MCL | *Lu. ylephiletor,*  *Lu. panamensis,*  *Lu. trapidoi* | Unknown |
| *L. braziliensis* | ZCL, MCL | *Lu. ovallesi,*  *Lu. panamensis,*  *Lu. ylephiletor* | Unknown |

**MAPS AND TRENDS**

**Visceral leishmaniasis**

**
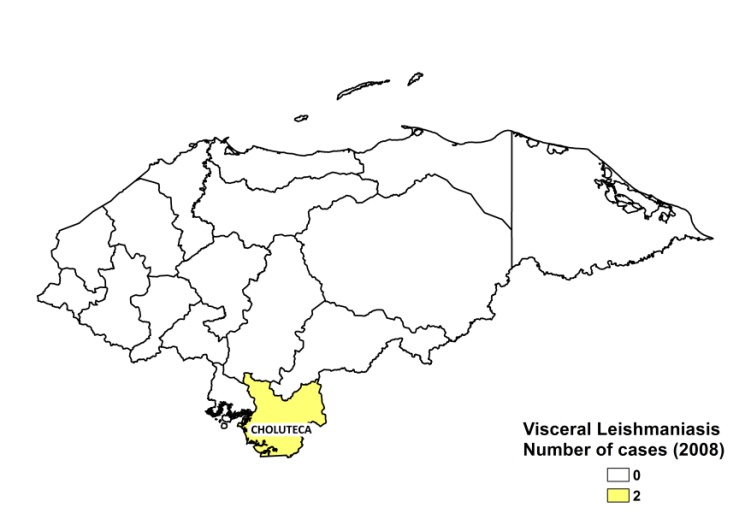
**

**Cutaneous leishmaniasis**


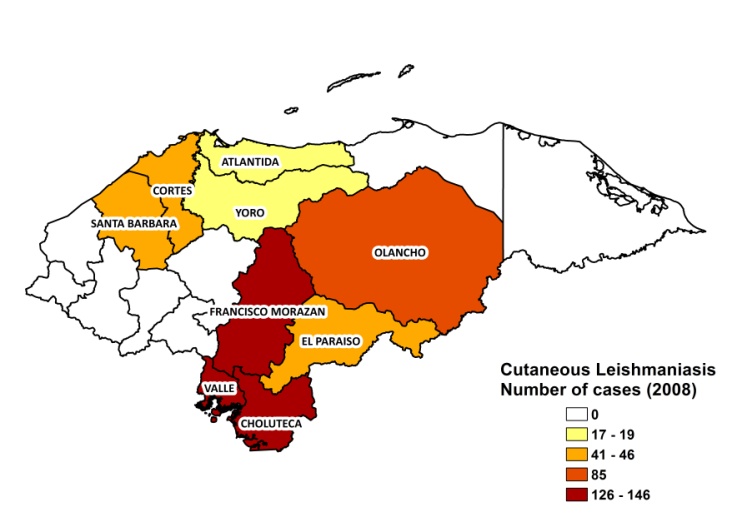

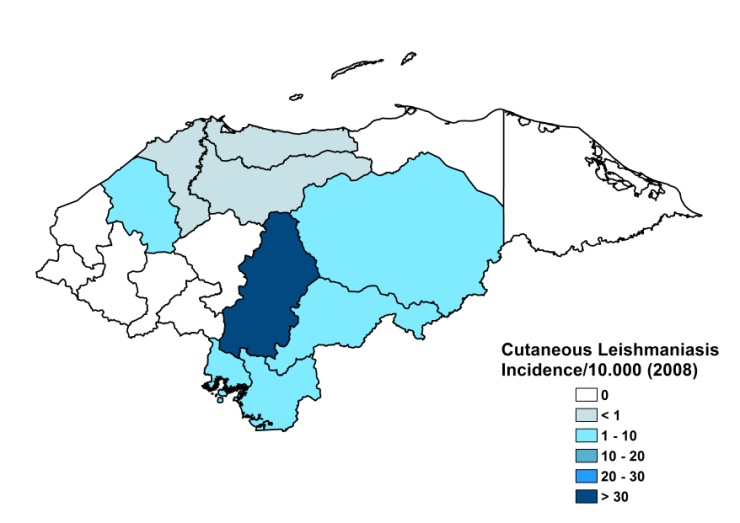


**Visceral leishmaniasis trend**

**Visceral leishmaniasis trend**

**Cutaneous leishmaniasis**

**CONTROL**

The Honduran leishmaniasis control program was set up in 1994. Only VL and MCL are notifiable. As a consequence of the poverty reduction strategy (Millennium Development Goals), the PENCHALE program (2008–2015) aims for a joint control of leishmaniasis and Chagas. Strategies intended are vector control, surveillance with community participation, enhanced diagnosis, access to treatment and environmental improvement.

**DIAGNOSIS, TREATMENT**

**Diagnosis**

CL: microscopic examination of skin lesion sample.

VL: microscopic examination of tissue sample.

**Treatment**

CL: antimonials.

VL: antimonials.

**ACCESS TO CARE**

The Ministry of Health provides meglumine antimoniate (Glucantime, Sanofi). There was a temporary shortage of antimonials in 2008, but it was resolved by a donation of 40,000 vials of generic sodium stibogluconate from Colombia. Treatment for leishmaniasis is provided for free. Diagnosis of VL and CL is possible at primary health care level facilities with laboratories. The private sector is used very little. Access to care is incomplete; only small amounts of patients get treated.

**ACCESS TO DRUGS**

Conventional amphotericin B, meglumine antimoniate and sodium stibogluconate are included in the National Essential Drug List. Meglumine antimoniate (Glucantime, Sanofi) is available in regulated pharmacies for 5.1 USD per vial. Miltefosine (Paladin, Canada) and Glucantime are registered.

**SOURCES OF INFORMATION**

- Dr Concepción Zúñiga Valeriano. Programa Nacional de Chagas y Leishmaniasis, Secretaría de Salud. *Leishmaniasis en la Región de las Américas. Reunión de coordinadores de Programa Nacional de Leishmaniasis. OPS/OMS. Medellín, Colombia. 4-6 junio 2008*

1. Ponce C, Ponce E, Morrison A, Cruz A, Kreutzer R et al (1991). [Leishmania donovani chagasi: new clinical variant of cutaneous leishmaniasis in Honduras.](http://www.ncbi.nlm.nih.gov/pubmed/1670724) Lancet 337(8733):67-70.

2. Noyes H, Chance M, Ponce C, Ponce E, Maingon R (1997). [Leishmania chagasi: genotypically similar parasites from Honduras cause both visceral and cutaneous leishmaniasis in humans.](http://www.ncbi.nlm.nih.gov/pubmed/9085923) Exp Parasitol 85(3):264-73.

3. Campos-Ponce M, Ponce C, Ponce E, Maingon RD (2005) [Leishmania chagasi/infantum: further investigations on Leishmania tropisms in atypical cutaneous and visceral leishmaniasis foci in Central America.](http://www.ncbi.nlm.nih.gov/pubmed/15755418) Exp Parasitol 109(4):209-19.

4. Zeledón R, Macaya G, Ponce C, Chaves F, Murillo J et al (1982). [Cutaneous leishmaniasis in Honduras, Central America.](http://www.ncbi.nlm.nih.gov/pubmed/7101410) Trans R Soc Trop Med Hyg 76(2):276-7.
