# Supplementary material for: Leishmaniasis Worldwide and Global Estimates of Its Incidence
Source: PLoS One. 2012 May 31;7(5):e35671. doi: 10.1371/journal.pone.0035671 (PMC3365071; doi:10.1371/journal.pone.0035671)
Supplement: Text S43 — Leishmaniasis Country Profiles, India. (DOCX) [file pone.0035671.s043.docx]

**INDIA**


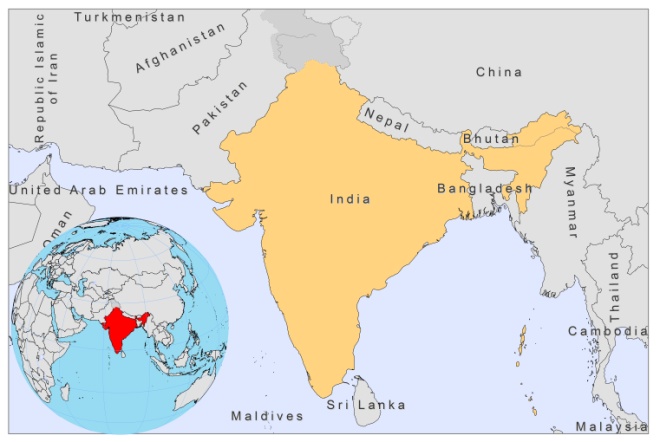


**BASIC COUNTRY DATA**

Total Population: 1,170,938,000

Population 0-14 years: 31%

Rural population: 70%

Population living under USD 1.25 a day: 41.6%

Population living under the national poverty line: no data

Income status: Lower middle income economy

Ranking: Medium human development (ranking 134)

Per capita total expenditure on health at average exchange rate (US dollar): 45

Life expectancy at birth (years): 65

Healthy life expectancy at birth (years): 53

**BACKGROUND INFORMATION**

Until 1947, VL by *L. donovani* was widely prevalent in India. After the DDT spraying campaign, undertaken during the national Malaria Eradication Programme, and effective treatment of all cases, no new cases were reported until 1962-1963, when extensive DDT spraying stopped. VL then returned in the form of large epidemic outbreaks (100,000 cases in 1977 and 40,000 in 1978) [1], which have continued to occur until today. Currently, the endemic area covers the largest part of Bihar and extends to West Bengal, Jharkhand and Uttar Pradesh (NVBDCP, Delhi). In addition, sporadic cases of VL occur in the foothills of the Himalayan mountain range in the northwestern sector of India [2]. Recently, a few cases were reported from Gujarat and Kerala [3,4,5].

Bihar is the most affected state; the case load in Bihar, where 90% of the population lives in extreme poverty, currently represents half of the worldwide burden of VL. Patients mostly belong to the poorest of the poor and VL prolongs the poverty cycle through loss of productivity and unaffordable treatment costs for the affected families [6].

The number of reported cases is a gross underestimation of the real number of cases [6,7,8]. A study conducted in 2006 estimated VL to be underreported with a factor 4,2 [7]. An annual case number of 270,900 was estimated for 2007 [9], with 44,533 reported cases and 203 reported deaths that same year (NVBDCP, Delhi).

HIV-*Leishmania* co-infection is estimated to occur in less than 1% of patients, although a higher incidence was found in some areas [10]. In Bihar, the incidence of HIV is rising and coinfected cases are increasingly reported among migrant workers that have spent time in India’s big cities.

No animal reservoir host could be identified, despite many surveys, and transmission is believed to be anthroponotic, with infected patients and PKDL cases constituting the source of infection [11]. PKDL occurs in an estimated 5-10% of patients and within 5 years of disease onset [12] . It often remains untreated. A recent survey of 4,323 households (2010) in a highly endemic area of Bihar showed a PKDL incidence of 0.5/1,000 population (Prof. Das, personal communication). Risk factors for infection are living in the same household as an active case, and poverty related factors such as poor nutrition and housing conditions [11,13]. Re-emergence of VL in VL free areas occurs periodically and is associated with diminishing herd immunity, the presence of untreated PKDL cases and changing environmental conditions [14].

CL by *L. tropica* and *L. major* occurs in the northwestern states of India (foci in Rajasthan and Punjab). The most affected area in Rajasthan is Bikaner district [15]. Cases identified in other districts usually are immigrants from Bikaner (Dr Bumb, personal communication).

Recently, CL (12 cases with active lesions, out of 38 people examined) and VL (2 cases) have been reported in South India, Kerala, which has implications for the existing elimination program [3,4].

**PARASITOLOGICAL INFORMATION**

| ***Leishmania* species** | **Clinical form** | **Vector species** | **Reservoirs** |
| --- | --- | --- | --- |
| *L. donovani* | AVL, PKDL | *P. argentipes* | Human |
| *L. major* | ZCL | *P. saheli,*  *P. papatasi* | *Meriones hurrianae* |
| *L. tropica* | ACL | *P. sergenti* | Human |

**MAPS AND TRENDS**

**Visceral leishmaniasis**

**
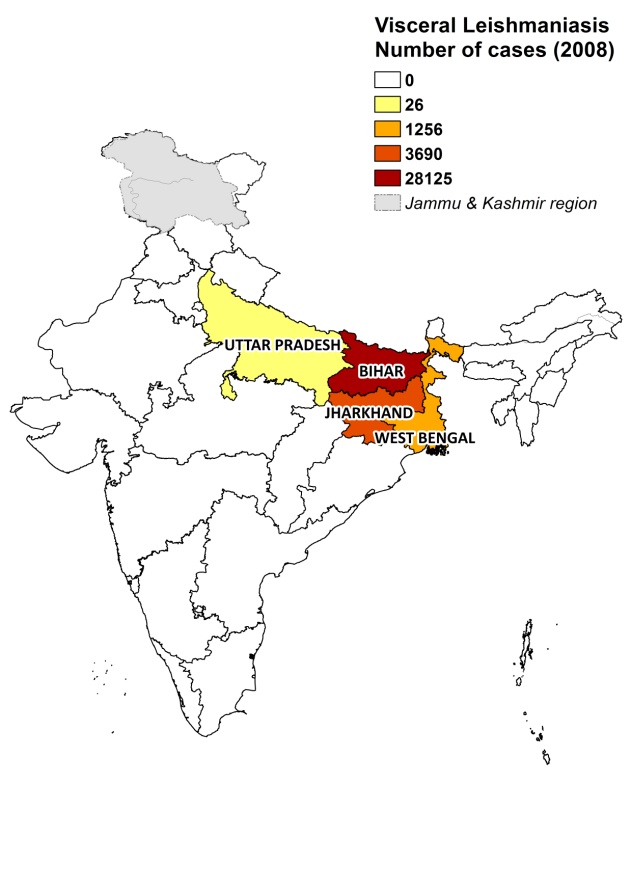

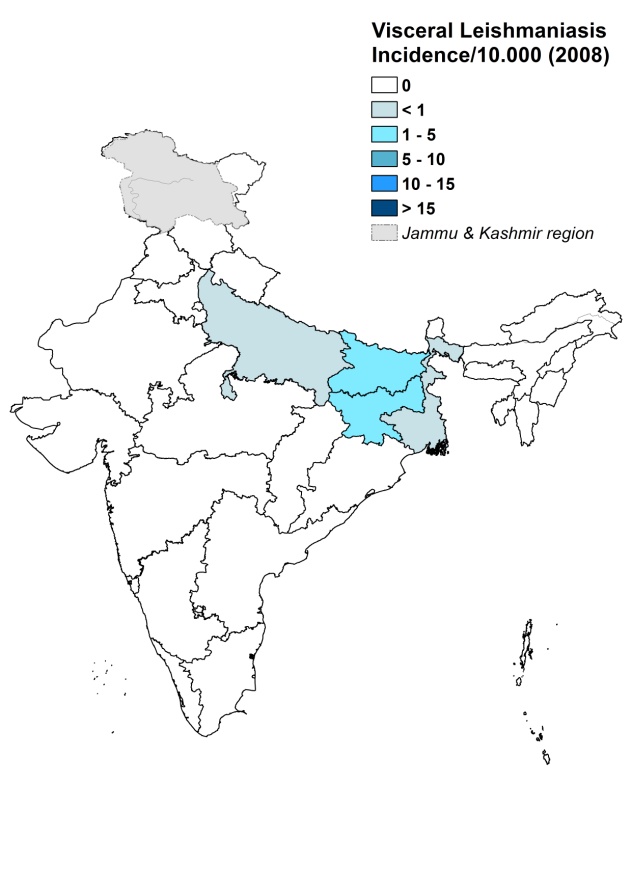
**

**Cutaneous leishmaniasis**

**
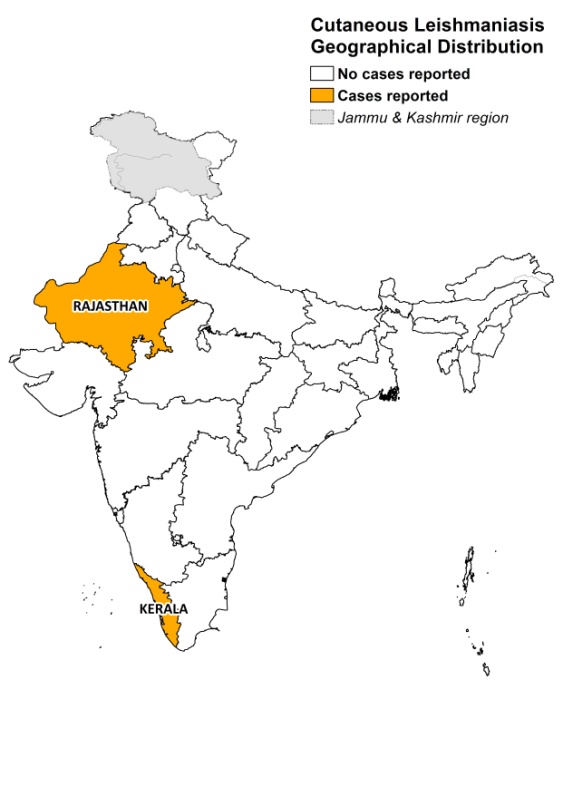
**

**Visceral leishmaniasis trend**

**Cutaneous leishmaniasis trend**

**CONTROL**

The notification of VL is mandatory. Case reporting is based on passive surveillance. A Kala-Azar Elimination program has been in place since 2005, aiming to reduce the incidence of VL to 1/10,000 by 2015 at district or subdistrict level. The plan includes early diagnosis and treatment, vector control, bednet distribution, active surveillance and social mobilization, but has only been implemented on a limited scale due to lack of funding. Reporting of CL is not mandatory and only started in 2005, through the Medical College Hospital (the reference center for case management).

**DIAGNOSIS, TREATMENT**

**Diagnosis**

VL: rK39 antigen-based immunochromatographic test (ICT), microscopic examination of spleen aspirate.

**Treatment**

VL: miltefosine, 2.5 mg/kg/day for 28 days. Cure rate is 94%. Second line: conventional amphotericin B, 1 mg/kg/day for 15 days. Cure rate is 98%. Resistance to antimonials was found to be as high as 60% in endemic foci in Bihar, but antimonials are still in use where miltefosine is not implemented and where no resistant cases have been found (Sb^v^ 20 mg/kg/day for 30 days).

PKDL: antimonials, 20 mg Sb^v^/kg/day for 120 days or conventional amphotericin B, 4-5 courses.

CL: intralesional antimonials, rifampicin, cryosurgery, radiofrequency heat therapy [16].

**ACCESS TO CARE**

The Kala-azar Elimination Plan includes free diagnosis and treatment for VL patients. However, public health facilities often lack trained human resources, diagnostics and drugs for the treatment of VL. In 2007 and 2008, according to estimates, a large amount of patients could not be treated in the public sector, as the government only purchased leishmaniasis treatment (miltefosine, antimonials and amphotericin B) for less than half of the estimated number of patients. The roll out of miltefosine in antimonial-resistant areas is still incomplete. In a survey held in Bihar in 2008, nearly half of the patients were treated with antimonials; 40% of whom needed a second treatment course [17].

Lack of access to treatment is also caused by patients suffering major economic loss when spending time away from home. Patients typically first seek care through a local, but unqualified, village health worker. When seeking care outside the community, only an estimated 30% of patients made use of the public sector, and over 50% of patients resort to poorly trained private practitioners. There is a lack of awareness of the serious nature of the disease, and there is thought to be gender inequality in seeking treatment.

**ACCESS TO DRUGS**

Miltefosine (Paladin, Canada), generic sodium stibogluconate (Albert David, India), paromomycin (Gland Pharma, India), amphotericin B (various generic manufacturers) and AmBisome (Gilead, US) are registered in India. The National Essential Drug List (2003 edition) includes sodium stibogluconate, amphotericin B and pentamidine for leishmaniasis.

Generic sodium stibogluconate, miltefosine and amphotericin B are widely available at the regulated (pharmacies) and unregulated (drug markets) private for-profit sector. One tablet of miltefosine (50 mg) costs 2.5 USD, and one vial of generic sodium stibogluconate costs 4.8 USD, leading to treatment costs of 140 resp. 34 USD for an adult patient of 35 kg. A vial of 50 mg amphotericin B costs 6.3 USD (Rs 301). Paromomycin and AmBisome are not for sale in the private sector. The cost of VL for patients in Bihar was 127 USD, on average, as shown in a recent survey; this is comparable to more than 3 months of income, plus additional high indirect costs due to weeks of wage loss [18] .

Because of lack of access to free treatment, many patients are forced to use the private for-profit sector and can in most cases only afford partial treatments. This forms a major risk for the development of resistant strains against miltefosine.

**SOURCES OF INFORMATION**

- Dr SN Sharma, NVBDCP, Ministry of Health & Family Welfare, Delhi.
- Dr Sujit Batthacharya, SEARO/WHO. *A WHO informal consultation on epidemiological information on disease burden due to kala-azar in Bangladesh, India and Nepal. Paro, Bhutan, 8-10 March 2011*
- Dr Shyam Sundar, Institute of Medical Sciences, Banaras Hindu University, Varanasi. A WHO informal consultation on epidemiological information on disease burden due to kala-azar in Bangladesh, India and Nepal. Paro, Bhutan, 8-10 March 2011.

1. Thakur CP (1984). Epidemiological, clinical and therapeutic features of Bihar kala-azar (including post kala-azar dermal leishmaniasis). [Trans R Soc Trop Med Hyg.](javascript:AL_get(this,%20'jour',%20'Trans%20R%20Soc%20Trop%20Med%20Hyg.');) 1984;78(3):391-8

2. Desjeux P (1991). Information on the epidemiology and control of the leishmaniases by country or territory. 1991. WHO/LEISH/91.30.

3. Simi SM, Anish TS, Jyothi R, Vijayakumar K, Philip RR et al (2010). Searching for cutaneous leishmaniasis in tribals from Kerala, India. [Glob Infect Dis.](javascript:AL_get(this,%20'jour',%20'J%20Glob%20Infect%20Dis.');)2(2):95-100.

4. Kesavan A, Parvathy VK, Thomas S, Sudha SP (2003). Indigenous visceral leishmaniasis: two cases from Kerala. Indian Pediatrics 40:373-374.

5. Sharma U, Redhu NS, Mathur P, Sarman S (2007). Re-emergence of visceral
leishmaniasis in Gujarat, India. J Vect Born Dis 44:230–232.

6. Boelaert M, Meheus P, Sanchez A, Singh SP, Vanlerberghe V et al (2009). The poorest of the poor: a poverty appraisal of households affected by visceral leishmaniasis in Bihar, India. Trop Med Int Health 14(6): 639–644.

7. Singh SP, Reddy DCS, Rai M, Sundar S (2006). Serious underreporting of visceral leishmaniasis through passive case reporting in Bihar. Trop Med Int Health 11(6):899-905.

8. Singh V, Ranjan A, K. Topno R, Verma R, Siddique N et al (2010). Short Report: Estimation of Under-Reporting of Visceral Leishmaniasis Cases in Bihar, India. Am J Trop Med Hyg 82(1): 9–11.

9. TDR multicentric studies on VL supported by WHO/TDR. WHO 2007; p 88.

10. Alvar J, Aparicio P, Aseffa A, Den Boer M, Cañavate C et al (2008). [The relationship between leishmaniasis and AIDS: the second 10 years.](http://www.ncbi.nlm.nih.gov/pubmed/18400800) Clin Microbiol Rev 21(2):334-59.

11. Bern C, Courtenay O, Alvar J (2010). Of cattle, sand flies and men: a systematic review of risk factor analyses for South Asian visceral leishmaniasis and implications for elimination. PLoS Negl Trop Dis 4(2):e599.

12. Ramesh V (1995). Post-kala-azar dermal leishmaniasis Int J Dermatol 34: 95-01.

13. Kesari S, Bhunia G, Kumar V, Jeyaram A, Ranjan A et al (2010). Study of house-level risk factors associated in the transmission of Indian Kala-azar. Parasit Vectors 12(3):94.

14. Sharma U, Redhu NS, Mathur P, Sarman S (2007). Re-emergence of visceral leishmaniasis in Gujarat, India. J Vect Born Dis 44, 230–232.

15. Sharma MID, Sure JOCK, Karla NL, Mohan K, Swami PN (1973). Epidemiological and entomological features of an outbreak of cutaneous leishmaniasis in Bikaner (Rajasthan) during 1971. J Com Dis 5: 54-71.

16. Bumb RA, Mehta R D, Ghiya BC. Jakhar R, Prasad N et al (2010). Efficacy of short-duration (twice weekly) intralesional sodium stibogluconate in treatment of cutaneous leishmaniasis in India. Br J Dermatol 163 (4): 854-8.

17. Hasker E, Singh SP, Malaviya P, Singh RP, Shankar R et al (2010). Management of visceral leishmaniasis in rural primary health care services in Bihar, India. Trop Med Int Health 15 Suppl 2:55-62.

18. Sundar S, Arora R, Singh SP, Boelaert M, Varghese B (2010). Household cost-of-illness of visceral leishmaniasis in Bihar, India. Trop Med Int Health 15 Suppl 2:50-4.
