# Supplementary material for: Leishmaniasis Worldwide and Global Estimates of Its Incidence
Source: PLoS One. 2012 May 31;7(5):e35671. doi: 10.1371/journal.pone.0035671 (PMC3365071; doi:10.1371/journal.pone.0035671)
Supplement: Text S44 — Leishmaniasis Country Profiles, Iran. (DOCX) [file pone.0035671.s044.docx]

**IRAN (Islamic Republic of)**

**
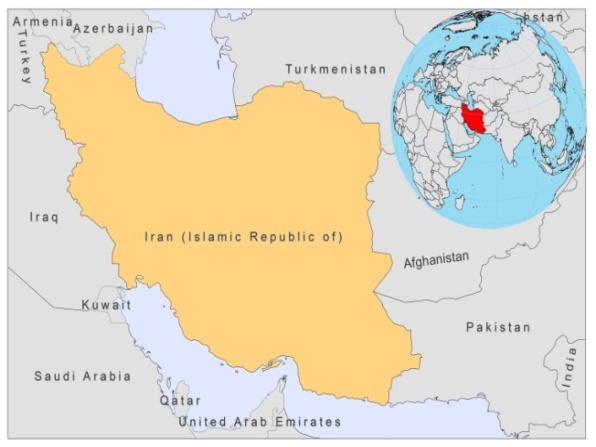
**

**BASIC COUNTRY DATA**

Total Population: 73,973,630

Population 0-14 years: 23%

Rural population: 31%

Population living under USD 1.25 a day: 1.5%

Population living under the national poverty line: no data

Income status: Upper middle income economy

Ranking: High human development (ranking 88)

Per capita total expenditure on health at average exchange rate (US dollar): 269

Life expectancy at birth (years): 72

Healthy life expectancy at birth (years): 58

**BACKGROUND INFORMATION**

CL has been an increasing public health problem with several new foci identified in the last years.

Anthroponotic CL caused by *L. tropica* is found in Teheran as well as in some other large or medium-sized cities and their outskirts. Outbreaks are related to population increase, unplanned urban development and an increase in sandfly population [1]. In the city of Bam, there was an 8-fold increase in the number of cases over the last 5 years after the 2003 earthquake. Recent outbreaks in Bam caused 2,884 cases in 2007, 3,442 in 2008 and 1,372 in 2009, with a high rate of recidivant leishmaniasis [2].

CL caused by *L.major* is endemic and very common in many rural areas, especially in the plains of the northeast, near the Russian border, and in the north of the Esfahan province, in the centre of the country, but has recently spread to its southern parts and to Fars province, in southwest Iran. About 70% of CL in Iran is caused by *L.major*. The endemicity is so high that almost 80% of the rural population contracts the disease before the age of 10 and non-immune newcomers practically all become infected. This area may be the most important focus. However, a recent epidemiological survey in three villages in the Shiraz province, where underreporting was suspected, found a prevalence of scars of 16.2% and of infection (identified by PCR) of 23% [3].

VL is caused by *L. infantum* and is less common [4,5]. The main endemic areas are the province of Fars, in the south, and the districts of Meshkin-Shahr in the northwest. It is thought to be underreported.

HIV-*Leishmania* co-infection has been reported recently [6].

**PARASITOLOGICAL INFORMATION**

| ***Leishmania* species** | **Clinical form** | **Vector species** | **Reservoirs** |
| --- | --- | --- | --- |
| *L. major* | CL | *P. papatasi, P. salehi, P. ansarii* | *Rhombomys opimus, Meriones libycus, Tatera indica, Nesokia sp.* |
| *L. tropica* | CL | *P. sergenti* |  |
| *L. infantum* | VL | *P. major,*  *P. kandelakii,*  *P. halapensis* |  |

**MAPS AND TRENDS**

**Cutaneous leishmaniasis**


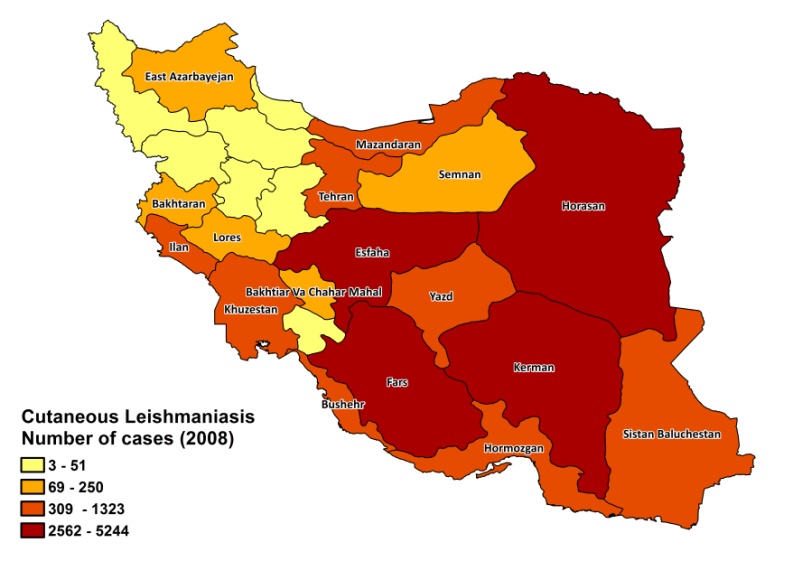

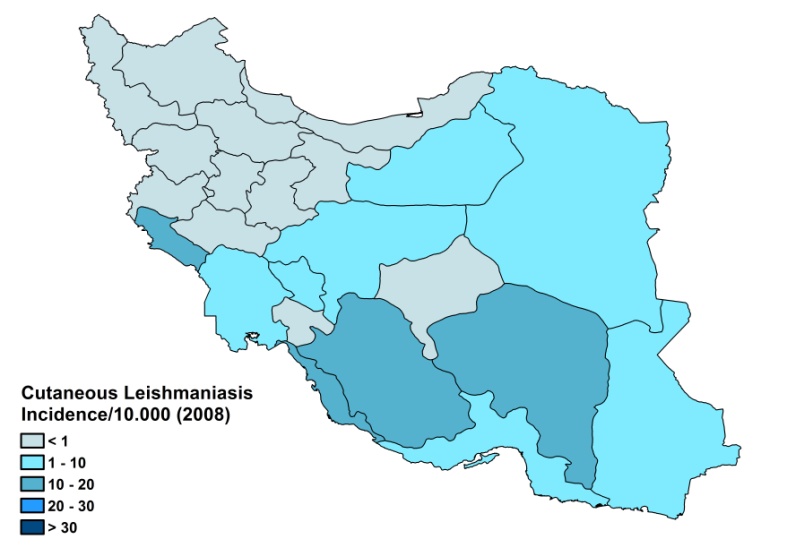


**Visceral leishmaniasis**


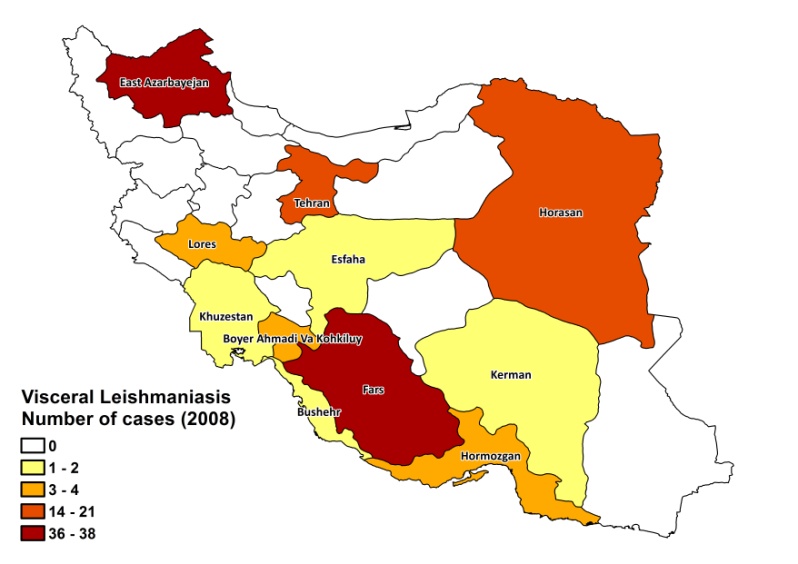

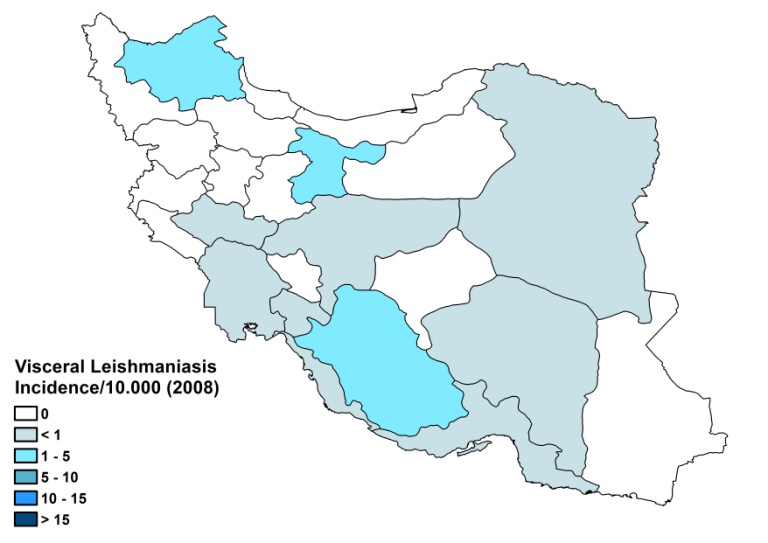


**Cutaneous leishmaniasis trend**

**Visceral leishmaniasis trend**

**CONTROL**

Notification of all forms of leishmaniasis is mandatory in Iran. A national leishmaniasis control program has been in effect since 1978. Active human case detection is regularly performed in new anthroponotic cutaneous leishmaniasis-infected areas. Case detection is passive in zoonotic cutaneous leishmaniasis areas.There is a vector control program in place that includes bednet distribution and regular insecticide spraying. The leishmaniasis reservoir control program includes the sacrifice of seropositive dogs (for VL) and regular rodent control (for CL). Due to a lack of budget and political commitment, the control program has not been efficient in controlling CL and VL.

**DIAGNOSIS, TREATMENT**

**Diagnosis**

CL: on clinical grounds, confirmation with microscopic examination of skin lesion sample.

VL: confirmation by microscopic examination of bone marrow aspirate or DAT.

**Treatment**

CL: CL due to *L.major* is not treated when lesions are small and not facial. In all other cases: antimonials, intralesional (weekly) and cryotherapy (once every two weeks) until cured and for a maximum of 12 weeks. If needed, systemic therapy (20 mg Sb^v^ /kg/day for 2 weeks in CL caused by *L.major* and 3 weeks in CL caused by *L.tropica*. The cure rate is 63% with 2.8% severe adverse events. Treatment failure is especially common in CL caused by *L.tropica*. In non-responsive patients, primary resistance to antimonials has been demonstrated in both *L.major* and *L.tropica* strains.

VL: antimonials, 20 mg Sb^v^/kg/day. Cure rate is 100%.

**ACCESS TO CARE**

Medical care is provided for free in Iran, which includes diagnosis and treatment for leishmaniasis. The Ministry of Health provided sufficient antimonials (Glucantime, Sanofi) in 2007 and 2008 to cover the needs of all patients. CL and VL can be diagnosed and treated at primary health care level; however, there is a lack of trained human resources in health centers for this purpose. The private sector is used by less than 10% of VL patients and less than 20% of CL patients. Due to several reasons, not all CL patients receive treatment. Traditional healers are often consulted in the case of CL before seeking care at health facilities. There is a lack of awareness of the nature of the disease, or patients delay seeking treatment due to the painful procedures of taking a smear for diagnosis and receiving injections of antimonials, which are not always effective in curing the disease. Other patients do not finish treatment due to financial or other constraints. There is a delay in seeking treatment for CL of approximately 1.5 months.

**ACCESS TO DRUGS**

No other drugs than antimonials are included in the National Essential Drug List for leishmaniasis. Meglumine antimoniate (Glucantime, Sanofi) and sodium stibogluconate (Pentostam, GSK) are the only drugs registered for leishmaniasis in Iran. Drugs for leishmaniasis are not available at private pharmacies.

**SOURCES OF INFORMATION**

- Dr Mohammed Reza Shirzadi, Director Zoonoses Control Programme, Ministry of Health and Medical Education, Tehran. *WHO Consultative meeting on Cutaneous Leishmaniasis in EMRO countries, Geneva, 30 April to 2 May 2007.*

1. Yaghoobi-Ershadi MR, Hanafi-Bojd AA, Javadian E, Jafari R, Zahraei-Ramazani AR et al (2002). [A new focus of cutaneous leishmaniasis caused by Leishmania tropica.](http://www.ncbi.nlm.nih.gov/pubmed/11938418) Saudi Med J 23(3):291-4.

2. [Sharifi I](http://www.ncbi.nlm.nih.gov/pubmed?term=%22Sharifi%20I%22%5BAuthor%5D), [Fekri AR](http://www.ncbi.nlm.nih.gov/pubmed?term=%22Fekri%20AR%22%5BAuthor%5D), [Aflatoonian MR](http://www.ncbi.nlm.nih.gov/pubmed?term=%22Aflatoonian%20MR%22%5BAuthor%5D), [Khamesipour A](http://www.ncbi.nlm.nih.gov/pubmed?term=%22Khamesipour%20A%22%5BAuthor%5D), [Mahboudi F](http://www.ncbi.nlm.nih.gov/pubmed?term=%22Mahboudi%20F%22%5BAuthor%5D) et al (2010). Leishmaniasis recidivans among school children in Bam, South-east Iran, 1994-2006. [Int J Dermatol.](javascript:AL_get(this,%20'jour',%20'Int%20J%20Dermatol.');)49(5):557-61.

3. Razmjou S, Hejazy H, Motazedian MH, Baghaei M, Emamy M et al (2009). [A new focus of zoonotic cutaneous leishmaniasis in Shiraz, Iran.](http://www.ncbi.nlm.nih.gov/pubmed/19223055) Trans R Soc Trop Med Hyg 103(7):727-30.

4. Nadim A, Navid-Hamidid A, Javadian E, Bidruni GT, Amini H (1978). [Present status of kala-azar in Iran.](http://www.ncbi.nlm.nih.gov/pubmed/626277) Am J Trop Med Hyg 27(1 Pt 1):25-8.

5. Rahim KM, Ashkan MM (2007). [Epidemiological, clinical and therapeutic features of pediatric kala-azar.](http://www.ncbi.nlm.nih.gov/pubmed/17882998) Southeast Asian J Trop Med Public Health 38(4):626-30.

6. Jafari S, Hajiabdolbaghi M, Mohebali M, Hajjaran H, Hashemian H (2010). [Disseminated leishmaniasis caused by Leishmania tropica in HIV-positive patients in the Islamic Republic of Iran.](http://www.ncbi.nlm.nih.gov/pubmed/20795452) East Mediterr Health J 16(3):340-3.
