# Supplementary material for: Leishmaniasis Worldwide and Global Estimates of Its Incidence
Source: PLoS One. 2012 May 31;7(5):e35671. doi: 10.1371/journal.pone.0035671 (PMC3365071; doi:10.1371/journal.pone.0035671)
Supplement: Text S45 — Leishmaniasis Country Profiles, Iraq. (DOCX) [file pone.0035671.s045.docx]

**IRAQ**

**
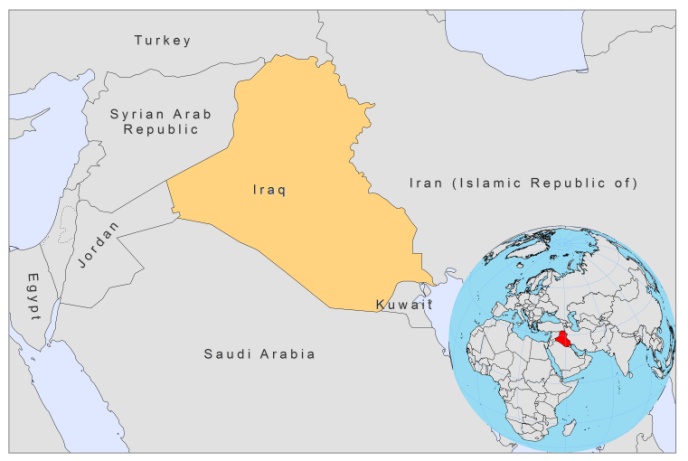
**

**BASIC COUNTRY DATA**

Total Population: 32,030,823

Population 0-14 years: 43%

Rural population: 34%

Population living under USD 1.25 a day: 4%

Population living under the national poverty line: 22.9%

Income status: Lower middle income economy

Ranking: Medium human development (ranking 132)

Per capita total expenditure on health at average exchange rate (US dollar): 98

Life expectancy at birth (years): 58

Healthy life expectancy at birth (years): 50

**BACKGROUND INFORMATION**

VL and CL are both endemic in Iraq. CL declined during the anti-malaria control program and anti-malaria house spraying with DDT, but when this was discontinued in the mid-1960s, the incidence surged. In the 1990s, during the Gulf War (1991), case numbers of VL and CL peaked with an incidence of 45.5/100,000 population for CL in 1992 [1]. Malnutrition is thought to be a risk factor for both forms. The war caused extensive population movement and the migration of a non immune population into endemic foci, and poor sanitary conditions and an upsurge in the sandfly and vector population are thought to be the underlying causes for the rise in cases. The incidence of both forms went down after 2004, but recently, leishmaniasis has again become a rapidly increasing health problem. The incidence of VL increased from 2.5 (2007) to 3.5/100,000 population in 2008, while the incidence of CL doubled from 2/100,000 (2007) to 4/100,000 population in 2008, and 6.6/100,000 population in 2009. In 2009, the number of VL cases doubled in Diala province, while CL caused several outbreaks in 2008 and 2009.

CL is widespread throughout the country, except for the three provinces in the northeast, bordering Turkey and Iran, where cases are rare. It seems that the majority of CL cases reported in Iraq are caused by *L.major* (ZCL) [1]. Two epidemic outbreaks of CL have been reported in Diwania Province in 2008 with about 300 cases and in Baghdad/ Rahmania in 2009 with about 400 cases. These may have been caused by CL by *L.tropica* (ACL), a very old disease in Iraq, also called "Baghdad boil", which used to be common until a few decades ago. CL is an important health problem, as a secondary bacterial infection was reported in 42% of cases [2].

VL is mostly present in central Iraq and the Greater Baghdad area, but since the Gulf War the disease has extended to new areas rarely affected before, such as Missan, Thi-Qar, and Basrah governorates in southeastern Iraq. 90% of VL cases occur in children under 5 years old. Deterioration of the health status, because of malnutrition among children under age 5, probably played a major role. Transmission is probably zoonotic, but anthroponotic transmission may also take place.

Both forms are believed to be underreported to a large degree.

No cases of HIV*-Leishmani*a co-infection have been reported.

**PARASITOLOGICAL INFORMATION**

**PARASITOLOGY**

| ***Leishmania* species** | **Clinical form** | **Vector species** | **Reservoirs** |
| --- | --- | --- | --- |
| *L donovani* | AVL | *P. alexandri* | Human |
| *L. tropica* | ACL | *P. sergenti* | Human |
| *L. major* | CL | *P. papatasi* |  |
| *L. infantum* | VL | unknown |  |

**MAPS AND TRENDS**

**Visceral leishmaniasis**

**
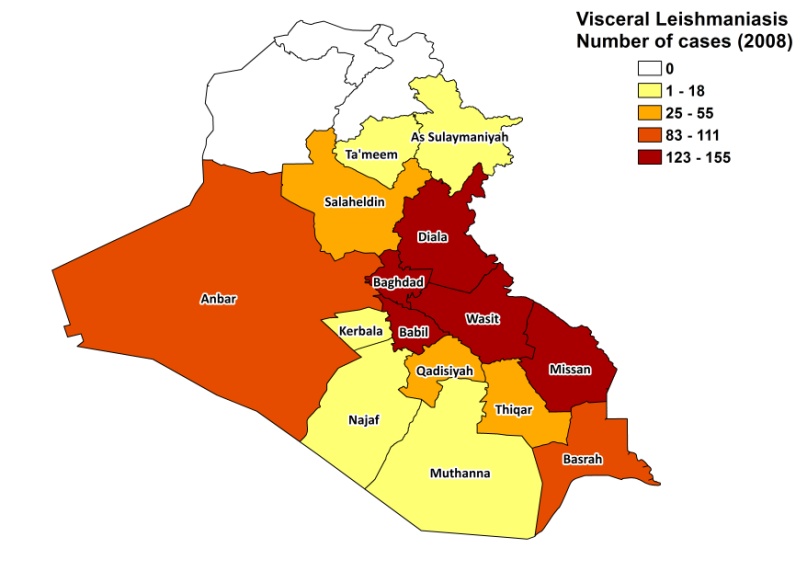

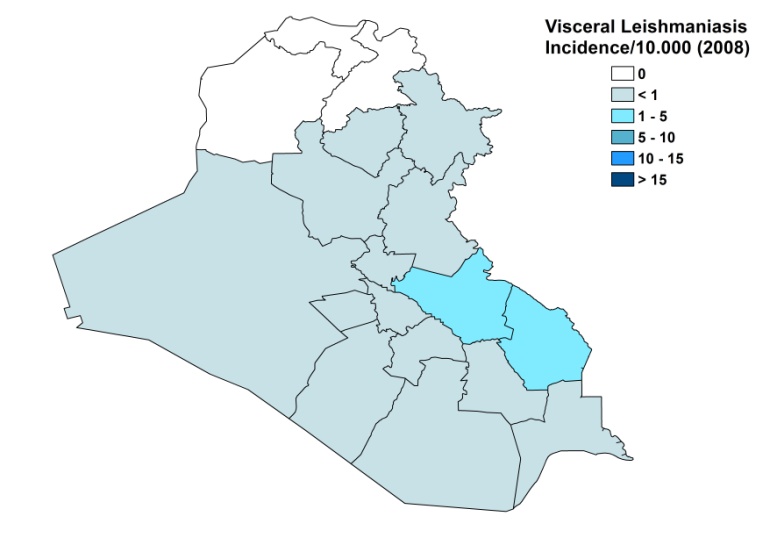
**

**Cutaneous leishmaniasis**

**
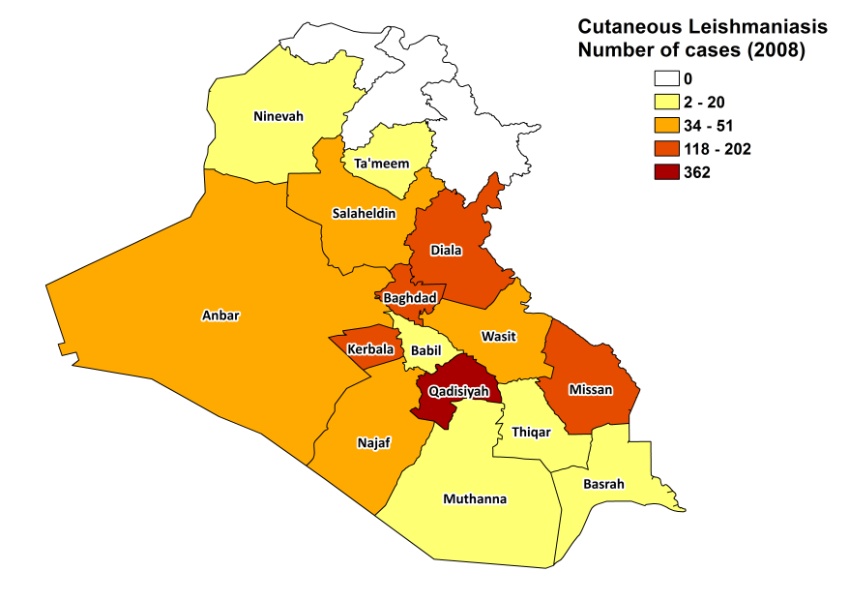
**

**
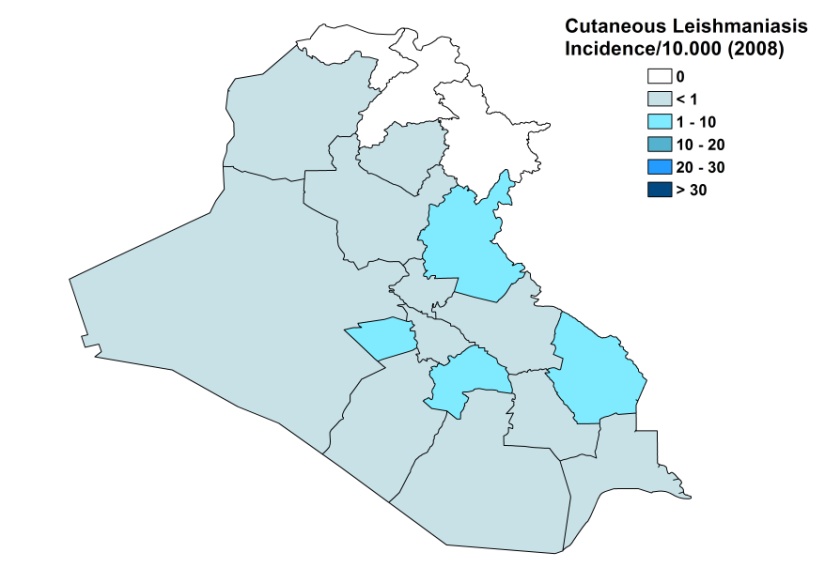
**

**Visceral leishmaniasis trend**

**Cutaneous leishmaniasis trend**

**Cutaneous leishmaniasis trend**

**CONTROL**

Notification of leishmaniasis is mandatory in Iraq. A national leishmaniasis control program has been in effect since 1970 for VL, but not for CL. Case detection is passive. A vector control program is in place that includes bednet distribution and regular insecticide spraying. The leishmaniasis reservoir control program includes the sacrifice of positive dogs (VL) and regular rodent control (CL) Due to a lack of budget and political commitment, the control program has not been efficient in controlling CL and VL.

**DIAGNOSIS, TREATMENT**

**Diagnosis**

VL: rapid diagnostic rK39 antigen-based tests, IFAT and microscopic examination of bone marrow aspirate.

CL: on clinical grounds, occasionally confirmation by microscopic examination of skin lesion sample.

**Treatment**

VL: antimonials, 20 mg Sb^v^/kg/day for 3 weeks. Treatment outcome for VL is a 90% cure rate, with a case fatality rate of 2.9 %. Serious side effects occur in 15% of cases.

CL: for severe cases: antimonials, 20 mg Sb^v^/kg/day for 3 weeks. The cure rate is 80% and lesions reappear in 15% of cases. Severe adverse events are experienced in 1% of cases.

**ACCESS TO CARE**

Medical care is provided for free in Iraq, which includes care for leishmaniasis. In 2007 and 2008, the health centers had the Ministry of Health's permission to purchase antimonials (generic SSG, Albert David, India) in the private sector in order to prevent shortages. In 2009, WHO donated an additional, small quantity of antimonials (Glucantime, Sanofi). VL can be diagnosed and treated at health center level. All VL patients are thought to have access to treatment in Iraq. CL often remains undiagnosed and untreated. The private sector is probably not used as treatment is available in the public sector.

**ACCESS TO DRUGS**

No other drugs than antimonials are included in the National Essential Drug List for VL and CL. Sodium stibogluconate (Pentostam, GSK) is the only drug registered for leishmaniasis in Iraq. Antimonials are sold at private pharmacies (generic SSG by Albert David, India).

**SOURCES OF INFORMATION**

- Dr Abdul Wadod Al-Khafaji, CDC, Baghdad.
- Dr. Somaia Badri El-twijri. Zoonotic Diseases, Ministry of Health, Baghdad. *WHO Consultative meeting on Cutaneous Leishmaniasis in EMRO countries, Geneva, 30 April to 2 May 2007.*

1. WHO (2003) Communicable Disease Working Group on Emergencies, HQ Division of Communicable Disease Control, EMRO, WHO OFFICE, Baghdad. WHO Office, Baghdad. Communicable Disease Toolkit, IRAQ CRISIS. WHO 2003:39-44. [www.who.int/diseasecontrol_emergencies/toolkits/Iraq_profile_ok.pdf](http://www.who.int/diseasecontrol_emergencies/toolkits/Iraq_profile_ok.pdf)
2. Abdulghani Mohamad AlSamarai, Hussein Saher AlObaidi (2009). Cutaneous leishmaniasis in Iraq. J Infect Developing Countries 3(2):123-129.
