# Supplementary material for: Leishmaniasis Worldwide and Global Estimates of Its Incidence
Source: PLoS One. 2012 May 31;7(5):e35671. doi: 10.1371/journal.pone.0035671 (PMC3365071; doi:10.1371/journal.pone.0035671)
Supplement: Text S46 — Leishmaniasis Country Profiles, Israel. (DOCX) [file pone.0035671.s046.docx]

**ISRAEL**


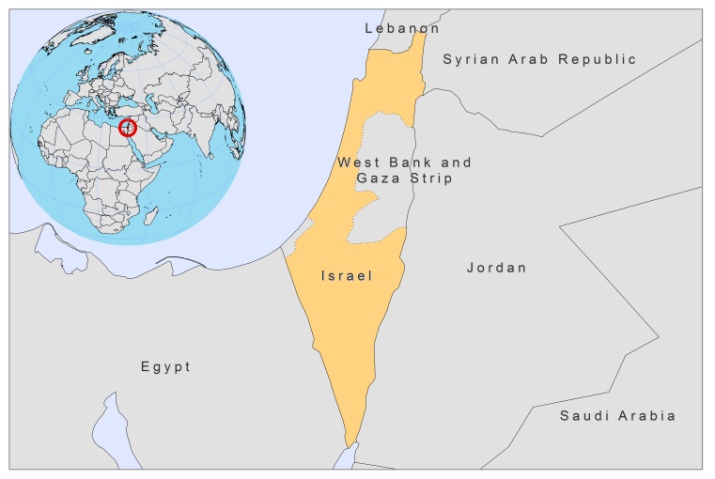


**BASIC COUNTRY DATA**

Total Population: 7,624,600

Population 0-14 years: 27%

Rural population: 8%

Population living under 1.25 USD a day: no data

Population living under the national poverty line: no data

Income status: High income economy: OECD

Ranking: Very high human development (ranking 17)

Per capita total expenditure on health at average exchange rate (US dollar): 1,960

Life expectancy at birth (years): 82

Healthy life expectancy at birth (years): 71

**BACKGROUND INFORMATION**

VL and CL are both endemic in Israel. Since 1995, both VL and CL have spread to new areas. Only sporadic cases of human VL used to occur, but in a survey of 1995, up to 11.5% of dogs were infected with *L. infantum* in villages between Jerusalem and Tel Aviv, suggesting a new endemic focus of VL [1].

The presence of CL was first documented at the beginning of the 20th century. CL by *L. major* is hyperendemic in the Jordan valley (especially in the lower and middle valley) and occurs mainly in the southern regions of Israel. There are small zoonotic foci in the Negev and Arava valleys [2]. Between 1983 and 1987, there was a gradual decrease in the incidence of CL as a result of the drought, which reduced the zoonotic foci and the *Psammomys* population [2]. Since 2003, the number of cases has increased again and 4 outbreaks have occurred in the last 5 years. In 2005, the incidence in soldiers was 200/100,000; the highest incidence now is among soldiers.

CL by *L. tropica* used to be uncommon, but has recently emerged in urban and rural foci of central and northern Israel and forms a potential public health concern [3]. In the past five years, outbreaks of CL due to *L. tropica* have occurred in Tiberias, Ma’ale Adumim and Pduel; outbreaks due to *L. major* have occurred in Nizzana and Beit Se’an.

21% of VL cases are coinfected with HIV (6/28 cases from 1990-2008), but they were all imported.

Both CL and VL are thought to be underreported.

**PARASITOLOGICAL INFORMATION**

| ***Leishmania* species** | **Clinical form** | **Vector species** | **Reservoirs** |
| --- | --- | --- | --- |
| *L. major* | ZCL | *P. papatasi* | *Psammomys obesus, Meriones. crassus, Microtus sociaocaviais, Nosema indica* |
| *L. tropica* | CL | *P. sergenti, P. arabicus* | Human*, Procavia capensis* |
| *L. infantum* | ZVL, CL | *P. syriacus, P. perfiliewi, P. tobbi* | *Canis familiaris* |
| *L. donovani* | CL | unknown |  |

**MAPS AND TRENDS**

**Cutaneous leishmaniasis**

**
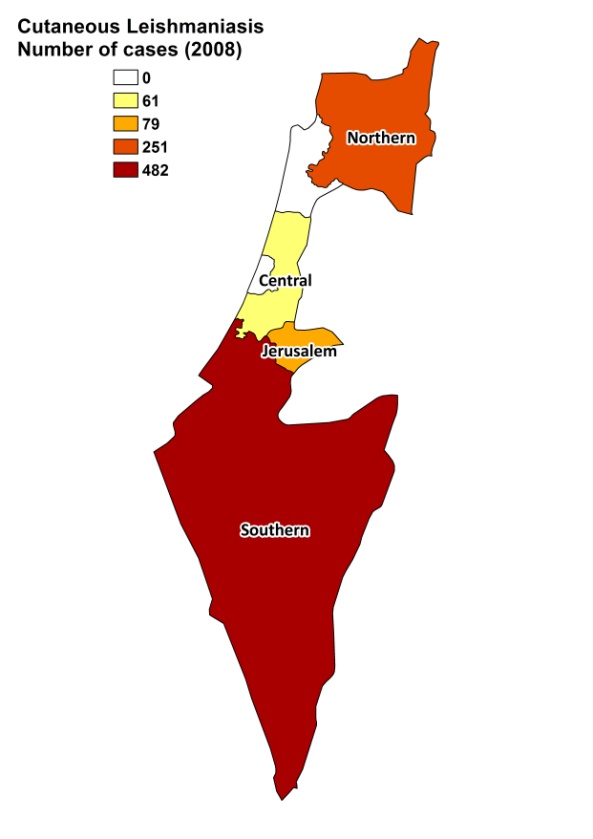
**

**
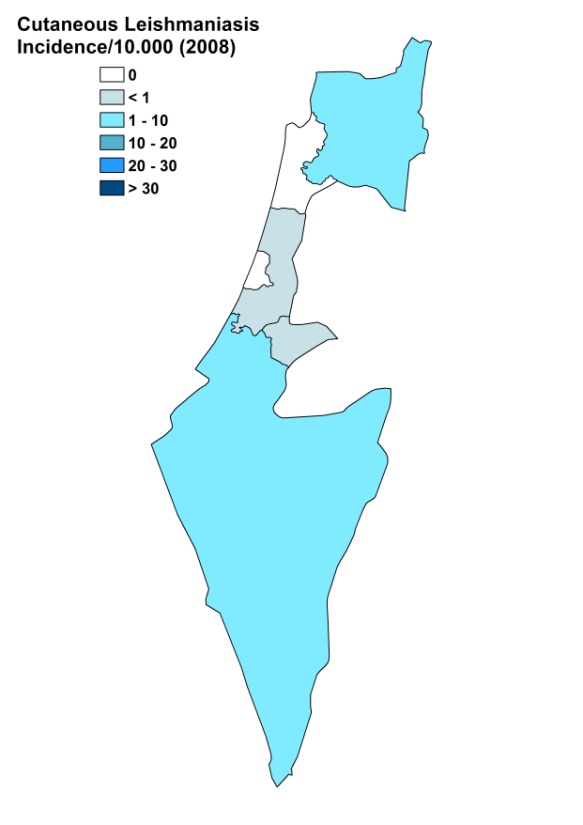
**

**Number of cases of visceral leishmaniasis**

| 1994 | 1995 | 1996 | 1997 | 1998 | 1999 | 2000 | 2001 | 2002 | 2003 | 2004 | 2005 | 2006 | 2007 |
| --- | --- | --- | --- | --- | --- | --- | --- | --- | --- | --- | --- | --- | --- |
| 2 | 2 | 0 | 3 | 2 | 2 | 3 | 0 | 0 | 3 | 3 | 3 | 2 | 0 |

**Cutaneous leishmaniasis trend**

**CONTROL**

The notification of leishmaniasis is mandatory in the country and a national leishmaniasis control program has been in place for CL since 2005. Case detection is passive. There is a leishmaniasis vector control program for CL; insecticide spraying occurs sporadically. There is a leishmaniasis reservoir program for CL; positive dogs are sacrificed if the owner agrees.

**DIAGNOSIS, TREATMENT**

**Diagnosis**

CL: on clinical grounds in local outpatient health centers. Confirmation by microscopic examination of a skin lesion sample can be performed in regional hospitals. PCR is performed in one diagnostic center in Israel.

VL: rK39 based ICT (in specialized hospitals) and PCR (in one diagnostic center).

**Treatment**

VL: antimonials, 20 mg Sb^v^/kg/day for 28 days. Cure rate is > 95%. Second line: liposomal amphotericin B, 3 mg/kg/day for day 1-5, 10, 14 and 21.

CL: antimonials, topical or systemic (20 mg Sb^v^/kg/day for 20 days). Paromomycin ointment is also used for CL.

**ACCESS TO CARE**

Care for leishmaniasis is provided for free, even though a small variable payment may be required for some drugs (e.g., paromomycin ointment). Treatment with liposomal amphotericin B (AmBisome, Gilead) is not reimbursed by all insurance companies. Treatment is thought to be accessible for all patients. CL is diagnosed and treated at health center and outreach post level. The diagnosis of VL takes place in specialized hospitals only. Only very few patients use the private sector (only for CL).

**ACCESS TO DRUGS**

Sodium stibogluconate and liposomal amphotericin B are included in the National Essential Drug List for VL, and sodium stibogluconate and paromomycin ointment are for CL. Drugs are not available at pharmacies or drug markets. Pentostam (GSK) and AmBisome (Gilead) are registered in Israel.

**SOURCES OF INFORMATION**

- Drs Dan Gandacu, Laor Orshan, Tamar Yeger, Ministry of Environmental Protection, IMRIC- Hebrew University-Hadassah, Jerusalem.
- Drs Eli Schwartz and Moshe Ephros, Medical Ctr. Tel Hashomer (ES) and Carmel Medical Ctr., Haifa (ME).
- Dr Charles L. Jaffe, Hebrew University-Hadassah, Jerusalem. *Leishmaniasis in the European Region, a consultative intercountry meeting, Istanbul, Turkey, 17–19 November 2009.*

1. Baneth G, Dank G, Keren-Kornblatt E, Sekeles E, Adini I et al (1998). Emergence of visceral leishmaniasis in central Israel. Am J Trop Med Hyg 59 (5):722-725.

2. Desjeux P (1991). Information on the epidemiology and control of the leishmaniases by country or territory. WHO/LEISH/91.30.

3. [Talmi-Frank D](http://www.ncbi.nlm.nih.gov/pubmed?term=%22Talmi-Frank%20D%22%5BAuthor%5D), [Jaffe CL](http://www.ncbi.nlm.nih.gov/pubmed?term=%22Jaffe%20CL%22%5BAuthor%5D), [Nasereddin A](http://www.ncbi.nlm.nih.gov/pubmed?term=%22Nasereddin%20A%22%5BAuthor%5D), [Warburg A](http://www.ncbi.nlm.nih.gov/pubmed?term=%22Warburg%20A%22%5BAuthor%5D), [King R](http://www.ncbi.nlm.nih.gov/pubmed?term=%22King%20R%22%5BAuthor%5D) et al (2010). Leishmania tropica in rock hyraxes (Procavia capensis) in a focus of human cutaneous leishmaniasis. [Am J Trop Med Hyg.](javascript:AL_get(this,%20'jour',%20'Am%20J%20Trop%20Med%20Hyg.');)82(5):814-8.
