# Supplementary material for: Leishmaniasis Worldwide and Global Estimates of Its Incidence
Source: PLoS One. 2012 May 31;7(5):e35671. doi: 10.1371/journal.pone.0035671 (PMC3365071; doi:10.1371/journal.pone.0035671)
Supplement: Text S47 — Leishmaniasis Country Profiles, Italy. (DOCX) [file pone.0035671.s047.docx]

**ITALY**


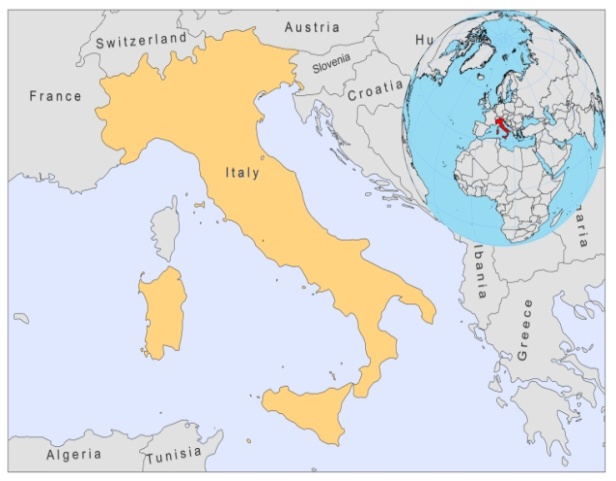


**BASIC COUNTRY DATA**

Total Population: 60,483,521

Population 0-14 years: 14%

Rural population: 32%

Population living under USD 1.25 a day: no data

Population living under the national poverty line: no data

Income status: High income economy: OECD

Ranking: Very high human development (ranking 24)

Per capita total expenditure on health at average exchange rate (US dollar): 3,328

Life expectancy at birth (years): 81

Healthy life expectancy at birth (years): 73

**BACKGROUND INFORMATION**

In Italy, zoonotic VL is endemic, and sporadically zoonotic CL occurs, both caused by *L. infantum*. The most important foci are in Tuscany, Sicily, Campania and Sardinia, with the highest incidence in Sicily and the Naples region [1]. In immunocompetent people, half of the cases occur in children. A high prevalence of canine VL has been recorded in Tuscany, Sicily and Puglia.

For the sandfly species of the subgenus *P. Larioussious* that are proven vectors of *L. infantum* in the Mediterranean, Italy represents an east-west bridge. The western species *P. perniciosus* and *P. ariasi,* as well as the eastern species *P. neglectus* and *P. perfiliewi,* are all found in Italy.

In 2003-2005, Italy provided the first evidence in Europe of the emergence and northward spreading of VL transmission as a probable result of climatic modifications, associated to global warming (influencing dispersion and density of sandflies), and human behavioral factors (importing infected dogs into non-endemic areas). No cases of canine leishmaniasis were recorded in North Italy before, but a prospective survey, carried out between 2003 and 2005, detected 47 cases of canine leishmaniasis and 2% seropositivity among asymptomatic dogs [2]. Since then, autochtonous cases of both VL and CL have been reported from the area. It is not sure if the dog is the only reservoir. Sandfly vectors were identified near the Alps, even in Bolzano/Bozen and near the Mont Blanc.

CL has been endemic in Italy at a relatively constant level since the 1970s, in the same areas that are endemic for VL. CL is largely underreported to the MoH. Only cases that are diagnosed and treated in hospitals are reported, but those diagnosed in private clinics are not. It was estimated that 450–500 cases occurred both in 2004 and 2005.

HIV/*Leishmania* co-infection rose steadily until 1997, after which the incidence decreased sharply as HAART was introduced. In 2007, 6 cases were reported (1.5%). In general, leishmaniasis is thought to be underreported in patients with multiple pathologies.

**PARASITOLOGICAL INFORMATION**

| ***Leishmania* species** | **Clinical form** | **Vector species** | **Reservoirs** |
| --- | --- | --- | --- |
| *L. infantum* | ZVL, CL | *P. perniciosus, P. perfiliewi,*  *P. neglectus, P. ariasi* | *Canis familaris*,  *Vulpes vulpes* |

**MAPS AND TRENDS**

**Visceral leishmaniasis**

**
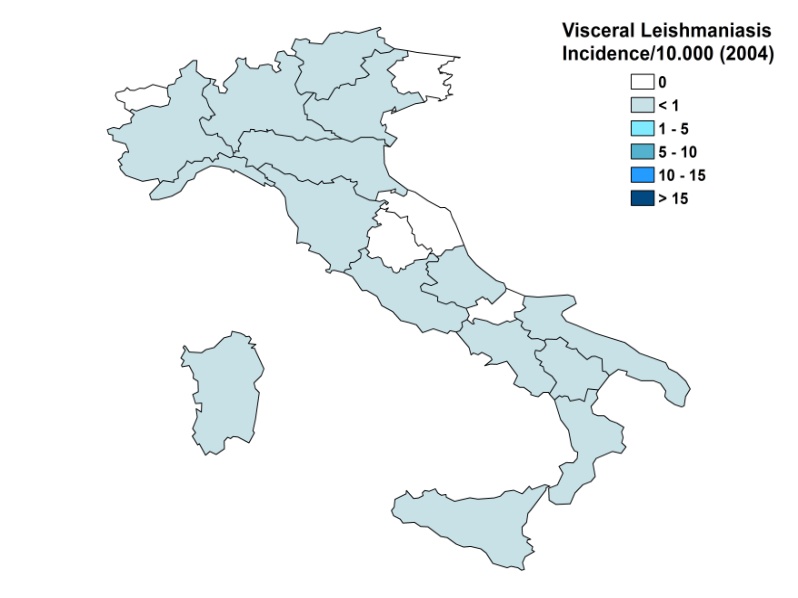

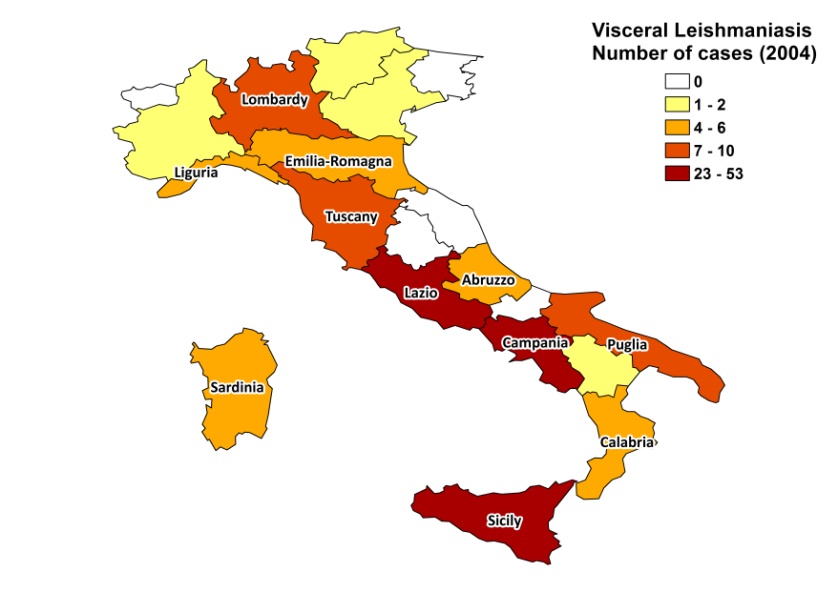
**

**Cutaneous leishmaniasis**

**
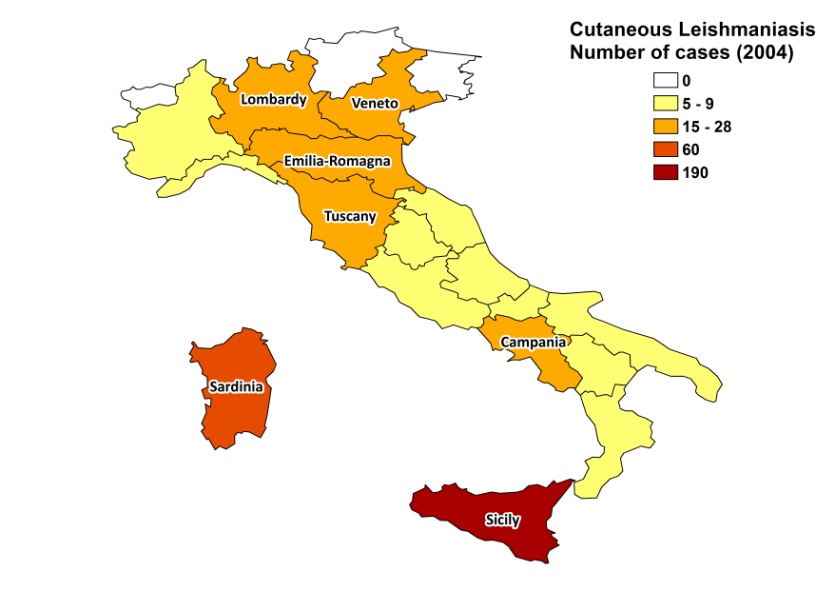
**

**
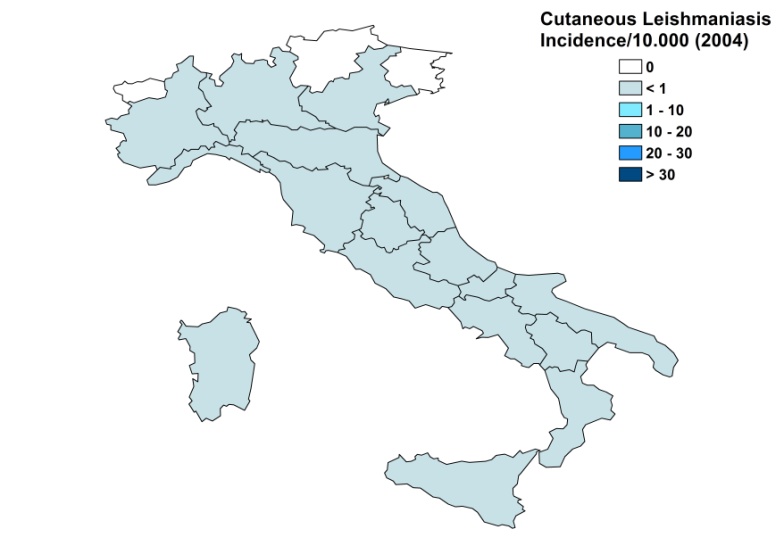
**

**Visceral leishmaniasis trend**

**Cutaneous leishmaniasis trend**

**CONTROL**

The notification of leishmaniasis has been mandatory since 1937 (VL and CL separately, but for VL no distinction between primary and relapsed cases). There is no national leishmaniasis control program and no leishmaniasis vector control program. Insecticide spraying is not done. A leishmaniasis reservoir control program exists in 2 regions, addressing canine leishmaniasis. Serological surveys of dogs are regularly performed. Positive dogs are not sacrificed, but treated and monitored.

**DIAGNOSIS, TREATMENT**

**Diagnosis**

Well-equipped diagnostic laboratories are available in hospitals, university clinics and public health institutions. Definitive diagnosis of leishmaniasis is mainly performed at specialized reference institutes.

**Treatment**

VL: liposomal amphotericin B 3 mg/kg/day for 6-7 days. Cure rate > 95%, relapse in < 4%.

VL/HIV: miltefosine 2.5 mg/kg/day for 28 days. No definitive cure.

CL: antimonials, 20 mg Sb^v^ /kg/day. Cure rate > 95%, recurring in <4%.

**ACCESS TO CARE**

Treatment for VL is given free of charge at public hospitals. There is no standard treatment for CL, which is often given in private clinics. All patients are thought to have access to care.

**ACCESS TO DRUGS**

Liposomal amphotericin B (AmBisome, Gilead) is registered in Italy. Miltefosine and antimonials are not registered in Italy, which means that they need to be imported under special MoH permission. This can cause delays in treatment.

**SOURCES OF INFORMATION**

- Dr. L. Gradoni, Istituto Superiore di Sanità , Rome. *Leishmaniasis in the European Region, a WHO consultative intercountry meeting, Istanbul, Turkey, 17–19 November 2009.*

1. Desjeux P (1991). Information on the epidemiology and control of the leishmaniases by country or territory. WHO/LEISH/91.30.
2. Maroli M, Rossi L, Baldelli R, Capelli G, Ferroglio E et al (2008). The northward spread of leishmaniasis in Italy: evidence from retrospective and ongoing studies on the canine reservoir and phlebotomine vectors. Trop Med Int Health 13(2): 256–264.
