# Supplementary material for: Leishmaniasis Worldwide and Global Estimates of Its Incidence
Source: PLoS One. 2012 May 31;7(5):e35671. doi: 10.1371/journal.pone.0035671 (PMC3365071; doi:10.1371/journal.pone.0035671)
Supplement: Text S48 — Leishmaniasis Country Profiles, Jordan. (DOCX) [file pone.0035671.s048.docx]

**JORDAN**

**
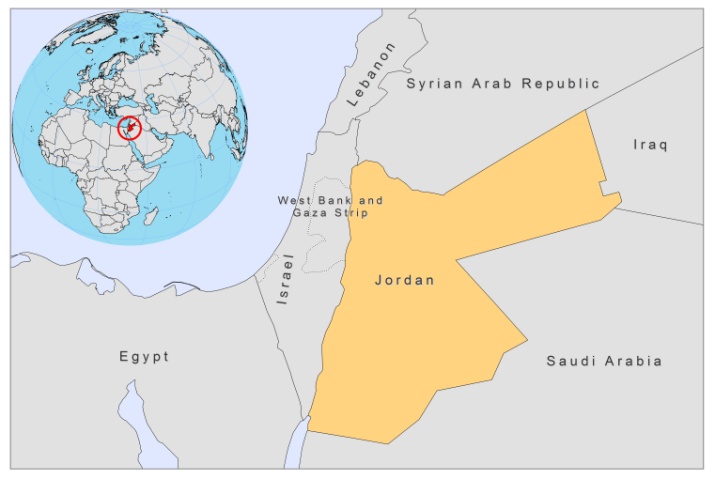
**

**BASIC COUNTRY DATA**

Total Population: 6,047,000

Population 0-14 years: 38%

Rural population: 22%

Population living under USD 1.25 a day: 0.4%

Population living under the national poverty line: 13.3%

Income status: Upper middle income economy

Ranking: Medium human development (ranking 95)

Per capita total expenditure on health at average exchange rate (US dollar): 336

Life expectancy at birth (years): 73

Healthy life expectancy at birth (years): 61

**BACKGROUND INFORMATION**

VL is a rare disease in Jordan, and only about 15 cases have been reported since 1960; the last two of which in 2003 [1]. VL is most likely underreported.

CL due to *L. major* is endemic and used to be known as "Jericho boil". Since 1985, outbreaks have appeared in areas where CL was previously unknown [2,3,4]. The Jordan valley is home to endemic areas with very high infection rates. In the hyperendemic area of Swaimeh, 100% of individuals over 5 years old were found positive in a leishmanin skin test survey in 1992. Higher infection rates (72.4%) are recorded in males than females (27.6%) in all age groups. Disease is more prevalent in children under 5 years (24%) than in those older than 50 (8%) [5].

CL due to *L. tropica* is less common. It occurs in the northern border area, where it is sporadic in rural villages [6]. Imported cases from Saudi Arabia have been found.

Outbreaks of CL have been reported every year for the past 5 years: in Aqaba (2006 and 2007), North Agwar (2008) and South Shuneh (2004 and 2005) with 100-200 cases. Severe underreporting of CL is suspected; between 2001 and 2003, an estimated incidence was 47 times higher than the officially reported number of cases [7]. Among the factors causing underreporting were a lack of physicians' awareness of the importance of notification and lack of treatment.

No cases of HIV*-Leishmani*a co-infection have been reported.

**PARASITOLOGICAL INFORMATION**

| ***Leishmania* species** | **Clinical form** | **Vector species** | **Reservoirs** |
| --- | --- | --- | --- |
| *L. infantum* | ZVL | unknown | *Canis familiaris* |
| *L. tropica* | CL | *P. sergenti* |  |
| *L. major* | ZCL | *P. papatasi* | *Psammomys obesus, Meriones. libycus* |

**MAPS AND TRENDS**

**
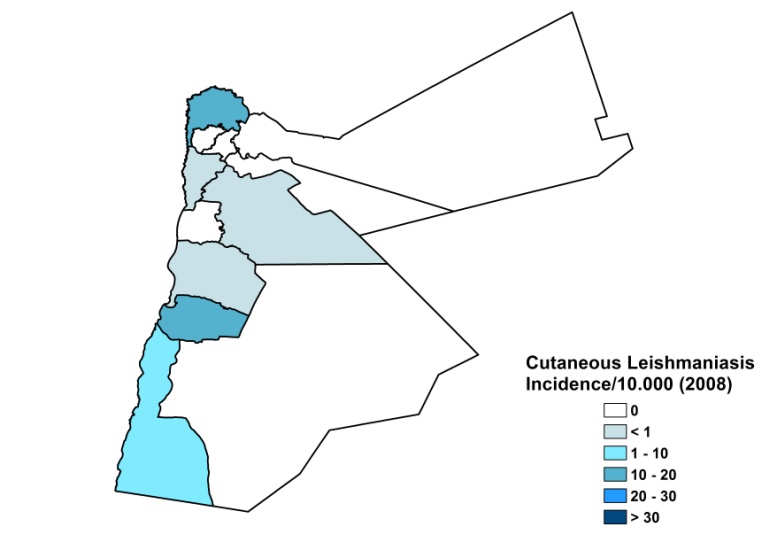

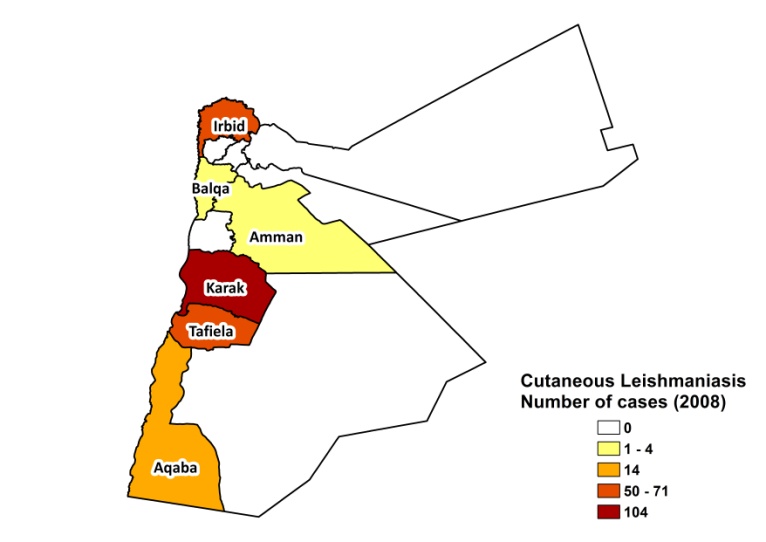
Cutaneous leishmaniasis**

**Cutaneous leishmaniasis trend**

**CONTROL**

The notification of leishmaniasis is mandatory. There is no national leishmaniasis control program. Case detection is passive.

**DIAGNOSIS, TREATMENT**

**Diagnosis**

CL: on clinical grounds. Confirmation with microscopic examination of skin lesion sample in specialized hospitals. PCR is only possible outside of Jordan.

VL: microscopic examination of aspirate (sometimes with cultures) in specialized hospitals. PCR is only possible outside of Jordan.

**Treatment**

CL: antimonials, intralesional and systemic (10 mg Sb^v^/kg/day), cryotherapy and antibiotics. Reported cure rate for topical treatment with antimonials is 100%.

**ACCESS TO CARE**

Medical care is provided for free in Jordan, and includes care for leishmaniasis. A small number of patients is treated outside the public health system, by the Royal Medical Services. VL can only be diagnosed and treated in specialized hospitals. CL is diagnosed (on clinical grounds) and treated in health posts and health centers, but there is no treatment available at this level, other than topical and oral antibiotics. Antimonials and cryotherapy are only provided at hospital level. The Ministry of Health provided antimonials (Pentostam, GSK) for the topical treatment of about 100 patients in 2007 and about 375 patients in 2008, which is less than the number of patients reported, therefore, leading to drug shortages. There is a lack of trained human resources to treat CL and a lack of awareness of the disease among the public and health workers. In some communities, CL lesions are considered a normal event; and in remote rural communities, traditional healing methods, such as plant extracts and lightened cigarettes, are used to destroy lesions.

**ACCESS TO DRUGS**

Sodium stibogluconate is included in the National Essential Drug List for VL and CL. For CL, also cryotherapy and several antibiotics are included. Pentostam (GSK) is the only drug registered in Jordan for leishmaniasis. Drugs for leishmaniasis are not available at private pharmacies and are not sold at informal drug markets.

**SOURCES OF INFORMATION**

- Dr. Khalil Abdul-Aziz Kanani, Ministry of Health.

1. Hicks L, Kant P, Tay PH, Vincini V, Schuster H et al (2009). [Visceral Leishmaniasis presenting with intestinal failure: a case report and literature review.](http://www.ncbi.nlm.nih.gov/pubmed/19011572) Eur J Gastroenterol Hepatol 21(1):117-22.

2. Jumaian N, Kamhawi SA, Halalsheh M, Abdel-Hafez SK (1998). [Short report: outbreak of cutaneous leishmaniasis in a nonimmune population of soldiers in Wadi Araba, Jordan.](http://www.ncbi.nlm.nih.gov/pubmed/9502598)Am J Trop Med Hyg 58(2):160-2.

3. Khoury S, Saliba EK, Oumeish OY (1999). [Short report: outbreak of cutaneous leishmaniasis in a nonimmune population of soldiers in Wadi Araba, Jordan.](http://www.ncbi.nlm.nih.gov/pubmed/10348221) Am J Trop Med Hyg 60(4):520.

4. Mosleh IM, Geith E, Schönian G, Kanani KA (2009). [Two recent but temporally distinct outbreaks of cutaneous leishmaniasis among foreign workers in the Dead-Sea area of Jordan.](http://www.ncbi.nlm.nih.gov/pubmed/19583910) Ann Trop Med Parasitol 103(5):393-400.

5. Nimri L, Soubani R, Gramiccia M (2002). [Leishmania species and zymodemes isolated from endemic areas of cutaneous leishmaniasis in Jordan.](http://www.ncbi.nlm.nih.gov/pubmed/12473179) Kinetoplastid Biol Dis 1(1):7.

6. Saliba E, Saleh N, Bisharat Z, Oumeish O, Khoury S et al (1993). [Cutaneous leishmaniasis due to Leishmania tropica in Jordan.](http://www.ncbi.nlm.nih.gov/pubmed/8296360) Trans R Soc Trop Med Hyg 87(6):633.

7. Mosleh IM, Geith E, Natsheh L, Abdul-Dayem M, Abotteen N (2008). [Cutaneous leishmaniasis in the Jordanian side of the Jordan Valley: severe under-reporting and consequences on public health management.](http://www.ncbi.nlm.nih.gov/pubmed/18363585) Trop Med Int Health 13(6):855-60.
