# Supplementary material for: Leishmaniasis Worldwide and Global Estimates of Its Incidence
Source: PLoS One. 2012 May 31;7(5):e35671. doi: 10.1371/journal.pone.0035671 (PMC3365071; doi:10.1371/journal.pone.0035671)
Supplement: Text S49 — Leishmaniasis Country Profiles, Kazakhstan. (DOCX) [file pone.0035671.s049.docx]

**KAZAKHSTAN**


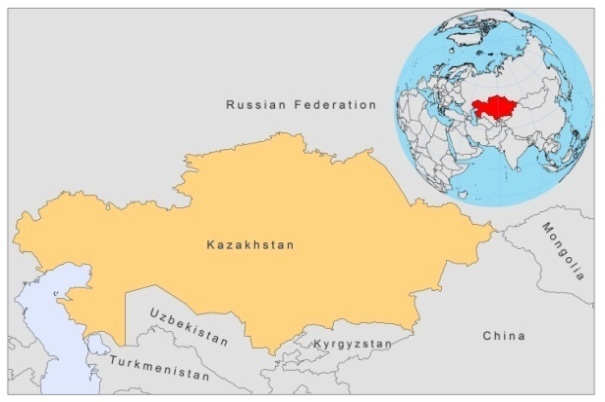


**BASIC COUNTRY DATA**

Total Population: 16,316,050

Population 0-14 years: 24%

Rural population: 42%

Population living under 1.25 USD a day: 0.2%

Population living under the national poverty line: no data

Income status: Upper middle income economy

Ranking: High human development (ranking 68)

Per capita total expenditure on health at average exchange rate (US dollar): 330

Life expectancy at birth (years): 68

Healthy life expectancy at birth (years): 56

**BACKGROUND INFORMATION**

Kzyl-Orda, Shimkent (Southern Kazakhstan) and Djambul are historically *L. infantum* VL endemic regions in Kazakhstan, with 96% of cases children under 3 years old. VL almost disappeared after 1960. Isolated fatal cases occurred after 2000.In 2007-2009, no more new cases were reported.

CL is an endemic disease in Kazakhstan (the southern regions). In the 1980s, outbreaks of CL were observed in Shymkent and Qyzylorda regions due to changes in environmental and climatic conditions, leading to an increase of vector populations. During 2002-2006, 147 CL cases were reported. 40% were children under 14 years old, helping their parents with agricultural work in the fields. In 2003 and 2004, 2 outbreaks occurred, with 51 and 65 cases. Following increased reservoir and vector control activities in endemic foci, the case load decreased dramatically and no further cases of CL were reported in the country in 2007 and 2008. However, in 2009, 9 new cases, including 3 cases among children under the age of 14, occurred in South-Kazakhstan.

Measures to increase public awareness of leishmaniasis were recently taken and local health workers are trained regularly. Despite these measures, both CL and VL are suspected to be underreported due to a continuing lack of awareness of the disease and a lack of trained lab personnel.

There are no reported cases of HIV-*Leishmania* co-infection.

**PARASITOLOGICAL INFORMATION**

| ***Leishmania* species** | **Clinical form** | **Vector species** | **Reservoirs** |
| --- | --- | --- | --- |
| *L. infantum* | ZVL | *P. smirnovi,*  *P. longiductus* | *Canis familiaris* |
| *L major* | ZCL | *P. papatasi,*  *P. mongolensis* | *Rhombomys opimus* |

**MAPS AND TRENDS**

**
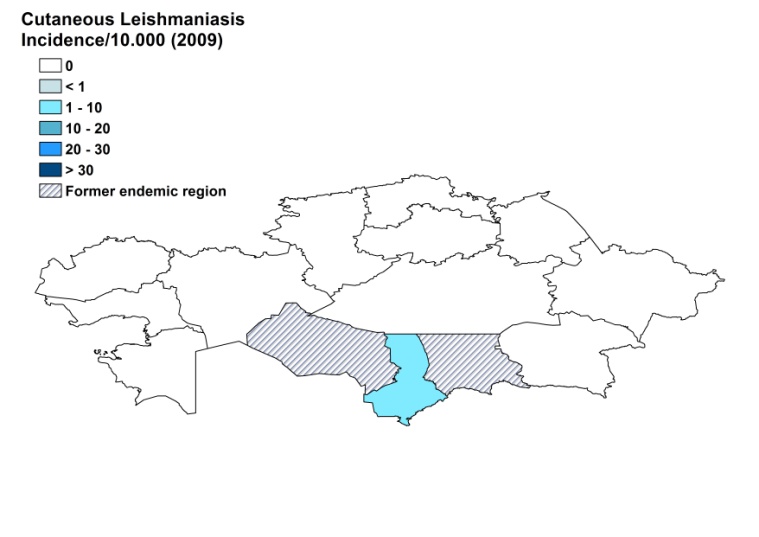

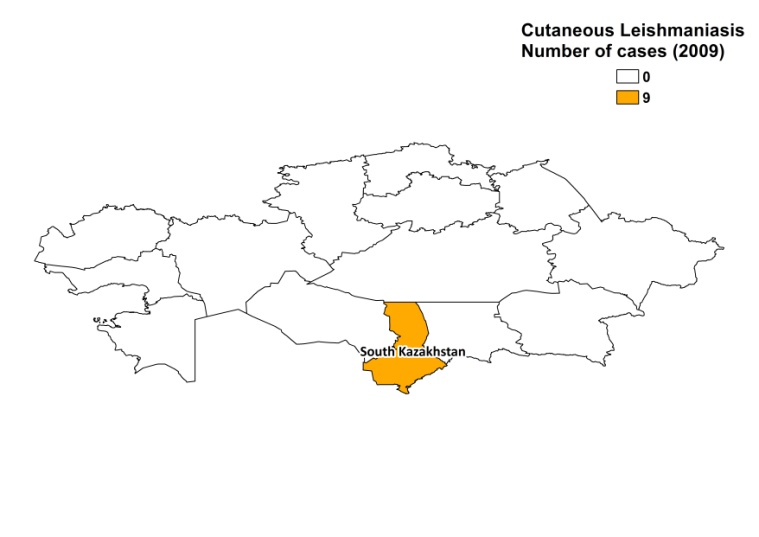
Cutaneous leishmaniasis**

**Visceral leishmaniasis cases**

| 2001 | 2002 | 2003 | 2004 | 2005 | 2006 | 2007 | 2008 |
| --- | --- | --- | --- | --- | --- | --- | --- |
| 2 | 1 | 2 | 2 | 0 | 2 | 0 | 0 |

**Cutaneous leishmaniasis trend**

**CONTROL**

The notification of leishmaniasis is mandatory in the country. There is no national leishmaniasis control program. New guidelines for control, prophylaxis and diagnosis were published in 2007 and for diagnosis in 2009. There is a leishmaniasis vector control program. No bednets are being distributed, but insecticide spraying is done regularly. There is a leishmaniasis reservoir control program; rodent control is performed regularly, by food poisoning, and in the southern regions, stray animals are caught.

**DIAGNOSIS, TREATMENT**

**Diagnosis**

VL and CL: parasitological confirmation by microscopic examination of tissue samples.

**Treatment**

VL and CL: antimonials, 20 mg Sb^v^/kg/day. Cure rate for both forms is 100%. For CL, erythromycin, doxycycline and metronidazole are used in absence of antimonials.

**ACCESS TO CARE**

Care for leishmaniasis is provided for free. However, access to treatment is problematic. Leishmaniasis can only be diagnosed and treated in specialized hospitals in the public sector. This forms a barrier for access to care, as patients often live in remote areas with no health facilities and transport. Furthermore, due to a lack of awareness of the disease, and the lack of available effective treatment, patients may not seek treatment in time or at all.

**ACCESS TO DRUGS**

Only doxycycline and metronidazole are included in the National Essential Drug List for CL. Antimonials for treatment are not registered and unavailable in the country. They can only be obtained at the French Embassy (Glucantime, Sanofi), which causes long delays in treatment.

**SOURCES OF INFORMATION**

- Dr Shapiyeva, Republican Sanitary and Epidemiology Station
