# Supplementary material for: Leishmaniasis Worldwide and Global Estimates of Its Incidence
Source: PLoS One. 2012 May 31;7(5):e35671. doi: 10.1371/journal.pone.0035671 (PMC3365071; doi:10.1371/journal.pone.0035671)
Supplement: Text S50 — Leishmaniasis Country Profiles, Kenya. (DOCX) [file pone.0035671.s050.docx]

**KENYA**


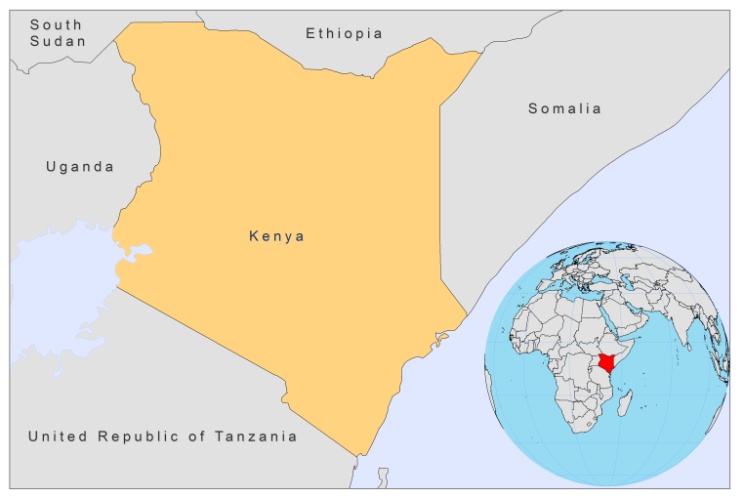


**BASIC COUNTRY DATA**

Total Population: 40,512,682

Population 0-14 years: 42%

Rural population: 78%

Population living under USD 1.25 a day: 19.7%

Population living under the national poverty line: no data

Income status: Low income economy

Ranking: Low human development (ranking 143)

Per capita total expenditure on health at average exchange rate (US dollar): 33

Life expectancy at birth (years): 56

Healthy life expectancy at birth (years): 44

**BACKGROUND INFORMATION**

VL seems to have been imported in Kenya by soldiers returning from southern Ethiopia after the Second World War. The disease spread with epidemic resurgences in different foci during the years that followed. Since 1980, the number of reported cases increased considerably and expanded into new areas. In Baringo and Pokot area, there is a continuous high endemicity, which affects the very poor tribal nomadic population [1]. In other areas, VL occurs in outbreaks that are associated with periods of drought, when the rate of malnutrition is high. The outbreaks are probably related to people movement from high to low endemic areas due to the lack of food security. In 2000-2001, a VL outbreak with over 349 suspected cases was confirmed in the eastern and northeastern provinces of the Wajir and Mandera districts [2]. In 2006, another outbreak of VL was reported in Wajir and Isiolo districts, with a total of 82 suspected cases (48 were confirmed), followed in 2008 by another outbreak, in the same area, with 75 confirmed cases.

Risk factors for VL have been studied and a good correlation was established between the proximity of houses or temporary settlements to termite hills and the risk of transmission. Having a low socio-economic status and treating livestock with insecticide were also identified as risk factors for VL. Sleeping near animals, owning a mosquito net and knowing about VL symptoms were associated with a reduced risk of VL [3]. Transmission is believed to be mainly anthroponotic.

CL caused by *L. aethiopica* is well known in the mountainous regions, such as Mount EIgon, and in the Rift valley escarpments. With the extension of farming and grazing in the region, there is an increased risk of cases since farmers and shepherds tend to sleep in caves where the vector is present [1].

CL is caused by *L. major* in the lowlands of Baringo district. Both VL and CL mainly affect children and young adults.

Two cases of HIV-*Leishmania* co-infection have been reported.

**PARASITOLOGICAL INFORMATION**

| ***Leishmania* species** | **Clinical form** | **Vector species** | **Reservoirs** |
| --- | --- | --- | --- |
| *L. tropica* | ZCL | *P. guggisbergi* | *Procavia capensis* |
| *L. aethiopica* | ZCL, DCL | *P. pedifer, P. aculeatus* | *Procavia capensis, Dendohyrax arboreous, Cricetomys sp.* |
| *L. major* | ZCL | *P. duboscqi* | *Tatera sp. Aethomys sp., Arvicanthis sp., Meriones sp.* |
| *L. donovani* | VL, PKDL | *P. martini, P. celiae,*  *P. vansomerenae* |  |

**MAPS AND TRENDS**

**Visceral leishmaniasis**

**
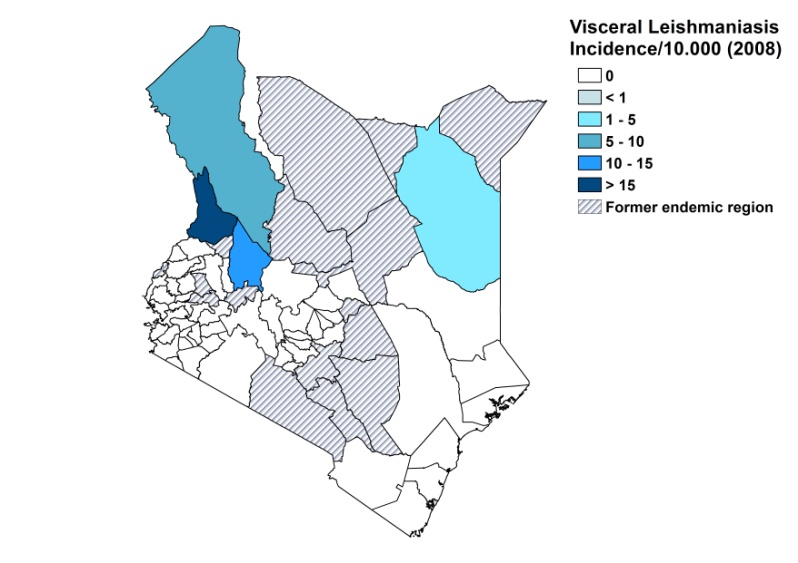

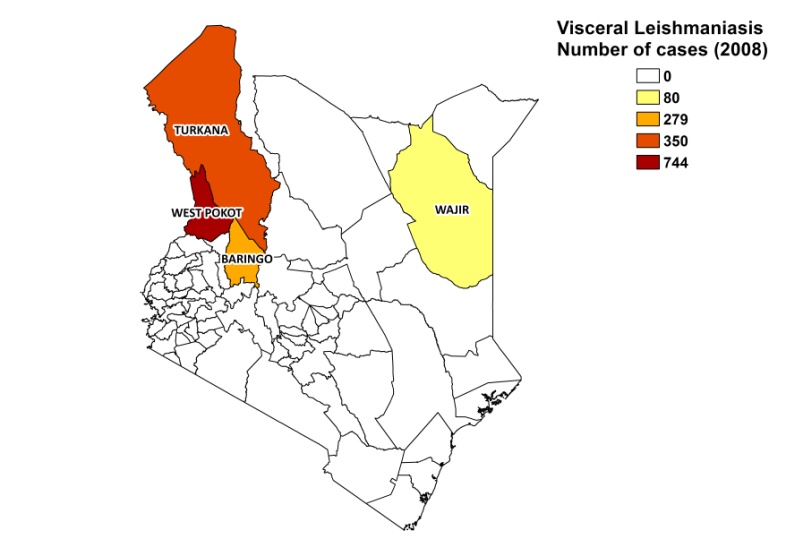
**

**Cutaneous leishmaniasis**

**
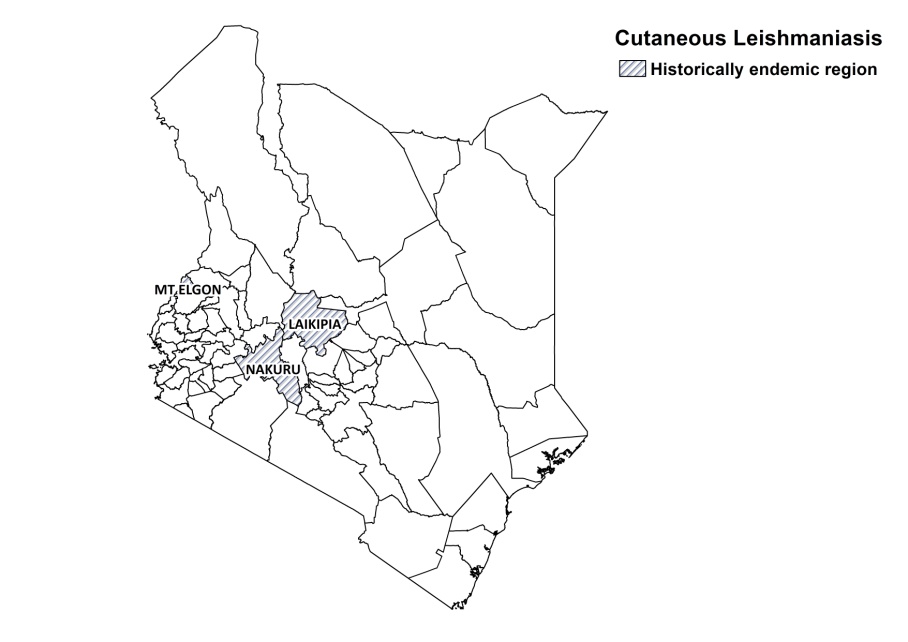
**

**Visceral leishmaniasis trend**

**CONTROL**

The notification of leishmaniasis is not mandatory in the country and there is no national leishmaniasis control program (it is planned for 2010 for VL and in 2012 for CL, depending on funding). Active human case detection is regularly performed in order to recruitment patients for clinical studies. There is no leishmaniasis vector control program, but in the east of the country, the case load was substantially reduced by the use of cloth sheets impregnated with insecticide (*Mbu cloth*) that were fixed on the inner walls of the rooms; and by the destruction of the breeding sites (termites hills and animals burrows) of the farming related vectors. There is a bednet distribution program in the context of malaria control, but insecticide spraying is not regularly performed.

**DIAGNOSIS, TREATMENT**

**Diagnosis**

VL: microscopic examination of spleen aspirate or DAT.

CL: clinical grounds, confirmation by microscopic examination of skin lesion.

**Treatment**

VL: antimonials, 20 mg Sb^v^/kg/day for 30 days. Cure rate is > 95%. Recurring disease occurs in 3% of cases and PKDL in 2%. Severe adverse events occur in 3% of cases. Case fatality rate is 0%. Second line: conventional amphotericin B, 1 mg/kg/day on alternate days for 15 days or liposomal amphotericin B (AmBisome).

CL: antimonials, 20 mg Sb^v^/kg/day for 40-60 days, sometimes up to 120 days. Cure rate is 80%. Allopurinol is also used.

**ACCESS TO CARE**

Care for leishmaniasis is not provided for free. Due to the cost of antimonials purchased by the government (Pentostam, GSK), there is a cost sharing scheme in place, where patients have to contribute 1,000 KSH (12-13 USD) for treatment. Most patients are extremely poor and cannot afford this. However, considerable amounts of patients received free care through NGOs in 2007 and 2008 (MSF and Merlin), and at the KEMRI research institute (in the context of clinical trials).

There is no continuous drug supply in public health facilities. The Ministry of Health supplied 300 vials of Pentostam in 2008, which was not sufficient for the number of patients reported in public health facilities that year. WHO and UNICEF donated additional quantities of Pentostam in 2008 and 2009.

Apart from programs offered by NGOs, diagnosis of VL only takes place in hospitals, although treatment with antimonials can be continued on an out-patient base in health centers. Diagnosis of CL is only done at KEMRI research institute. As many patients live in remote areas, it is very difficult or impossible to reach treatment sites. They generally suffer major economic loss by missed working days while staying away from home in order to receive treatment.

As access to care is problematic, many patients resort to traditional healers before reporting to the hospital.

The private sector is not used for obtaining diagnosis and treatment.

**ACCESS TO DRUGS**

Sodium stibogluconate and pentamidine are included in the National Essential Drug List for the treatment of VL. Pentostam (GSK) is registered in Kenya, and generic SSG (Albert David, India) as well. Pentostam is available in pharmacies for 157 USD per bottle. Generic SSG is not available at private pharmacies. AmBisome is not registered and not available in Kenya.

**SOURCES OF INFORMATION**

- Dr Monique Wasunna. Kenya Medical Research Institute and Drugs for Neglected Diseases Initiative.
- Dr Davis Wachira. Division of Vector Borne and Neglected Tropical Diseases. Ministry of Health.

1. Desjeux P (1991). Information on the epidemiology and control of the leishmaniases by country or territory. WO/LEISH/91.31.

2. Marlet M, Sang D, Ritmeijer K, Muga R, Onsongo J et al (2003). Emergence and re-emergence of visceral leishmaniasis in areas of Somalia, north-eastern Kenya, and south-eastern Ethiopia in 2000-01. Trans Roy Soc Trop Med Hyg 97: 515-518.

3. Kolaczinski JH, Reithinger R, Worku DT, Ocheng A, Kasimiro J et al (2008). [Risk factors of visceral leishmaniasis in East Africa: a case-control study in Pokot territory of Kenya and Uganda](http://www.ncbi.nlm.nih.gov/pubmed/18184669). Int J Epidemiol 37(2):344-52. Epub 2008 Jan 9.
