# Supplementary material for: Leishmaniasis Worldwide and Global Estimates of Its Incidence
Source: PLoS One. 2012 May 31;7(5):e35671. doi: 10.1371/journal.pone.0035671 (PMC3365071; doi:10.1371/journal.pone.0035671)
Supplement: Text S51 — Leishmaniasis Country Profiles, Kuwait. (DOCX) [file pone.0035671.s051.docx]

**KUWAIT**


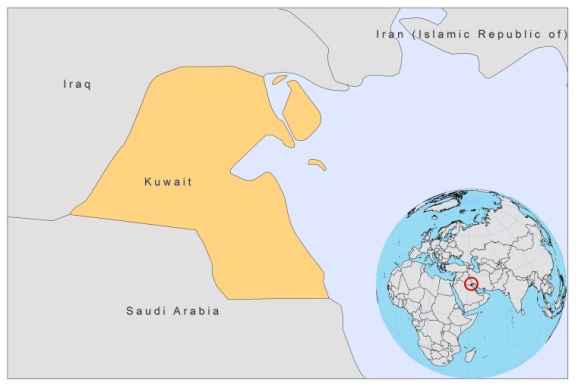


**BASIC COUNTRY DATA**

Total Population: 2,736,732

Population 0-14 years: 27%

Rural population: 2%

Population living under USD 1.25 a day: no data

Population living under the national poverty line: no data

Income status: High Income economy

Ranking: High human development (ranking 63)

Per capita total expenditure on health at average exchange rate (US dollar): 1,416

Life expectancy at birth (years): 74

Healthy life expectancy at birth (years): 67

**BACKGROUND**

More than one million immigrants work in Kuwait and the majority come from VL endemic countries such as India, Bangladesh and Nepal; and CL endemic countries such as Syrian Arab Republic, Iran (Islamic Republic of) and Afghanistan. VL and CL are reported in Kuwait, but mainly as an imported disease. VL is not endemic in Kuwait and imported cases occur sporadically in migrant workers [1]. As the vector is present, there is a theoretical risk of VL spreading within Kuwait [2]. CL may be endemic, although no infected reservoir was found in an extensive study [3]. There were no proven autochtonous cases until 1983 [3], but in 1991, CL was claimed to be endemic [4]. Hundreds of cases have been admitted and treated in hospitals in Kuwait [5], but most are thought to be imported. CL is also occasionally reported in returning military personnel. Two returning US soldiers were thought to have contracted CL in Kuwait, near the Iraqi border [6].

**PARASITOLOGICAL INFORMATION**

| ***Leishmania* species** | **Clinical form** | **Vector species** | **Reservoirs** |
| --- | --- | --- | --- |
| *L major* | ZCL | Unknown | Unknown |

**NO FURTHER INFORMATION IS AVAILABLE**

**SOURCES OF INFORMATION**

1. [Iqbal J](http://www.ncbi.nlm.nih.gov/pubmed?term=%22Iqbal%20J%22%5BAuthor%5D), [Hira PR](http://www.ncbi.nlm.nih.gov/pubmed?term=%22Hira%20PR%22%5BAuthor%5D), [Saroj G](http://www.ncbi.nlm.nih.gov/pubmed?term=%22Saroj%20G%22%5BAuthor%5D), [Philip R](http://www.ncbi.nlm.nih.gov/pubmed?term=%22Philip%20R%22%5BAuthor%5D), [Al-Ali F](http://www.ncbi.nlm.nih.gov/pubmed?term=%22Al-Ali%20F%22%5BAuthor%5D), et al (2002). Imported Visceral Leishmaniasis: Diagnostic Dilemmas and Comparative Analysis of Three Assays. J Clin Microbiol 40 (2): 475–479.

2. Cope SE, Schultz GW, Richards AL, Savage HM, Smith GC et al (1996). Assessment of arthropod vectors of infectious diseases in areas of U.S. troop deployment in the Persian Gulf. Am J Trop Med Hyg 54:49–53.

3. [Hussein MS](http://www.ncbi.nlm.nih.gov/pubmed?term=%22Hussein%20MS%22%5BAuthor%5D) (1983). The epidemiology of cutaneous leishmaniasis in Kuwait. [Ann Trop Med Parasitol](http://www.ncbi.nlm.nih.gov/pubmed/6882053) 77(1):27-33.

4. [al-Fouzan AS](http://www.ncbi.nlm.nih.gov/pubmed?term=%22al-Fouzan%20AS%22%5BAuthor%5D), [al Saleh QA](http://www.ncbi.nlm.nih.gov/pubmed?term=%22al%20Saleh%20QA%22%5BAuthor%5D), [Najem NM](http://www.ncbi.nlm.nih.gov/pubmed?term=%22Najem%20NM%22%5BAuthor%5D), [Rostom A (1991)](http://www.ncbi.nlm.nih.gov/pubmed?term=%22Rostom%20AI%22%5BAuthor%5D). Cutaneous leishmaniasis in Kuwait. Clinical experience with itraconazole. [Int J Dermatol](http://www.ncbi.nlm.nih.gov/pubmed/1663089) 30(7):519-21.

5. [Al-Mutairi N](http://www.ncbi.nlm.nih.gov/pubmed?term=%22Al-Mutairi%20N%22%5BAuthor%5D), [Alshiltawy M](http://www.ncbi.nlm.nih.gov/pubmed?term=%22Alshiltawy%20M%22%5BAuthor%5D), [El Khalawany M](http://www.ncbi.nlm.nih.gov/pubmed?term=%22El%20Khalawany%20M%22%5BAuthor%5D), [Joshi A](http://www.ncbi.nlm.nih.gov/pubmed?term=%22Joshi%20A%22%5BAuthor%5D), [Eassa BI](http://www.ncbi.nlm.nih.gov/pubmed?term=%22Eassa%20BI%22%5BAuthor%5D), et al (2009). Tropical medicine rounds: Treatment of Old World cutaneous leishmaniasis with dapsone, itraconazole, cryotherapy, and imiquimod, alone and in combination. [Int J Dermatol](http://www.ncbi.nlm.nih.gov/pubmed/19673049) 48(8):862-9.

6. Cutaneous leishmaniasis in U.S. military personnel--Southwest/Central Asia, 2002-2003. [Centers for Disease Control and Prevention (CDC)](http://www.ncbi.nlm.nih.gov/pubmed?term=%22Centers%20for%20Disease%20Control%20and%20Prevention%20%28CDC%29%22%5BCorporate%20Author%5D). [MMWR Morb Mortal Wkly Rep.](http://www.ncbi.nlm.nih.gov/pubmed/14574274) 2003; 24;52(42):1009-12.
