# Supplementary material for: Leishmaniasis Worldwide and Global Estimates of Its Incidence
Source: PLoS One. 2012 May 31;7(5):e35671. doi: 10.1371/journal.pone.0035671 (PMC3365071; doi:10.1371/journal.pone.0035671)
Supplement: Text S52 — Leishmaniasis Country Profiles, Kyrgyzstan. (DOCX) [file pone.0035671.s052.docx]

**KYRGYZSTAN**


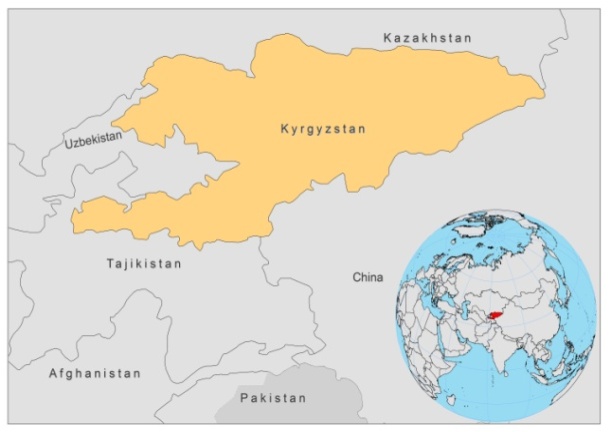


**BASIC COUNTRY DATA**

Total Population: 5,365,167

Population 0-14 years: 30%

Rural population: 63%

Population living under USD 1.25 a day: 1.9%

Population living under the national poverty line: no data

Income status: Low income economy

Ranking: Medium human development (ranking 126)

Per capita total expenditure on health at average exchange rate (US dollar): 57

Life expectancy at birth (years): 69

Healthy life expectancy at birth (years): 55

**BACKGROUND INFORMATION**

VL was first registered in Kyrgyzstan in 1939. During the period 1939 to 1960, 191 patients were reported in Osh city and 6 rural districts, with a peak in 1953 (22 cases). All patients were under 7 years of age. No more cases have been reported since 1968.

CL was also registered in Osh region with a peak (28 cases) in 1956. 60 CL cases were reported in total between 1950 and 1982. After 1982, CL was no more reported.

As both vector and reservoir are present in the country and neighboring countries are endemic, it is strongly suspected that both VL and CL cases still occur, but are never diagnosed. The awareness of leishmaniasis among doctors is very low and there are no trained laboratory workers to perform the diagnosis.

**PARASITOLOGICAL INFORMATION**

| ***Leishmania* species** | **Clinical form** | **Vector species** | **Reservoirs** |
| --- | --- | --- | --- |
| *L. infantum* | ZVL, CL | *P. longiductus* | *Canis familiaris* |

**MAPS AND TRENDS**

**Visceral leishmaniasis**

**
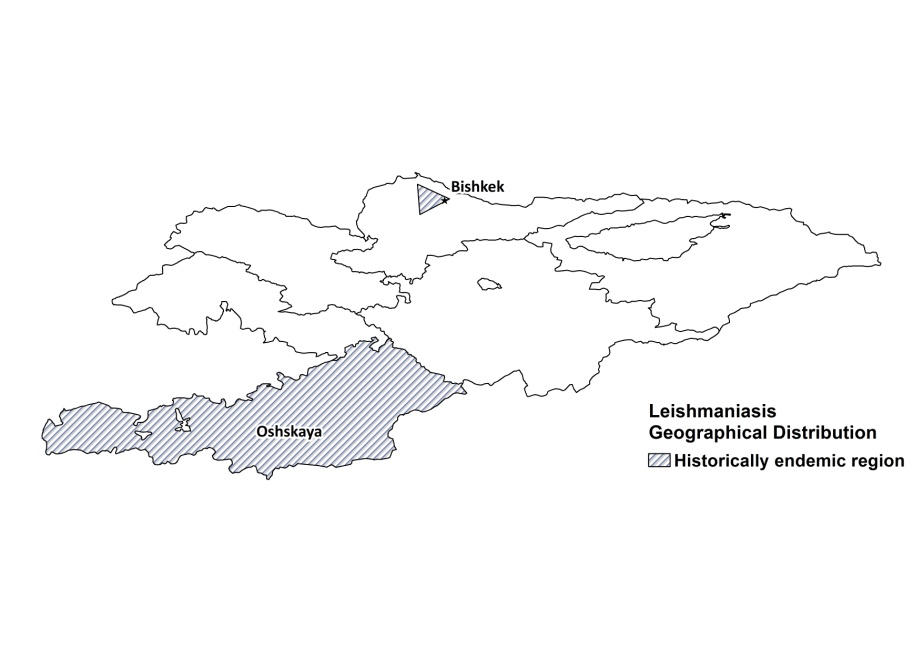
**

**Visceral leishmaniasis trend**

**CONTROL**

The notification of leishmaniasis is mandatory. There is no national leishmaniasis control program. There is no vector control program for leishmaniasis, but there is for malaria. There is no reservoir control program.

**DIAGNOSIS, TREATMENT**

**Diagnosis**

VL and CL cannot be diagnosed, as the expertise is missing in the country.

**Treatment**

In hospitals, Amphotericin B is used for treatment of systemic candidiasis and can also be used to treat VL and CL. In case of official notification of VL and CL, permission will be given for the use of antimonials.

**ACCESS TO CARE**

Health care is not provided for free. In case of leishmaniasis, patients will have to pay for hospitalization. This, plus the fact that awareness of leishmaniasis is very low, both form barriers to treatment access. Cases of leishmaniasis most likely occur, but they are either not recognized, or patients do not seek medical care due to its cost.

**ACCESS TO DRUGS**

Sodium stibogluconate (‘solyusurmin’) and furazolidon (‘aminohinol’) are included in the National Essential Drug List for the treatment of leishmaniasis. However, antimonials are not available, nor are they registered in the country. Amphotericin B is included in the list for candidiasis and can be used for the treatment of leishmaniasis.

**SOURCES OF INFORMATION**

- Dr Usubalieva Jumagul, Department of State Sanitary Epidemiological Surveillance of Ministry of Health.
- Drs Shavkat Razakov and Dmitriy Kovalenko, temporary consultants to WHO.
