# Supplementary material for: Leishmaniasis Worldwide and Global Estimates of Its Incidence
Source: PLoS One. 2012 May 31;7(5):e35671. doi: 10.1371/journal.pone.0035671 (PMC3365071; doi:10.1371/journal.pone.0035671)
Supplement: Text S53 — Leishmaniasis Country Profiles, Lebanon. (DOCX) [file pone.0035671.s053.docx]

**LEBANON**


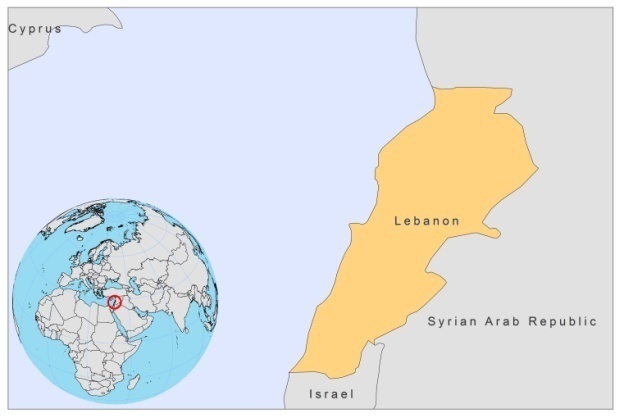


**BASIC COUNTRY DATA**

Total Population: 4,227,597

Population 0-14 years: 25%

Rural population: 13%

Population living under USD 1.25 a day: no data

Population living under the national poverty line: no data

Income status: Upper middle income economy

Ranking: High human development (ranking 71)

Per capita total expenditure on health at average exchange rate (US dollar): 663

Life expectancy at birth (years): 72

Healthy life expectancy at birth (years): 60

**BACKGROUND INFORMATION**

Sporadic cases of VL were recorded in western Lebanon, near the coast, among children under 10 years old between 1962 and 1964. No recent cases have been reported, but it is likely that a few cases of VL occur [1].

Known foci of CL used to exist. Cases of CL may still occur, but go unreported. Cutaneous leishmaniasis is sporadic in Lebanon and is mainly caused by *L. infantum*. Three of these stocks were isolated from patients living in the outskirts of Latakia (Syrian Arab Republic), where an outbreak occurred the previous year. This focus in Latakia could extend to the coastal areas of northern Lebanon, which have similar topography and probably similar biotope.

In 1993-1997, prevalence of leishmaniasis in Lebanon was studied in a population sample of about 81,000 subjects (60% rural and 40% urban), roughly constituting 3.4% of the total population. The prevalence of cutaneous leishmaniasis was 0.18% in the rural, versus 0.41% in the urban areas. All the affected communities were also affected by poverty, resulting in poor living conditions. Three cases of VL were detected [2].

*L. major* CL cases were diagnosed from a patient living in Homs, a dry and semi-arid region where *Psammomys obesus*, the suspected reservoir of *L. major,* is common.

**PARASITOLOGICAL INFORMATION**

| ***Leishmania* species** | **Clinical form** | **Vector species** | **Reservoirs** |
| --- | --- | --- | --- |
| *L. infantum* | ZVL, CL | *P. syriacus* | *Canis familiaris* |

**MAPS AND TRENDS**

**Visceral and cutaneous leishmaniasis**

**
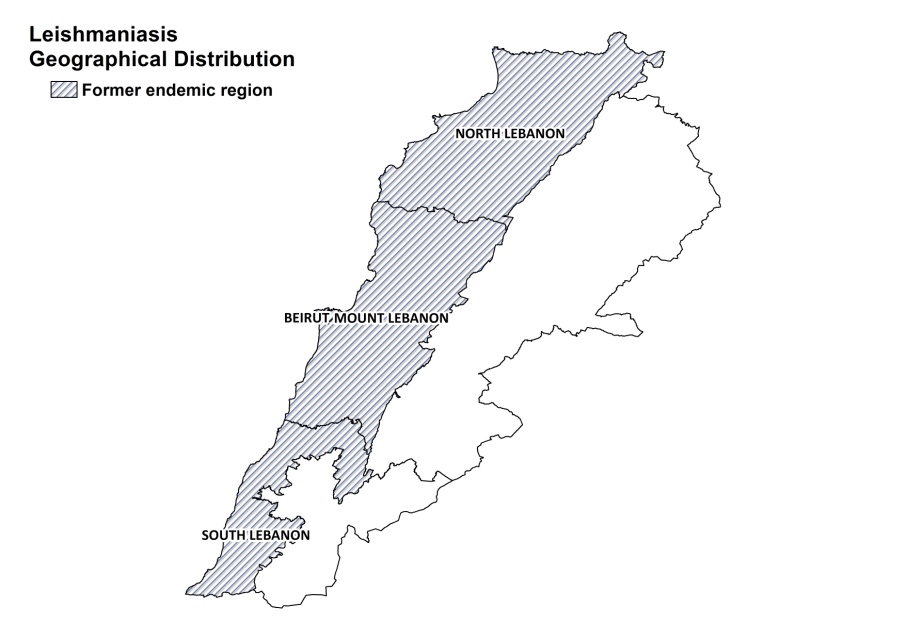
**

**Visceral and cutaneous leishmaniasis trends**

Not available.

**CONTROL**

Notification of leishmaniasis is mandatory in the country. There is no national leishmaniasis control program, but rodent control is regularly performed. Case detection is passive.

**DIAGNOSIS, TREATMENT**

**Diagnosis**

On clinical grounds.

**Treatment**

CL is treated with a mixture of antibiotics and steroids.

**ACCESS TO CARE**

Access to care, including care for leishmaniasis, is not free in Lebanon. Patients have to pay for consultation, drugs, lab tests and hospitalization.

**ACCESS TO DRUGS**

No drugs for leishmaniasis are included in the National Essential Drug list. No antimonials are registered in Lebanon.

**SOURCES OF INFORMATION**

1. Desjeux P (1991). Information on the epidemiology and control of the leishmaniases by country or territory.WHO/LEISH/91/30.

2. Nuwayri-Salti N, Baydoun E, El-Tawk R, Fakhoury R, Knio K (2000). The epidemiology of leishmaniases in Lebanon. Trans Roy Soc Trop Med Hyg 94:164-166.
