# Supplementary material for: Leishmaniasis Worldwide and Global Estimates of Its Incidence
Source: PLoS One. 2012 May 31;7(5):e35671. doi: 10.1371/journal.pone.0035671 (PMC3365071; doi:10.1371/journal.pone.0035671)
Supplement: Text S54 — Leishmaniasis Country Profiles, Libya. (DOCX) [file pone.0035671.s054.docx]

**LIBYA**


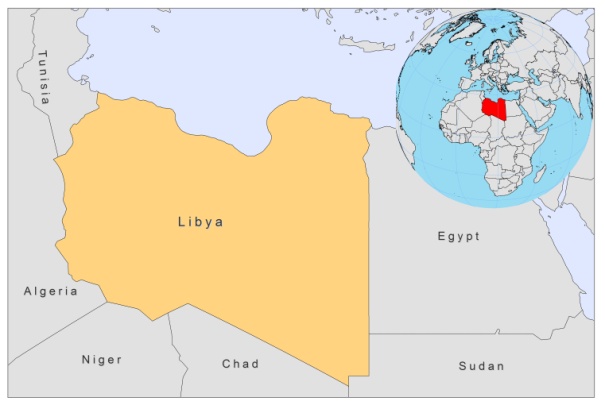


**BASIC COUNTRY DATA**

Total Population: 6,355,112

Population 0-14 years: 30%

Rural population: 22%

Population living under USD 1.25 a day: no data

Population living under the national poverty line: no data

Income status: Upper middle income economy

Ranking: High human development (ranking 64)

Per capita total expenditure on health at average exchange rate (US dollar): 417

Life expectancy at birth (years): 75

Healthy life expectancy at birth (years): 64

**BACKGROUND INFORMATION**

*Leishmania infantum* VL has been known since 1910, with a few sporadic cases from the southern and northeastern regions of the country. A total of 153 cases have been recorded in the last 4 decades. There is little information about the epidemiology of VL in Libya [1].

ZCL is endemic with occasional outbreaks. The estimated total number of cases between 1971 and 2009 was over 55,000. In 2006, over 7,000 cases were recorded in 8 districts, with an outbreak of 3,961 cases in Musrata and the disease spread to non endemic areas [2]. That same year, a control program was started and the number of cases decreased to 1,800 in 2008. In 2009, the prevalence of CL was 7.1/10,000 population. Risk factors were identified: the occupational exposure to infection of outdoor workers, a lack of knowledge on how to prevent infection, the closeness of animal shelters to houses, and increased soil moisture. Underreporting of CL is a problem, and is due to a lack of public awareness of the disease.

No cases of HIV-*Leishmania* co-infection have been reported.

**PARASITOLOGICAL INFORMATION**

| ***Leishmania* species** | **Clinical form** | **Vector species** | **Reservoirs** |
| --- | --- | --- | --- |
| *L. infantum* | ZVL, CL | *P. syriacus* | *Canis familiaris* |
| *L. killicki* | CL | unknown |  |
| *L. major* | ZCL | *P. papatasi* | *Psammomys obesus, Meriones spp.* |

**MAPS AND TRENDS**

**Visceral leishmaniasis**

**
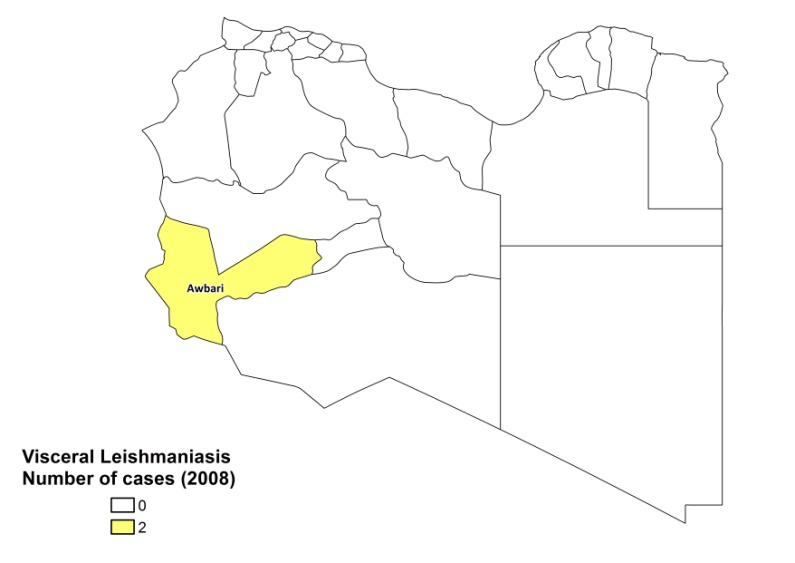

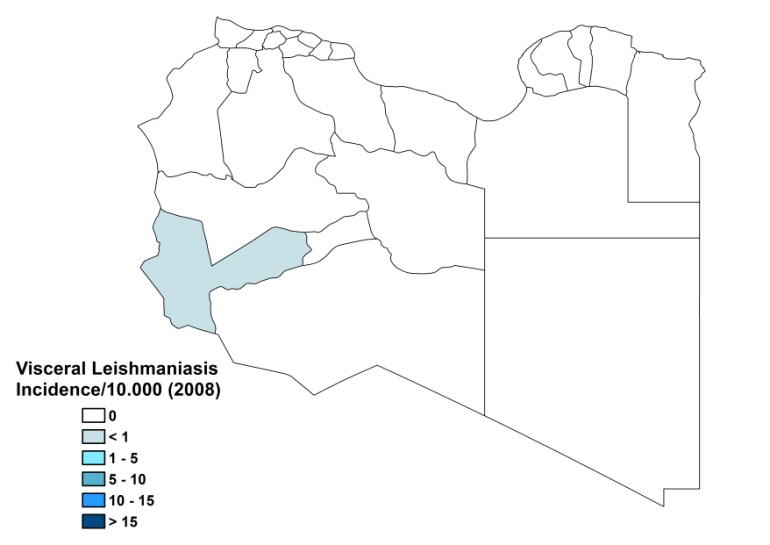
**

**Cutaneous leishmaniasis**

**
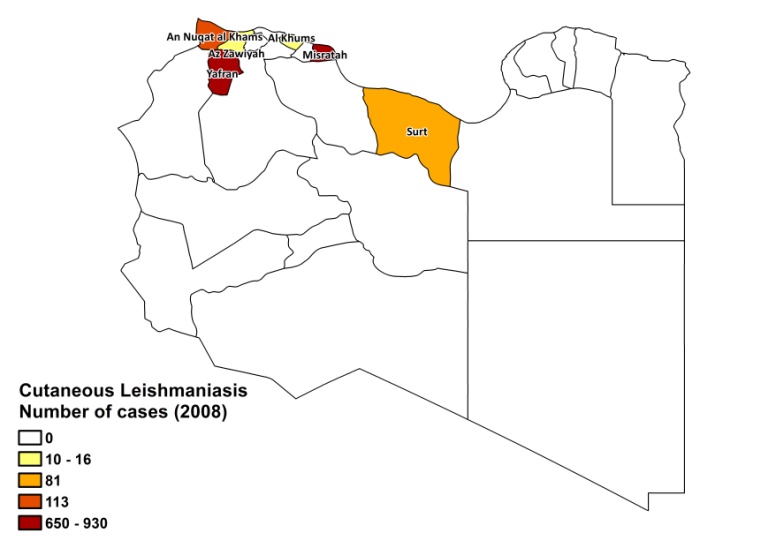
**

**
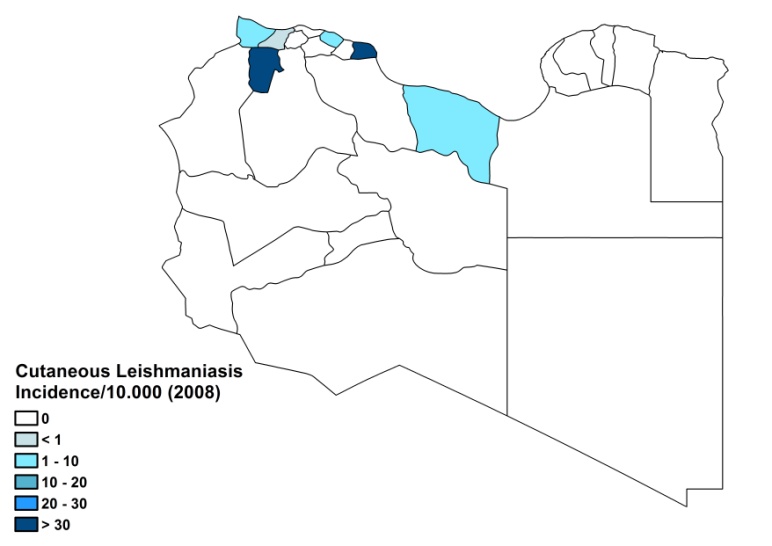
**

**Number of visceral leishmaniasis cases**

| 2007 | 2008 |
| --- | --- |
| 2 | 3 |

**Cutaneous leishmaniasis trend**

**CONTROL**

Leishmaniasis notification is mandatory and a national control program for CL has been in place since 2006. Case detection is passive. There is a vector control program that involves insecticide spraying and regular rodent control is performed.

**DIAGNOSIS, TREATMENT**

**Diagnosis**

CL: on clinical grounds (85%), microscopic examination of skin lesion sample (10%) and isotyping or PCR (5%).

VL: confirmation by microscopic examination of bone marrow aspirate. PCR is possible in specialized hospitals and isotyping or PCR (5%).

**Treatment**

VL: antimonials, 10-20 mg Sb^v^/kg/day. Cure rate is 96%, with a fatality rate of 4%.

CL: antimonials, intralesional or systemic, 10-20 mg Sb^v^/kg/day. Cure rate is 98% with recurring lesions in 2% of cases.

**ACCESS TO CARE**

Care for CL is offered for free through the 15 leishmaniasis clinics established in the endemic areas by the National Control program. VL is only diagnosed and treated in regional and specialized hospitals. The government supplied sufficient antimonials (Pentostam, GSK) for all patients in 2008. Approximately 5% of CL patients seek care in the private sector. All patients are thought to have access to care for leishmaniasis in Libya.

**ACCESS TO DRUGS**

No other drug than sodium stibogluconate is included in the National Essential Drug List. No antimonials are registered in Libya, but are available at private pharmacies (10 USD/vial of Glucantime, Sanofi, leading to a treatment cost of around 1,100 USD for a course of intralesional injection).

**SOURCES OF INFORMATION**

• Dr Badereddin Annajar. National program for Leishmaniasis Control, Ministry of Health, Libya.

1. Mehabresh MI (1994). [Visceral leishmaniasis: new foci of infection in Libya.](http://www.ncbi.nlm.nih.gov/pubmed/7932924) J Trop Med Hyg 97(5):282-5.

2. el-Buni AA, Jabeal I, Ben-Darif AT (2000). [Cutaneous leishmaniasis in the Libyan Arab Jamahiriya: a study of the Yafran area.](http://www.ncbi.nlm.nih.gov/pubmed/12197345) East Mediterr Health J 6(5-6):884-7.
