# Supplementary material for: Leishmaniasis Worldwide and Global Estimates of Its Incidence
Source: PLoS One. 2012 May 31;7(5):e35671. doi: 10.1371/journal.pone.0035671 (PMC3365071; doi:10.1371/journal.pone.0035671)
Supplement: Text S55 — Leishmaniasis Country Profiles, Malawi. (DOCX) [file pone.0035671.s055.docx]

**MALAWI**

**
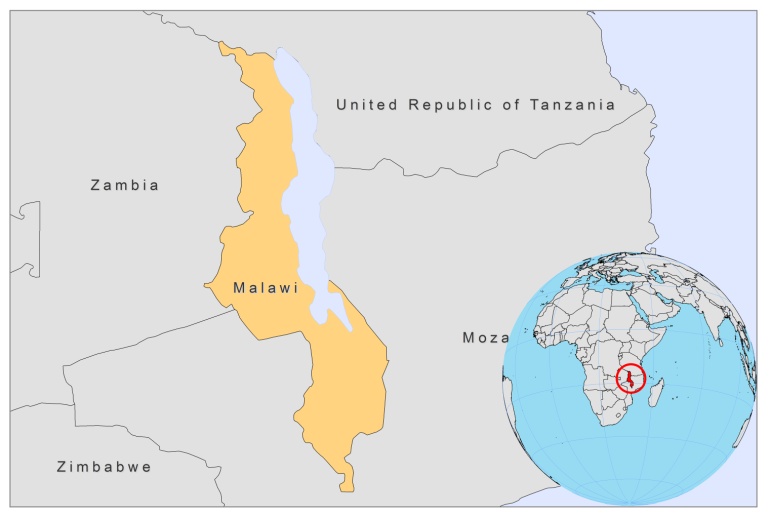
**

**BASIC COUNTRY DATA**

Total Population: 14,900,841

Population 0-14 years: 46%

Rural population: 80%

Population living under USD 1.25 a day: no data

Population living under the national poverty line: no data

Income status: Low income economy

Ranking: Low human development (ranking 171)

Per capita total expenditure on health at average exchange rate (US dollar): 19

Life expectancy at birth (years): 53

Healthy life expectancy at birth (years): 35

**BACKGROUND INFORMATION**

Leishmaniasis is rare in Malawi. The first case of autochtonous VL caused by *L.donovani* was reported in 1979 in a 38-year-old male [1].

Leishmaniasis was diagnosed -post mortem- in 2 cases in Northern Malawi [2]. One was suspected VL in an 18-months-old malnourished child, with wide spread rash, in 1989; the other was CL in an HIV-1 positive adult, with skin lesions that had been present for 6 months, in the late 1990s. Both patients had a history of travel to the United Republic of Tanzania. No further cases have been documented.

**PARASITOLOGICAL INFORMATION**

| ***Leishmania* species** | **Clinical form** | **Vector species** | **Reservoirs** |
| --- | --- | --- | --- |
| *Unknown* | VL, CL | Unknown | Unknown |

**MAPS AND TRENDS, CONTROL, DIAGNOSIS, TREATMENT, ACCESS TO CARE, ACCESS TO DRUGS**

No information available.

No antimonials are registered.

**SOURCES OF INFORMATION**

1. [Knowles JK](http://www.ncbi.nlm.nih.gov/pubmed?term=%22Knowles%20JK%22%5BAuthor%5D), [Paul B](http://www.ncbi.nlm.nih.gov/pubmed?term=%22Paul%20B%22%5BAuthor%5D), [Hutt MS](http://www.ncbi.nlm.nih.gov/pubmed?term=%22Hutt%20MS%22%5BAuthor%5D), [Lucas S](http://www.ncbi.nlm.nih.gov/pubmed?term=%22Lucas%20S%22%5BAuthor%5D) (198). A case of visceral leishmaniasis in Malawi. [Trans R Soc Trop Med Hyg](javascript:AL_get(this,%20'jour',%20'Trans%20R%20Soc%20Trop%20Med%20Hyg.');) 75(3):474-5.

2. [Pharoah PD](http://www.ncbi.nlm.nih.gov/pubmed?term=%22Pharoah%20PD%22%5BAuthor%5D), [Ponnighaus JM](http://www.ncbi.nlm.nih.gov/pubmed?term=%22Ponnighaus%20JM%22%5BAuthor%5D), [Chavula D](http://www.ncbi.nlm.nih.gov/pubmed?term=%22Chavula%20D%22%5BAuthor%5D), [Lucas SB](http://www.ncbi.nlm.nih.gov/pubmed?term=%22Lucas%20SB%22%5BAuthor%5D) (1993). Two cases of cutaneous leishmaniasis in Malawi. [Trans R Soc Trop Med Hyg](javascript:AL_get(this,%20'jour',%20'Trans%20R%20Soc%20Trop%20Med%20Hyg.');) 87(6):668-70.
