# Supplementary material for: Leishmaniasis Worldwide and Global Estimates of Its Incidence
Source: PLoS One. 2012 May 31;7(5):e35671. doi: 10.1371/journal.pone.0035671 (PMC3365071; doi:10.1371/journal.pone.0035671)
Supplement: Text S56 — Leishmaniasis Country Profiles, Mali. (DOCX) [file pone.0035671.s056.docx]

**MALI**

**
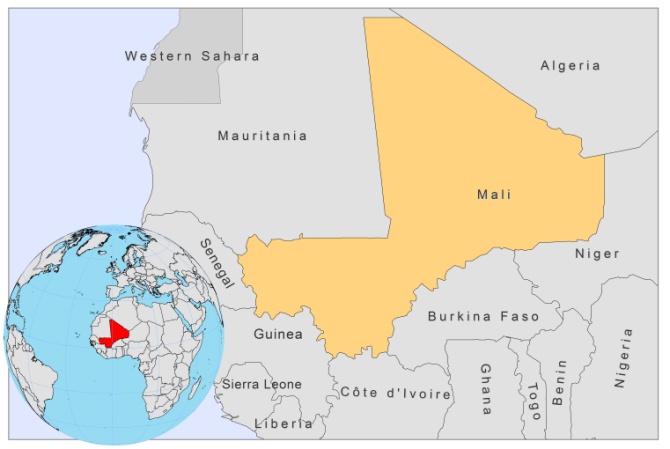
**

**BASIC COUNTRY DATA**

Total Population: 15,369,809

Population 0-14 years: 47%

Rural population: 67%

Population living under USD 1.25 a day: 51.4%

Population living under the national poverty line: 47.4%

Income status: Low income economy

Ranking: Low human development (ranking 175)

Per capita total expenditure on health at average exchange rate (US dollar): 38

Life expectancy at birth (years): 51

Healthy life expectancy at birth (years): 38

**BACKGROUND INFORMATION**

VL is unknown in Mali. CL was first reported in 1948 [1]. Since then, 2 principal endemic areas have been identified: Nioro, in the region of Kayes, in south-western Mali, and Segou (Segou region). Between 1958 and 1966, 589 cases of CL were reported: 70% of which were from the Kayes area (413 cases). In the same period, leishmanin skin test surveys were undertaken across the country and among 1,649 subjects, the global positivity rate was 18.6%. Only 14 cases were reported from 1973 to 1977. During the last 12 years (1997-2008), 650 cases of CL have been reported, the annual number of cases ranging from 18 to 86. This is expected to be far less than the actual number of cases. Seasonal spikes of cases are observed during the months of October and November.

In 2009, a leishmanin skin test survey showed a high prevalence of infection (45.4%) in Kemena, a village in central Mali. There was a marked difference in the prevalence and annual incidence of *Leishmania* factors in Sougoula, a neighboring village [2]. This difference could be explained by the different availability of infected reservoirs in each focus. While the presence of *P. duboscqi* sandflies in Mali is well established [3], the reservoir for CL has not yet been identified [4].

Between 1997 and 2001, 6 HIV/*Leishmania* coinfected patients have been reported (2.4%).

**PARASITOLOGICAL INFORMATION**

| ***Leishmania* species** | **Clinical form** | **Vector species** | **Reservoirs** |
| --- | --- | --- | --- |
| *L. major* | ZCL | *P. duboscqi* | unknown |

**MAPS AND TRENDS**

**Cutaneous leishmaniasis**

**
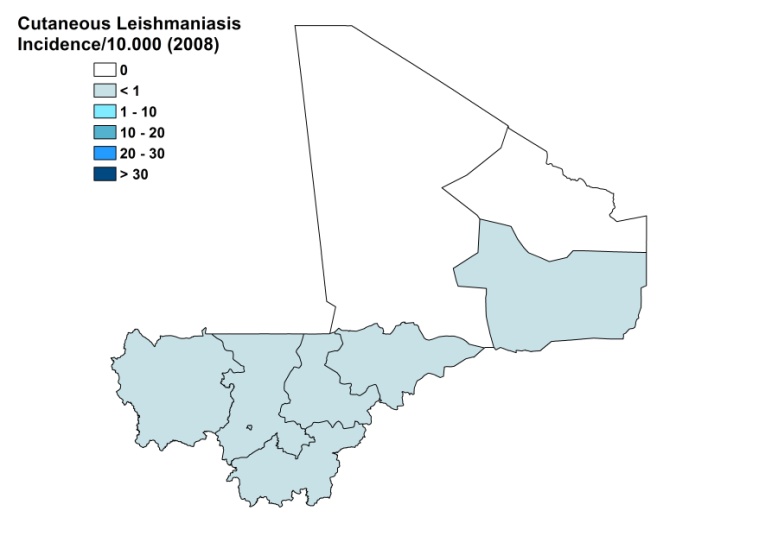

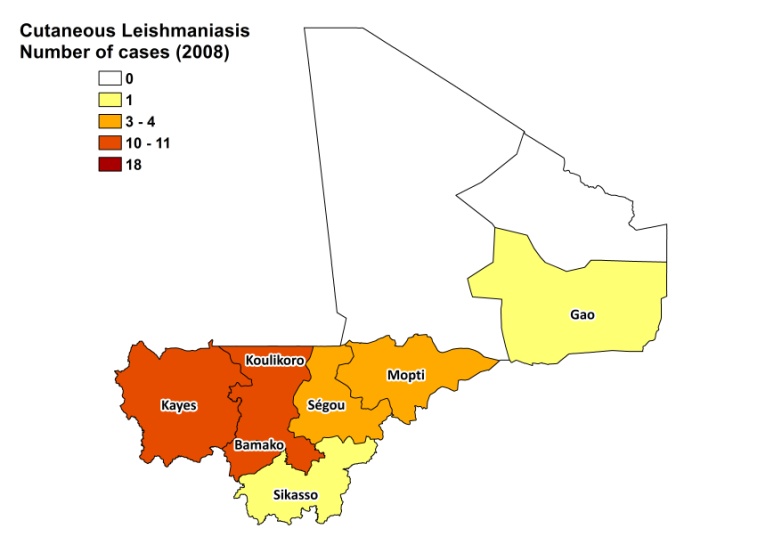
**

**Cutaneous leishmaniasis trend**

**CONTROL**

The notification of leishmaniasis has been mandatory in the country since 1960. There is no national leishmaniasis control program, nor a leishmaniasis vector control program. Bednets distribution and insecticide spraying take place in the context of malaria control. There is no leishmaniasis reservoir control program.

**DIAGNOSIS, TREATMENT**

**Diagnosis**

CL: on clinical grounds and confirmation by microscopic examination of skin lesion sample; sometimes PCR is used.

**Treatment**

CL: antimonials, intralesionsal and systemic in case of severe lesions. Thermotherapy is also used for patients with limited number of lesions on limbs.

**ACCESS TO CARE**

Care for leishmaniasis is not provided for free and the Ministry of Health does not provide drugs for leishmaniasis. Patients have to pay for drugs, tests and treatment themselves. In 2007, 120 patients were treated by an NGO, but currently, no NGO’s treating leishmaniasis are present. The main reason for lack of access to treatment is the unavailability of antimonials in the country. There is also a lack of awareness of the disease among health workers and the population. Most patients seek substandard private care. Diagnosis and treatment only takes place in one specialized center (Centre National d’Appui à la lutte contre la Maladie, CNAM), which is inaccessible for most patients due to distance, lack of money for transport and economic loss caused by spending time away from home.

**ACCESS TO DRUGS**

Meglumine antimoniate is included in the National Essential Drug List, but no antimonials are registered in Mali. Meglumine antimoniate (Glucantime, Sanofi) is available in private pharmacies for USD 57 per box of 5 vials, leading to a treatment cost of around USD 125 for intralesional treatment, and was only found in 2 out of 200 pharmacies.

**SOURCES OF INFORMATION**

- Dr Sibiry Samake, Faculté de Médicine, de Pharmacie et d'Odonto-Stomatologie, Mali. *Consultative Meeting on The Control of Leishmaniasis in the African Region. WHO/AFRO Addis Ababa, 23-25 Feb 2010.*

1. Lefrou G (1948). La Leishmaniose cutanee au Soudan francais. Frequence de la forme seche papulo-tuberculeuse. Bull Soc Path Exot 41:622-7.

2. [Oliveira F](http://www.ncbi.nlm.nih.gov/pubmed?term=%22Oliveira%20F%22%5BAuthor%5D), [Doumbia S](http://www.ncbi.nlm.nih.gov/pubmed?term=%22Doumbia%20S%22%5BAuthor%5D), [Anderson JM](http://www.ncbi.nlm.nih.gov/pubmed?term=%22Anderson%20JM%22%5BAuthor%5D), [Faye O](http://www.ncbi.nlm.nih.gov/pubmed?term=%22Faye%20O%22%5BAuthor%5D), [Diarra SS](http://www.ncbi.nlm.nih.gov/pubmed?term=%22Diarra%20SS%22%5BAuthor%5D), [Traoré P](http://www.ncbi.nlm.nih.gov/pubmed?term=%22Traor%C3%A9%20P%22%5BAuthor%5D) et al (2009). [Discrepant prevalence and incidence of Leishmania infection between two neighboring villages in Central Mali based on Leishmanin skin test surveys.](http://www.ncbi.nlm.nih.gov/pubmed/20016847) PLoS Negl Trop Dis 3(12):e565.

3. Lariviere M, Abonnenc E, Kramer R (1961). Chronicle of cutaneous leishmaniasis in West Africa. The problem of the vector. Bull Soc Path Exot 54: 1031–1046.

4. Paz C, Doumbia S, Keita S, Sethi A (2011). [Cutaneous leishmaniasis in Mali.](http://www.ncbi.nlm.nih.gov/pubmed/21095531) Dermatol Clin 29(1):75-8.
