# Supplementary material for: Leishmaniasis Worldwide and Global Estimates of Its Incidence
Source: PLoS One. 2012 May 31;7(5):e35671. doi: 10.1371/journal.pone.0035671 (PMC3365071; doi:10.1371/journal.pone.0035671)
Supplement: Text S57 — Leishmaniasis Country Profiles, Malta. (DOCX) [file pone.0035671.s057.docx]

**MALTA**

**
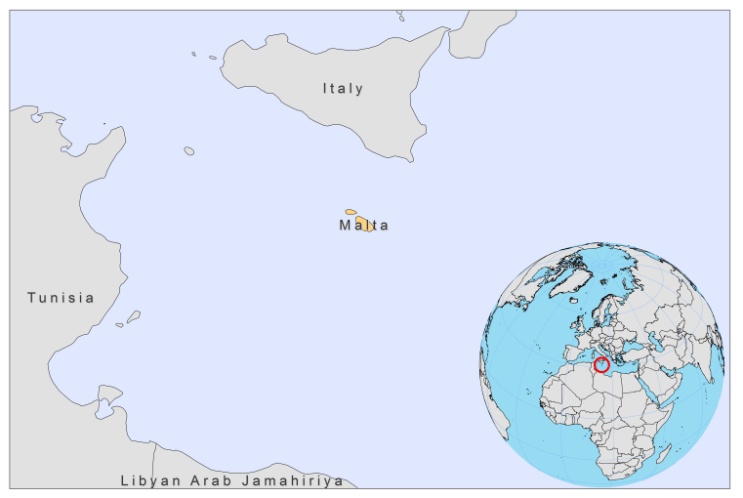
**

**BASIC COUNTRY DATA**

Total Population: 412,961

Population 0-14 years: 15%

Rural population: 5%

Population living under USD 1.25 a day: no data

Population living under the national poverty line: no data

Income status: High income economy

Ranking: Very high human development (ranking 36)

Per capita total expenditure on health at average exchange rate (US dollar): 1,446

Life expectancy at birth (years): 80

Healthy life expectancy at birth (years): 71

**BACKGROUND INFORMATION**

VL was first diagnosed in 1911 [1]. When reporting became mandatory in 1946, VL was highly prevalent with 1,264 cases [2]. The incidence has declined significantly since the 1960s. Canine VL has also decreased in the last 10 years, due to an effective control program. Before 1963, nearly all cases were aged under 10 [3]. Adult cases have become more prevalent since then: between 1980-1998, only 56% of cases were under 14 years of age [4].

CL is less prevalent in Malta, and was first reported in the early 1980s, mostly in a small eastern coastal area of Gozo [5]. The incidence of CL has recently increased.

**PARASITOLOGICAL INFORMATION**

| ***Leishmania* species** | **Clinical form** | **Vector species** | **Reservoirs** |
| --- | --- | --- | --- |
| *L. infantum* | ZVL, CL | *P. pemiciosus* | *Canis familiaris* |

**MAPS AND TRENDS**

**Visceral and cutaneous leishmaniasis**

Not available.

**Cutaneous leishmaniasis trend**

**CONTROL**

The notification of leishmaniasis has been mandatory in the country since 1946; there has been a national leishmaniasis control program since 1971. There is no leishmaniasis vector control program, but there is a reservoir control program that includes sacrifice of stray dogs and sacrifice or treatment of infected dogs with lifelong allopurinol.

**DIAGNOSIS, TREATMENT**

**Diagnosis**

CL: confirmation by microscope examination of skin lesion sample.

VL: confirmation by microscope examination of bone marrow or spleen aspirate.

**Treatment**

VL: antimonials 20 mg Sb^v^/kg/day for 30 days. Cure rate is 94% in children. Second line therapy is with liposomal amphotericin B.

CL: cryotherapy.

**ACCESS TO CARE**

All patients are thought to have access to care.

**ACCESS TO DRUGS**

Antimonials are not registered in Malta. Sodium stibogluconate (Pentostam, GSK) is used for treatment of VL.

**SOURCES OF INFORMATION**

1. Critien A (1911). Infantile leishmaniasis in Malta. Ann Trop Med Parasitol 1911;5:37.

2. [Fenech FF](http://www.ncbi.nlm.nih.gov/pubmed?term=%22Fenech%20FF%22%5BAuthor%5D) (1997). Leishmaniasis in Malta and the Mediterranean basin. [Annals of Trop Med Parasitology](javascript:__doLinkPostBack('','mdb%7E%7Eaph%7C%7Cjdb%7E%7Eaphjnh%7C%7Css%7E%7EJN%20%22Annals%20of%20Tropical%20Medicine%20%26%20Parasitology%22%7C%7Csl%7E%7Ejh','');) 91(7):747-8.

3. Grech V, et al (2000). Visceral leishmaniasis in Malta—an 18 year paediatric, population based study. Arch Dis Child 82:381–385.

4. Vella C, Grech V (2008). Declining visceral leishmaniasis in Malta. Malta Medical Journal 20 (1):25-8.

5. Desjeux P (1991). Information on the epidemiology and control of the leishmaniases by country or territory. World Health Organization. WHO/LEISH/91.30.
