# Supplementary material for: Leishmaniasis Worldwide and Global Estimates of Its Incidence
Source: PLoS One. 2012 May 31;7(5):e35671. doi: 10.1371/journal.pone.0035671 (PMC3365071; doi:10.1371/journal.pone.0035671)
Supplement: Text S58 — Leishmaniasis Country Profiles, Mauritania. (DOCX) [file pone.0035671.s058.docx]

**MAURITANIA**

**
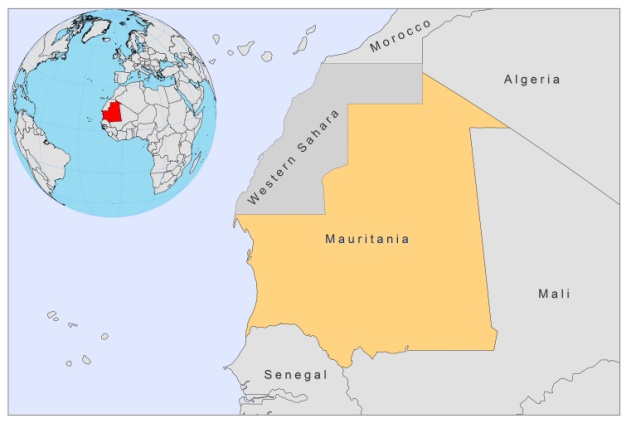
**

**BASIC COUNTRY DATA**

Total Population: 3,459,773

Population 0-14 years: 40%

Rural population: 59%

Population living under USD 1.25 a day: 21.2%

Population living under the national poverty line: no data

Income status: Lower middle income economy

Ranking: Low human development (ranking 159)

Per capita total expenditure on health at average exchange rate (US dollar): 22

Life expectancy at birth (years): 58

Healthy life expectancy at birth (years): 45

**BACKGROUND INFORMATION**

Leishmaniasis is rare in Mauritania. VL is unknown and only sporadic cases of CL have been reported. These originated from Aloun-el-Atrouss, the Nema region and near the borders with Senegal and Mali [1]. As Mauritania is part of a proposed CL endemicity belt, running across West Africa, more cases may occur than are officially reported.

**PARASITOLOGICAL INFORMATION**

| ***Leishmania* species** | **Clinical form** | **Vector species** | **Reservoirs** |
| --- | --- | --- | --- |
| *L. major* | CL | *P. duboscqi,*  *P. bergeroti* | Unknown |
| *L. infantum* | VL, CL | Unknown | Unknown |

**MAPS AND TRENDS**

No information available.

**CONTROL**

Notification of leishmaniasis is mandatory.

**DIAGNOSIS, TREATMENT, ACCESS TO CARE**

No information available.

**ACCESS TO DRUGS**

No antimonials are registered.

**SOURCES OF INFORMATION**

1. Desjeux P (1991) Information on the epidemiology and control of the leishmaniases by country or territory. World Health Organization. WHO/LEISH/91.30.
