# Supplementary material for: Leishmaniasis Worldwide and Global Estimates of Its Incidence
Source: PLoS One. 2012 May 31;7(5):e35671. doi: 10.1371/journal.pone.0035671 (PMC3365071; doi:10.1371/journal.pone.0035671)
Supplement: Text S59 — Leishmaniasis Country Profiles, Mexico. (DOCX) [file pone.0035671.s059.docx]

**MEXICO**

**
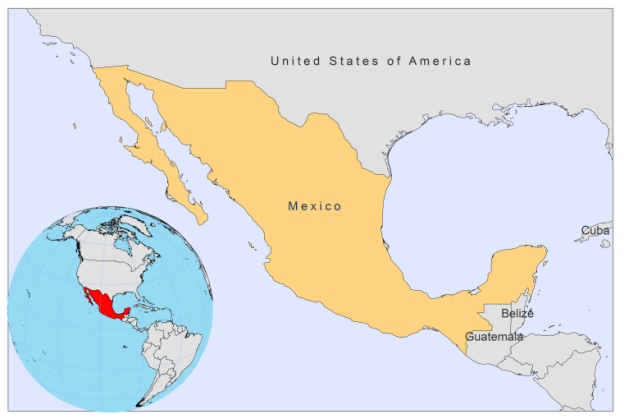
**

**BASIC COUNTRY DATA**

Total Population: 113,423,047

Population 0-14 years: 29%

Rural population: 22%

Population living under USD 1.25 a day: 3.4%

Population living under the national poverty line: 47.41%

Income status: Upper middle income economy

Ranking: High human development (ranking 57)

Per capita total expenditure on health at average exchange rate (US dollar): 525

Life expectancy at birth (years): 76

Healthy life expectancy at birth (years): 65

**BACKGROUND INFORMATION**

In Mexico, CL, MCL, DCL and VL have been reported. Localized CL, or ‘chiclero ulcer’ (‘rotting ear’), occurred mainly in southern Mexico states, in the Yucatan peninsula, whereas DCL is reported in Tabasco; MCL is very rare and has been reported from 2 states (Chiapas and Veracruz). The term *L. tropica mexicana* was first used in 1953, in Campeche, due to the similarity with the lesions by this species in the Old World [1]. Since then, *L. mexicana* was reported in many states [2] and it was believed that it was the only CL species present in the country. However, in a study done in Nayarit, a state at the central Pacific coast in 1987, the parasites found belonged to the *L.braziliensis* complex, with two different variants [3]*.* The recent introduction of this species in this area was ruled out because there is no immigration into this area. The main risk factor that was identified is working in coffee plantations [4].

The population at risk for CL is 7,613,221 in 11 states, mainly located in southeastern Mexico. Until 1986, transmission of CL was limited to the Yucatán peninsula (Belunchén in the southwest of the peninsula with 17% positive leishmanin skin tests, but in villages in the forest it was up to 90% [5]), Tabasco (with 37% of the total number of cases), Veracruz, Coahuila, Oaxaca, Guerrero, Michoacán, Morelos (very low endemicity, 2 cases reported in Cuernavaca in 1958) and Puebla. After 1987, a new focus was confirmed in Nayarit, where the species found was *L.braziliensis*, and subsequently in Jalisco and Sinaloa, which probably already existed without having been registered. Between 1990 and 2007 a total of 16,992 cases were registered, 99% of them CL. In 2006, a CL outbreak occurred in Veracruz state, affecting 76 persons, mainly in cacao plantations.

Other imported *Leishmania* species can be found in Mexico. Quintana Roo, Chiapas and Campeche states share a border with Guatemala, where *L.panamensis* and *L. guayanensis* are endemic. Many people migrate from Guatemala to Mexico, which might account for the appearance of CL imported cases due to these species in the mentioned states.

VL has been known in Mexico (Guerrero state) since 1952 [6]. Approximately 921,273 people are considered at risk for VL in 2 states. VL is mainly reported from Chiapas state, with 141 cases between 1981-2010 and an average of 8-15 cases per year, accounting for 90% of all VL cases from Mexico. VL occurs in the central-south arid area of the state, concentrated in 4-5 districts close to the river Grijalva, and is associated with malnutrition and non-immune displaced ethnic groups (*sauciles*) moving into endemic areas. Guerrero state reports cases only very sporadically; the level of underreporting is suspected to be high.

Sporadic cases of HIV-*Leishmania* co-infection have been reported.

**PARASITOLOGICAL INFORMATION**

| ***Leishmania* species** | **Clinical form** | **Vector species** | **Reservoirs** |
| --- | --- | --- | --- |
| *L. braziliensis* | ZCL, MCL | *Lu. ovallesi,*  *Lu. cruciata* | Unknown |
| *L. mexicana* | ZCL, MCL, DCL | *Lu. olmeca olmeca, Lu. cruciata,*  *Lu. shannoni* | *Heteromys spp., Nyctomys spp., Ototylomys spp., Sigmodon spp., Peromyscus spp.* |
| *L. infantum* | ZVL | *Lu. longipalpis,*  *Lu. evansi* | *Canis familiaris* |

**MAPS AND TRENDS**

**Visceral leishmaniasis**


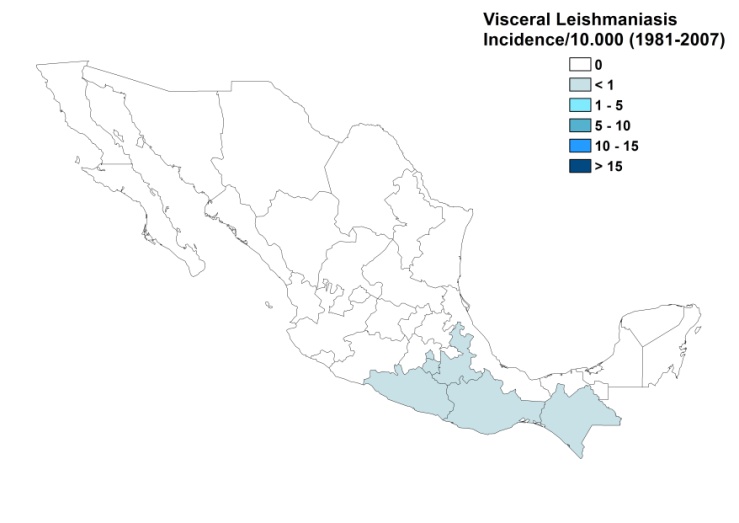

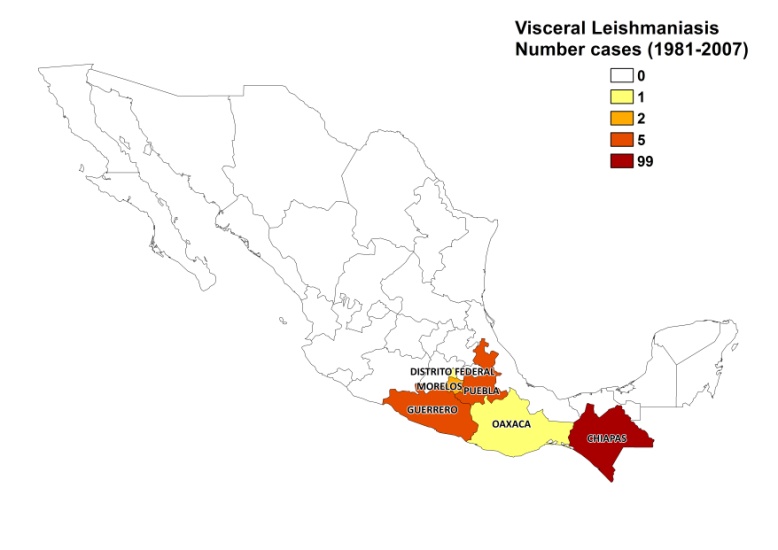


**Cutaneous leishmaniasis**

**Cutaneous leishmaniasis**

**
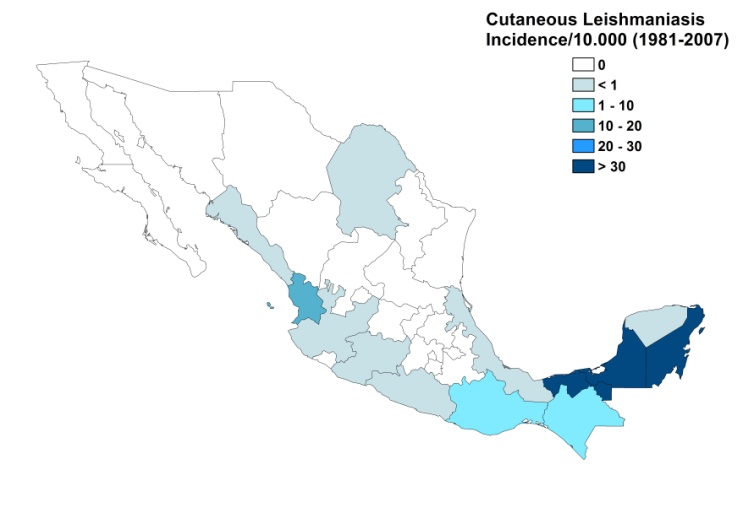

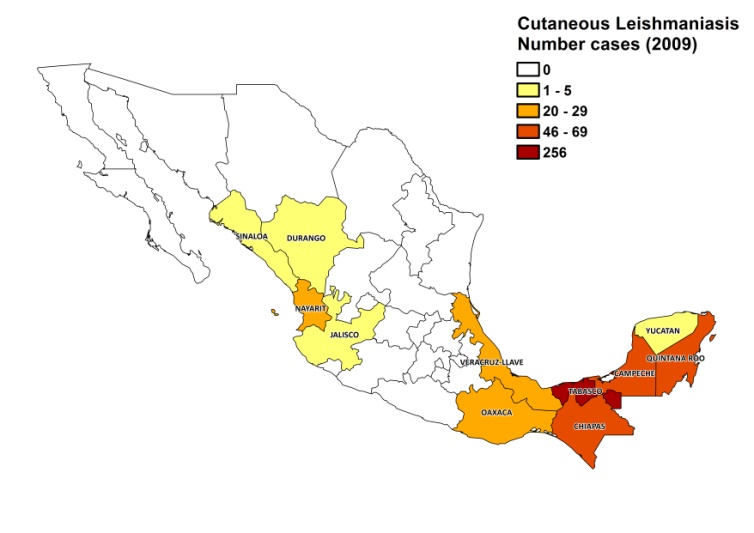
**

**Mucocutaneous and diffuse cutaneous leishmaniasis**

**
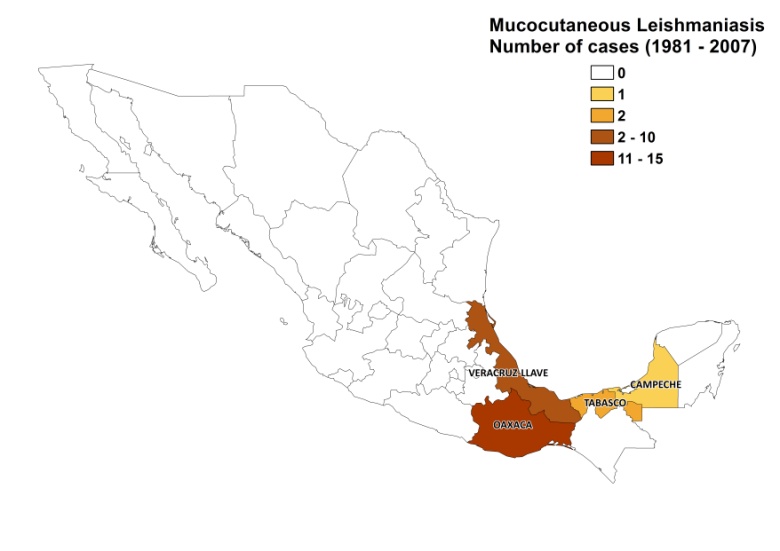

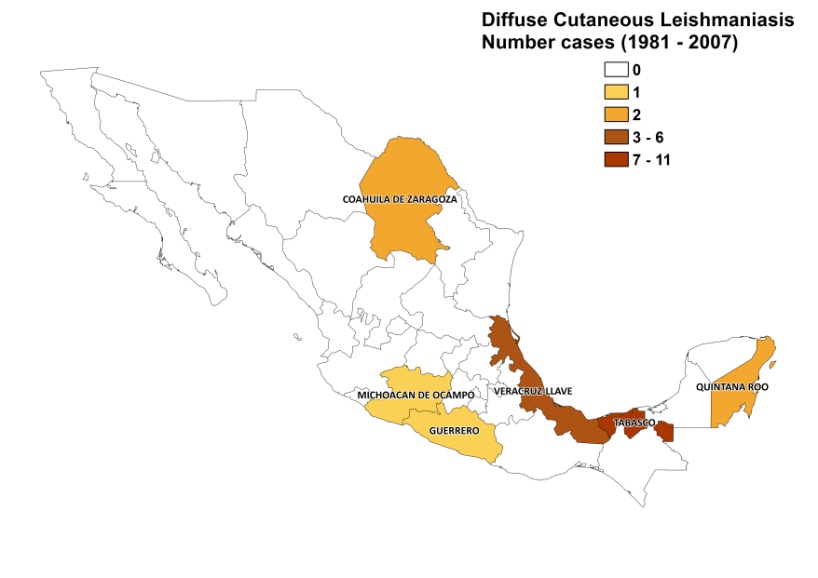
**

**Visceral leishmaniasis trend**

**Cutaneous leishmaniasis trend**

**CONTROL**

There has been a national control program for leishmaniasis since 1984 and in 1986, notification of cases became mandatory. The program practices passive epidemiological surveillance, where cases are detected during consultations in hospitals, primary health-care facilities and health houses, as well as active detection involving vector-control staff, traveling brigades and community volunteer reporters. Because of the limited resources available, the control program focuses on early case detecting and providing timely treatment. There is no vector or reservoir control program.

**DIAGNOSIS, TREATMENT**

**Diagnosis**:

CL: confirmation of clinical diagnosis at health unit level is done through microscopic examination of skin lesion samples (sent to Public Health State Laboratories). The Montenegro skin test is used if treatment needs to be started immediately.

VL: microscopic examination of bone marrow aspirate and IFAT (in the national reference laboratory).

**Treatment:**

CL: antimonials, intralesional or systemic, 20 mg Sb^v^/kg/day. Cure rate is 96%. The intralesional regimen is used for cases of CL with a single lesion or various lesions with a diameter less than 10 cm. Thermotherapy is used in the seven states that possess the equipment: Campeche, Chiapas, Nayarit, Oaxaca, Quintana Roo, Tabasco and Veracruz. Second line treatment is with amphotericin B.

VL: antimonials (20 mg Sb^v^/kg/day for 30 days). Second line treatment is with amphotericinB.

**ACCESS TO CARE**

Treatment is available for free. VL can only be diagnosed and treated by a specialist in large hospitals. CL is diagnosed and treated at health unit level. Drug purchase for leishmaniasis (Glucantime, Sanofi) is done mostly in small and insufficient amounts due to a lack of resources. Most reported cases are treated with thermotherapy. However, a major restriction is the high maintenance cost of thermotherapy equipment, and the fact that some items of equipment are in disrepair.

There is a lack of access to treatment. Most CL patients do not seek treatment in time due to a lack of awareness of the serious nature of the disease, but also because most patients come from rural areas, and because males cannot take time off from work to seek treatment. VL often occurs in children and many cannot afford taking their children with VL to a specialized hospital for treatment. There are no NGOs treating leishmaniasis in Mexico.

**ACCESS TO DRUGS**

The General Council of Health has not accepted the inclusion of meglumine antimoniate in the Essential Drug List, due to the fact that the need for this drug is very small at a national level. At present, only the state health services can buy the drug; the federal offices cannot buy it directly because the drug is not in the official list. Miltefosine (Paladin, Canada) and Glucantime (Sanofi) are registered. Drugs for leishmaniasis are not available in private pharmacies.

**SOURCES OF INFORMATION**

- Dr Juan Ignacio Arredondo Jiménez, Vector Borne Diseases Programme, Secretaria de Salud de México, Centro Nacional de Vigilancia Epidemiológica y Control de Enfermedades.
- Dr Nancy Treviño Garza, Leishmaniasis National Programme, Secretaria de Salud de México, Centro Nacional de Vigilancia Epidemiológica y Control de Enfermedades. *Leishmaniasis en la Región de las Américas. Reunión de coordinadores de Programa Nacional de Leishmaniasis. OPS/OMS. Medellín, Colombia. 4-6 junio 2008.*
- Dr Graciela P. Peña Flores, Oncocercosis and other Vector Borne Diseases, Secretaria de Salud de México, Centro Nacional de Vigilancia Epidemiológica y Control de Enfermedades.

1. Biagi FF (1953). Algunas consideraciones sobre la leishmaniasis y sus agentes etiologicos: *Leishmania tropica mexicana*. Nueva subespecie. Medicina Mex 31:401-6.

2. Hernandez-Montes O, Monroy-Ostria A, McCann S, Barker DC (1998). [Identification of Mexican Leishmania species by analysis of PCR amplified DNA.](http://www.ncbi.nlm.nih.gov/pubmed/9821463) Acta Trop 15;71(2):139-53.

3. Velasco-Castrejon O, Savarino S, Neva F, Guzman-Bracho C (1989). Los agentes etiológicos de las leishmaniasis cutáneas en México. Presencia de *L.* *braziliensis* en Mexico. Rev Lat Amer Microbiol 31: 231-234.

4. Sanchez-Tejeda G, Rodríguez N, Parra CI, Hernandez-Montes O, Barker DC et al (2001). [Cutaneous leishmaniasis caused by members of Leishmania braziliensis complex in Nayarit, State of Mexico.](http://www.ncbi.nlm.nih.gov/pubmed/11285471) Mem Inst Oswaldo Cruz 96(1):15-9.

5. Andrade-Narváez FJ, Simmonds-Díaz E, Rico-Aguilar S, Andrade-Narvaez M, Palomo-Cetina A et al (1990). [Incidence of localized cutaneous leishmaniasis (chiclero's ulcer) in Mexico.](http://www.ncbi.nlm.nih.gov/pubmed/2389310) Trans R Soc Trop Med Hyg 84(2):219-20.

6. Baez-Villaseñor J, Ruiola J, Rojas E (1952). Presentacion de un caso de Kala-azar. Rev Invest Clin Mex 4:57-58.
