# Supplementary material for: Leishmaniasis Worldwide and Global Estimates of Its Incidence
Source: PLoS One. 2012 May 31;7(5):e35671. doi: 10.1371/journal.pone.0035671 (PMC3365071; doi:10.1371/journal.pone.0035671)
Supplement: Text S60 — Leishmaniasis Country Profiles, Monaco. (DOCX) [file pone.0035671.s060.docx]

**MONACO**

**
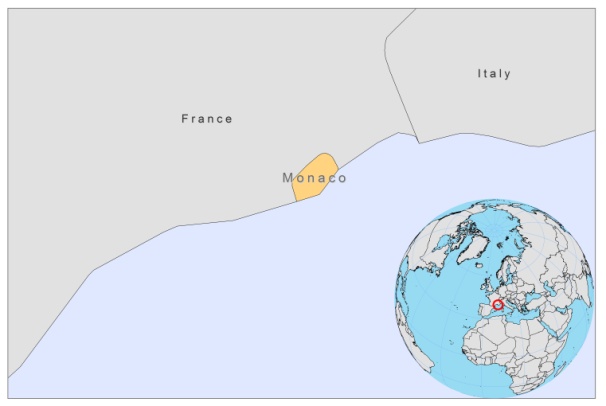
**

**BASIC COUNTRY DATA**

Total Population: 35,407

Population 0-14 years: 18%

Rural population: 0%

Population living under USD 1.25 a day: no data

Population living under the national poverty line: no data

Income status: High Income economy

Ranking: Very high human development

Per capita total expenditure on health at average exchange rate (US dollar): 7,137

Life expectancy at birth (years): 82

Healthy life expectancy at birth (years): 73

**BACKGROUND**

The Alpes Maritimes are a known VL endemic region and Monaco is no exception. VL has a high prevalence in dogs in Monaco [1]. It is relatively rare in humans, but between 1991-1992, 8 cases were diagnosed in Monaco and Beausoleil [1]. The sandfly vector was identified in 1993, and was found to be present at Monaco beach, the harbour, as well as in the surrounding hills. There was a high rate of infected *Phlebotomus* spp. (3%), which marked Monaco as an active focus of VL. In a screening of blood samples taken from 565 donors at the Monaco bloodbank, 13.4% was infected subclinically with *L.infantum* [2].

**PARASITOLOGICAL INFORMATION**

| ***Leishmania***  **species** | **Clinical form** | **Vector species** | **Reservoirs** |
| --- | --- | --- | --- |
| *L.infantum* | VL | *P. perniciosus* | *Canis familiaris* |

**No further information is available**

**SOURCES OF INFORMATION**

1. Izril MA, Marty P, Fauran P, Le Fichoux Y, Rousset J-J (1996). Presumed vectors of leishmaniasis in the Principality of Monaco, Trans R Soc Trop Med Hyg 90:114.

2. le Fichoux Y, Quaranta JF, Aufeuvre JP, Lelievre A, Marty P et al (1999). Occurrence of Leishmania infantum Parasitemia in Asymptomatic Blood Donors Living in an Area of Endemicity in Southern France. J Clin Microbiol 37(6): 1953–1957.
