# Supplementary material for: Leishmaniasis Worldwide and Global Estimates of Its Incidence
Source: PLoS One. 2012 May 31;7(5):e35671. doi: 10.1371/journal.pone.0035671 (PMC3365071; doi:10.1371/journal.pone.0035671)
Supplement: Text S61 — Leishmaniasis Country Profiles, Mongolia. (DOCX) [file pone.0035671.s061.docx]

**MONGOLIA**

**
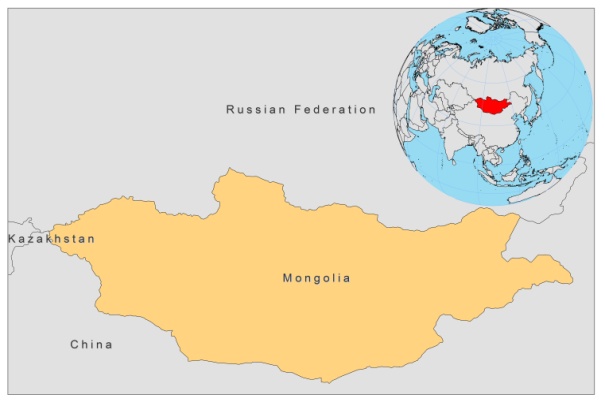
**

**BASIC COUNTRY DATA**

Total Population: 2,756,001

Population 0-14 years: 28%

Rural population: 43%

Population living under USD 1.25 a day: 22.4%

Population living under the national poverty line: 35.2%

Income status: Lower middle income economy

Ranking: Medium human development (ranking 110)

Per capita total expenditure on health at average exchange rate (US dollar): 75

Life expectancy at birth (years): 68

Healthy life expectancy at birth (years): 56

**BACKGROUND**

*Leishmania* infection was first reported in Mongolia in 1976 in great gerbils; up to 100% of them were infected in some areas in the south of the country [1]. In an 8-year-long and extensive study in Mongolia, no other reservoir could be identified, indicating that *L. major* in Mongolia is limited to great gerbils only. The parasites identified were *L. major* and *L. gerbilii*, the latter a non virulent species. No human cases have been notified. In 1990 a new species, *L. turanica*, was identified, also only occurring in great gerbils [2].

**PARASITOLOGICAL INFORMATION**

| ***Leishmania* species** | **Clinical form** | **Vector species** | **Reservoirs** |
| --- | --- | --- | --- |
| *L. major* | ? | Unknown | Unknown |

**No further information is available**

**SOURCES OF INFORMATION**

1. Neronov VM, Strelkova MV, Shurkhal AA, Luschekina AA, Artemyev MM (1987). Natural focality of zoonotic cutaneous leishmaniasis in the Mongolia’s Peoples Republic; results and objectives of integrated research. Folia Parasitologica 34: 1-9.

2. Strelkovaa MV, Shurkhal AV, Kellina OI, Eliseev LN, Evans DA et al (1990). A new species of Leishmania isolated from the great gerbil Rhombomys opimus. Parasitology 101: 327-335.
