# Supplementary material for: Leishmaniasis Worldwide and Global Estimates of Its Incidence
Source: PLoS One. 2012 May 31;7(5):e35671. doi: 10.1371/journal.pone.0035671 (PMC3365071; doi:10.1371/journal.pone.0035671)
Supplement: Text S62 — Leishmaniasis Country Profiles, Montenegro. (DOCX) [file pone.0035671.s062.docx]

**MONTENEGRO**

**
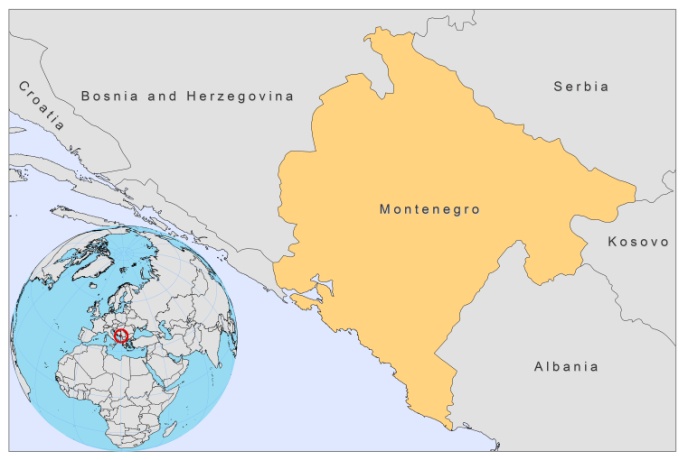
**

**BASIC COUNTRY DATA**

Total Population: 631,490

Population 0-14 years: 19%

Rural population: 41%

Population living under USD 1.25 a day: no data

Population living under the national poverty line: 4.9%

Income status: Upper middle income economy

Ranking: High human development (ranking 54)

Per capita total expenditure on health at average exchange rate (US dollar): 617

Life expectancy at birth (years): 74

**BACKGROUND INFORMATION**

Montenegro is endemic for leishmaniasis. From 1992 to 2009, 55 cases of VL were reported, and one case of CL. Underreporting is common; a survey between 1992-1995 showed that out of 19 children treated, only 8 were officially reported. 40% of cases occur in children under 5, and 67% in children between 0 and 15 years old. 62% of cases are male.

From 2006-2009, serological surveys of clinically suspect dogs coming from VL endemic areas found a 58% average rate of infection.

There are no reported cases of HIV/*Leishmania* co-infection.

**PARASITOLOGICAL INFORMATION**

| ***Leishmania* species** | **Clinical form** | **Vector species** | **Reservoirs** |
| --- | --- | --- | --- |
| *L. infantum* | ZVL | *P. neglectus* | *Canis familiaris* |

**MAPS AND TRENDS**

**Visceral leishmaniasis**


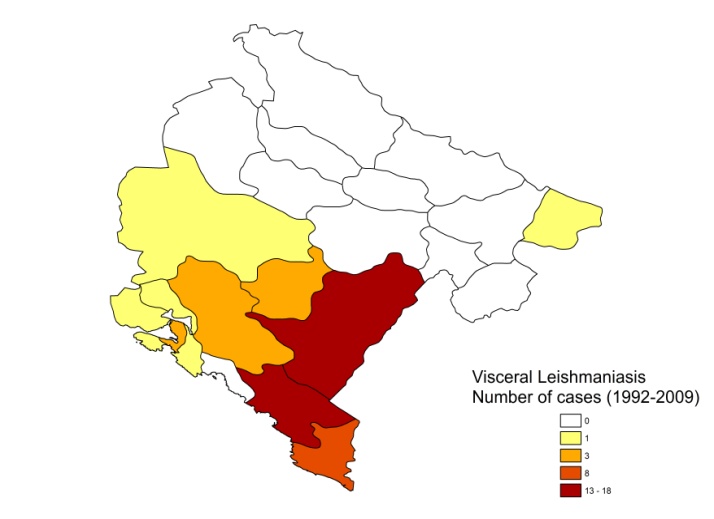

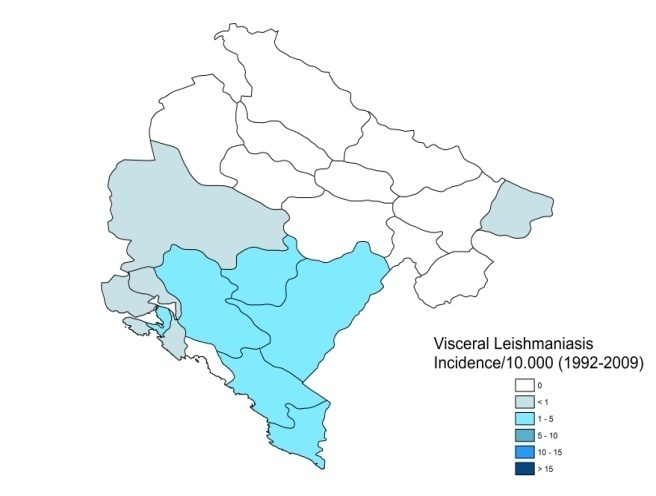


**Cutaneous leishmaniasis**

No information available.

**Visceral leishmaniasis trend**

**CONTROL**

The notification of leishmaniasis is mandatory in the country. There is no national leishmaniasis control program, no leishmaniasis vector control program and no leishmaniasis reservoir control program.

**DIAGNOSIS, TREATMENT**

**Diagnosis:**

VL: microscopic examination of bone marrow aspirate, IFAT and ELISA.

**Treatment:**

VL: antimonials, 20 mg Sb^v^/kg/day. Mortality rate 0%.

**ACCESS TO CARE**

No information.

**ACCESS TO DRUGS**

Antimonials are not registered in Montenegro and no drugs for leishmaniasis are included in the essential drugs list. Patients are known to privately purchase antimonials (Glucantime, Sanofi) in Italy. Antimonials are not included in the list of drugs that are reimbursed by the National Health Insurance.

**SOURCES OF INFORMATION**

- Dr Veseselinka Beatović, Institute of Public Health, Ministry of Health. *WHO exploratory meeting on Leishmaniasis in the Balkan Countries. Dubrovnik, Croatia, 10-12 February 2010.*
