# Supplementary material for: Leishmaniasis Worldwide and Global Estimates of Its Incidence
Source: PLoS One. 2012 May 31;7(5):e35671. doi: 10.1371/journal.pone.0035671 (PMC3365071; doi:10.1371/journal.pone.0035671)
Supplement: Text S63 — Leishmaniasis Country Profiles, Morocco. (DOCX) [file pone.0035671.s063.docx]

**MOROCCO**

**
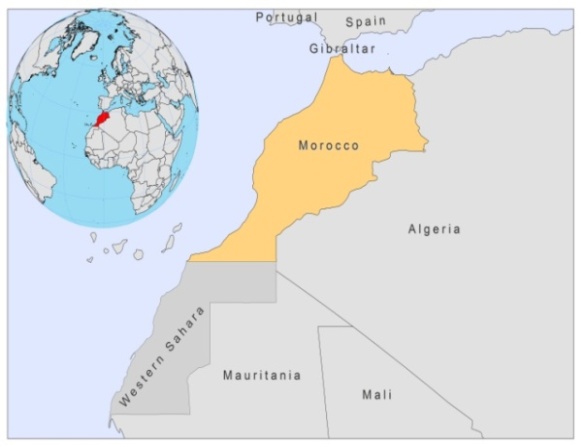
**

**BASIC COUNTRY DATA**

Total Population: 31,951,412

Population 0-14 years: 30%

Rural population: 43%

Population living under USD 1.25 a day: 2.5%

Population living under the national poverty line: 9%

Income status: Lower middle income economy

Ranking: Medium human development (ranking 130)

Per capita total expenditure on health at average exchange rate (US dollar): 156

Life expectancy at birth (years): 72

Healthy life expectancy at birth (years): 60

**BACKGROUND INFORMATION**

The first case of VL was described in 1922 [1]. VL is endemic in the Rift and pre-Rift mountains, with over 150 cases per year in 2006-2008.

CL caused by *L.major* has been reported since 1914 [2,3]. Previously sporadic, it has become epidemic since 1976. It occurs in unpredictable outbreaks, in the south and south-east of the Atlas mountains, and seems to have moved in waves from west to east over several years. In the 1980s, 20,000 cases were notified in this area. In 2008, there were 3,414 reported cases, more than double the number of cases the previous year.

CL due to *L.tropica* was first identified in 1987, and since 1997, has been considered a major public health threat [1,4]. It has a large distribution throughout Morocco, in a band stretching from the Atlantic Ocean along the length of the Atlas Mountains almost to the Mediterranean Sea, where it is considered epidemic in suburban areas. In 2008, 1,697 cases were reported.

Occasional cases of CL caused by *L. Infantum* occur in the north of the country [1].

CL and VL are both suspected to be underreported.

No cases of HIV/*Leishmania* co-infection have been reported.

**PARASITOLOGICAL INFORMATION**

| ***Leishmania* species** | **Clinical form** | **Vector species** | **Reservoirs** |
| --- | --- | --- | --- |
| *L. major* | ZCL | *P. papatasi* | *Meriones shawi, Psammomys obesus* |
| *L. tropica* | CL | *P. sergenti,*  *P. chabaudi* | Human,  *Canis familiaris* |
| *L. infantum* | ZVL, CL | *P. perniciosus,*  *P. ariasi,*  *P. longicuspis* | *Canis familiaris* |

**MAPS AND TRENDS**

**Visceral leishmaniasis**


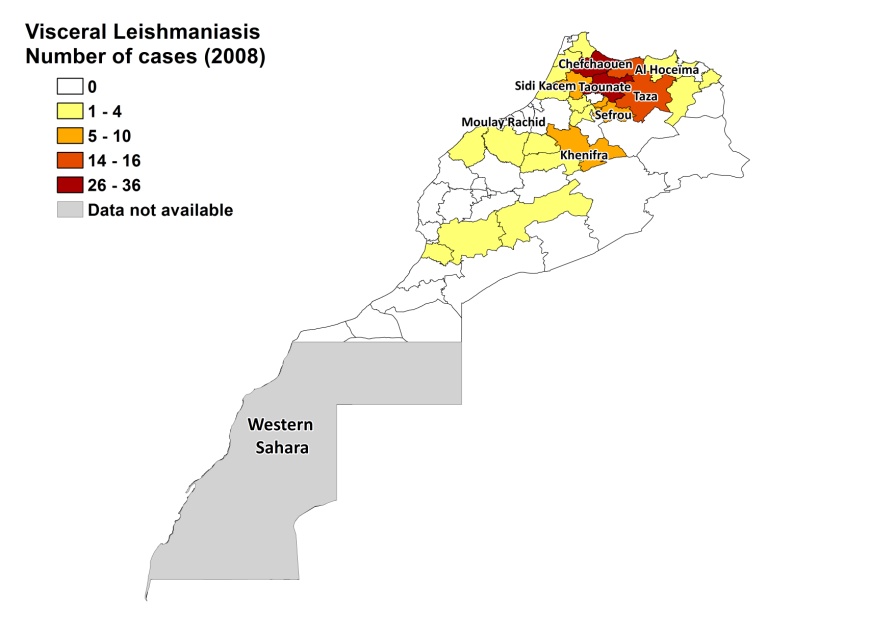


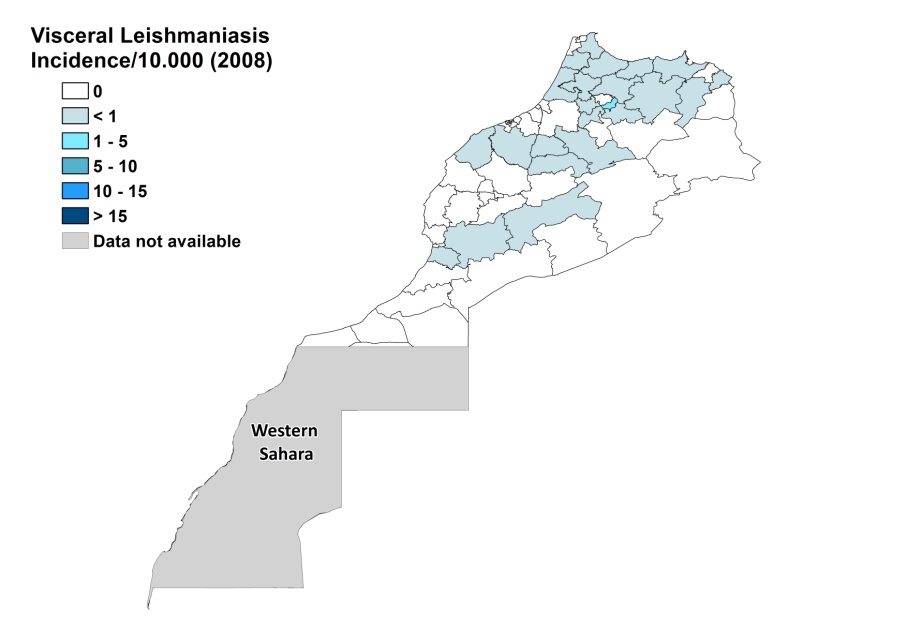


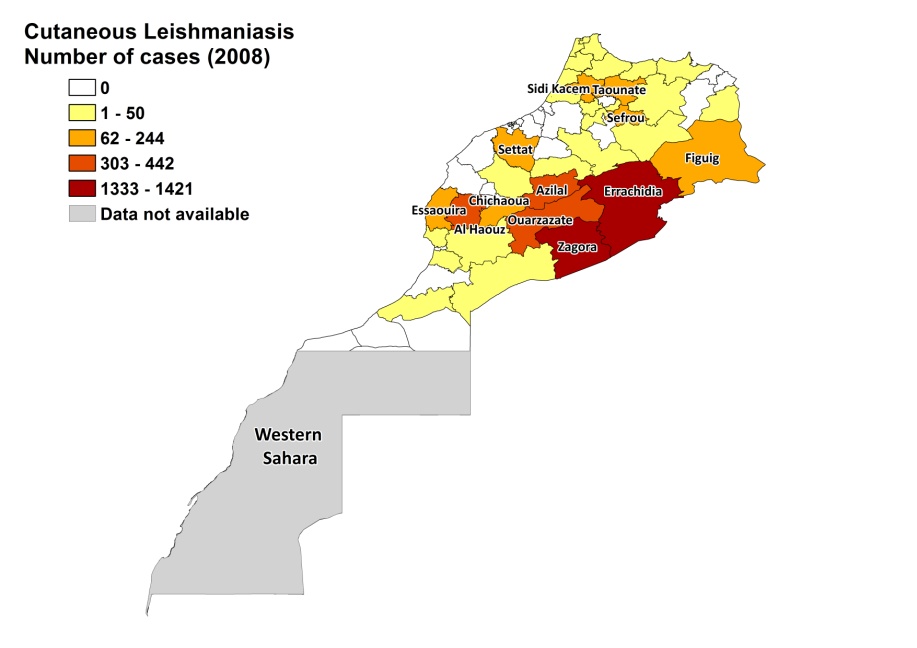

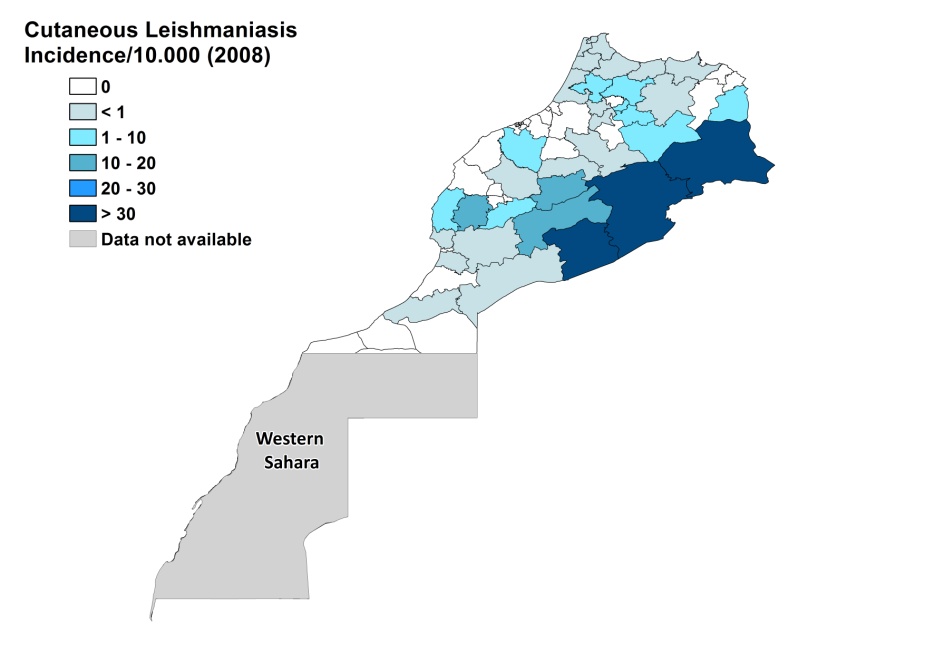
**Cutaneous Leishmaniais**

**Visceral leishmaniasis trend**

**Cutaneous leishmaniasis trend**

**CONTROL**

Notification of leishmaniasis is mandatory and a national leishmaniasis control program for VL and CL was established in 1997. Active human case detection is regularly performed and there is a vector control program, including bednet distribution and insecticide spraying. There is also a reservoir control program in place, with regular rodent control and collective hygiene promotion, including garbage collection.

**DIAGNOSIS, TREATMENT**

**Diagnosis:**

CL: on clinical grounds, confirmation with microscopic examination of skin lesion sample, PCR.

VL: confirmation by microscopic examination of bone marrow aspirate, PCR, IFAT.

**Treatment:**

CL: antimonials, intralesional in case of CL caused by *L.tropica,* and intralesional or systemic (10-15 mg Sb^v^/kg/day for 15 days) for CL caused by *L.major*. Cure rate is 99% and in 1% lesions relapse.

VL: antimonials, 20 mg Sb^v^/kg/day for 3 weeks. Cure rate is 99%, and the fatality rate is 1%.

**ACCESS TO CARE**

Medical care is provided for free in Morocco, which includes care for leishmaniasis, although informal payments are charged by health workers. 99% of cases are treated in the public sector. VL can be diagnosed and treated in district hospitals, and CL can be diagnosed and treated at primary health care level. All patients are thought to have access to care. The Ministry of Health provided sufficient antimonials (Glucantime, Sanofi) to treat all reported cases in 2007 and 2008.

**ACCESS TO DRUGS**

Meglumine antimoniate is included in the National Essential Drug List for leishmaniasis. Drugs for leishmaniasis are not available at private pharmacies in Morocco. Glucantime (Sanofi) is the only drug registered in Morocco for leishmaniasis.

**SOURCES OF INFORMATION**

- Dr Laamrani El Idrissi Abderrahmane, Ministry of Health. *WHO Consultative meeting on Cutaneous Leishmaniasis in EMRO countries, Geneva, 30 April to 2 May 2007.*

1. [Rhajaoui M](http://www.ncbi.nlm.nih.gov/pubmed?term=%22Rhajaoui%20M%22%5BAuthor%5D) (2011). Human leishmaniases in Morocco: A nosogeographical diversity. [Pathol Biol (Paris)](javascript:AL_get(this,%20'jour',%20'Pathol%20%0d%0aBiol%20(Paris).');) 59(4):226-9.

2. Rioux JA, Lanotte G, Petter F, Dereure J, Akalay O et al (1986). Les leishmanioses cutanées du bassin Méditerranéen occidental. De l'identification enzymatique à l'analyse éco-épidémiologique. L'exemple de trois foyers, tunisien, marocain et français. In: Rioux JA, editor. Leishmania taxonomie et phylogénèse. Applications éco-épidémiologiques. Colloque International Centre National de la Recherche Scientifique, Institut National de la Santé et de la Recherche Médicale (CNRS INSERM) 1984. L’Institut Méditerranéen d’Etudes Epidémiologiques et Ecologiques (IMEEE), Montpellier p. 365–95.

3. Rhajaoui M, Nasereddin A, Fellah H, Azmi K, Amarir F et al (2007). New Clinicoepidemiologic Profile of Cutaneous Leishmaniasis, Morocco. Emerg Infect Dis 13: 1358-60.

4. Guessous-Idrissi N, Chiheb S, Hamdani A, Riyad M, Bichichi M et al (1997). Cutaneous leishmaniasis: an emerging epidemic focus of Leishmania tropica in North Morocco*.* Trans R Soc Trop Med Hyg 91:600–3.
