# Supplementary material for: Leishmaniasis Worldwide and Global Estimates of Its Incidence
Source: PLoS One. 2012 May 31;7(5):e35671. doi: 10.1371/journal.pone.0035671 (PMC3365071; doi:10.1371/journal.pone.0035671)
Supplement: Text S64 — Leishmaniasis Country Profiles, Namibia. (DOCX) [file pone.0035671.s064.docx]

**NAMIBIA**


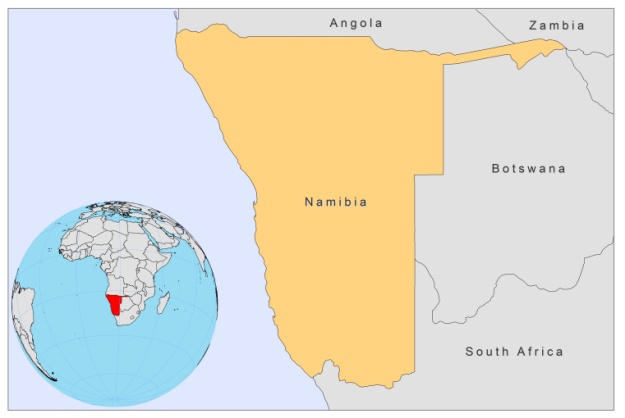


**BASIC COUNTRY DATA**

Total Population: 2,293,289

Population 0-14 years: 36%

Rural population: 62%

Population living under USD 1.25 a day: no data

Population living under the national poverty line: no data

Income status: Upper middle income economy

Ranking: Medium human development (ranking 120)

Per capita total expenditure on health at average exchange rate (US dollar): 258

Life expectancy at birth (years): 62

Healthy life expectancy at birth (years): 43

**BACKGROUND INFORMATION**

VL is unknown in Namibia. The first case of CL was described in 1970. Since then, sporadic cases have been reported (34 between 1970 and 1989) from a wide area [1]. The ecology is partly similar to *L. aethiopica,* but the causative agent is different. It was similar, but not identical, to *L. tropica*. Its designation is still under discussion [2].

**PARASITOLOGICAL INFORMATION**

| ***Leishmania* species** | **Clinical form** | **Vector species** | **Reservoirs** |
| --- | --- | --- | --- |
| *L. tropica* | CL | *P. rossi, P. grovei* | *Procavia capensis* |

**MAPS AND TRENDS, CONTROL, DIAGNOSIS, TREATMENT, ACCESS TO CARE, ACCESS TO DRUGS**

No information available.

No antimonials are registered.

**SOURCES OF INFORMATION**

1. Grové SS (1989). Leishmaniasis in South West Africa Namibia to date. S Afr Med J 75: 290–292.

2. Jacobson RL (2003). *Leishmania tropica* (Kinetoplastida: Trypanosomatidae) – a perplexing parasite. Folia Parasitologica 50: 241–250.
