# Supplementary material for: Leishmaniasis Worldwide and Global Estimates of Its Incidence
Source: PLoS One. 2012 May 31;7(5):e35671. doi: 10.1371/journal.pone.0035671 (PMC3365071; doi:10.1371/journal.pone.0035671)
Supplement: Text S65 — Leishmaniasis Country Profiles, Nepal. (DOCX) [file pone.0035671.s065.docx]

**NEPAL**


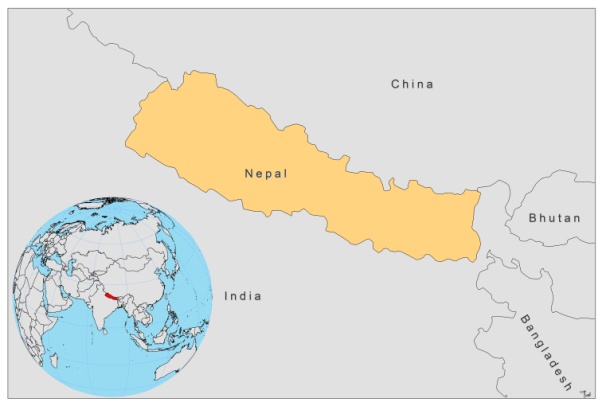


**BASIC COUNTRY DATA**

Total Population: 29,959,364

Population 0-14 years: 36%

Rural population: 82%

Population living under USD 1.25 a day: 78.1%

Population living under the national poverty line: no data

Income status: Low income economy

Ranking: Low human development (ranking 157)

Per capita total expenditure on health at average exchange rate (US dollar): 25

Life expectancy at birth (years): 68

Healthy life expectancy at birth (years): 52

**BACKGROUND INFORMATION**

VL was highly prevalent before 1958, but in the 1960s, during the Malaria Eradication Program, DDT spraying was thought to have eliminated the disease [1].

VL was however officially reported again in 1980 and was believed to have been reintroduced in Nepal from the Indian state of Bihar, where a large outbreak took place in 1977 [2]. From 1980 to 1989, the incidence rate per 100,000 person-years remained below 10. Since then, the incidence has grown steadily and in the last few years it increased from 43 to 55 per 100,000 person-years [3]. 25,890 cases and 599 deaths were reported from 16 affected districts between 1980 and July 2006, all in regions bordering the endemic districts of Bihar, India. The majority of patients were older than 15 [4]. Since 2006, when the Kala-azar Elimination Program started (targeting less than 1/10,000 population in 2015), a sharp decline in the number of cases has been observed, but between 2007 and 2010, VL was notified from an increasing number of districts (from 14 in 2007 to 26 in 2010). In 9 districts, the elimination target has been reached. However, underreporting is substantial, and the real number of cases may be 1.5 times higher than the reported numbers (BPKHIS, 2010). VL affects the poorest of the poor, and risk factors for infection are poverty related factors such as poor nutrition and housing conditions, with cracked mud walls and damp floors, and sleeping outside during the summer months [5]. Humans seem to be the only reservoir, with infected patients and PKDL cases constituting the source of infection. PKDL incidence was estimated based on screening of past VL cases treated by BPKHIS at 2.3% of VL cases. Of all cases reported to BPKHIS from 1999-2010, the male:female ration was 1.5:1 and 74% was older than 15.

HIV-*Leishmania* co-infection is estimated to occur in 1-2% of patients.

CL is very rare. In 2008, an autochtonous case of CL caused by *L. major* was reported [6].

**PARASITOLOGICAL INFORMATION**

| ***Leishmania* species** | **Clinical form** | **Vector species** | **Reservoirs** |
| --- | --- | --- | --- |
| *L. donovani* | AVL, PKDL | *P. argentipes* | Human |

**MAPS AND TRENDS**


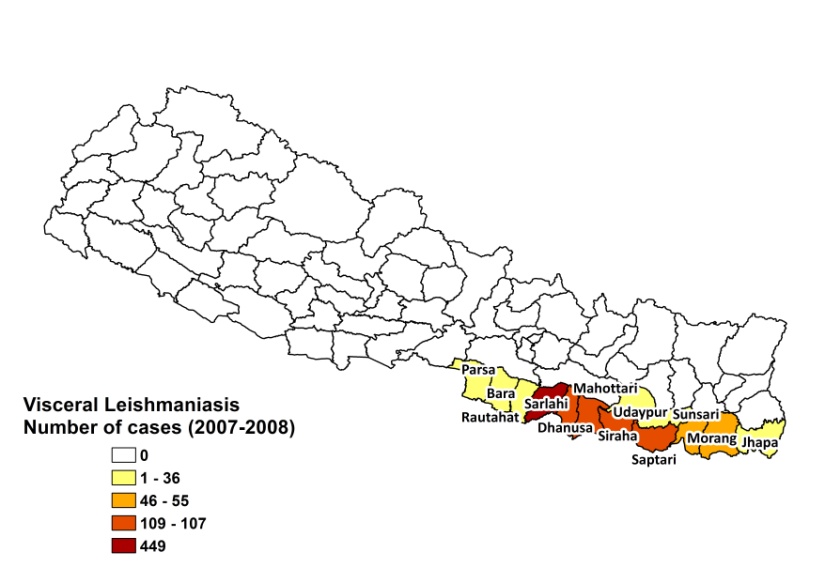
**Visceral leishmaniasis**


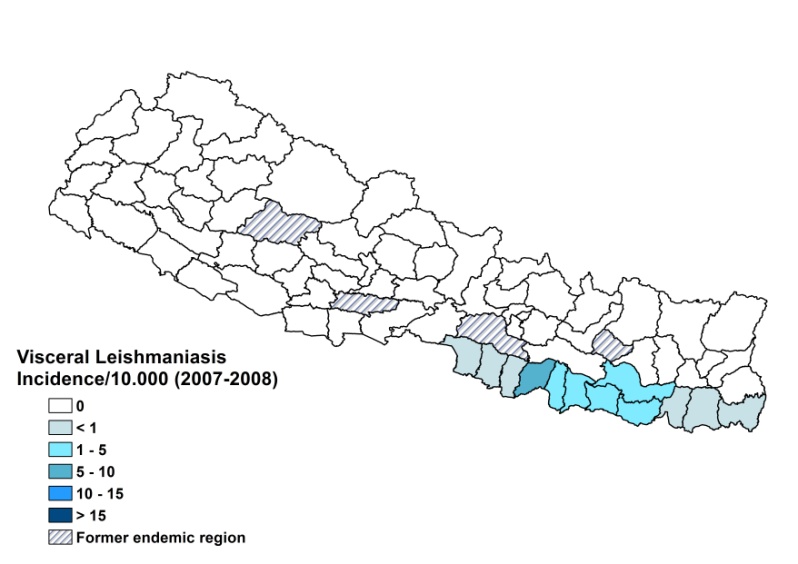


**Visceral leishmaniasis trend**

**CONTROL**

The notification of VL is not mandatory. Case reporting is based on passive surveillance. A leishmaniasis control program has been in place since 1993. An agreement for a Kala Azar Elimination Program was signed in 2005 in order to reduce the incidence of VL to 1/10,000 at district level by 2015.

**DIAGNOSIS, TREATMENT**

**Diagnosis**

VL: rK39 antigen-based immunochromatographic test (ICT), microscopic examination of spleen aspirate.

**Treatment**

VL: miltefosine, 2.5 mg/kg/day for 28 days. There are 11% relapses and the fatality rate is 1.5%. Second line: conventional amphotericin B, 1 mg/kg/day, 14 doses. Resistance to antimonials is suspected as it is highly prevalent in the neighboring Indian state of Bihar. Sodium stibogluconate is being phased out, but still offered as treatment in 4 districts (20 mg Sb^v^/kg/day for 30 days).

**ACCESS TO CARE**

Treatment for leishmaniasis patients has been provided free of charge since 2 years; it also includes a reimbursement of transport to the health facility of 1,000 Nepalese Rupees. However, access to treatment remains incomplete. Not all health centers have trained human resources and drugs for treating leishmaniasis. A significant amount of patients live in very remote areas, with no health facilities and no transport, or are very poor and lack the funds to travel. As the transport fee is only given after treatment is finished, some patients can not reach the health centers because of a lack of cash. Outside the official endemic areas, VL diagnosis and treatment are not provided. Patients in these areas have no access to treatment; however, sporadic cases of leishmaniasis occur. Poor patients suffer major economic loss when they spend time away from home. For these reasons, most first seek care at a local village health worker, unqualified to treat leishmaniasis. When seeking care outside the community, 45% of patients use of the public sector, 11% use poorly trained private practitioners and 23% resort to indigenous healers [7].

The Government purchased miltefosine, amphotericin and SSG treatments for around 1,000 patients in 2008. In 2007 and 2008, 1,433 resp. 1,371 patients were reported and treated in the public sector. The real number of patients in Nepal is thought to be higher than the number reported and treated in the public sector.

**ACCESS TO DRUGS**

Sodium stibogluconate, miltefosine and amphotericin B are included in the National Essential Drug List for VL. Miltefosine (Paladin, Canada) is registered in Nepal. These drugs are not available in private pharmacies or drug markets.

**SOURCES OF INFORMATION**

- Dr Suman Rijal, Koirala Institute of Health Sciences (BPKHIS). *WHO informal consultation on epidemiological information on disease burden due to kala-azar in Bangladesh, India and Nepal. Paro, Bhutan, 8-10 March 2011.*

1. Dahal S (2008). Climatic determinants of malaria and kala-azar in Nepal. Regional Health Forum 12 (1).

2. Bista MB (1998). National overview of kala-azar in Nepal. In: Kala-azar in Nepal: Principles, Practice and Public Health Perspectives (eds S Bastola, P Karki, S Rijal & A Gautam). EDCD/BPKIHS/WHO, Kathmandu, Nepal. 1–5.

3. Schenkel K, Rijal S, Koirala S, Koirala S, Vanlerberghe V, et al (2006). Visceral leishmaniasis in southeastern Nepal: A cross-sectional survey on Leishmania donovani infection and its risk factors. Trop Med Int Health 11(12): 1792–1799.

4. Joshi DD, Sharma M, Bhandari S (2006). Visceral leishmaniasis in Nepal during 1980-2006. J Commun Dis 38(2):139-48.

5. Bern C, Courtenay O, Alvar J (2010). Of cattle, sand flies and men: a systematic review of risk factor analyses for South Asian visceral leishmaniasis and implications for elimination.PLoS Negl Trop Dis 9;4(2):e599.

6. Kumar R, Ansari NA, Avninder S, Ramesh V and Salotra P (2008). Cutaneous leishmaniasis in Nepal: *Leishmania major* as a cause. Trans R Soc Trop Med Hyg 102: 202—203.

7. [Mondal D](http://www.ncbi.nlm.nih.gov/pubmed?term=%22Mondal%20D%22%5BAuthor%5D), [Singh SP](http://www.ncbi.nlm.nih.gov/pubmed?term=%22Singh%20SP%22%5BAuthor%5D), [Kumar N](http://www.ncbi.nlm.nih.gov/pubmed?term=%22Kumar%20N%22%5BAuthor%5D), [Joshi A](http://www.ncbi.nlm.nih.gov/pubmed?term=%22Joshi%20A%22%5BAuthor%5D), [Sundar S](http://www.ncbi.nlm.nih.gov/pubmed?term=%22Sundar%20S%22%5BAuthor%5D) et al (2009). Visceral leishmaniasis elimination programme in India, Bangladesh, and Nepal: reshaping the case finding/case management strategy. [PLoS Negl Trop Dis.](javascript:AL_get(this,%20'jour',%20'PLoS%20Negl%20Trop%20Dis.');)3(1):e355.
