# Supplementary material for: Leishmaniasis Worldwide and Global Estimates of Its Incidence
Source: PLoS One. 2012 May 31;7(5):e35671. doi: 10.1371/journal.pone.0035671 (PMC3365071; doi:10.1371/journal.pone.0035671)
Supplement: Text S66 — Leishmaniasis Country Profiles, Nicaragua. (DOCX) [file pone.0035671.s066.docx]

**NICARAGUA**


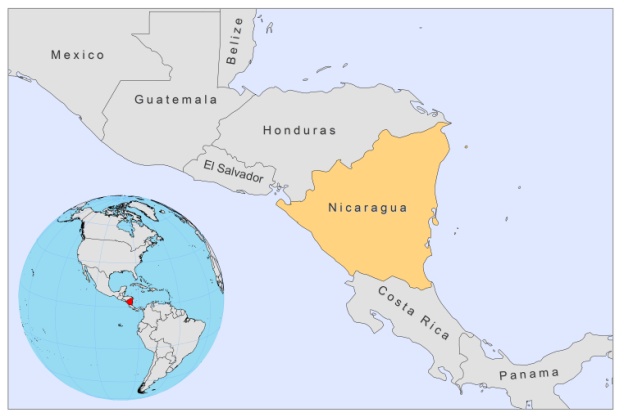


**BASIC COUNTRY DATA**

Total Population: 5,788,163

Population 0-14 years: 34%

Rural population: 43%

Population living under USD 1.25 a day: 15.8%

Population living under the national poverty line: no data

Income status: Lower middle income economy

Ranking: Medium human development (ranking 129)

Per capita total expenditure on health at average exchange rate (US dollar): 105

Life expectancy at birth (years): 73

Healthy life expectancy at birth (years): 61

**BACKGROUND INFORMATION**

Leishmaniasis was described in Nicaragua for the first time in 1917. Four clinical forms are present in the country: conventional CL, MCL, atypical CL, and VL [1]. CL is widespread in the central highlands and the Atlantic lowland plains and it is caused by *L.braziliensis* and *L.panamensis* [2], although a hybrid of both species has been characterized in almost one third of the cases in the north of the country [3] .

Like in other countries in central America, atypical non-ulcerative CL caused by *L.infantum* is present, more specifically in the Pacific region, and may or may not be caused by previous VL [2,4]. It has been suggested that these cases, with atypical CL, could be a potential reservoir for *L.infantum*, and should be targeted in the control program [4].

CL incidence is approximately 3.8/100,000 inhabitants, which makes this the country with the highest number of CL and MCL cases in Central America.

VL was first reported in 1994 [5].

**PARASITOLOGICAL INFORMATION**

| ***Leishmania* species** | **Clinical form** | **Vector species** | **Reservoirs** |
| --- | --- | --- | --- |
| *L. infantum* | ZVL, CL | *Lu. longipalpis, Lu. evansi* | *Canis familiaris* |
| *L. panamensis* | ZCL | *Lu. trapidoi, Lu. ylephiletor,*  *Lu. cruciata, Lu. panamensis* | unknown |
| *L. braziliensis* | ZCL, MCL | *Lu. panamensis* | unknown |

**MAPS AND TRENDS**

**Visceral leishmaniasis**

**
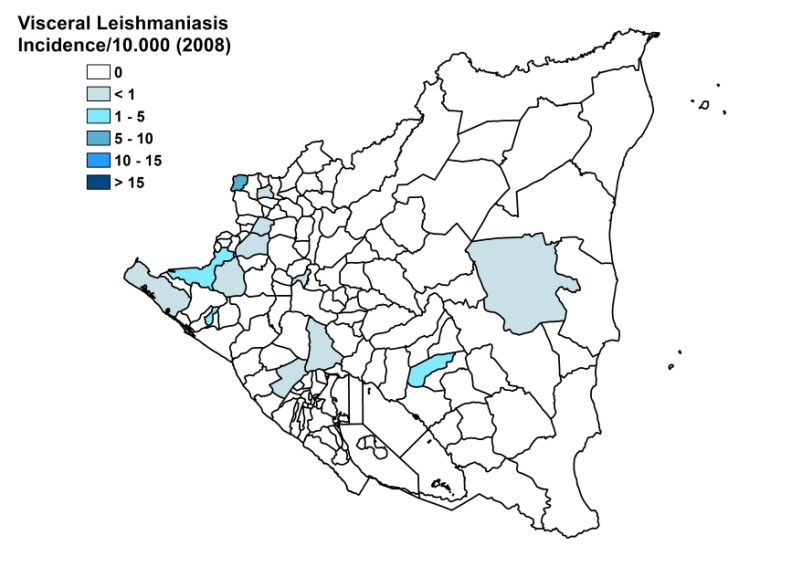

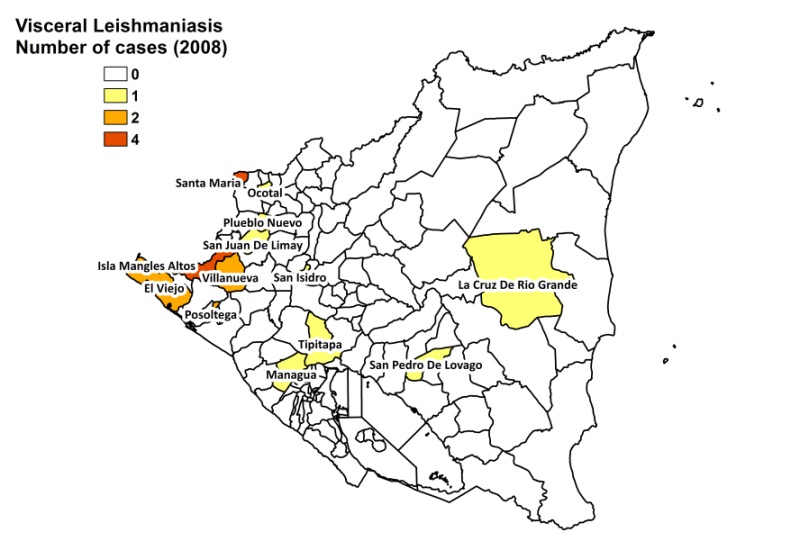
**

**Cutaneous leishmaniasis**

**
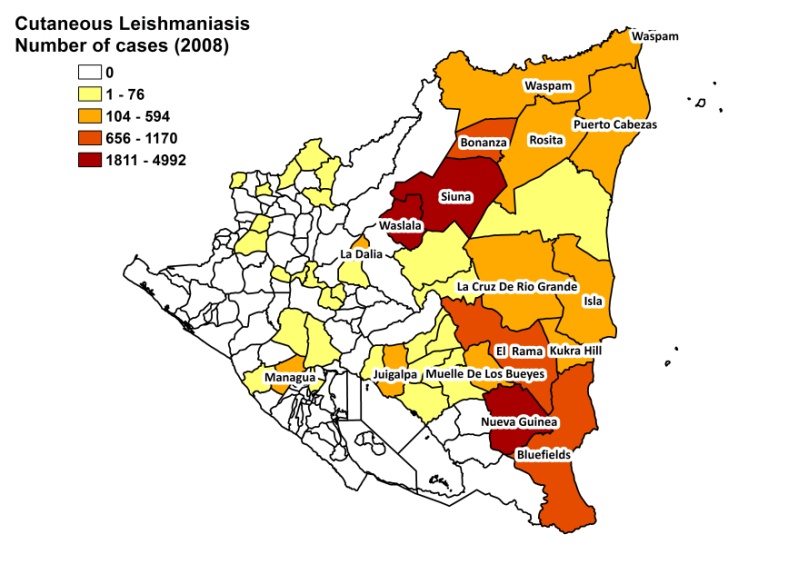

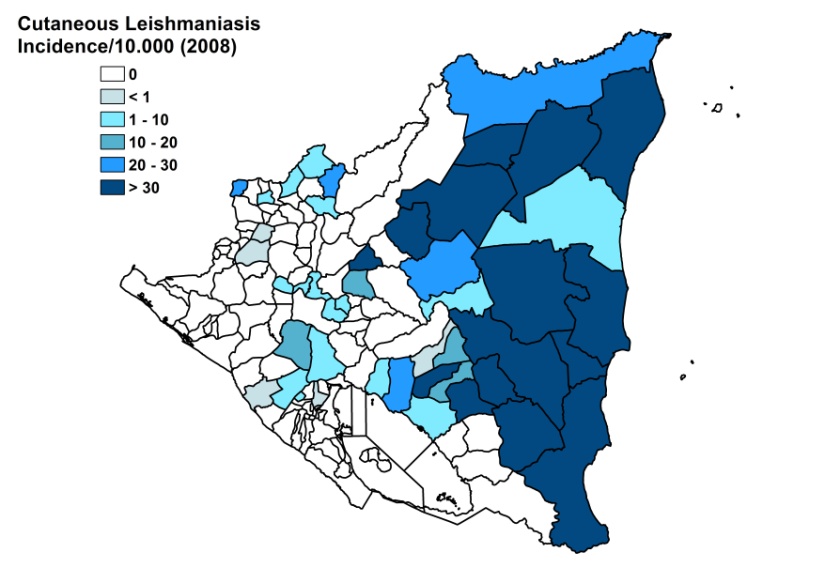
**

**Cutaneous leishmaniasis trend**

**Number of cutaneous and mucocutaneous leishmaniasis cases**

|  | **2008** | **2009** | **2010** |
| --- | --- | --- | --- |
| CL cases | 5941 | 4110 | 3570 |
| MCL cases | 34 | 30 | 31 |
| MCL % of CL cases | 5.6 | 7.2 | 8.6 |

**Visceral leishmaniasis cases**

| **2003** | **2004** | **2005** | **2006** | **2007** | **2008** | **2009** | **2010** |
| --- | --- | --- | --- | --- | --- | --- | --- |
| 5 | 5 | 1 | 1 | 1 | 4 | 1 | 1 |

**CONTROL**

Leishmaniasis has been a notifiable disease since 1980. A leishmaniasis control program was established in 1994. The vector-control strategy adopted relies on controlling foci (spatial spraying in a 500 m radius, using a portable motorized sprayer) and using treated bednets. There is under-reporting of the disease and a lack of systematic monitoring of patients and focused vector-control measures.

**DIAGNOSIS, TREATMENT**

**Diagnosis:**

CL: microscopic examination of skin lesion sample, Montenegro skin test, IFAT, PCR.

VL: IFAT, PCR.

**Treatment:**

CL: antimonials.

VL: antimonials.

**ACCESS TO CARE**

The Ministry of Health provides 95% coverage and is assisted by two NGO’s, that help carry out some activities, such as staff training, drug procurement and medical consultations, etc.

**ACCESS TO DRUGS**

Glucantime (Sanofi) is registered.

**SOURCES OF INFORMATION**

- Dr Emperatriz Lugo. *Leishmaniasis en la Región de las Américas. Reunión de coordinadores de Programa Nacional de Leishmaniasis. OPS/OMS. Medellín, Colombia. 4-6 junio 2008.*

1. [Missoni E](http://www.ncbi.nlm.nih.gov/pubmed?term=%22Missoni%20E%22%5BAuthor%5D), [Morelli R](http://www.ncbi.nlm.nih.gov/pubmed?term=%22Morelli%20R%22%5BAuthor%5D) (1984). Survey of 259 cases of American cutaneous leishmaniasis in Nicaragua. [J Trop Med Hyg](http://www.ncbi.nlm.nih.gov/pubmed/6520890##) 87:159-65.

2. Darce M, Moran J, Palacios X, Belli A, Gomez-Urcuyo F et al (1991). [Etiology of human cutaneous leishmaniasis in [Etiology of human cutaneous leishmaniasis in Nicaragua.](http://www.ncbi.nlm.nih.gov/pubmed/2068761) Trans R Soc Trop Med Hyg 85(1):58-9.](http://www.ncbi.nlm.nih.gov/pubmed/2068761)

3. [Belli AA](http://www.ncbi.nlm.nih.gov/pubmed?term=%22Belli%20AA%22%5BAuthor%5D), [Miles MA](http://www.ncbi.nlm.nih.gov/pubmed?term=%22Miles%20MA%22%5BAuthor%5D), [Kelly JM](http://www.ncbi.nlm.nih.gov/pubmed?term=%22Kelly%20JM%22%5BAuthor%5D) (1994). A putative Leishmania panamensis/Leishmania braziliensis hybrid is a causative agent of human cutaneous leishmaniasis in Nicaragua. [Parasitology](http://www.ncbi.nlm.nih.gov/pubmed/7800411##) 109 (4):435-42.

4. [Belli A](http://www.ncbi.nlm.nih.gov/pubmed?term=%22Belli%20A%22%5BAuthor%5D), [García D](http://www.ncbi.nlm.nih.gov/pubmed?term=%22Garc%C3%ADa%20D%22%5BAuthor%5D), [Palacios X](http://www.ncbi.nlm.nih.gov/pubmed?term=%22Palacios%20X%22%5BAuthor%5D), [Rodriguez B](http://www.ncbi.nlm.nih.gov/pubmed?term=%22Rodriguez%20B%22%5BAuthor%5D), [Valle S](http://www.ncbi.nlm.nih.gov/pubmed?term=%22Valle%20S%22%5BAuthor%5D) et al (1999). Widespread atypical cutaneous Leishmaniasis caused by Leishmania (L.) chagasi in Nicaragua. [Am J Trop Med Hyg](http://www.ncbi.nlm.nih.gov/pubmed/10497975##) 61:380-5.

5. Duarte Z, Munguia J, Orozco M, Gantier JC (1994) First report of visceral leishmaniasis in Nicaragua. Trans R Soc Trop Med Hyg 88: 38.
