# Supplementary material for: Leishmaniasis Worldwide and Global Estimates of Its Incidence
Source: PLoS One. 2012 May 31;7(5):e35671. doi: 10.1371/journal.pone.0035671 (PMC3365071; doi:10.1371/journal.pone.0035671)
Supplement: Text S67 — Leishmaniasis Country Profiles, Niger. (DOCX) [file pone.0035671.s067.docx]

**NIGER**


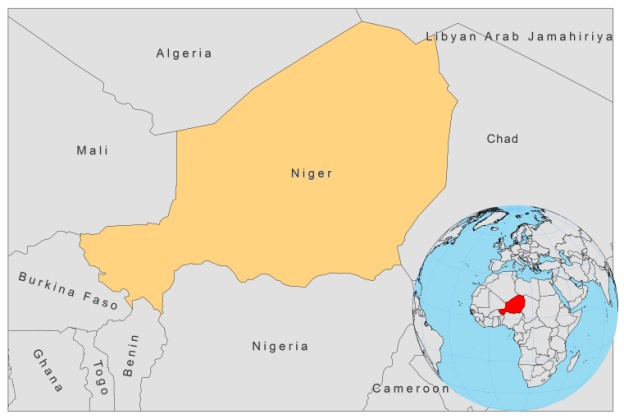


**BASIC COUNTRY DATA**

Total Population: 15,511,953

Population 0-14 years: 49%

Rural population: 83%

Population living under USD 1.25 a day: 43.1%

Population living under the national poverty line: 59.5%

Income status: Low income economy

Ranking: Low human development (ranking 186)

Per capita total expenditure on health at average exchange rate (US dollar): 21

Life expectancy at birth (years): 54

Healthy life expectancy at birth (years): 36

**BACKGROUND INFORMATION**

From 1981 to 1987, 9 VL autochtonous cases were reported from the Aïr mountains, near the Algerian border: one case was confirmed parasitologically and the others serologically. In 1987, a new case from Zinder, in southeast Niger, was parasitologically proven [1]. Between January 1992 and January 1995, six further cases were found in young soldiers in Niamey, Niger. All of them had previously spent time in the Aïr mountains [2]. No further VL has been documented. VL’s prevalence is probably much higher and widespread than these reported figures reflect.

The first CL case found in West Africa was reported in Niger in 1911 [3]. A second case was reported in 1943 [4], and 64 more cases between 1984-1990, mostly from rural areas. No further cases have been documented. As Niger is part of a proposed CL endemicity belt, running across West Africa, cases may still occur and go unreported.

**PARASITOLOGICAL INFORMATION**

| ***Leishmania* species** | **Clinical form** | **Vector species** | **Reservoirs** |
| --- | --- | --- | --- |
| *L. major* | ZCL | *P. duboscqi* | Unknown |
| Unknown | VL | Unknown | Unknown |

**MAPS AND TRENDS, CONTROL, DIAGNOSIS, TREATMENT**

No information available.

**ACCESS TO CARE**

Between 1992 and 1995, two patients died because they did not have access to antimonials on time.

**ACCESS TO DRUGS**

No antimonials are registered.

**SOURCES OF INFORMATION**

1. Desjeux P (1991) Information on the epidemiology and control of the leishmaniases by country or territory. World Health Organization. WHO/LEISH/91.30.

2. Djidingar D, Chippaux JP, Gragnic G, Tchani O, Meynard D et al (1997). Visceral leishmaniasis in Niger: six new parasitologically confirmed cases Bull Soc Pathol Exot 90(1):27-9.

3. Stevenel (1911). Les Cro-cro de la region de Zinder et leur identification avec l'ulcere phagedenique des pays chaud, et le bouton d'Orient. Bull Soc Path Exotique 4:80, I2 April.

4. Parro L, Gougis R (1943). Sur l’argent probable de la transmission du bouton d’Orient dans la colonie de Niger. Arch Institut Pasteur d’Algerie 21: 268-269.
