# Supplementary material for: Leishmaniasis Worldwide and Global Estimates of Its Incidence
Source: PLoS One. 2012 May 31;7(5):e35671. doi: 10.1371/journal.pone.0035671 (PMC3365071; doi:10.1371/journal.pone.0035671)
Supplement: Text S68 — Leishmaniasis Country Profiles, Nigeria. (DOCX) [file pone.0035671.s068.docx]

**NIGERIA**


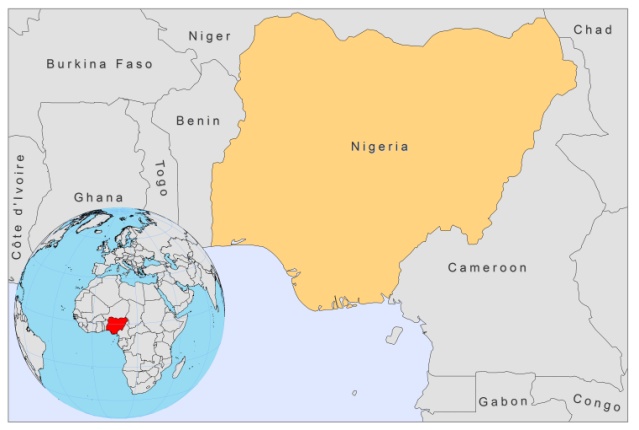


**BASIC COUNTRY DATA**

Total Population: 158,423,182

Population 0-14 years: 43%

Rural population: 50%

Population living under USD 1.25 a day: no data

Population living under the national poverty line: no data

Income status: Lower middle income economy

Ranking: Low human development (ranking 156)

Per capita total expenditure on health at average exchange rate (US dollar): 69

Life expectancy at birth (years): 51

Healthy life expectancy at birth (years): 42

**BACKGROUND INFORMATION**

The first case of CL was reported in 1924 [1]. Foci for CL are in the northwestern part and in the Plateau state, located in central Nigeria. An outbreak occurred at a secondary school in Sokoto, in 2003; 10% of the school children were affected [2].

A study in school children from the Plateau area showed that CL was confirmed in 63 out of 1,120 cases [3]. A recent house-to-house survey in this area found CL in 18 (2.6%) of a population of 703 inhabitants of the area [4]. In another study in Kaena, in the Plateau state, 197 (3.9%) of 5,046 inhabitants had CL [5]. CL is likely to be vastly underreported.

In 2010 a CL/HIV co-infection was diagnosed in a 38-year-old woman [6].

**PARASITOLOGICAL INFORMATION**

| ***Leishmania* species** | **Clinical form** | **Vector species** | **Reservoirs** |
| --- | --- | --- | --- |
| *L. major* | ZCL | *P. duboscqi, P. rodhaini* | Unknown |

**MAPS AND TRENDS**

**Cutaneous leishmaniasis**

**
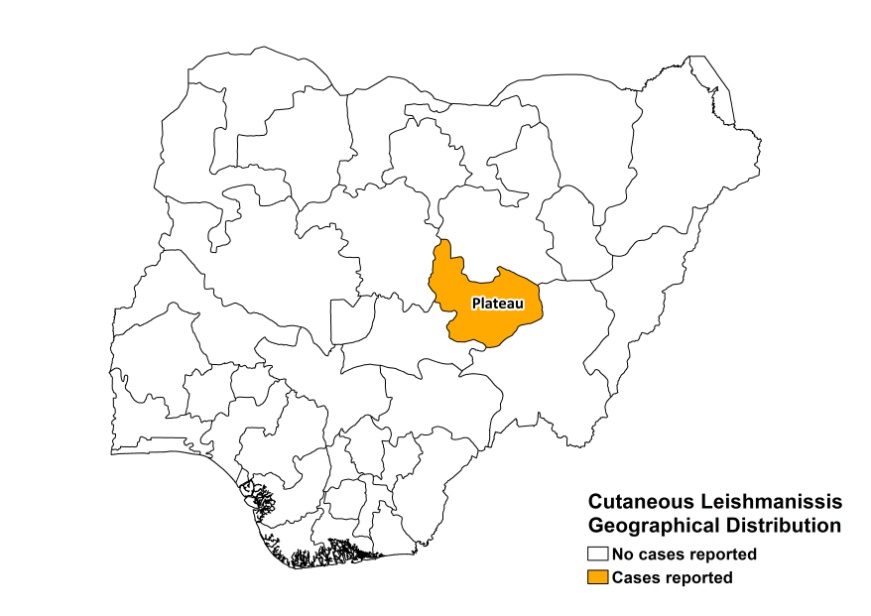
**

**Visceral leishmaniasis trend**

**Cutaneous leishmaniasis trend**

**CONTROL, DIAGNOSIS, TREATMENT**

No information available.

**ACCESS TO CARE**

Between 1992 and 1995, two patients died because they did not have access to antimonials in time.

**ACCESS TO DRUGS**

No antimonials are registered.

**SOURCES OF INFORMATION**

1. Dyce-Shar (1924). Oriental sore in Nigeria. Trans Roy Soc Trop Med Hyg 1924; 18: 336.

2. Jiya NM, Ahmed H, Jibrin B, Philips AO (2007). An outbreak of Cutaneous Leishmaniasis in a Boarding Senior Secondary School in Sokoto, North Western Nigeria: Clinical Presentation and Outcome. Nigerian Medical Practitioner 5: 86-89.

3 Agwale SM, Dondji B, Okolo CJ, Duhlinska DD (1993). Clinical and parasitological prevalence of leishmaniasis in school children in Keana, Awe, L.G.A. of Plateau State. Nigeria. 1993. Mem Inst Oswaldo Cruz 88:347.

4. Igbe M, Duhlinska D, Agwale S (2009). Epidemiological survey of cutaneous leishmaniasis in Jos East L.G.A. Of Plateau State Nigeria. The Internet Journal of Parasitic Diseases 4 (1).

5. Ike El, Ajayi JA, Bello CSS (1993). Outbreak of human Cutaneous leishmaniasis in Keana District of Plateau State Nigeria. A preliminary communication. Niger Med J 24 (3): 101 – 102.

6. Yusuf SM, Uloko AE, Adamu HA, Iliyasu G, Mohammed AM (2010). [Disseminated cutaneous leishmaniasis in HIV positive patient--a case report.](http://www.ncbi.nlm.nih.gov/pubmed/20232766) Niger J Med 19(1):112-4.
