# Supplementary material for: Leishmaniasis Worldwide and Global Estimates of Its Incidence
Source: PLoS One. 2012 May 31;7(5):e35671. doi: 10.1371/journal.pone.0035671 (PMC3365071; doi:10.1371/journal.pone.0035671)
Supplement: Text S69 — Leishmaniasis Country Profiles, Oman. (DOCX) [file pone.0035671.s069.docx]

**OMAN**


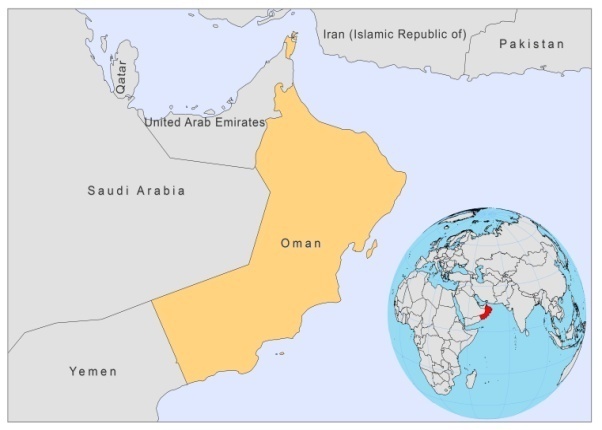


**BASIC COUNTRY DATA**

Total Population: 2,782,435

Population 0-14 years: 27%

Rural population: 28%

Population living under USD 1.25 a day: no data

Population living under the national poverty line: no data

Income status: High income economy

Ranking: High human development (ranking 89)

Per capita total expenditure on health at average exchange rate (US dollar): 497

Life expectancy at birth (years): 73

Healthy life expectancy at birth (years): 64

**BACKGROUND INFORMATION**

VL has been reported since the 1970s, mainly in children living in the foothills of the northern mountains and in southern Dhofar province. In the early 1990s, the annual incidence was low, but in 1992, there was a sudden rise in cases. Between 1993 and 1999, 33 children were treated in the Sultan Qaboos University Hospital [1]. Epidemiological & entomological studies around the patients’ houses indicated that children at the periphery of villages, close to rocky terrain, are most at risk. Spraying operations were conducted in the affected areas of Shahan in order to interrupt the transmission. The number of cases has reduced since then. In recent years, only sporadic cases (less than 10 per year) were recorded, several of which from border areas with Yemen. In 2008, no VL cases were reported.

CL was not reported until 1986; however, that same year, 50 cases were reported, 43 of which were individuals from Sudan, following a major outbreak of CL in Khartoum. In 1998, 25 CL cases were reported [2]. *L. tropica* was characterized as causative agent in one patient. However, this patient had been in Pakistan 18 months earlier, and could, therefore, not be confirmed as an autochtonous case [3]. Currently, CL is reported only sporadically in Oman, from northern and southern Dhofar region. The reduction in the number of cases could be explained by malaria activities that led to a decrease in the sandfly population, and by the fact that less people now sleep outside. According to health workers in the endemic regions, CL is not underreported.

There is currently neither any known evidence of active transmission in any part of the country nor has an existing animal reservoir been established; therefore, leishmaniasis does not form a public health threat in Oman at this point.

In 2008 one case of *Leishmania/*HIV co-infection due to *L. infantum* was reported [4].

**PARASITOLOGICAL INFORMATION**

| ***Leishmania* species** | **Clinical form** | **Vector species** | **Reservoirs** |
| --- | --- | --- | --- |
| *L. infantum* | ZVL | *P. alexandri* | *Canis familiaris* |
| *L. major* | ZCL | *P. papatasi* | unknown |

**MAPS AND TRENDS**

**Cutaneous leishmaniasis**

**
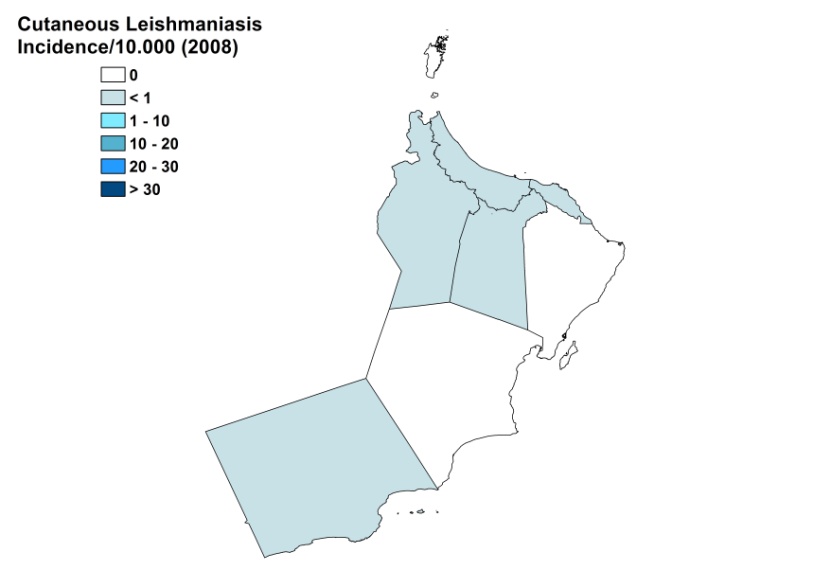

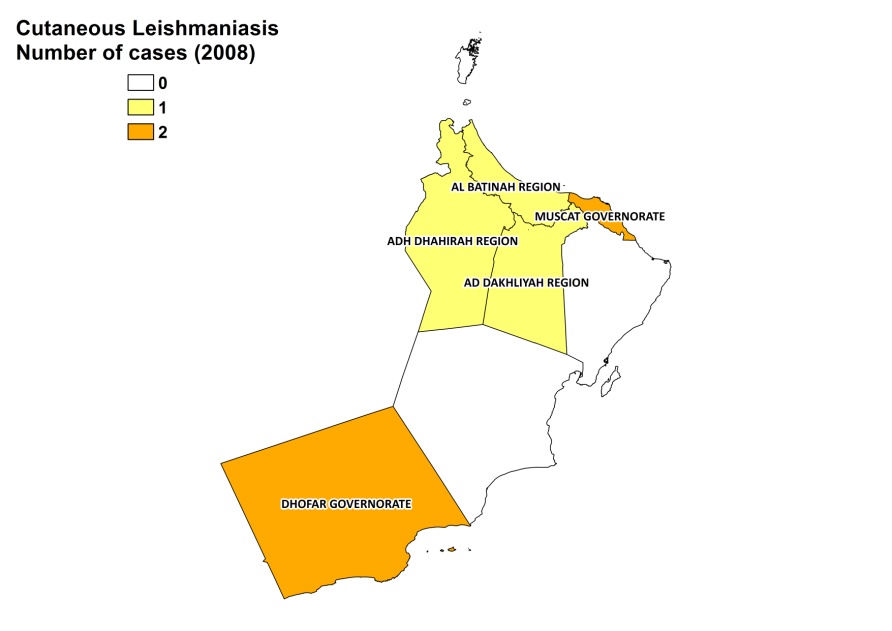
**

**Visceral leishmaniasis trend**

**Cutaneous leishmaniasis trend**

**CONTROL**

The notification of leishmaniasis is mandatory. There is no national leishmaniasis control program, but since 1991, leishmaniasis has been integrated in the Communicable Disease Surveillance & Control. Case detection is passive. There is no leishmaniasis vector or reservoir control program.

**DIAGNOSIS, TREATMENT**

**Diagnosis**

VL: confirmation by microscopic examination of bone marrow aspirate or a serological method (IFAT, ELISA, DAT).

CL: on clinical grounds and confirmation by microscopic examination of skin lesion sample.

**Treatment**

VL: antimonials, 20 mg Sb^v^/kg/day for 30 days. Cure rate is 100%.

CL: antimonials, intralesional or systemic. Cure rate is 100%.

**ACCESS TO CARE**

Medical care is officially provided for free in Oman, which includes care for leishmaniasis. Treatment by antimonials (Pentostam, GSK) is available in all referral hospitals. All patients are thought to have access to care.

**ACCESS TO DRUGS**

Sodium stibogluconate (Pentostam) is registered and included in the National Essential Drug List for leishmaniasis. Pentostam (GSK) is the only antimonial registered in Oman.

**SOURCES OF INFORMATION**

- Dr Idris Al-Abaidani, Department of Communicable Disease Surveillance & Control Leishmaniasis in Oman, 2009. Directorate General of Health Affairs, Ministry of Health, Oman.

1. Elnour IB, Akinbami FO, Shakeel A, Venugopalan P (2001). [Visceral leishmaniasis in Omani children: a review.](http://www.ncbi.nlm.nih.gov/pubmed/11471261) Ann Trop Paediatr 21(2):159-63.

2. Scrimgeour EM, Mehta FR, Suleiman AJ (1991). [Infectious and tropical diseases in Oman: a review.](http://www.ncbi.nlm.nih.gov/pubmed/10674671) Am J Trop Med Hyg 61(6):920-5.

3. Scrimgeour EM, Windsor JJ, Shetty MK, Banodkar DD, Lambson B, et al (1999). [Leishmania tropica is a probable cause of cutaneous leishmaniasis in the Sultanate of Oman: case report in a Pakistani resident.](http://www.ncbi.nlm.nih.gov/pubmed/10492747) Trans R Soc Trop Med Hyg 93(3):233-4.

4. Balkhair A, Ben Abid F (2008). [Gastric and cutaneous dissemination of visceral leishmaniasis in a patient with advanced HIV.](http://www.ncbi.nlm.nih.gov/pubmed/17643333) Int J Infect Dis 12(1):111-3.
