# Supplementary material for: Leishmaniasis Worldwide and Global Estimates of Its Incidence
Source: PLoS One. 2012 May 31;7(5):e35671. doi: 10.1371/journal.pone.0035671 (PMC3365071; doi:10.1371/journal.pone.0035671)
Supplement: Text S70 — Leishmaniasis Country Profiles, Pakistan. (DOCX) [file pone.0035671.s070.docx]

**PAKISTAN**

**
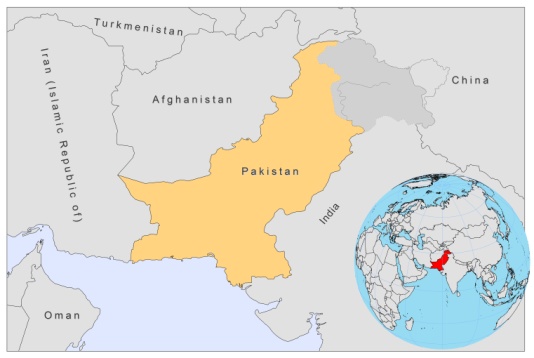
**

**BASIC COUNTRY DATA**

Total Population: 173,593,383

Population 0-14 years: 35%

Rural population: 63%

Population living under 1.25 USD a day: 22.6%

Population living under the national poverty line: 22.3%

Income status: Lower middle income economy

Ranking: Low human development (ranking 145)

Per capita total expenditure on health at average exchange rate (US dollar): 23

Life expectancy at birth (years): 65

Healthy life expectancy at birth (years): 53

**BACKGROUND INFORMATION**

VL was first reported in 1960, and now sporadically occurs in the Northern Areas and neighbouring parts of North West Frontier Province (NWFP) and Punjab. The transmission occurs mostly between the altitude of 1500 to1800 meters, where the valley population usually migrate to during summer months. Most cases are children under 10 years old. Serological surveys indicate the existence of many asymptomatic or self-curing infections. Currently, the amount of VL cases is unknown, as no reporting system is in place.

CL is a major and fast increasing public health problem, both among the local Pakistani population and the Afghan refugees in camps. Its extensive spreading has been associated with mass migration, from endemic to non-endemic areas and vice versa, and with Afghan refugees from areas where CL is highly endemic. Outbreaks are frequent, for example, early 2011, in Mianwali, Punjab province, which is a bordering district with Kyber Pakhtunkhwa province, and probably cases have come from Afghan refugees residing in the area. This was the second outbreak in Punjab province since the first one, in Chakwal, in 2004. Two causative parasites are present: CL by *L. major* is mainly occurring in Baluchistan and neighbouring Punjab and Sindh provinces, while CL by *L. tropica* has the widest distribution and is prevalent in urban areas of southern Punjab (Multan) and Balochistan (Quetta), but also focally in the Northern Areas. Cases have increasingly been reported from towns of NWFP and Balochistan. In a large survey held in 2002-2003, in NWFP province [1], the prevalence of active CL in refugee camps was found to be 2.7% and the prevalence of scars 4.2%. In neighbouring villages, active CL was prevalent in 1.7% of cases, and scars in 4.9%. Active CL was mostly found in children between 6 and 15 years old. CL was strongly associated with poverty.

In 1997, a large CL outbreak was reported from Timargara refugee camp in NWFP [2]. Survey results showed that 38% of the camp population was affected during the outbreak, compared to 17% the previous year, suggesting that the outbreak originated within the camp. *L. tropica* was confirmed [2]. An epidemic occurred in Kurrum, in the Federally Administered Tribal Area (FATA), in October 2001, with an estimated 2,000 cases. A WHO conducted survey showed that the population was new to VL in the last 1-3 years and that they had very little knowledge about its spreading mode or about control measures. Another outbreak, in Larkana and Dadu city, in Sindh province, from December 2001-February 2002 caused an estimated 11,700 cases. The mean age of cases was 17 during this outbreak, and the male-female ratio was 1. Current (2009-2010) high case loads in hospitals suggest another outbreak in FATA, Khyber Agency (Bara), and Torkhum (bordering Afghanistan). CL is now estimated to affect 15,000-20,000 people in Pakistan yearly.

CL causes severe stigma in Pakistan. Due to a lack of awareness of the nature and mode of transmission of the disease, people with lesions and scars are victimized and excluded from communal life. Especially young women are affected and are often deemed unfit for marriage and child rearing [3].

No HIV/*Leishmania* co-infection has been reported.

**PARASITOLOGICAL INFORMATION**

| **Leishmania species** | **Clinical form** | **Vector species** | **Reservoirs** |
| --- | --- | --- | --- |
| *L. infantum* | ZVL | *unknown* | *Canis familiaris* |
| *L. tropica* | ACL | *P.sergenti* | Human |
| *L. major* | ZCL | *P.papatasi, P.salehi* | *Meriones hurrianae, Rhombomys. opimus, Tatera indica* |

**MAPS AND TRENDS**

**Cutaneous leishmaniasis: local cases**

**
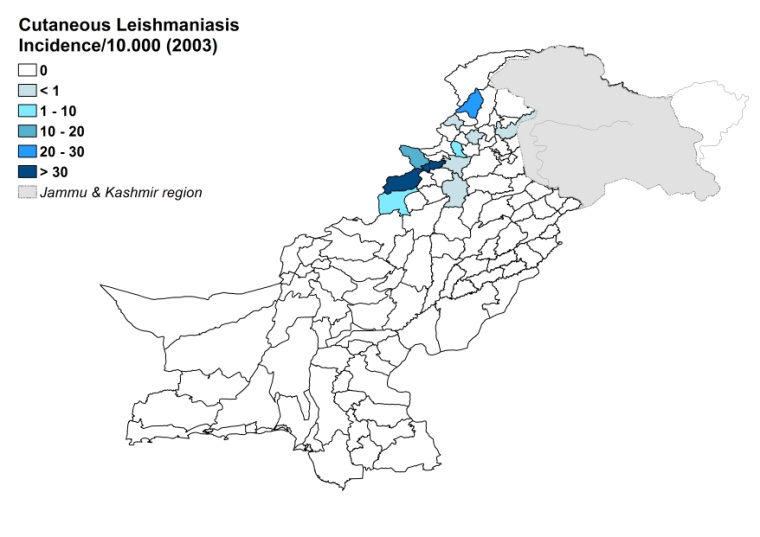

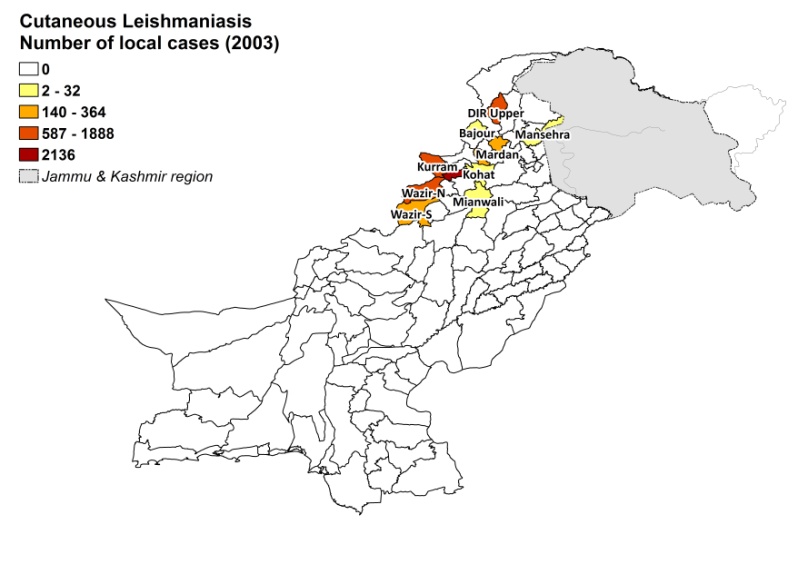
**

**Cutaneous leishmaniasis: refugee camps**

**
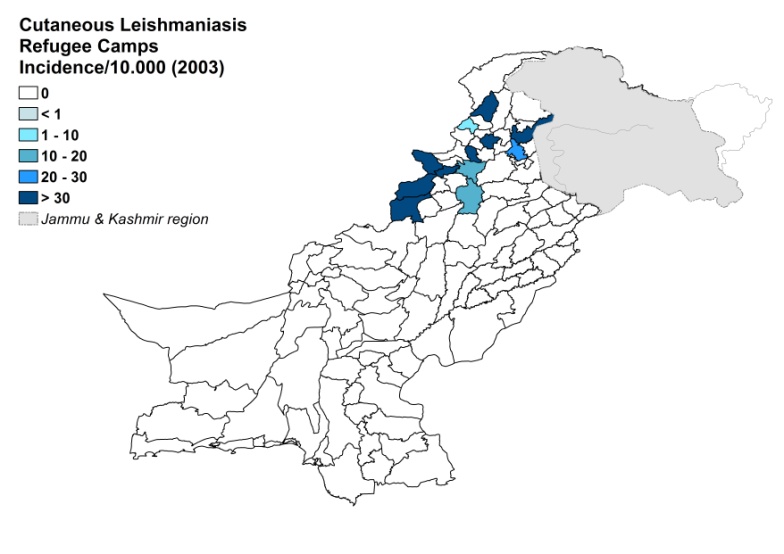

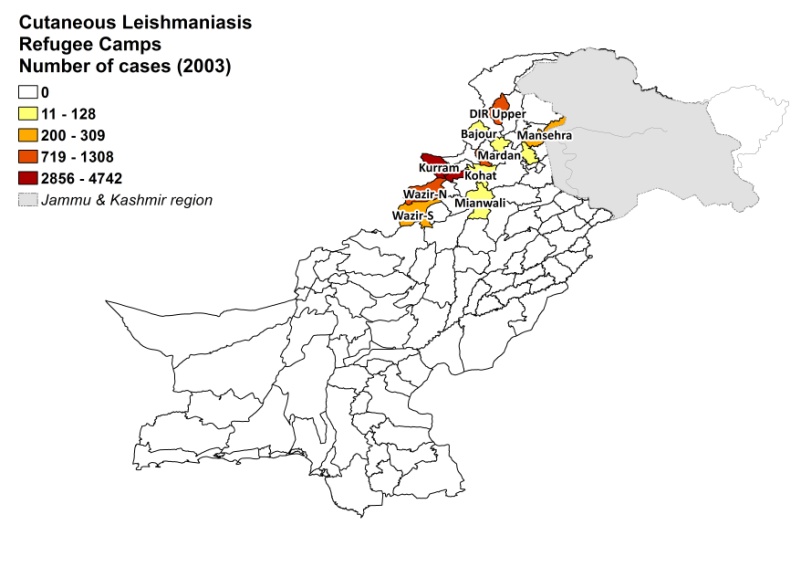
**

**Cutaneous leishmaniasis trend**

**Cutaneous leishmaniasis cases according to infection origin**

**CONTROL**

The notification of leishmaniasis is not mandatory in the country. There is no national leishmaniasis control program and no leishmaniasis vector control program, but bednet distribution takes place in refugee camps. There is no leishmaniasis reservoir program.

**DIAGNOSIS, TREATMENT**

**Diagnosis**

CL: mostly on clinical grounds. Confirmation by microscopic examination of a skin lesion sample.

**Treatment**

CL: antimonials, intralesional or systemic (20 mg Sb^v^/kg/day). Cure rate for local therapy is

94% and 95% for systemic therapy. In FATA, thermal therapy is used.

**ACCESS TO CARE**

An estimated 72% of patients are unable to access medical treatment, mainly because treatment is only provided for free in a few hospitals and in treatment centers in refugee camps. Most government hospitals do not have any drugs or skilled staff for the treatment of leishmaniasis. Patients cannot afford to buy the drugs themselves, nor can they pay the cost of travelling long distances in order to access free treatment. Most patients suffer major economic loss when spending time away from home. Between 2000 and 2005, 22,126 people of the local population sought free treatment in the Afghan refugee camps. Gender inequality in seeking treatment is very common; women are often not allowed treatment by their husbands. There is a great lack of awareness of leishmaniasis, which causes many to not seek treatment at all. An estimated 20-30% of patients use the private sector for diagnosis and treatment, which is mostly substandard.

**DRUG ACCESS**

Meglumine antimoniate and sodium stibogluconate are included in the National Essential Drug List for CL. Antimonials are difficult to obtain and not widely available in Pakistan. Meglumine antimoniate (Meglutin, Star Laboratories, Pakistan) was recently registered and is sold in some private pharmacies and illegal drugs markets for 0.6 and 1.7-3 USD per vial respectively. Glucantime (Sanofi) is smuggled into Pakistan and sold for 4-5 USD per vial (leading to a treatment cost of around 150 USD for systemic CL treatment).

**Sources of information**

- Dr Akhtar Muneer, Kuwait Teaching Hospital (Leishmaniasis Reference centre, NWFP).
- Dr Nazma Habib Khan, London School of Hygiene and Tropical Medicine, UK.
- Dr Azam Samdani, head of dept of dermatology, Aga Khan hospital.
- Dr Chaudhry Muhammad Arif Munir, Pakistan Medical Research Council, Off Constitution. *WHO Consultative meeting on Cutaneous Leishmaniasis in EMRO countries, Geneva, 30 April to 2 May 2007.*

1. Brooker S, Mohammed N, Adil K, Agha S, Reithinger R et al (2004). Leishmaniasis in refugee and local Pakistani populations. Emerg Infect Dis 10(9):1681-4.

2. Rowland M, Munir A, Durrani N, Noyes H, Reyburn H (1999). An outbreak of cutaneous leishmaniasis in an Afghan refugee settlement in north-west Pakistan. Trans R Soc Trop Med Hyg 93(2):133-6.

3. Kassi M, Afghan AK, Rehman R, Kasi PM (2008). Marring leishmaniasis: the stigmatization and the impact of cutaneous leishmaniasis in Pakistan and Afghanistan. PLoS Negl Trop Dis 2(10):e259.
