# Supplementary material for: Leishmaniasis Worldwide and Global Estimates of Its Incidence
Source: PLoS One. 2012 May 31;7(5):e35671. doi: 10.1371/journal.pone.0035671 (PMC3365071; doi:10.1371/journal.pone.0035671)
Supplement: Text S71 — Leishmaniasis Country Profiles, Panama. (DOCX) [file pone.0035671.s071.docx]

**PANAMA**


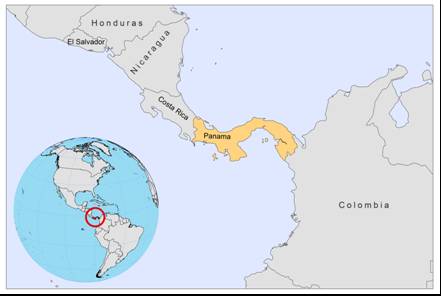


**BASIC COUNTRY DATA**

Total Population: 3,516,820

Population 0-14 years: 29%

Rural population: 25%

Population living under USD 1.25 a day: 9.5%

Population living under the national poverty line: 32.7%

Income status: Upper middle income economy

Ranking: High human development (ranking 58)

Per capita total expenditure on health at average exchange rate (US dollar): 591

Life expectancy at birth (years): 76

Healthy life expectancy at birth (years): 66

**BACKGROUND INFORMATION**

CL was reported for the first time in 1911. Since 1977, the number of cases has been increasing. CL occurs throughout the year, with a peak in May, at the beginning of the rainy season, when there is increased agricultural activity, and a second peak in October and November, during the harvest, which is also a rainy period [1,2]. *L. panamensis* is responsible for the great majority of CL cases, mostly in forested areas from west to east, including Bocas del Toro, Coclé, Colón, Veraguas, Panama, San Blas, and Darien provinces. The two-toed sloth is the main reservoir, reaching prevalences of up to 48% [3]. 9% of dogs were found infected, but are most likely not playing a role in transmission [4]. *L. mexicana* is also present, overlapping in north-central Panama and in the Darién forest, although not much is known about the epidemiology of this species in Panama, apart from reports on subclinical infections [5]. Other *Leishmania* species (i.e., *L.colombiensis*) are found in mammals, but not in humans.

The main affected population groups are settlers that live close to the forest and male young adults who work in the forest (prevalence of 60% among males; 50% of cases is under 15 years of age) ^[6]^. Infection among new settlers can reach up to 42% in an exposure time ranking from one to 14 months [7]. In the protected forest of the Panama Canal area, an outbreak occurred in 1984, with an attack rate of 22% in a short period of time, ranking from 2 to 78 days (average of 17 days) [8]. The annual incidence (number of individuals with lesions or scars acquired annually) and prevalence (those exhibiting lesions or scars at any given moment) vary according to areas, but can be as high as 27% and 77%, respectively, in the San Miguel area [9].

Almost 5% of the patients evolve to MCL. Associated risk factors are being female and a lack of past CL treatment [1,10].

VL is unknown, but *Lu. longipalpis* is present. Considering that Panama is a biological corridor between Central and South American countries, where *L.infantum* exists, the possibility of introduction into Panama cannot be ruled out.

**PARASITOLOGICAL INFORMATION**

| ***Leishmania* species** | **Clinical form** | **Vector species** | **Reservoirs** |
| --- | --- | --- | --- |
| *L. panamensis* | ZCL, MCL | *Lu. trapidoi, Lu. ylephiletor,*  *Lu. gomezi, Lu. sanguinaria,*  *Lu. panamensis,* | *Choloepus hoffmanni* |
| *L. braziliensis* | ZCL | *Lu. panamensis* | Unknown |
| *L. colombiensis* | ZCL | unknown | *Choloepus hoffmanni* |

**MAPS AND TRENDS**

**Cutaneous leishmaniasis**

**
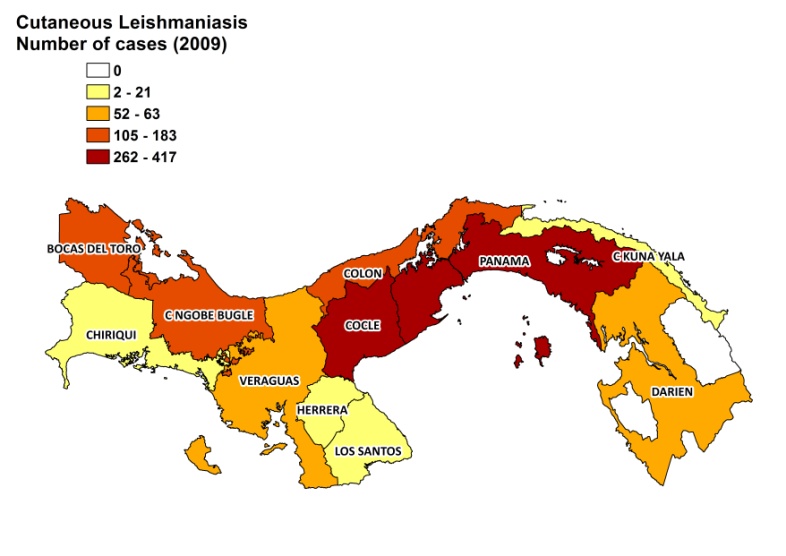

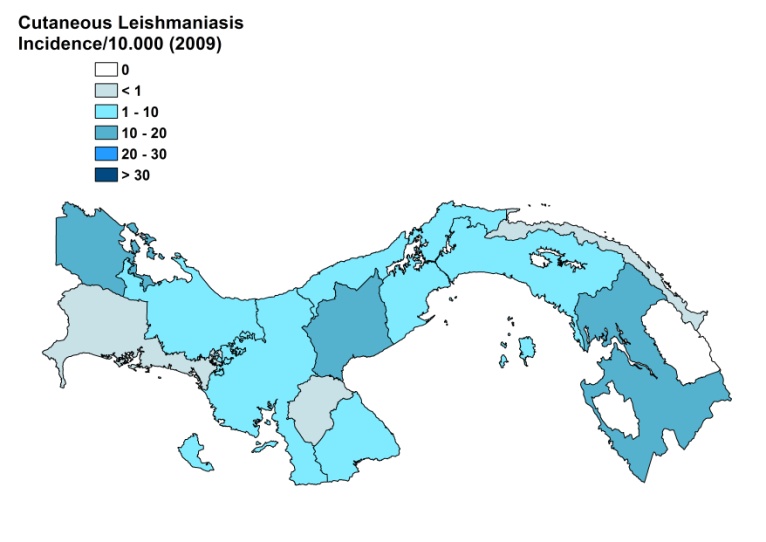
**

**Cutaneous leishmaniasis trend**

**CONTROL**

An epidemiological surveillance system has been in place since 1980, and notification of leishmaniasis is mandatory, but there is no national program. Case detection and registration is passive. Vector control, which relies on education and spraying, is carried out whenever cases are detected.

**DIAGNOSIS, TREATMENT**

**Diagnosis**: microscopy of skin lesions samples.

**Treatment:**

CL: antimonials

VL: antimonials

**ACCESS TO CARE**

No information available

**ACCESS TO DRUGS**

Meglumine antimoniate (Glucantime, Sanofi) is registered.

**SOURCES OF INFORMATION**

1. [Christensen HA](http://www.ncbi.nlm.nih.gov/pubmed?term=%22Christensen%20HA%22%5BAuthor%5D), [Fairchild GB](http://www.ncbi.nlm.nih.gov/pubmed?term=%22Fairchild%20GB%22%5BAuthor%5D), [Herrer A](http://www.ncbi.nlm.nih.gov/pubmed?term=%22Herrer%20A%22%5BAuthor%5D), [Johnson CM](http://www.ncbi.nlm.nih.gov/pubmed?term=%22Johnson%20CM%22%5BAuthor%5D), [Young DG](http://www.ncbi.nlm.nih.gov/pubmed?term=%22Young%20DG%22%5BAuthor%5D), [et](http://www.ncbi.nlm.nih.gov/pubmed?term=%22de%20V%C3%A1squez%20AM%22%5BAuthor%5D) al (1983). The ecology of cutaneous leishmaniasis in the Republic of Panama. [J Med Entomol.](http://www.ncbi.nlm.nih.gov/pubmed/6358496##) 20:463-84.

2. Christensen HA, de Vasquez AM, Petersen JL (1999). Short report: epidemiologic studies on cutaneous leishmaniasis in eastern Panama. Am J Trop Med Hyg 60: 54–57.

3. Herrer A, Christensen HA (1980). [Leishmania braziliensis in the Panamanian two-toed sloth, Choloepus hoffmanni.](http://www.ncbi.nlm.nih.gov/pubmed/7446811) Am J Trop Med Hyg 29(6):1196-200.

4. Herrer A, Christensen HA (1976). [Natural cutaneous leishmaniasis among dogs in Panama.](http://www.ncbi.nlm.nih.gov/pubmed/943961) Am J Trop Med Hyg 25(1):59-63.

5. de Vasquez AM, Saenz RE, Petersen JL (1990). Christensen HA, Johnson CM. [Leishmania mexicana complex: human infections in the Republic of Panama.](http://www.ncbi.nlm.nih.gov/pubmed/2267966) Am J Trop Med Hyg 43(6):619-22.

6. Herrer A, Christensen HA (1976). [Epidemiological patterns of cutaneous leishmaniasis in Panama. I. Epidemics among small groups of settlers.](http://www.ncbi.nlm.nih.gov/pubmed/1267510) Ann Trop Med Parasitol 70(1):59-65.

7. Miranda A, Carrasco R, Paz H, Pascale J, Samudio F et al (2009). Molecular Epidemiology of American Tegumentary Leishmaniasis in Panama. Am J Trop Med Hyg 81: 565–571.

8. [Sanchez JL](http://www.ncbi.nlm.nih.gov/pubmed?term=%22Sanchez%20JL%22%5BAuthor%5D), [Diniega BM](http://www.ncbi.nlm.nih.gov/pubmed?term=%22Diniega%20BM%22%5BAuthor%5D), [Small JW](http://www.ncbi.nlm.nih.gov/pubmed?term=%22Small%20JW%22%5BAuthor%5D), [Miller RN](http://www.ncbi.nlm.nih.gov/pubmed?term=%22Miller%20RN%22%5BAuthor%5D), [Andujar JM](http://www.ncbi.nlm.nih.gov/pubmed?term=%22Andujar%20JM%22%5BAuthor%5D) et al (1992). Epidemiologic investigation of an outbreak of cutaneous leishmaniasis in a defined geographic focus of transmission. [Am J Trop Med Hyg](http://www.ncbi.nlm.nih.gov/pubmed/1636883##) 47:47-54.

9. Christensen HA, de Vasquez AM, Petersen JL (1990). [Short report epidemiologic studies on cutaneous leishmaniasis in eastern Panama.](http://www.ncbi.nlm.nih.gov/pubmed/9988322) Am J Trop Med Hyg 1:54-7.

10. [Sáenz RE](http://www.ncbi.nlm.nih.gov/pubmed?term=%22S%C3%A1enz%20RE%22%5BAuthor%5D), [Paz HM](http://www.ncbi.nlm.nih.gov/pubmed?term=%22Paz%20HM%22%5BAuthor%5D), [de Rodriguez GC](http://www.ncbi.nlm.nih.gov/pubmed?term=%22de%20Rodriguez%20GC%22%5BAuthor%5D), [de Vásquez AM](http://www.ncbi.nlm.nih.gov/pubmed?term=%22de%20V%C3%A1squez%20AM%22%5BAuthor%5D), [Mata RE](http://www.ncbi.nlm.nih.gov/pubmed?term=%22Mata%20RE%22%5BAuthor%5D) [et](http://www.ncbi.nlm.nih.gov/pubmed?term=%22Johnson%20CM%22%5BAuthor%5D) al (1989). Mucocutaneous leishmaniasis in Panama. Etiologic agent, epidemiologic and clinical aspects. [Rev Med Panama.](http://www.ncbi.nlm.nih.gov/pubmed/2727332##) 14:6-15.
