# Supplementary material for: Leishmaniasis Worldwide and Global Estimates of Its Incidence
Source: PLoS One. 2012 May 31;7(5):e35671. doi: 10.1371/journal.pone.0035671 (PMC3365071; doi:10.1371/journal.pone.0035671)
Supplement: Text S72 — Leishmaniasis Country Profiles, Paraguay. (DOCX) [file pone.0035671.s072.docx]

**PARAGUAY**


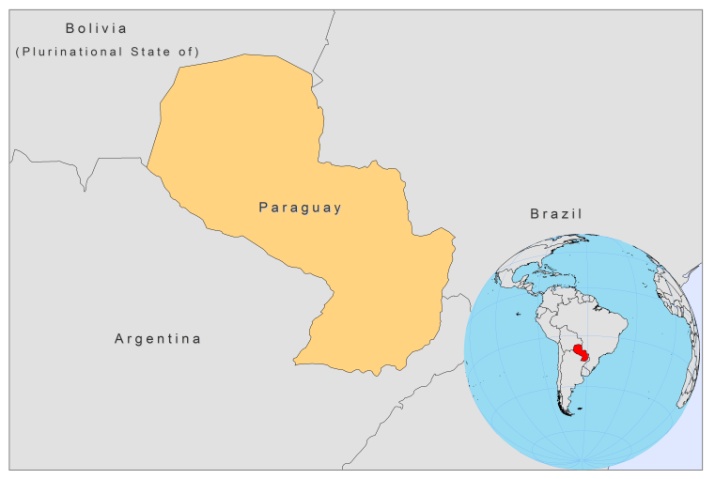


**BASIC COUNTRY DATA**

Total Population: 6,454,548

Population 0-14 years: 34%

Rural population: 39%

Population living under USD 1.25 a day: 5.1%

Population living under the national poverty line: 35.1%

Income status: Lower middle income economy

Ranking: Medium human development (ranking 107)

Per capita total expenditure on health at average exchange rate (US dollar): 159

Life expectancy at birth (years): 72

Healthy life expectancy at birth (years): 62

**BACKGROUND INFORMATION**

Leishmaniasis has been known in Paraguay for many years [1]. The first documented outbreak of CL occurred in 1934 [2]. The incidence of CL has increased since then; between 1972 and 1981, approximately 100-200 cases per year were reported, but in 1982 a sharp increase occurred with over 1,600 cases. In 1983-1984, the epidemiological situation stabilized with 100-200 cases annually. In 1985, 1,083 cases were registered and cases continued to be fluctuating with local outbreaks every few years and gradually increasing case numbers [3]. In 2007, outbreaks occurred in the town of Tava Yopoi (Canindeyu department), where the incidence rate rose to 144.9 per 100,000 inhabitants (243 cases), and in the departments of San Pedro (17.8 per 100,000 inhabitants; 64 cases), Alto Paraná (12.4 per 100,000 inhabitants; 81 cases), and Caaguazú (9.3 per 100,000 inhabitants; 45 cases). MCL accounted for 31% of cases in 2007, and the number of cases was the highest recorded in the world. 64% of CL cases occur in males.

The first VL case in South America was described in Paraguay in 1913, but it was possibly imported [4]. A confirmed autochtonous case was first reported in 1945 [5]. 90% of VL cases originate in central departments and in the capital (Asunción), where the urban transmission is a fact of high concern. There is a high proportion of dogs with canine VL in the capital [6] and a high vector density, uncontrolled urbanization and population growth are associated with the rise in case numbers. Adjacent areas in Argentina and Brazil share the epidemic progression of zoonotic VL. More than 66% of the cases occurred in males; the case fatality rate has been reduced from 16% to 5-7%.

**PARASITOLOGICAL INFORMATION**

| ***Leishmania***  **species** | **Clinical form** | **Vector species** | **Reservoirs** |
| --- | --- | --- | --- |
| *L. braziliensis* | ZCL, MCL | *Lu. whitmani,,*  *Lu. migoni,*  *Lu. intermedia* | Unknown |
| *L. infantum* | ZVL | *Lu. longipalpis* | *Canis familiaris* |

**MAPS AND TRENDS**

**Visceral leishmaniasis**


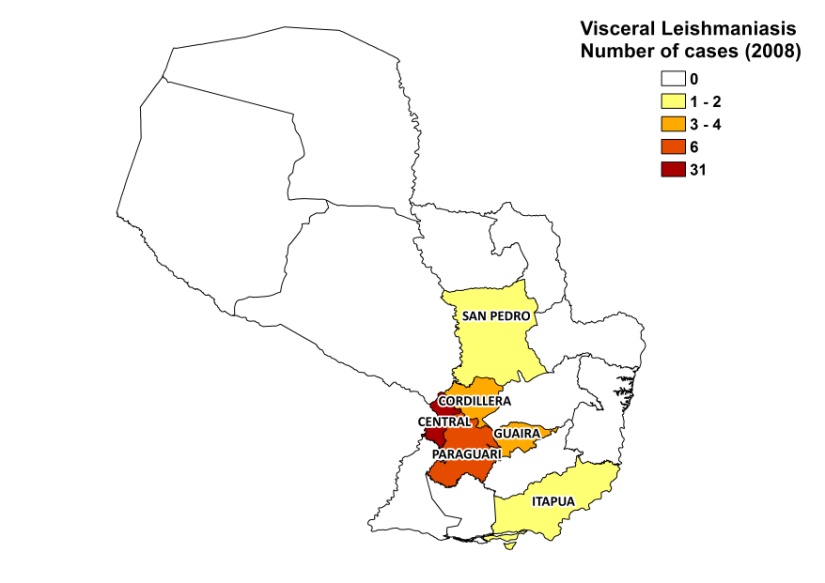

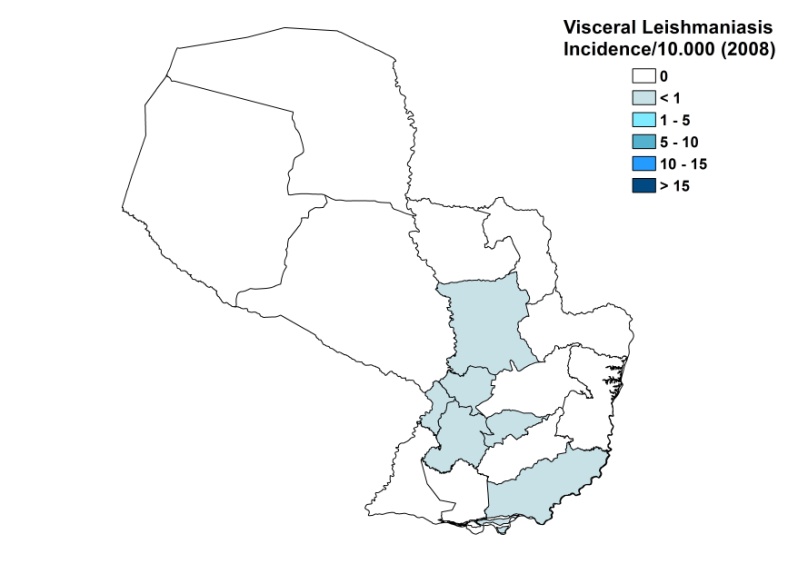


**Cutaneous leishmaniasis**

**
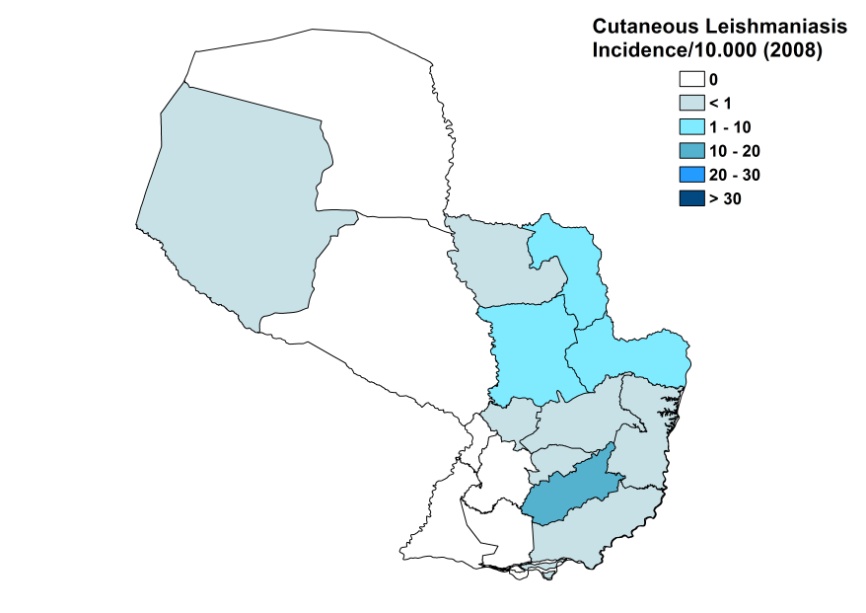
**

**
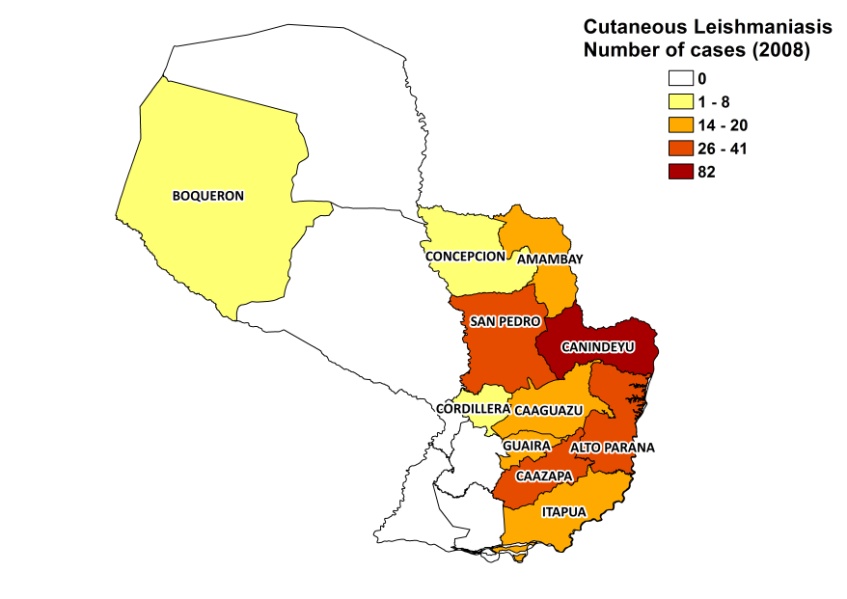
**

**Mucocutaneous leishmaniasis**

**
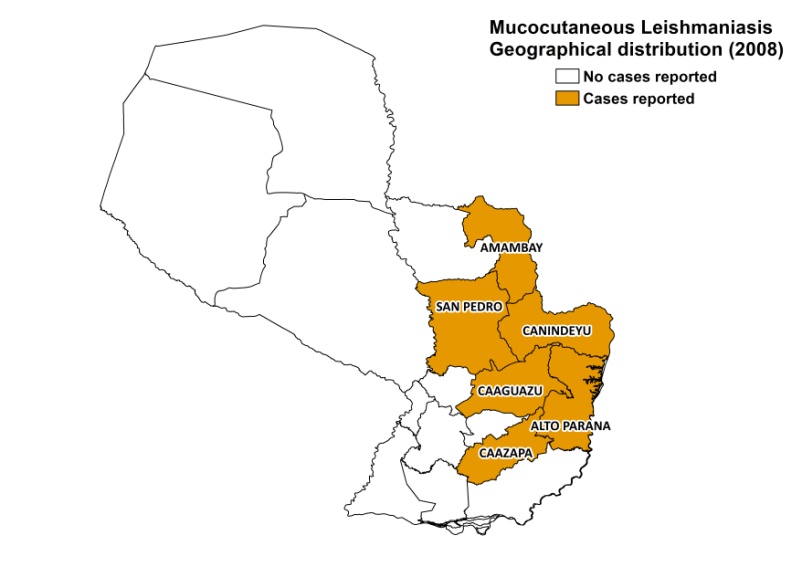
**

**Visceral leishmaniasis trend**

**Cutaneous leishmaniasis trend**

**Mucocutaneous leishmaniasis trend**

| **2008** | **2007** | **2006** |
| --- | --- | --- |
| 173 | 167 | 199 |

**CONTROL**

Notification of leishmaniasis is mandatory and a national leishmaniasis control program has been in place since 1990. There is a vector control program, with regular spraying of insecticides. There is no bednet distribution. There is no formal reservoir control program, but dog serological surveys are performed and positive dogs are sacrificed.

**DIAGNOSIS, TREATMENT**

**Diagnosis:**

CL: on clinical grounds. The Montenegro skin test is used for confirmation at PHC level. In specialized hospitals, PCR is possible.

VL: on clinical grounds in district hospitals. In specialized hospitals, by microscopic examination of bone marrow aspirate or rK39 antigen-based dipstick.

**Treatment:**

CL: antimonials, 20 mg Sb^v^/kg/day for 20 days. Cure rate is 97%, with 3% recurring lesions. In 30% of cases, there is progression into MCL. Mortality rate is 0.5%. Second line treatment is with amphotericin B, 0.5 mg/kg/day for 20 days (cure rate is 100%) Third line treatment is with miltefosine, 2.5 mg/kg/dag for 42 days (cure rate is 75%).

VL: antimonials, 20 mg Sb^v^/kg/day for 30 days. Cure rate is 89%, with 1% relapse and a 8.5% mortality rate. Second line is amphotericin B, 0.5 mg/kg/day for 20 days.

**ACCESS TO CARE**

Treatment is provided for free. VL can only be diagnosed and treated at district hospital level, but CL is usually dealt with at PHC level. The government purchased conventional amphotericin B and meglumine antimoniate (Glucantime, Sanofi) for use in public health clinics in 2007 and 2008. Miltefosine was not provided. In 2008, there was a small donation of liposomal amphotericin B by the Brazilian government. Diagnosis by the Montenegro skin test and serology is free of charge, but for parasitological confirmation the patient has to pay USD 30-50. The private sector is little used, an estimated 3% for VL and 0.4-1% for CL. All patients are thought to have access to care.

**ACCESS TO DRUGS**

In Paraguay, there is no formal essential drug list. Miltefosine (Paladin, Canada), Glucantime (Sanofi) and Pentostam (GSK) are registered, Miltefosine is sold in regular pharmacies, at 8.65 USD per 50 mg tablet, leading to a treatment cost of nearly 500 USD for an adult patient.

**SOURCES OF INFORMATION**

- Dr Juan Domingo Maciel, SENEPA, Ministry of Health. *Leishmaniasis en la Región de las Américas. Reunión de coordinadores de Programa Nacional de Leishmaniasis. OPS/OMS. Medellín, Colombia. 4-6 junio 2008.*
- Dr Martha Rossana Torales Ruotti, National Program for Leishmaniasis Control, SENEPA, Ministry of Health. Head of the Leishmaniasis Program.

1. Migone LE (1913). La buba du Paraguay, leishmaniose ameri-caine. Bull Soc Pathol Exot 6: 210-218.

2. González G, Oliveira, Silva M (1939). La leishmaniosis forestal americana en la guerra del Chaco. Novena Reunión Sociedad Argentina de Patología Regional 2: 959-974.

3. Arias JR, Beltrán F, Desjeux P, Walton B (1996). Epidemiología y Control de la Leishmaniasis en las Américas, por País o Territorio, Cuaderno Técnico OPS Nº 44, Organización Panamericana de la Salud, Washington.

4. Migone LE (1913). Un cas de kala-azar a Asunción, Paraguay. Bull Soc Pathol Exot 6: 118-120.

5. Boggino J, Maas C (1945). Primer caso autóctono de leishmaniosis visceral. An Fac Clin Med (Asunción) 5: 319-326.

6. Canese A (2000). Leishmaniosis visceral canina en el área metropolitana de la "Gran Asunción", Paraguay. Medicina (Buenos Aires) 60 (Supl. 3): 65.
