# Supplementary material for: Leishmaniasis Worldwide and Global Estimates of Its Incidence
Source: PLoS One. 2012 May 31;7(5):e35671. doi: 10.1371/journal.pone.0035671 (PMC3365071; doi:10.1371/journal.pone.0035671)
Supplement: Text S73 — Leishmaniasis Country Profiles, Peru. (DOCX) [file pone.0035671.s073.docx]

**PERU**

**
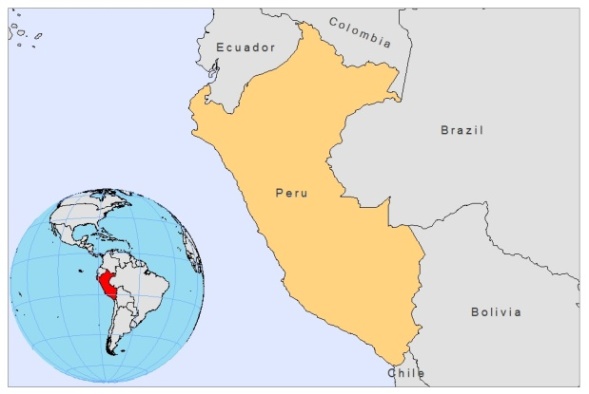
**

**BASIC COUNTRY DATA**

Total Population: 29,076,512

Population 0-14 years: 30%

Rural population: 28%

Population living under USD 1.25 a day: 5.9%

Population living under the national poverty line: 31.3%

Income status: Upper middle income economy

Ranking: High human development (ranking 80)

Per capita total expenditure on health at average exchange rate (US dollar): 201

Life expectancy at birth (years): 74

Healthy life expectancy at birth (years): 61

**BACKGROUND INFORMATION**

Leishmaniasis has been an important public health problem in Peru and transmission takes place in around 74% of the country surface [1]. There are two forms of CL in Peru, mainly defined by geographical and clinical characteristics: Andean leishmaniasis (‘uta’) and sylvatic leishmaniasis (‘espundia’). Uta occurs on the western slopes of the Andean and inter-Andean valleys, between 900 and 3000 m and is caused by *L.peruviana*. People are mainly engaged in agricultural activities, with a high risk of becoming infected when sleeping in shelters in crop areas, where the vector is abundant (*Lu. peruensis, Lu. verrucarum*) [2]. Dogs have been claimed to be the reservoir [3], with 8-45% infected in some areas (Huanuco) [4]. Other wild mammals have also been found infected, but in a much smaller proportion. Most uta cases are in children and more than 80% of the adult population exhibits scars.

Espundia is caused mostly by *L.braziliensis* in the primary tropical forest, where close contact between humans and the sylvatic vector occurs. The risk of infection is associated with occupational activities in the forest [1]. Since 2000, the entire forest area has become an area of high leishmaniasis morbidity. The number of cases has increased rapidly because of the new settlement areas in the lowlands. MCL is common and represents 5% of all CL cases. It is caused by *L. braziliensis* and mostly affects young male adults. 98% of MCL cases were found to occur in the tropical forests [1]. In 2007 alone, almost 12,000 CL cases were reported in Peru, 1,500 of which were MCL.

Apart from *L braziliensis*, other species such as *L. amazonensis, L guyanensis* and *L. lainsoni* are also present, in a lesser degree, in the rainforest, with peculiar distributions [1]. DCL, probably due to *L. amazonensis,* was reported in northwestern Peru.

VL is unknown. There are very few reported cases of HIV/CL co-infection (estimated 0.0001%).

**CONTROL**

A national leishmaniasis control program for CL has been in place since 1985, and notification of leishmaniasis is mandatory. Case detection is passive. There is no vector or reservoir control program, but insecticide spraying is done in places where many cases are reported and there is bednet distribution.

**PARASITOLOGICAL INFORMATION**

| ***Leishmania***  **species** | **Clinical form** | **Vector species** | **Reservoirs** |
| --- | --- | --- | --- |
| *L. peruviana* | ZCL, MCL | *Lu. peruensis,*  *Lu. verrucarum*  *Lu. ayacuchensis* | *Canis familiaris, Didelphis albiventris,* *Phyllotis andinum*, *Akodon* sp. |
| *L. lainsoni* | ZCL | *Lu. ubiquitalis* | unknown |
| *L.amazonensis* | ZCL | unknown | unknown |
| *L. guyanensis* | ZCL MCL | unknown | unknown |
| *L. braziliensis* | ZCL MCL  DCL | *Lu. tejadai, Lu. pescei* | unknown |

**MAPS AND TRENDS**

**Cutaneous leishmaniasis**

**
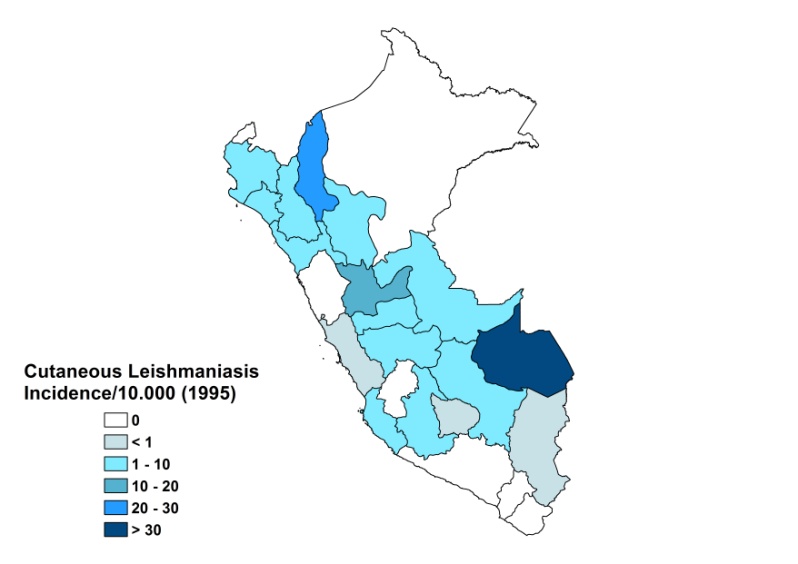

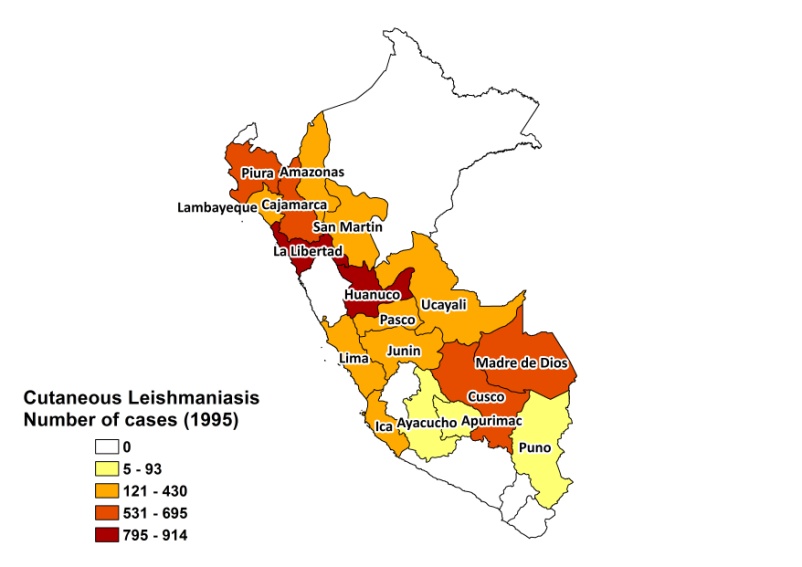
**

**Mucocutaneous leishmaniasis**

**Mucotaneous leishmaniasis**

**
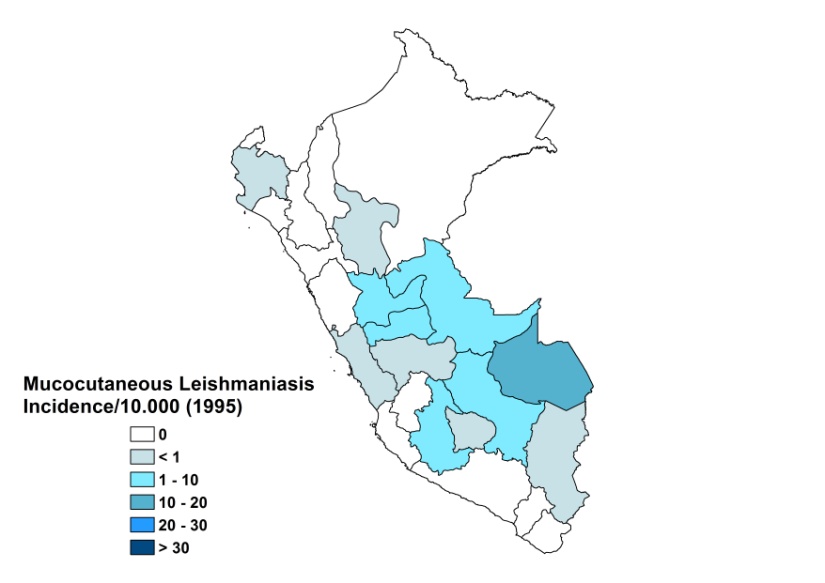

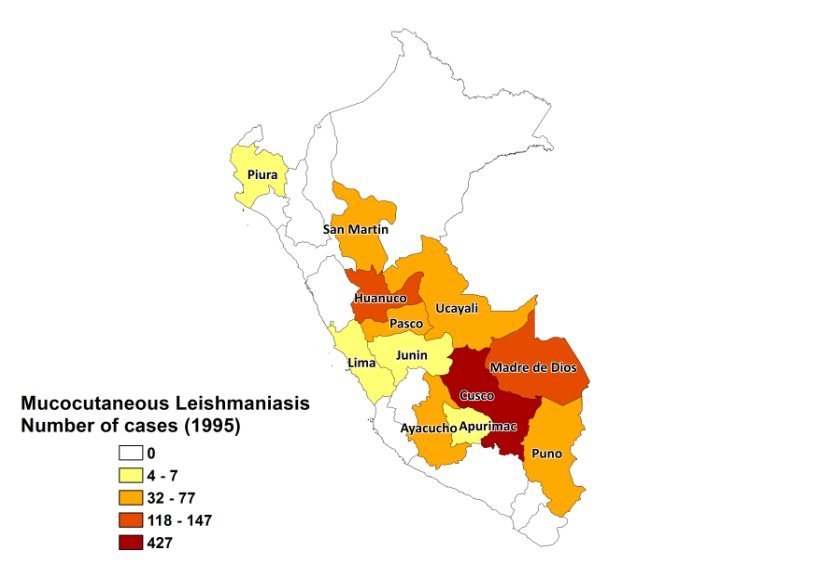
**

**Cutaneous leishmaniasis trend**

**Mucocutaneous leishmaniasis trend**

**DIAGNOSIS, TREATMENT**

**Diagnosis**

CL: on clinical grounds, confirmation by microscopic examination of skin lesion sample.

**Treatment**

CL: antimonials 20 mg Sb^v^/kg/day. Cure rate is 100%. Second line treatment is with Amphotericin B, 1 mg/kg/day until a total dose of 3 g is reached.

**ACCESS TO CARE**

Care of leishmaniasis is provided for free. The government purchased generic SSG from India and China and treatment and diagnosis is provided at outreach level. An unknown percentage of people uses the private sector for treatment of CL. Access to treatment is poor. There is a lack of awareness of the serious nature of the disease and patients often first seek care from traditional healers. Patients live in very remote areas with no health facilities and no transport and suffer major economic loss when they spend time away from home. Often, there is also a shortage of drugs at health facilities.

**DRUG ACCESS**

Sodium stibogluconate and amphotericin B are included in the National Essential Drug List. Meglumine antimoniate (Glucantime, Sanofi) is sold in private pharmacies. One vial of Glucantime costs 7 USD, leading to a treatment cost of 77 USD for intralesional treatment.

**SOURCES OF INFORMATION**

- Dr Yeni Otilia Herrera Hurtado. Ministerio de Salud, Perú.
- Dr. Abelardo Tejada Valencia. Instituto de Medicina Tropical “Daniel A. Carrión”. Universidad Nacional Mayor de San Marcos, Lima. *Leishmaniasis en la Región de las Américas. Reunión de coordinadores de Programa Nacional de Leishmaniasis. OPS/OMS. Medellín, Colombia. 4-6 junio 2008.*

1. Lucas CM, Franke ED, Cachay MI, Tejada A, Cruz ME, et al (1998). [Geographic distribution and clinical description of leishmaniasis cases in Peru.](http://www.ncbi.nlm.nih.gov/pubmed/9715953) Am J Trop Med Hyg 59(2):312-7.

2. Davies CR, Llanos-Cuentas EA, Campos P, Monge J, Villaseca P et al (1997). [Cutaneous leishmaniasis in the Peruvian Andes: risk factors identified from a village cohort study.](http://www.ncbi.nlm.nih.gov/pubmed/9063368) Am J Trop Med Hyg 56(1):85-95.

3. Llanos-Cuentas EA, Roncal N, Villaseca P, Paz L, Ogusuku E et al (1999). [Natural infections of Leishmania peruviana in animals in the Peruvian Andes.](http://www.ncbi.nlm.nih.gov/pubmed/10492779) Trans R Soc Trop Med Hyg 93(1):15-20.

4. Reithinger R, Canales Espinoza J, Llanos-Cuentas A, Davies CR (2003). [Domestic dog ownership: a risk factor for human infection with Leishmania (Viannia) species.](http://www.ncbi.nlm.nih.gov/pubmed/14584365) Trans R Soc Trop Med Hyg 97(2):141-5.
