# Supplementary material for: Leishmaniasis Worldwide and Global Estimates of Its Incidence
Source: PLoS One. 2012 May 31;7(5):e35671. doi: 10.1371/journal.pone.0035671 (PMC3365071; doi:10.1371/journal.pone.0035671)
Supplement: Text S74 — Leishmaniasis Country Profiles, Portugal. (DOCX) [file pone.0035671.s074.docx]

**PORTUGAL**


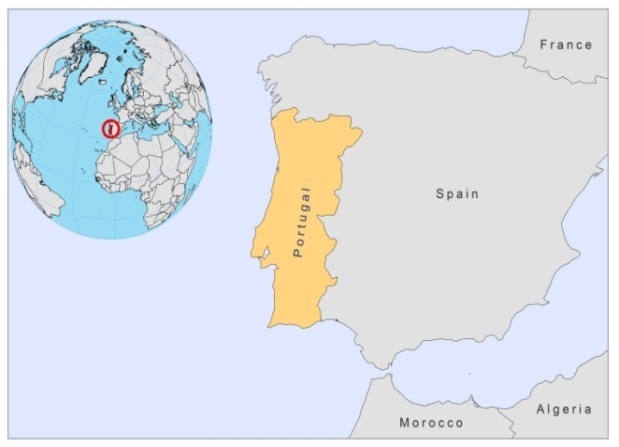


**BASIC COUNTRY DATA**

Total Population: 10,642,841

Population 0-14 years: 15%

Rural population: 39%

Population living under USD 1.25 a day: no data

Population living under the national poverty line: no data

Income status: High income economy: OECD

Ranking: Very high human development (ranking 41)

Per capita total expenditure on health at average exchange rate (US dollar): 2,409

Life expectancy at birth (years): 79

Healthy life expectancy at birth (years): 69

**BACKGROUND INFORMATION**

*L infantum* causes VL, and more rarely CL, in Portugal [1,2]. The incidence of VL was high until 1951, then a decline occurred because of the anti-malarial spraying campaign and the lowest prevalence was reached in 1970; the incidence increasing again after that. Although sporadic cases occur in the whole country, three main endemic foci are recognized, from north to the south: Alto Douro region has the highest incidence with 8.3 human cases/100,000 and 18.7% seroprevalence in dogs [3]; the metropolitan Lisbon region, where most of the HIV/*Leishmania* co-infection cases concentrate (0.2/100,000 inhabitants) [4]; and the Algarve, south Portugal, with 1.2 cases/100,000 inhabitants in the 1980s [5], but with no recent cases among immunocompetent subjects. Before 1985, most of the cases were in children, but between 1996 and 2010, the majority were HIV infected adults (198 of 272). Coinfected patients are found through passive case detection only. The HIV/*Leishmania* co-infection rate has not decreased since the introduction of HAART in 1997, as opposed to all other western European countries [6]. This might be due to the fact that drug users run the highest risk of coinfection, a group which is difficult to target for active case finding and follow-up.

The dog is the main reservoir, with a seroprevalence rate increasing in some areas from 5.5% in the 1980s to 18% according to a survey held in 2002 [7,8]. The nation-wide average seroprevalence is around 6% as determined in a screening in 2009. Around 0.5% of the sandflies have been found infected by *Leishmania* [9]. A survey among 138 cats the year before showed an infection rate of 20.3% in some areas [10].

Underreporting is common. In 1996, and since then almost every year, the number of yearly VL cases reported to the MoH was lower than the number of cases diagnosed at Instituto de Medicina Tropical, Lisbon. CL has been reported very infrequently [2] (only 24 cases between 1996 and the first quarter of 2009).

**PARASITOLOGICAL INFORMATION**

| ***Leishmania* species** | **Clinical form** | **Vector species** | **Reservoirs** |
| --- | --- | --- | --- |
| *L. infantum* | ZVL, CL | *P. perniciosus, P. ariasi* | *Canis familiaris, Vulpes vulpes* |

**MAPS AND TRENDS**

**Visceral leishmaniasis**

**
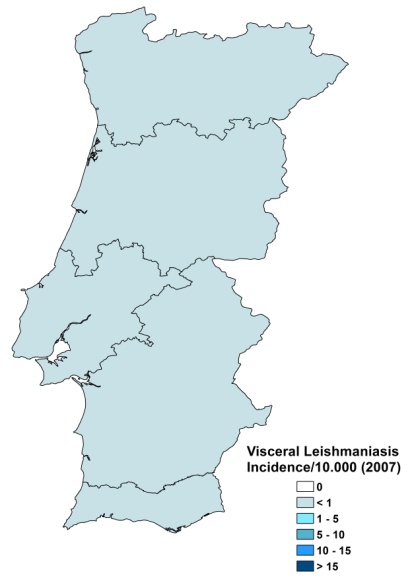

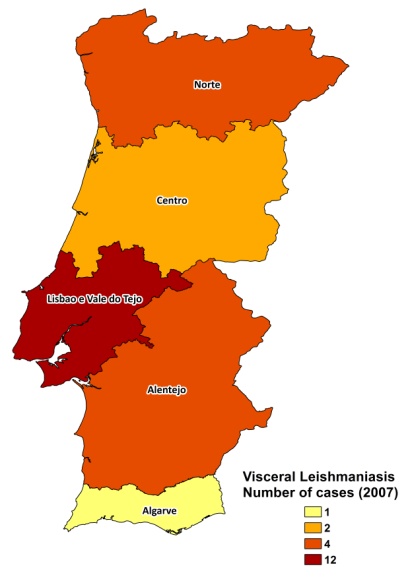
**

**Visceral leishmaniasis trend**

**CONTROL**

The notification of VL is mandatory in the country and there is a national leishmaniasis control program for VL. There is no leishmaniasis vector control program and insecticide spraying is not regularly done. There is a leishmaniasis reservoir control program: obligatory diagnosis and treatment, or sacrifice, of all suspected dogs during the anti-rabies vaccination campaign.

**DIAGNOSIS, TREATMENT**

**Diagnosis**

VL: various serological, parasitological and molecular techniques are available.

**Treatment**

VL: antimonials, 20 mg Sb^v^/kg/day for 20-28 days or liposomal amphotericin B 3-5 mg/kg/day, for 5-10 days. Cure rate for both drugs is >95% (except in coinfected patients). Second line treatment is with miltefosine.

**ACCESS TO CARE**

Care for leishmaniasis is provided for free. Diagnosis and treatment for VL and CL is only possible in secondary and tertiary hospitals. Diagnosis is sometimes done by private laboratories. All patients are thought to have access to care.

**ACCESS TO DRUGS**

Meglumine antimoniate, liposomal amphotericin B and conventional amphotericin B are included in the National Essential Drug List for VL and CL. Drugs for leishmaniasis are not sold in private pharmacies. Liposomal amphotericin B (AmBisome, Gilead) is registered, but no antimonials are registered in Portugal. Miltefosine is only registered for treatment of dogs.

**SOURCES OF INFORMATION**

- Direcção Geral de Saúde (www.dgs.pt).
- Direcção Geral de Veterinária” (<http://ww-w.dgv.min-agricultura.pt/>).
- Dr Lenea Campino, Instituto de Higiene e Medicina Tropical, Lisboa. *Leishmaniasis in the European Region, a WHO consultative intercountry meeting, Istanbul, Turkey, 17–19 November 2009.*

1. Campino L, Maia C (2010). [Epidemiology of leishmaniases in Portugal.](http://www.ncbi.nlm.nih.gov/pubmed/21144327) Acta Med Port 23(5):859-64.

2. Campino L, Abranches P (2002). Leishmaniose cutânea. Uma doença rara em Portugal? Acta Med Port 15:387-390.

3. Cardoso L, Rodrigues M, Santos H, Schoone GJ, Carreta P et al (2004). [Sero-epidemiological study of canine Leishmania spp. infection in the municipality of Alijó (Alto Douro, Portugal).](http://www.ncbi.nlm.nih.gov/pubmed/15110400) Vet Parasitol 121(1-2):21-32.

4. Campino L, Santos-Gomes GM, Pratlong F, Antunes F, Maurício I, et al (1997). [HIV/Leishmania co-infections in Portugal: diagnosis and isoenzyme characterization of Leishmania.](http://www.ncbi.nlm.nih.gov/pubmed/9290851) Ann Trop Med Parasitol 91(4):433-6.

5. Vicente A. (1990) Kala-azar infantil. Dados epidemiológicos e incidência do kala-azar no Internamento de Pediatria do Hospital de Faro. Rev Port Doencas Infec 13:173-182.

6. Alvar J, Aparicio P, Aseffa A, Den Boer M, Cañavate C et al (2008). [The relationship between leishmaniasis and AIDS: the second 10 years.](http://www.ncbi.nlm.nih.gov/pubmed/18400800) Clin Microbiol Rev 21(2):334-59.

7. Abranches P, Lopes FJC, Conceição-Silva FM, Ribeiro MMS, Pires CA (1983). Kala-azar in Portugal. III. Results of a survey on canine leishmaniasis performed in the Lisbon region. Comparison of urban and rural zones [in French] Ann Parasitol Hum Comp 58:307–15.

8. Cortes S, Afonso MO, Alves-Pires C, Campino L. [Stray dogs and leishmaniasis in urban areas, Portugal.](http://www.ncbi.nlm.nih.gov/pubmed/18252134) Emerg Infect Dis 13(9):1431-2.

9. Maia C, Afonso MO, Neto L, Dionísio L, Campino L (2009). [Molecular detection of Leishmania infantum in naturally infected Phlebotomus perniciosus from Algarve region, Portugal.](http://www.ncbi.nlm.nih.gov/pubmed/19959852) Vector Borne Dis 46(4):268-72.

10. Maia C, Gomes J, Cristóvão J, Nunes M, Martins A et al (2010). [Feline Leishmania infection in a canine leishmaniasis endemic region, Portugal.](http://www.ncbi.nlm.nih.gov/pubmed/20869810) Vet Parasitol 174(3-4):336-40.
