# Supplementary material for: Leishmaniasis Worldwide and Global Estimates of Its Incidence
Source: PLoS One. 2012 May 31;7(5):e35671. doi: 10.1371/journal.pone.0035671 (PMC3365071; doi:10.1371/journal.pone.0035671)
Supplement: Text S75 — Leishmaniasis Country Profiles, Romania. (DOCX) [file pone.0035671.s075.docx]

**ROMANIA**


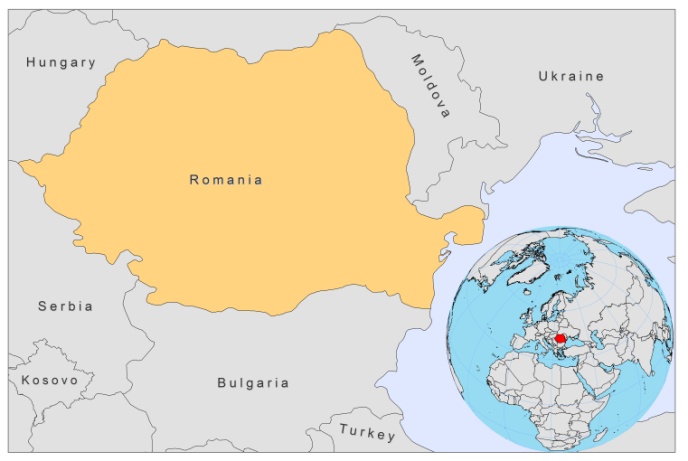


**BASIC COUNTRY DATA**

Total Population: 21,442,012

Population 0-14 years: 15%

Rural population: 45%

Population living under USD 1.25 a day: 0.5%

Population living under the national poverty line: 13.8%

Income status: Upper middle income economy

Ranking: High human development (ranking 50)

Per capita total expenditure on health at average exchange rate (US dollar): 408

Life expectancy at birth (years): 73

Healthy life expectancy at birth (years): 63

**BACKGROUND INFORMATION**

The first case of VL was reported in 1912 [1]. In 1954, an outbreak of VL was described in Oltenia region [2]. No further cases were documented until 1989. However, since increasing numbers of Romanians have become seasonal migrant workers in southern Mediterranean countries, imported VL is now seen in Romania. Six cases of VL were imported, between 1999 and 2006, by returning male migrant workers that acquired VL in Italy, Greece and Spain [3]. In 2007, another 5 imported cases from Italy and Greece were documented and in 2010, a case of VL was documented in a woman that had spent a long time in Greece.

**PARASITOLOGICAL INFORMATION**

| ***Leishmania* species** | **Clinical form** | **Vector species** | **Reservoirs** |
| --- | --- | --- | --- |
| *L. infantum* | ZVL | *P. neglectus, P. perfiliewi* | *Canis familiaris* |

**MAPS AND TRENDS**

No information available.

**CONTROL**

The notification of leishmaniasis is not mandatory in the country and there is no national leishmaniasis control program. There is no leishmaniasis vector control program and no leishmaniasis reservoir control program.

**DIAGNOSIS, TREATMENT**

**Diagnosis**

VL: confirmation by microscopic examination of bone marrow.

**Treatment**

VL: amphotericin B or pentamidine.

**ACCESS TO CARE**

VL is little known in Romania. The period between clinical onset and diagnosis of VL in imported cases varied between 2 and 14 months.

**ACCESS TO DRUGS**

No antimonials are registered.

**SOURCES OF INFORMATION**

1. Gaman A, Dobrea C, Gaman G (2010). A case of visceral leishmaniasis in Oltenia región (Romania). Romanian Journal of Morphology and Embryology 51(2):391–394.

2. Desjeux P (1991) Information on the epidemiology and control of the leishmaniases by country or territory. World Health Organization. WHO/LEISH/91.30.

3. Neghina R, Neghina AM, Merkler C, Marincu I, Moldovan R et al (2009). Importation of visceral leishmaniasis in returning Romanian workers from Spain. Travel Med Infect Dis 7(1):35-9.
