# Supplementary material for: Leishmaniasis Worldwide and Global Estimates of Its Incidence
Source: PLoS One. 2012 May 31;7(5):e35671. doi: 10.1371/journal.pone.0035671 (PMC3365071; doi:10.1371/journal.pone.0035671)
Supplement: Text S76 — Leishmaniasis Country Profiles, Saudi Arabia. (DOCX) [file pone.0035671.s076.docx]

**SAUDI ARABIA**


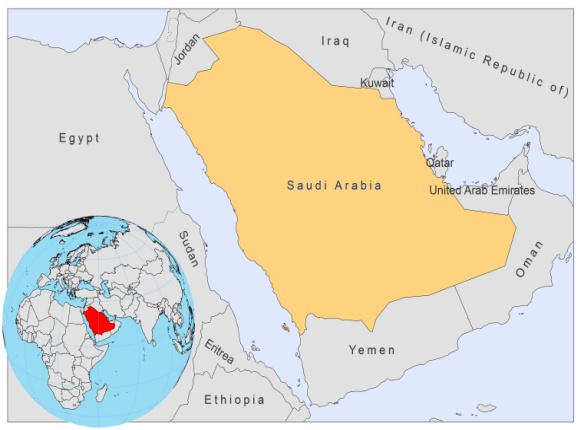


**BASIC COUNTRY DATA**

Total Population: 27,448,086

Population 0-14 years: 30%

Rural population: 16%

Population living under USD 1.25 a day: no data

Population living under the national poverty line: no data

Income status: High income economy

Ranking: High human development (ranking 56)

Per capita total expenditure on health at average exchange rate (US dollar): 714

Life expectancy at birth (years): 74

Healthy life expectancy at birth (years): 61

**BACKGROUND INFORMATION**

VL is sporadic and mostly seen in Jazan region. In 2008, 29 of 32 cases reported occurred here. 69% of cases were among young, male children. VL may be underreported to a small degree.

CL is more common, and mainly reported in adult, male patients (75%). 45% of cases are among foreigners, and most cases occur between 15 and 44 years of age. In 2008, the majority of cases were reported in Al-Qasim. CL is known to be underreported.

CL caused by *L.major* has been known in several areas of the country, but was considered a minor problem until 1975. Several factors produced an increase of transmission: rapid urbanization, migration, intensive agriculture, poor living conditions at farms, and massive immigration. Consequently, leishmaniasis became a public health problem, with over 18,000 cases in 1983. The case load reduced rapidly after a National Control Program was set up.

CL caused by *L.tropica* is endemic in the southwestern part of the country. Cases of leishmaniasis recidivans are not uncommon in the area.

There are no cases of HIV/*Leishmania* co-infection.

**PARASITOLOGICAL INFORMATION**

| ***Leishmania* species** | **Clinical form** | **Vector species** | **Reservoirs** |
| --- | --- | --- | --- |
| *L. tropica* | CL | *P. sergenti* |  |
| *L. infantum* | ZVL | unknown | *Canis familiaris* |
| *L. major* | ZCL | *P. papatasi* | *Meriones spp., Psammomys obesus* |
| *L. donovani* | VL | unknown |  |

**MAPS AND TRENDS**

**
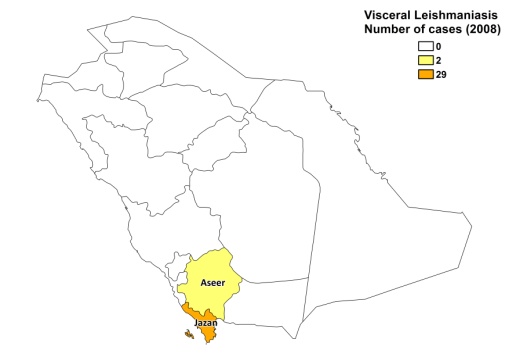
Visceral leishmaniasis**

**
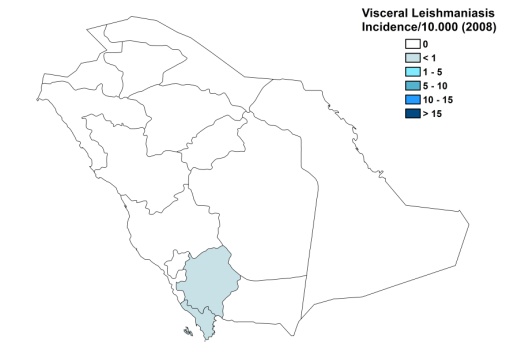
**

**Cutaneous leishmaniasis**

**
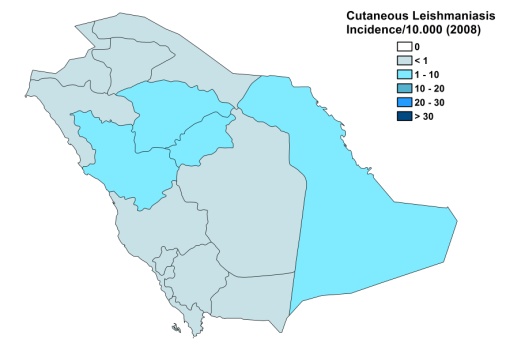
**

**
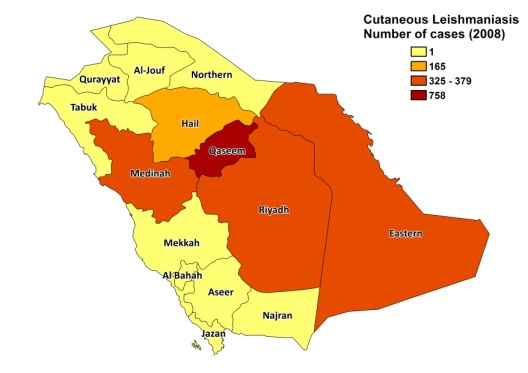
**

**Visceral leishmaniasis trend**

**Cutaneous leishmaniasis trend**

**CONTROL**

The notification of leishmaniasis is mandatory in the country and a national control program for VL and CL has been in place for over 20 years. Active human case detection is performed in foci where one or more cases were found. There is a vector control program, with regular insecticide spraying. There is also a reservoir control program; serological surveys of dogs are occasionally performed. Positive dogs are not sacrificed. Limited rodent control is performed.

**DIAGNOSIS, TREATMENT**

**Diagnosis**

VL: confirmation by microscopic examination of bone marrow or spleen aspirate.

CL: on clinical grounds, confirmation with microscopic examination of skin lesion sample.

**Treatment**

VL: antimonials, 10-20 mg Sb^v^/kg/day. Cure rate is 98%. Second line treatment is with amphotericin B.

CL: antimonials, intralesional or systemic (10-20 mg Sb^v^/kg/day). Cure rate is 95% (5% loss to follow up).

**ACCESS TO CARE**

Medical care is provided for free, which includes care for leishmaniasis, but health workers occasionally charge informal payments to patients. VL is only diagnosed and treated in secondary and specialized hospitals. CL is diagnosed and treated at health center level. About 5% of people seek private care for CL (those that can afford private hospitals).

All patients are thought to have access to care and the Ministry of Health provided sufficient antimonials (Pentostam, GSK) to treat all patients in 2007 and 2008. However, underreporting for CL occurs and patients often use traditional healing practices before seeking regular care.

**ACCESS TO DRUGS**

Antimonials (for VL and CL) and amphotericin B (for VL) are included in the National Essential Drug List for leishmaniasis. Sodium stibogluconate (Pentostam, GSK) is registered in Saudi Arabia. Drugs for leishmaniasis are not available at private pharmacies or drug markets.

**SOURCES OF INFORMATION**

- Dr Suleiman Mohammed Al Seghayer, *Leishmania* department, Ministry of Health.
