# Supplementary material for: Leishmaniasis Worldwide and Global Estimates of Its Incidence
Source: PLoS One. 2012 May 31;7(5):e35671. doi: 10.1371/journal.pone.0035671 (PMC3365071; doi:10.1371/journal.pone.0035671)
Supplement: Text S77 — Leishmaniasis Country Profiles, Senegal. (DOCX) [file pone.0035671.s077.docx]

**SENEGAL**


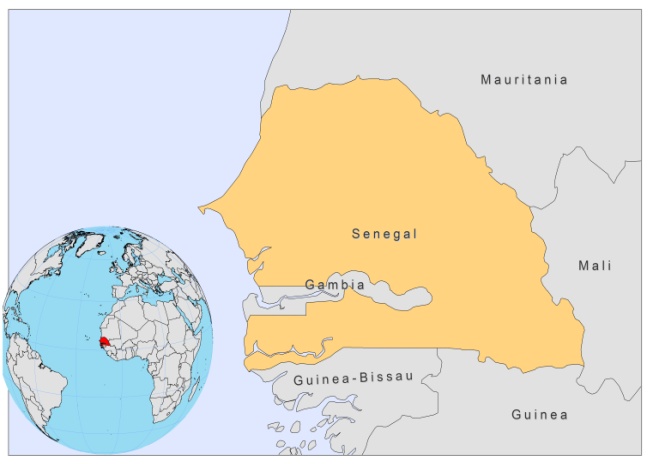


**BASIC COUNTRY DATA**

Total Population: 12,433,728

Population 0-14 years: 44%

Rural population: 57%

Population living under USD 1.25 a day: 33.5%

Population living under the national poverty line: no data

Income status: Lower middle income economy

Ranking: Low human development (ranking 155)

Per capita total expenditure on health at average exchange rate (US dollar): 59

Life expectancy at birth (years): 59

Healthy life expectancy at birth (years): 48

**BACKGROUND INFORMATION**

Human VL has not been reported but canine VL is well known in Senegal since 1910. VL is widespread and very common in domestic and stray dogs, mainly in western Senegal, where it is enzootic. *L. infantum* was recently typed from dogs in the Mont Rolland District (Thies area), where the seroprevalence of canine leishmaniasis was up to 40% [1]. A serological survey among the human population was carried out and 23% seropositivity (73/315), but no active VL cases, was found. Risk factors associated to infection exposure were: age, the presence of Nebedaye trees (*Moringa oleifera*) and infected dogs in the household [2].

Zoonotic cutaneous leishmaniasis due to *L. major* has been known since 1933 [3]. The disease is endemic throughout Senegal. Between 1976 and 1980, an intensive study, carried out during an outbreak in a localized focus (Keur Moussa Monastery) between the cities of Dakar and Thies, revealed an endemo-epidemic type of evolution with a permanent active focus maintaining the endemicity and, from time to time, an outbreak with a great number of cases [4]. The outbreaks occur when the ecology is modified by a new agricultural project and/or by the introduction into a new non-immune population; the zoonosis itself is related to the fluctuations in rodent reservoir populations [5].

From 1994-2008, 117 CL cases were diagnosed, all in one specialized hospital in Dakar) 44 of which between 2004 and 2008. The sex ratio was 3:1 (male:female). CL is believed to be vastly underreported.

4 cases of HIV/CL co-infection were reported (an estimated 5-10% of patients are coinfected).

**PARASITOLOGICAL INFORMATION**

| ***Leishmania* species** | **Clinical form** | **Vector species** | **Reservoirs** |
| --- | --- | --- | --- |
| *L. major* | ZCL | *P. duboscqi* | *Arvicantis niloticus*, *Tatera gambiana,* *Mastomys erythroleucus* |
| *L. infantum* | ZVL | unknown | *Canis familiaris* |

**MAPS AND TRENDS**

**Cutaneous leishmaniasis**

**
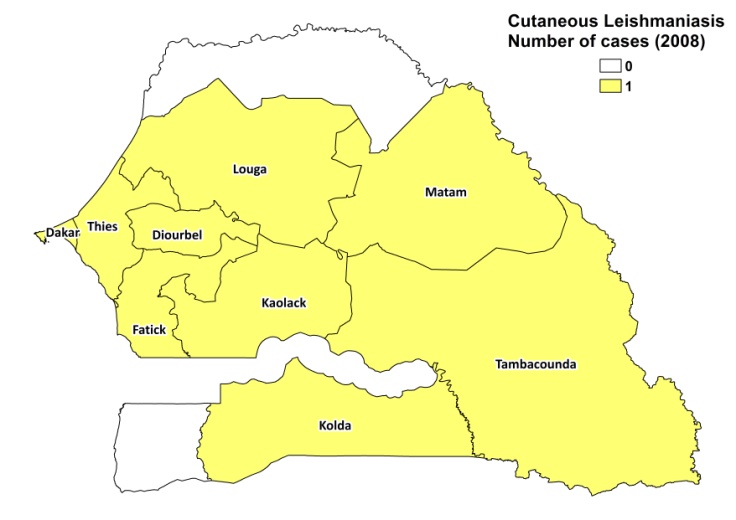

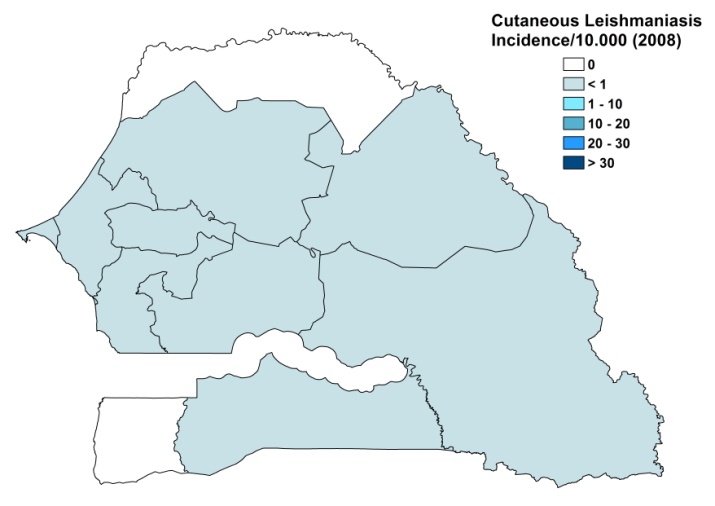
**

**Cutaneous leishmaniasis trend**

**CONTROL**

The notification of leishmaniasis is not mandatory in the country and there is no national leishmaniasis control program. Case detection is passive. There is no leishmaniasis vector control program and no leishmaniasis reservoir control program.

**DIAGNOSIS, TREATMENT**

**Diagnosis**

CL: on clinical grounds, confirmation by microscopic examination of skin lesion sample (20% of cases) or by histological examination (80% of cases).

**Treatment**

CL: antimonials, intralesional or systemic (20 mg Sb^v^/kg/day for 15 days). Cure rate 80-100% (89% in case of systemic treatment). 4 patients with diffuse CL (HIV-coinfected) died during systemic treatment.

**ACCESS TO CARE**

Care for leishmaniasis is not provided for free. Patients need to pay for registration, consultation, tests (200 USD), hospitalization, and for the drugs themselves (240 USD), which amounts to a total fee of over 600 USD. This is unaffordable for most patients, as they are often extremely poor. Access to treatment is very limited. The Ministry of Health does not provide drugs for leishmaniasis and there were no drug donations by other agents. The only place where CL cases can be diagnosed and treated is in one specialized hospital in Dakar. There is a lack of awareness of CL among the public as well as among health workers. The symptoms of CL are not seen as a medical priority.

**ACCESS TO DRUGS**

No drugs for leishmaniasis are included in the National Essential Drug List or registered in Senegal. Meglumine antimoniate (Glucantime) is available in private pharmacies for 60 USD per box of 5 ampoules, leading to a treatment cost of about 360 USD for one 15-day systemic treatment.

**SOURCES OF INFORMATION**

- Dr Mame Thierno Dieng, CHUA Le Dantek Dakar. Consultative Meeting on the Control of Leishmaniasis in the African Region. WHO/AFRO Addis Ababa, 23-25 Feb 2010.

**1. Faye** **B,** **Bañuls AL,**  **Bucheton B,** **Dione M,** **Bassanganam O** **et al (2010).** Canine visceral leishmaniasis caused by *Leishmania infantum* in Senegal: risk of emergence in humans? [Microbes and Infection](http://www.sciencedirect.com/science/journal/12864579) 12 (14-15): 1219-1225.

**2.** Faye B, Bucheton B, Bañuls AL, Senghor MW, Niang AA et al (2011). [Seroprevalence of Leishmania infantum in a rural area of Senegal: analysis of risk factors involved in transmission to humans.](http://www.ncbi.nlm.nih.gov/pubmed/21543098) Trans R Soc Trop Med Hyg 105(6):333-40.

3. Riou M, Advier M (1933). Leishmaniose cutanée cantractée au Sénégal. Bull Soc Path Exot 26:254–256.

4. [Blanchot M](http://www.ncbi.nlm.nih.gov/pubmed?term=%22Blanchot%20M%22%5BAuthor%5D), [Lusina D](http://www.ncbi.nlm.nih.gov/pubmed?term=%22Lusina%20D%22%5BAuthor%5D), [Beunier E](http://www.ncbi.nlm.nih.gov/pubmed?term=%22Beunier%20E%22%5BAuthor%5D) (1984). Interepidemic surveillance of a cutaneous leishmaniasis focus in Senegal. [Med Trop](javascript:AL_get(this,%20'jour',%20'Med%20Trop%20(Mars).');) 44(1):35-40.

5. Desjeux P (1991) Information on the epidemiology and control of the leishmaniases by country or territory. World Health Organization. WHO/LEISH/91.30.
