# Supplementary material for: Leishmaniasis Worldwide and Global Estimates of Its Incidence
Source: PLoS One. 2012 May 31;7(5):e35671. doi: 10.1371/journal.pone.0035671 (PMC3365071; doi:10.1371/journal.pone.0035671)
Supplement: Text S78 — Leishmaniasis Country Profiles, Slovenia. (DOCX) [file pone.0035671.s078.docx]

**SLOVENIA**

**
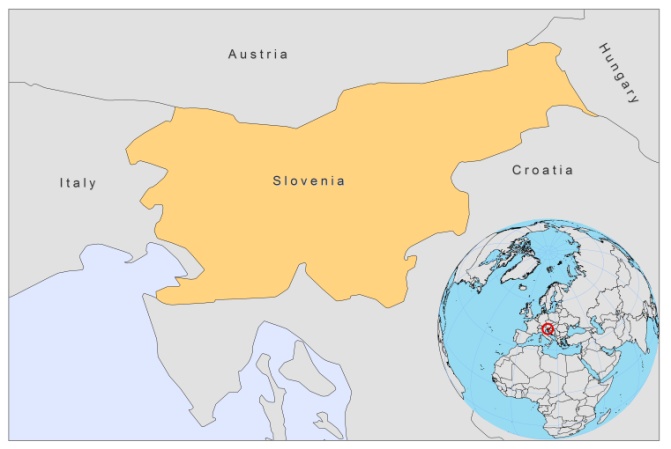
**

**BASIC COUNTRY DATA**

Total Population: 2,052,821

Population 0-14 years: 14%

Rural population: 52%

Population living under USD 1.25 a day: no data

Population living under the national poverty line: no data

Income status: High income economy

Ranking: Very high human development (ranking 21)

Per capita total expenditure on health at average exchange rate (US dollar): 2,175

Life expectancy at birth (years): 79

Healthy life expectancy at birth (years): 69

**BACKGROUND INFORMATION**

Leishmaniasis is rare in Slovenia. In 2010, a case of CL was documented in a 10-year-old boy and was probably imported [1]. In 2000, a case of CL was detected that was imported from Peru [2].

**PARASITOLOGICAL INFORMATION**

| ***Leishmania***  **species** | **Clinical form** | **Vector species** | **Reservoirs** |
| --- | --- | --- | --- |
| *L. infantum* | VL, CL | *P. neglectus* | *Canis familiaris* |

**MAPS & TREND**

No information available.

**CONTROL**

The notification of leishmaniasis is not mandatory in the country and there is no national leishmaniasis control program. There is no leishmaniasis vector control program and no leishmaniasis reservoir control program.

**DIAGNOSIS, TREATMENT, ACCESS TO CARE and ACCESS TO DRUGS**

No information available.

**SOURCES OF INFORMATION**

1. [Marovt M](http://www.ncbi.nlm.nih.gov/pubmed?term=%22Marovt%20M%22%5BAuthor%5D), [Kokol R](http://www.ncbi.nlm.nih.gov/pubmed?term=%22Kokol%20R%22%5BAuthor%5D), [Stanimirović A](http://www.ncbi.nlm.nih.gov/pubmed?term=%22Stanimirovi%C4%87%20A%22%5BAuthor%5D), [Miljković J](http://www.ncbi.nlm.nih.gov/pubmed?term=%22Miljkovi%C4%87%20J%22%5BAuthor%5D) (2010). Cutaneous leishmaniasis: A case report. [Acta Dermatovenerol Alp Panonica Adriat .](javascript:AL_get(this,%20'jour',%20'Acta%20Dermatovenerol%20Alp%20Panonica%20Adriat.');)19(2):41-3.

2. [Brecelj M](http://www.ncbi.nlm.nih.gov/pubmed?term=%22Brecelj%20M%22%5BAuthor%5D), [Pikelj F](http://www.ncbi.nlm.nih.gov/pubmed?term=%22Pikelj%20F%22%5BAuthor%5D), [Gubensek F](http://www.ncbi.nlm.nih.gov/pubmed?term=%22Gubensek%20F%22%5BAuthor%5D), [Anderluh G](http://www.ncbi.nlm.nih.gov/pubmed?term=%22Anderluh%20G%22%5BAuthor%5D) (2000). Polymerase chain reaction as a diagnostic tool for detecting Leishmania. [Infection](javascript:AL_get(this,%20'jour',%20'Infection.');) 28(2):111-3.
