# Supplementary material for: Leishmaniasis Worldwide and Global Estimates of Its Incidence
Source: PLoS One. 2012 May 31;7(5):e35671. doi: 10.1371/journal.pone.0035671 (PMC3365071; doi:10.1371/journal.pone.0035671)
Supplement: Text S79 — Leishmaniasis Country Profiles, Somalia. (DOCX) [file pone.0035671.s079.docx]

**SOMALIA**


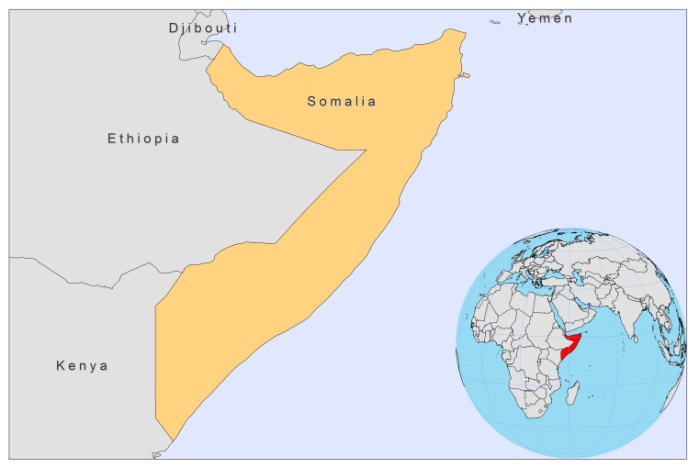


**BASIC COUNTRY DATA**

Total Population: 9,330,872

Population 0-14 years: 45%

Rural population: 63%

Population living under USD 1.25 a day: no data

Population living under the national poverty line: no data

Income status: Low income economy

Ranking: no data

Per capita total expenditure on health at average exchange rate (US dollar): no data

Life expectancy at birth (years): 51

Healthy life expectancy at birth (years): 37

**BACKGROUND INFORMATION**

VL is endemic along the Webe Shebelle and Juba river basins of southern Somalia. The ecological features are similar to those in southern Ethiopia and Kenya. The first cases date back to 1930-40 in Jowhar area, Middle Shabelle Region. In this region, positive skin test results were found in 15% of people in 1967 and 26% in 1995, with a low prevalence of cases during these studies. Cases have been described all over Somalia, but currently occur most frequently in the Bay and Bakool regions, along the border with Ethiopia, and the Gedo region, along the border with Kenya. In 2003, in a refugee camp in Bakool region, positive leishmanin skin test result were found in 15% of inhabitants. The case numbers found were high, while previously there were only few cases. In 2006, an outbreak of 1,000 cases was reported in Hudur, Bakool region, by an NGO (MSF). In 2008, the number of cases found by MSF in this region was nearly as high. However, the case load dropped with 65% when comparing the first 4 months of 2009 to the first 4 months of 2008. In Bay and Gado regions, NGOs have diagnosed and treated some cases in 2008, in other areas, cases may exist, but may never have been detected due to security concerns.

Termite hills and red acacia trees are breeding sites for the vector. Children under 5 years old are mostly affected. In one observation, 126 out of 143 cases were children under 5 (88.1%). PKDL seems rare.

Sporadic imported cases of CL have been reported from the south and a few cases of mucosal and cutaneous leishmaniasis have been reported from northern Somalia.

**PARASITOLOGICAL INFORMATION**

| ***Leishmania* species** | **Clinical form** | **Vector species** | **Reservoirs** |
| --- | --- | --- | --- |
| *L. donovani* | VL, PKDL | *P. martini* | Human |

**MAPS AND TRENDS**

**Visceral leishmaniasis**


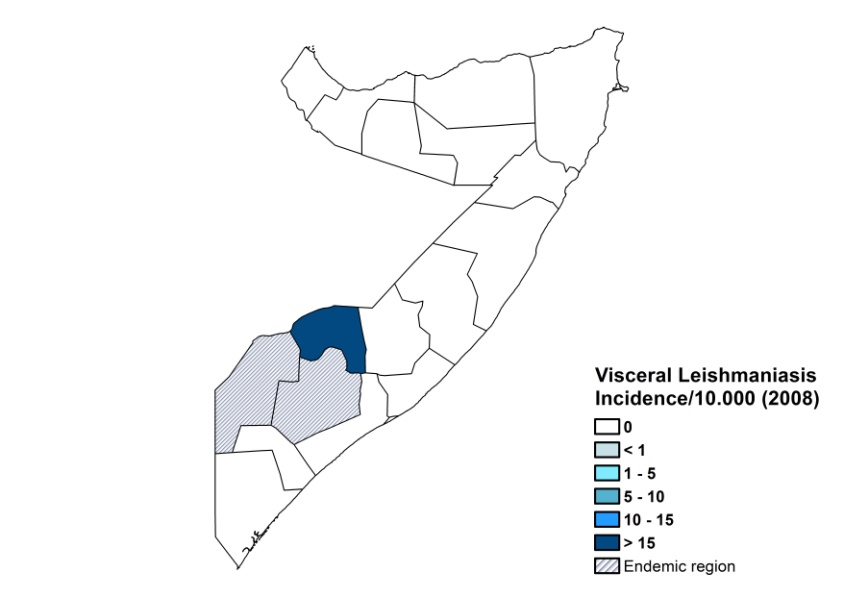

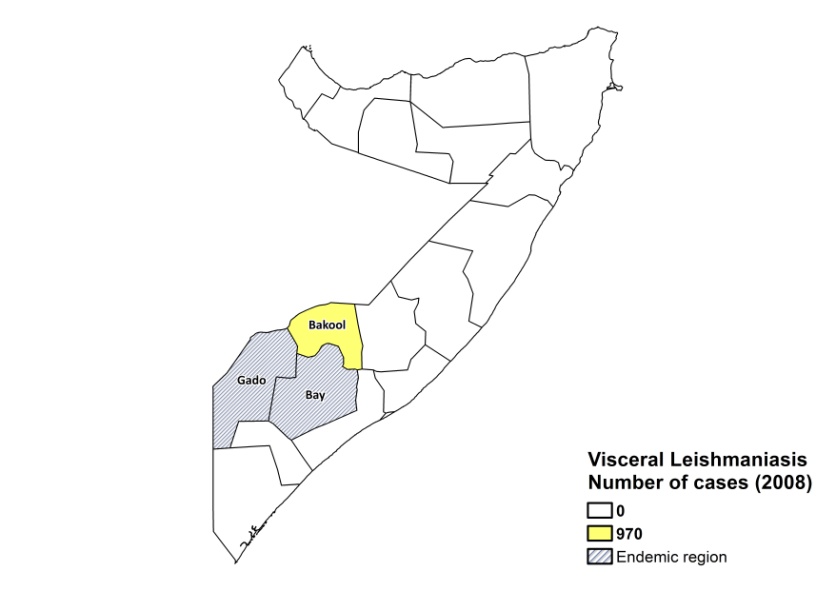


**Visceral leishmaniasis trend**

Data not available.

**CONTROL**

The notification of leishmaniasis is mandatory in the country. There is no national leishmaniasis control program. Active human case detection is not performed. There is no leishmaniasis vector control program and no leishmaniasis reservoir control program. Bednet distribution takes place in the context of malaria control.

**DIAGNOSIS, TREATMENT**

**Diagnosis:**

VL: antigen-based rK39 rapid diagnostic tests or DAT.

**Treatment:**

VL: antimonials, 20 mg Sb^v^/kg/day for 30 days, plus 14 days if test of cure cannot be done. Second line: Amphotericin B, 0.5 mg/kg on alternate days until a total dose of 20 mg/kg is reached. Initial cure rate of 94.8% with antimonials, default rate of 2.3% and fatality rate of 2.9% (MSF data).

**ACCESS TO CARE**

Care for leishmaniasis is provided for free by NGOs. The Ministry of Health does not provide drugs for leishmaniasis; drugs are donated by NGOs or WHO. Several NGOs were present until 2009, treating up to a thousand patients yearly in the endemic regions of Bay and Bakool, but due to insecurity, most of them closed their programs; this means that patients have no access to treatment now. In general, there is a lack of awareness of the disease, and many patients live too far away from the locations where treatment is given. There is insufficient trained staff to treat leishmaniasis and an irregular supply of anti-leishmania drugs and diagnostics. Many patients resort to traditional healing practices.

**ACCESS TO DRUGS**

Sodium stibogluconate is included in the National Essential Drug List. No drugs for leishmaniasis are available in pharmacies and unregulated drug markets. No antimonials are registered in Somalia.

**SOURCES OF INFORMATION**

- Dr Mohamed M Ali , WHO Somalia. *Consultative Meeting on The Control of Leishmaniasis in the African Region. WHO/AFRO Addis Ababa, 23-25 Feb 2010.*
