# Supplementary material for: Leishmaniasis Worldwide and Global Estimates of Its Incidence
Source: PLoS One. 2012 May 31;7(5):e35671. doi: 10.1371/journal.pone.0035671 (PMC3365071; doi:10.1371/journal.pone.0035671)
Supplement: Text S80 — Leishmaniasis Country Profiles, South Africa. (DOCX) [file pone.0035671.s080.docx]

**SOUTH AFRICA**


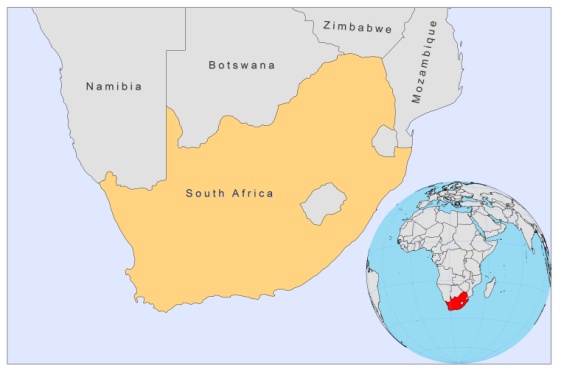


**BASIC COUNTRY DATA**

Total Population: 49,991,300

Population 0-14 years: 30%

Rural population: 38%

Population living under USD 1.25 a day: 17.4%

Population living under the national poverty line: 23%

Income status: Upper middle income economy

Ranking: Medium human development (ranking 123)

Per capita total expenditure on health at average exchange rate (US dollar): 485

Life expectancy at birth (years): 52

Healthy life expectancy at birth (years): 44

**BACKGROUND**

A number of CL cases were identified in former South-West Africa (now Namibia) in the 1970s [1]. As environmental conditions are very similar in the neighboring Northern Cape province of South Africa, it is thought that CL is likely to be present there as well.

**PARASITOLOGICAL INFORMATION**

| ***Leishmania* species** | **Clinical form** | **Vector species** | **Reservoirs** |
| --- | --- | --- | --- |
| Unknown | CL | Unknown | Unknown |

**MAPS & TRENDS, CONTROL, DIAGNOSIS & TREATMENT, ACCESS TO CARE and ACCESS TO DRUGS**

No information available.

**SOURCES OF INFORMATION**

1. Rutherfoord GS, Uys CJ (1978). Cutaneous Leishmaniasis in Southern Africa. A Case Report. South African Medical Journal. 6 May, 716-18.
