# Supplementary material for: Leishmaniasis Worldwide and Global Estimates of Its Incidence
Source: PLoS One. 2012 May 31;7(5):e35671. doi: 10.1371/journal.pone.0035671 (PMC3365071; doi:10.1371/journal.pone.0035671)
Supplement: Text S81 — Leishmaniasis Country Profiles, South Sudan. (DOCX) [file pone.0035671.s081.docx]

**SOUTH SUDAN**


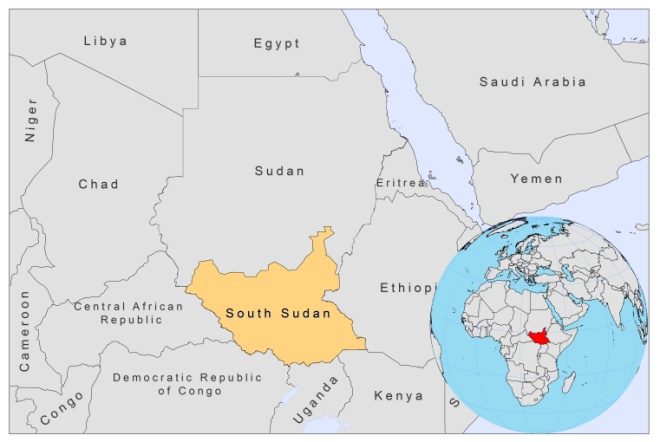


**BASIC COUNTRY DATA**

No official data available yet.

**BACKGROUND INFORMATION**

One of the distinctive characteristics of VL in South Sudan is that outbreaks occur regularly and unexpectedly in areas that were previously assumed to be unaffected. The main risk factor for these epidemics is introduction of the disease in non-immune populations through migrations from endemic to non-endemic areas. In 1937, the first reported outbreak occurred in the Dinka population of what is now Jonglei State, a previously non-endemic area [1]. Outbreaks continued to occur in this area during the 1960s [2]. In 1956, a VL outbreak severely affected nomadic tribes in the Southern Fung district of the former Blue Nile province, an area where the disease had not been reported for 25 years [3]. The mortality rate in the Yum Yum tribe was estimated to be over 50%. Between 1984 and 1994, during the Sudanese civil war, a devastating outbreak took place in former Western Upper Nile (now called Unity State), in an area that spanned from Bentiu southward to Adok [4-6]. Over 100,000 people, among a population of 280,000, were estimated to be killed [5,7,8]. Most were from semi-nomadic Nuer and Dinka tribes, dependent on cattle rearing and small-scale cultivation of sorghum and maize for their survival. The war completely disrupted their way of life, causing persistent large-scale malnutrition. It also forced them to migrate to remote and isolated areas, from where they could not reach treatment centers. The civil war also forced many people to seek shelter and food in the Acacia-Balanites woodlands, the natural habitat of the sandfly. Unity State was not known to be endemic for VL and the population was largely non-immune. The epidemic followed the return of soldiers from the endemic Sudan-Ethiopian border.

In 1994, a VL outbreak occurred in northern Jonglei and eastern Upper Nile States, also an area where mainly Nuer nomadic tribes live. This outbreak was also prompted by the war and food shortages. A much more severe outbreak followed in the same area in 2002-2003 [7]. Afterwards, the numbers of reported cases in this area gradually declined to become historically low, but at the end of 2009, a new outbreak started, with thousands of cases, possibly caused by massive population movements following the peace agreement in 2005 and growing food insecurity. This outbreak continued to cause extremely high case numbers in both 2010 and 2011, however, due to an increased access to treatment, the average case fatality was only 3,8%, much lower than previously reported care fatality rates of 30% in South Sudanese outbreaks.

Ethnicity was identified as a risk factor for disease in several Sudanese epidemic sites [9-11]. Sudanese tribal people appear to be exceptionally susceptible for developing full blown VL. A genetic factor has been considered.

An estimated 10% of VL cases in Southern Sudan is coinfected with HIV.

**PARASITOLOGICAL INFORMATION**

| ***Leishmania* species** | **Clinical form** | **Vector species** | **Reservoirs** |
| --- | --- | --- | --- |
| *L. donovani* | VL, PKDL, MCL | *P. martini* | Human |

**MAPS AND TRENDS**

**Visceral leishmaniasis**

**
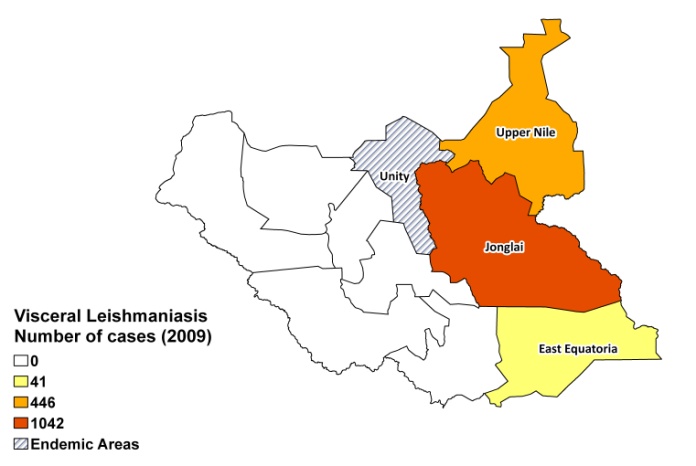
** **
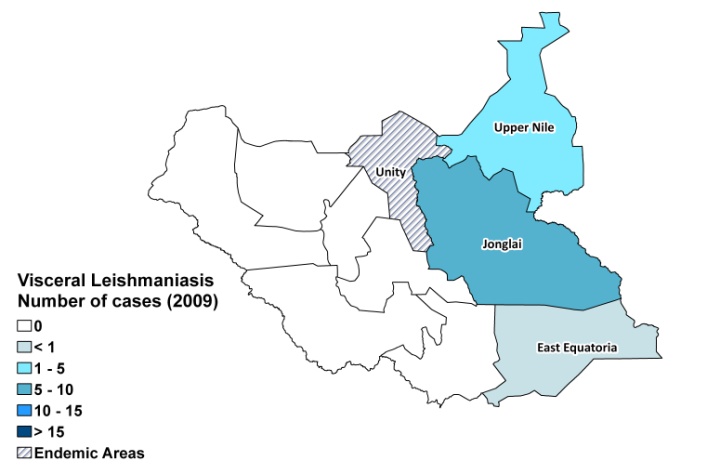
**

**Visceral leishmaniasis trend**

**CONTROL**

There is no national leishmaniasis control program and notification of cases is not mandatory. Leishmaniasis National Guidelines were developed in April 2011. There is no leishmaniasis vector control program and no bednet distribution program in South Sudan.

**DIAGNOSIS, TREATMENT**

**Diagnosis** (Malakal teaching hospital)

VL: confirmation is done by microscopic examination of lymph or spleen aspirate.

**Treatment**

VL: antimonials, 20 mg Sb^v^/kg/day for 30 days.

In MSF’s treatment programs, rK39 antigen-based rapid tests are used as first line diagnosis and a regimen of SSG and paromomycin (20 mg Sb^v^/kg/day and 15 mg PM/kg/day for 17 days) as first line treatment. Second line treatment is with liposomal amphotericin B, 5 mg/kg for 6 days.

**ACCESS TO CARE**

The only MoH facility providing VL care (formally free of charge) is the Malakal Teaching Hospital. MSF was until 2008 the only NGO providing treatment for VL on a significant scale in South Sudan; since then other NGO’s have joined in the effort. WHO and the Malaria Consortium are donating drugs (MSF provide their own drugs for treatment programs), and WHO is as supporting with funding, coordination and capacity building. Access to care has significantly improved because of this, however, still many locations with suspected patients cannot be reached because of logistic difficulties and security concerns, and where is a great lack of access to treatment. Patients are extremely poor and live mostly in very remote areas with no health facilities and no transport and thus often seek treatment only in a very late stage of the disease. There is a widespread lack of awareness of the serious nature of VL among people. About 2% of people use the private sector (illegal drug markets) for treatment, even though prices are prohibitive.

**ACCESS TO DRUGS**

Sodium stibogluconate, miltefosine 10 and 50 mg tablets and paromomycin are included in the National Essential Drug List for South Sudan. Liposomal amphotericin B is not included, but there is permission to import and use it in South Sudan. Generic sodium stibogluconate is available in unregulated drug markets. One (30 ml) vial of generic SSG (Albert David) is sold for the price of a bull.

The sale of sodium stibogluconate in unregulated markets is of great concern. Resistance to antimonials is still rare in South Sudan, but this practice will promote misuse and the possible development of resistant strains.

**SOURCES OF INFORMATION**

- Dr Godwin ‘O. Orkeh, Emergencies & Communicable Diseases, WHO – Southern Sudan Office. *Consultative Meeting on The Control of Leishmaniasis in the African Region. WHO/AFRO Addis Ababa, 23-25 Feb 2010*
- Dr Koert Ritmeijer, MSF Holland

1. Stephenson R (1940). An epidemic of kala-azar in the Upper Nile province of the Anglo-Egyptian Sudan. Ann Trop Med Parasitol 34:175-179.

2. Hoogstraal H, Heyneman D (1969). Leishmaniasis in the Sudan. Am J Trop Med Hyg (18):1091-1210.

3. Sati MH (1958). Early phases of an outbreak of kala-azar in the Southern Fung. Sudan Medical Journal 1 (98-111).

4. de Beer P, el Harith A, Deng LL, Semiao-Santos SJ, Chantal B, van Grootheest M (1991). A killing disease epidemic among displaced Sudanese population identified as visceral leishmaniasis. Am J Trop Med Hyg 44 (3):283-289.

5. Seaman J, Mercer AJ, Sondorp E (1996). The epidemic of visceral leishmaniasis in western Upper Nile, southern Sudan: course and impact from 1984 to 1994. Int J Epidemiol 25 (4):862-871.

6. Zijlstra EE, Ali MS, el-Hassan AM, el-Toum IA, Satti M, Ghalib HW, Sondorp E, Winkler A (1991). Kala-azar in displaced people from southern Sudan: epidemiological, clinical and therapeutic findings. Trans R Soc Trop Med Hyg 85 (3):365-369.

7. Ritmeijer K, Davidson RN (2003). Royal Society of Tropical Medicine and Hygiene joint meeting with Medecins Sans Frontieres at Manson House, London, 20 March 2003: field research in humanitarian medical programmes. Medecins Sans Frontieres interventions against kala-azar in the Sudan, 1989-2003. Trans R Soc Trop Med Hyg 97 (6):609-613.

8. Seaman J, Mercer AJ, Sondorp HE, Herwaldt BL (1996). Epidemic visceral leishmaniasis in southern Sudan: treatment of severely debilitated patients under wartime conditions and with limited resources. Ann Intern Med 124 (7):664-672.

9. Bucheton B, Kheir MM, El-Safi SH, Hammad A, Mergani A, Mary C, Abel L, Dessein A (2002). The interplay between environmental and host factors during an outbreak of visceral leishmaniasis in eastern Sudan. Microbes Infect 4 (14):1449-1457. doi:S1286457902000278 [pii].

10. Ibrahim ME (2002). The epidemiology of visceral leishmaniasis in east Africa: hints and molecular revelations. Trans R Soc Trop Med Hyg 96 Suppl 1:S25-29.

11. Zijlstra EE, el-Hassan AM, Ismael A, Ghalib HW (1994). Endemic kala-azar in eastern Sudan: a longitudinal study on the incidence of clinical and subclinical infection and post-kala-azar dermal leishmaniasis. Am J Trop Med Hyg 51 (6):826-836.
