# Supplementary material for: Leishmaniasis Worldwide and Global Estimates of Its Incidence
Source: PLoS One. 2012 May 31;7(5):e35671. doi: 10.1371/journal.pone.0035671 (PMC3365071; doi:10.1371/journal.pone.0035671)
Supplement: Text S82 — Leishmaniasis Country Profiles, Spain. (DOCX) [file pone.0035671.s082.docx]

**SPAIN**

**
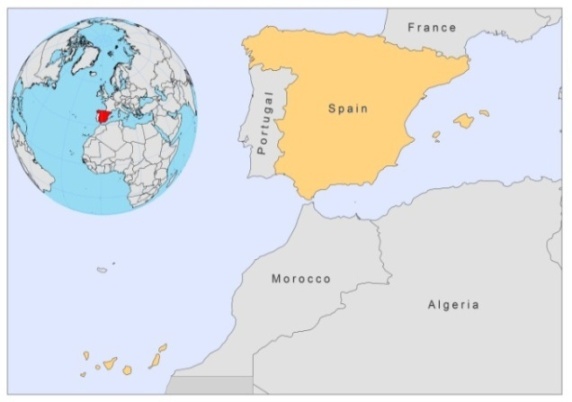
**

**BASIC COUNTRY DATA**

Total Population: 46,081,574

Population 0-14 years: 15%

Rural population: 23%

Population living under USD 1.25 a day: no data

Population living under the national poverty line: no data

Income status: High income economy: OECD

Ranking: Very high human development (ranking 23)

Per capita total expenditure on health at average exchange rate (US dollar): 3,076

Life expectancy at birth (years): 81

Healthy life expectancy at birth (years): 73

**BACKGROUND INFORMATION**

Leishmaniasis is hypoendemic in Spain (0.41 cases every 100,000 inhabitants) and is caused by *Leishmania infantum.* The dog is the main reservoir host. Between February 1982, when leishmaniasis was declared a notifiable disease, and December 1995 a total of 1,574 accumulated cases of leishmaniasis were reported [1]. VL and CL are not reported separately. In July 1996 a new decentralized surveillance system, based on the political structure of the autonomous regions was implemented, but leishmaniasis is no longer a notifiable disease in any of the 17 regions of Spain. In order to get accurate information, a recent publication analyzed the VL cases recorded in hospital databases during the period 1997-2008 [2], concluding that one third of these patients was coinfected with HIV. The incidence of hospitalizations was highest in Madrid and on the Mediterranean coast, the mortality rate was 3.31%, and the economic burden of leishmaniasis in the 12-year study period represented more than € 13 million (€ 6,740 per patient). Cases that are not coinfected occur mostly in children.

Most of the *Leishmania*/HIV coinfected cases occur in adult males, aged 25-35 years, 74% of which are IV drug users [3,4]. The disease presents mainly as VL in these patients. Leishmaniasis in immunocompromised patients is due to reactivation of subclinical infections, but also transmitted by sharing used syringes among intravenous drug users [5]. The number of *Leishmania/*HIV co-infections peaked in 1994 and decreased significantly, after introduction of HAART, to less than 10 cases in 2008 [6,7].

CL is less frequent, but there is no accurate information. Cases of CL are rarely reported; only 2% of cases between 1989 and 2008. These cases are estimated to represent only a third of the real number of cases and are treated by dermatologists outside hospitals [1].

Canine VL is endemic and its prevalence varies in the different regions of Spain. Studies in different areas have detected prevalences varying from 4 to 35% seropositive dogs, with 7,8% seropositive dogs in the Madrid region and a high proportion of seropositive dogs not showing any clinical signs of leishmaniasis [8]. The incidence of human leishmaniasis then started to increase in the Madrid area [9], and during 2010 and 2011 an outbreak of both cutaneous and visceral leishmaniasis due to *L.infantum* occurred in the province of Madrid affecting three municipalities. 204 human cases (100 CL and 104 VL) have been reported in this period. Interestingly, hares may be a reservoir since 30% of 138 hares were found infected either in viscera or in skin, and a few of them were able to infect *Ph. perniciosus* by xenodiagnosis. Seropositivity in dogs was found to be 3,6% and 7,9% in two screenings, which equals the normal seroprevalence in this region. Environmental man-made changes may explain the high density of hares (WHO Symposium on Visceral Leishmaniasis Outbreaks, Madrid, March 2012).

**PARASITOLOGICAL INFORMATION**

| ***Leishmania* pecies** | **Clinical form** | **Vector species** | **Reservoirs** |
| --- | --- | --- | --- |
| *L. infantum* | ZVL, CL | *P. perniciosus, P. ariasi* | *Canis familiaris* |

**MAPS AND TRENDS**

**Cutaneous and visceral leishmaniasis**

**
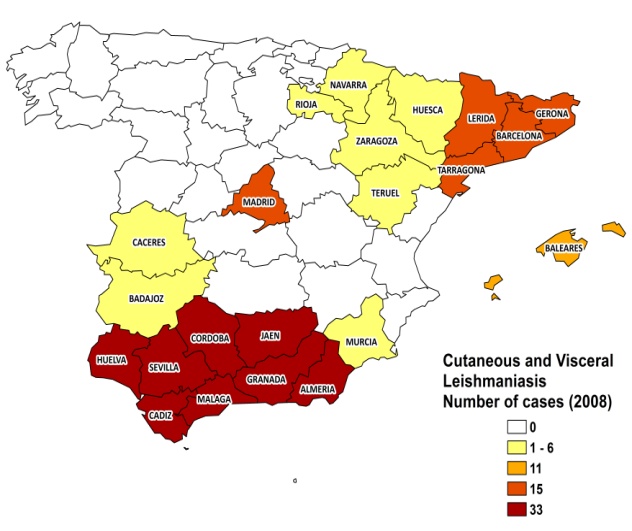
**

**Cutaneous and visceral leishmaniasis trend**

**CONTROL**

The notification of leishmaniasis is not mandatory in the country and there is no national leishmaniasis control program. There is no leishmaniasis vector control program, but some regions carry out control activities. Treatment or sacrifice of infected dogs is recommended by veterinarians. Infected stray dogs are sacrificed.

**DIAGNOSIS, TREATMENT**

**Diagnosis**

VL and CL: various serological and parasitological techniques.

**Treatment:**

VL: liposomal amphotericin B, 3-5 mg/kg/day, for 3-10 days, or antimonials, 20 mg Sb^v^/kg/day for 28 days. Fatality rate is 5%. Second line treatment with miltefosine.

**ACCESS TO CARE**

Health care is free in Spain, which includes care for leishmaniasis. Diagnosis is not performed on primary health care level, but in hospitals for VL, and by dermatologists for CL. All patients are thought to have access to care.

**ACCESS TO DRUGS**

Only pentamidine is included in the National Essential Drug List for VL. Drugs for leishmaniasis are not sold without prescription in pharmacies. Meglumine antimoniate (Glucantime, Sanofi) and liposomal amphotericin B (AmBisome, Gilead) are registered in Spain. Miltefosine is registered for use in dogs, but not for humans.

**SOURCES OF INFORMATION**

• Dr Luisa Sánchez Serrano, National Center of Epidemiology, Instituto de Salud Carlos III, Ministerio de Ciencia e Innovación. *Leishmaniasis in the European Region, a WHO consultative intercountry meeting, Istanbul, Turkey, 17–19 November 2009.*

1. Alvar J (2001) Lab. Las leishmaniasis. de la biología al control, InterVet S.A.; 2ª ed. 236 págs.

2. Gil-Prieto R, Walter S, Alvar J, Gil de Miguel A. Epidemiology of Leishmaniais in Spain based on hospitalization records (1997-2008). Am J Trop Med Hyg (in press).

3. Alvar J, Cañavate C, Gutiérrez-Solar B, Jiménez M, Laguna F et al (1997). Leishmania and human immunodeficiency virus coinfection: the first 10 years. Clin Microbiol Rev. 10(2):298--319.

4. López-Vélez R, Pérez Molina JA, Guerrero J, Baquero F, Villarrubia J et al (1998). Clinicoepidemiologic characteristics, prognostic factors, and survival análisis of patients coinfected with human immunodeficiency virus and Leishmania in an area of Madrid, Spain. Am J Trop Med Hyg 58:436--443.

5. Cruz I, Morales MA, Noguer I, Rodríguez A, Alvar J (2002). Leishmania in discarded syringes from intravenous drug users. Lancet 59 (9312):1124--5.

6. López-Vélez R, Casado JL, Pintado V (2001). Decline of a visceral leishmaniasis epidemic in HIV-infected patients after the introduction of highly active antiretroviral therapy (HAART). Clin Microbiol Infect 7:394--5.

7. Alvar J, Aparicio P, Aseffa A, Den Boer M, Cañavate C et al (2008). The relationship between leishmaniasis and AIDS: the second 10 years. Clin Microbiol Rev 21(2):334--59.

8. [Miró G](http://www.ncbi.nlm.nih.gov/pubmed?term=%22Mir%C3%B3%20G%22%5BAuthor%5D), [Montoya A](http://www.ncbi.nlm.nih.gov/pubmed?term=%22Montoya%20A%22%5BAuthor%5D), [Mateo M](http://www.ncbi.nlm.nih.gov/pubmed?term=%22Mateo%20M%22%5BAuthor%5D), [Alonso A](http://www.ncbi.nlm.nih.gov/pubmed?term=%22Alonso%20A%22%5BAuthor%5D), [García S](http://www.ncbi.nlm.nih.gov/pubmed?term=%22Garc%C3%ADa%20S%22%5BAuthor%5D) et al (2007). A leishmaniosis surveillance system among stray dogs in the region of Madrid: ten years of serodiagnosis (1996-2006). [Parasitol Res](http://www.ncbi.nlm.nih.gov/pubmed/17323100##) 101(2):253-7. Epub 2007 Feb 25.

9. [Gálvez R](http://www.ncbi.nlm.nih.gov/pubmed?term=%22G%C3%A1lvez%20R%22%5BAuthor%5D), [Miró G](http://www.ncbi.nlm.nih.gov/pubmed?term=%22Mir%C3%B3%20G%22%5BAuthor%5D), [Descalzo MA](http://www.ncbi.nlm.nih.gov/pubmed?term=%22Descalzo%20MA%22%5BAuthor%5D), [Nieto J](http://www.ncbi.nlm.nih.gov/pubmed?term=%22Nieto%20J%22%5BAuthor%5D), [Dado D](http://www.ncbi.nlm.nih.gov/pubmed?term=%22Dado%20D%22%5BAuthor%5D) et al (2910). Emerging trends in the seroprevalence of canine leishmaniasis in the Madrid region (central Spain). [Vet Parasitol](http://www.ncbi.nlm.nih.gov/pubmed/20031330##) 11;169(3-4):327-34.
