# Supplementary material for: Leishmaniasis Worldwide and Global Estimates of Its Incidence
Source: PLoS One. 2012 May 31;7(5):e35671. doi: 10.1371/journal.pone.0035671 (PMC3365071; doi:10.1371/journal.pone.0035671)
Supplement: Text S83 — Leishmaniasis Country Profiles, Sri Lanka. (DOCX) [file pone.0035671.s083.docx]

**SRI LANKA**

**
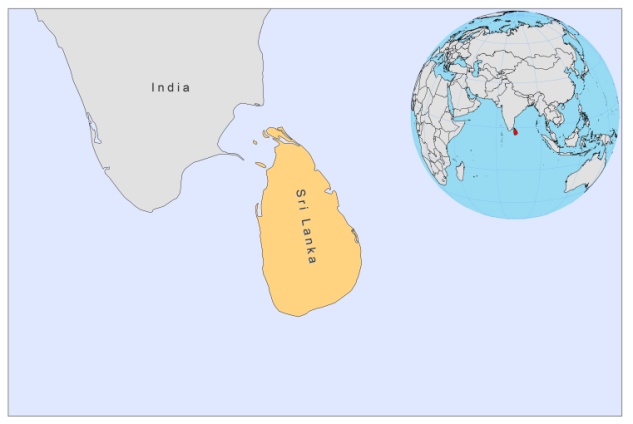
**

**BASIC COUNTRY DATA**

Total Population: 20,859,949

Population 0-14 years: 25%

Rural population: 85%

Population living under USD 1.25 a day: 7%

Population living under the national poverty line: 15.2%

Income status: Lower middle income economy

Ranking: Medium human development (ranking 97)

Per capita total expenditure on health at average exchange rate (US dollar): 84

Life expectancy at birth (years): 75

Healthy life expectancy at birth (years): 62

**BACKGROUND INFORMATION**

Sri Lanka is endemic for CL by *L.donovani*, and results taken from genotyping studies point to a long history of the parasite in Sri Lanka. However, no autochtonous cases were described until 1992 [1]. In 1978, a report described several CL cases, but these were imported from Saudi Arabia. *L.donovani* is thought to have been prevalent in Sri Lanka without causing many cases or possibly with symptoms misdiagnosed by medical personnel.

After the first report of CL in 1992, it has become an established disease, with an explosive increase in numbers, and now affects almost all provinces. The reason for its spread has been associated with the movement of military personnel into former uninhabited areas and population movements due to the civil war. Over 2,000 cases have been reported between 2001 and 2011, and this number is seen as an underrepresentation of the true incidence of the disease [2]. Infections are found in all ages; there is a difference, however, between the North Central province, where most cases are in between the ages of 25 and 39 and mostly among soldiers, and the South, where males and females are equally affected and most cases are between 10-19 years [3,4]. The disease was mostly reported from low-altitude areas of Sri Lanka. The reservoir is unknown and may be zoonotic. In population surveys held in Welioya (n=6279), Kataragama (n=200) and Mamadala (n=2868), CL prevalences of resp. 2.4%, 0.5% and 3.5% were found.

Several recent cases of MCL [5] and VL [6] have been reported and the risk of future VL outbreaks is of concern and cannot be excluded [2].

**PARASITOLOGICAL INFORMATION**

| ***Leishmania* species** | **Clinical form** | **Vector species** | **Reservoirs** |
| --- | --- | --- | --- |
| *L. donovani* | AVL, CL, PKDL | *P. argentipes* | Human |

**MAPS AND TRENDS**

**Cutaneous leishmaniasis**


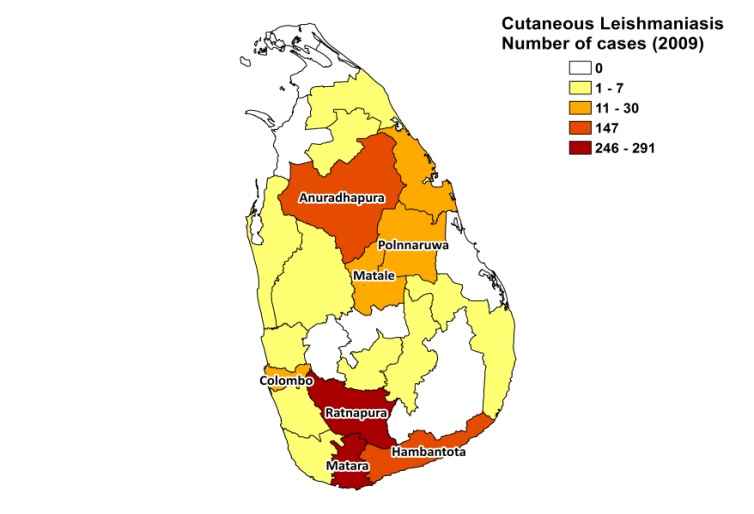


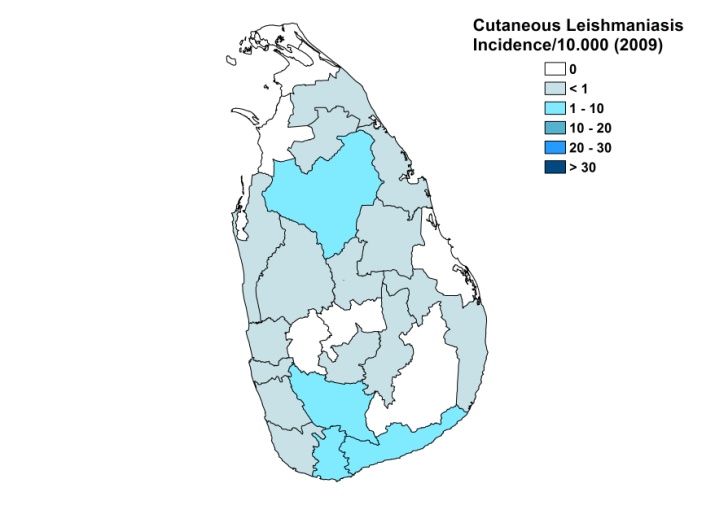


**Trend in cutaneous leishmaniasis**

**CONTROL**

The notification of CL and VL has been mandatory since 2008. A focal point for the control of leishmaniasis was set up in September of 2008; an action plan was drawn up in 2009. Case detection is passive. At present, no organized control efforts are made for the control of CL.

**DIAGNOSIS, TREATMENT**

**Diagnosis**

CL: on clinical picture and confirmation with microscopic examination of skin lesion sample (only in specialized hospitals). PCR can be carried out in specialized laboratories.

VL: microscopic evaluation of splenic/bone marrow/lymph node aspirates.

**Treatment**

CL: cryotherapy is available in most district-level hospitals. In some major hospitals with functional dermatology units, topical therapy with sodium stibogluconate is provided.

**ACCESS TO CARE**

Treatment for CL is provided free of charge. Patients with CL are thought to have good access to care, but the duration of symptoms may last years before treatment is sought (one month to 6 years, median 5 months). Reasons are a lack of awareness among patients and doctors, the self-healing nature of the disease, and the difficulties patients from remote areas have in accessing hospitals, especially because cryotherapy is required to be repeated for several weeks. Treatment is not offered in the primary health care setting.

**ACCESS TO DRUGS**

Sodium stibogluconate and amphotericin B are included in the National Essential Drug List for leishmaniasis. No drugs are registered for leishmaniasis. Sodium stibogluconate is provided in specialized hospitals, but its use is limited due to its high cost.

**SOURCES OF INFORMATION**

- Dr Nadira D. Karunaweera, department of Parasitology, Faculty of Medicine, University of Colombo. *WHO informal consultation on epidemiological information on disease burden due to kala-azar in Bangladesh, India and Nepal. Paro, Bhutan, 8-10 March 2011.*

1. Athukorale DN, Seneviratne JK, Ihalamulla RL, Premaratne UN (1992). [Locally acquired cutaneous leishmaniasis in Sri Lanka.](http://www.ncbi.nlm.nih.gov/pubmed/1460704) J Trop Med Hyg 95(6):432-3.

2. Karunaweera ND (2009). [Leishmania donovani causing cutaneous leishmaniasis in Sri Lanka: a wolf in sheep's clothing?](http://www.ncbi.nlm.nih.gov/pubmed/19734098) Trends Parasitol 25(10):458-63.

3. Siriwardena HV, Udagedara CU, Karunaweera ND (2003). [Clinical features, risk factors and efficacy of cryotherapy in cutaneous leishmaniasis in Sri Lanka.](http://www.ncbi.nlm.nih.gov/pubmed/12795012) Ceylon Med J 48(1):10-2.

4. Rajapaksa US, Ihalamulla RL, Udagedera C, Karunaweera ND (2007). [Cutaneous leishmaniasis in southern Sri Lanka.](http://www.ncbi.nlm.nih.gov/pubmed/17499826) Trans R Soc Trop Med Hyg 101(8):799-803.

5. Rajapaksa US, Ihalamulla RL, Karunaweera ND (2005). [First report of mucosal tissue localisation of leishmaniasis in Sri Lanka.](http://www.ncbi.nlm.nih.gov/pubmed/16114778) Ceylon Med J 50(2):90-1.

6. Abeygunasekara PH, Costa YJ, Seneviratne N, Ratnatunga N, Wijesundera Mde S (2007). [Locally acquired visceral leishmaniasis in Sri Lanka.](http://www.ncbi.nlm.nih.gov/pubmed/17585579) Ceylon Med J 52(1):30-1.
