# Supplementary material for: Leishmaniasis Worldwide and Global Estimates of Its Incidence
Source: PLoS One. 2012 May 31;7(5):e35671. doi: 10.1371/journal.pone.0035671 (PMC3365071; doi:10.1371/journal.pone.0035671)
Supplement: Text S84 — Leishmaniasis Country Profiles, Sudan. (DOCX) [file pone.0035671.s084.docx]

**SUDAN**

**
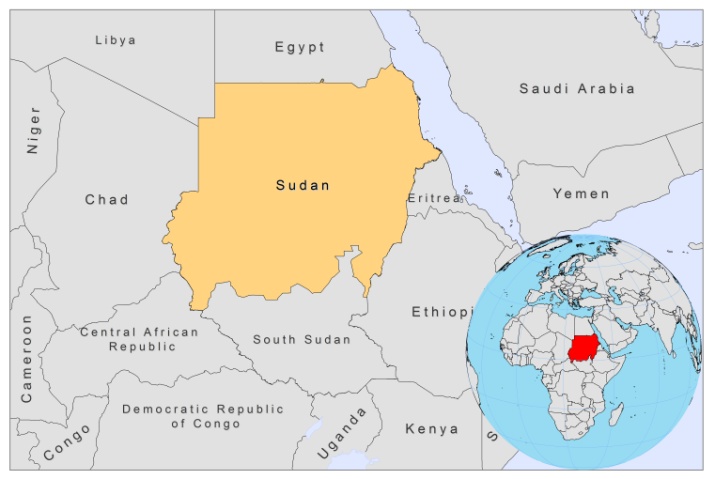
**

**BASIC COUNTRY DATA**

Total Population: 43,551,941

Population 0-14 years: 40%

Rural population: 55%

Population living under USD 1.25 a day: no data

Population living under the national poverty line: no data

Income status: Lower middle income economy

Ranking: Low human development (ranking 169)

Per capita total expenditure on health at average exchange rate (US dollar): 95

Life expectancy at birth (years): 61

Healthy life expectancy at birth (years): 49

**BACKGROUND INFORMATION**

Sudan is the original focus of VL; *L.donovani* DNA was found in bone marrow samples from ancient Egyptian and Nubian mummies originating from around 4000 BC [1] and it has been proposed that the *Leishmania* parasite has evolved before or at the same time as *Homo sapiens* in East Africa [2]. The reported occurrence of VL in Sudan is wide, erratic and variable [3]. Following the first reported case of VL in 1904, only sporadic cases were reported until the 1930s. Then, endemic areas started to expand considerably and erratically, following an epidemic pattern [4,5]. Gedaref State, in northern Sudan, is the only known stable hyperendemic region in Sudan, with a consistently high annual incidence of thousands of reported cases.

In 1990-1992, VL also spread northwards to the south of Kordofan State, with nomadic tribes moving between this area and Western Upper Nile, causing an outbreak in the non-immune population [3,6]. Since 2006, after VL had been absent for 25 years, a surge of new cases has been observed in White Nile State, central Sudan, close to Khartoum [7]. Transmission during epidemics is predominantly anthroponotic. However, VL occurred in game wardens in uninhabited Dinder National Park, suggesting transmission was zoonotic in this case [8]. However, no important animal reservoir has been identified since the discovery of the first case of VL in Sudan, although thousands of small and large mammals and reptiles have been investigated [3]. Climatic events, such as increased annual rainfall, have preceded past epidemics on several occasions [3,9]. In Gedaref state, annual rainfall and altitude were the best predictors, among many factors that were studied, for the occurrence of VL [10]. A resurgence of the sandfly population, as a consequence of regrowth of Acacia and Balanites forests destroyed by floods, or the interrupted insecticide spraying have also been associated with epidemics [3,11].

PKDL occurs in about 55% of Sudanese patients [12]. No HIV coinfected cases have yet been found. Mucosal involvement in VL is uncommon; since the disease was first described in 1914, only 64 cases, mostly in adult males from several closely related tribes in western Sudan, were reported up until 1992 [13].

The first autochtonous case of CL was found in 1911 [14]. The reporting of sporadic cases was followed by three outbreaks: in the Shendi Atbara area in 1976-1977, in El Garrasa in the White Nile area early 1985, and in Tuti island, at the junction of the Blue and White Nile, in 1985. In 1986, the number of cases increased dramatically; all age groups were affected with a total of 100,000 cases. Currently, CL is endemic in Darfur and Kordofan, in addition to the area from Khartoum state up to Atbra, in the northern part of the country.

**PARASITOLOGICAL INFORMATION**

| ***Leishmania* species** | **Clinical form** | **Vector species** | **Reservoirs** |
| --- | --- | --- | --- |
| *L. donovani* | VL, PKDL, ML | *P. orientalis, P. martini* | Human |
| *L. infantum* | ZVL | Unknown | *Canis familiaris* |
| *L. major* | ZCL, ML | *P. papatasi, P. duboscqi* | *Arvicantis niloticus* |

**MAPS AND TRENDS**

**Visceral leishmaniasis**

**
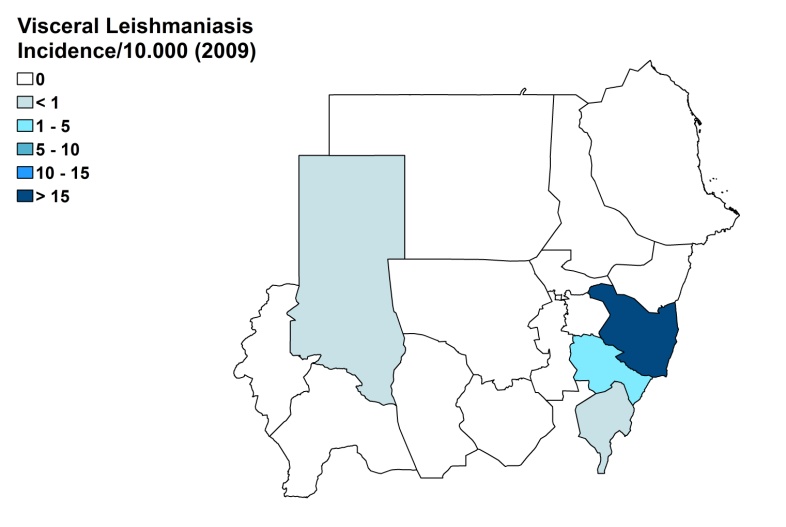

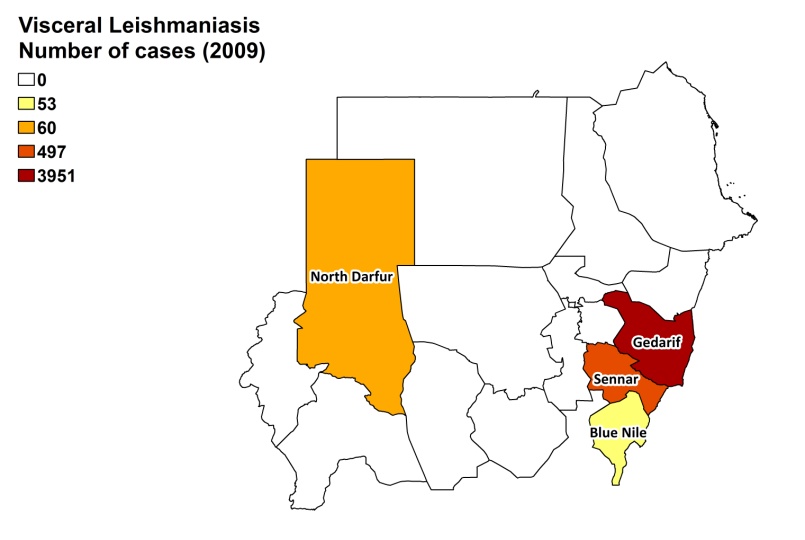
**

**Cutaneous leishmaniasis**

**
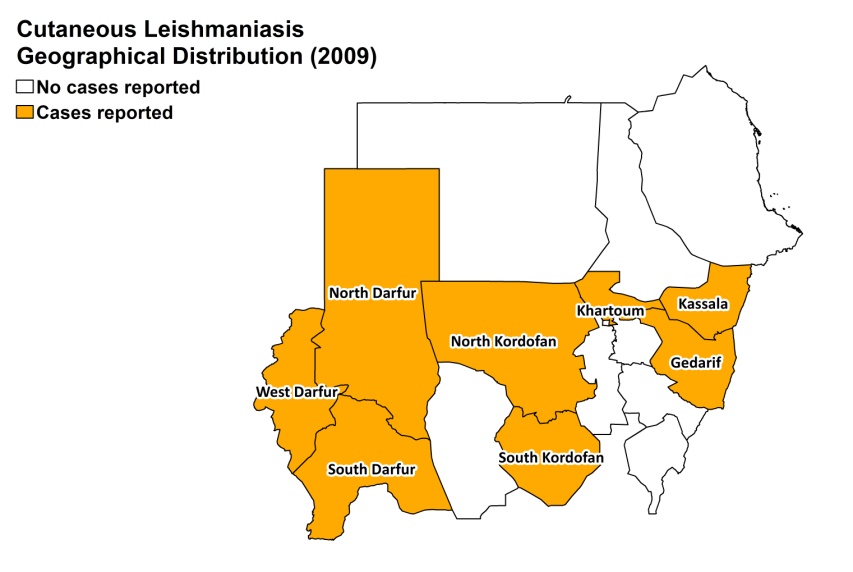
**

**Visceral leishmaniasis trend**

**Cutaneous leishmaniasis**

| **Year** | **Cases** |
| --- | --- |
| **2011** | **6062** |

**CONTROL**

In Sudan, there is a leishmaniasis control program addressing both CL and VL and notification of VL is mandatory. There is no leishmaniasis vector control program and no bednet distribution program in Sudan.

**DIAGNOSIS, TREATMENT**

**Diagnosis:**

VL: confirmation by microscopic examination of lymph or bone marrow aspirate.

CL: only diagnosed in one specialized center, by microscopic examination of skin lesions samples.

**Treatment**:

VL: antimonials, 20 mg Sb^v^/kg/day for 30 days. Second line: liposomal amphotericin B, 3 mg/kg/alternate days for 14 days (but this drug is not yet in use). Cure rate with antimonials is close to 100%, with 3% recurring cases. PKDL occurs in 60-80%, severe adverse events in 20% of cases and fatality rate is less than 1%.

CL: antimonials.

**ACCESS TO CARE**

The Ministry of Health, in collaboration with other organizations and NGOs (MSF, IED, DNDi, WHO), provides treatment free of charge for patients, but in teaching hospitals there are fees for registration (2 USD), hospitalization (7 USD) and lab tests (variable). The Ministry of Health provided antimonials for about 1,000 patients in 2007 and 2008. In addition, NGOs donated substantial amounts of antimonials in Gedaref these same years. However, there were severe shortages of antimonials in 2007-2009, which meant a lack of access to treatment for many. Another problem is that there are not enough health facilities where VL can be treated; poor patients mostly live in remote areas with no transport and no awareness of the disease. In Gedaref, about 10% seeks care in private clinics.

CL is mostly not diagnosed nor treated in Sudan.

**ACCESS TO DRUGS**

Sodium stibogluconate, amphotericin B and liposomal amphotericin B are included in the National Essential Drug List for Sudan, and there is permission for the use of meglumine antimoniate. During the shortage of antimonials in the past years, generic sodium stibogluconate was sold in pharmacies in Gedaref for 10 USD per vial (leading to an adult treatment cost of 70 USD). Sodium stibogluconate (Pentostam, GSK) is registered in Sudan.

**SOURCES OF INFORMATION**

- Dr Muzamil Abelraheem WHOL/FMoH North Sudan.
- Dr Musa, Institute of Endemic Diseases, University of Khartoum.
- Dr Manica Balasegeram, DNDi.
- Dr Koert Ritmeijer, MSF-Holland.

1. Zink AR, Spigelman M, Schraut B, Greenblatt CL, Nerlich AG, Donoghue HD (2006) Leishmaniasis in ancient Egypt and Upper Nubia. Emerg Infect Dis 12 (10):1616-1617.

2. Ibrahim ME (2002) The epidemiology of visceral leishmaniasis in east Africa: hints and molecular revelations. Trans R Soc Trop Med Hyg 96 Suppl 1:S25-29.

3. Zijlstra EE, el-Hassan AM (2001) Leishmaniasis in Sudan. Visceral leishmaniasis. Trans R Soc Trop Med Hyg 95 Suppl 1:S27-58.

4. Stephenson R (1940) An epidemic of kala-azar in the Upper Nile province of the Anglo-Egyptian Sudan. Ann Trop Med Parasitol 34:175-179..

5. Hoogstraal H, Heyneman D (1969) Leishmaniasis in the Sudan. Am J Trop Med Hyg (18):1091-1210.

6. Hashim FA, Ali MS, Satti M, el-Hassan AM, Ghalib HW, el Safi S, el Hag IA (1994) An outbreak of acute kala-azar in a nomadic tribe in western Sudan: features of the disease in a previously non-immune population. Trans R Soc Trop Med Hyg 88 (4):431-432.

7. Khalil EA, Musa AM, Elgawi SH, Meshasha A, Gamar Eldawla I, Elhassan MO, Eljaleel KA, Younis BM, Elfaki ME, El-Hassan AM (2008) Revival of a focus of visceral leishmaniasis in central Sudan. Ann Trop Med Parasitol 102 (1):79-80. doi:10.1179/136485908X252269.

8. Ibrahim ME, Lambson B, Yousif AO, Deifalla NS, Alnaiem DA, Ismail A, Yousif H, Ghalib HW, Khalil EA, Kadaro A, Barker DC, El Hassan AM (1999) Kala-azar in a high transmission focus: an ethnic and geographic dimension. Am J Trop Med Hyg 61 (6):941-944.

9. Marlet MV, Sang DK, Ritmeijer K, Muga RO, Onsongo J, Davidson RN (2003) Emergence or re-emergence of visceral leishmaniasis in areas of Somalia, north-eastern Kenya, and south-eastern Ethiopia in 2000-01. Trans R Soc Trop Med Hyg 97 (5):515-518.

10. Elnaiem DE, Schorscher J, Bendall A, Obsomer V, Osman ME, Mekkawi AM, Connor SJ, Ashford RW, Thomson MC (2003) Risk mapping of visceral leishmaniasis: the role of local variation in rainfall and altitude on the presence and incidence of kala-azar in eastern Sudan. Am J Trop Med Hyg 68 (1):10-17.

11. Zijlstra EE, Ali MS, el-Hassan AM, el-Toum IA, Satti M, Ghalib HW, Sondorp E, Winkler A (1991) Kala-azar in displaced people from southern Sudan: epidemiological, clinical and therapeutic findings. Trans R Soc Trop Med Hyg 85 (3):365-369.

12. Zijlstra EE, el-Hassan AM (2001) Leishmaniasis in Sudan. Post kala-azar dermal leishmaniasis. Trans R Soc Trop Med Hyg 95 Suppl 1:S59-76.

13. el-Hassan AM, Meredith SE, Yagi HI, Khalil EA, Ghalib HW, Abbas K, Zijlstra EE, Kroon CC, Schoone GJ, Ismail A (1995) Sudanese mucosal leishmaniasis: epidemiology, clinical features, diagnosis, immune responses and treatment. Trans R Soc Trop Med Hyg 89 (6):647-652.

14. el-Hassan AM, Zijlstra EE (2001) Leishmaniasis in Sudan. Cutaneous leishmaniasis. Trans R Soc Trop Med Hyg 95 Suppl 1:S1-17.
