# Supplementary material for: Leishmaniasis Worldwide and Global Estimates of Its Incidence
Source: PLoS One. 2012 May 31;7(5):e35671. doi: 10.1371/journal.pone.0035671 (PMC3365071; doi:10.1371/journal.pone.0035671)
Supplement: Text S85 — Leishmaniasis Country Profiles, Surinam. (DOCX) [file pone.0035671.s085.docx]

**SURINAME**


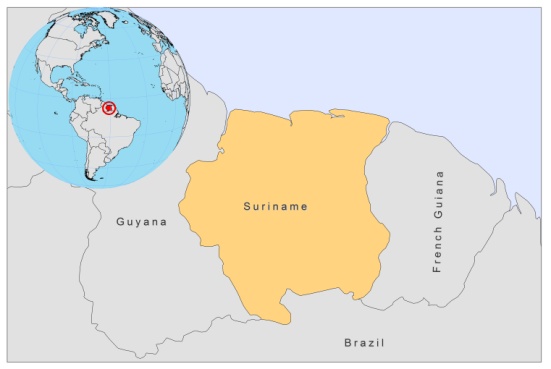


**BASIC COUNTRY DATA**

Total Population: 524,636

Population 0-14 years: 29%

Rural population: 24%

Population living under USD 1.25 a day: no data

Population living under the national poverty line: no data

Income status: Upper middle income economy

Ranking: Medium human development (ranking 104)

Per capita total expenditure on health at average exchange rate (US dollar): 1,558

Life expectancy at birth (years): 70

Healthy life expectancy at birth (years): 59

**BACKGROUND INFORMATION**

The first case of CL was reported in 1911. Now, 10 districts are affected, the majority of which is located in the interior forested area of the country. CL is known locally as "Boschyaws" or "Boessie-Yassi". It is an increasing health threat in Suriname, mainly affecting poor populations in the interior and gold miners, mostly Brazilians, from across the border. Gold miners are especially thought to be at high risk of contracting CL [1]. Incidence is high; 5.32 to 6.13/1,000 in the hinterland and 0.64 to 0.74 patients per 1,000 inhabitants for the whole country, and likely to be underestimated. 49% of patients, in a prospective review, were women and children, suggesting that CL has become endemic within villages ^2^.

Only one case of VL has been reported in 1953 [2]. There is no reported HIV/*Leishmania* coinfection.

**PARASITOLOGICAL INFORMATION**

| ***Leishmania* species** | **Clinical form** | **Vector species** | **Reservoirs** |
| --- | --- | --- | --- |
| *L. guyanensis* | ZCL | *Lu. umbratilis, Lu. anduzei* | Unknown |
| *L. amazonensis* | ZCL | *Lu. flaviscutellata* | Unknown |
| *L. lainsoni* | ZCL | Unknown | Unknown |

**MAPS AND TRENDS**

**Cutaneous leishmaniasis**

**
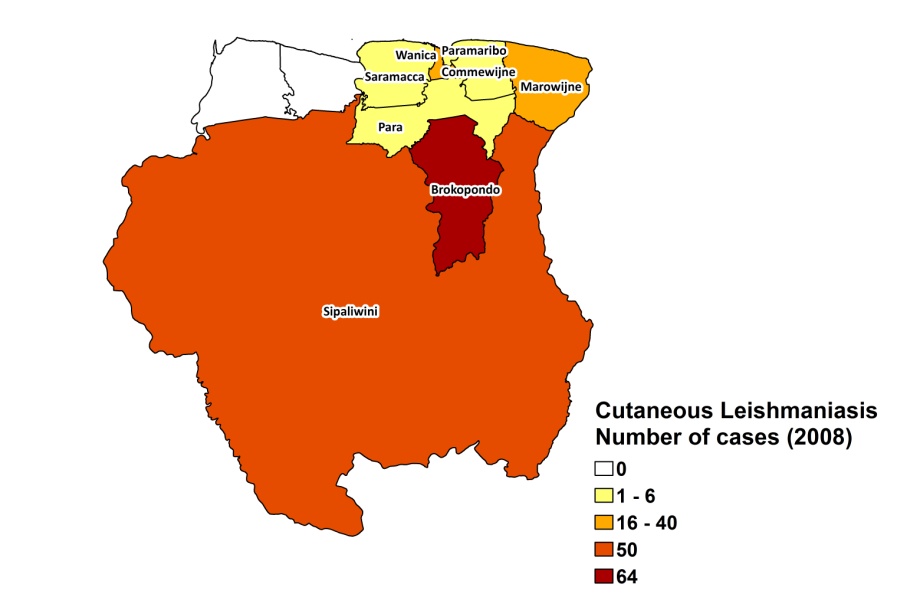

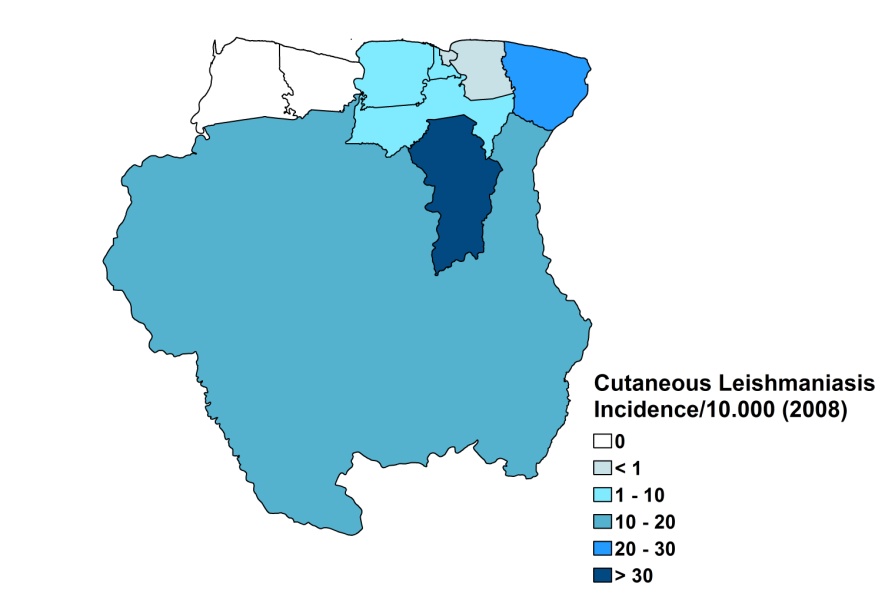
**

**Cutaneous leishmaniasis trend**

**CONTROL**

The notification of leishmaniasis is not mandatory in the country and there is no national leishmaniasis control program (it is expected to be established in the coming 5 years). Case detection is passive. There are no leishmaniasis vector or reservoir control programs. Bednet distribution is done in the context of malaria control.

**DIAGNOSIS, TREATMENT**

**Diagnosis**

CL: microscopic examination of skin lesion sample. PCR is only possible at a research laboratory.

**Treatment**

CL: pentamidine, with a total dose of 300 mg/week, for a minimum of 3 weeks. Cure rate is 90% with 10% recurring lesions. Amphothericin B is used as second line drug.

**ACCESS TO CARE**

Diagnosis and treatment for CL is free if it includes drugs from the National Essential Drug List. The government provides pentamidine (Sanofi and Combinopharm, Spain) and amphotericin B. Access to miltefosine is problematic, as this is not provided for free. Treatment takes place in health centers all over the country and specialized centers in Paramaribo. Some patients are treated in the Medical Mission Hospital. The amount of essential drugs in health facilities is not always sufficient; however, availability went up by 50-90% in the last 6 months. Access to treatment is thought to be satisfactory, however, some patients live in very remote areas with no health facilities and transport, such as the gold miners that live deep in the forest. These are known to self-medicate and not report to clinics. The indigenous population in the forest often seeks traditional care before reporting to health facilities.

**ACCESS TO DRUGS**

Conventional amphotericin B and pentamidine are included in the National Essential Drug List. Both drugs are supplied by the Ministry of Health, and are also available in regulated pharmacies, where pentamidine is sold for 36 USD per vial. Drugs for treatment of CL are free in the clinics in the interior of the country, but at times, drug supply at public health facilities is not sufficient and many patients lack the resources to purchase them in pharmacies in Paramaribo, the capital. No pentavalent antimonials are registered or available in Suriname.

**SOURCES OF INFORMATION**

- Dr Leslie Sabajo, Dermatology Service and Dr Miriam Naarendorp, Pharmacy policy.

1. V[an der Meide WF](http://www.ncbi.nlm.nih.gov/pubmed?term=%22van%20der%20Meide%20WF%22%5BAuthor%5D), [Jensema AJ](http://www.ncbi.nlm.nih.gov/pubmed?term=%22Jensema%20AJ%22%5BAuthor%5D), [Akrum RA](http://www.ncbi.nlm.nih.gov/pubmed?term=%22Akrum%20RA%22%5BAuthor%5D), [Sabajo LO](http://www.ncbi.nlm.nih.gov/pubmed?term=%22Sabajo%20LO%22%5BAuthor%5D), [Lai A Fat RF](http://www.ncbi.nlm.nih.gov/pubmed?term=%22Lai%20A%20Fat%20RF%22%5BAuthor%5D) et al (2008). Epidemiology of cutaneous leishmaniasis in Suriname: a study performed in 2006. [Am J Trop Med Hyg](javascript:AL_get(this,%20'jour',%20'Am%20J%20Trop%20Med%20Hyg.');) 79(2):192-7.

2. [Winckel WE](http://www.ncbi.nlm.nih.gov/pubmed?term=%22WINCKEL%20WE%22%5BAuthor%5D), [Aalstein M](http://www.ncbi.nlm.nih.gov/pubmed?term=%22AALSTEIN%20M%22%5BAuthor%5D). First case of kala-azar in Surinam (1953). [Doc Med Geogr Trop](javascript:AL_get(this,%20'jour',%20'Doc%20Med%20Geogr%20Trop.');) 4:339-42.
