# Supplementary material for: Leishmaniasis Worldwide and Global Estimates of Its Incidence
Source: PLoS One. 2012 May 31;7(5):e35671. doi: 10.1371/journal.pone.0035671 (PMC3365071; doi:10.1371/journal.pone.0035671)
Supplement: Text S86 — Leishmaniasis Country Profiles, Syrian Arab Republic. (DOCX) [file pone.0035671.s086.docx]

**SYRIAN ARAB REPUBLIC**


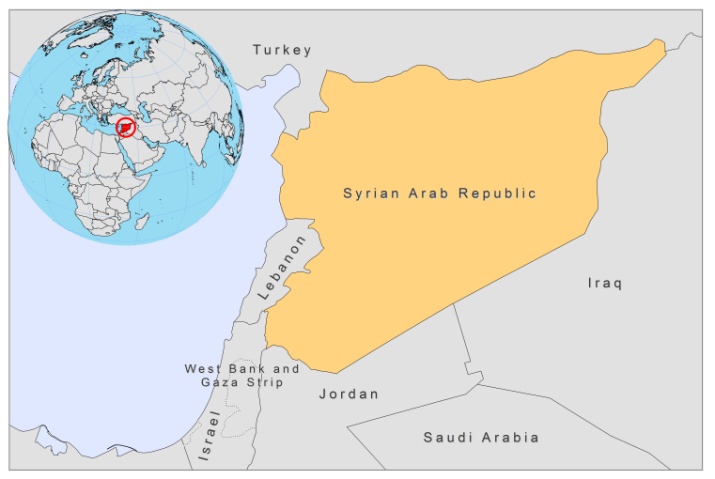


**BASIC COUNTRY DATA**

Total Population: 20,446,609

Population 0-14 years: 37%

Rural population: 45%

Population living under USD 1.25 a day: no data

Population living under the national poverty line: no data

Income status: Lower middle income economy

Ranking: Medium human development (ranking 119)

Per capita total expenditure on health at average exchange rate (US dollar): 72

Life expectancy at birth (years): 76

Healthy life expectancy at birth (years): 62

**BACKGROUND INFORMATION**

Only a few cases of VL are recorded yearly, mainly from Edlib province. VL is probably underreported. Serological screenings showed up to 23% seropositivity, but no cases with full-blown disease [1].

CL is a very old endemic disease in Syria. In the 1950s, antimalarial spraying caused a considerable decrease of the incidence and during the years that followed, just a few cases were reported [2]. After this decline, in 1985, the number of CL cases started increasing sharply again [3,4], with nearly 30,000 cases reported in 2008, almost double the number of cases of the previous year.

CL caused by *L. tropica* is still endemic in its traditional home of Aleppo, but also in Edlib, Lattakia, Tortous, Hama and the city of Damascus. It now represents about 90% of all CL cases and is one of the most important public health problems in the Syrian Arab Republic, especially in Aleppo. The population density in urban areas of the Syrian Arab Republic has increased, as well as population movement across the country, which could explain the rise in cases. As a recent survey estimated that 20% of cases are not reported or treated, a considerable human reservoir exists.

CL caused by *L.major* is less common and occurs in rural areas close to Damascus, Deir al Zour and Al Hasakeh [5].

No cases of HIV/*Leishmania* co-infection are reported.

**PARASITOLOGICAL INFORMATION**

| ***Leishmania* species** | **Clinical form** | **Vector species** | **Reservoirs** |
| --- | --- | --- | --- |
| *L. tropica* | ACL | *P. sergenti* | Human |
| *L. major* | ZCL | *P. papatasi* | *Psammomys obesus, Meriones spp.,*  *Nesokia indica* |
| *L. infantum* | ZVL | *P. galilaeus*, *P. syriacus*, *P. tobbi,* *P. halepensis* | *Canis familiaris* |

**MAPS AND TRENDS**

**Visceral leishmaniasis**


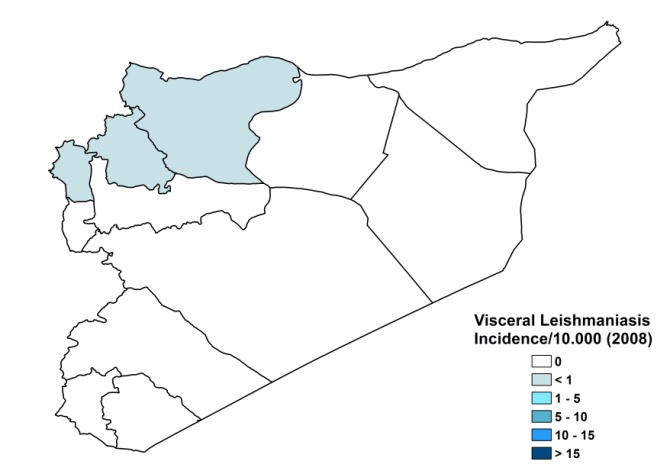

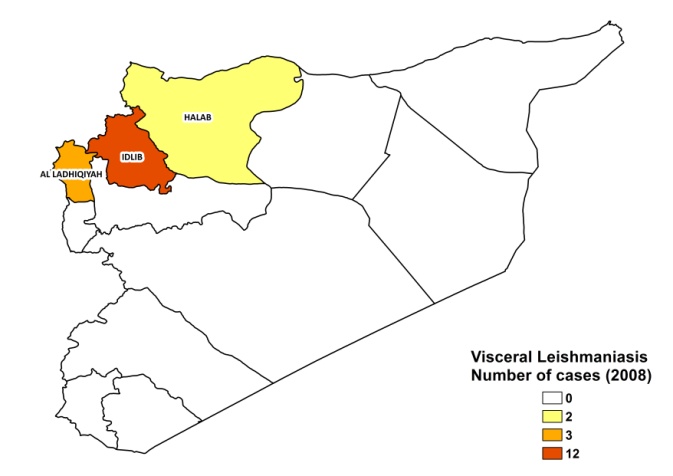


**Cutaneous leishmaniasis**


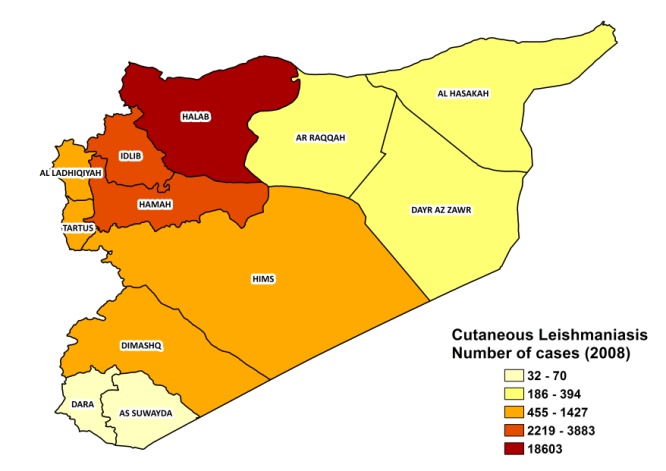

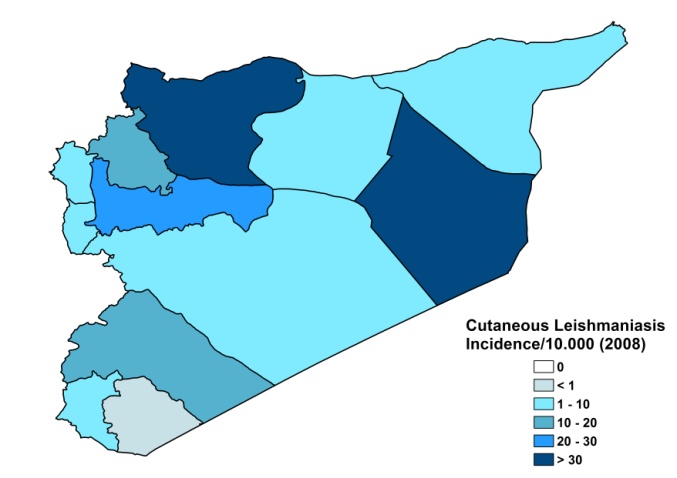


**Visceral leishmaniasis trend**

**Cutaneous leishmaniasis trend**

**CONTROL**

The national control program includes passive case detection in primary health centers (PHCs) and provides diagnosis and treatment free of charge. Active case detection is regularly performed in primary schools of ACL endemic areas. The vector control program consists in insecticide spraying (IRS) in the endemic areas twice a year; however, this does not seem to be effective in controlling the CL incidence. There is a plan to distribute LLINs. For ZCL, there is a reservoir control program, involving regular rodent monitoring and control. This program is updated yearly in order to fit the disease incidence more effectively. A national strategy will be established in the near future. Notification of leishmaniasis is mandatory.

**DIAGNOSIS, TREATMENT**

**Diagnosis**

CL: on clinical grounds, confirmation with microscopic examination of skin lesion sample.

VL: confirmation by microscopic examination of bone marrow aspirate and rapid diagnostic rK39 antigen-based tests.

**Treatment**

CL: antimonials, intralesional (every 6 days) or systemic, 10-20 mg Sb^v^/kg/day. Cure rate is 85-90%, with recurring lesions in 8-10% and rare mucosal involvement.

VL; antimonials, 20 mg Sb^v^/kg/day. Cure rate is 100%

**ACCESS TO CARE**

Medical care is provided for free, which includes diagnosis and treatment of leishmaniasis. CL can be diagnosed and treated in about 250 health centers around the country. Diagnosis for VL is provided in hospitals, and sometimes in health centers, using rapid diagnostic dipsticks. The Ministry of Health provided sufficient antimonials (Glucantime, Sanofi and generic SSG, Albert David, India) for all reported patients in 2007 and 2008. All patients are thought to have access to care. The delay between symptoms and seeking treatment is estimated to be less than 2 months. However, the case load can be very high and the treatment is time intensive. As there are not sufficient health workers qualified to diagnose and treat the disease, treatment delays occur.

**ACCESS TO DRUGS**

No other drugs than antimonials are included in the National Essential Drug List for leishmaniasis. Meglumine antimoniate (Glucantime, Sanofi) and sodium stibogluconate (Pentostam, GSK) are registered for leishmaniasis in Syria. Drugs for leishmaniasis are not available at private pharmacies.

**SOURCES OF INFORMATION**

- Dr Hind Bakour, Ministry of Health.
- Dr Lama Jalouk, Leishmaniasis National Program manager, Ministry of Health. *WHO Consultative meeting on Cutaneous Leishmaniasis in EMRO countries, Geneva, 30 April to 2 May 2007.*

1. [Al-Nahhas S](http://www.ncbi.nlm.nih.gov/pubmed?term=%22Al-Nahhas%20S%22%5BAuthor%5D), [Shabaan M](http://www.ncbi.nlm.nih.gov/pubmed?term=%22Shabaan%20M%22%5BAuthor%5D), [Hammoud L](http://www.ncbi.nlm.nih.gov/pubmed?term=%22Hammoud%20L%22%5BAuthor%5D), [Al-Taweel A](http://www.ncbi.nlm.nih.gov/pubmed?term=%22Al-Taweel%20A%22%5BAuthor%5D), [Al-Jorf S](http://www.ncbi.nlm.nih.gov/pubmed?term=%22Al-Jorf%20S%22%5BAuthor%5D) (2003). Visceral leishmaniasis in the Syrian Arab Republic: early detection using rK39. [East Mediterr Health J.](http://www.ncbi.nlm.nih.gov/pubmed/15748082##) 2003 Jul;9(4):856-62.

2. Desjeux P (1991) Information on the Epidemiology and Control of the Leishmaniases by Country or Territory. Geneva: World Health Organization.

3. [Ashford RW](http://www.ncbi.nlm.nih.gov/pubmed?term=%22Ashford%20RW%22%5BAuthor%5D), [Rioux JA](http://www.ncbi.nlm.nih.gov/pubmed?term=%22Rioux%20JA%22%5BAuthor%5D), [Jalouk L](http://www.ncbi.nlm.nih.gov/pubmed?term=%22Jalouk%20L%22%5BAuthor%5D), [Khiami A](http://www.ncbi.nlm.nih.gov/pubmed?term=%22Khiami%20A%22%5BAuthor%5D), [Dye C](http://www.ncbi.nlm.nih.gov/pubmed?term=%22Dye%20C%22%5BAuthor%5D) (1993). Evidence for a long-term increase in the incidence of Leishmania tropica in Aleppo, Syria. [Trans R Soc Trop Med Hyg](http://www.ncbi.nlm.nih.gov/pubmed/8236380##) 87(3):247-9.

# 4. Tayeh A, Lama J, Cairncross S (1997). Twenty years of cutaneous leishmaniasis in Aleppo, Syria. Trans R Soc Trop Med Hyg 91,657-659.

5. Rioux JA, Dereure J, Khiami A, Pratlong F, Sirdar K, et al (1990). [Ecoepidemiology of leishmaniasis in Syria. 1. Leishmania major Yakimoff and Schokhor (Kinetoplastida-Trypanosomatidae) infestation of Psammomys obesus Cretzschmar (Rodentia-Gerbillidae)].](http://www.ncbi.nlm.nih.gov/pubmed/2097930) Ann Parasitol Hum Comp 65(5-6):203-7.
