# Supplementary material for: Leishmaniasis Worldwide and Global Estimates of Its Incidence
Source: PLoS One. 2012 May 31;7(5):e35671. doi: 10.1371/journal.pone.0035671 (PMC3365071; doi:10.1371/journal.pone.0035671)
Supplement: Text S87 — Leishmaniasis Country Profiles, Taiwan. (DOCX) [file pone.0035671.s087.docx]

**TAIWAN, CHINA**


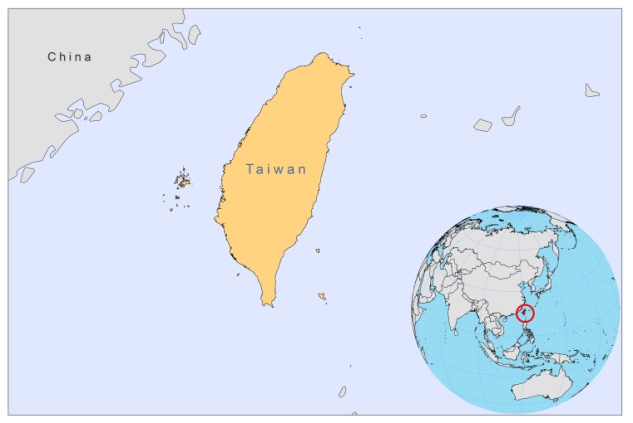


**BASIC DATA**

Total Population: no data

Population 0-14 years: no data

Rural population: no data

Population living under USD 1.25 a day: no data

Population living under the national poverty line: no data

Income status: no data

Ranking: no data

Per capita expenditure on health at average exchange rate (US dollar): no data

Life expectancy at birth (years): no data

Healthy life expectancy at birth (years): no data

**BACKGROUND**

Leishmaniasis appeared in Taiwan, China, for the first time in 1942 [1], but was an imported disease, occurring only in soldiers that spent time on mainland China. Over 100 cases of imported VL and PKDL were documented between 1952 and 1958, due to extensive population movement into Taiwan after the Second World War [2]. The first reports of autochtonous CL were in 2 Taiwanese aboriginals in 1968-1970 [2]. In 2008, a later report followed about a patient with a cutaneous nasal lesion, similar to those caused by *L.tropica;* the causative organism was isolated and concluded to be probable *L.tropica* [3]. In 2009, three more autochtonous cases of CL were reported [4].

**PARASITOLOGICAL INFORMATION**

| ***Leishmania* species** | **Clinical form** | **Vector species** | **Reservoirs** |
| --- | --- | --- | --- |
| Unknown | CL, DCL | *P. kiangsuensis* | Unknown |

**No further information is available**

**SOURCES OF INFORMATION**

1. Hongyo R (1942) Some observations on the blood and urine of kala-azar; especially on Tomita's urine reaction. Taiwan Igakkai Zassi 41: 279-284.

2. Cross JH, Gunning J-J, Drutz DJ, Lien JC (1985). Autochtonous cutaneous-subcutaneous Leishmaniasis on Taiwan. Am. J. Trop. Med. Hyg. 1985; 34(2), 254-256.

3. [Wang JR](http://www.ncbi.nlm.nih.gov/pubmed?term=%22Wang%20JR%22%5BAuthor%5D), [Lee ST](http://www.ncbi.nlm.nih.gov/pubmed?term=%22Lee%20ST%22%5BAuthor%5D), [Juan WH](http://www.ncbi.nlm.nih.gov/pubmed?term=%22Juan%20WH%22%5BAuthor%5D), [Chuang WL](http://www.ncbi.nlm.nih.gov/pubmed?term=%22Chuang%20WL%22%5BAuthor%5D), [Hung SI](http://www.ncbi.nlm.nih.gov/pubmed?term=%22Hung%20SI%22%5BAuthor%5D) et al (2008). Indigenous leishmaniasis in Taiwan: report of a case. [Int J Dermatol](http://www.ncbi.nlm.nih.gov/pubmed/18173599) 47(1):40-3.

4. [Lee JY](http://www.ncbi.nlm.nih.gov/pubmed?term=%22Lee%20JY%22%5BAuthor%5D), [Hsu MM](http://www.ncbi.nlm.nih.gov/pubmed?term=%22Hsu%20MM%22%5BAuthor%5D), [Wang CY](http://www.ncbi.nlm.nih.gov/pubmed?term=%22Wang%20CY%22%5BAuthor%5D), [Ho JC](http://www.ncbi.nlm.nih.gov/pubmed?term=%22Ho%20JC%22%5BAuthor%5D) (2009). Indigenous cutaneous leishmaniasis in Taiwan: three additional cases in southern Taiwan. [Int J Dermatol](http://www.ncbi.nlm.nih.gov/pubmed/19335438) 48(4):441-3.
