# Supplementary material for: Leishmaniasis Worldwide and Global Estimates of Its Incidence
Source: PLoS One. 2012 May 31;7(5):e35671. doi: 10.1371/journal.pone.0035671 (PMC3365071; doi:10.1371/journal.pone.0035671)
Supplement: Text S88 — Leishmaniasis Country Profiles, Tajikistan. (DOCX) [file pone.0035671.s088.docx]

**TAJIKISTAN**


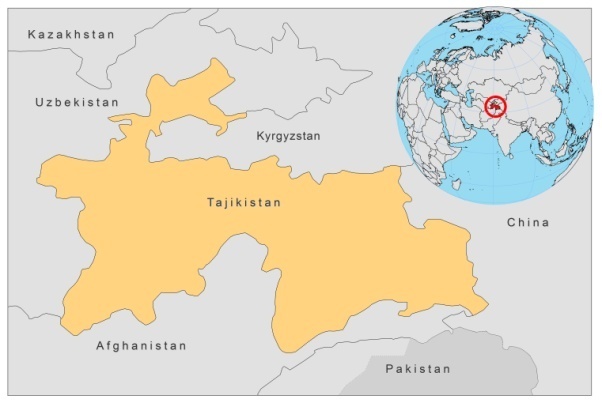


**BASIC COUNTRY DATA**

Total Population: 6,878,637

Population 0-14 years: 37%

Rural population: 74%

Population living under USD 1.25 a day: no data

Population living under the national poverty line: 47.2%

Income status: Low income economy

Ranking: Medium human development (ranking 127)

Per capita total expenditure on health at average exchange rate (US dollar): 38

Life expectancy at birth (years): 67

Healthy life expectancy at birth (years): 55

**BACKGROUND INFORMATION**

Both VL and CL (ZCL and ACL) cases have been reported in the country. Until 1990, the leishmaniasis situation was under control. Only sporadic cases of ZCL and ACL were reported, mainly in areas bordering Afghanistan, in Khatlon region. VL occurred in Panjakent (Sordiy region) and in Darvoz (Gorno Badakhshan autonomic region).

The civil war in 1992 had very negative consequences on leishmaniasis morbidity, due to mass migration of population, lack of vector and reservoir control, and collapse of the health system. Registration of VL cases restarted in 1994 and of CL in 2007.

The disease also spread to new areas. While there used to be only one focus in the south, near the Afghanistan border, the disease was prevalent in 4 regions in 2003-2006 and in 9 regions in 2007-2009. Since 2003, 2 outbreaks of VL and one of CL have occurred, with 42-51 reported cases.

VL as well as CL are underreported. The number of cases of VL is estimated to be 10 times higher than the number reported. For CL, the estimated number of cases was 1,900 in 2008 and 600 in 2007, while only 28 and 13 cases were reported.

There are no cases of HIV/*Leishmania* co-infection reported.

**PARASITOLOGICAL INFORMATION**

| ***Leishmania* species** | **Clinical form** | **Vector species** | **Reservoirs** |
| --- | --- | --- | --- |
| unknown | CL | Unknown | unknown |
| unknown | VL | Unknown | unknown |

**MAPS AND TRENDS**

**Visceral leishmaniasis**


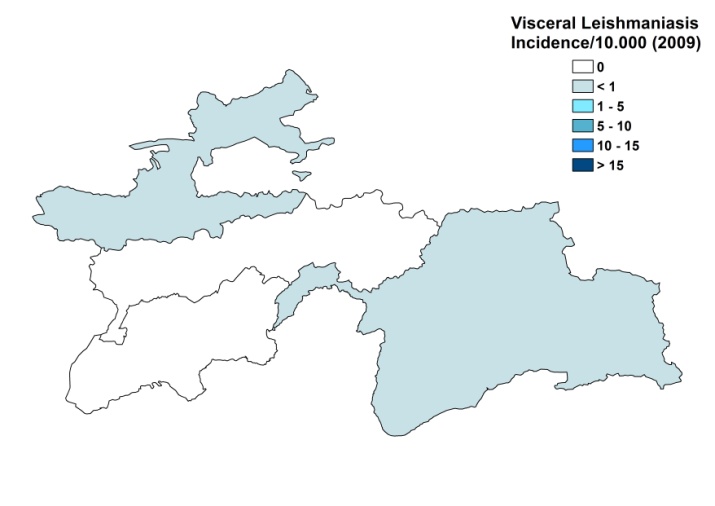

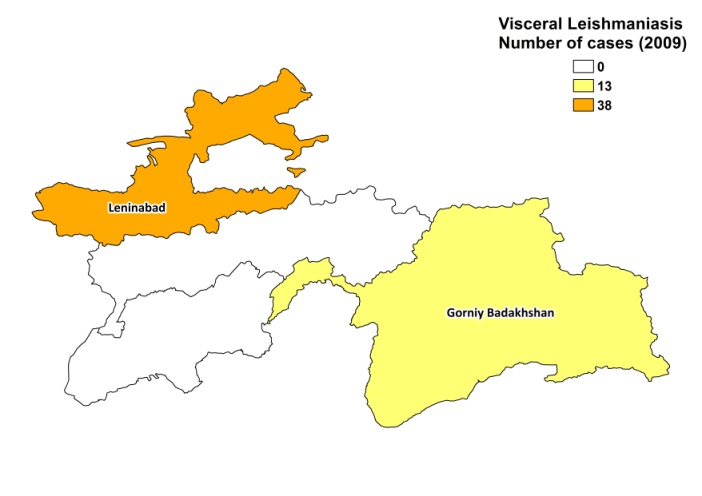


**Cutaneous leishmaniasis**


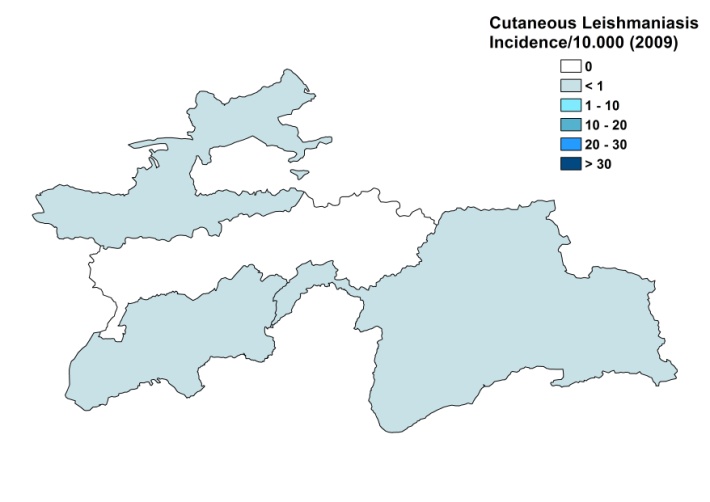

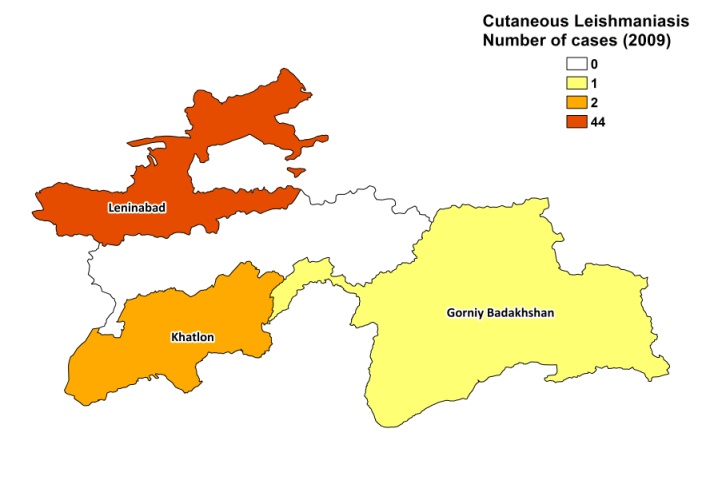


**Visceral leishmaniasis trend**

**Number of cutaneous leishmaniasis**

| **2007** | **2008** |
| --- | --- |
| 13 | 38 |

**CONTROL**

The notification of leishmaniasis is mandatory in the country. There is no national leishmaniasis control program, but it is expected to be established in 2010. There is no leishmaniasis vector control program and no leishmaniasis reservoir control program.

**DIAGNOSIS, TREATMENT**

**Diagnosis**

CL: on clinical grounds

VL: on clinical grounds. Confirmation by microscopic examination of bone marrow aspirate was done in 87% of cases.

**Treatment**

CL: antimonials, 15-20 mg Sb^v^/kg/day for 7 days. Reported cure rate for VL is 95% with 5% relapse. In case of relapse, blood transfusion and antibiotics are given.

VL: antimonials, intralesional or 15-20 mg Sb^v^/kg/day for 7 days. 5% of CL cases heals spontaneously without treatment.

**ACCESS TO CARE**

Care for leishmaniasis is not provided for free. Patients have to pay for the drugs themselves. Possibilities for diagnosis and treatment are very limited. There is no well-equipped laboratory in the country, so samples have to be sent to Uzbekistan. The test costs 30 USD, which needs to be covered by the patient. Patients to not seek treatment in time because a lack of awareness of the disease; therefore, diagnosis is often delayed. The costs of treatment are the main barrier for access. Furthermore, treatment is only given in one specialized hospital and the large distances between endemic foci and the capital make it even more difficult for poor patients to access treatment, as they cannot afford the travel costs and cannot stay away from home for a long time.

**ACCESS TO DRUGS**

Meglumine antimoniate and pentamidine are included in the National Essential Drug List for the treatment of VL and CL. No antimonials are registered. Meglumine antimoniate (Glucantime, Sanofi) is for sale in private pharmacies for 18 USD per 5 ml vial, leading to a treatment cost of around 200 USD for intralesional treatment and around 900 USD for a full treatment of VL.

**SOURCES OF INFORMATION**

- Dr Sherkhonov Tokhir, State Tropical Diseases Center Organization Ministry of Health. *Leishmaniasis in the European Region, a WHO consultative intercountry meeting, Istanbul, Turkey, 17–19 November 2009.*
