# Supplementary material for: Leishmaniasis Worldwide and Global Estimates of Its Incidence
Source: PLoS One. 2012 May 31;7(5):e35671. doi: 10.1371/journal.pone.0035671 (PMC3365071; doi:10.1371/journal.pone.0035671)
Supplement: Text S89 — Leishmaniasis Country Profiles, Thailand. (DOCX) [file pone.0035671.s089.docx]

**THAILAND**

**
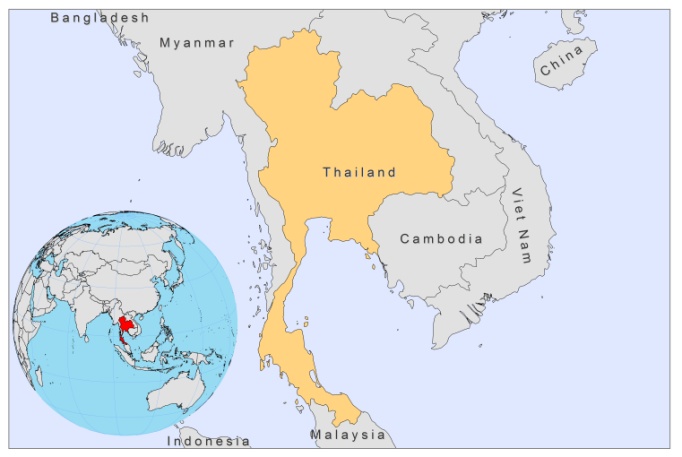
**

**BASIC COUNTRY DATA**

Total Population: 69,122,234

Population 0-14 years: 21%

Rural population: 66%

Population living under USD 1.25 a day: 10.8%

Population living under the national poverty line: 8.1%

Income status: Upper middle income economy

Ranking: Medium human development (ranking 103)

Per capita total expenditure on health at average exchange rate (US dollar): 168

Life expectancy at birth (years): 74

Healthy life expectancy at birth (years): 60

**BACKGROUND INFORMATION**

VL was first notified in 1960; 6 more cases were reported between 1960 and1987. CL was first seen in 1981 and 40 cases were reported until 1997. All these cases were imported, mostly among Thai workers returning from Saudi Arabia. Between 1996 and 2010, the first 13 autochtonous cases (10 VL and 3 CL) were reported from 10 provinces. Most of these cases occurred in the south. Diagnosis was often late and underreporting is suspected, due to a low awareness of leishmaniasis in the country. Three cases were coinfected with HIV, of which two VL cases and one CL case. The trend of HIV spreading to rural areas is a risk factor for an increase of co-infected cases.

**PARASITOLOGICAL INFORMATION**

| ***Leishmania* species** | **Clinical form** | **Vector species** | **Reservoirs** |
| --- | --- | --- | --- |
| Unknown | VL, CL | Unknown | Unknown |

**MAPS AND TRENDS**

**Visceral and cutaneous leishmaniasis**


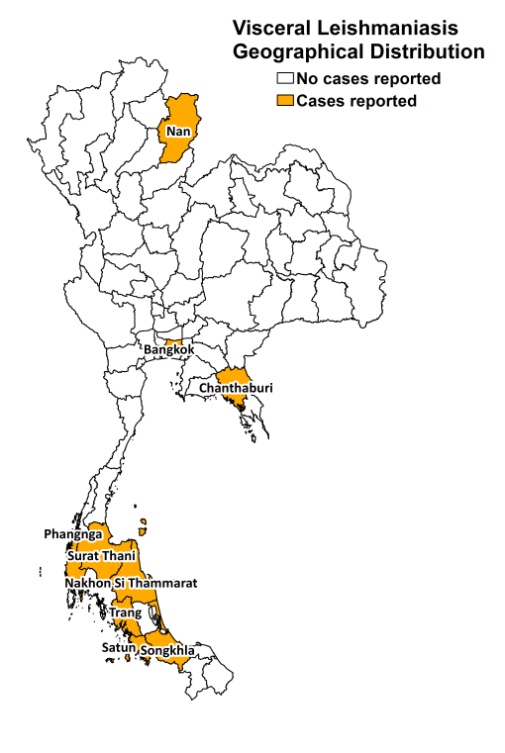

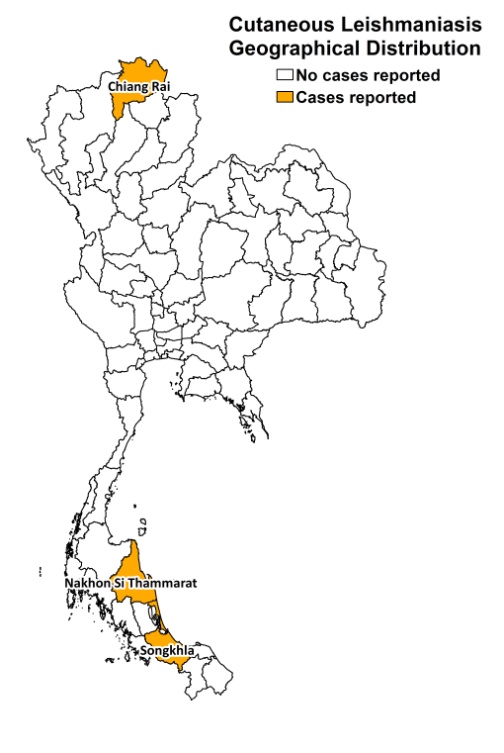


**Trend in visceral leishmaniasis**

**CONTROL**

On-the-spot control measures were introduced, such as IRS, ITN and insect repellent distribution, cleaning-up around houses and raising awareness among population and health workers. The national program has set an elimination target of <1/10,000 population before 2016, with a comprehensive approach involving surveillance, prevention, early diagnosis and treatment with miltefosine, and control measures, including culling, IRS and rehabilitation (counselling) offered to cases

**DIAGNOSIS, TREATMENT**

**Diagnosis**

CL: on clinical picture and confirmation with microscopic examination of skin lesion sample (possible only in specialized hospitals). PCR can be carried out in specialized laboratories.

VL: microscopic evaluation of bone marrow aspirates (possible only in specialized hospitals). PCR can be carried out in specialized laboratories.

**Treatment**

CL and VL: conventional amphotericin B, 1 mg/kg/day. Cure rate for VL is 70%, with 30% recurring infection, and 100% for CL.

**ACCESS TO CARE**

Treatment for CL and VL is provided free of charge. All patients are thought to have access to care, however, diagnosis often takes places very late (up to one year after onset of symptoms as health personnel have little experience and knowledge of leishmaniasis). Often, CL is wrongly diagnosed at first and treated with antibiotics.

**ACCESS TO DRUGS**

Sodium stibogluconate and amphotericin B are included in the National Essential Drug List for leishmaniasis.

**SOURCES OF INFORMATION**

- Dr Kobkan Kanjanopas. Bureau of Vector Borne Disease Department, Ministry of Public Health. *WHO informal consultation on epidemiological information on disease burden due to kala-azar in Bangladesh, India and Nepal. Paro, Bhutan, 8-10 March 2011.*
