# Supplementary material for: Leishmaniasis Worldwide and Global Estimates of Its Incidence
Source: PLoS One. 2012 May 31;7(5):e35671. doi: 10.1371/journal.pone.0035671 (PMC3365071; doi:10.1371/journal.pone.0035671)
Supplement: Text S90 — Leishmaniasis Country Profiles, The Former Yugoslav Republic of Macedonia. (DOCX) [file pone.0035671.s090.docx]

**THE FORMER YUGOSLAV REPUBLIC OF MACEDONIA**


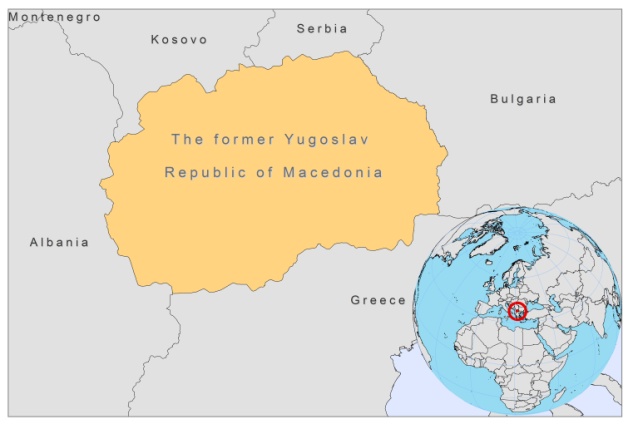


**BASIC COUNTRY DATA**

Total Population: 2,060,563

Population 0-14 years: 18%

Rural population: 32%

Population living under USD 1.25 a day: 0.3%

Population living under the national poverty line: 19%

Income status: Upper middle income economy

Ranking: High human development (ranking 78)

Per capita total expenditure on health at average exchange rate (US dollar): 314

Life expectancy at birth (years): 74

Healthy life expectancy at birth (years): 63

**BACKGROUND INFORMATION**

The Former Yugoslav Republic of Macedonia is endemic for VL. Between 1924 and 1948, nearly all cases were seen in children and infants. This pattern changed over time: between 1996 and 2009, 85 cases were diagnosed, with 50% coming from the central part of the country; 85% of these cases were males, 52% of these cases were between 20 and 40 years old, and none of them occurred in children. In a survey held in 2005, 13% of dogs were found to be infected.

CL is not prevalent in the country.

There are no cases of HIV/*Leishmania* co-infection.

**PARASITOLOGICAL INFORMATION**

| ***Leishmania* species** | **Clinical form** | **Vector species** | **Reservoirs** |
| --- | --- | --- | --- |
| *L. infantum* | ZVL, CL | unknown | *Canis familiaris* |

**MAPS AND TRENDS**


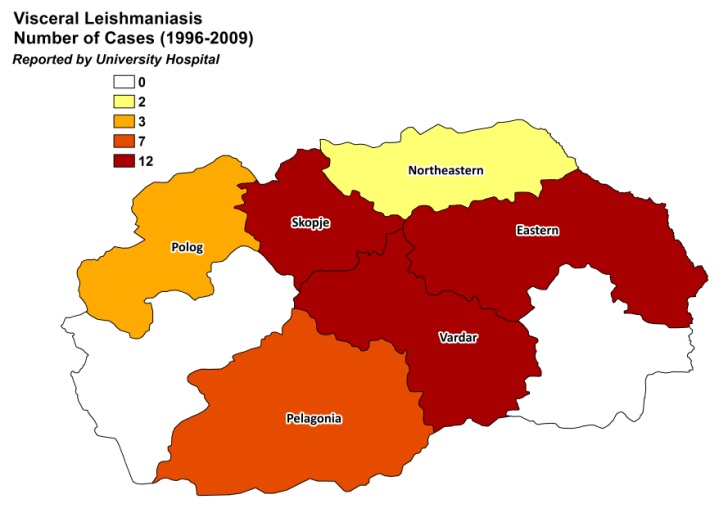

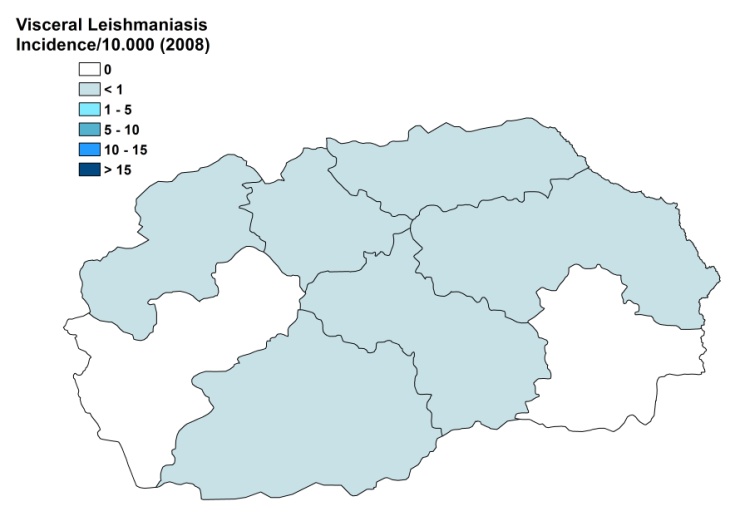
**Visceral leishmaniasis**

**Visceral leishmaniais trend**

**CONTROL**

The notification of leishmaniasis is mandatory in the country. There is no national leishmaniasis control program, nor a leishmaniasis vector control program. However, there is a leishmaniasis reservoir control program. Serological surveys of dogs are regularly performed and it is recommended that positive dogs are sacrificed.

**DIAGNOSIS, TREATMENT**

**Diagnosis**

VL: IFAT, DAT, and microscopic examination of bone marrow aspirate.

**Treatment**

**VL:** antimonials 20 mg Sb^v^/kg/day for 28 days. Cure rate is 98%, and mortality rate 2%. Second line treatment is with amphotericin B, 0.5 mg/kg/day for 20 days.

**ACCESS TO CARE**

Care for leishmaniasis is provided for free, but sometimes health workers charge informal fees to patients. All patients are thought to have access to care.

The government did not purchase antimonials in 2007 and 2008, but donations of a private agent of Glucantime, Sanofi, were sufficient to treat all patients those years. The average time until diagnosis is one month in 60% of patients, but in 40% of cases it took longer, even up to 7-8 months for 3 patients. Diagnosis is only possible in secondary and tertiary hospitals.

**ACCESS TO DRUGS**

Meglumine antimoniate is included in the National Essential Drug List. Antimonials are not registered and drugs for leishmaniasis are not available at private pharmacies.

**SOURCES OF INFORMATION**

• Dr Sarja Caparoska. University Hospital for Infectious Diseases and Febrile Conditions, Medical Faculty, University "St. Cyril and Methodious", Skopje.

• Dr Žaklina Sopova*.* University Hospital for Infectious Diseases and Febrile Conditions, Medical Faculty, University "St. Cyril and Methodious", Skopje. *WHO exploratory meeting on Leishmaniasis in the Balkan Countries. Dubrovnik, Croatia, 10-12 February 2010.*
