# Supplementary material for: Leishmaniasis Worldwide and Global Estimates of Its Incidence
Source: PLoS One. 2012 May 31;7(5):e35671. doi: 10.1371/journal.pone.0035671 (PMC3365071; doi:10.1371/journal.pone.0035671)
Supplement: Text S91 — Leishmaniasis Country Profiles, Tunisia. (DOCX) [file pone.0035671.s091.docx]

**TUNISIA**


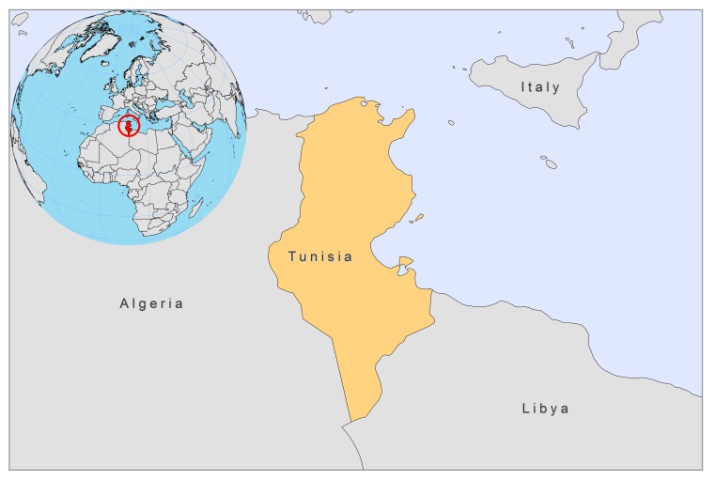


**BASIC COUNTRY DATA**

Total Population: 10,549,100

Population 0-14 years: 23%

Rural population: 33%

Population living under USD 1.25 a day: 2.6%

Population living under the national poverty line: no data

Income status: Upper middle income economy

Ranking: High human development (ranking 94)

Per capita total expenditure on health at average exchange rate (US dollar): 240

Life expectancy at birth (years): 74

Healthy life expectancy at birth (years): 62

**BACKGROUND INFORMATION**

VL was endemic in the north of the country, but the number of cases decreased importantly during the antimalarial (DDT) spraying campaign in the 70s (1968-1974). Currently, cases are however on the increase; around 100 cases/year were reported between 1996-2006, probably linked to irrigation development and an agriculture that is favorable to the multiplication of vector sandflies and dogs, reservoirs of *L. infantum* [1]. Patients are mostly children (80%) from 1 to 5 years old [2].

CL caused by *L.major* is a major public health problem. It occurs mainly in central and southwestern Tunisia (semi-arid and arid areas) with thousands of cases [3]. There are foci with a permanent active transmission, so, from time to time, outbreaks occur, related to new agricultural projects or large population movements (introduction to a non-immune population) [4]. In some villages, up to 60% of the population is infected.

Less frequent, CL caused by *L.tropica* occurs mostly in southeastern Tunisia (in the Tataouine region, where a small focus was discovered in 1980).

Sporadic CL due to *L. infantum* occurs in towns and villages in the north of the country. Sporadic CL due to *L. killicki* occurs further south, sometimes in small outbreaks [5].

In 2005, over 15,000 cases of CL were reported. Since then, the incidence of both VL and CL has been decreasing to all time low numbers. Both VL and CL are probably underreported.

There are no cases of HIV/*Leishmania* co-infection reported.

**PARASITOLOGICAL INFORMATION**

| ***Leishmania* species** | **Clinical form** | **Vector species** | **Reservoirs** |
| --- | --- | --- | --- |
| *L. infantum* | ZVL, CL | *P. langeroni, P. pemiciosus,*  *P. perfiliewi, P. longicuspis* | *Canis familiaris* |
| *L. major* | ZCL | *P. papatasi* | *Psammomys obesus*, *Meriones spp.* |
| *L. killicki* | CL | unknown |  |

**MAPS AND TRENDS**

**Visceral leishmaniais**

**
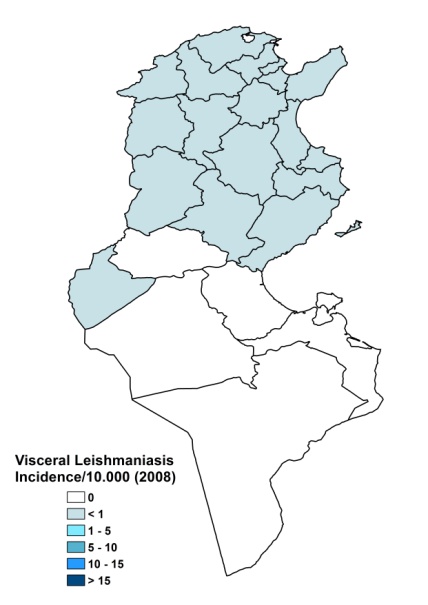

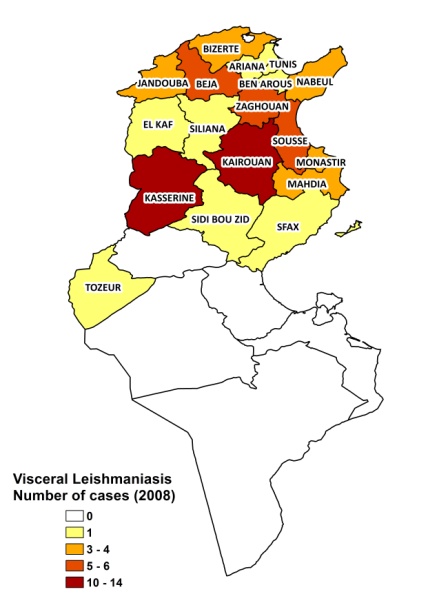
**

**Cutaneous leishmaniasis**


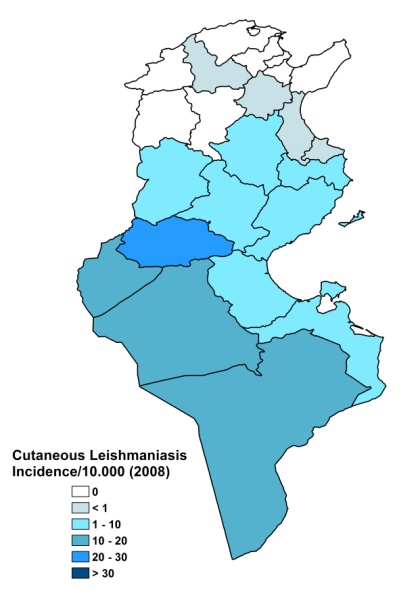

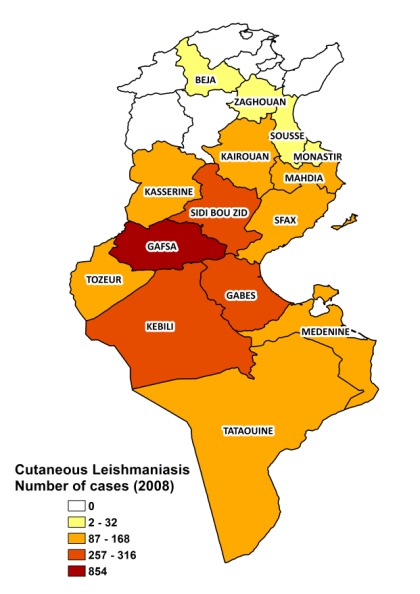


**Visceral leishmaniasis trend**

**Cutaneous leishmaniasis trend**

**CONTROL**

The notification of CL and VL is mandatory. Leishmaniasis control is included in the national health program. Case detection is passive. There is no vector control program, but there is a reservoir control program, with a regular control of dog and rodent populations.

**DIAGNOSIS, TREATMENT**

**Diagnosis:**

CL: on clinical grounds, confirmation with microscopic examination of skin lesion sample.

VL: confirmation by microscopic examination of bone marrow or spleen aspirate.

**Treatment:**

VL: antimonials, 20 mg Sb^v^/kg/day for 21-28 days. Cure rate is ~95%.

CL: antimonials, intralesional or systemic, 10-20 mg Sb^v^/kg/day for 10-15 days.

**ACCESS TO CARE**

Medical care for leishmaniasis is not provided for free. Patients are charged a 2.5 USD registration fee. CL, as well as VL, can be diagnosed and treated at primary health care level. The Ministry of Health provided sufficient antimonials (Glucantime, Sanofi) for all reported patients in 2007 and 2008. All patients are thought to have access to care in Tunisia. About 5% of VL patients and 2% of CL patients seek private care for diagnosis, but are referred to the public sector for treatment.

**ACCESS TO DRUGS**

No other drugs than meglumine antimoniate are included in the National Essential Drug List for leishmaniasis. Drugs for leishmaniasis are not available at private pharmacies. Glucantime (Sanofi) is the only drug for leishmaniasis that is registered in Tunisia.

**SOURCES OF INFORMATION**

- Dr Mondher Bejaoui and Melle Meriem Sekrafi. National Programme for Leishmaniasis, Direction des Primary Health Care, Ministère de la Santé publique. *WHO Consultative meeting on Cutaneous Leishmaniasis in EMRO countries, Geneva, 30 April to 2 May 2007.*

1. [Aoun K](http://www.ncbi.nlm.nih.gov/pubmed?term=%22Aoun%20K%22%5BAuthor%5D), [Jeddi F](http://www.ncbi.nlm.nih.gov/pubmed?term=%22Jeddi%20F%22%5BAuthor%5D), [Amri F](http://www.ncbi.nlm.nih.gov/pubmed?term=%22Amri%20F%22%5BAuthor%5D), [Ghrab J](http://www.ncbi.nlm.nih.gov/pubmed?term=%22Ghrab%20J%22%5BAuthor%5D), [Bouratbine A](http://www.ncbi.nlm.nih.gov/pubmed?term=%22Bouratbine%20A%22%5BAuthor%5D) (2009). Current epidemiological data on visceral leishmaniasis in Tunisia. [Med Mal Infect](javascript:AL_get(this,%20'jour',%20'Med%20Mal%20Infect.');) 39(10):775-9.

2. Pousse H, Besbes A, Ben Said M, Ghenimi L, Kharrat H (1995). Epidemiology of human visceral leishmaniasis in Tunisia. J Trop Pediatr 41: 191–192.

3. [Chaffai M](http://www.ncbi.nlm.nih.gov/pubmed?term=%22Chaffai%20M%22%5BAuthor%5D), [Ben Rachid MS](http://www.ncbi.nlm.nih.gov/pubmed?term=%22Ben%20Rachid%20MS%22%5BAuthor%5D), [Ben-Ismail R](http://www.ncbi.nlm.nih.gov/pubmed?term=%22Ben-Ismail%20R%22%5BAuthor%5D), [Ben Osman A](http://www.ncbi.nlm.nih.gov/pubmed?term=%22Ben%20Osman%20A%22%5BAuthor%5D), [Makni N](http://www.ncbi.nlm.nih.gov/pubmed?term=%22Makni%20N%22%5BAuthor%5D) (1988). Clinico-epidemiologic forms of cutaneous leishmaniasis in Tunisia. [Ann Dermatol Venereol.](javascript:AL_get(this,%20'jour',%20'Ann%20Dermatol%20Venereol.');) 115(12):1255-60.

4. Kamarianakis, Prastacos P, Salah AB, Schlif S, Alaya NB (2007). Risk maps for Leishmaniasis in Central Tunisia. 10th AGILE International Conference on Geographic Information Science 2007. Aalborg University, Denmark.

**5. Maubon D,** **Thurot-Guillou C, Ravel C, Leccia M, and Pelloux H (2009).** Leishmania killicki Imported from Tunisian Desert. Emerg Infect Dis 15 (11) (letter).
