# Supplementary material for: Leishmaniasis Worldwide and Global Estimates of Its Incidence
Source: PLoS One. 2012 May 31;7(5):e35671. doi: 10.1371/journal.pone.0035671 (PMC3365071; doi:10.1371/journal.pone.0035671)
Supplement: Text S92 — Leishmaniasis Country Profiles, Turkey. (DOCX) [file pone.0035671.s092.docx]

**TURKEY**


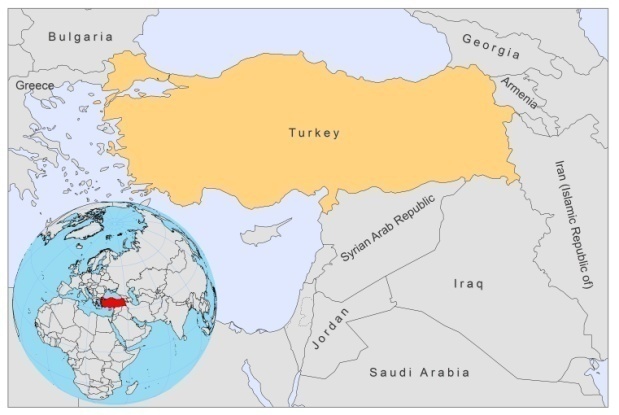


**BASIC COUNTRY DATA**

Total Population: 72,752,325

Population 0-14 years: 26%

Rural population: 30%

Population living under USD 1.25 a day: 2.7%

Population living under the national poverty line: 18.1%

Income status: Upper middle income economy

Ranking: High human development (ranking 92)

Per capita total expenditure on health at average exchange rate (US dollar): 571

Life expectancy at birth (years): 73

Healthy life expectancy at birth (years): 62

**BACKGROUND INFORMATION**

The first case of VL in Turkey was reported from Trabzon, in the eastern part of the Black Sea Region, in 1916; the second from Izmir, in the Aegean Region, in 1918. VL is endemic, with sporadic cases reported from 38 of 81 provinces (in 2008, there were less than 10 cases). Most cases occur at the Armenian border, in the Aegean, Mediterranean, and Central Anatolia Regions [1,2]. Dogs seem to be the main animal reservoir with a high seroprevalence of over 20% in some of the endemic regions [3].

CL is caused by *L. tropica* and is more prevalent in southeastern Anatolia [4], where 96% of cases are located, central Anatolia, the western regions, and, less frequently, in the Mediterranean and Aegean Regions [1,5,6]. In Sanliurfa, southeastern Anatolia, the number of CL cases reported from 1981 to 2000 was 22,335. The reported incidence reached a peak in 1994, with 4,185 cases. There has been a considerable reduction in the number of reported cases from 1995 onwards [7]. A recent upsurge of CL cases in the southeastern Anatolia Region is attributed to the environmental impact of a big irrigation project (Güneydoğu Anadolu Projesi-GAP) [8]. Some CL cases are attributed to *L. infantum* in the eastern Mediterranean region.

VL and CL are both suspected to be underreported, due to a lack of awareness of the disease among physicians.

No HIV/*Leishmania* co-infection has yet been reported.

**PARASITOLOGICAL INFORMATION**

| ***Leishmania* species** | **Clinical form** | **Vector species** | **Reservoirs** |
| --- | --- | --- | --- |
| *L infantum* | ZVL, CL | *P. neglectus*, *P. syriacus*, *P. tobbi, P. alexandri* | *Canis familiaris* |
| *L tropica* | ACL | *P. sergenti* | Human |

**MAPS AND TRENDS**

**Visceral leishmaniasis**


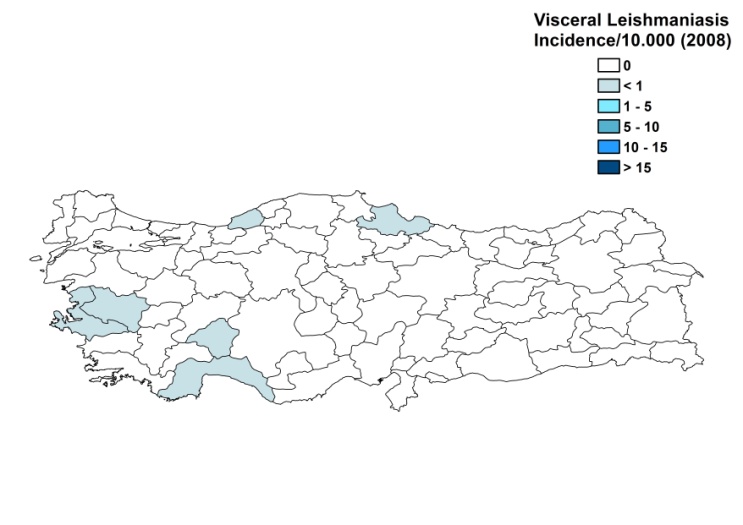

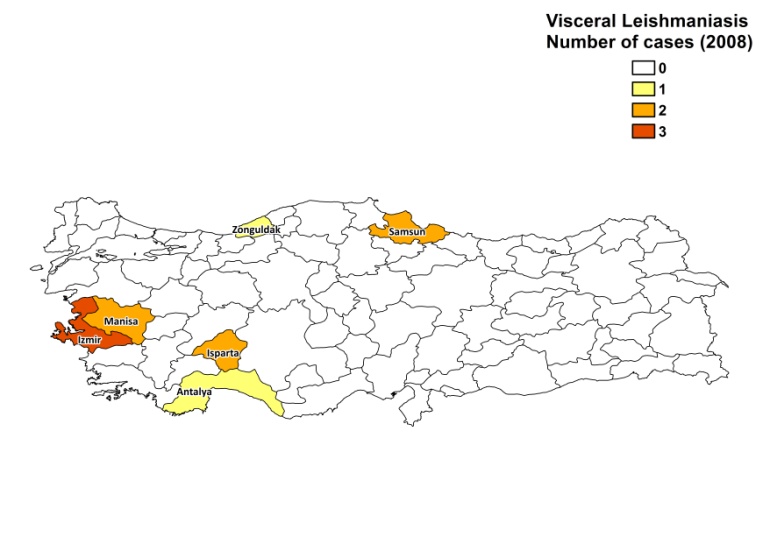


**Cutaneous leishmaniasis**


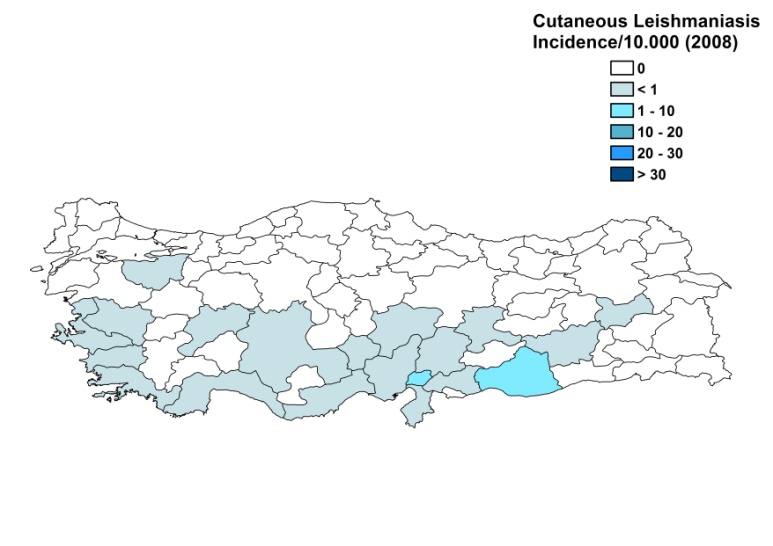

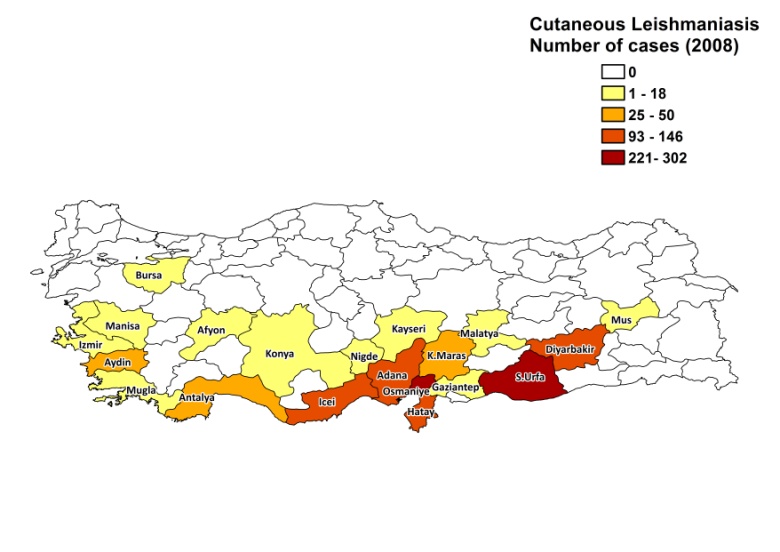


**Visceral leishmaniasis trend**

**
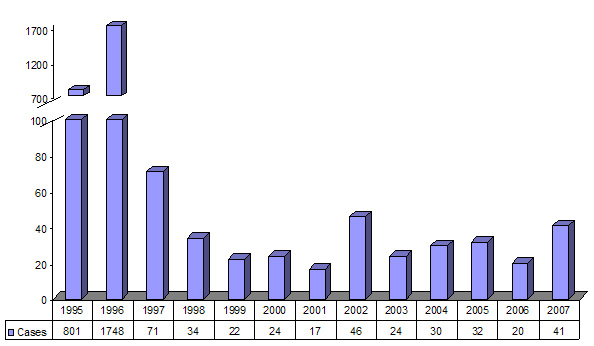
**

**Cutaneous leishmaniasis trend**

**CONTROL**

The notification of leishmaniasis is mandatory for CL and VL. A national leishmaniasis control program has been in place since 1996. There is no leishmaniasis vector control program, but vector control strategies are integrated into a malaria control program and include use of insecticides and promotion of the use of regular small mesh and impregnated bednets. There is no leishmaniasis reservoir control program. Dogs are not checked for infection and there is no program for sacrifice of positive dogs.

**DIAGNOSIS, TREATMENT**

**Diagnosis**

VL: antigen-based rapid diagnostic tests (rK39), IFAT, ELISA. PCR is used in reference centers.

CL: confirmation with microscopic examination of skin lesion sample. In specialized hospitals, cultures are done.

**Treatment**

VL: antimonials, 20 mg Sb^v^/kg/day for 30 days. Cure rate is around 95%. Second line treatment is with liposomal amphotericin B.

CL: antimonials, 20 mg Sb^v^/kg/day for 28 days. Second line treatment is amphotericin B.

**ACCESS TO CARE**

Care for leishmaniasis is provided for free. Diagnosis and treatment for VL is only possible in secondary and tertiary hospitals. CL can be diagnosed and treated at health center level. The Ministry of Health purchased sufficient antimonials (Glucantime, Sanofi, and Pentostam, GSK) to treat all patients in 2007 and 2008. All patients are thought to have access to care.

**ACCESS TO DRUGS**

Meglumine antimoniate and sodium stibogluconate are included in the National Essential Drug List for leishmaniasis. Sodium stibogluconate (Pentostam, GSK) is registered in Turkey, but not available in private pharmacies. Liposomal amphotericin B (AmBisome, Gilead) is available in private pharmacies for 228 USD per vial.

**SOURCES OF INFORMATION**

- Dr Yusuf Özbel, University Medical School, Department of Parasitology, Izmir. *Leishmaniasis in the European Region, a WHO consultative intercountry meeting, Istanbul, Turkey, 17–19 November 2009.*

1. Özbel Y, Turgay N, Ozensoy S, Ozbilgin A, Alkan MZ et al (1995). [Epidemiology, diagnosis and control of leishmaniasis in the Mediterranean region.](http://www.ncbi.nlm.nih.gov/pubmed/8745931) Ann Trop Med Parasitol 89 Suppl 1:89-93.

2. Ozensoy S, Özbel Y, Turgay N, Alkan MZ, Gul K et al (1998). [Serodiagnosis and epidemiology of visceral leishmaniasis in Turkey.](http://www.ncbi.nlm.nih.gov/pubmed/9749626) Am J Trop Med Hyg 59(3):363-9.

3. Marsella R, de Gopegui RR (1998). Leishmaniasis: a re-emerging zoonozis. Int. J. Dermatol **37:** 801–814.

4. [Gramiccia M](http://www.ncbi.nlm.nih.gov/pubmed?term=%22Gramiccia%20M%22%5BAuthor%5D), [Bettini S](http://www.ncbi.nlm.nih.gov/pubmed?term=%22Bettini%20S%22%5BAuthor%5D), [Yasarol S](http://www.ncbi.nlm.nih.gov/pubmed?term=%22Yasarol%20S%22%5BAuthor%5D) (1984). Isoenzyme characterization of Leishmania isolates from human cases of cutaneous leishmaniasis in Urfa, south-east Turkey. [Trans R Soc Trop Med Hyg](http://www.ncbi.nlm.nih.gov/pubmed?term=Gramiccia%2C%20leishmaniasis%2C%20turkey##) 78(4):568.

5. Uzun S, Uslular C, Yücel A, Acar MA, Ozpoyraz M et al (1999). [Cutaneous leishmaniasis: evaluation of 3,074 cases in the Cukurova region of Turkey.](http://www.ncbi.nlm.nih.gov/pubmed/10233236) Br J Dermatol 140(2):347-50.

6. Demirel R, Erdoğan S (2009). Determination of High Risk Regions of Cutaneous Leishmaniasis in Turkey Using Spatial Analysis Türkiye Parazitoloji Dergisi 33 (1): 8 - 14.

7. Zeyrek FY, Korkmaz M, Özbel Y (2007). [Serodiagnosis of anthroponotic cutaneous leishmaniasis (ACL) caused by Leishmania tropica in Sanliurfa Province, Turkey, where ACL Is highly endemic.](http://www.ncbi.nlm.nih.gov/pubmed/17761525) Clin Vaccine Immunol 14(11):1409-15.

8. [Ok UZ](http://www.ncbi.nlm.nih.gov/pubmed?term=%22Ok%20UZ%22%5BAuthor%5D), [Balcioğlu IC](http://www.ncbi.nlm.nih.gov/pubmed?term=%22Balcio%C4%9Flu%20IC%22%5BAuthor%5D), [Taylan Özkan A](http://www.ncbi.nlm.nih.gov/pubmed?term=%22Taylan%20Ozkan%20A%22%5BAuthor%5D), [Ozensoy S](http://www.ncbi.nlm.nih.gov/pubmed?term=%22Ozensoy%20S%22%5BAuthor%5D), [Özbel Y](http://www.ncbi.nlm.nih.gov/pubmed?term=%22Ozbel%20Y%22%5BAuthor%5D) (2002). Leishmaniasis in Turkey. [Acta Trop](http://www.ncbi.nlm.nih.gov/pubmed/12387909##) 84(1):43-8.
