# Supplementary material for: Leishmaniasis Worldwide and Global Estimates of Its Incidence
Source: PLoS One. 2012 May 31;7(5):e35671. doi: 10.1371/journal.pone.0035671 (PMC3365071; doi:10.1371/journal.pone.0035671)
Supplement: Text S93 — Leishmaniasis Country Profiles, Turkmenistan. (DOCX) [file pone.0035671.s093.docx]

**TURKMENISTAN**


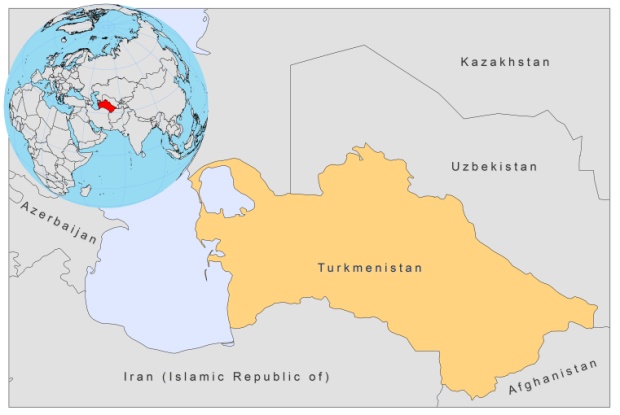


**BASIC COUNTRY DATA**

Total Population: 5,041,995

Population 0-14 years: 29%

Rural population: 51%

Population living under USD 1.25 a day: no data

Population living under the national poverty line: no data

Income status: Lower middle income economy

Ranking: Medium human development (ranking 102)

Per capita total expenditure on health at average exchange rate (US dollar): 77

Life expectancy at birth (years): 65

Healthy life expectancy at birth (years): 54

**BACKGROUND INFORMATION**

Over the last 9 years (2000-2009), 1,562 cases of CL have been reported, mostly in the southern and eastern parts of the country, bordering Iran and Afghanistan. 34% of these cases were found in children under 14 years of age. Most patients are male, field workers and school children (WHO internal report, 2009). Underreporting may happen, as no active case detection is done.

VL is not reported, but is likely to be prevalent. The last VL case was notified in 2000. 79 VL cases were reported in the period of 1981-1987 (WHO internal report, 2009). Sero-epidemiological studies of children in this period found antibodies in 10-11% [1].

No HIV/*Leishmania* co-infection has been reported.

**PARASITOLOGICAL INFORMATION**

| ***Leishmania* species** | **Clinical form** | **Vector species** | **Reservoirs** |
| --- | --- | --- | --- |
| *L. major* | ZCL | *P. papatasi* | *Rhombomys opimus* |
| *L. tropica* | CL | *P. sergenti* |  |
| *L. infantum* | ZVL | Unknown | *Canis familiaris* |

**MAPS AND TRENDS**

**Cutaneous leishmaniasis**

**
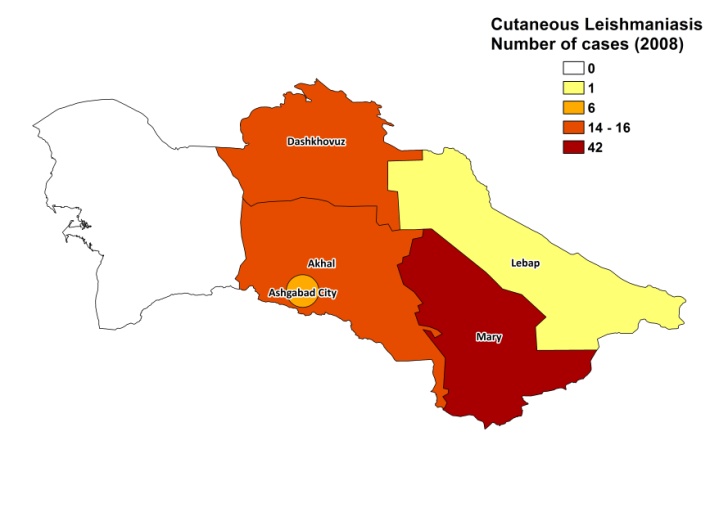

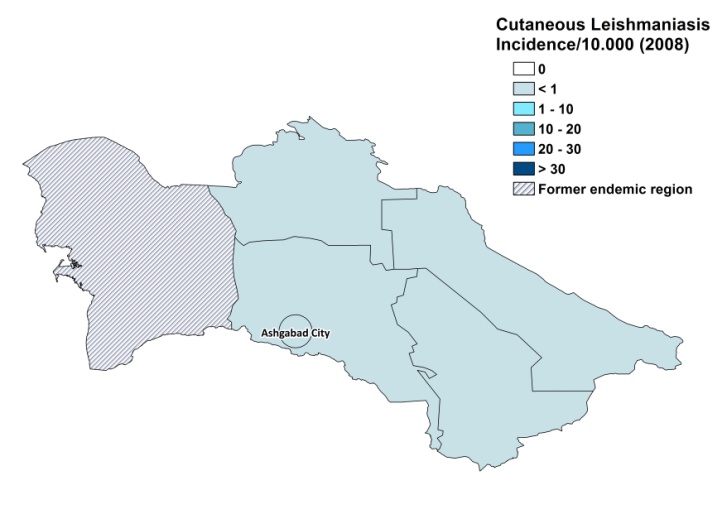
**

**Cutaneous leishmaniasis trend**

**CONTROL**

The notification of leishmaniasis is not mandatory in the country and there is no national leishmaniasis control program, but awareness and training of health personnel in the ZCL foci are maintained. There is no leishmaniasis vector control program, but indoor insecticide spraying is done and bednets are distributed for the malaria control program. There is no leishmaniasis reservoir control program, but rodent control in urban and periurban areas, by poison and destruction of holes, takes place in ZCL foci.

**DIAGNOSIS, TREATMENT**

**Diagnosis**

CL: on clinical grounds and sometimes confirmation with microscopic examination of skin lesion sample.

VL: confirmation by microscopic examination of bone marrow aspirate.

**Treatment**

CL: takes place mostly at local level, with nonspecific methods (antibiotics, traditional medicine, etc.). In complicated cases: antimonials.

VL: antimonials. There are no National Guidelines for treatment.

**ACCESS TO CARE**

Diagnostics and treatment of CL is possible at local level in the endemic zones. However, there are few trained lab technicians and some patients have to go to the regional centers and Ashgabat city for diagnosis and treatment.

**ACCESS TO DRUGS**

No antimonials are registered in Turkmenistan. Antimonials must be obtained unofficially by patients themselves.

**SOURCES OF INFORMATION**

- Drs Shavkat Razakov and Dmitriy Kovalenko. Leishmaniasis in Turkmenistan. *Rapid assessment for leishmaniasis control. WHO internal report. May 2009*.

1. Mizgireva MF, Ponirovsky EN, Sabitov EA (1984). The Application of Immunofluorescent Reaction in Visceral Leishmaniasis Diagnostics in Turkmenian Soviet Socialist Republic. Medical Parasitology and Parasitic Diseases 3:78-81.
