# Supplementary material for: Leishmaniasis Worldwide and Global Estimates of Its Incidence
Source: PLoS One. 2012 May 31;7(5):e35671. doi: 10.1371/journal.pone.0035671 (PMC3365071; doi:10.1371/journal.pone.0035671)
Supplement: Text S94 — Leishmaniasis Country Profiles, Uganda. (DOCX) [file pone.0035671.s094.docx]

**UGANDA**


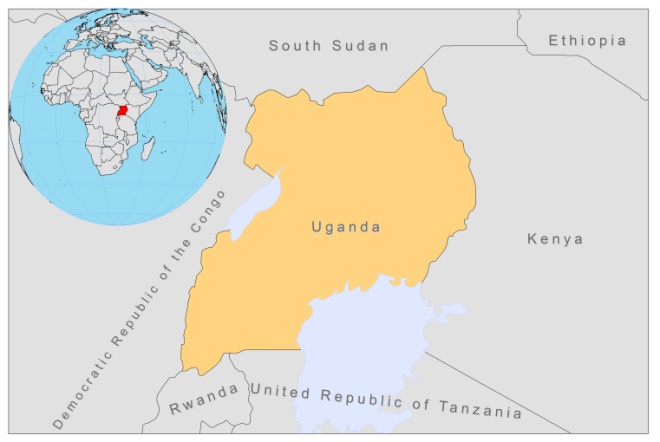


**BASIC COUNTRY DATA**

Total Population: 33,424,683

Population 0-14 years: 48%

Rural population: 87%

Population living under USD 1.25 a day: 28.7%

Population living under the national poverty line: 24.5%

Income status: Low income economy

Ranking: Low human development (ranking 161)

Per capita total expenditure on health at average exchange rate (US dollar): 43

Life expectancy at birth (years): 53

Healthy life expectancy at birth (years): 43

**BACKGROUND INFORMATION**

The first case of VL was recorded in 1946. In 1959, VL appeared to have become endemic in the Karamoja Sub region, Amudat district, an area of semi-arid steppe (northeastern Uganda). Recently, new cases have been reported from 2 neighboring districts: Moroto and Kotido (Amudat hospital report, MoH, 2010).

The total burden of the disease and its geographical spread remain unknown, due to the lack of a systematic surveillance system [1]. Most VL cases are among nomads in Amudat region, and the male:female ratio is 3:1. The only hospital where VL can be diagnosed and treated is in this region. It was operated by MSF until 2006, when MSF left the hospital and opened a VL project across the border in Kenya. As a consequence, VL was not diagnosed and treated in Uganda that year and patients sought treatment over the border in Kenya. In 2007, the hospital became functional again. A survey, conducted in 2007, identified risk factors for contracting VL, including sitting on termite mounts, treating livestock with insecticides and having a low socio-economic status. Owning a mosquito bednet was associated with a reduced risk of contracting the disease [2].

CL was reported for several years (1926-1936). Today, it probably occurs in the foothills of Mount Eigon, but this needs confirmation. The disease is caused by *L. aethiopica* (Mulago hospital report; MOH). A case of CL due to *L.donovani,* in an HIV co-infected patient living in the Ssese Islands in Lake Victoria, was reported recently [3].

2 cases of HIV/*Leishmania* co-infection occurred in the last 2 years.

**PARASITOLOGICAL INFORMATION**

| ***Leishmania* species** | **Clinical form** | **Vector species** | **Reservoirs** |
| --- | --- | --- | --- |
| *L. donovani* | VL, CL | *P. martini* |  |

**MAPS AND TRENDS**

**Visceral leishmaniasis**


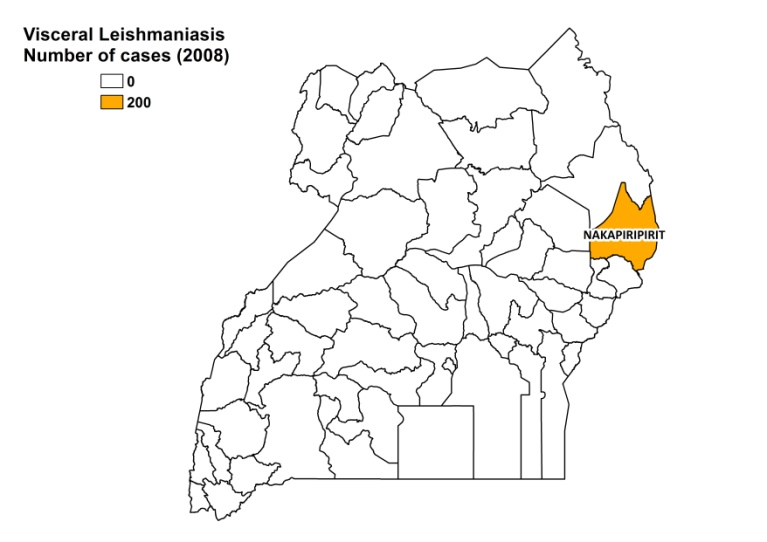


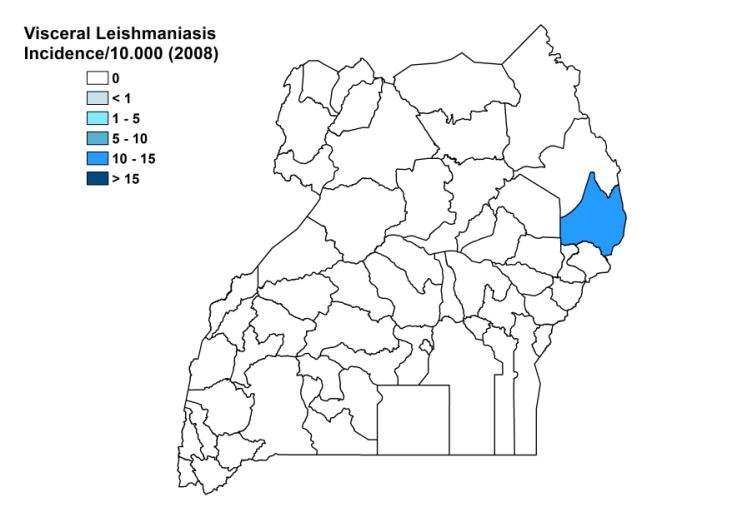


**Visceral leishmaniasis trend**

**CONTROL**

The notification of leishmaniasis is not mandatory in the country. A national leishmaniasis control program has been in place since 1999 and recently, the Ministry of Health included leishmaniasis in the NTDs' strategy, with the National Master Plan for Neglected Tropical Diseases Program, 2011-2015. There is no leishmaniasis vector control program. Bednet distribution is done in the context of malaria control.

**DIAGNOSIS, TREATMENT & OUTCOMES**

**Diagnosis:**

VL: confirmation by rK 39 antigen-based rapid test and, if needed, by microscopic examination of spleen aspirate.

**Treatment:**

VL: antimonials, 20 mg Sb^v^/kg/day for 30 days. Cure rate is 100% with 0.46% of PKDL and 2% severe adverse effects. Overall case fatality rate was 1.4%. Second line treatment is with liposomal amphotericin B, 3 mg/kg/day for 6 days.

**ACCESS TO CARE**

Care for leishmaniasis is provided for free. There is only one hospital in Uganda where VL can be diagnosed and treated (Amudat hospital). Drugs for this hospital are provided by DNDi (Drugs for Neglected Diseases initiative), as a donation in the context of a clinical study by MSF, held until August of 2007. The Ministry of Health does not provide drugs for leishmaniasis, so access to drugs is not assured when clinical studies are finished. Access to patients, in order to perform active case finding, is difficult; the population is nomadic and lives in very remote areas. All patients are found by passive case detection and it is suspected that not all have access to treatment.

**ACCESS TO DRUGS**

Sodium stibogluconate and pentamidine are included in the National Essential Drug List for VL. Amphotericin B is listed for fungal infections. Sodium stibogluconate (Pentostam, GSK, and generic SSG, Albert David, India) are registered in Uganda. Generic SSG is available in private pharmacies. Liposomal amphotericin B is not registered.

**SOURCES OF INFORMATION**

- Dr Sagaki Patrick, Amudat Hospital.
- Dr Mbulamberi Dawson, Ministry of Health.
- Dr Joseph Olobo, Makerere University, Uganda. *Consultative Meeting on The Control of Leishmaniasis in the African Region. WHO/AFRO Addis Ababa, 23-25 Feb 2010.*

1. Guerin PJ, Olliaro P, Sundar S, et al (2002) Visceral leishmaniasis: current status of control, diagnosis, and treatment, and a proposed research and development agenda. Lancet Infectious Diseases 2, 494–501.

2. Kolaczinski JH, Reithinger R, Worku DT, Ocheng A, Kasimiro J et al (2008). Risk factors of visceral leishmaniasis in East Africa: a case-control study in Pokot territory of Kenya and Uganda. International Journal of Epidemiology 37:344–352.

3. Sentongo E, Ddumba E, Amandua J (2010). Cutaneous leishmaniasis: Report of the first case in Mulago National Referral and Teaching Hospital. Joint 6^th^ MU-CHS Annual Scientific Conference and 18^th^ UNACOH Annual Scientific Conference 22^nd^ - 24^th^ September 2010, ASC 10/Poster 1, p 12.
