# Supplementary material for: Leishmaniasis Worldwide and Global Estimates of Its Incidence
Source: PLoS One. 2012 May 31;7(5):e35671. doi: 10.1371/journal.pone.0035671 (PMC3365071; doi:10.1371/journal.pone.0035671)
Supplement: Text S95 — Leishmaniasis Country Profiles, Ukraine. (DOCX) [file pone.0035671.s095.docx]

**UKRAINE**


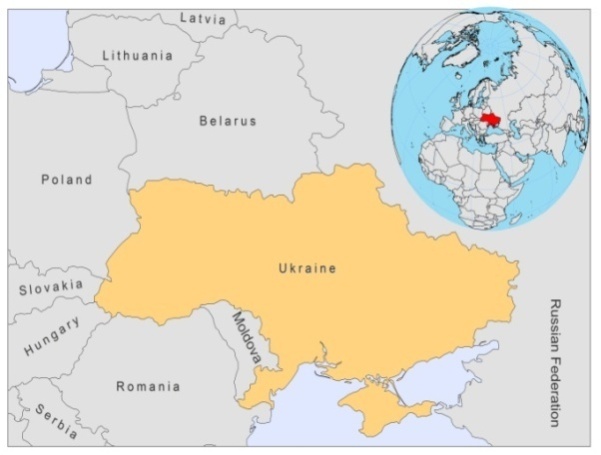


**BASIC COUNTRY DATA**

Total Population: 45,870,700

Population 0-14 years: 14%

Rural population: 32%

Population living under USD 1.25 a day: 0.1%

Population living under the national poverty line: no data

Income status: Lower middle income economy

Ranking: High human development (ranking 76)

Per capita total expenditure on health at average exchange rate (US dollar): 180

Life expectancy at birth (years): 69

Healthy life expectancy at birth (years): 59

**BACKGROUND INFORMATION**

From 1990 to 2007, 24 CL and 7 VL cases were reported in Ukraine. All cases were imported, mainly from Tajikistan and Armenia. 3 CL and 3 VL cases were diagnosed in 2008-2009 and 2 of the VL cases were fatal in 2008.

CL and VL are likely to be largely underreported as there is no awareness among doctors, and no provisions for diagnosis and treatment.

One case of HIV/*Leishmania* co-infection was diagnosed in 2009.

**PARASITOLOGICAL INFORMATION**

| ***Leishmania* species** | **Clinical form** | **Vector species** | **Reservoirs** |
| --- | --- | --- | --- |
| *L. infantum* | ZVL | *P. neglectus,*  *P. longiductus,* | *Canis familiaris* |
| *L. donovani* | VL | unknown |  |

**MAPS AND TRENDS**

**Visceral leishmaniasis**


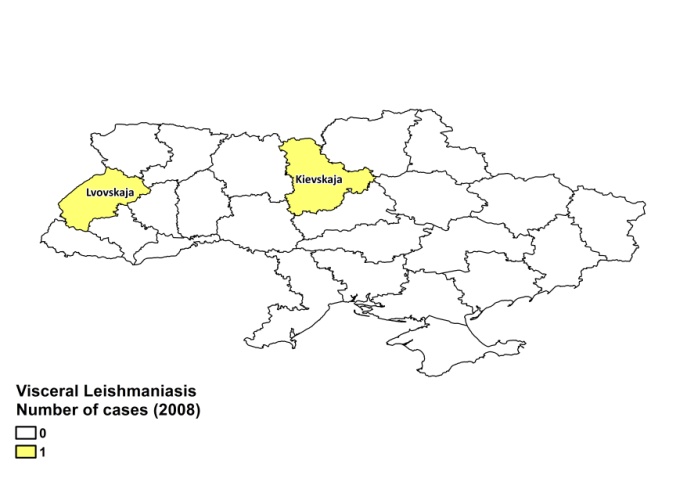

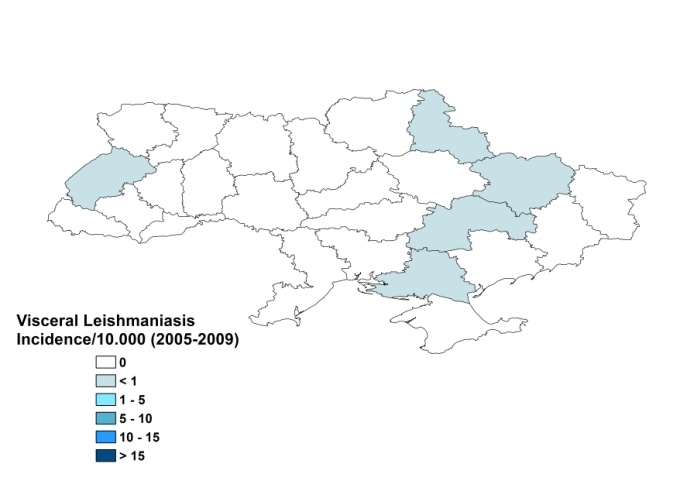


**Cutaneous leishmaniasis**


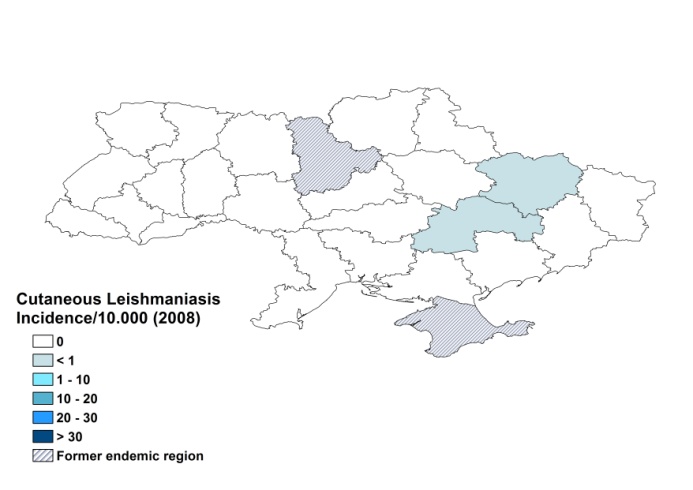

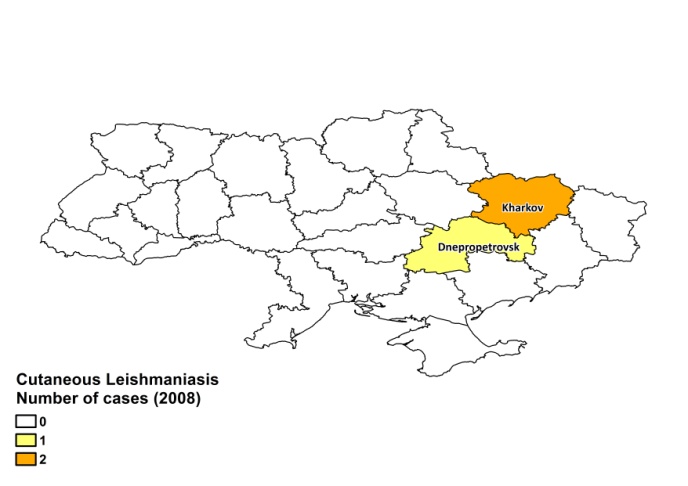


**Cases of visceral leishmaniasis**

| **2005** | **2006** | **2007** | **2008** |
| --- | --- | --- | --- |
| 1 | 3 | 3 | 2 |

**Cutaneous leishmaniasis trend**

**CONTROL**

The notification of leishmaniasis is mandatory in the country. There is no national leishmaniasis control program, no leishmaniasis vector control program and no bednet distribution program. Insecticide spraying is not done. There is no leishmaniasis reservoir control program.

**DIAGNOSIS, TREATMENT**

There are no protocols for diagnosis and treatment of leishmaniasis in Ukraine. Antimonials are used if available.

**ACCESS TO CARE**

Care for leishmaniasis is not provided for free in Ukraine. There is very problematic access to treatment. Drugs can only be obtained by hospitals and with great difficulties. In 2008, 2 cases of VL were fatal due to a lack of specific drugs. Diagnosis, if at all, is mostly very late, due to a lack of awareness of the disease among doctors and patients.

**ACCESS TO DRUGS**

There are no drugs registered for leishmaniasis and they are not available in the country. No drugs for leishmaniasis are included in the National Essential Drug List. Meglumine antimoniate (Glucantime, Sanofi) can sometimes be obtained, with difficulty, through private import.

**SOURCES OF INFORMATION**

- Dr Tamara Pavlikovska, Central Sanitary and Epidemiological Station of the Ministry of Health, Kiev. *Leishmaniasis in the European Region, a WHO consultative intercountry meeting, Istanbul, Turkey, 17–19 November 2009.*
