# Supplementary material for: Leishmaniasis Worldwide and Global Estimates of Its Incidence
Source: PLoS One. 2012 May 31;7(5):e35671. doi: 10.1371/journal.pone.0035671 (PMC3365071; doi:10.1371/journal.pone.0035671)
Supplement: Text S96 — Leishmaniasis Country Profiles, United States of America. (DOCX) [file pone.0035671.s096.docx]

**UNITED STATES OF AMERICA**


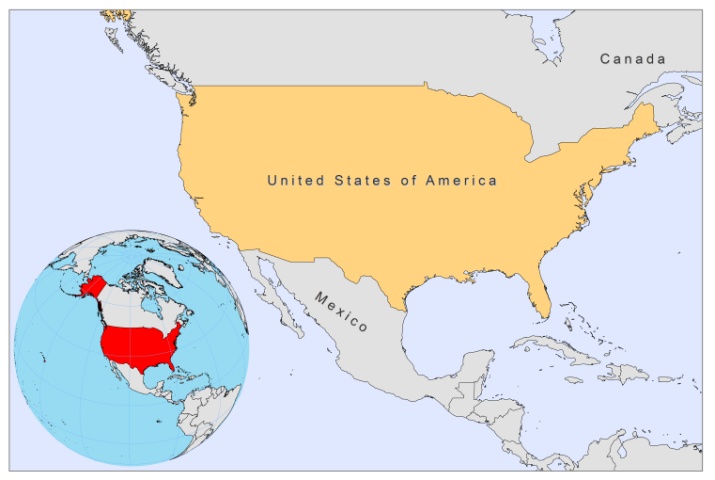


**BASIC COUNTRY DATA**

Total Population: 309,050,816

Population 0-14 years: 20%

Rural population: 18%

Population living under USD 1.25 a day: no data

Population living under the national poverty line: no data

Income status: High income economy: OECD

Ranking: Very high human development (ranking 4)

Per capita total expenditure on health at average exchange rate (US dollar): 7,410

Life expectancy at birth (years): 78

Healthy life expectancy at birth (years): 69

**BACKGROUND INFORMATION**

Canine VL (*L. infantum*) is highly prevalent among dogs, but autochtonous human cases have not been reported to date. Outbreaks in foxhound kennels occurred in 18 states (data only available for 2000-2003) and surveys in kennels demonstrated 326 seropositive dogs and 62 parasitologically confirmed infections [1]. VL is an imported disease among returning soldiers, but it is very rare [2].

CL is very common among returning soldiers from Iraq and Afghanistan; hundreds of cases have been reported [2]. CL is also often imported from Latin American countries. Autochtonous CL in humans is very rare, but occurs in Texas and Oklahoma; zoonotic *L. mexicana* transmission was demonstrated in Texas, Oklahoma and Arizona [3]. The first autochtonous case of CL was reported in Texas, near the Mexican border, in 1903. Until 1993, 26 more autochtonous cases were documented in Texas alone [4]. Exposure to vector and reservoir is predicted to have doubled by 2080, as the presence of both will extend northwards, in line with climate change [5].

HIV/*Leishmania* co-infection is very rare (to date, less than 5 in total) and there have only been imported cases so far.

**PARASITOLOGICAL INFORMATION**

| ***Leishmania***  **species** | **Clinical form** | **Vector species** | **Reservoirs** |
| --- | --- | --- | --- |
| *L. mexicana* | ZCL, DCL | *Lu. anthophora,*  *Lu. diabolica* | *Neotoma* spp. |
| *L. infantum* | Unknown | Unknown | *Canis familiaris* |

**MAPS AND TRENDS**

**Cutaneous leishmaniasis**


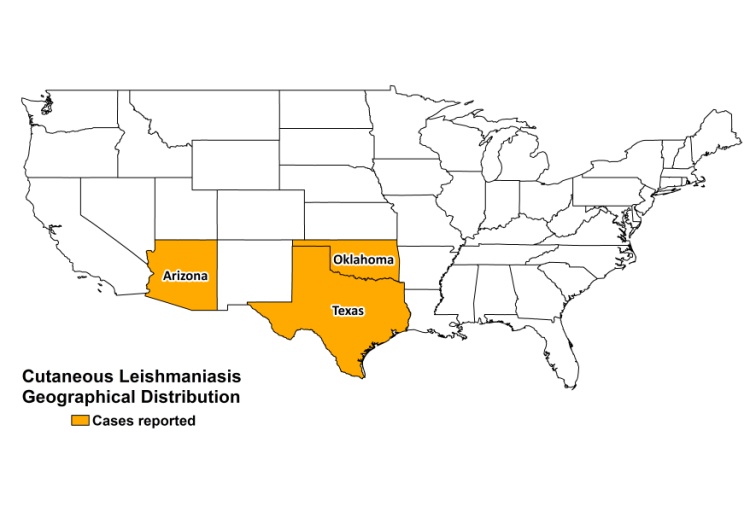


**Cutaneous leishmaniasis cases**

| **2005** | **2006** | **2007** | **2008** | **2009** | **2010** |
| --- | --- | --- | --- | --- | --- |
| 2 | 6 | 3 | 0 | 2 | 0 |

**CONTROL**

An epidemiological surveillance system has been in place since 1980. Notification of leishmaniasis is not mandatory and there is no national leishmaniasis control program. Case detection is passive. Vector control, which relies on education and spraying, is carried out whenever cases are detected. However, the current registration system does not record these activities. Positive dogs are sacrificed only if the owner agrees.

**DIAGNOSIS, TREATMENT**

**Diagnosis:**

CL: microscopic examination of skin samples and PCR.

**Treatment**

CL: antimonials, 20 mg Sb^v^/kg/day for 20 days. Second line is with liposomal amphotericin B.

**ACCESS TO CARE**

Care for leishmaniasis is not free; the cost depends on the individual health insurance of the patient. Antimonials (Pentostam, GSK) is provided for free through the CDC drug service, but other drugs must be obtained through the private sector (may be covered by insurance). The military service treats cases in military personnel.

**ACCESS TO DRUGS**

The USA does not have an essential drug list. Drugs for leishmaniasis, such as liposomal amphotericin (AmBisome, Gilead), are sold in private pharmacies, for commercial prices. Miltefosine is available, but very expensive (one adult treatment costs $5,714.80). Antimonials and miltefosine are not registered. There are plans to register miltefosine (Paladin, Canada) in 2010.

**SOURCES OF INFORMATION**

1. Duprey ZH, Steurer FJ, Rooney JA, Kirchhoff LV, Jackson JE et al (2006). [Canine visceral leishmaniasis, United States and Canada, 2000-2003.](http://www.ncbi.nlm.nih.gov/pubmed/16704782) Emerg Infect Dis 12(3):440-6.

2. Peter J. Weina, Ronald C. Neafie, Glenn Wortmann et al (2004). Old World Leishmaniasis: An Emerging Infection among Deployed US Military and Civilian Workers. CID 39:1674-80.

3. Wright NA, Davis LE, Aftergut KS, Parrish CA, Cockerell CJ (2008). [Cutaneous leishmaniasis in Texas: A northern spread of endemic areas.](http://www.ncbi.nlm.nih.gov/pubmed/18249464) J Am Acad Dermatol 58(4):650-2.

4 Mc Hugh CP, Melby PC, Lafon SG (1996). Leishmaniasis in Texas: epidemiology and clinical aspects of human cases. Am J Trop Med Hyg 55 (5): p 547-555.

5 . González C, Wang O, Strutz SE, González-Salazar C, Sánchez-Cordero V et al (2010). [Climate change and risk of leishmaniasis in north america: predictions from ecological niche models of vector and reservoir species.](http://www.ncbi.nlm.nih.gov/pubmed/20098495) PLoS Negl Trop Dis 4(1):e585.
