# Supplementary material for: Leishmaniasis Worldwide and Global Estimates of Its Incidence
Source: PLoS One. 2012 May 31;7(5):e35671. doi: 10.1371/journal.pone.0035671 (PMC3365071; doi:10.1371/journal.pone.0035671)
Supplement: Text S97 — Leishmaniasis Country Profiles, Uzbekistan. (DOCX) [file pone.0035671.s097.docx]

**UZBEKISTAN**


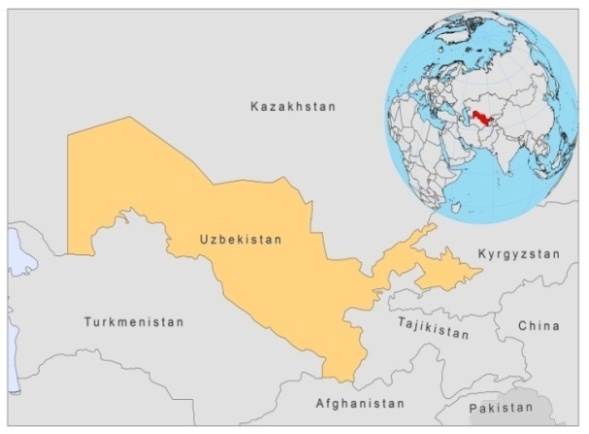


**BASIC COUNTRY DATA**

Total Population: 28,160,361

Population 0-14 years: 29%

Rural population: 63%

Population living under USD 1.25 a day: no data

Population living under the national poverty line: no data

Income status: Lower middle income economy

Ranking: Medium human development (ranking 115)

Per capita total expenditure on health at average exchange rate (US dollar): 62

Life expectancy at birth (years): 68

Healthy life expectancy at birth (years): 59

**BACKGROUND INFORMATION**

VL was practically eradicated (1943-1949: 1,220 cases; in 1967: 2 cases), but the number of VL cases is on the rise again. Before 2004, less than 5 cases were reported per year, but in 2009, 25 cases were registered before the end of the year, all in one district (Namangan), and most probably due to an increased awareness. Most cases are in children. In a recent survey of 521 children in endemic areas, 11 clinically suspect cases were identified; 10% of the children were seropositive and *Leishmania* DNA was detected in 53% of the 142 children tested by PCR. Canine infection rates are high. A total of 25% of 162 dogs surveyed were suspected of being infected and 39% of the dogs were seropositive (all seropositive dogs were sacrificed) [1]. Underreporting is a problem.

CL is prevalent in 5 districts. In 2004, there was a local outbreak with 210 cases. There is an increasing awareness of CL in local communities and among doctors, due to recent training programs.

No cases of HIV/*Leishmania* co-infection have been reported.

**PARASITOLOGICAL INFORMATION**

| ***Leishmania* species** | **Clinical form** | **Vector species** | **Reservoirs** |
| --- | --- | --- | --- |
| *L. infantum* | ZVL | *P. longiductus* | *Canis familiaris* |
| *L. major* | CL | *P. papatasi* | *Rhombomys opimus* |
| *L. tropica* | ACL | *P. sergenti* | Human |

**MAPS AND TRENDS**

**Visceral leishmaniasis**


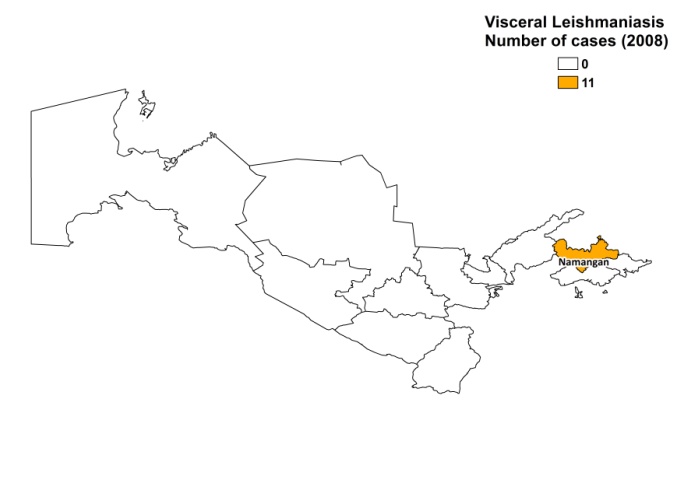

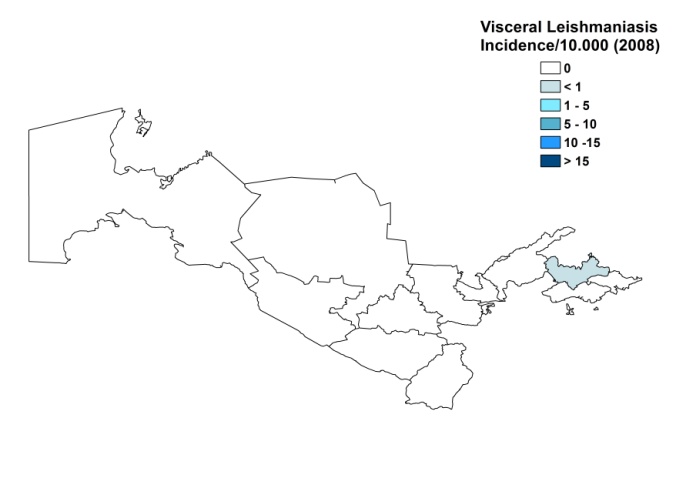


**Cutaneous leishmaniasis**

**
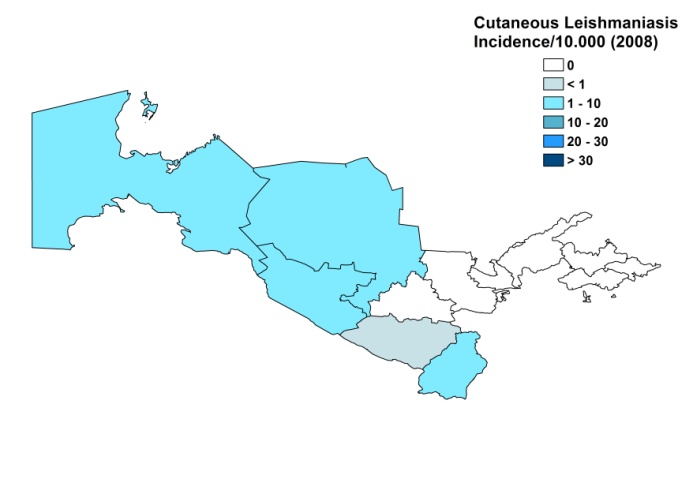

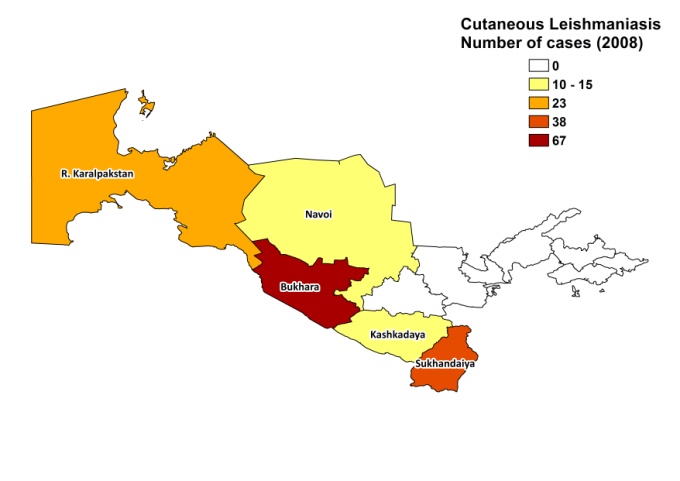
**

**Visceral leishmaniasis trend**

**Cutaneous leishmaniasis trend**

**CONTROL**

The notification of leishmaniasis is mandatory in the country and a national leishmaniasis control program has been in place since 2004. There is a leishmaniasis vector control program (no bednet distribution). Insecticide spraying is done, but insufficiently. There is a leishmaniasis reservoir control program. For rodent controls, mechanical burrow destruction and food poisoning have been used. Control for dogs is linked to the anti-rabies program. In the VL focus, all seropositive dogs found in a survey were sacrificed.

**DIAGNOSIS, TREATMENT**

**Diagnosis**

CL: mostly on clinical grounds. Confirmation with microscopic examination of skin lesion sample is rarely done.

VL: confirmation by microscopic examination of bone marrow sample.

**Treatment**

VL: antimonials, 20 mg Sb^v^/kg/day for 28 days. Cure rate reported is 100%.

CL: unspecific means (broad-range antibiotics, sterile bandages, local ointments). Complicated cases: antimonials, 20 mg Sb^v^/kg/day.

**ACCESS TO CARE**

Care for leishmaniasis is provided for free. However, diagnosis of VL is often made very late, due to a lack of awareness among doctors. In CL foci, the awareness of doctors has increased, however, diagnosis and treatment are often not available. The government does not provide drugs for leishmaniasis, but in 2008, WHO donated 2,860 vials of Glucantime (Sanofi), enough for the systemic treatment of about 60 patients. This may have been sufficient for the patients reported that year. However, in 2007, there was no WHO donation and patients could only be treated with the remainder of a 2006 donation. Not all patients had access to treatment.

**ACCESS TO DRUGS**

No drugs for leishmaniasis are included in the National Essential Drug List. They are not registered in the country and are not available, except if donated by WHO and in illegal drugs markets (antimonials, smuggled in from Afghanistan).

**SOURCES OF INFORMATION**

- Drs Dmitriy A. Kovalenko and Shavkat A. Razakov. Isaev Scientific Research Institute of Medical Parasitology, Samarqand. *Leishmaniasis in the European Region, a WHO consultative intercountry meeting, Istanbul, Turkey, 17–19 November 2009.*

1. [Kovalenko DA](http://www.ncbi.nlm.nih.gov/pubmed?term=%22Kovalenko%20DA%22%5BAuthor%5D), [Razakov SA](http://www.ncbi.nlm.nih.gov/pubmed?term=%22Razakov%20SA%22%5BAuthor%5D), [Ponirovsky EN](http://www.ncbi.nlm.nih.gov/pubmed?term=%22Ponirovsky%20EN%22%5BAuthor%5D), [Warburg A](http://www.ncbi.nlm.nih.gov/pubmed?term=%22Warburg%20A%22%5BAuthor%5D), [Nasyrova RM](http://www.ncbi.nlm.nih.gov/pubmed?term=%22Nasyrova%20RM%22%5BAuthor%5D) et al (2011). Canine leishmaniosis and its relationship to human visceral leishmaniasis in Eastern Uzbekistan. [Parasit Vectors](http://www.ncbi.nlm.nih.gov/pubmed/21489254) 4:58.
