# Supplementary material for: Leishmaniasis Worldwide and Global Estimates of Its Incidence
Source: PLoS One. 2012 May 31;7(5):e35671. doi: 10.1371/journal.pone.0035671 (PMC3365071; doi:10.1371/journal.pone.0035671)
Supplement: Text S98 — Leishmaniasis Country Profiles, Venezuela. (DOCX) [file pone.0035671.s098.docx]

**VENEZUELA (Bolivarian Republic of)**

**
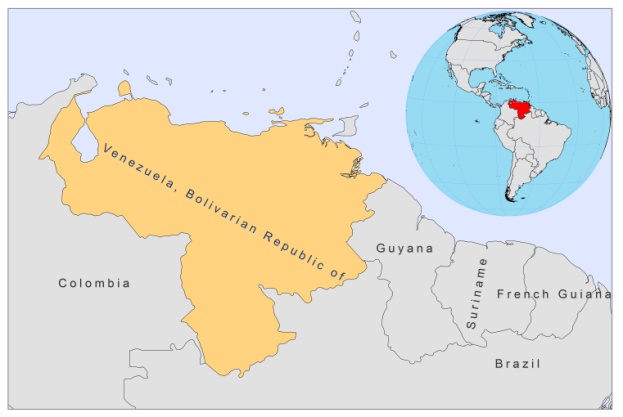
**

**BASIC COUNTRY DATA**

Total Population: 28,834,000

Population 0-14 years: 29%

Rural population: 6%

Population living under USD 1.25 a day: 3.5%

Population living under the national poverty line: 29%

Income status: Upper middle income economy

Ranking: High human development (ranking 73)

Per capita total expenditure on health at average exchange rate (US dollar): 686

Life expectancy at birth (years): 74

Healthy life expectancy at birth (years): 64

**BACKGROUND INFORMATION**

CL is mainly an occupational disease, affecting, among others, farmers, hunters and military personnel. It occurs throughout the Venezuelan territory, in 22 municipalities, and it can be considered as both a rural and urban disease; 27,212,600 people are considered to be at risk for CL, while 21,397,700 are at risk for MCL, in 14 municipalities. The incidence is estimated at 9.9 per 100,000 inhabitants. 80% of CL cases are caused by *L.braziliensis,* although other species have been demonstrated, including *L.infantum* [1]. *L.braziliensis* was found in a significant rate in dogs (up to 20%), but also in donkeys (up to 30%), in different localities of the Cojedes state [2,3].

CL cases are reported from small villages, new settlements located near wooded areas and the outskirts of towns or cities. The highest incidence in old endemic foci is in the Andean region, in the west, and in the mountains of the Serranía de la Costa, in central Venezuela (Bolivarian Republic of). New foci are mainly located in the lowlands of the Amazonian basin, including outbreaks in non-immune populations, mainly immigrant agricultural workers and miners, close to the forest [4]. The first human case caused by *L. venezuelensis* was diagnosed near Barquisimeto, in Lara state, in 1974.

During the period from 1988 to 2007, 47,762 cases of CL were reported, with an annual average of 2,388 cases. Of this total, 98.5% are localized CL, 0.84% MCL, 0.5% intermediate CL and 0.16% DCL [5]. The male:female ratio has been 1:1.7 in the last five years. Although cases have been reported in all 23 states and the federal capital district, the most affected areas are those in the mountains, in particular the Andes states. Between 1994-2008, around 20-50 MCL cases occurred every year.

VL occurs sporadically, with a very low endemicity, in almost all states, but there are four major foci in central, western, southern and eastern Venezuela (Bolivarian Republic of) [5]. The annual average from 1990 to 2007 was 31 cases, with a global incidence of 0.2 per 100,000 persons. 26% of the cases are from the island of Margarita, Nueva Esparta state, where the incidence is around 3-4% and the case fatality rate is 3% [5,7,8]. In the eastern focus of Anzoátegui state, the incidence is estimated at 6 per 100,000 inhabitants [9]. The main reservoir is the dog, with an infection rate of 21.6% in the island of Margarita [9] and a certain degree of clustering with human cases in micro-foci of transmission [7]. Approximately 15,034,800 people are considered to be at risk for VL in 14 municipalities.

HIV-*Leishmania* co-infection has been reported.

**PARASITOLOGICAL INFORMATION**

| ***Leishmania* species** | **Clinical form** | **Vector species** | **Reservoirs** |
| --- | --- | --- | --- |
| *L. braziliensis* | ZCL, MCL | *Lu. ovallesi,*  *Lu. trinidanensis,*  *Lu. spinicrassa,*  *Lu. panamensis* | Unknown |
| *L. colombiensis* | ZCL | *Lu. panamensis,*  *Lu. gomezi* | Unknown |
| *L. venezuelensis* | ZCL, DCL | *Lu. olmeca bicolor* | Unknown |
| *L. amazonensis* | ZCL, DCL | *Lu. flaviscutellata,*  *Lu. reducta* | Unknown |
| *L. pifanoi* | DCL | *Lu. flaviscutellata* | Unknown |
| *L. garnhami* | ZCL | *Lu. youngi* | Unknown |
| *L. infantum* | ZVL | *Lu. longipalpis,*  *Lu. evansi,*  *Lu. pseudolongipalpis* | *Canis familiaris* |
| *L. guyanensis* | ZCL | Unknown | Unknown |

**MAPS AND TRENDS**

**Visceral leishmaniasis**


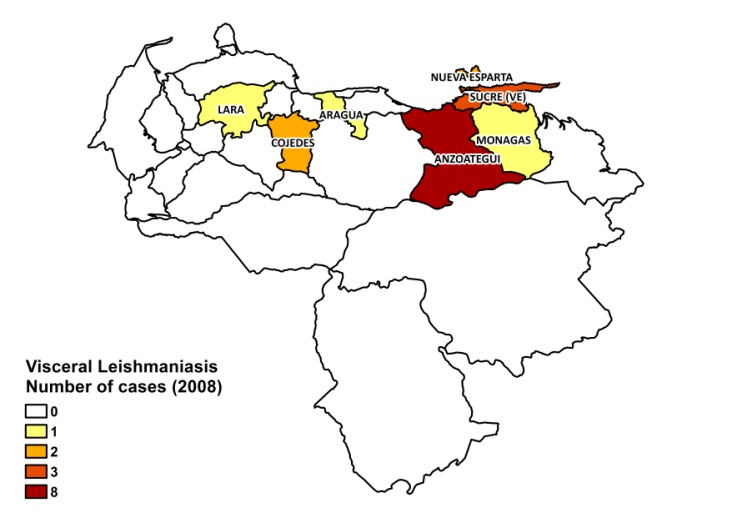

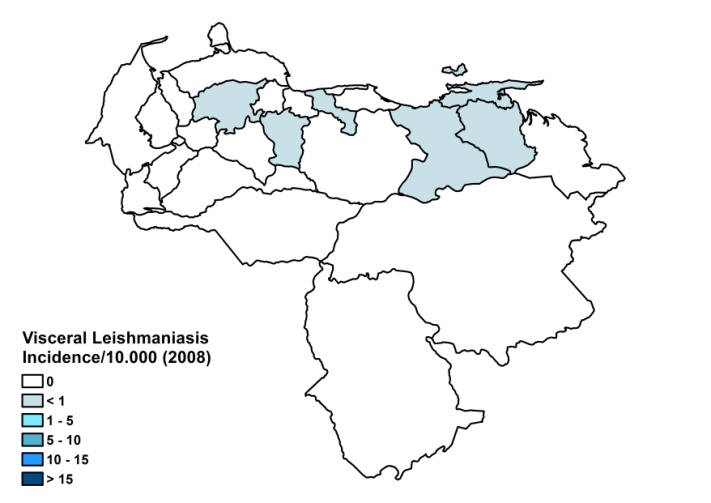


**Cutaneous leishmaniasis**

**
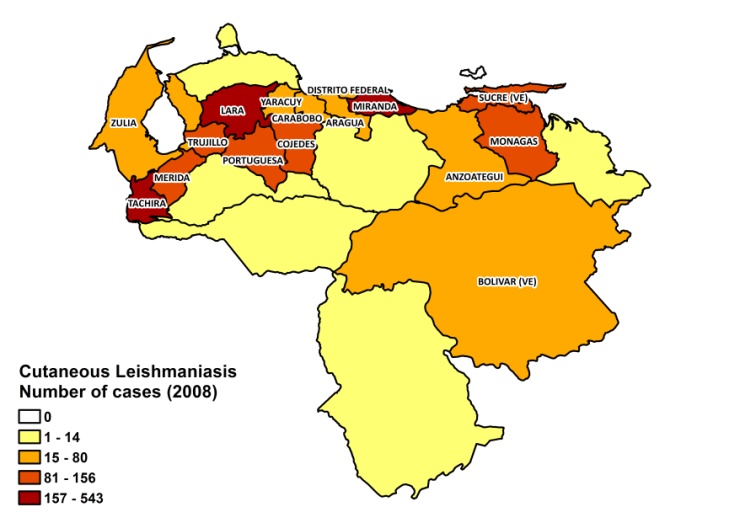
**

**
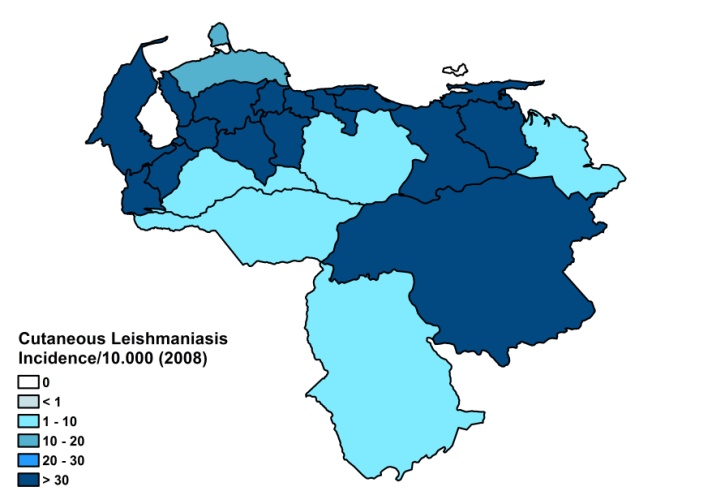
**

**Visceral leishmaniasis trend**

**Cutaneous leishmaniasis trend**

**Mucocutaneous leishmaniasis trend**

**CONTROL**

Leishmaniasis is a notifiable disease. A national control program has been in place since 1965. Case detection is passive. Control strategies include vector control (spraying) and canine control (elimination of seropositive dogs).

**DIAGNOSIS, TREATMENT**

**Diagnosis:**

CL: clinical, confirmation by the Montenegro test and microscopic examination of lesion samples (in >90% of cases). PCR is available at the Instituto de Biomedicina.

VL: rapid diagnostics rK39 antigen-based tests, at health facility level, and ELISA of bone marrow aspirates, at central level and in the state of Nueva Esparta.

**Treatment:**

CL: first line treatment is 3 doses of immunotherapy. There is a 92% cure rate, with 5% recurring lesions and 1% mucosal involvement. Second line treatment: antimonials, 20 mg Sb^v^/kg/day for 20 days. Cure rate is 99%. Third line treatment: miltefosine, 2-2.5 mg/kg/day, 30 to 45 days. Cure rate is 50%.

MCL: is treated with a combination of immunotherapy and antimonials. Overall cure rate for MCL is 90%, with 10% recurring lesions.

VL: first line treatment: antimonials. Second line treatment is with amphotericin B. Overall cure rate is 100%.

**ACCESS TO CARE**

Patients are treated in the public sector, where treatment is provided for free. VL can be diagnosed and treated at primary health care level and CL at outreach post level. 90% of CL cases are cured with immunotherapy and do not need drugs. However, often, there is a shortage of drugs and patients then need to purchase them themselves. The government provided no antimonials in 2007 and only a small quantity in 2008. Sanofi donated an additional quantity and a small donation of miltefosine was also received. Around 10% of cases are treated in the private sector. All patients are thought to have access to care.

**ACCESS TO DRUGS**

Meglumine antimoniate is included in the essential drug list and Glucantime (Sanofi) is registered. Drugs for leishmaniasis (Glucantime and miltefosine) are sold in private pharmacies. One vial of Glucantime costs 2 USD, leading to a treatment cost of 22 USD for intralesional treatment. Miltefosine is very expensive and out of reach for most.

**SOURCES OF INFORMATION**

- Dr Rafael Borges. Instituto de Biomedicina, Ministry of Health. *Leishmaniasis en la Región de las Américas. Reunión de coordinadores de Programa Nacional de Leishmaniasis. OPS/OMS. Medellín, Colombia. 4-6 junio 2008.*

1. De Lima H, Rodríguez N, Feliciangeli MD, Barrios MA, Sosa A et al (2009). [Cutaneous leishmaniasis due to Leishmania chagasi/Le. infantum in an endemic area of Guarico State, Venezuela.](http://www.ncbi.nlm.nih.gov/pubmed/19150102) Trans R Soc Trop Med Hyg 103(7):721-6.

2. Cardenas R, Sandoval CM, Rodriguez-Morales AJ, Bendezu H, Gonzalez A et al (2006). [Epidemiology of American tegumentary leishmaniasis in domestic dogs in an endemic zone of western Venezuela.](http://www.ncbi.nlm.nih.gov/pubmed/17253053) Bull Soc Pathol Exot 99(5):355-8.

3. Aguilar CM, Fernández E, Fernández R, Cannova DC, Ferrer E et al (1998). [Urban visceral leishmaniasis in Venezuela.](http://www.ncbi.nlm.nih.gov/pubmed/9698836) Mem Inst Oswaldo Cruz 93(1):15-6.

4. González R, Devera R, Madrid C, Zghayer S (2000). [An evaluation of an outbreak of American cutaneous leishmaniasis in a rural community in Bolivar State, Venezuela].](http://www.ncbi.nlm.nih.gov/pubmed/10881116) Rev Soc Bras Med Trop33(1):31-7.

5. Convit J, Ulrich M, Fernández CT, Tapia FJ, Cáceres-Dittmar G et al (1993). [The clinical and immunological spectrum of American cutaneous leishmaniasis.](http://www.ncbi.nlm.nih.gov/pubmed/8249076) Trans R Soc Trop Med Hyg 87(4):444-8.

6. Zerpa O, Ulrich M, Borges R, Rodríguez V, Centeno M et al (2003). [Epidemiological aspects of human and canine visceral leishmaniasis in Venezuela.](http://www.ncbi.nlm.nih.gov/pubmed/12804153) Rev Panam Salud Publica 4:239-45.

7. Zerpa O, Ulrich M, Benitez M, Avila C, Rodríguez V et al (2002). [Epidemiological and immunological aspects of human visceral leishmaniasis on Margar](http://www.ncbi.nlm.nih.gov/pubmed/12563469)**[i](http://www.ncbi.nlm.nih.gov/pubmed/12563469)**[ta Island, Venezuela.](http://www.ncbi.nlm.nih.gov/pubmed/12563469) Mem Inst Oswaldo Cruz 97(8):1079-83.

8. [Zulueta AM](http://www.ncbi.nlm.nih.gov/pubmed?term=%22Zulueta%20AM%22%5BAuthor%5D), [Villarroel E](http://www.ncbi.nlm.nih.gov/pubmed?term=%22Villarroel%20E%22%5BAuthor%5D), [Rodriguez N](http://www.ncbi.nlm.nih.gov/pubmed?term=%22Rodriguez%20N%22%5BAuthor%5D), [Feliciangeli MD](http://www.ncbi.nlm.nih.gov/pubmed?term=%22Feliciangeli%20MD%22%5BAuthor%5D), [Mazzarri M](http://www.ncbi.nlm.nih.gov/pubmed?term=%22Mazzarri%20M%22%5BAuthor%5D) et al (1999). Epidemiologic aspects of American visceral leishmaniasis in an endemic focus in Eastern Venezuela. [Am J Trop Med Hyg](http://www.ncbi.nlm.nih.gov/pubmed?term=leishmaniasis%2C%20venezuela%2C%20zulueta##) 61(6):945-50.

9. Zerpa O, Ulrich M, Negrón E, Rodríguez N, Centeno M et al (2000). [Canine visceral leishmaniasis on Margarita Island (Nueva Esparta, Venezuela).](http://www.ncbi.nlm.nih.gov/pubmed/11132371) Trans R Soc Trop Med Hyg 94(5):484-7.
