# Supplementary material for: Leishmaniasis Worldwide and Global Estimates of Its Incidence
Source: PLoS One. 2012 May 31;7(5):e35671. doi: 10.1371/journal.pone.0035671 (PMC3365071; doi:10.1371/journal.pone.0035671)
Supplement: Text S99 — Leishmaniasis Country Profiles, West Bank and Gaza Strip. (DOCX) [file pone.0035671.s099.docx]

**WEST BANK AND GAZA STRIP**

**
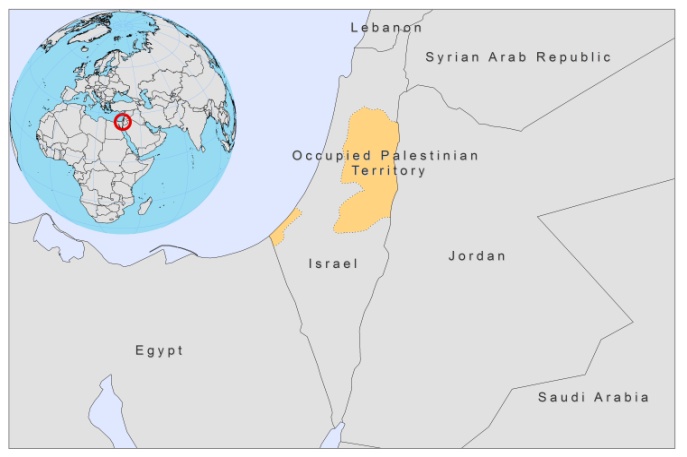
**

**BASIC DATA**

Total Population: 4,152,102

Population 0-14 years: 42%

Rural population: 28%

Population living under 1.25 USD a day: no data

Population living under the national poverty line: 21.9%

Income status: Lower middle income economy

Ranking: Medium human development (ranking 114)

Per capita total expenditure on health at average exchange rate (US dollar): no data

Life expectancy at birth (years): 72

**BACKGROUND INFORMATION:**

CL was first detected in the beginning of the 20^th^ century [1]. VL and CL are both endemic, except in the Gaza strip, where both forms have not been reported. Both CL and VL are thought to be underreported.

VL by *L.infantum* is endemic in the West Bank and more common than in Israel. Between 1993 and 2007, 76 cases of VL were reported from the Hebron district, all in children under 9 years old [2]. Two large surveys among school children in the north and the south of West Bank showed a 7.5% resp. 8.4% seropositivity [2,3]. In bordering northern Israel, seroprevalence was 3.0% [4], probably referring to better economic circumstances.

The incidence of CL increased dramatically after 1967. Its incidence has further increased steadily over the last 4 years, with the highest incidence in the Jericho area. CL by *L.tropica* is endemic in the West Bank, while CL in the Jericho area seems to be mainly caused by *L.major* [1].

One case of HIV/CL co-infection was reported in a child in Jericho in 1994.

**PARASITOLOGICAL INFORMATION**

| ***Leishmania***  **species** | **Clinical form** | **Vector species** | **Reservoirs** |
| --- | --- | --- | --- |
| *L. infantum* | ZVL | *P. syriacus P. perfiliewi,*  *P. tobbi* | *Canis familiaris* |
| *L. tropica* | ACL | *P.sergenti* |  |
| *L. major* | ZCL | *P.papatasi* | *Psammomys obesus* |

**MAPS AND TRENDS**

**
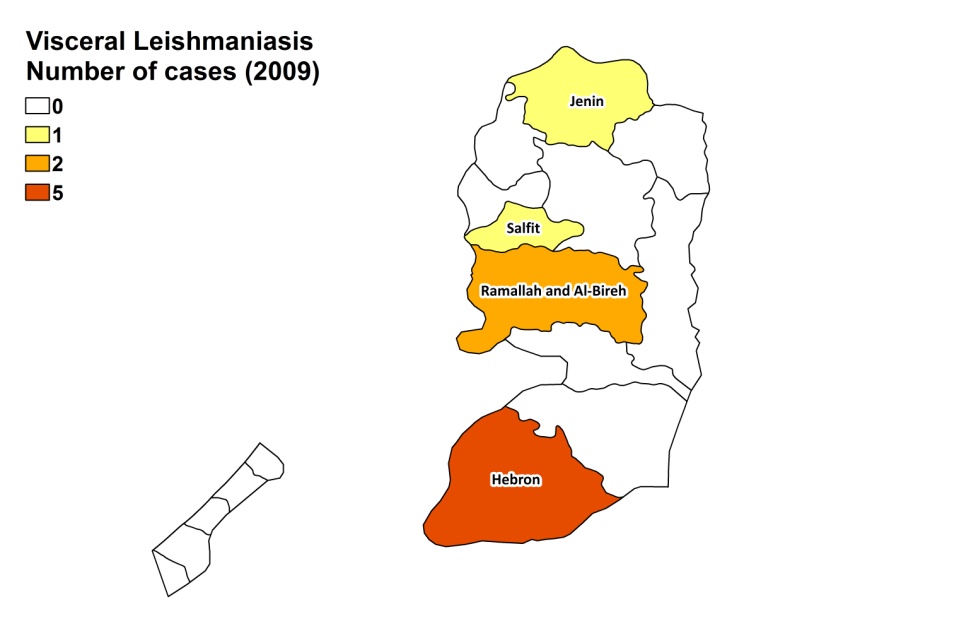
Visceral leishmaniasis**

**
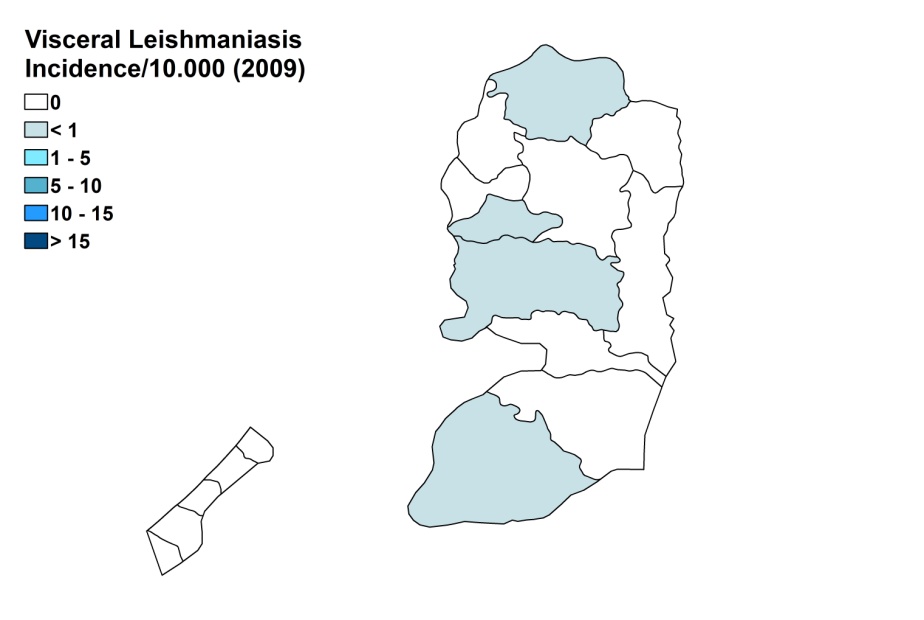
**

**Cutaneous leishmaniasis**

**
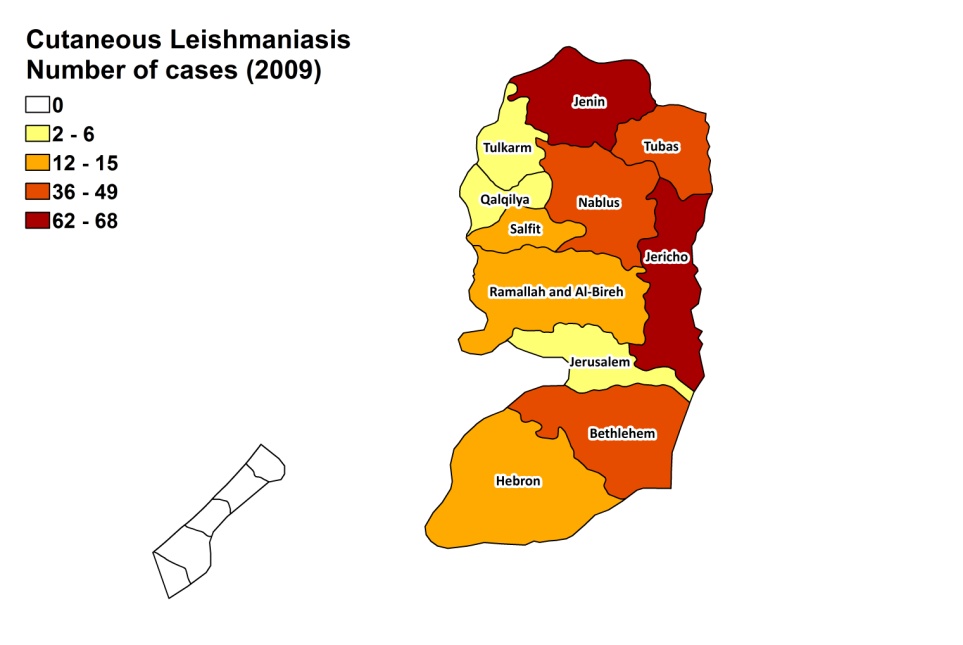
**

**
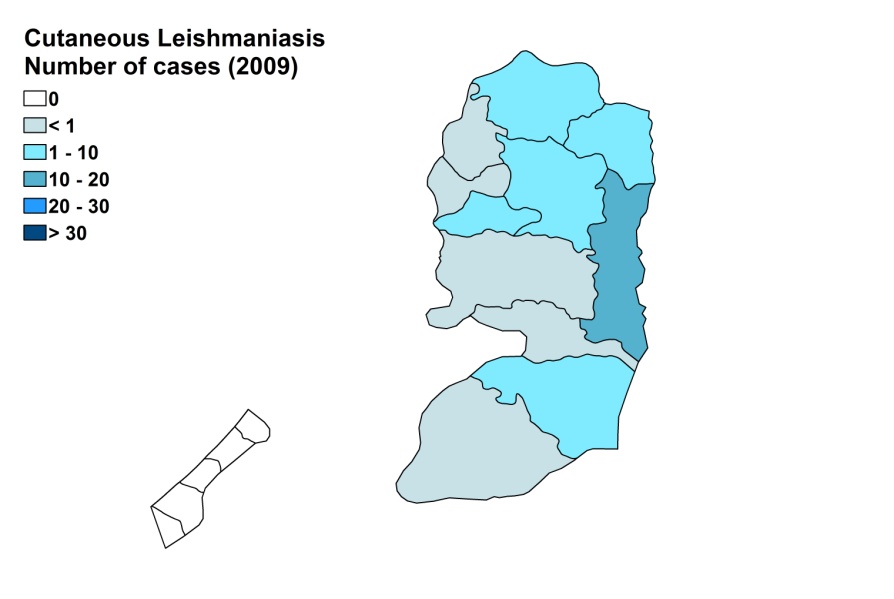
**

**Visceral leishmaniasis trend**

**Cutaneous leishmaniasis trend**

**CONTROL**

The notification of leishmaniasis is mandatory in the territory and a leishmaniasis control program for CL has been in place since 1996. There is a leishmaniasis vector control program and insecticide spraying is regularly done. There is no leishmaniasis reservoir program, but surveys of dogs are regularly done and positive dogs are sacrificed.

**DIAGNOSIS, TREATMENT**

**Diagnosis:**

CL: on clinical grounds. Confirmation by microscopic examination of a skin lesion sample. PCR is performed in one diagnostic center in Jericho.

VL: confirmation by microscopic examination of bone marrow aspirate and PCR (in one diagnostic center).

**Treatment**

VL: antimonials, 20 mg Sb^v^/kg/day for 3 weeks. In 2005, there were 2 treatment failures. Full recovery was achieved after treatment with amphotericin B.

CL: antimonials, intralesional or systemic (20 mg Sb^v^/kg/day) for 14 days and in case of poor response, an additional 7 days. Paromomycin ointment and cryotherapy are also sometimes used for CL.

**ACCESS TO CARE**

Care for leishmaniasis is provided for free. An unknown proportion of patients seek care in private facilities, which is very expensive. CL and VL are diagnosed and treated at primary health care level, but advanced diagnostic techniques are restricted to one research center in Jericho. The Ministry of Health purchases antimonials (Pentostam, GSK) for the treatment of VL and CL. Treatment is thought to be accessible for all patients.

**ACCESS TO DRUGS**

Sodium stibogluconate is included in the National Essential Drug List for VL and CL. Drugs for leishmaniasis are not available at pharmacies or drug markets. No antimonials are registered in West Bank and Gaza Strip.

**SOURCES OF INFORMATION**

1. Jaffe CL, Baneth G, Abdeen ZA, Schlein J, Warburg A (2004). Leishmaniasis in Israel and the Palestinian Authority. Trends Parasitol 20(7):328-32.

2. Amroa A, Azmib K, Schönian G, Nasereddinb A, Alsharabatic MB et al (2009). Epidemiology of paediatric visceral leishmaniasis in Hebron district, Palestine. Trans R Soc Trop Med Hyg 103(7):731-6.

3. Bader KA. Visceral Leishmaniasis in the West Bank; epidemiological aspects [PhD thesis]. Jerusalem: Hebrew University; 2005.

4. Adini I, Ephros M, Chen J, Jaffe CL (2003). Asymptomatic visceral leishmaniasis, northern Israel. Emerg Infect Dis 9:397-8.
