# Supplementary material for: Leishmaniasis Worldwide and Global Estimates of Its Incidence
Source: PLoS One. 2012 May 31;7(5):e35671. doi: 10.1371/journal.pone.0035671 (PMC3365071; doi:10.1371/journal.pone.0035671)
Supplement: Text S100 — Leishmaniasis Country Profiles, Yemen. (DOCX) [file pone.0035671.s100.docx.docx]

**YEMEN**


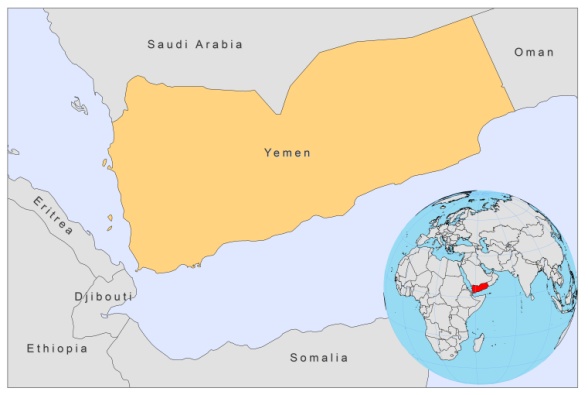


**BASIC COUNTRY DATA**

Total Population: 24,052,514

Population 0-14 years: 44%

Rural population: 68%

Population living under USD 1.25 a day: 17.5%

Population living under the national poverty line: no data

Income status: Lower middle income economy

Ranking: Low human development (ranking 154)

Per capita total expenditure on health at average exchange rate (US dollar): 64

Life expectancy at birth (years): 65

Healthy life expectancy at birth (years): 49

**BACKGROUND INFORMATION**

The whole country of Yemen is endemic for CL, except for the highlands above 2300 m. Both CL caused by *L.major* and CL caused by *L. tropica* are prevalent. *L. tropica* is probably the predominant species. In two recent surveys held in Yemen the causative parasite was *L.tropica* in around 90% of CL cases [1,2]. In 2004, 2 outbreaks were reported, one of 500 cases in the Taiz region, and another one of 900 cases. Leishmaniasis is a highly neglected disease in Yemen. Reporting of cases is unreliable and there is very substantial underreporting. In lack of a functioning reporting system in rural areas, all reported cases are from urban areas only. The reporting system does not differentiate between CL and VL. In 2006, reporting of leishmaniasis was absent.

VL is rare, and probably substantially underreported. Taiz, Lahj and Ibb governorates seem the most affected areas.

*Leishmania*/HIV co-infection has not been reported.

**PARASITOLOGICAL INFORMATION**

| ***Leishmania* species** | **Clinical form** | **Vector species** | **Reservoirs** |
| --- | --- | --- | --- |
| *L. infantum* | ZVL | unknown | *Canis familiaris* |
| *L. donovani* | AVL | *P. orientalis* |  |
| *L. major* | ZCL | *P. bergeroti, P. duboscqi,*  *P. papatasi* | unknown |
| *L. tropica* | ACL | *P. sergenti* |  |

**MAPS AND TRENDS**

**Visceral leishmaniasis**

**
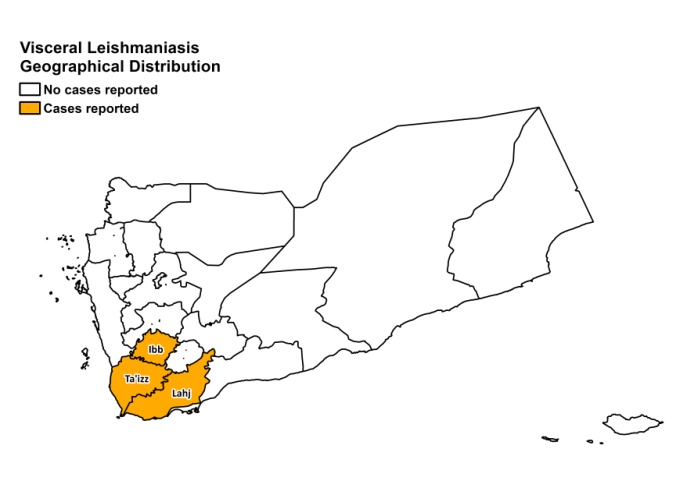
**

**Cutaneous leishmaniasis**

**
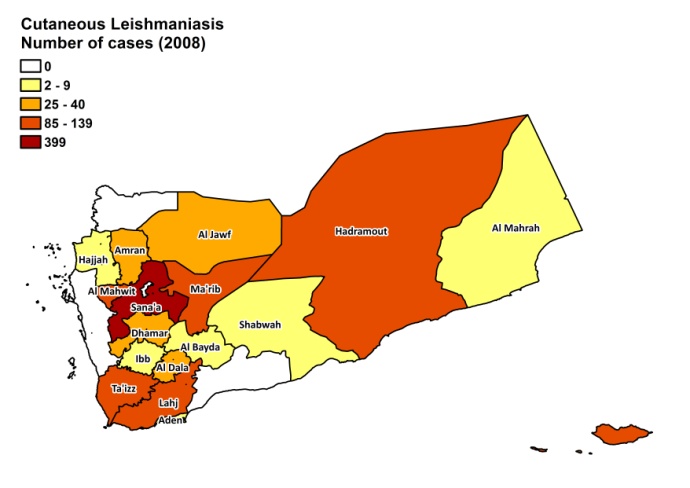
**

**
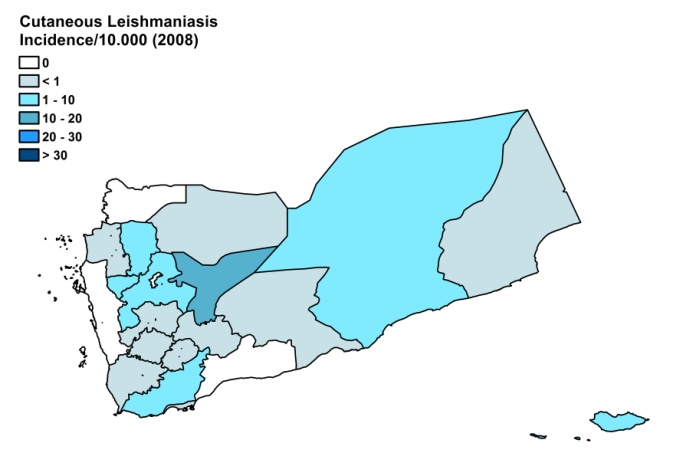
**

**Cutaneous leishmaniasis trend**

**CONTROL**

Notification of leishmaniasis is not mandatory in the country and there is no national leishmaniasis control program. Case detection is passive. There is no vector control program, but the wide use of insecticide by farmers for agriculture and the spraying of insecticide for malaria control led to a decrease of the prevalence of sandflies in some parts of the country. There is a bednet distribution program for malaria. There is no reservoir control program.

**DIAGNOSIS, TREATMENT**

**Diagnosis**

CL: mostly only on clinical grounds. Confirmation with microscopic examination of skin lesion sample or ELISA.

VL: confirmation by microscopic examination of bone marrow aspirate or by ELISA.

**Treatment**

VL: antimonials, 20 mg Sb^v^/kg/day for 21 days.

CL: antimonials, intralesional or systemic (15-20 mg Sb^v^/kg/day for 12-20 days) and/or cryotherapy. Cure rate for cryotherapy is estimated at 90%, for antimonials at 80%.

**ACCESS TO CARE**

Access to care for leishmaniasis is not free in Yemen. Patients have to pay a registration fee of 0.25-0.50 USD per visit. The consultation itself is free, but a fee of 1-10 USD for laboratory tests has to be paid by the patients. The government did not provide any anti-leishmanial drugs in 2007 and 2008. Therefore, antimonials have to purchased by patients themselves and cost 30-50 USD for a course of intralesional treatment. Health workers are known to charge informal fees to patients. All these costs form a barrier in seeking treatment for poor patients.

Diagnosis and treatment for VL is only possible in regional and specialized hospitals. Diagnosis for CL is done on clinical grounds at primary health care level. Parasitological confirmation is only possible in regional and specialized hospitals. Treatment for CL can be given at primary health care level, but there is a lack of trained human resources for diagnosing and treating leishmaniasis. Patients in rural areas often live in very remote places, with no health facilities and no transport; if transport exists, patients cannot afford it.

There is a great lack of awareness of the nature of the disease and the possibility of medical treatment. About 90% of patients are estimated to seek substandard private care or care from traditional healers. Methods used are corrosive (chemicals, such as sulphuric and hydrochloric acid, direct heat, and a poultice prepared from plants).

**ACCESS TO DRUGS**

Sodium stibogluconate is included in the National Essential Drug List. Sodium stibogluconate is for sale in pharmacies as well as in unregulated drug markets (smuggled from abroad). In unregulated markets, generic SSG from Albert David, India, is available: the solution is evacuated from vials into 5 ml containers that are then sold for 3 USD each. In pharmacies, Pentostam (GSK) is sold for 100 USD per vial. No drugs for leishmaniasis are registered in Yemen.

**SOURCES OF INFORMATION**

- Dr Yasin Al Qubati, Advisor to The Minister of Health in Communicable diseases. Taiz University.
- Dr. Ali Hamoud Al Mahaqri, Ministry of Health, Yemen. *Consultative Meeting on The Control of Leishmaniasis in the African Region. WHO/AFRO Addis Ababa, 23-25 Feb 2010.*

1. [Khatri ML](http://www.ncbi.nlm.nih.gov/pubmed?term=%22Khatri%20ML%22%5BAuthor%5D), [Di Muccio T](http://www.ncbi.nlm.nih.gov/pubmed?term=%22Di%20Muccio%20T%22%5BAuthor%5D), [Gramiccia M](http://www.ncbi.nlm.nih.gov/pubmed?term=%22Gramiccia%20M%22%5BAuthor%5D) (2009). Cutaneous leishmaniasis in North-Western Yemen: a clinicoepidemiologic study and Leishmania species identification by polymerase chain reaction-restriction fragment length polymorphism analysis. [J Am Acad Dermatol.](javascript:AL_get(this,%20'jour',%20'J%20Am%20Acad%20%0d%0aDermatol.');) 61(4).

2. [Khatri ML](http://www.ncbi.nlm.nih.gov/pubmed?term=%22Khatri%20ML%22%5BAuthor%5D), [Haider N](http://www.ncbi.nlm.nih.gov/pubmed?term=%22Haider%20N%22%5BAuthor%5D), [Di Muccio T](http://www.ncbi.nlm.nih.gov/pubmed?term=%22Di%20Muccio%20T%22%5BAuthor%5D), [Gramiccia M](http://www.ncbi.nlm.nih.gov/pubmed?term=%22Gramiccia%20M%22%5BAuthor%5D) (2006) Cutaneous leishmaniasis in Yemen: clinicoepidemiologic features and a preliminary report on species identification. [Int J Dermatol](javascript:AL_get(this,%20'jour',%20'Int%20J%20%0d%0aDermatol.');) 45(1):40-5.
