# Supplementary material for: Leishmaniasis Worldwide and Global Estimates of Its Incidence
Source: PLoS One. 2012 May 31;7(5):e35671. doi: 10.1371/journal.pone.0035671 (PMC3365071; doi:10.1371/journal.pone.0035671)
Supplement: Text S101 — Leishmaniasis Country Profiles, Zambia. (DOCX) [file pone.0035671.s101.docx]

**ZAMBIA**


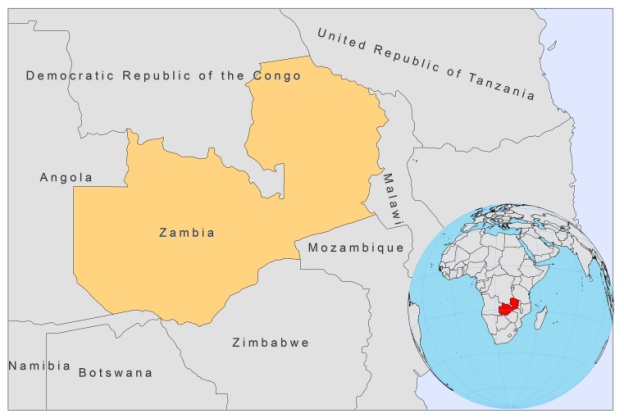


**BASIC COUNTRY DATA**

Total Population: 12,926,409

Population 0-14 years: 46%

Rural population: 64%

Population living under USD 1.25 a day: no data

Population living under the national poverty line: 59.3%

Income status: Lower middle income economy

Ranking: Low human development (ranking 164)

Per capita total expenditure on health at average exchange rate (US dollar): 61

Life expectancy at birth (years): 48

Healthy life expectancy at birth (years): 35

**BACKGROUND INFORMATION**

The first two parasitologically proven *cases* of VL were reported in 1973, from a village near Chip Ata (eastern area), and in 1976 [1], from southern Zambia (Katete). Both had cutaneous manifestations, with generalized macules, papules and nodules, without ulceration and both also had coexisting tuberculosis. No more cases have been documented. CL is unknown.

In 1997, the first case of canine leishmaniasis was reported, which necessitates further investigation of the dog as a suspect reservoir for VL in Zambia [2].

**PARASITOLOGICAL INFORMATION**

| ***Leishmania* species** | **Clinical form** | **Vector species** | **Reservoirs** |
| --- | --- | --- | --- |
| Unknown | VL | Unknown | Unknown |

No further information available on distribution, control, case management, access to care or drugs.

**SOURCES OF INFORMATION**

1. [Naik KG](http://www.ncbi.nlm.nih.gov/pubmed?term=%22Naik%20KG%22%5BAuthor%5D), [Hira PR](http://www.ncbi.nlm.nih.gov/pubmed?term=%22Hira%20PR%22%5BAuthor%5D), [Bhagwandeen SB](http://www.ncbi.nlm.nih.gov/pubmed?term=%22Bhagwandeen%20SB%22%5BAuthor%5D), [Egere JU](http://www.ncbi.nlm.nih.gov/pubmed?term=%22Egere%20JU%22%5BAuthor%5D), [Versey AA](http://www.ncbi.nlm.nih.gov/pubmed?term=%22Versey%20AA%22%5BAuthor%5D) (1976). Kala-azar in Zambia: first report of two cases. [Trans R Soc Trop Med Hyg](javascript:AL_get(this,%20'jour',%20'Trans%20R%20Soc%20Trop%20Med%20Hyg.');) 70(4):328-32.

2. Matsukawa K, Chiti L, Yoshima M, Sayer PD (1997). Onderstepoort J Canine visceral leishmaniosis: first case in Zambia Vet Res 64(1):77-9. No abstract available. PMID: 9204507.
